# One-Pot Sequential Alcohol Activation and Nickel-Catalyzed Cross-Electrophile Coupling with Chlorosilanes

**DOI:** 10.1021/acs.orglett.5c00830

**Published:** 2025-03-31

**Authors:** Xiaojie Liu, Biping Xu, Martin Oestreich

**Affiliations:** Institut für Chemie, Technische Universität Berlin, Straße des 17. Juni 115, 10623 Berlin, Germany

## Abstract

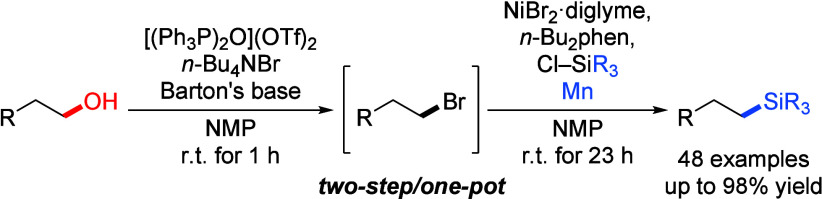

A formal deoxygenative silylation of primary alcohols is reported. The one-pot procedure consists of an *in situ* bromination of the alcohol and a subsequent nickel-catalyzed cross-electrophile coupling of the formed alkyl bromide and various vinyl-substituted chlorosilanes. The key to success is the compatibility of the nickel catalysis as well as the chlorosilane electrophile with the byproducts of the preceding bromination step, especially with triphenylphosphine oxide likely acting as a weak ligand for the excess nickel catalyst used.

Alcohols and chlorosilanes are ubiquitous, diverse, and inexpensive feedstocks.^1^ The most common interaction between them is the classical etherification reaction, typically exploited for effective protection of hydroxy groups in organic synthesis.^2^ For example, the first total synthesis of the famous marine toxin palytoxin involves a fully protected structure with 41 hydroxy groups, of which nearly half are protected as silyl ethers using chlorosilanes.^3^ Unlike the strategic use of silicon-based protecting groups,^4^ the formation of C(sp^3^)–Si bonds from alcohols and chlorosilanes is challenging and has not been reported yet (Scheme 1, top).

**Scheme 1:**
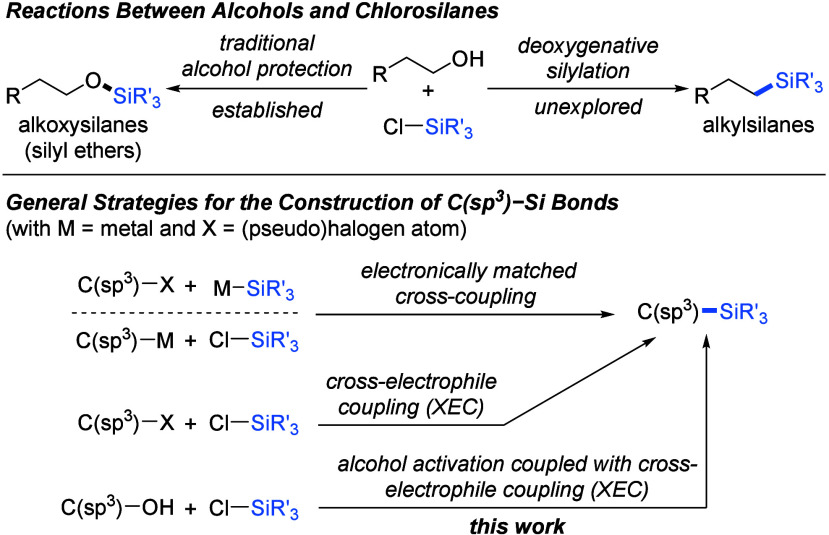
Methods for the Synthesis of Alkylsilanes by C(sp^3^)–Si Bond Formation

Traditional cross-coupling to form C(sp^3^)–Si bonds relies on the combination of two electronically matched components: either alkyl metal reagents and silicon halides or alkyl (pseudo)halides and silicon (pro)nucleophiles (Scheme 1, bottom).^5^ Only recently, the emergence of cross-electrophile coupling (XEC) opened another opportunity to introduce an alkyl group at the silicon atom from organohalides and silyl halides.^6^ In the past decade, alkyl triflates^7^ and acetates^8^ synthesized from alcohols were shown to be competent starting materials to produce alkylsilanes by us and the Shishido laboratory, respectively. However, we are not aware of any method that directly or at least in one pot converts alcohols into alkylsilanes. Given that alcohols are more available in both variety and price compared to alkyl halides,^9^ it is desirable to establish an efficient method for synthesizing alkylsilanes directly from alcohols. Such deoxygenative silylation can be regarded as a reversal of the Tamao–Fleming oxidation.^10^

Several challenges need to be properly addressed before realizing the formal deoxygenative cross-coupling of free alcohols, with the foremost being the chemoselective activation of the C(sp^3^)–O bond.^11^ While there is a substantial number of methods available to convert hydroxy groups into leaving groups,^12^ our approach would require compatibility of the required reagents with the standard setup of nickel-catalyzed XEC. Such preactivation seems necessary because the hydroxy group is likely to engage in silyl ether formation with chlorosilanes. Hence, we present here a formal nickel-catalyzed deoxygenative XEC of primary alcohols and chlorosilanes by way of *in situ* bromination of the hydroxy group.

Considering that alkyl bromides have been shown to be reliable reactants in various C(sp^3^)–Si cross-coupling reactions,^5d−5g,6b,6d−6f,13^ we hypothesized that deoxygenative silylation of alcohols could be achieved via alkyl bromides as intermediates. We therefore began our investigation with functionalized primary alcohol **1a** and chlorodimethyl(vinyl)silane (**2a**) using a two-step/one-pot strategy (Table 1). According to the literature precedent, triphenylphosphonium anhydride trifluoromethanesulfonate (POP), also known as the Hendrickson reagent, can rapidly activate the C(sp^3^)–O bond in alcohols.^14^ Encouraged by Weix’s recent work,^12d^ this easily prepared and cost-effective deoxygenating reagent was chosen as a promising candidate for this transformation. After systematic optimization of reaction parameters (see the Supporting Information for details), we found that alcohol **1a** undergoes efficient *in situ* bromination with that reagent and tetrabutylammonium bromide (TBAB) as the bromide source in the presence of 2-*tert*-butyl-1,1,3,3-tetramethylguanidine (Barton’s base) in *N*-methyl-2-pyrrolidone (NMP) as the solvent.

**Table 1:**
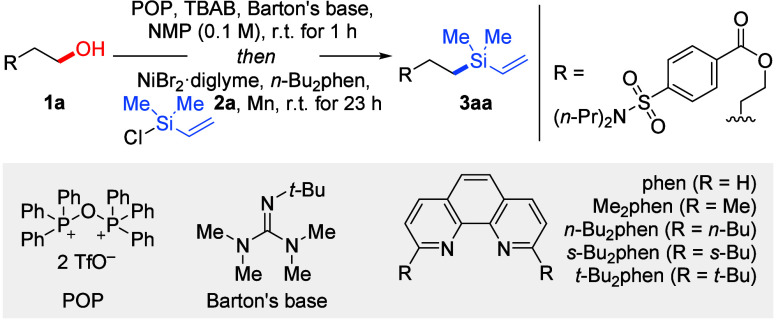
Selected Examples of Optimization^a^

entry	variation from the standard conditions	yield of product **3aa** (%)^b^
1	none	94 (91^c^)
2	without sequential addition	36
3	without NiBr_2_·diglyme	ND^d^
4	without *n*-Bu_2_phen	ND^d^
5	without POP	ND^d^
6	without Mn	ND^d^
7	bpy instead of *n*-Bu_2_phen	ND^d^
8	dtbpy instead of *n*-Bu_2_phen	ND^d^
9	phen instead of *n*-Bu_2_phen	ND^d^
10	Me_2_phen instead of *n*-Bu_2_phen	9
11	*s*-Bu_2_phen instead of *n*-Bu_2_phen	89
12	*t*-Bu_2_phen instead of *n*-Bu_2_phen	ND^d^
13	DMF instead of NMP	75
14	DMA instead of NMP	ND^d^
15	60 °C instead of room temperature	44

a Reaction conditions: compound **1a** (0.20 mmol), POP (1.5 equiv), TBAB (1.0 equiv), Barton’s base (0.20 equiv), and NMP (2.0 mL) were mixed and stirred for 1 h at room temperature, and then NiBr_2_·diglyme (12 mol %), *n*-Bu_2_phen (1.0 mol %), chlorodimethyl(vinyl)silane (**2a**, 3.0 equiv), and Mn powder (3.0 equiv) were added before the reaction was maintained at room temperature for another 23 h.

b The yield was determined by gas–liquid chromatography (GLC) analysis with methyl benzoate as an internal standard.

c Isolated yield. bpy, 2,2′-bipyridine; dtbpy, 4,4′-di-*tert*-butyl-2,2′-dipyridyl.

d ND = not detected.

In the same vessel with no purification procedure, this is followed by a nickel-catalyzed XEC with chlorosilane **2a** and manganese powder as the stoichiometric reductant. This two-step sequence afforded the desired product **3aa** in 91% yield after 24 h of total reaction time at room temperature (entry 1). Performing the reaction in a non-sequential manner resulted in a markedly lower yield of 36% (entry 2). Other bromination methods were also tested but emerged as either less effective or incompatible with the subsequent XEC. For example, the Appel reaction did furnish alkyl bromide in 95% yield, yet the nickel catalysis did only proceed sluggishly. In turn, Gong’s method^12i^ using 2-chloro-3-ethylbenzo[*d*]oxazol-3-ium tetrafluoroborate (CEBO) and TBAB was partially tolerated, affording the product **3aa** in 35% yield.

Control experiments confirmed that the nickel catalyst, ligand, activation reagent, and reducing agent are required for the reaction to proceed (entries 3–6). It is important to note that 1.0 mol % of the ligand is enough at a 12-fold excess of the nickel precatalyst (see mechanistic control experiments for an explanation). A ligand screening revealed that substituents adjacent to the nitrogen atoms of the phenanthroline backbone are essential for facilitating the reaction, while steric hindrance entirely halts the reaction (entries 7–12). Interestingly, the reaction proceeded in *N*,*N*-dimethylformamide (DMF) in a lower yet good yield but failed completely when *N*,*N*-dimethylacetamide (DMA) was used as the solvent (entries 13 and 14). Increasing the reaction temperature was detrimental, producing the product **3aa** in only a moderate yield (entry 15).

With the optimized reaction conditions in hand, we sought to explore the substrate scope of alcohols (Scheme 2). As anticipated, simple primary alcohols containing phenyl rings underwent deoxygenative silylation in excellent yields for products **3ba** and **3ca**. Product **3ba** was obtained in a slightly lower yield of 76% when compound **1b** was used on a 1.0 mmol scale. Also, 2-arylethanols prone to undergoing β-elimination to form styrenes in the presence of base yielded silylated products **3da** and **3ea** in decent yields. The reaction efficiency appears to be largely independent of the chain length, as demonstrated by the consistently reliable formation of products **3fa**–**ia**. Notably, alcohols with a remote internal double or triple bond afforded desired products **3ja** and **3ka** in high yields with π functionalities intact. Additionally, alcohols bearing diverse substituents, including a silyl ether (**3la**), an ester group (**3ma** and **3na**), a ketone (**3oa**), an amide (**3pa**), a cyano group (**3qa**), a thioether (**3ra**), and a trifluoromethyl group (**3sa**), were well-compatible with the two-step protocol. It is worth noting that halogen atoms (**3ta**–**wa** and **3ya**), a boronic ester (**3xa**), and a silyl group (**3za**) also “survived” in this nickel-catalyzed reaction in satisfactory yields. Alcohols containing a naphthalene ring (**5aa**) and various heterocycles (**5ba**–**ea**) were found to be compatible. Conversely, secondary and tertiary alcohols did not deliver any deoxygenative silylation product under the standard conditions because of competing β-elimination to the corresponding alkenes (gray box).

**Scheme 2:**
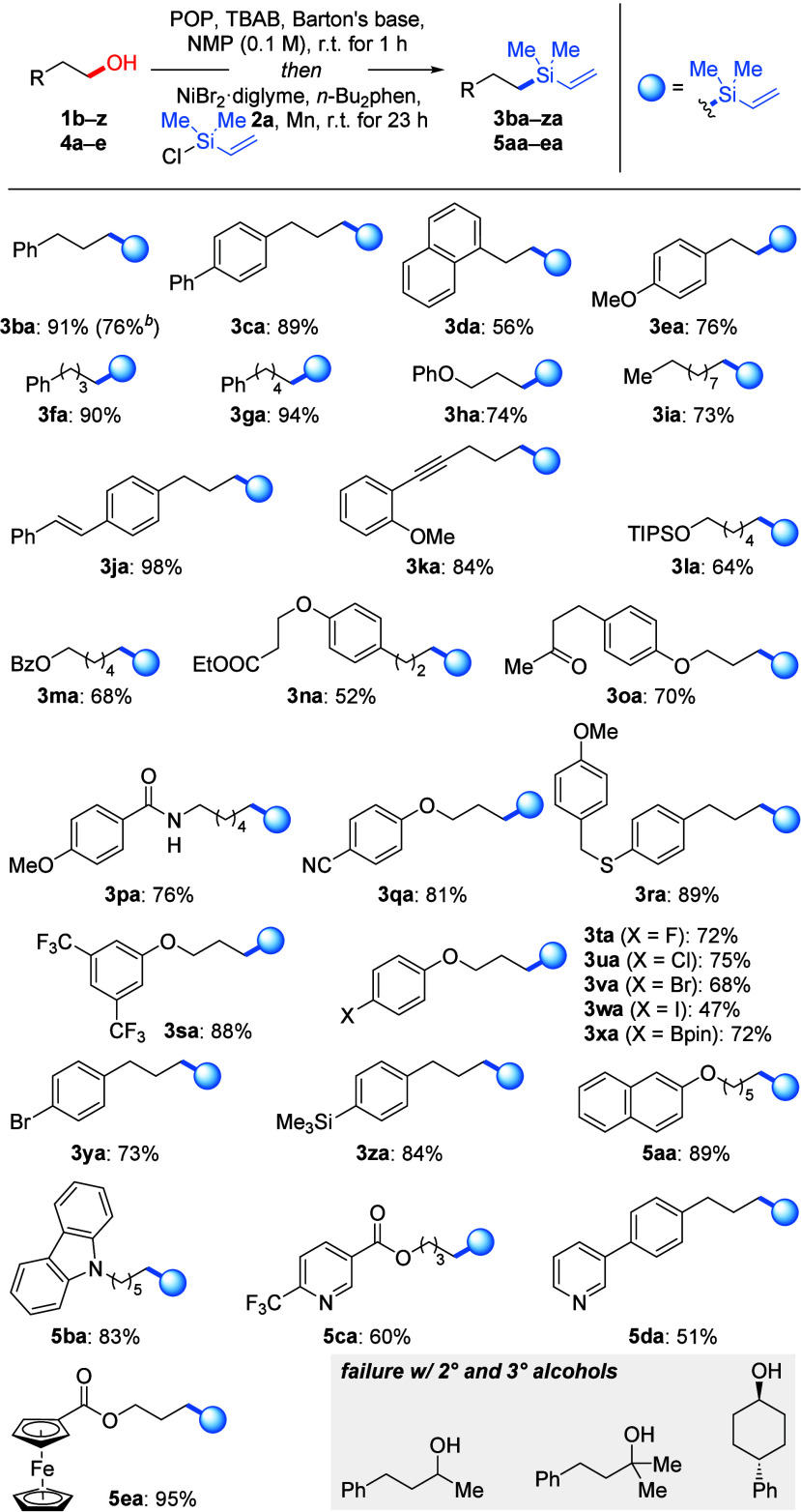
Scope I: Variation of the Alcohol Reaction conditions: alcohol **1** or **4** (0.20 mmol), POP (1.5 equiv), TBAB (1.0 equiv), Barton’s base (0.20 equiv), and NMP (2.0 mL) were mixed and stirred for 1 h at room temperature, and then NiBr_2_·diglyme (12 mol %), *n*-Bu_2_phen (1.0 mol %), chlorodimethyl(vinyl)silane (**2a**, 3.0 equiv), and Mn powder (3.0 equiv) were added before the reaction was maintained at room temperature for another 23 h. Yields are isolated after purification by flash chromatography on silica gel. Using compound **1b** on a 1.0 mmol scale.

The scope of vinyl-substituted chlorosilanes **2** was evaluated in the reaction with model compound **1a** (Scheme 3). Aryl-substituted derivatives **2b** and **2c** proved to be suitable and furnished a good yield for product **3ab** and a low yield for product **3ac**. No product **3ad** was observed with two aryl groups at the silicon atom as in compound **2d**. Trivinylchlorosilane (**2e**) reacted in a moderate yield to give product **3ae**.

**Scheme 3:**
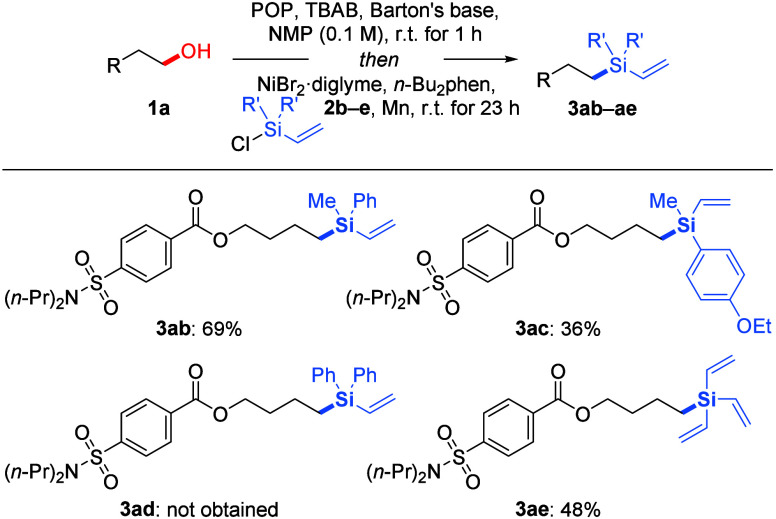
Scope II: Variation of Vinyl-Substituted Chlorosilane Reaction conditions: compound **1a** (0.20 mmol), POP (1.5 equiv), TBAB (1.0 equiv), Barton’s base (0.20 equiv), and NMP (2.0 mL) were mixed and stirred for 1 h at room temperature, and then NiBr_2_·diglyme (12 mol %), *n*-Bu_2_phen (1.0 mol %), vinyl-substituted chlorosilane **2** (3.0 equiv), and Mn powder (3.0 equiv) were added before the reaction was maintained at room temperature for another 23 h. Yields are isolated after purification by flash chromatography on silica gel.

The high functional group tolerance of this transformation encouraged us to extend the protocol to the late-stage functionalization of natural product derivatives and pharmaceutically relevant molecules (Scheme 4). A series of primary alcohol derivatives (**6a**–**m**) derived from structurally complex bioactive molecules participated in this one-pot deoxygenative silylation in high yields, showcasing the generality of the method.

**Scheme 4:**
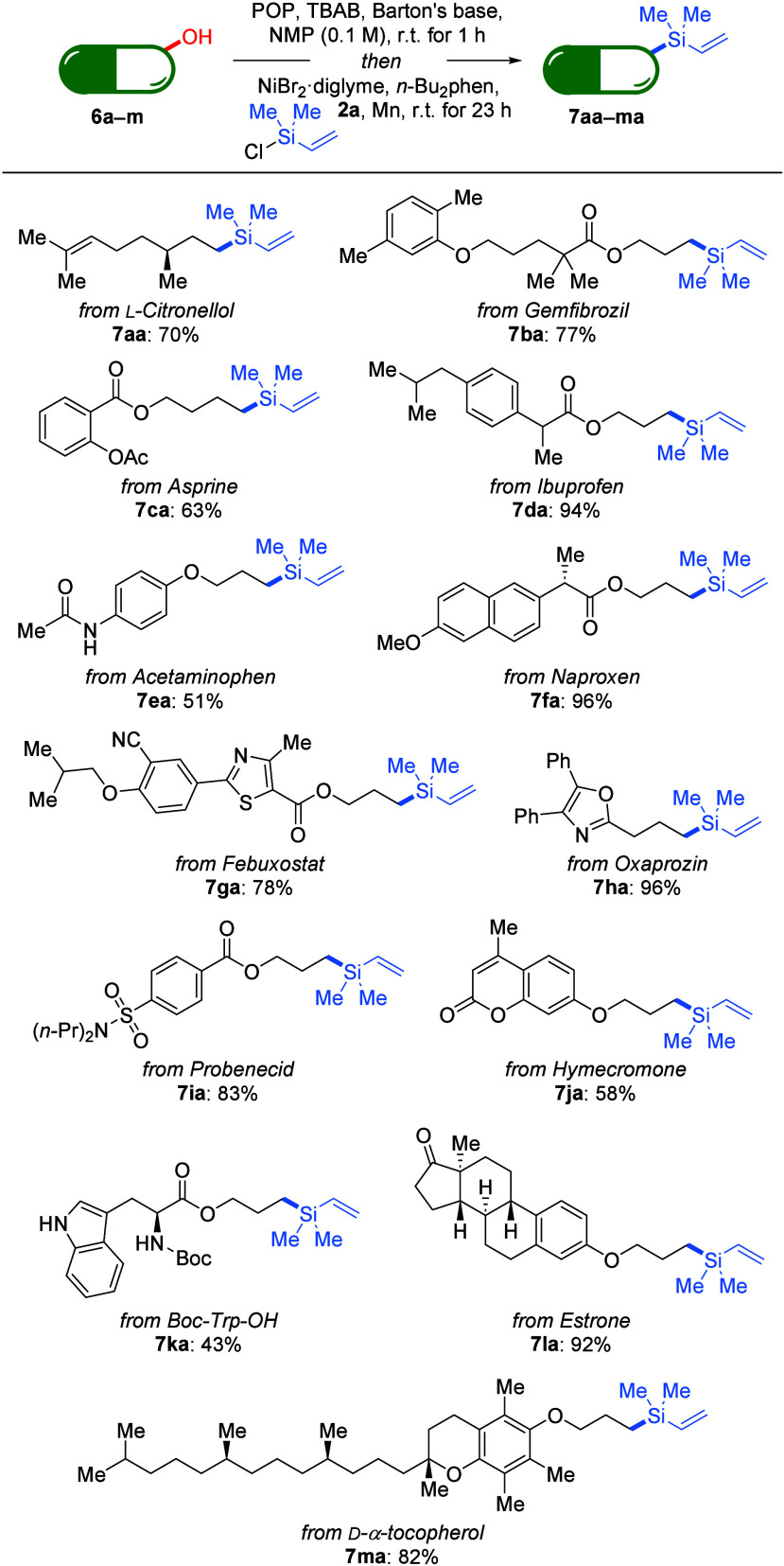
Scope III: Late-Stage Functionalization of Drug Molecules Reaction conditions: alcohol **6** (0.20 mmol), POP (1.5 equiv), TBAB (1.0 equiv), Barton’s base (0.20 equiv), and NMP (2.0 mL) were mixed and stirred for 1 h at room temperature, and then NiBr_2_·diglyme (12 mol %), *n*-Bu_2_phen (1.0 mol %), chlorodimethyl(vinyl)silane (**2a**, 3.0 equiv), and Mn powder (3.0 equiv) were added before the reaction was maintained at room temperature for another 23 h. Yields are isolated after purification by flash chromatography on silica gel.

To probe the mechanistic features of this one-pot/two-step deoxygenative silylation, three control experiments were conducted (Scheme 5). As expected, the assumed intermediate, alkyl bromide **8a**, was isolated in 91% yield when alcohol **1a** was treated with the Hendrickson reagent, ammonium bromide, and Barton’s base in NMP for 1 h. Alkyl bromide **8a** was then directly used as a starting material for the XEC with chlorosilane **2a**, and product **3aa** was isolated in 82% yield (Scheme 5, top). Also, the side product formed after deoxygenation using the POP reagent, that is, triphenylphosphine oxide, was found to be beneficial for the reaction (Scheme 5, bottom). Phosphine oxide might serve as a supporting ligand for the excess amount of the nickel precatalyst (12 mol %) relative to the phenanthroline ligand (1.0 mol %).^15^ Based on control experiments and previous literature,^6e,6f^ this deoxygenative silylation of alcohols is thought to proceed via the sequential *in situ* bromination and the previously described XEC between the resulting alkyl bromides and vinyl-substituted chlorosilanes.

**Scheme 5:**
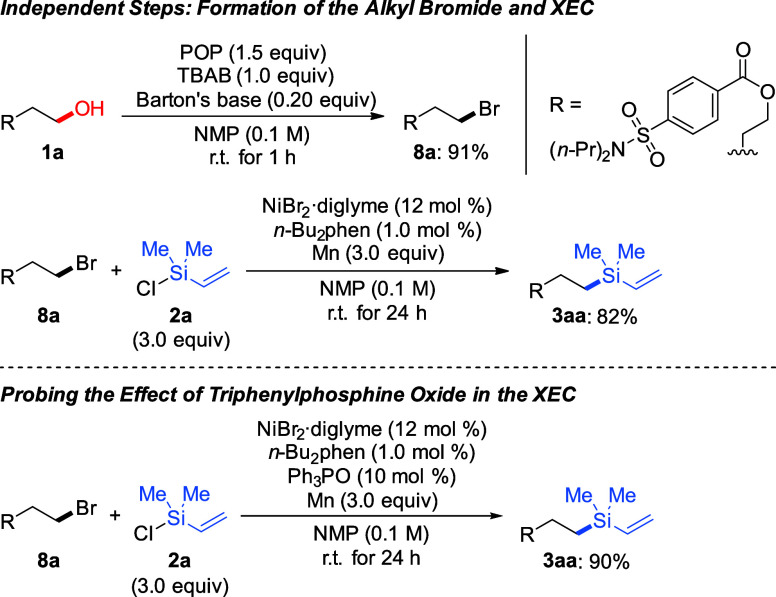
Mechanistic Control Experiments

In summary, we developed a straightforward nickel-catalyzed method for an efficient formal deoxygenative silylation of primary alcohols and chlorosilanes through the intermediacy of *in situ* generated alkyl bromides. The Hendrikson “POP” reagent is employed for the activation of the alcohol, eventually releasing triphenylphosphine oxide as a stoichiometric byproduct, which is in turn believed to act as a supporting ligand for the nickel complexes in the subsequent XEC step. The operational simplicity, the mild reaction conditions, and the superb functional group tolerance are documented by a broad range of primary alcohols.

## Data Availability

The data underlying this study are available in the published article and its Supporting Information.

## Supporting Information Available

Screening data, general procedures, experimental details, and spectral data for all new compounds (PDF)
