# Supplementary material for: One-Pot Sequential Alcohol Activation and Nickel-Catalyzed Cross-Electrophile Coupling with Chlorosilanes
Source: Org Lett. 2025 Mar 31;27(14):3686–90. doi: 10.1021/acs.orglett.5c00830 (PMC11998080; doi:10.1021/acs.orglett.5c00830)
Supplement: Supplementary file 1 — ol5c00830_si_001.pdf [file ol5c00830_si_001.pdf]

One-Pot Sequential Alcohol Activation and Nickel-Catalyzed Cross-Electrophile Coupling with Chlorosilanes

Xiaojie Liu, Biping Xu, and Martin Oestreich*

Institut für Chemie, Technische Universität Berlin

Straße des 17. Juni 115, 10623 Berlin, Germany

martin.oestreich@tu-berlin.de

Supporting Information

Table of Contents

1 General Information S3

2 Optimization Study S4

2.1 Optimization of reaction parameters S4

2.2 General procedure for deoxygenative silylation of alcohols S15

3 Syntheses of Starting Materials S16

3.1 Procedure for preparing the *n*-Bu_2phen S16

3.2 Peparation of triphenylphosphonium anhydride trifluoromethanesulfonate S17

3.3 Typical procedures for preparing alcohols from acids and acid chlorides S17

3.4 Typical procedure for preparing alcohols from phenols S21

3.5 Procedure for preparing alcohols through transition metal catalysis S22

3.6 Miscellaneous methods for the preparation alcohols S25

3.7 Syntheses of vinyl chlorosilane S27

4 Mechanistic Control Experiments S26

4.1 Validate alky bromide as possible reaction intermediate S28

4.2 Using alky bromide as starting material S28

4.3 Influence of Ph_3PO on the reductive cross-coupling process S29

5 Characterization Data of Products S28

5.1 Characterization data of starting materials S30

5.2 Characterization data of deoxygenative silylated products S55

6 NMR Spectra of Products S88

7 References S318

1 General Information

All reactions were performed in flame-dried glassware using conventional Schlenk techniques under a static pressure of nitrogen unless stated otherwise. Liquids and solutions were transferred with syringes. THF, toluene and Et_2O were distilled from metal Na under nitrogen following standard procedures; 1,4-dioxane, CH_2Cl_2, DMA, DMF, NMP and MeCN were distilled from CaH_2 under nitrogen following standard procedures; DCE and 1,2-dichlorobenzene were distilled from P_2O_5 under nitrogen following standard procedures. Technical grade solvents for extraction or chromatography (*n*-hexane, *n*-pentane, ethyl acetate, CH_2Cl_2) were distilled prior to use. All nickel catalysts, chlorosilanes, and ligands were purchased from Sigma Aldrich, TCI, ABCR, Fisher, Strem and BLD and used as received unless otherwise noted. Analytical thin layer chromatography (TLC) was performed on ALUGRAM® Xtra SIL G/UV254 TLC-Sheets by Macherey-Nagel. Flash column chromatography was performed on silica gel 60 (40-63 µm, 230-400 mesh, ASTM) by Grace using the indicated solvents. ^{1}H, ^{13}C, ^{19}F, and ^{29}Si NMR spectra were recorded in $CDCl_3$ on Bruker AV400 or AV500 instruments. Chemical shifts were reported in parts per million (ppm) and were referenced to the residual solvent resonance as the internal standard ($CHCl_3$: δ = 7.26 ppm for ^{1}H NMR and $CDCl_3$: δ = 77.00 ppm for ^{13}C NMR). All other nuclei (^{19}F and ^{29}Si) were referenced in compliance with the unified scale for NMR chemical shifts as recommended by the IUPAC stating the chemical shift relative to $BF_3 \cdot Et_2O$, CCl_3F, and Me_4Si.[S1]Data were reported as follows: chemical shift, multiplicity (br = broad signal, s = singlet, d = doublet, t = triplet, q = quartet, sept = septet, m = multiplet), coupling constants (Hz), and integration. Gas liquid chromatography (GLC) was performed on an *Agilent Technologies 7820A* gas chromatograph equipped with a HP-5 capillary column (30 m × 0.32 mm, 0.25 µm film thickness) by *Agilent Technologies/CS-Chromatographie Service* using the following program: N_2 carrier gas, injection temperature 250 °C, detector temperature 300 °C, flow rate: 1.7 mL/min; temperature program: start temperature 40 °C, heating rate 10 °C/min, end temperature 280 °C for 10 min. Structural assignments were made with additional information from gCOSY, gHSQC, and gHMBC experiments. Infrared (IR) spectra were recorded on an *Agilent Technologies Cary* 630 FT-IR spectrometer equipped with an ATR unit and the signals were reported in wave-numbers (cm^{-1}). Melting points (M.p.) were determined with a Stuart Scientific SMP20 melting point apparatus and were not corrected. High resolution mass spectra (HRMS) were obtained from the Analytical Facility at the *Institut für Chemie, Technische Universität Berlin* on a Thermo Fisher Scientific LTQ Orbitrap XL apparatus using APCI techniques with a linear ion trap analyzer.

2 Optimization Study

2.1 Optimization of reaction parameters

General procedure for the initial optimization reactions:

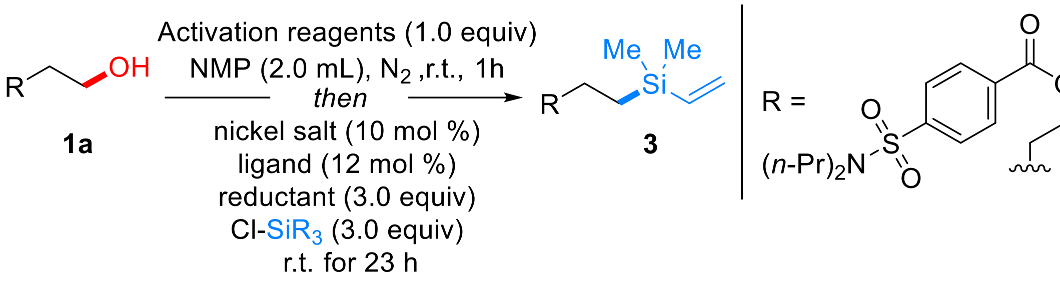

In a nitrogen-filled glovebox, a 35 mL pressure tube equipped with a stir bar was charged with 4-hydroxybutyl 4-(*N*,*N*-dipropylsulfamoyl)benzoate (72 mg, 0.20 mmol), alcohol activation reagents (0.20 mmol), then *N*-methyl-2-pyrrolidone (NMP) (2.0 mL) was added to the pressure tube *via* syringe. After the reaction mixture was stirred at room temperature for 1 hour, and then nickel salt (20 umol, 10 mol %), ligand (24 umol, 12 mol %), reductant (0.60 mmol, 3.0 equiv) and chlorosilane (**2**) (0.60 mmol, 3.0 equiv) was added before the reaction mixture was stirred at room temperature for another 23 hours. After the indicated reaction time, the mixture was diluted with ethyl acetate (10 mL) and washed with water. The organic phase was then dried over anhydrous Na_2SO_4, filtered, and concentrated *in vacuo*. The residue was dissolved with 1.0 mL ethyl acetate and subjected to GLC analysis with methyl benzoate as the internal standard.

Table S1. Screening of alcohol activation reagents.[a]

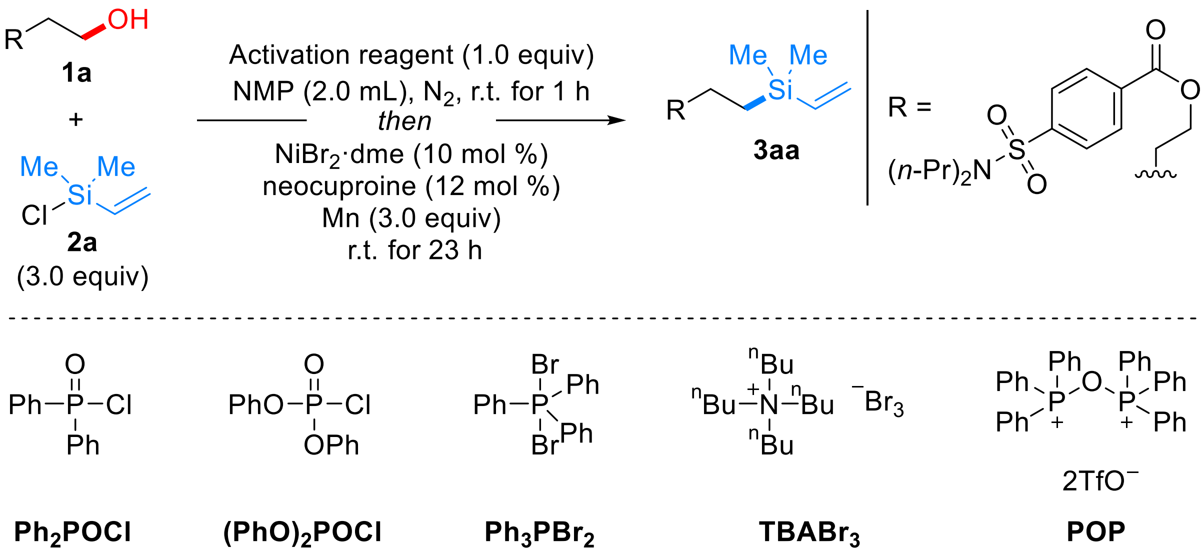

entry	alcohol activation reagent	yield of **3aa** (%)[b]
1	Ph_2POCl (1.0 equiv)	N.D.
2	$(PhO)_2POCl$ (1.0 equiv)	N.D.
3	Ph_3PBr_2 (1.0 equiv)	5
4	Ph_3P (1.0 equiv) + $TBABr_3$ (1.0 equiv)	15
5	POP (1.0 equiv)	19

[a]All reactions were performed on a 0.20 mmol scale. [b]Yield was determined by GLC analysis with methyl benzoate as an internal standard. N.D. = Not detected.

Table S2. Screening of chlorosilanes[a]

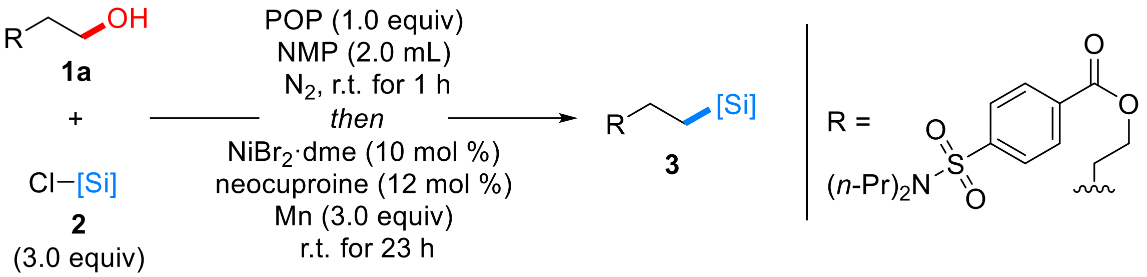

entry	chlorosilane	yield of **3** (%)[b]
1	Me_3SiCl	N.D.
2	Et_3SiCl	N.D.
3	$PhMe_2SiCl$	N.D.
4	$Ph_2MeSiCl$	N.D.
5	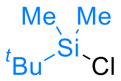	N.D.
6	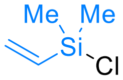	19
7	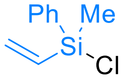	18

[a]All reactions were performed on a 0.20 mmol scale. [b]Yield was determined by GLC analysis with methyl benzoate as an internal standard. N.D. = Not detected.

Table S3. Screening of solvents[a]

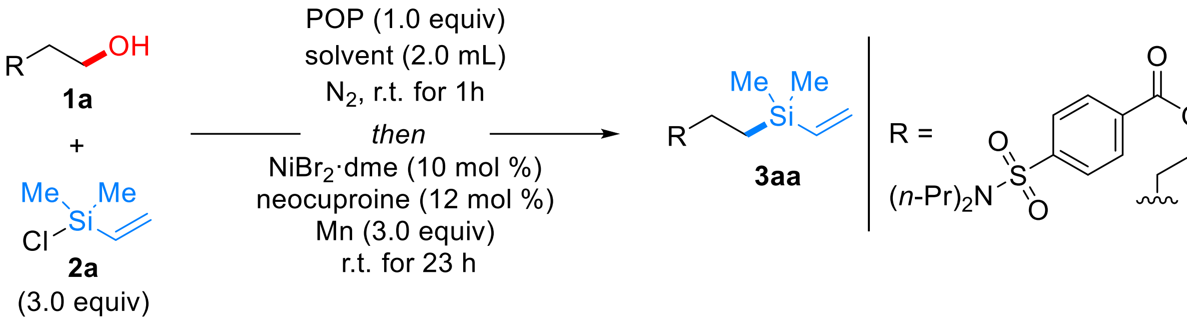

entry	solvent	yield of **3aa** (%)[b]
1	THF	N.D.
2	1,4-dioxane	N.D.
3	toluene	N.D.
4	1,2-dichlorobenzene	N.D.
5	DCM	N.D.
6	DCE	N.D.
7	MeCN	N.D.
8	DMF	14
9	DMA	N.D.

[a]All reactions were performed on a 0.20 mmol scale. [b]Yield was determined by GLC analysis with methyl benzoate as an internal standard. DCE = 1,2-dichloroethane, DMF **=** *N*,*N*-dimethylformamide, DMA = *N*,*N*-dimethylacetamide

Table S4. Screening of nickel salts[a]

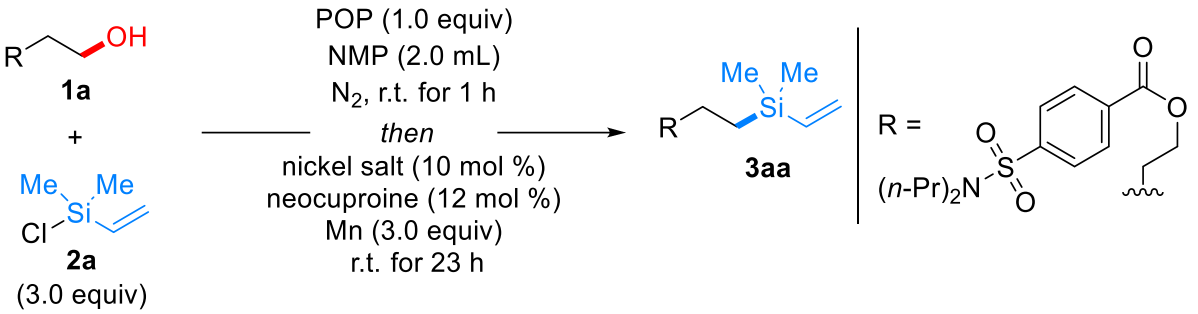

entry	nickel salt (10 mol %)	yield of **3aa** (%)[b]
1	$Ni(COD)_2$	N.D.
2	$NiCl_2$	N.D.
3	$NiBr_2$	14
4	NiI_2	21
5	$Ni(OTf)_2$	N.D.
6	$NiBr_2$·dme	19
7	$NiBr_2$·diglyme	24
8	$Ni(PPh_3)_2Cl_2$	N.D.

[a]All reactions were performed on a 0.20 mmol scale. [b]Yield was determined by GLC analysis with methyl benzoate as an internal standard.

Table S5. Screening of halogen source[a]

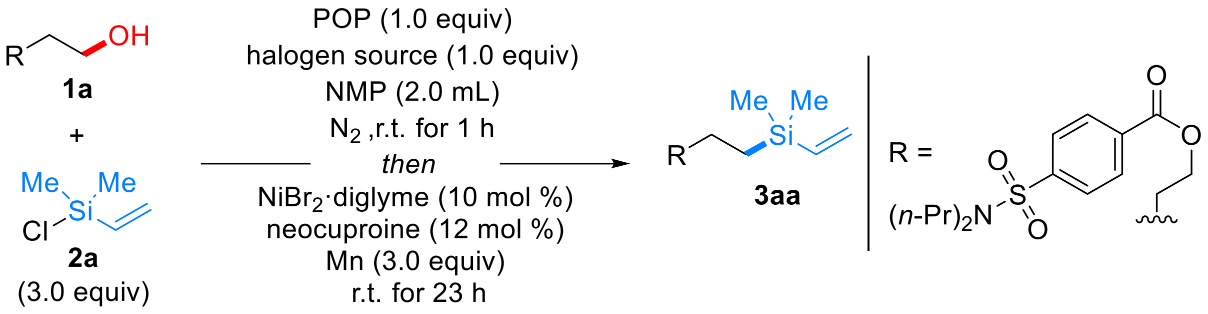

entry	halogen source	yield of **3aa** (%)[b]
1	TBAC	5
2	TBAB	35
3	TBAI	28
4	LiBr	10
5	NaBr	31
6	KBr	32
7	CsBr	18
8	$ZnBr_2$	16
9	$MgBr_2$	9
10	LiI	19
11	NaI	21
12	KI	23

[a]All reactions were performed on a 0.20 mmol scale. [b]Yield was determined by GLC analysis with methyl benzoate as an internal standard. TBAC = tetrabutylammonium chloride, TBAB = tetrabutylammonium bromide, TBAI = tetrabutylammonium iodide.

Table S6. Screening of bases[a]

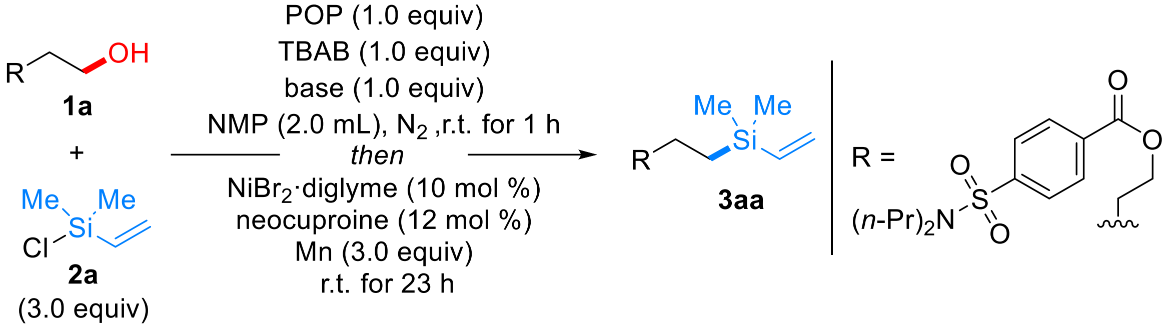

entry	base	yield of **3aa** (%)[b]
1	Et_3N	27
2	DIPEA	29
3	DMAP	23
4	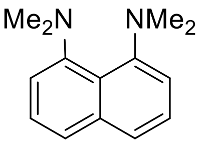	25
5	Cy_2NMe	N.D.
6	DBACO	21
7	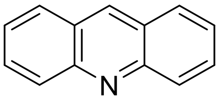	22
8	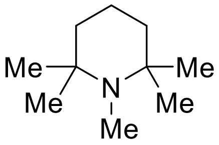	N.D.
9	Cs_2CO_3	N.D.
10	K_3PO_4	N.D.
11	KF	21
12	Barton's base	36
13	Barton's base (0.20 equiv)	41
14[c]	Barton's base (0.20 equiv)	45

[a]All reactions were performed on a 0.20 mmol scale. [b]Yield was determined by GLC analysis with methyl benzoate as an internal standard. [c]POP 1.5 equivalents were used instead of 1.0 equivalents. DABCO = 1,4-diazabicyclo [2.2.2] octane, DIPEA = *N*,*N*-diisopropylethylamine, DMAP = 4-(dimethylamino)pyridine.

Table S7. Screening of ligands[a]

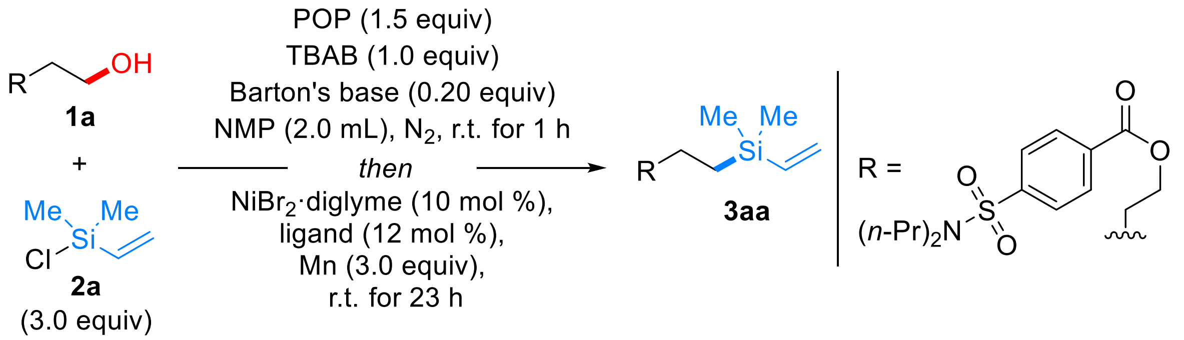

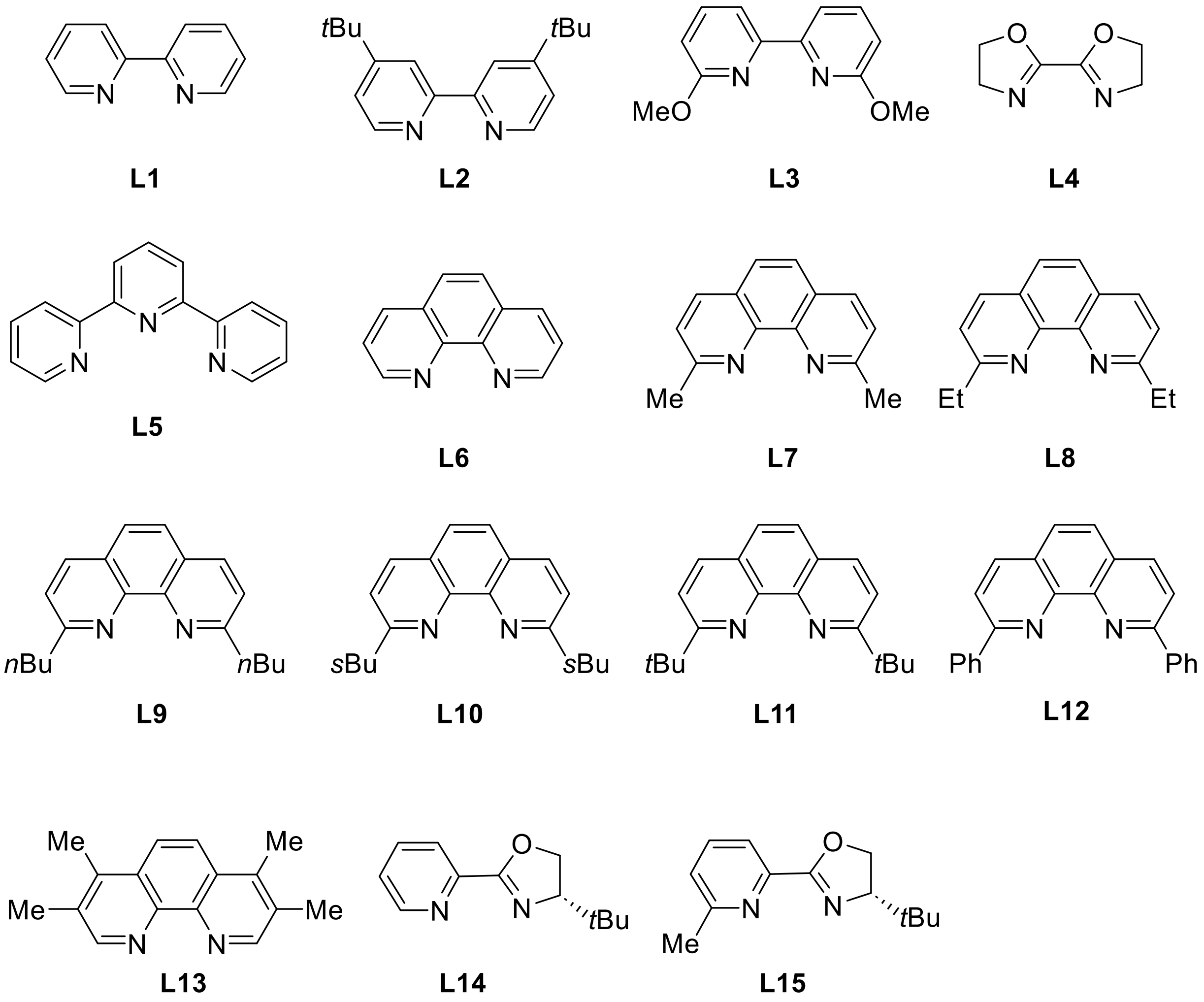

entry	ligand	yield of **3aa** (%)[b]
1	**L1**	N.D.
2	**L2**	N.D.
3	**L3**	N.D.
4	**L4**	N.D.
5	**L5**	N.D.
6	**L6**	N.D.
7	**L7**	45
8	**L8**	47
9	**L9**	60
10	**L10**	59
11	**L11**	N.D.
12	**L12**	N.D.
13	**L13**	N.D.
14	**L14**	54
15	**L15**	59
16	PPh_3	N.D.
17	PCy_3	N.D.
18	dppe	N.D.
19	dppb	N.D.
20	dppf	N.D.

[a]All reactions were performed on a 0.20 mmol scale. [b]Yield was determined by GLC analysis with methyl benzoate as an internal standard.

Table S8. Screening of ligands loading[a]

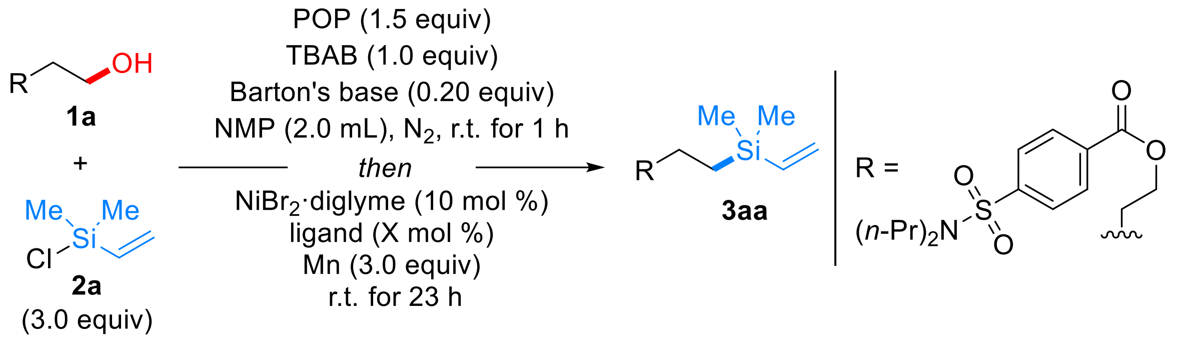

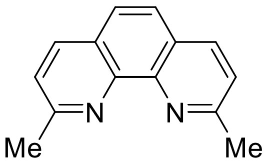

L7

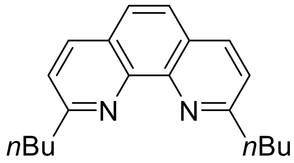

L9

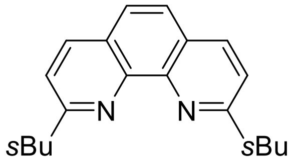

L10

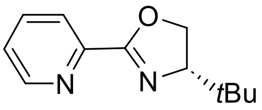

L14

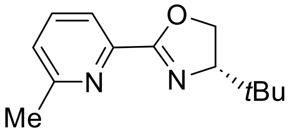

L15

entry	ligand loading	yield of **3aa** (%)[b]
1	**L9** (9 mol %)	59
2	**L9** (6 mol %)	63
3	**L9** (3 mol %)	85
4	**L9** (1 mol %)	89
5	**L7** (1 mol %)	9
6	**L10** (1 mol %)	89
7	**L14** (1 mol %)	N.D.
8	**L15** (1 mol %)	N.D.
9[c]	**L9** (1 mol %)	94

[a]All reactions were performed on a 0.20 mmol scale. [b]Yield was determined by GLC analysis with methyl benzoate as an internal standard. [c]12 mol % $NiBr_2$·diglyme was used.

Table S9. Screening of alternative reductant[a]

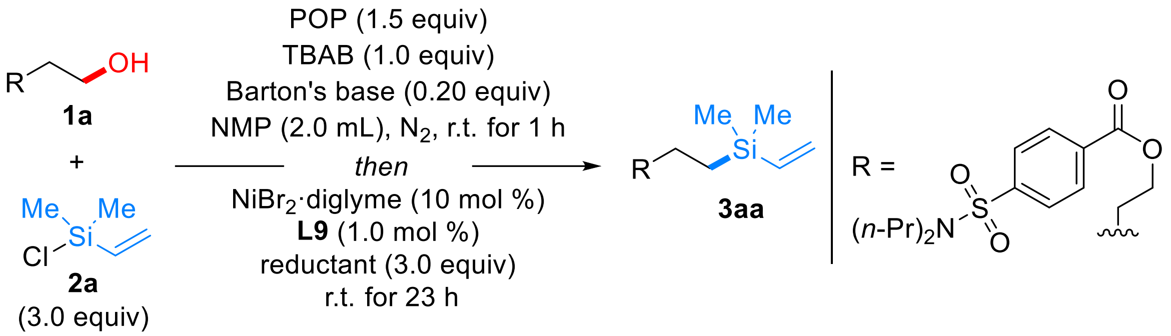

entry	reductant	yield of **3aa** (%)[b]
1	Zn powder	11
2	Mg powder	N.D.
3	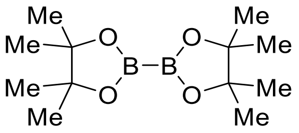	N.D.
4	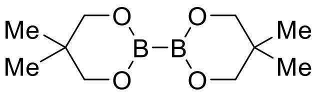	N.D.
5	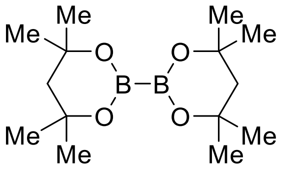	N.D.
6	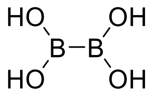	N.D.
7	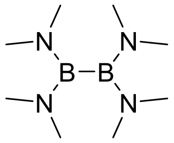	N.D.
8	$PhSiH_3$	N.D.
9	Et_3SiH	N.D.
10	Poly(methylhydrosiloxan)	N.D.
11	$(MeO)_3SiH$	N.D.
12	$(EtO)_3SiH$	N.D.
13	$(MeO)_2MeSiH$	N.D.
14	$(EtO)_2MeSiH$	N.D.

[a]All reactions were performed on a 0.20 mmol scale. [b]Yield was determined by GLC analysis with methyl benzoate as an internal standard.

2.2 General procedure (GP) for deoxygenative silylation of alcohols

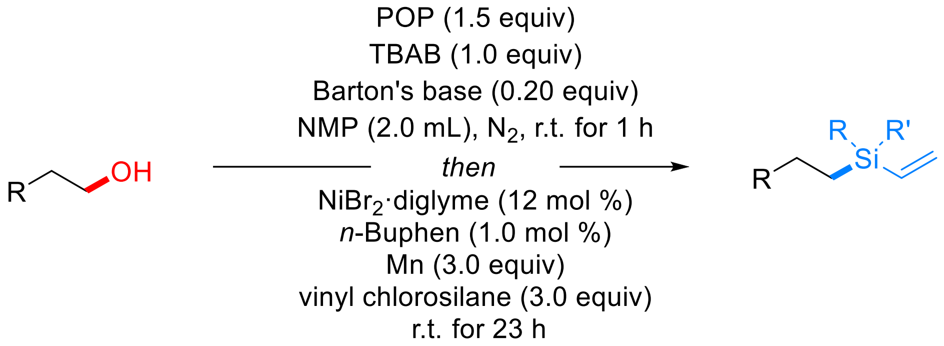

In a nitrogen-filled glovebox, a 35 mL pressure tube equipped with a stir bar was charged with alcohol (0.20 mmol), POP (252 mg, 0.30 mmol), Barton's base (6.8 mg, 40 umol) and tetrabutylammonium bromide (64 mg, 0.20 mmol), then NMP (2.0 mL) was added to the pressure tube via syringe. After the reaction mixture was stirred at room temperature for 1 hour, and then nickel (II) bromide 2-methoxyethyl ether ($NiBr_2$·diglyme) (8.4 mg, 24 umol), 2,9-dibutyl-1,10-phenanthroline (0.58 mg, 2.0 umol), vinyl chlorosilane (72 mg, 0.60 mmol) and manganese powder (33 mg, 0.60 mmol) was added before the reaction mixture was stirred at room temperature for another 23 hours. After the indicated reaction time, the mixture was diluted with ethyl acetate (10 mL) and washed with water. The organic phase was then dried over anhydrous Na_2SO_4, filtered, and concentrated *in vacuo*. The residue was dissolved with 1.0 mL ethyl acetate and subjected to GLC analysis with methyl benzoate as the internal standard.

3 Syntheses of Starting Materials

3.1 Procedure for preparing *n*-Bu_2phen

Method A

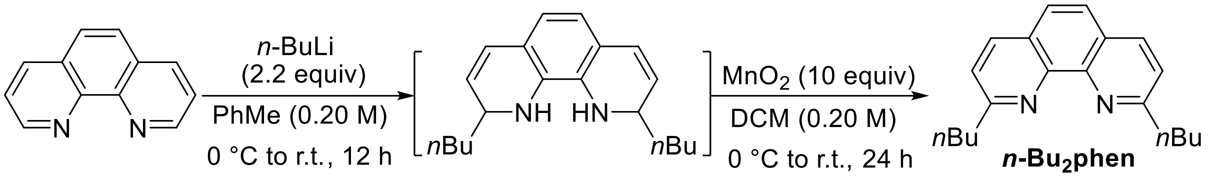

Step 1: According to the literature[S2], in a nitrogen-filled Schlenk tube with a rubber septum was charged with 1,10-phenanthroline (3.6 g, 20 mmol) and toluene 100 mL. After cooling to 0 °C with ice bath, 18 mL of *n*-butyllithium (44 mmol, 2.5 mol/L in *n*-hexane) was dropwise added to the solution via syringe through a rubber septum over a period of 2 hours, the color of reaction mixture changed into wine red and yellow upon the dropwise addition. Later, the reaction mixture was stirred at room temperature for 12 hours. After the indicated reaction time, it was quenched with saturated NH_4Cl solution and extracted with DCM twice. Then the combined organic layer was dried with anhydrous Na_2SO_4, followed by evaporation under reduced pressure to give out yellow oil, which was subsequently used in the next step without further purification.

Step 2: In a 250 mL round-bottom flask equipped with a Teflon-coated magnetic stir bar was charged with yellow oil obtained in last step, then anhydrous dichloromethane 50 mL was added. The reaction was cooled down to 0 °C with ice bath. To this mixture was portionwise added activated MnO_2 (18 g, 200 mmol). After addition, the solution was allowed to warm to room temperature and stirred for another 24 hours. After the indicated reaction time, the reaction mixture was filtered through a pad of silica gel and washed with DCM. The filtrate was concentrated under reduced pressure and the residue was purified by flash chromatography on silica gel (eluent = *n*-pentane:ethyl acetate = 5:1) to obtain the ligand *n*-Bu_2phen.

3.2 Peparation of triphenylphosphonium anhydride trifluoromethanesulfonate

Method B

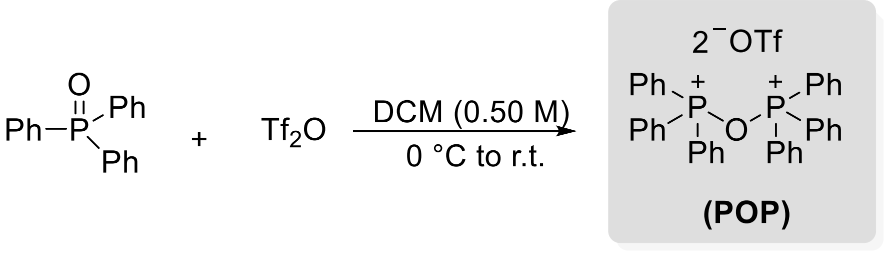

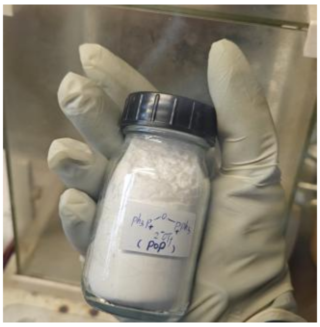

According to the literature[S3], to a 500 mL nitrogen filled round-bottom flask equipped with a Teflon-coated magnetic stir bar was added triphenylphosphine oxide (27.8 g, 100 mmol), then anhydrous DCM (200 mL) was added via syringe through a rubber septum. After the solution was cooled to 0 °C with ice bath, 17.6 mL of trifluoromethanesulfonic anhydride (Tf_2O) (18.4 mL, 30.9 g, 110 mmol) was dropwise added to the solution via syringe through a rubber septum over a period of 2 h, upon which a white precipitate was observed. Then, the solution was allowed to warm to room temperature and stirred overnight. After the indicated reaction time, the reaction mixture was filtered under nitrogen atmosphere and the precipitated solid was washed with anhydrous DCM (2 x 50 mL). The solid was collected and dried *in vacuo* to afford the triphenylphosphonium anhydride trifluoromethanesulfonate as white powder.

3.3 Typical procedures for preparing alcohols from acids and acid chlorides

3.3.1 Preparation of alcohols through in-situ generation of acid chlorides

Method C

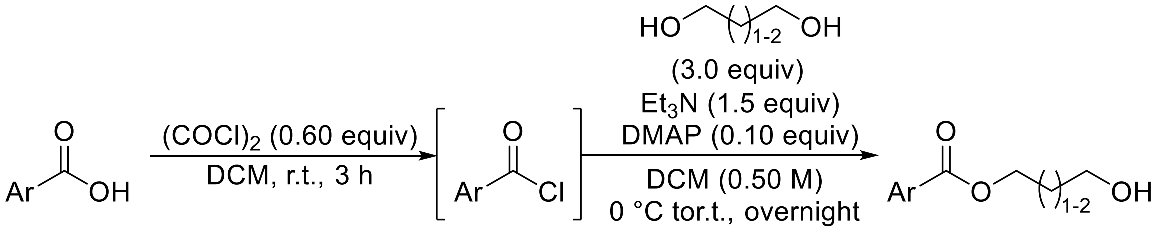

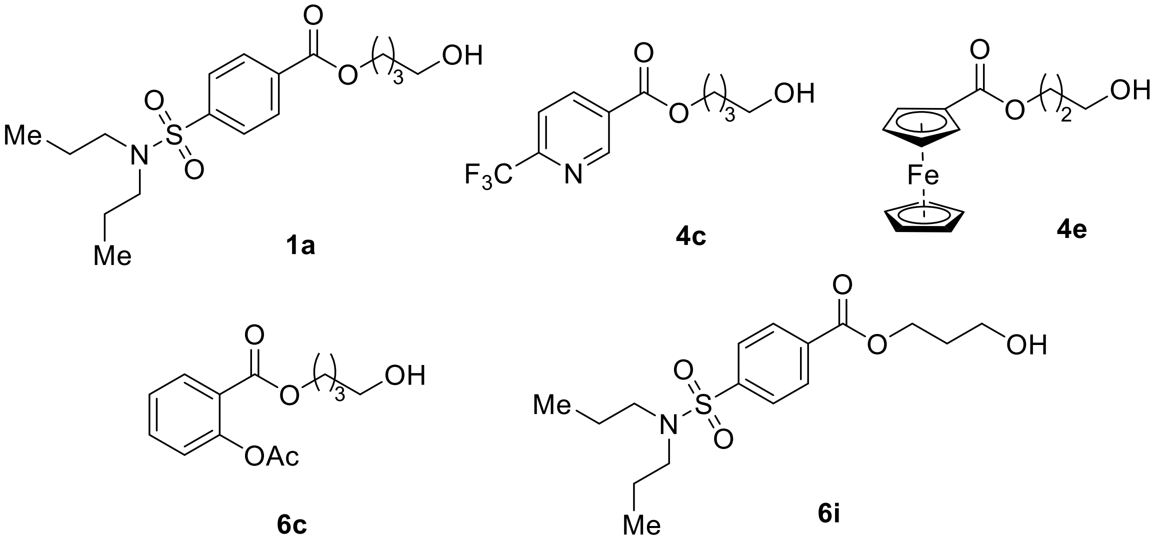

1a **4c** **4e**

6c **6i**

Step 1: To a 100 mL round-bottom flask equipped with a Teflon-coated magnetic stir bar was added carboxylic acid (10 mmol), then dry DCM 20 mL and two drops of DMF were added. To this solution was dropwise added oxalyl chloride (0.76 g, 6.0 mmol) over 15 min via syringe, during which vigorous bubbles were emitted from the reaction mixture. Upon finishing addition, the solution was stirred at room temperature for 3 hours. After the reaction was judged complete (stop bubbling), the reaction mixture was directly concentrated under reduced pressure carefully.

Step 2: To a 100 mL round-bottom flask equipped with a Teflon-coated magnetic stir bar was added 1,3-diol (2.3 g, 30 mmol) or 1,4-diol (2.7 g, 30 mmol), DMAP (0.12 g, 1.0 mmol) and Et_3N (1.5 g, 15 mmol). Then anhydrous dichloromethane 20 mL was added. The reaction was cooled down to 0 °C with ice bath. To this mixture was added the freshly prepared acid chloride in anhydrous dichloromethane (20 mL). After addition, the solution was allowed to warm to room temperature and stirred overnight. After the indicated reaction time, H_2O (50 mL) was added to the reaction mixture before it was extract with DCM (2 × 30 mL), washed with saturated $NaHCO_3$, 1N aqueous HCl solution, brine. Then the combined organic layer was dried with anhydrous Na_2SO_4 and concentrated under reduced pressure to remove the solvent. The residue was purified by flash chromatography on silica gel (eluent = *n*-pentane:ethyl acetate = 10:1) to obtain ester products.

3.3.2 Preparation of alcohols through condensation reagents

Method D

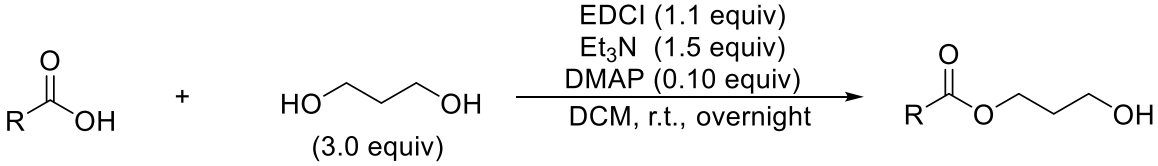

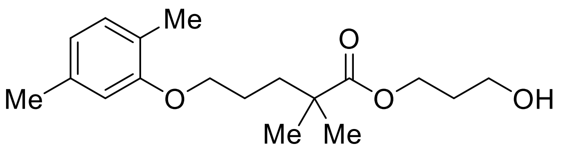

6b

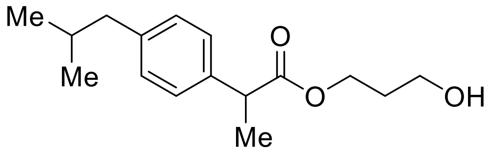

6d

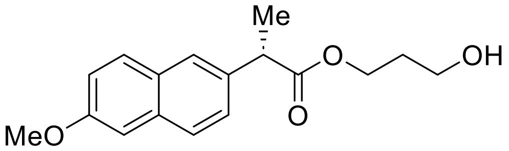

6f

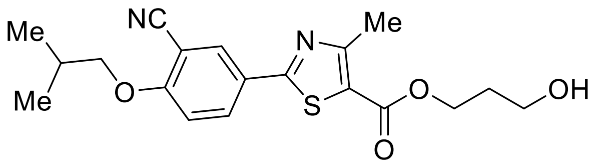

6g

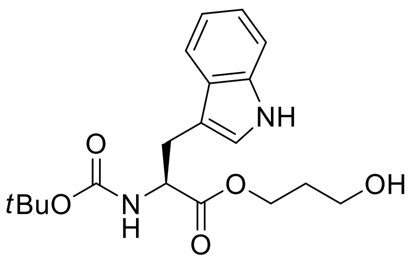

6k

To a 250 mL round-bottom flask equipped with a Teflon-coated magnetic stir bar was added carboxylic acid (10 mmol), propane-1,3-diol (2.3 g, 30 mmol), Et_3N (1.5 g, 15 mmol), DMAP (0.12 g, 1.0 mmol) and *N*-(3-dimethylaminopropyl)-*N*'-ethyl carbodiimide hydrochloride (EDCI) (2.1 g, 11 mmol), then anhydrous dichloromethane 50 mL was added and the reaction mixture was stirred overnight at room temperature. After the reaction was judged complete by TLC, the reaction solvent was carefully removed under reduced pressure. Later, 50 mL ethyl acetate was added to the reaction mixture before it was washed with water, saturated $NaHCO_3$, 1N aqueous HCl solution, brine. Then the collected organic layer was dried with anhydrous Na_2SO_4 and concentrated under reduced pressure to remove the solvent. The residue was purified by flash chromatography on silica gel (eluent = *n*-pentane:ethyl acetate = 10:1) to obtain ester products.

3.3.3 Preparation of alcohols from acid chlorides

Method E

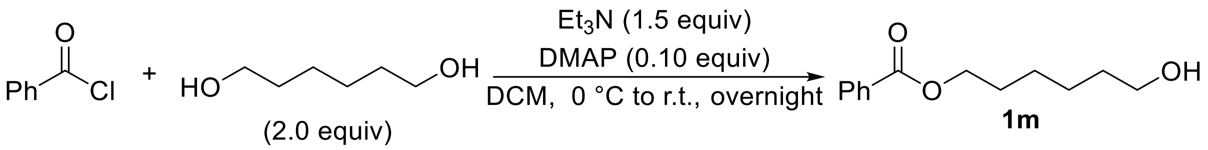

To a 100 mL round-bottom flask equipped with a Teflon-coated magnetic stir bar was added hexane-1,6-diol (2.4 g, 20 mmol), Et_3N (1.5 g, 15 mmol) and DMAP (0.12 g, 1.0 mmol), then dry DCM 50 mL was added and the reaction was cooled down to 0 °C with ice bath. To this mixture was dropwise added benzoyl chloride (1.4 g, 10 mmol in DCM 10 mL) over 10 min via syringe. After addition, the solution was allowed to warm to room temperature and stirred overnight. The reaction mixture was quenched with water (50 mL) and washed with water, saturated $NaHCO_3$, 1N aqueous HCl solution, brine. Then the collected organic layer was dried with anhydrous Na_2SO_4 and concentrated under reduced pressure to remove the solvent. The residue was purified by flash chromatography on silica gel (eluent = *n*-pentane:ethyl acetate = 4:1) to obtain ester products.

Method F

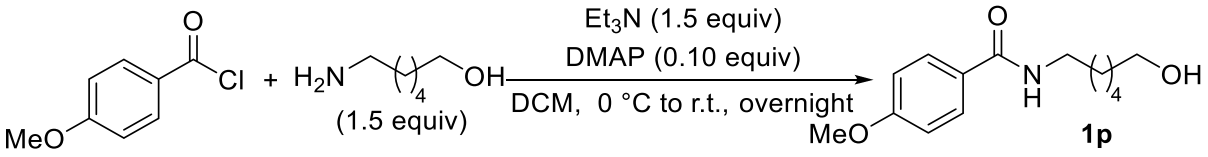

To a 100 mL round-bottom flask equipped with a Teflon-coated magnetic stir bar was added 6-aminohexan-1-ol (1.8 g, 15 mmol), Et_3N (1.5 g, 15 mmol) and DMAP (0.12 g, 1.0 mmol), then dry DCM 50 mL was added and the reaction was cooled down to 0 °C with ice bath. To this mixture was dropwise added 4-methoxybenzoyl chloride (1.7 g, 10 mmol in DCM 10 mL) over 10 min via syringe. After addition, the solution was allowed to warm to room temperature and stirred overnight. The reaction mixture was quenched with water (50 mL) and washed with water, saturated $NaHCO_3$, 1N aqueous HCl solution, brine. Then the collected organic layer was dried with anhydrous Na_2SO_4 and concentrated under reduced pressure to remove the solvent. The residue was purified by flash chromatography on silica gel (eluent = *n*-pentane:ethyl acetate = 4:1) to obtain amide products.

3.4 Typical procedure for preparing alcohols from phenols

Method G

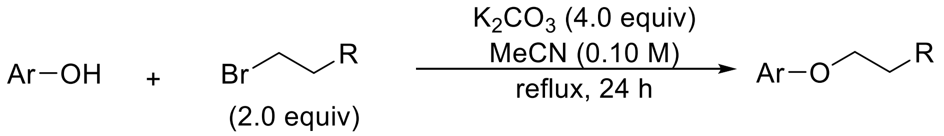

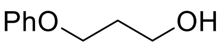
1h

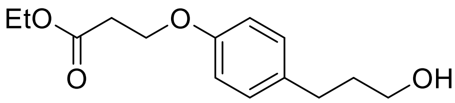
1n

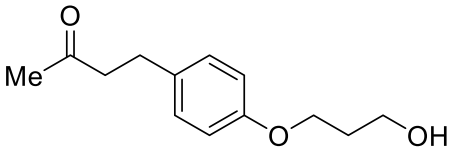
1o

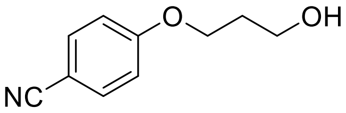
1q

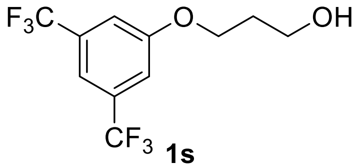
1s

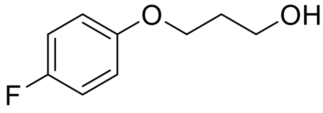
1t

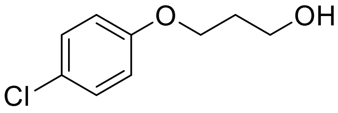
1u

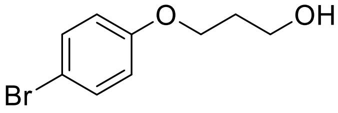
1v

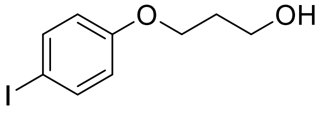
1w

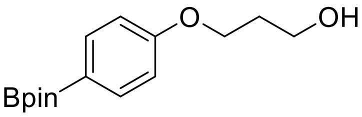
1x

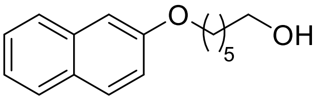
4a

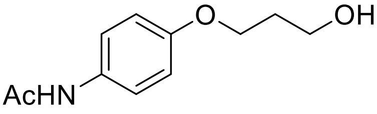
6e

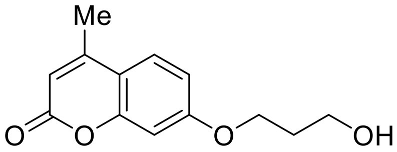
6j

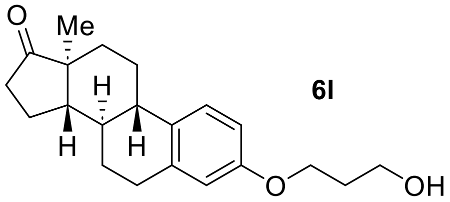
6l

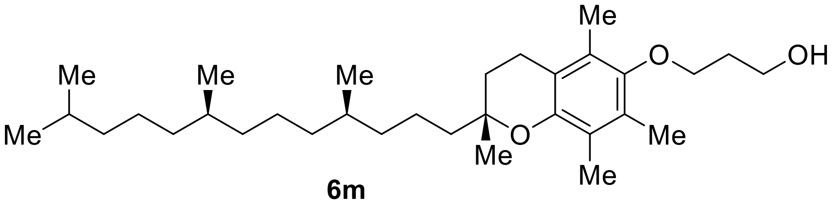
6m

To a 100 mL nitrogen filled round-bottom flask equipped with a Teflon-coated magnetic stir bar was added phenol (10 mmol), 3-bromopropan-1-ol (2.8 g, 20 mmol) and K_2CO_3 (5.5 g, 40 mmol), then MeCN (50 mL) was added *via* syringe before the reaction mixture was allowed to heat to reflux in an oil bath for 24 hours. After the indicated reaction time, the reaction mixture was cooled to room temperature, filtered through a pad of silica gel and washed with ethyl

acetate. The filtrate was concentrated under reduced pressure and the residue was purified by flash chromatography on silica gel (eluent = *n*-pentane:ethyl acetate = 5:1) to obtain the product.

3.5 Procedure for preparing alcohols through transition metal catalysis

Method H

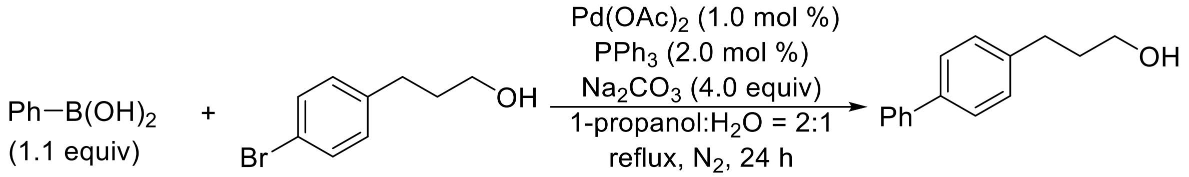

1c

According to the literature[S4], the pressure tube was charged with 3-(4-bromophenyl)propan-1-ol (2.2 g, 10 mmol), phenylboronic acid (1.3 g, 11 mmol), $Pd(OAc)_2$ (22 mg, 0.10 mmol), PPh_3 (52 mg, 0.20 mmol), Na_2CO_3 (4.2 g, 40 mmol). Then 1-propanol (66 mL) and H_2O (33 mL) were added via syringe through a rubber septum, and then the tube was sealed with a Teflon screwcap under nitrogen flow. The reaction mixture was stirred at reflux in an oil bath for 24 hours. After the reaction mixture was cooled to room temperature, the reaction mixture was filtered through a pad of silica gel and washed with ethyl acetate. The filtrate was concentrated under reduced pressure and the residue was purified by flash chromatography on silica gel (eluent = *n*-pentane:ethyl acetate = 10:1) to obtain the Suzuki coupling product.

Method I

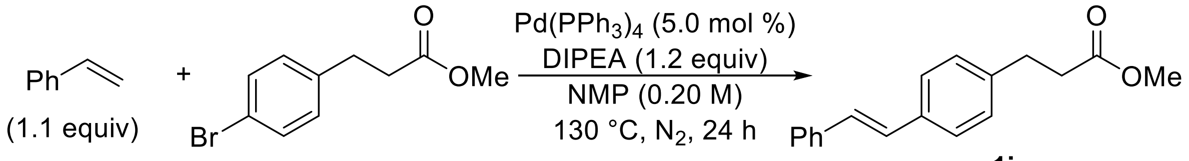

1j-s

Step 1: According to the literature[S5], in the nitrogen-filled pressure tube was charged with methyl 3-(4-bromophenyl)propanoate (2.4 g, 10 mmol), styrene (1.1 g, 11 mmol), $Pd(PPh_3)_4$ (0.29 g, 0.50 mmol), DIPEA (1.6 g, 12 mmol). Then anhydrous NMP (50 mL) were added via syringe through a rubber septum, and then the tube was sealed with a Teflon screwcap under nitrogen flow. The reaction mixture was stirred at 130 °C in an oil bath for 24 hours. After the reaction mixture was cooled to room temperature, the reaction mixture was diluted with ethyl acetate (10 mL) and washed with water twice, brine. The organic phase was collected, dried

over anhydrous Na_2SO_4 and concentrated under reduced pressure. The residue was purified by flash chromatography on silica gel (eluent = *n*-pentane:ethyl acetate = 95:5) to obtain the product **1j-s**.

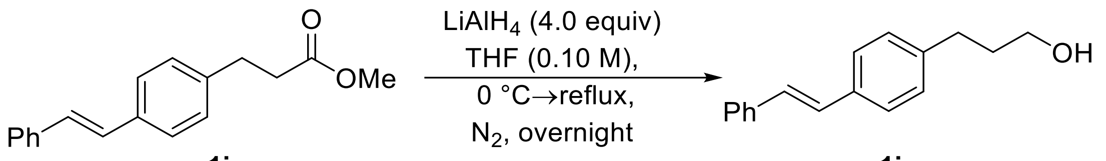

1j-s **1j**

Step 2: According to the literature, to a 100 mL flame-dried round-bottom Schlenk flask charged with a Teflon-coated magnetic stir bar and lithium aluminum hydride ($LiAlH_4$) (0.38 g, 10 mmol) was added dry THF 50 mL. When the solution was cooled to 0 °C with ice bath, methyl (*E*)-3-(4-styrylphenyl)propanoate (1.1 g, 4.0 mmol) was potion wise added over 30 minutes under a nitrogen flow. Upon finishing addition of ester, the reaction mixture was allowed to heat to reflux in an oil bath and stir overnight. After the reaction was cooled to room temperature, it was quenched with saturated NH_4Cl solution and extracted with DCM twice. Then the combined organic layer was dried with anhydrous Na_2SO_4, followed by evaporation under reduced pressure. The residue was purified by flash chromatography on silica gel (eluent = *n*-pentane:ethyl acetate = 5:1) to obtain the product **1j**.

Method J

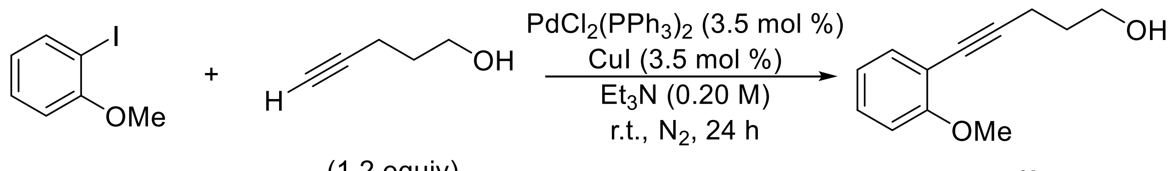

1k

According to the literature[S6], in a nitrogen-filled Schlenk flask with a rubber septum was charged with 1-iodo-2-methoxybenzene (2.3 g, 10 mmol), pent-4-yn-1-ol (1.0 g, 11 mmol), $Pd(PPh_3)_2Cl_2$ (0.25 g, 0.35 mmol), CuI (66 mg, 0.35 mmol). Then anhydrous Et_3N (50 mL) was added via syringe through a rubber septum, and the reaction mixture was stirred at room temperature for 24 hours. After the reaction mixture was cooled to room temperature, the reaction mixture was filtered through a pad of silica gel and washed with ethyl acetate. The filtrate was concentrated under reduced pressure and the residue was purified by flash chromatography on silica gel to obtain the Sonogashira coupling product **1k**.

Method K

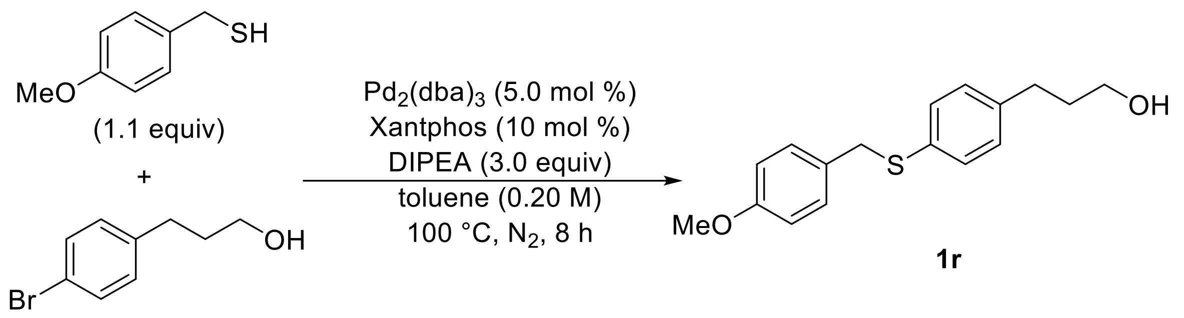

According to the literature[S7], the pressure tube was charged with 3-(4-bromophenyl)propan-1-ol (1.0 g, 4.6 mmol), (4-methoxyphenyl)methanethiol (0.77 g, 5.0 mmol), $Pd_2(dba)_3$ (0.21 g, 0.23 mmol), 4,5-bis(diphenylphosphino)-9,9-dimethylxanthene (Xantphos) (0.27 g, 0.46 mmol), DIPEA (1.8 g, 14 mmol), then anhydrous toluene (25 mL) was added via syringe through a rubber septum, and then the tube was sealed with a Teflon screwcap under nitrogen flow. The reaction mixture was stirred at 100 °C in an oil bath for 24 hours. After the reaction mixture was cooled to room temperature, the reaction mixture was filtered through a pad of silica gel and washed with ethyl acetate. The filtrate was concentrated under reduced pressure and the residue was purified by flash chromatography on silica gel (eluent = *n*-pentane:ethyl acetate = 5:1) to obtain the product **1r**.

Method L

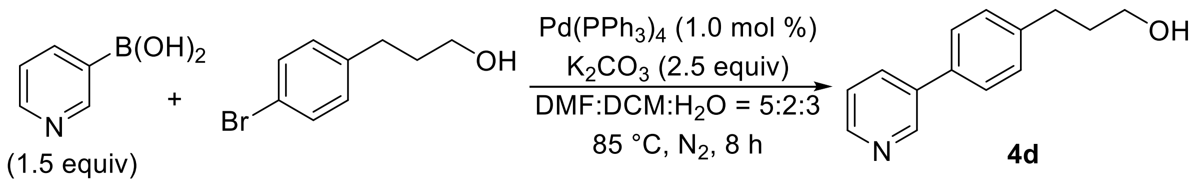

According to the literature[S8], the pressure tube was charged with 3-(4-bromophenyl)propan-1-ol (2.2 g, 10 mmol), pyridin-3-ylboronic acid (1.9 g, 15 mmol), $Pd(PPh_3)_4$ (0.12 g, 0.10 mmol), K_2CO_3 (3.5 g, 25 mmol), then DMF (25 mL), DCM (10 mL) and H_2O (15 mL) were added via syringe through a rubber septum, and then the tube was sealed with a Teflon screwcap under nitrogen flow. The reaction mixture was stirred at 85 °C in an oil bath for 8 hours. After the reaction mixture was cooled to room temperature, the reaction mixture was diluted with ethyl acetate (50 mL) and washed with water twice, brine. The organic phase was collected, dried over anhydrous Na_2SO_4 and concentrated under reduced pressure. The residue was purified by flash chromatography on silica gel to obtain the product **4d**.

3.6 Miscellaneous methods for the preparation alcohols

Method M

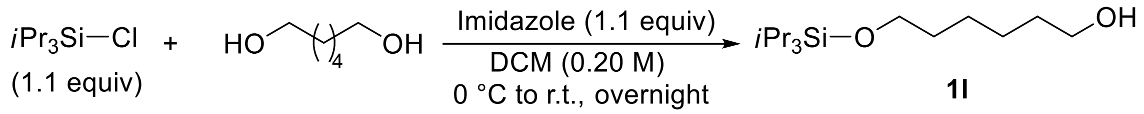

1l

To a 100 mL round-bottom flask equipped with a Teflon-coated magnetic stir bar was added hexane-1,6-diol (1.2 g, 10 mmol), and imidazole (0.75 g, 11 mmol). Then anhydrous dichloromethane 50 mL was added and the reaction mixture was cooled down to 0 °C with ice bath. To this mixture was dropwise added *i*Pr_3SiCl (2.1 g, 11 mmol), and the reaction mixture was stirred at room temperature overnight. After the reaction was judged complete by TLC, the reaction mixture was quenched with H_2O (30 mL), extract with DCM (2 × 30 mL). Then the combined organic layer was concentrated under reduced pressure and filtrated. The filtrate was dried with anhydrous Na_2SO_4, followed by evaporation under reduced pressure to remove the solvent. The residue was purified by flash chromatography on silica gel to obtain the TIPS mono-protected diol **1l**.

Method N

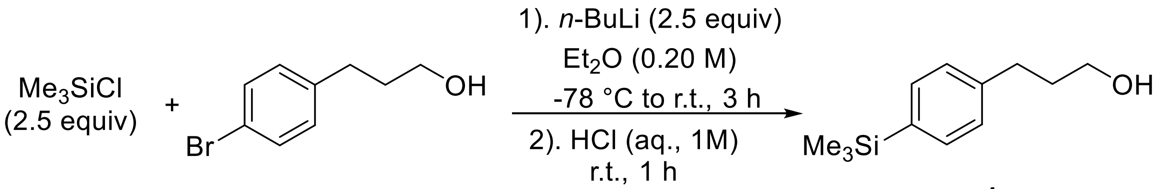

1z

Step 1: In a nitrogen-filled Schlenk flask with a rubber septum was charged with 3-(4-bromophenyl)propan-1-ol (2.2 g, 10 mmol) and diether 50 mL. When the solution was cooled to –78 °C with liquid nitrogen with acetone, 10 mL of *n*-butyllithium (25 mmol, 2.5 mol/L in *n*-hexane) was dropwise added to the solution via syringe through a rubber septum over a period of 30 minutes, and the reaction mixture was stirred at –78 °C for 1 hour. After the indicated reaction time, chlorotrimethylsilane (2.2 g, 25 mmol) was dropwise added to the solution via syringe through a rubber septum over a period of 30 minutes, and the reaction mixture was stirred at –78 °C for another 1 hour.

Step 2: After completion of the reaction as indicated by TLC, the reaction system was allowed to warm to room temperature and quenched with 20 mL HCl solution (aqueous, 1.0 mol/L). Then, the reaction mixture was stirred at room temperature for 1 hour. Upon finishing, the reaction mixture was extract with DCM 30 mL twice. Then the combined organic layer was

concentrated under reduced pressure and filtrated. The filtrate was dried with anhydrous Na_2SO_4, followed by evaporation under reduced pressure to remove the solvent. The residue was purified by flash chromatography on silica gel to obtain the silylated alcohol product **1z**.

Method O

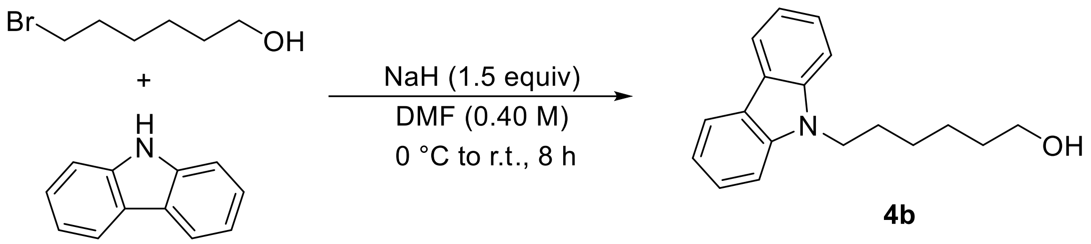

4b

According to the literature[S9], in a nitrogen-filled Schlenk flask with a rubber septum was charged with carbazole (1.7 g, 10 mmol) and DMF 25 mL. When the solution was cooled to 0 °C with ice bath, sodium hydride (60%, dispersion in Paraffin Liquid) (0.60 g, 15 mmol) was portionwise added to the solution over a period of 10 minutes, during which small bubbles were emitted from the reaction mixture. After the reaction mixture was stirred at this temperature for another 30 minutes, 6-bromohexan-1-ol (2.7 g, 15 mmol) was dropwise added to the solution via syringe through a rubber septum, and the reaction mixture was allowed to warm to room temperature and stirred for 8 hours. Upon finishing, the reaction mixture was diluted with ethyl acetate (50 mL) and washed with water twice, brine. The organic phase was collected, dried over anhydrous Na_2SO_4 and concentrated under reduced pressure. The residue was purified by flash chromatography on silica gel to obtain the product **4b**.

Method P

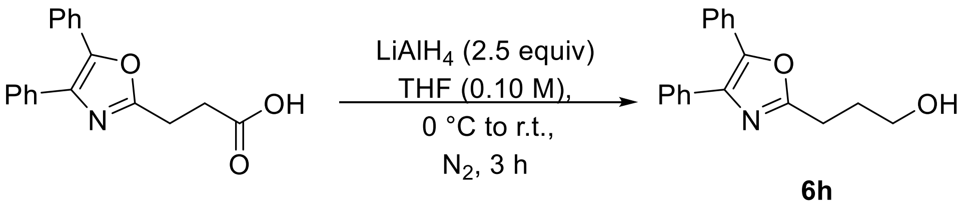

6h

According to the literature[S10], to a 250 mL flame-dried round-bottom Schlenk flask charged with a Teflon-coated magnetic stir bar and lithium aluminum hydride ($LiAlH_4$) (0.50 g, 13 mmol) was added dry THF 50 mL. When the solution was cooled to 0 °C with ice bath, 3-(4,5-diphenyloxazol-2-yl)propanoic acid (*Oxaprozin*) (1.5 g, 5.0 mmol) was potion wise added over 30 minutes under a nitrogen flow. Upon finishing addition of acid, the reaction mixture was allowed to warm up to room temperature and stirred for 3 hours. After the reaction was finished,

it was quenched with saturated NH_4Cl solution and extracted with DCM twice. Then the combined organic layer was dried with anhydrous Na_2SO_4, followed by evaporation under reduced pressure. The residue was purified by flash chromatography on silica gel (eluent = *n*-pentane:ethyl acetate = 5:1) to obtain the product **6h**.

3.7 Syntheses of vinyl chlorosilane

Method Q

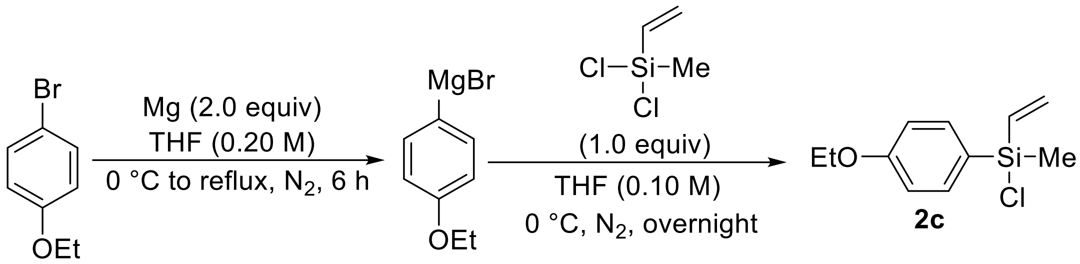

2c

Step 1: According to the literature[S11], to a 100 mL flame-dried two-neck round-bottom flask charged with a Teflon-coated magnetic stir bar and magnesium turnings (0.96 g, 40 mmol) was added dry THF 40 mL. Then two grain of iodine was added, and 1-bromo-4-ethoxybenzene (4.0 g, 20 mmol) was dropwise added over 15 minutes under vigorous stirring. After the addition of the aryl bromide, the reaction mixture was allowed to heated to the refluxed temperature in an oil bath and stirred for 6 hours.

Step 2: After the indicated reaction time, the reaction mixture was cooled down to 0 °C with ice bath. To this mixture was dropwise added dichloro(methyl)(vinyl)silane (2.6 mL, 2.8 g, 20 mmol) over 15 minutes. After addition, the solution was allowed to warm to room temperature and stirred overnight. Upon finishing, the reaction mixture was filtrated through Celite® and washed with *n*-hexane, and the filtrate was concentrated under reduced pressure. The residue was further purified by short-path distillation (160 °C, 3.0 × 10^{-1} mbar), yielding the vinyl chlorosilane **2c** as a colorless oil.

4 Mechanistic Control Experiments

4.1 Validate alky bromide as possible reaction intermediate

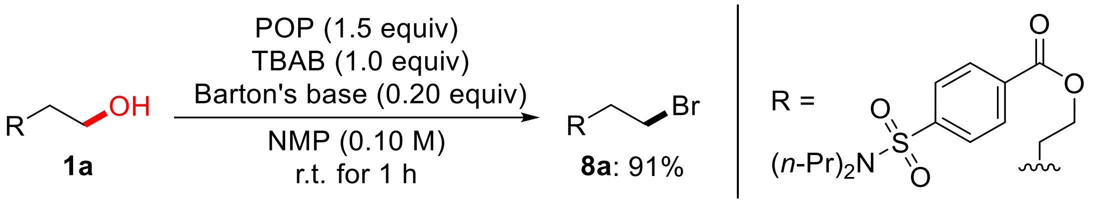

In a nitrogen-filled glovebox, a 35 mL pressure tube equipped with a stir bar was charged with 4-hydroxybutyl 4-(*N*,*N*-dipropylsulfamoyl)benzoate (**1a**, 84 mg, 0.20 mmol), POP (252 mg, 0.30 mmol), Barton's base (6.8 mg, 40 umol) and tetrabutylammonium bromide (64 mg, 0.20 mmol), then NMP (2.0 mL) was added to the pressure tube via syringe. After the reaction mixture was stirred at room temperature for 1 hour, reaction the mixture was diluted with ethyl acetate (10 mL) and washed with water twice. The organic phase was collected, dried over anhydrous Na_2SO_4, filtered and concentrated *in vacuo*. The residue was purified by flash chromatography on silica gel (eluent = *n*-pentane:ethyl acetate = 97:3) to obtain the deoxygenative brominated product **8a** in 91% yield (77 mg, yellow oil).

4.2 Using alky bromide as starting material

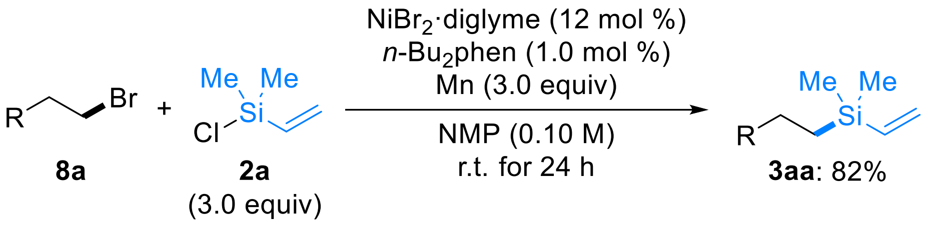

In a nitrogen-filled glovebox, a 35 mL pressure tube equipped with a stir bar was charged with 4-bromobutyl 4-(*N*,*N*-dipropylsulfamoyl)benzoate (**8a**, 84 mg, 0.20 mmol), $NiBr_2$·diglyme (8.4 mg, 24 umol), 2,9-dibutyl-1,10-phenanthroline (0.58 mg, 2.0 umol), chlorodimethyl(vinyl)silane (82 uL, 72 mg, 0.60 mmol) and manganese powder (33 mg, 0.60 mmol), then NMP (2.0 mL) was added to the pressure tube via syringe. After the reaction mixture was stirred at room temperature for 24 hours, the mixture was diluted with ethyl acetate (10 mL) and washed with water twice. The organic phase was collected, dried over anhydrous Na_2SO_4, filtered and concentrated *in vacuo*. The residue was purified by flash chromatography on silica gel (eluent = *n*-pentane:ethyl acetate = 97:3) to obtain the silylated product **3aa** in 82% yield (70 mg, colorless oil). Characterization data matched those obtained on 0.20 mmol scale (*vide infra*).

4.3 Influence of Ph_3PO on the reductive cross-coupling process

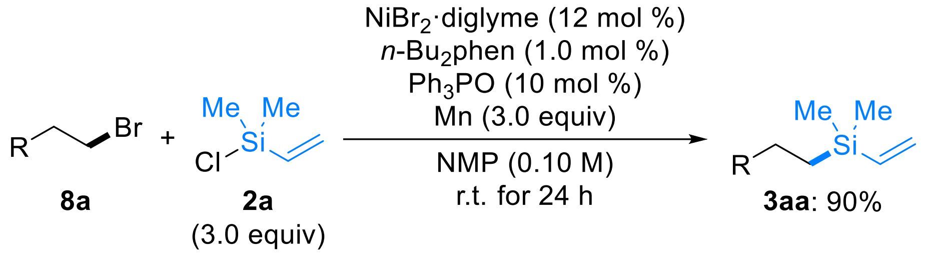

In a nitrogen-filled glovebox, a 35 mL pressure tube equipped with a stir bar was charged with 4-bromobutyl 4-(*N*,*N*-dipropylsulfamoyl)benzoate (**8a**, 84 mg, 0.20 mmol), $NiBr_2$·diglyme (8.4 mg, 24 umol), 2,9-dibutyl-1,10-phenanthroline (0.58 mg, 2.0 umol), triphenylphosphine oxide (5.6 mg, 20 umol), chlorodimethyl(vinyl)silane (82 uL, 72 mg, 0.60 mmol) and manganese powder (33 mg, 0.60 mmol), then NMP (2.0 mL) was added to the pressure tube via syringe. After the reaction mixture was stirred at room temperature for 24 hours, the mixture was diluted with ethyl acetate (10 mL) and washed with water twice. The organic phase was collected, dried over anhydrous Na_2SO_4, filtered and concentrated *in vacuo*. The residue was purified by flash chromatography on silica gel (eluent = *n*-pentane:ethyl acetate = 97:3) to obtain the silylated product **3aa** in 90% yield (77 mg, colorless oil). Characterization data matched those obtained on 0.20 mmol scale (*vide infra*).

5 Characterization Data of Products

5.1 Characterization data of starting materials

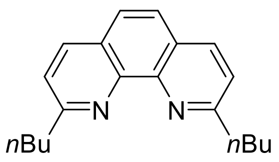

***n*-Bu₂phen**
$C_{20}H_{24}N_2$
M= 292.19 g/mol

2,9-Dibutyl-1,10-phenanthroline: Prepared from 1.10-phenanthroline according to ***Method A***. Purification by flash column chromatography on silica gel using *n*-pentane:ethyl acetate = 10:1 afforded *n*-Bu_2phen as a yellow solid (3.1 g, 86% yield). The NMR spectroscopic data are in accordance with those reported.[S2]

R_f = 0.80 (*n*-pentane:ethyl acetate = 4:1).

^{1}H NMR (500 MHz, $CDCl_3$) δ 8.14 (d, *J* = 8.4 Hz, 1H), 7.70 (s, 1H), 7.51 (d, *J* = 8.2 Hz, 1H), 3.24–3.20 (m, 2H), 1.94–1.88 (m, 2H), 1.56–1.48 (m, 2H), 1.01 (t, *J* = 7.4 Hz, 3H) ppm.

^{13}C NMR (126 MHz, $CDCl_3$) δ 163.3, 136.2, 127.1, 125.4, 122.4, 39.2, 31.9, 22.9, 14.0 ppm.

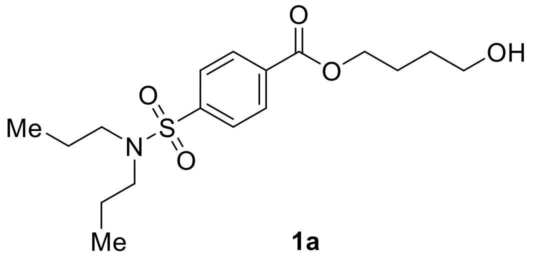

1a
$C_{17}H_{27}NO_5S$
M = 357.47 g/mol

4-Hydroxybutyl 4-(*N*,*N*-dipropylsulfamoyl)benzoate (1a): Prepared from probenecid (2.9 g, 10 mmol) and 1,4-diol (2.7 g, 30 mmol) according to ***Method C***. Purification by flash column chromatography on silica gel using *n*-pentane:ethyl acetate =3:1 afforded **1a** as a colorless oil (3.1 g, 86% yield).

R_f = 0.30 (*n*-pentane:ethyl acetate = 2:1).

^{1}H NMR (500 MHz, $CDCl_3$) δ 8.07 (d, *J* = 8.5 Hz, 2H), 7.79 (d, *J* = 8.5 Hz, 2H), 4.30 (t, *J* = 6.6 Hz, 2H), 3.62 (t, *J* = 6.5 Hz, 2H), 3.03–3.00 (m, 4H), 2.57 (s, 1H), 1.82–1.77 (m, 2H), 1.66–1.61 (m, 2H), 1.49–1.42 (m, 4H), 0.78 (t, *J* = 7.5 Hz, 6H) ppm.

^{13}C NMR (126 MHz, $CDCl_3$) δ 165.1, 143.8, 133.4, 130.0, 126.7, 65.3, 61.8, 49.7, 28.8, 24.9 21.7, 10.9 ppm.

IR (ATR): ṽ/cm^{-1} = 3543, 3444, 2934, 2874. 2292, 2107, 1718, 1599, 1464, 1398, 1338, 1270, 1156, 1085, 990, 863, 739, 693.

HRMS (APCI) for $C_{17}H_{28}NO_5S$ $[M+H]^+$: calculated 358.1683, found 358.1684.

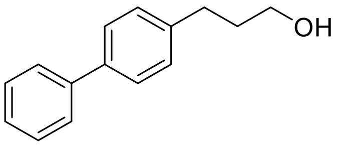

1c
$C_{15}H_{16}O$
M = 212.29 g/mol

3-([1,1'-Biphenyl]-4-yl)propan-1-ol (1c): Prepared from phenylboronic acid (1.3 g, 11 mmol) and 3-(4-bromophenyl)propan-1-ol (2.2 g, 10 mmol) according to ***Method H***. Purification by flash column chromatography on silica gel using *n*-pentane:ethyl acetate = 4:1 afforded **1c** as a white solid (1.5 g, 72% yield).

R_f = 0.35 (*n*-pentane:ethyl acetate = 2:1).

M.p.: 74–76 °C.

^{1}H NMR (500 MHz, $CDCl_3$) δ 7.63 (d, *J* = 7.7 Hz, 2H), 7.57 (d, *J* = 7.8 Hz, 2H), 7.47 (t, *J* = 7.6 Hz, 2H), 7.37 (t, *J* = 7.4 Hz, 1H), 7.31 (d, *J* = 7.8 Hz, 2H), 3.73 (t, *J* = 6.5 Hz, 2H), 2.79 (t, *J* = 7.8 Hz, 2H), 2.00–1.92 (m, 3H) ppm.

^{13}C NMR (126 MHz, $CDCl_3$) δ 140.9, 140.9, 138.7, 128.8, 128.7, 127.0, 127.0, 126.9, 62.1, 34.1, 31.6 ppm.

IR (ATR): ṽ/cm^{-1} = 3269, 3031, 2935, 2860, 2321, 2100, 204, 1890, 1796, 1598, 1562, 1522, 1483, 1406, 1338, 1155, 1037, 1005, 907, 814, 756, 685.

HRMS (APCI) for $C_{15}H_{16}O$ $[M]^+$: calculated 212.1196, found 212.1196.

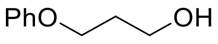

1h
$C_9H_{12}O_2$
M = 152.19 g/mol

3-Phenoxypropan-1-ol (1h): Prepared from phenol (0.94 g, 10 mmol) and 3-bromopropan-1-ol (2.8 g, 20 mmol) according to ***Method G***. Purification by flash column chromatography on silica gel using *n*-pentane:ethyl acetate = 4:1 afforded **1h** as a colorless oil (1.3 g, 87% yield).

R_f = 0.30 (*n*-pentane:ethyl acetate = 2:1).

^{1}H NMR (500 MHz, $CDCl_3$) δ 7.29 (t, *J* = 7.9 Hz, 2H), 6.96 (t, *J* = 7.4 Hz, 1H), 6.92 (d, *J* = 8.3 Hz, 2H), 4.10 (t, *J* = 6.1 Hz, 2H), 3.83 (t, *J* = 6.1 Hz, 2H), 2.63(s, 1H), 2.05–2.00 (m, 2H) ppm.

^{13}C NMR (126 MHz, $CDCl_3$) δ 158.7, 129.3, 120.7, 114.4, 65.2, 59.9, 31.9 ppm.

IR (ATR): ṽ/cm^{-1} = 3338, 2948, 2878, 2328, 2089, 1929, 1719, 1598, 1494, 1390, 1291, 1239, 1055, 999, 949, 881, 816, 749, 689.

HRMS (APCI) for $C_9H_{13}O_2$ $[M+H]^+$: calculated 153.0910, found 153.0911.

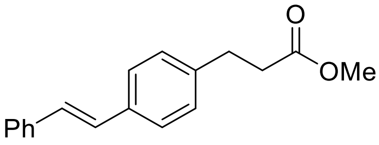

1j-s
$C_{18}H_{18}O_2$
M = 266.34 g/mol

Methyl (*E*)-3-(4-styrylphenyl)propanoate (1j-s): Prepared from 3-(4-bromophenyl)propanoate (2.4 g, 10 mmol) and styrene (1.1 g, 11 mmol) according to ***Method I***. Purification by flash column chromatography on silica gel using *n*-pentane:ethyl acetate = 95:5 afforded **1j-s** as a white solid (2.0 g, 76% yield).

R_f = 0.40 (*n*-pentane:ethyl acetate = 90:10).

^{1}H NMR (500 MHz, $CDCl_3$) δ 7.53 (d, *J* = 7.7 Hz, 2H), 7.47 (d, *J* = 7.8 Hz, 2H), 7.38 (t, *J* = 7.6 Hz, 2H), 7.28 (d, *J* = 2.5 Hz, 1H), 7.22 (d, *J* = 7.8 Hz, 2H), 7.10 (s, 2H), 3.70 (s, 3H), 2.98 (t, *J* = 7.8 Hz, 2H), 2.67 (t, *J* = 7.9 Hz, 2H) ppm.

^{13}C NMR (126 MHz, $CDCl_3$) δ 173.3, 140.0, 137.4, 135.5, 128.7, 128.6, 128.4, 128.2, 127.5, 126.6, 126.4, 51.6, 35.6, 30.7 ppm.

IR (ATR): ṽ/cm^{-1} = 3026, 2948, 2849, 2623, 2341, 2220, 2105, 1910, 1804, 1729, 1512, 1439, 1370, 1295, 1267, 1169, 1012, 964, 898, 864, 819, 751, 694.

HRMS (APCI) for $C_{18}H_{18}O_2$ [M]$^+$: calculated 266.1301, found 266.1302.

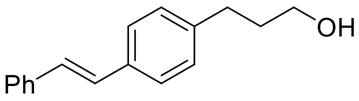

1j
$C_{17}H_{18}O$
M = 238.33 g/mol

(*E*)-3-(4-styrylphenyl)propan-1-ol (1j): Prepared from 1j-s (1.1 g, 4.0 mmol) and lithium aluminum hydride ($LiAlH_4$) (0.38 g, 10 mmol) ccording to ***Method I***. Purification by flash column chromatography on silica gel using *n*-pentane:ethyl acetate = 5:1 afforded **1j** as a white solid (0.72 g, 76% yield).

R_f = 0.30 (*n*-pentane:ethyl acetate = 2:1).

M.p.: 126–128 °C.

^{1}H NMR (500 MHz, $CDCl_3$) δ 7.53 (d, *J* = 7.1 Hz, 2H), 7.47 (d, *J* = 8.0 Hz, 2H), 7.38 (t, *J* = 7.7 Hz, 2H), 7.28 (t, *J* = 3.7 Hz, 1H), 7.22 (d, *J* = 8.1 Hz, 2H), 7.11 (d, *J* = 2.1 Hz, 2H), 3.72 (t, *J* = 6.4 Hz, 2H), 2.75 (t, *J* = 7.7 Hz, 2H), 1.96–1.90 (m, 2H), 1.47 (s, 1H) ppm.

^{13}C NMR (126 MHz, $CDCl_3$) δ 141.4, 137.4, 135.1, 128.8, 128.6, 128.5, 128.0, 127.4, 126.6, 126.4, 62.2, 34.1, 31.8 ppm.

IR (ATR): ṽ/cm^{-1} = 3231, 3022, 2936, 2861, 2343, 2115, 1995, 1878, 1802, 1655, 1593, 1490, 1446, 1374, 1221, 1180, 1117, 1041, 966, 910, 864, 803, 744, 689.

HRMS (APCI) for $C_{17}H_{18}O$ [M]$^+$: calculated 238.1352, found 238.1352.

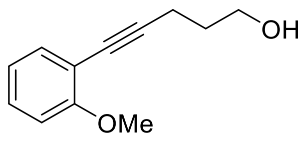

1k
$C_{12}H_{14}O_2$
M = 190.24 g/mol

5-(2-Methoxyphenyl)pent-4-yn-1-ol (1k): Prepared from 1-iodo-2-methoxybenzene (2.3 g, 10 mmol) and pent-4-yn-1-ol (1.0 g, 11 mmol) according to ***Method J***. Purification by flash column chromatography on silica gel using *n*-pentane:ethyl acetate = 4:1 afforded **1k** as a brown oil (1.6 g, 85% yield).

R_f = 0.30 (*n*-pentane:ethyl acetate = 2:1).

^{1}H NMR (500 MHz, $CDCl_3$) δ 7.29 (d, *J* = 7.8 Hz, 1H), 7.14 (t, *J* = 7.7 Hz, 1H), 6.79 (t, *J* = 7.6 Hz, 1H), 6.73 (d, *J* = 8.5 Hz, 1H), 3.74–3.70 (m, 5H), 3.44 (s, 1H), 2.48 (t, *J* = 7.0 Hz, 2H), 1.79–1.76 (m, 2H) ppm.

^{13}C NMR (126 MHz, $CDCl_3$) δ 159.4, 132.9, 128.6, 120.0, 112.4, 110.1, 93.4, 76.7, 61.0, 55.1, 30.9, 16.0 ppm.

IR (ATR): ṽ/cm^{-1} = 3519, 3337, 2934, 2328, 2122, 1997, 1783, 1733, 1594, 1490, 1460, 1432, 1258, 1179, 1115, 1021, 923, 848, 795, 748, 699.

HRMS (APCI) for $C_{12}H_{15}O_2$ $[M+H]^+$: calculated 191.1067, found 191.1064.

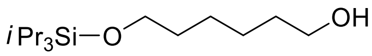

1l
$C_{15}H_{34}O_2Si$
M = 274.52 g/mol

6-((Triisopropylsilyl)oxy)hexan-1-ol (1l): Prepared from hexane-1,6-diol (1.2 g, 10 mmol) and chlorotriisopropylsilane (*i*Pr_3Si–Cl) (2.1 g, 11 mmol) according to ***Method M***. Purification by flash column chromatography on silica gel using *n*-pentane:ethyl acetate = 4:1 afforded **1l** as a colorless oil (2.2 g, 81% yield).

R_f = 0.30 (*n*-pentane:ethyl acetate = 2:1).

^{1}H NMR (500 MHz, $CDCl_3$) δ 3.66 (t, *J* = 6.6 Hz, 2H), 3.62 (t, *J* = 6.7 Hz, 2H), 1.82 (s, 1H), 1.57–1.52 (m, 4H), 1.38–1.35 (m, 4H), 1.09–1.02 (m, 21H) ppm.

^{13}C NMR (126 MHz, $CDCl_3$) δ 63.3, 62.9, 32.9, 32.7, 25.6, 25.5, 18.0, 12.0 ppm.

IR (ATR): ṽ/cm^{-1} = 3340, 2934, 2863, 2726, 2353, 2125, 2089, 1887, 1744, 1461, 1384, 1245, 1100, 1068, 1011, 918, 881, 788, 716, 678.

HRMS (APCI) for $C_{15}H_{35}O_2Si$ [M+H]$^+$: calculated 275.2401, found 275.2397.

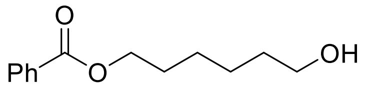

1m
$C_{13}H_{18}O_3$
M = 222.28 g/mol

6-Hydroxyhexyl benzoate (1m): Prepared from benzoyl chloride (1.4 g, 10 mmol) and 1,6-diol (2.4 g, 20 mmol) according to ***Method E***. Purification by flash column chromatography on silica gel using *n*-pentane:ethyl acetate = 3:1 afforded **1m** as a colorless oil (2.2 g, 99% yield).

R_f = 0.25 (*n*-pentane:ethyl acetate = 2:1).

^{1}H NMR (500 MHz, $CDCl_3$) δ 8.03 (d, *J* = 6.9 Hz, 2H), 7.54 (t, *J* = 7.5 Hz, 1H), 7.43 (t, *J* = 7.8 Hz, 2H), 4.32 (t, *J* = 6.6 Hz, 2H), 3.65 (t, *J* = 6.5 Hz, 2H), 1.80–1.75 (m, 2H), 1.62–1.57 (m, 3H), 1.49–1.42 (m, 4H) ppm.

^{13}C NMR (126 MHz, $CDCl_3$) δ 166.7, 132.8, 130.4, 129.5, 128.3, 64.9, 62.7, 32.6, 28.7, 25.8, 25.4 ppm.

IR (ATR): ṽ/cm^{-1} = 3419, 2933, 2859, 2339, 2096, 1714, 1601, 1451, 1387, 1270, 1175, 1111, 1069, 1025, 847, 752, 708.

HRMS (APCI) for $C_{13}H_{19}O_3$ [M+H]$^+$: calculated 223.1329, found 223.1328.

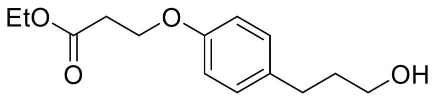

1n
$C_{14}H_{20}O_4$
M = 252.31 g/mol

Ethyl 3-(4-(3-hydroxypropyl)phenoxy)propanoate (1n): Prepared from 4-(3-hydroxypropyl)phenol (1.5 g, 10 mmol) and ethyl 3-bromopropanoate (3.6 g, 20 mmol)

according to ***Method G***. Purification by flash column chromatography on silica gel using *n*-pentane:ethyl acetate = 3:1 afforded **1n** as a colorless oil (1.0 g, 40% yield).

R$_f$ = 0.15 (*n*-pentane:ethyl acetate = 2:1).

^{1}H NMR (500 MHz, $CDCl_3$) δ 7.10 (d, *J* = 8.1 Hz, 2H), 6.83 (d, *J* = 8.1 Hz, 2H), 4.22 (t, *J* = 6.6 Hz, 2H), 4.18 (q, *J* = 7.0 Hz, 2H), 3.65 (t, *J* = 6.5 Hz, 2H), 2.77 (t, *J* = 6.6 Hz, 2H), 2.64 (t, *J* = 7.5 Hz, 2H), 1.88–1.84 (m, 2H), 1.70 (s, 1H), 1.27 (t, *J* = 7.8 Hz, 3H) ppm.

^{13}C NMR (126 MHz, $CDCl_3$) δ 171.1, 156.7, 134.2, 129.3, 114.6, 63.6, 62.2, 60.7, 34.7, 34.4, 31.1, 14.2 ppm.

IR (ATR): ṽ/cm^{-1} = 3426, 2934, 2299, 2112, 2084, 1885, 1731, 1610, 1510, 1465, 1372, 1235, 1176, 1030, 914, 831, 751, 666.

HRMS (APCI) for $C_{14}H_{21}O_4$[M+H]$^+$: calculated 253.1434, found 253.1433.

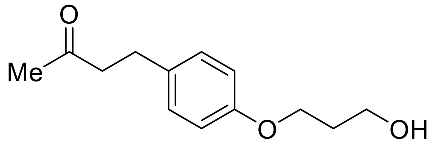

1o
$C_{13}H_{18}O_3$
M = 222.28 g/mol

4-(4-(3-Hydroxypropoxy)phenyl)butan-2-one (1o): Prepared from 4-(4-hydroxyphenyl)butan-2-one (1.6 g, 10 mmol) and 3-bromopropan-1-ol (2.8 g, 20 mmol) according to ***Method G***. Purification by flash column chromatography on silica gel using *n*-pentane:ethyl acetate = 3:1 afforded **1o** as a white solid (1.6 g, 74% yield).

R$_f$ = 0.40 (*n*-pentane:ethyl acetate = 2:1).

M.p.: 44–46 °C.

^{1}H NMR (500 MHz, $CDCl_3$) δ 7.01 (d, *J* = 8.3 Hz, 2H), 6.76 (d, *J* = 8.4 Hz, 2H), 3.99 (t, *J* = 6.2 Hz, 2H), 3.74 (t, *J* = 6.2 Hz, 2H), 3.09 (s, 1H), 2.75 (t, *J* = 7.6 Hz, 2H), 2.64 (t, *J* = 7.7 Hz, 2H), 2.05 (s, 3H), 1.96–1.92 (m, 2H) ppm.

^{13}C NMR (126 MHz, $CDCl_3$) δ 208.2, 156.9, 132.7, 128.8, 114.2, 114.2, 65.0, 59.3, 44.9, 31.8, 29.7, 28.5 ppm.

IR (ATR): ṽ/cm^{-1} = 3277, 3075, 3030, 2939, 2877, 2651, 2288, 2086, 1930, 1889, 1702, 1652, 1609, 1558, 1511, 1472, 1408, 1367, 1284, 1241, 1159, 1104, 1052, 988, 953, 885, 808, 748.

HRMS (APCI) for $C_{13}H_{19}O_3$ $[M+H]^+$: calculated 223.1329, found 223.1329.

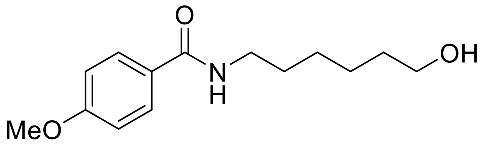

1p
$C_{14}H_{21}NO_3$
M = 251.33 g/mol

N*-(6-hydroxyhexyl)-4-methoxybenzamide (1p):** Prepared from 4-methoxybenzoyl chloride (1.7 g, 10 mmol) and 6-aminohexan-1-ol (1.8 g, 15 mmol) according to ***Method F. Purification by flash column chromatography on silica gel using *n*-pentane:ethyl acetate = 1:1 afforded **1p** as a white solid (2.2 g, 86% yield).

R_f = 0.15 (*n*-pentane:ethyl acetate = 1:1).

M.p.: 94–96 °C.

^{1}H NMR (500 MHz, $CDCl_3$) δ 7.72 (d, *J* = 8.8 Hz, 2H), 6.91 (d, *J* = 8.7 Hz, 2H), 6.20 (s, 1H), 3.84 (s, 3H), 3.63 (t, *J* = 6.5 Hz, 2H), 3.43 (t, *J* = 7.1 Hz, 2H), 1.88 (s, 1H), 1.63–1.56 (m, 4H), 1.41 (d, *J* = 3.7 Hz, 4H) ppm.

^{13}C NMR (126 MHz, $CDCl_3$) δ 167.1, 162.1, 128.6, 127.0, 113.7, 62.7, 55.4, 39.8, 32.6, 29.7, 26.6, 25.3 ppm.

IR (ATR): ṽ/cm^{-1} = 3397, 3315, 3060, 2932, 2856, 2341, 2209, 2101, 1995, 1905, 1730, 1627, 1533, 1500, 1358, 1297, 1247, 1179, 1109, 1031, 982, 845, 765.

HRMS (APCI) for $C_{14}H_{22}NO_3$ $[M+H]^+$: calculated 252.1594, found 252.1592.

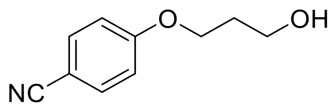

1q
$C_{10}H_{11}NO_2$
M = 177.20 g/mol

4-(3-Hydroxypropoxy)benzonitrile (1q): Prepared from 4-hydroxybenzonitrile (1.2 g, 10 mmol) and 3-bromopropan-1-ol (2.8 g, 20 mmol) according to ***Method G***. Purification by flash

column chromatography on silica gel using *n*-pentane:ethyl acetate = 2:1 afforded **1q** as a white solid (1.1 g, 64% yield).

R_f = 0.10 (*n*-pentane:ethyl acetate = 2:1).

M.p.: 41–43 °C.

^{1}H NMR (500 MHz, $CDCl_3$) δ 7.52 (d, *J* = 8.6 Hz, 2H), 6.92 (d, *J* = 8.8 Hz, 2H), 4.13 (t, *J* = 5.5 Hz, 2H), 3.82–3.81 (m, 2H), 2.20 (s, 1H), 2.03 (t, *J* = 5.1 Hz, 2H) ppm.

^{13}C NMR (126 MHz, $CDCl_3$) δ 162.1, 133.9, 119.1, 115.1, 103.6, 65.4, 59.2, 31.7 ppm.

IR (ATR): ṽ/cm^{-1} = 3515, 3205, 2947, 2880, 2564, 2342, 2221, 2105, 2012, 1909, 1739, 1651, 1602, 1505, 1465, 1421, 1389, 1304, 1256, 1227, 1167, 1062, 987, 951, 831, 707.

HRMS (APCI) for $C_{10}H_{12}NO_2$ $[M+H]^+$: calculated 178.0863, found 178.0861.

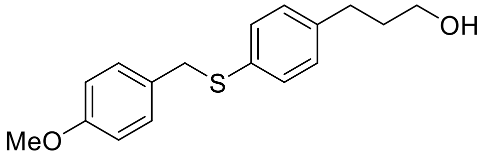

1r
$C_{17}H_{20}O_2S$
M = 288.40 g/mol

3-(4-((4-Methoxybenzyl)thio)phenyl)propan-1-ol (1r): Prepared from 3-(4-bromophenyl)propan-1-ol (1.0 g, 4.6 mmol) and (4-methoxyphenyl)methanethiol (0.77 g, 5.0 mmol) according to ***Method K***. Purification by flash column chromatography on silica gel using *n*-pentane:ethyl acetate = 3:1 afforded **1r** as an orange solid (0.92 g, 69% yield).

R_f = 0.40 (*n*-pentane:ethyl acetate = 2:1).

M.p.: 91–93 °C.

^{1}H NMR (500 MHz, $CDCl_3$) δ 7.23 (d, *J* = 8.1 Hz, 2H), 7.19 (d, *J* = 8.6 Hz, 2H), 7.09 (d, *J* = 7.8 Hz, 2H), 6.81 (d, *J* = 8.6 Hz, 2H), 4.04 (s, 2H), 3.78 (s, 3H), 3.66 (t, *J* = 6.0 Hz, 2H), 2.67 (t, *J* = 7.7 Hz, 2H), 1.88–1.85 (m, 2H), 1.45 (s, 1H) ppm.

^{13}C NMR (126 MHz, $CDCl_3$) δ 158.7, 140.2, 133.4, 130.4, 129.9, 129.6, 128.9, 113.8, 62.1, 55.2, 38.9, 34.0, 31.5 ppm.

IR (ATR): ṽ/cm^{-1} = 3306, 3015, 2932, 2840, 2321, 2110, 1987, 1892, 1773, 1654, 1609, 1510, 1491, 1448, 1378, 1299, 1238, 1173, 1065, 1028, 907, 828, 794, 753, 699.

HRMS (APCI) for $C_{17}H_{19}O_2S$ $[M-H]^+$: calculated 287.1106, found 287.1100.

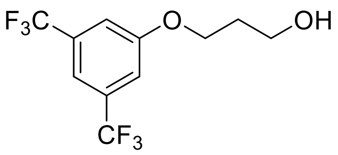

1s
$C_{11}H_{10}F_6O_2$
M = 288.19 g/mol

3-(3,5-Bis(trifluoromethyl)phenoxy)propan-1-ol (1s): Prepared from 2,5-bis(trifluoromethyl)phenol (2.3 g, 10 mmol) and 3-bromopropan-1-ol (2.8 g, 20 mmol) according to ***Method G***. Purification by flash column chromatography on silica gel using *n*-pentane:ethyl acetate = 3:1 afforded **1s** as a yellow oil (2.6 g, 91% yield).

R_f = 0.40 (*n*-pentane:ethyl acetate = 2:1).

^{1}H NMR (500 MHz, $CDCl_3$) δ 7.43 (s, 1H), 7.30 (s, 2H), 4.17 (t, *J* = 6.0 Hz, 2H), 3.84 (t, *J* = 6.0 Hz, 2H), 2.68 (s, 1H), 2.07–2.04 (m, 2H) ppm.

^{13}C NMR (126 MHz, $CDCl_3$) δ 159.5, 132.8 (q, *J* = 33.3 Hz), 123.2 (q, *J* = 272.9 Hz), 114.8 (q, *J* = 3.5 Hz), 114.3 (p, *J* = 3.9 Hz), 65.9, 59.4, 31.7 ppm.

^{19}F NMR(471 MHz, $CDCl_3$) δ –63.3 ppm.

IR (ATR): ṽ/cm^{-1} = 3348, 2955, 2888, 2643, 2462, 2326, 1907, 1726, 1612, 1463, 1372, 1273, 1169, 1122, 1055, 1005, 868, 759, 700, 680.

HRMS (APCI) for $C_{11}H_{11}F_6O_2$ $[M+H]^+$: calculated 289.0658, found 289.0656.

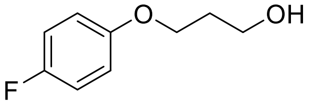

1t
$C_9H_{11}FO_2$
M = 170.18 g/mol

3-(4-Fluorophenoxy)propan-1-ol (1t): Prepared from 4-fluorophenol (1.1 g, 10 mmol) and 3-bromopropan-1-ol (2.8 g, 20 mmol) according to ***Method G***. Purification by flash column

chromatography on silica gel using *n*-pentane:ethyl acetate = 3:1 afforded **1t** as a colorless oil (1.5 g, 90% yield).

R$_f$ = 0.40 (*n*-pentane:ethyl acetate = 2:1).

^{1}H NMR (500 MHz, $CDCl_3$) δ 6.95 (t, *J* = 8.5 Hz, 2H), 6.82 (dd, *J* = 9.1, 4.4 Hz, 2H), 4.05 (t, *J* = 6.2 Hz, 2H), 3.82 (t, *J* = 6.1 Hz, 2H), 2.29 (s, 1H), 2.02–1.98 (m, 2H) ppm.

^{13}C NMR (126 MHz, $CDCl_3$) δ 157.2 (d, *J* = 238.2 Hz), 154.8 (d, *J* = 2.2 Hz), 115.7 (d, *J* = 22.9 Hz), 115.4 (d, *J* = 8.1 Hz), 66.1, 60.0, 31.9 ppm.

^{19}F NMR(471 MHz, $CDCl_3$) δ –123.9 ppm.

IR (ATR): ṽ/cm^{-1} = 3348, 2950, 2879, 2106, 1860, 1732, 1600, 1503, 1392, 1292, 1200, 1096, 1056, 989, 950, 825, 754.

HRMS (APCI) for $C_9H_{12}FO_2$ [M+H]$^+$: calculated 171.0816, found 171.0814.

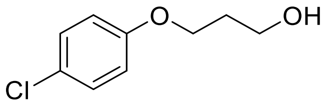

1u
$C_9H_{11}ClO_2$
M = 186.63 g/mol

3-(4-Chlorophenoxy)propan-1-ol (1u): Prepared from 4-chlorophenol (1.3 g, 10 mmol) and 3-bromopropan-1-ol (2.8 g, 20 mmol) according to ***Method G***. Purification by flash column chromatography on silica gel using *n*-pentane:ethyl acetate = 3:1 afforded **1u** as a colorless oil (1.7 g, 91% yield).

R$_f$ = 0.40 (*n*-pentane:ethyl acetate = 2:1).

^{1}H NMR (500 MHz, $CDCl_3$) δ 7.21 (d, *J* = 9.1 Hz, 2H), 6.81 (d, *J* = 9.0 Hz, 2H), 4.05 (t, *J* = 6.1 Hz, 2H), 3.81 (t, *J* = 6.0 Hz, 2H), 2.22 (s, 1H), 2.01–1.99 (m, 2H) ppm.

^{13}C NMR (126 MHz, $CDCl_3$) δ 157.3, 129.2, 125.6, 115.7, 65.7, 59.9, 31.9 ppm.

IR (ATR): ṽ/cm^{-1} = 3345, 2948, 2878, 2536, 2277, 2087, 1940, 1870, 1733, 1595, 1489, 1391, 1282, 1239, 1169, 1092, 1055, 988, 948, 820, 757, 664.

HRMS (APCI) for $C_9H_{12}ClO_2$ [M+H]$^+$: calculated 187.0520, found 187.0519.

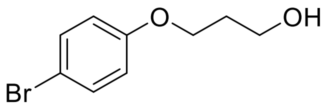

1v
$C_9H_{11}BrO_2$
M = 231.09 g/mol

3-(4-Bromophenoxy)propan-1-ol (1v): Prepared from 4-bromophenol (1.7 g, 10 mmol) and 3-bromopropan-1-ol (2.8 g, 20 mmol) according to ***Method G***. Purification by flash column chromatography on silica gel using *n*-pentane:ethyl acetate = 3:1 afforded **1v** as a colorless oil (2.1 g, 89% yield).

R_f = 0.40 (*n*-pentane:ethyl acetate = 2:1).

^{1}H NMR (500 MHz, $CDCl_3$) δ 7.35 (d, *J* = 8.9 Hz, 2H), 6.76 (d, *J* = 8.9 Hz, 2H), 4.05 (t, *J* = 6.1 Hz, 2H), 3.81 (t, *J* = 6.0 Hz, 2H), 2.12 (s, 1H), 2.03–1.98 (m, 2H) ppm.

^{13}C NMR (126 MHz, $CDCl_3$) δ 157.8, 132.2, 116.2, 112.9, 65.6, 59.9, 31.8 ppm.

IR (ATR): $\tilde{v}$/cm^{-1} = 3343, 3070, 2948, 2879, 2283, 2088, 1941, 1871, 1720, 1589, 1487, 1390, 1283, 1237, 1170, 1054, 988, 948, 818, 754.

HRMS (APCI) for $C_9H_{12}BrO_2$ $[M+H]^+$: calculated 231.0015, found 231.0014.

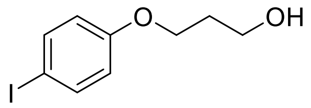

1w
$C_9H_{11}IO_2$
M = 278.09 g/mol

4-Hydroxybutyl 4-(*N*,*N*-dipropylsulfamoyl)benzoate (1w): Prepared from 4-iodophenol (2.2 g, 10 mmol) and 3-bromopropan-1-ol (2.8g, 20 mmol) according to ***Method G***. Purification by flash column chromatography on silica gel using *n*-pentane:ethyl acetate = 3:1 afforded **1w** as a white solid (2.6 g, 92% yield).

R_f = 0.40 (*n*-pentane:ethyl acetate = 2:1).

M.p.: 62–64 °C.

^{1}H NMR (500 MHz, $CDCl_3$) δ 7.54 (d, J = 8.4 Hz, 2H), 6.68 (d, J = 8.4 Hz, 2H), 4.07 (t, J = 6.0 Hz, 2H), 3.84 (t, J = 5.9 Hz, 2H), 2.05–2.00 (m, 2H), 1.80 (s, 1H) ppm.

^{13}C NMR (126 MHz, $CDCl_3$) δ 158.6, 138.2, 116.9, 82.8, 65.6, 60.1, 31.9 ppm.

IR (ATR): ṽ/cm^{-1} = 3247, 3167, 2908, 2872, 2526, 2289, 2112, 1874, 1736, 1583, 1483, 1358, 1283, 1245, 1171, 1113, 1058, 995, 935, 819, 691.

HRMS (APCI) for $C_9H_{11}IO_2$ $[M]^+$: calculated 277.9798, found 277.9798.

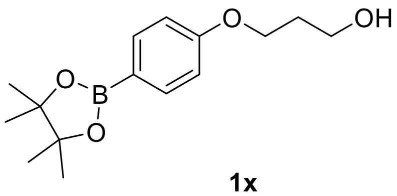

1x
$C_{15}H_{23}BO_4$
M = 278.15 g/mol

3-(4-(4,4,5,5-Tetramethyl-1,3,2-dioxaborolan-2-yl)phenoxy)propan-1-ol (1x): Prepared from 4-(4,4,5,5-tetramethyl-1,3,2-dioxaborolan-2-yl)phenol (2.2 g, 10 mmol) and 3-bromopropan-1-ol (2.8 g, 20 mmol) according to ***Method G***. Purification by flash column chromatography on silica gel using *n*-pentane:ethyl acetate = 3:1 afforded **1x** as a colorless oil (2.3 g, 83% yield).

R_f = 0.20 (*n*-pentane:ethyl acetate = 2:1).

^{1}H NMR (500 MHz, $CDCl_3$) δ 7.73 (d, *J* = 8.3 Hz, 2H), 6.86 (d, *J* = 8.3 Hz, 2H), 4.05 (t, *J* = 6.2 Hz, 2H), 3.76 (t, *J* = 6.2 Hz, 2H), 2.89 (s, 1H), 1.99–1.96 (m, 2H), 1.30 (s, 12H) ppm.

^{13}C NMR (126 MHz, $CDCl_3$) δ 161.3, 136.3, 113.7, 83.4, 64.8, 59.3, 31.8, 24.6 ppm.

IR (ATR): ṽ/cm^{-1} = 3422, 2976, 2932, 2250, 2125, 2091, 1947, 1731, 1603, 1515, 1469, 1356, 1315, 1243, 1140, 1088, 1056, 960, 831, 753.

HRMS (APCI) for $C_{15}H_{24}BO_4$ $[M+H]^+$: calculated 279.1762, found 279.1762.

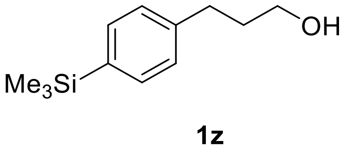

1z
$C_{12}H_{20}OSi$
M = 208.38 g/mol

3-(4-(Trimethylsilyl)phenyl)propan-1-ol (1z): Prepared from3-(4-bromophenyl)propan-1-ol (2.2 g, 10 mmol) and chlorotrimethylsilane (2.2 g, 25 mmol) according to ***Method N***.

Purification by flash column chromatography on silica gel using *n*-pentane:ethyl acetate = 4:1 afforded **1z** as a colorless oil (1.5 g, 73% yield).

R_f = 0.40 (*n*-pentane:ethyl acetate = 2:1).

^{1}H NMR (500 MHz, $CDCl_3$) δ 7.48 (d, *J* = 7.9 Hz, 2H), 7.23 (d, *J* = 7.5 Hz, 2H), 3.70 (t, *J* = 6.5 Hz, 2H), 2.73 (t, *J* = 7.8 Hz, 2H), 1.94–1.89 (m, 3H), 0.29 (s, 9H) ppm.

^{13}C NMR (126 MHz, $CDCl_3$) δ 142.4, 137.4, 133.4, 127.9, 62.2, 34.0, 32.0, –1.1 ppm.

IR (ATR): ṽ/cm^{-1} = 3324, 3064, 3011, 2950, 2305, 2197, 2122, 1909, 1738, 1600, 1448, 1396, 1246, 1107, 1041, 912, 829, 752, 690.

HRMS (APCI) for $C_{12}H_{21}OSi$ [M+H]$^+$: calculated 209.1356, found 209.1357.

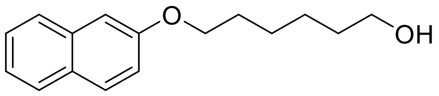

4a
$C_{16}H_{20}O_2$
M = 244.33g/mol

6-(Naphthalen-2-yloxy)hexan-1-ol (4a): Prepared from naphthalen-2-ol (1.4 g, 10 mmol) and 6-bromohexan-1-ol (3.6 g, 20 mmol) according to ***Method G***. Purification by flash column chromatography on silica gel using *n*-pentane:ethyl acetate = 4:1 afforded **4a** as an off-white solid (2.0 g, 83% yield).

R_f = 0.45 (*n*-pentane:ethyl acetate = 2:1).

M.p.: 59–61 °C.

^{1}H NMR (500 MHz, $CDCl_3$) δ 7.77–7.71 (m, 3H), 7.43 (t, *J* = 7.5 Hz, 1H), 7.32 (t, *J* = 6.9 Hz, 1H), 7.14 (dd, *J* = 11.2, 2.5 Hz, 2H), 4.09 (t, *J* = 6.5 Hz, 2H), 3.68 (t, *J* = 6.6 Hz, 2H), 1.90–1.84 (m, 2H), 1.66–1.60 (m, 2H), 1.58–1.53 (m, 2H), 1.50–1.45 (m, 2H), 1.27 (s, 1H) ppm.

^{13}C NMR (126 MHz, $CDCl_3$) δ 157.0, 134.6, 129.3, 128.9, 127.6, 126.7, 126.3, 123.5, 119.0, 106.5, 67.8, 62.9, 32.7, 29.2, 26.0, 25.6 ppm.

IR (ATR): ṽ/cm^{-1} = 3337, 3059, 2920, 2854, 2340, 2116, 1998, 1903, 1828, 1718, 1626, 1598, 1508, 1463, 1387, 1256, 1215, 1183, 1116, 1072, 1037, 989, 961, 840, 811, 741.

HRMS (APCI) for $C_{16}H_{21}O_2$ [M+H]$^+$: calculated 245.1536, found 245.1536.

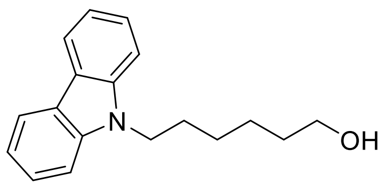

4b
$C_{18}H_{21}NO$
M = 267.37 g/mol

6-(9*H*-carbazol-9-yl)hexan-1-ol (4b): Prepared from carbazole (1.7 g, 10 mmol) and 6-bromohexan-1-ol (2.7 g, 15 mmol) according to ***Method O***. Purification by flash column chromatography on silica gel using *n*-pentane:ethyl acetate = 4:1 afforded **4b** as a white solid (2.5 g, 95% yield).

R_f = 0.45 (*n*-pentane:ethyl acetate = 2:1).

M.p.: 122–124 °C.

^{1}H NMR (500 MHz, $CDCl_3$) δ 8.11 (d, *J* = 7.7 Hz, 2H), 7.47 (t, *J* = 7.6 Hz, 2H), 7.41 (d, *J* = 8.1 Hz, 2H), 7.24 (t, *J* = 7.4 Hz, 2H), 4.31 (t, *J* = 7.2 Hz, 2H), 3.60 (t, *J* = 6.5 Hz, 2H), 1.91–1.89 (m, 2H), 1.53 (t, *J* = 6.7 Hz, 2H), 1.42–1.39 (m, 4H) ppm.

^{13}C NMR (126 MHz, $CDCl_3$) δ 140.4, 125.6, 122.8, 120.3, 118.7, 108.6, 62.7, 42.9, 32.5, 28.9, 27.0, 25.5 ppm.

IR (ATR): ṽ/cm^{-1} = 3293, 3048, 2923, 2854, 2342, 2093, 1895, 1775, 1592, 1449, 1374, 1322, 1229, 1150, 1119, 1056, 1018, 926, 845, 747, 722.

HRMS (APCI) for $C_{18}H_{22}NO$ $[M+H]^+$: calculated 268.1696, found 268.1693.

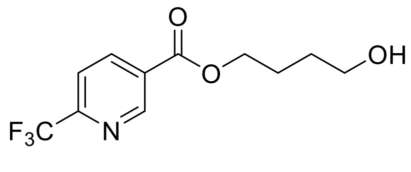

4c
$C_{11}H_{12}F_3NO_3$
M = 263.22 g/mol

4-Hydroxybutyl 6-(trifluoromethyl)nicotinate (4c): Prepared from 6-(trifluoromethyl)nicotinic acid (1.9 g, 10 mmol) and 1,4-diol (2.7 g, 30 mmol) according to ***Method C***. Purification by flash column chromatography on silica gel using *n*-pentane:ethyl acetate = 2:1 afforded **4c** as a yellow oil (2.4 g, 92% yield).

R_f = 0.30 (*n*-pentane:ethyl acetate = 1:1).

^{1}H NMR (500 MHz, $CDCl_3$) δ 9.27 (s, 1H), 8.46 (d, *J* = 8.1 Hz, 1H), 7.76 (d, *J* = 8.1 Hz, 1H), 4.42 (t, *J* = 6.6 Hz, 2H), 3.71 (t, *J* = 6.4 Hz, 2H), 2.29 (s, 1H), 1.91–1.86 (m, 2H), 1.73–1.67 (m, 2H) ppm.

^{13}C NMR (126 MHz, $CDCl_3$) δ 164.0, 151.3 (q, *J* = 35.0 Hz), 150.9, 138.7, 128.7, 121.0 (q, *J* = 274.7 Hz), 120.2 (q, *J* = 2.6 Hz), 65.8, 62.0, 28.9, 25.1 ppm.

^{19}F NMR (471 MHz, $CDCl_3$) δ –68.3 ppm.

IR (ATR): ṽ/cm^{-1} = 3443, 3381, 2944, 2419, 2352, 2203, 2116, 1722, 1601, 1467, 1389, 1332, 1279, 1178, 1118, 1083, 1023, 943, 862, 790, 735.

HRMS (APCI) for $C_{11}H_{13}F_3NO_3$ $[M+H]^+$: calculated 264.0842, found 264.0840.

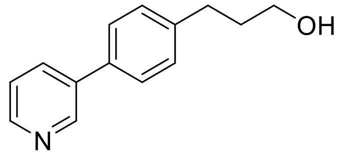

4d
$C_{14}H_{15}NO$
M = 213.28 g/mol

3-(4-(Pyridin-3-yl)phenyl)propan-1-ol (4d): Prepared from 3-(4-bromophenyl)propan-1-ol (2.2 g, 10 mmol) and pyridin-3-ylboronic acid (1.9 g, 15 mmol) according to ***Method L***. Purification by flash column chromatography on silica gel using *n*-pentane:ethyl acetate = 2:1 afforded **4d** as a colorless oil (1.7 g, 80% yield).

R_f = 0.30 (*n*-pentane:ethyl acetate = 1:1).

^{1}H NMR (500 MHz, $CDCl_3$) δ 8.74 (s, 1H), 8.47 (d, *J* = 5.2 Hz, 1H), 7.78 (d, *J* = 8.0 Hz, 1H), 7.40(d, *J* = 7.9 Hz, 2H), 7.28–7.23 (m, 3H), 4.12 (s, 1H), 3.66 (t, *J* = 6.5 Hz, 2H), 2.72 (t, *J* = 7.9 Hz, 2H), 1.91–1.86 (m, 2H) ppm.

^{13}C NMR (126 MHz, $CDCl_3$) δ 147.6, 147.6, 142.2, 136.4, 134.8, 134.2, 129.0, 126.8, 123.5, 61.3, 34.0, 31.6 ppm.

IR (ATR): ṽ/cm^{-1} = 3748, 3306, 3027, 2930, 2859, 2481, 2157, 2025, 1974, 1730, 1668, 1577, 1516, 1473, 1428, 1396, 1351, 1272, 1240, 1184, 1119, 1044, 915, 844, 796, 748, 708.

HRMS (APCI) for $C_{14}H_{16}NO$ $[M+H]^+$: calculated 214.1226, found 214.1226.

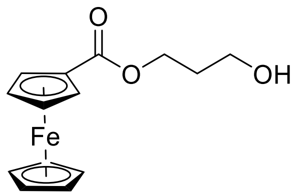

4e
$C_{14}H_{16}FeO_3$
M = 288.12 g/mol

3-Hydroxypropyl ferrocene carboxylate (4e): Prepared from ferrocenecarboxylic acid (2.3 g, 10 mmol) and 1,3-diol (2.3 g, 30 mmol) according to ***Method C***. Purification by flash column chromatography on silica gel using *n*-pentane:ethyl acetate = 2:1 afforded **4e** as a brown oil (2.5 g, 86% yield).

$\mathbf{R_f}$ = 0.30 (*n*-pentane:ethyl acetate = 2:1).

^{1}H NMR (500 MHz, $CDCl_3$) δ 4.77 (s, 2H), 4.37–4.33 (m, 4H), 4.17 (s, 5H), 3.75 (d, *J* = 6.2 Hz, 2H), 2.70 (s, 1H), 1.95–1.93 (m, 2H) ppm.

^{13}C NMR (126 MHz, $CDCl_3$) δ 172.2, 71.3, 70.8, 70.0, 69.6, 61.2, 59.1, 31.9 ppm.

IR (ATR): ṽ/cm^{-1} = 3928, 3419, 3096, 2239, 2120, 1896, 1685, 1457, 1373, 1272, 1135, 1048, 970, 929, 820, 751, 665.

HRMS (APCI) for $C_{14}H_{17}FeO_3$ $[M+H]^+$: calculated 289.0516, found 289.0520.

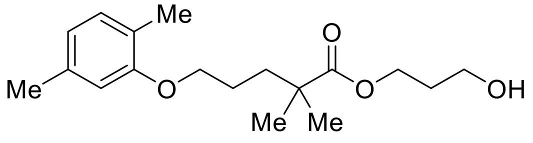

6b
$C_{18}H_{28}O_4$
M = 308.42 g/mol

3-Hydroxypropyl 5-(2,5-dimethylphenoxy)-2,2-dimethylpentanoate (6b): Prepared from 5-(2,5-dimethylphenoxy)-2,2-dimethylpentanoic acid (2.5 g, 10 mmol) and 1,3-diol (2.3 g, 30 mmol) according to ***Method D***. Purification by flash column chromatography on silica gel using *n*-pentane:ethyl acetate = 4:1 afforded **6b** as a colorless oil (2.7 g, 88% yield).

$\mathbf{R_f}$ = 0.30 (*n*-pentane:ethyl acetate = 2:1).

^{1}H NMR (500 MHz, $CDCl_3$) δ 7.00 (d, *J* = 7.5 Hz, 1H), 6.66 (d, *J* = 7.5 Hz, 1H), 6.61 (s, 1H), 4.23 (t, *J* = 6.1 Hz, 2H), 3.91 (d, *J* = 5.5 Hz, 2H), 3.67 (t, *J* = 6.1 Hz, 2H), 2.31 (s, 3H), 2.24 (s, 1H), 2.18 (s, 3H), 1.89–1.84 (m, 2H), 1.73 (s, 4H), 1.23 (s, 6H) ppm.

^{13}C NMR (126 MHz, $CDCl_3$) δ 178.2, 156.8, 136.4, 130.2, 123.5, 120.7, 111.9, 67.8, 61.2, 60.3, 59.0, 42.1, 37.0, 31.7, 25.1, 21.3, 15.7, 14.1 ppm.

IR (ATR): ṽ/cm^{-1} = 3446, 2952, 2924, 2353, 2223, 2122, 1723, 1613, 1584, 1508, 1472, 1389, 1310, 1262, 1192, 1149, 1046, 936, 844, 803, 753, 666.

HRMS (APCI) for $C_{18}H_{29}O_4$ $[M+H]^+$: calculated 309.2060, found 309.2060.

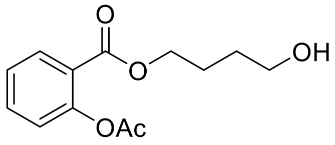

6c
$C_{13}H_{16}O_5$
M = 252.27 g/mol

4-Hydroxybutyl 2-acetoxybenzoate (6c): Prepared from Asprin (1.8 g, 10 mmol) and 1,4-diol (2.7 g, 30 mmol) according to ***Method C***. Purification by flash column chromatography on silica gel using *n*-pentane:ethyl acetate = 2:1 afforded **6c** as a colorless oil (1.9 g, 75% yield).

R$_f$ = 0.20 (*n*-pentane:ethyl acetate = 2:1).

^{1}H NMR (500 MHz, $CDCl_3$) δ 7.98 (d, *J* = 7.8 Hz, 1H), 7.53 (t, *J* = 7.8 Hz, 1H), 7.30–7.26 (m, 1H), 7.07 (d, *J* = 8.1 Hz, 1H), 4.28 (t, *J* = 6.6 Hz, 2H), 3.62 (t, *J* = 6.4 Hz, 2H), 2.32 (s, 3H), 2.29 (s, 1H), 1.82–1.76 (m, 2H), 1.67–1.61 (m, 2H) ppm.

^{13}C NMR (126 MHz, $CDCl_3$) δ 169.7, 164.4, 150.5, 133.8, 131.6, 126.0, 123.7, 123.3, 64.9, 62.0, 28.9, 25.0, 21.0 ppm.

IR (ATR): ṽ/cm^{-1} = 3512, 2944, 2240, 2125, 1766, 1715, 1606, 1451, 1368, 1293, 1253, 1188, 1133, 1077, 1039, 954, 914, 876, 816, 748, 703, 667.

HRMS (APCI) for $C_{13}H_{17}O_5$ $[M+H]^+$: calculated 253.1071, found 253.1069.

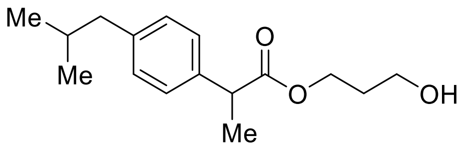

6d
$C_{16}H_{24}O_3$
M = 264.37 g/mol

3-Hydroxypropyl 2-(4-isobutylphenyl)propanoate (6d): Prepared from Ibuprofen (2.1 g, 10 mmol) and 1,3-diol (2.3 g, 30 mmol) according to ***Method D***. Purification by flash column chromatography on silica gel using *n*-pentane:ethyl acetate = 4:1 afforded **6d** as a colorless oil (2.3 g, 85% yield).

$\mathbf{R_f}$ = 0.40 (*n*-pentane:ethyl acetate = 2:1).

^{1}H NMR (500 MHz, $CDCl_3$) δ 7.06 (d, *J* = 8.1 Hz, 2H), 6.95 (d, *J* = 8.0 Hz, 2H), 4.04 (t, *J* = 6.3 Hz, 2H), 3.58–3.54 (m, 1H), 3.38 (t, *J* = 6.1 Hz, 2H), 3.20 (s, 1H), 2.31 (d, *J* = 7.5 Hz, 2H), 1.74–1.69 (m, 1H), 1.62 (t, *J* = 6.5 Hz, 2H), 1.34 (d, *J* = 7.5 Hz, 3H), 0.76 (d, *J* = 8.6 Hz, 6H) ppm.

^{13}C NMR (126 MHz, $CDCl_3$) δ 174.7, 140.1, 137.3, 128.9, 126.7, 61.3, 58.3, 44.7, 44.6, 31.2, 29.8, 22.0, 18.0 ppm.

IR (ATR): ṽ/cm^{-1} = 3452, 2953, 2310, 2075, 1903, 1730, 1630, 1511, 1460, 1382, 1332, 1247, 1163, 1053, 970, 929, 847, 798, 726.

HRMS (APCI) for $C_{16}H_{25}O_3$ $[M+H]^+$: calculated 265.1798, found 265.1799.

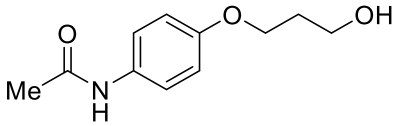

6e
$C_{11}H_{15}NO_3$
M = 209.25 g/mol

N*-(4-(3-hydroxypropoxy)phenyl)acetamide (6e):** Prepared from *N*-(4-hydroxyphenyl)acetamide (1.5 g, 10 mmol) and 3-bromopropan-1-ol (2.8 g, 20 mmol) according to ***Method G. Purification by flash column chromatography on silica gel using *n*-pentane:ethyl acetate = 3:2 afforded **6e** as a white solid (1.5 g, 71% yield).

$\mathbf{R_f}$ = 0.20 (*n*-pentane:ethyl acetate = 1:1).

M.p.: 102–104 °C.

^{1}H NMR (500 MHz, $CDCl_3$) δ 7.37 (d, *J* = 8.5 Hz, 2H), 7.12 (s, 1H), 6.86 (d, *J* = 8.8 Hz, 2H), 4.10 (t, *J* = 6.0 Hz, 2H), 3.86 (t, *J* = 5.8 Hz, 2H), 2.15 (s, 3H), 2.04–2.02 (m, 2H), 1.68 (s, 1H) ppm.

^{13}C NMR (126 MHz, $CDCl_3$) δ 168.1, 155.6, 131.1, 121.9, 114.8, 66.1, 60.5, 32.0, 24.4 ppm.

IR (ATR): ṽ/cm^{-1} = 3223, 3132, 3076, 2933, 2872, 2520, 2302, 2098, 1880, 1746, 1648, 1610, 1556, 1509, 1411, 1368, 1275, 1243, 1174, 1096, 1062, 1032, 962, 931, 884, 827, 751.

HRMS (APCI) for $C_{11}H_{16}NO_3$ [M+H]$^+$: calculated 210.1125, found 210.1125.

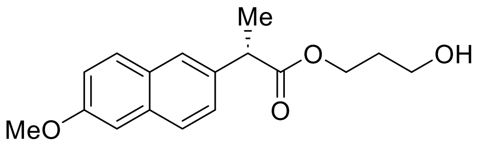

6f
$C_{17}H_{20}O_4$
M = 288.34 g/mol

3-Hydroxypropyl (*S*)-2-(6-methoxynaphthalen-2-yl)propanoate (6f): Prepared from Naproxen (2.3 g, 10 mmol) and 1,3-diol (2.3 g, 30 mmol) according to ***Method D***. Purification by flash column chromatography on silica gel using *n*-pentane:ethyl acetate = 4:1 afforded **6f** as a white solid (2.6g, 91% yield).

R$_f$ = 0.30 (*n*-pentane:ethyl acetate = 2:1).

M.p.: 52–54 °C.

^{1}H NMR (500 MHz, $CDCl_3$) δ 7.69 (d, *J* = 8.6 Hz, 2H), 7.66 (s, 1H), 7.40 (d, *J* = 8.6 Hz, 1H), 7.14 (dd, *J* = 8.9, 2.5 Hz, 1H), 7.11 (s, 1H), 4.23 (t, *J* = 6.2 Hz, 2H), 3.91 (s, 3H), 3.85 (t, *J* = 7.1 Hz, 1H), 3.53 (t, *J* = 6.1 Hz, 2H), 1.82–1.77 (m, 3H), 1.58 (d, *J* = 7.2 Hz, 3H) ppm.

^{13}C NMR (126 MHz, $CDCl_3$) δ 175.1, 157.6, 135.5, 133.7, 129.2, 128.9, 127.2, 126.0, 125.9, 119.0, 105.6, 61.7, 59.1, 55.3, 45.4, 31.6, 18.4 ppm.

IR (ATR): ṽ/cm^{-1} = 3497, 2935, 2870, 2615, 2291, 2084, 1918, 1794, 1710, 1603, 1482, 1453, 1388, 1334, 1260, 1190, 1092, 1057, 1028, 964, 920, 856, 818, 687.

HRMS (APCI) for $C_{17}H_{21}O_4$ [M+H]$^+$: calculated 289.1434, found 289.1432.

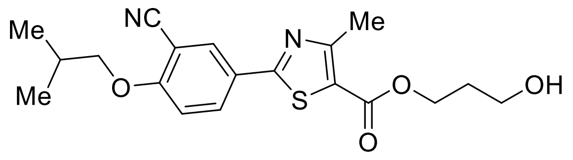

6g
$C_{19}H_{22}N_2O_4S$
M = 374.46 g/mol

3-Hydroxypropyl 2-(3-cyano-4-isobutoxyphenyl)-4-methylthiazole-5-carboxylate (6g): Prepared from Febuxostat (3.2 g, 10.0 mmol) and 1,3-diol (2.3 g, 30 mmol) according to ***Method D***. Purification by flash column chromatography on silica gel using *n*-pentane:ethyl acetate = 1:1 afforded **6g** as a white solid (3.2 g, 86% yield).

$\mathbf{R_f}$ = 0.25 (*n*-pentane:ethyl acetate = 1:1).

M.p.: 115–117 °C.

^{1}H NMR (500 MHz, $CDCl_3$) δ 8.16 (s, 1H), 8.08 (d, *J* = 9.1 Hz, 1H), 7.00 (d, *J* = 8.9 Hz, 1H), 4.46 (t, *J* = 6.1 Hz, 2H), 3.89 (d, *J* = 6.5 Hz, 2H), 3.78 (t, *J* = 6.0 Hz, 2H), 2.75 (s, 3H), 2.24–2.16 (m, 1H), 2.02–1.97 (m, 3H), 1.09 (d, *J* = 6.8 Hz, 6H) ppm.

^{13}C NMR (126 MHz, $CDCl_3$) δ 167.4, 162.6, 162.2, 161.3, 132.6, 132.1, 125.7, 121.5, 115.3, 112.6, 103.0, 75.7, 62.2, 59.0, 31.7, 28.1, 19.0, 17.4 ppm.

IR (ATR): $\tilde{v}$/cm^{-1} = 3319, 2954, 2873, 2647, 2342, 2227, 2119, 1996, 1734, 1689, 1600, 1508, 1469, 1428, 1393, 1339, 1270, 1174, 1107, 1047, 1010, 949, 919, 812.

HRMS (APCI) for $C_{19}H_{23}N_2O_4S$ $[M+H]^+$: calculated 375.1373, found 375.1374.

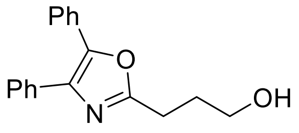

6h
$C_{18}H_{17}NO_2$
M = 279.34 g/mol

3-(4,5-Diphenyloxazol-2-yl)propan-1-ol (6h): Prepared from Oxaprozin (1.5 g, 5.0 mmol) and $LiAlH_4$ (0.50 g, 13 mmol) according to ***Method P***. Purification by flash column chromatography on silica gel using *n*-pentane:ethyl acetate = 2:1 afforded **6h** as a yellow solid (1.8 g, 65% yield).

$\mathbf{R_f}$ = 0.20 (*n*-pentane:ethyl acetate = 2:1).

M.p.: 90–92 °C.

^{1}H NMR (500 MHz, $CDCl_3$) δ 7.62 (d, *J* = 7.4 Hz, 2H), 7.57 (d, *J* = 7.3 Hz, 2H), 7.35 (d, *J* = 9.4 Hz, 6H), 3.80 (s, 2H), 2.99 (t, *J* = 7.1 Hz, 2H), 2.10 (t, *J* = 6.6 Hz, 2H) ppm.

^{13}C NMR (126 MHz, $CDCl_3$) δ 163.5, 145.3, 134.7, 132.2, 128.9, 128.6, 128.5, 128.4, 128.1, 127.8, 126.4, 61.9, 29.4, 25.3 ppm.

IR (ATR): ṽ/cm^{-1} = 3325, 3057, 2926, 2869, 2646, 2341, 2091, 1893, 1815, 1720, 1566, 1500, 1443, 1369, 1317, 1285, 1218, 1141, 1059, 987, 962, 919, 757, 692.

HRMS (APCI) for $C_{18}H_{18}NO_2$ $[M+H]^+$: calculated 280.1332, found 280.1329.

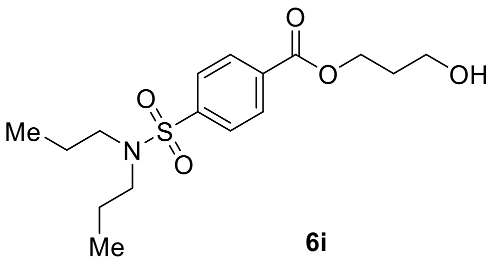

6i
$C_{16}H_{25}NO_5S$
M = 343.44 g/mol

3-Hydroxypropyl 4-(*N*,*N*-dipropylsulfamoyl)benzoate (6i): Prepared from probenecid (2.9 g, 10 mmol) and 1,3 diol (2.3 g, 30 mmol) according to ***Method C***. Purification by flash column chromatography on silica gel using *n*-pentane:ethyl acetate = 4:1 afforded **6i** as a colorless oil (3.1 g, 89% yield).

R_f = 0.30 (*n*-pentane:ethyl acetate = 2:1).

^{1}H NMR (500 MHz, $CDCl_3$) δ 8.13 (d, *J* = 8.5 Hz, 2H), 7.85 (d, *J* = 8.4 Hz, 2H), 4.50 (t, *J* = 6.2 Hz, 2H), 3.77 (t, *J* = 6.1 Hz, 2H), 3.10–3.06 (m, 4H), 2.04–1.99 (m, 3H), 1.56–1.49 (m, 4H), 0.85 (t, *J* = 7.4 Hz, 6H) ppm.

^{13}C NMR (126 MHz, $CDCl_3$) δ 165.5, 144.3, 133.4, 130.2, 126.9, 62.4, 59.0, 49.9, 31.7, 21.8, 11.1 ppm.

IR (ATR): ṽ/cm^{-1} = 3539, 3443, 3067, 2964, 2933, 2876, 2290, 1909, 1719, 1599, 1463, 1397, 1338, 1270, 1156, 1107, 1085, 1044, 990, 924, 863, 764, 737, 693.

HRMS (APCI) for $C_{16}H_{26}NO_5S$$[M+H]^+$: calculated 344.1526, found 344.1526.

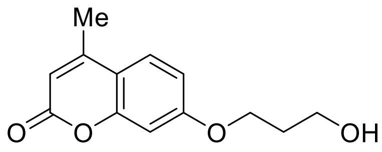

6j
$C_{13}H_{14}O_4$
M = 234.25 g/mol

7-(3-Hydroxypropoxy)-4-methyl-2*H*-chromen-2-one (6j): Prepared from Hymecromone (1.8 g, 10 mmol) and 3-bromopropan-1-ol (2.8 g, 20 mmol) according to ***Method G***. Purification by flash column chromatography on silica gel using *n*-pentane:ethyl acetate = 1:1 afforded **6j** as a white solid (2.1 g, 88% yield).

R_f = 0.15 (*n*-pentane:ethyl acetate = 1:1).

M.p.: 96–98 °C.

^{1}H NMR (500 MHz, $CDCl_3$) δ 7.47 (d, J = 8.8 Hz, 1H), 6.84 (d, J = 24.4 Hz, 2H), 6.11 (s, 1H), 4.17 (t, J = 6.1 Hz, 2H), 3.87 (t, J = 6.0 Hz, 2H), 2.38 (s, 3H), 2.10–2.05 (m, 2H) ppm.

^{13}C NMR (126 MHz, $CDCl_3$) δ 161.9, 161.4, 155.1, 152.6, 125.5, 113.5, 112.5, 111.8, 101.4, 65.7, 59.6, 31.8, 18.6 ppm.

IR (ATR): $\tilde{v}$/cm^{-1} = 3226, 2954, 2890, 2403, 2306, 2103, 1898, 1714, 1604, 1511, 1473, 1388, 1283, 1203, 1141, 1038, 996, 879, 828, 771, 705.

HRMS (APCI) for $C_{13}H_{15}O_4$ $[M+H]^+$: calculated 235.0965, found 235.0965.

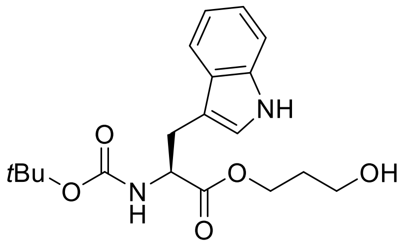

6k
$C_{19}H_{26}N_2O_5$
M = 362.43 g/mol

3-Hydroxypropyl (*tert*-butoxycarbonyl)-*L*-tryptophanate (6k): Prepared from (tert-butoxycarbonyl)-*L*-tryptophan (3.0 g, 10 mmol) and 1,3-diol (2.3 g, 30 mmol) according to ***Method D***. Purification by flash column chromatography on silica gel using *n*-pentane:ethyl acetate = 1:9 afforded **6k** as a white solid (2.4 g, 65% yield).

R_f = 0.40 (DCM:Methanol = 9:1).

M.p.: 127–129 °C.

^{1}H NMR (500 MHz, $CDCl_3$) δ 8.26 (s, 1H), 7.57 (d, *J* = 7.9 Hz, 1H), 7.35 (d, *J* = 8.1 Hz, 1H), 7.19 (t, *J* = 7.5 Hz, 1H), 7.13 (t, *J* = 7.5 Hz, 1H), 7.02 (s, 1H), 5.10 (s, 1H), 4.61 (s, 1H), 4.22–4.12 (m, 2H), 3.46–3.42 (m, 2H), 3.27 (d, *J* = 6.0 Hz, 2H), 1.89 (s, 1H), 1.75–1.70 (m, 2H), 1.43 (s, 9H) ppm.

^{13}C NMR (126 MHz, $CDCl_3$) δ 172.8, 155.3, 136.1, 127.6, 122.8, 122.2, 119.6, 118.8, 111.2, 110.1, 80.0, 62.2, 58.8, 54.4, 31.3, 28.3, 28.1 ppm.

IR (ATR): ṽ/cm^{-1} = 3458, 3335, 3059, 2963, 2926, 2230, 2079, 1993, 1918, 1726, 1683, 1507, 1456, 1354, 1298, 1220, 1158, 1057, 1017, 924, 848, 784, 738, 658.

HRMS (APCI) for $C_{19}H_{27}N_2O_5$ $[M+H]^+$: calculated 363.1914, found 363.1914.

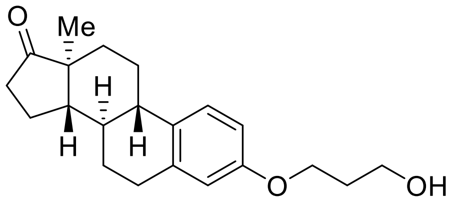

6l
$C_{21}H_{28}O_3$
M = 328.45 g/mol

(8*R*,9*S*,13*S*,14*S*)-3-(3-Hydroxypropoxy)-13-methyl-6,7,8,9,11,12,13,14,15,16-decahydro-17*H*-cyclopenta[*a*]phenanthren-17-one (6l): Prepared from estrone (2.7 g, 10 mmol) and 3-bromopropan-1-ol (2.8 g, 20 mmol) according to ***Method G***. Purification by flash column chromatography on silica gel using *n*-pentane:ethyl acetate = 4:1 afforded **6l** as a white solid (2.5 g, 77% yield).

R_f = 0.40 (*n*-pentane:ethyl acetate = 2:1).

M.p.: 92–94 °C.

^{1}H NMR (500 MHz, $CDCl_3$) δ 7.20 (d, *J* = 8.6 Hz, 1H), 6.72 (d, *J* = 8.7 Hz, 1H), 6.66 (s, 1H), 4.10 (t, *J* = 5.9 Hz, 2H), 3.86 (t, *J* = 5.8 Hz, 2H), 2.94–2.88 (m, 2H), 2.50 (dd, *J* = 19.0, 8.7 Hz, 1H), 2.41–2.37 (m, 1H), 2.27–2.22 (m, 1H), 2.18–2.10 (m, 1H), 2.08–1.99 (m, 4H), 1.95 (d, *J* = 9.4 Hz, 1H), 1.83 (s, 1H), 1.66–1.39 (m, 6H), 0.91 (s, 3H) ppm.

^{13}C NMR (126 MHz, $CDCl_3$) δ 220.9, 156.7, 137.8, 132.3, 126.3, 114.6, 112.1, 65.9, 60.7, 50.4, 48.0, 43.9, 38.3, 35.8, 32.0, 31.5, 29.6, 26.5, 25.9, 21.6, 13.8 ppm.

IR (ATR): ṽ/cm^{-1} = 3412, 2918, 2857, 2633, 2306, 2097, 1871, 1723, 1610, 1572, 1498, 1409, 1378, 1341, 1253, 1158, 1105, 1057, 1004, 960, 872, 812, 790, 736.

HRMS (APCI) for $C_{21}H_{28}O_3$ $[M+H]^+$: calculated 328.2038, found 328.2033.

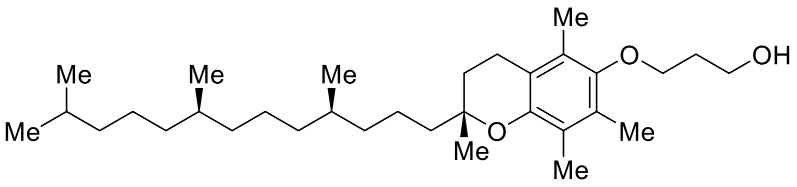

6m
$C_{32}H_{56}O_3$
M = 488.80 g/mol

3-(((*R*)-2,5,7,8-Tetramethyl-2-((4*R*,8*R*)-4,8,12-trimethyltridecyl)chroman-6-yl)oxy)propan-1-ol (6m): Prepared from α-tocopherol (2.2 g, 5.0 mmol) and 3-bromopropan-1-ol (1.4 g, 10 mmol) according to ***Method G***. Purification by flash column chromatography on silica gel using *n*-pentane:ethyl acetate = 2:1 afforded **6m** as a colorless oil (2.3 g, 95% yield).

R_f = 0.35 (*n*-pentane:ethyl acetate = 2:1).

^{1}H NMR (500 MHz, $CDCl_3$) δ 3.97(t, *J* = 5.8 Hz, 2H), 3.85 (t, *J* = 5.9 Hz, 2H), 2.75 (s, 1H), 2.61 (t, *J* = 6.9 Hz, 2H), 2.22 (s, 3H), 2.18 (s, 3H), 2.13 (s, 3H), 2.09–2.05 (m, 2H), 1.88–1.76 (m, 2H), 1.64–1.53 (m, 3H), 1.52–1.27 (m, 14H), 1.22–1.09 (m, 6H), 0.92–0.89 (m, 13H) ppm.

^{13}C NMR (126 MHz, $CDCl_3$) δ 147.8, 147.8, 127.6, 125.6, 122.8, 117.4, 77.2, 74.7, 71.5, 61.4, 39.9, 39.3, 37.4, 37.3, 37.2, 32.7, 32.6, 31.2, 27.9, 24.7, 24.4, 23.8, 22.6, 22.5, 20.9, 20.6, 19.7, 19.6, 12.6, 11.7, 11.7 ppm.

IR (ATR): ṽ/cm^{-1} = 3400, 2923, 2866, 2609, 2279, 2059, 2006, 1743, 1575, 1457, 1414, 1376, 1253, 1157, 1086, 1057, 981, 944, 858, 755, 667.

HRMS (APCI) for $C_{32}H_{57}O_3$ $[M+H]^+$: calculated 489.4302, found 489.4294.

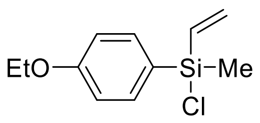

2c
$C_{11}H_{15}ClOSi$
M = 226.77 g/mol

Chloro(4-ethoxyphenyl)(methyl)(vinyl)silane (2c): Prepared from 1-bromo-4-ethoxybenzene (4.0 g, 20 mmol) according to ***Method Q***. Purification by short-path distillation (160 °C, 3.0×10^{-1} mbar) afforded **2c** as a colorless oil (1.1 g, 48% yield).

^{1}H NMR (500 MHz, $CDCl_3$) δ 7.56 (d, *J* = 8.7 Hz, 2H), 6.95 (d, *J* = 8.7 Hz, 2H), 6.33 (dd, *J* = 20.1, 14.6 Hz, 1H), 6.20 (dd, *J* = 14.5, 3.3 Hz, 1H), 5.96 (dd, *J* = 19.9, 3.4 Hz, 1H), 4.07 (q, *J* = 7.0 Hz, 2H), 1.43 (t, *J* = 7.0 Hz, 3H), 0.74 (s, 3H) ppm.

^{13}C NMR (126 MHz, $CDCl_3$) δ 160.9, 135.6, 135.3, 134.7, 125.1, 114.4, 63.3, 14.7, 0.5 ppm.

IR (ATR): ṽ/cm^{-1} = 3443, 2964, 2932, 2874, 2516, 2300, 2114, 2077, 1891, 1720, 1593, 1501, 1459, 1397, 1343, 1269, 1157, 1108, 1043, 990, 866, 795, 737, 693.

HRMS (APCI) for $C_{11}H_{15}OSi$ [M–Cl]$^{+}$: calculated 191.0887, found 191.0885.

5.2 Characterization data of deoxygenative silylated products

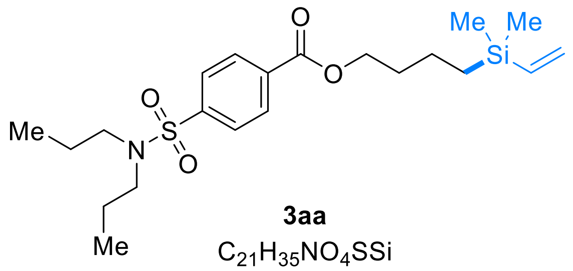

3aa
$C_{21}H_{35}NO_4SSi$
M = 425.66 g/mol

4-(Dimethyl(vinyl)silyl)butyl 4-(*N*,*N*-dipropylsulfamoyl)benzoate (3aa): Prepared from **1a** (75 mg, 0.20 mmol) and **2a** (72 mg, 0.60 mmol) according to **GP**. Purification by flash column chromatography on silica gel using *n*-pentane:ethyl acetate = 97:3 afforded **3aa** as a colorless oil (78 mg, 91% yield).

R_f = 0.40 (*n*-pentane:ethyl acetate = 90:10).

^{1}H NMR (500 MHz, $CDCl_3$) δ 8.13 (d, *J* = 8.4 Hz, 2H), 7.86 (d, *J* = 8.4 Hz, 2H), 6.12(dd, *J* = 20.3, 14.7 Hz, 1H), 5.94 (dd, *J* = 14.8, 3.8 Hz, 1H), 5.66 (dd, *J* = 20.3, 3.8 Hz, 1H), 4.34 (t, *J* =

6.7 Hz, 2H), 3.11–3.07 (m, 4H), 1.82–1.77 (m, 2H), 1.57–1.53 (m, 4H), 1.50–1.43 (m, 2H), 0.85 (t, *J* = 7.4 Hz, 6H), 0.64–0.61 (m, 2H), 0.06 (s, 6H) ppm.

^{13}C NMR (126 MHz, $CDCl_3$) δ 165.2, 144.1, 138.8, 133.8, 131.6, 130.1, 126.9, 65.3, 49.9, 32.1, 21.9, 20.2, 14.9, 11.1, –3.5 ppm.

IR (ATR): ṽ/cm^{-1} = 3444, 2960, 2875, 2478, 2299, 2118, 1935, 1720, 1598, 1464, 1398, 1343, 1269, 1157, 1106, 990, 950, 833, 763, 738, 693.

HRMS (APCI) for $C_{21}H_{36}NO_4SSi^+$ [M+H]$^+$: calculated 426.2129, found 426.2124.

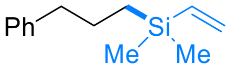

3ba
$C_{13}H_{20}Si$
M = 204.39 g/mol

Dimethyl(3-phenylpropyl)(vinyl)silane (3ba): Prepared from **1b** (27 mg, 0.20 mmol) and **2a** (72 mg, 0.60 mmol) according to **GP**. Purification by flash column chromatography on silica gel using *n*-pentane afforded **3ba** as a colorless oil (37 mg, 91% yield).

Product **3ba** was obtained as colorless oil (155 mg, 76% yield) when **1b** was used on a 1.0 mmol scale. The characterization data matched those obtained on 0.20 mmol scale (*vide infra*).

R_f = 0.95 (*n*-pentane).

^{1}H NMR (500 MHz, $CDCl_3$) δ 7.31–7.28 (m, 2H), 7.20 (d, *J* = 8.1 Hz, 3H), 6.16 (dd, *J* = 20.3, 14.7 Hz, 1H), 5.97 (dd, *J* = 14.7, 3.8 Hz, 1H), 5.68 (dd, *J* = 20.3, 3.9 Hz, 1H), 2.65 (t, *J* = 7.7 Hz, 2H), 1.69–1.62 (m, 2H), 0.66–0.62 (m, 2H), 0.08 (s, 6H) ppm.

^{13}C NMR (126 MHz, $CDCl_3$) δ 142.6, 139.1, 131.5, 128.5, 128.2, 125.6, 39.8, 26.0, 15.3, –3.5 ppm.

IR (ATR): ṽ/cm^{-1} = 3026, 2924, 2857, 2062, 1602, 1495, 1453, 1404, 1343, 1248,

1169, 1041, 1007, 950, 832, 743, 696

HRMS (APCI) for $C_{11}H_{17}Si^+$ [M–C_2H_3]$^+$: calculated 177.1094, found 177.1096.

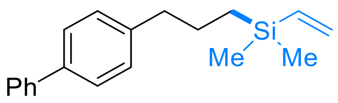

3ca
$C_{19}H_{24}Si$
M = 280.49 g/mol

(3-([1,1'-Biphenyl]-4-yl)propyl)dimethyl(vinyl)silane (3ca): Prepared from **1c** (43 mg, 0.20 mmol) and **2a** (72 mg, 0.60 mmol) according to **GP**. Purification by flash column chromatography on silica gel using *n*-pentane afforded **3ca** as a colorless oil (50 mg, 89% yield).

$\mathbf{R_f}$ = 0.85 (*n*-pentane).

^{1}H NMR (500 MHz, $CDCl_3$) δ 7.62 (d, *J* = 6.7 Hz, 2H), 7.55 (d, *J* = 8.2 Hz, 2H), 7.45 (t, *J* = 7.6 Hz, 2H), 7.35 (t, *J* = 7.4 Hz, 1H), 7.27 (d, *J* = 8.1 Hz, 2H), 6.18 (dd, *J* = 20.3, 14.7 Hz, 1H), 5.99 (dd, *J* = 14.7, 3.9 Hz, 1H), 5.70 (dd, *J* = 20.3, 3.9 Hz, 1H), 2.70 (t, *J* = 7.7 Hz, 2H), 1.74–1.67 (m, 2H), 0.70–0.67 (m, 2H), 0.10 (s, 6H) ppm.

^{13}C NMR (126 MHz, $CDCl_3$) δ 141.8, 141.2, 139.1, 138.6, 131.5, 128.9, 128.7, 127.0, 39.4, 26.0, 15.3, –3.4 ppm.

IR (ATR): $\tilde{\nu}/cm^{-1}$ = 3026, 2922, 2853, 2662, 2340, 2091, 1943, 1901, 1726, 1958, 1485, 1448, 1404, 1247, 1122, 1073, 1007, 948, 831, 757, 694

HRMS (APCI) for $C_{19}H_{25}Si^{+}$ $[M+H]^{+}$: calculated 281.1720, found 281.1718.

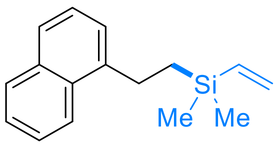

3da
$C_{16}H_{20}Si$
M = 240.42 g/mol

Dimethyl(2-(naphthalen-1-yl)ethyl)(vinyl)silane (3da): Prepared from **1d** (35 mg, 0.20 mmol) and **2a** (72 mg, 0.60 mmol) according to **GP**. Purification by flash column chromatography on silica gel using *n*-pentane afforded **3da** as a colorless oil (27 mg, 56% yield).

$\mathbf{R_f}$ = 0.85 (*n*-pentane).

^{1}H NMR (500 MHz, $CDCl_3$) δ 8.04 (d, *J* = 8.4 Hz, 1H), 7.88 (d, *J* = 8.0 Hz, 1H), 7.72 (d, *J* = 8.1 Hz, 1H), 7.51 (dt, *J* = 14.8, 7.7 Hz, 2H), 7.42 (t, *J* = 7.6 Hz, 1H), 7.37 (d, *J* = 7.1 Hz, 1H), 6.27

(dd, *J* = 20.4, 14.7 Hz, 1H), 6.06 (dd, *J* = 14.7, 3.7 Hz, 1H), 5.80 (dd, *J* = 20.3, 3.7 Hz, 1H), 3.13–3.10 (m, 2H), 1.12–1.09 (m, 2H), 0.20 (s, 6H) ppm.

^{13}C NMR (126 MHz, $CDCl_3$) δ 141.3, 138.7, 134.0, 132.0, 131.6, 128.8, 126.3, 125.6, 125.6, 125.3, 124.9, 123.7, 27.1, 17.1, –3.5 ppm.

IR (ATR): ṽ/cm^{-1} = 3045, 2952, 2306, 2090, 1910, 1594, 1509, 1458, 1401, 1247, 1173, 1051, 1007, 949, 907, 820, 782, 702.

HRMS (APCI) for $C_{16}H_{20}Si^+$ $[M]^+$: calculated 240.1329, found 240.1330.

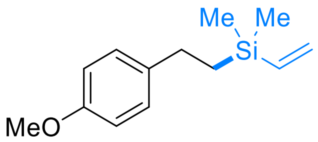

3ea

$C_{13}H_{20}OSi$

M = 220.39 g/mol

(4-Methoxyphenethyl)dimethyl(vinyl)silane (3ea): Prepared from **1e** (30 mg, 0.20 mmol) and **2a** (72 mg, 0.60 mmol) according to **GP**. Purification by flash column chromatography on silica gel using *n*-pentane:ethyl acetate = 99:1 afforded **3ea** as a colorless oil (34 mg, 76% yield).

R_f = 0.80 (*n*-pentane:ethyl acetate = 90:10).

^{1}H NMR (500 MHz, $CDCl_3$) δ 7.13 (d, *J* = 8.3 Hz, 2H), 6.84 (d, *J* = 8.2 Hz, 2H), 6.17 (dd, *J* = 20.3, 14.6 Hz, 1H), 5.99 (dd, *J* = 14.7, 3.8 Hz, 1H), 5.72 (dd, *J* = 20.3, 3.8 Hz, 1H), 3.80 (s, 3H), 2.62–2.59 (m, 2H), 0.95–0.92 (m, 2H), 0.10 (s, 6H) ppm.

^{13}C NMR (126 MHz, $CDCl_3$) δ 157.5, 138.8, 137.2, 131.7, 128.6, 113.7, 55.2, 29.0, 17.6, –3.5 ppm.

IR (ATR): ṽ/cm^{-1} = 2953, 2475, 2323, 2112, 2067, 1988, 1877, 1728, 1610, 1510, 1462, 1404, 1242, 1175, 1125, 1037, 950, 903, 819, 776, 700.

HRMS (APCI) for $C_{13}H_{20}OSi^+$ $[M]^+$: calculated 220.1278, found 220.1280.

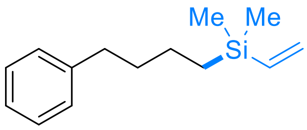

3fa
$C_{14}H_{22}Si$
M = 218.42 g/mol

Dimethyl(4-phenylbutyl)(vinyl)silane (3f): Prepared from **1f** (30 mg, 0.20 mmol) and **2a** (72 mg, 0.60 mmol) according to **GP**. Purification by flash column chromatography on silica gel using *n*-pentane afforded **3fa** as a colorless oil (39 mg, 90% yield).

R_f = 0.90 (*n*-pentane).

^{1}H NMR (500 MHz, $CDCl_3$) δ 7.29 (t, *J* = 7.6 Hz, 2H), 7.19 (d, *J* = 7.2 Hz, 3H), 6.16(dd, *J* = 20.4, 14.7 Hz, 1H), 5.96 (dd, *J* = 14.7, 3.9 Hz, 1H), 5.68 (dd, *J* = 20.3, 3.9 Hz, 1H), 2.62 (t, *J* = 7.9 Hz, 2H), 1.68–1.65 (m, 2H), 1.43–1.36 (m, 2H), 0.64–0.61 (m, 2H), 0.07 (s, 6H) ppm.

^{13}C NMR (126 MHz, $CDCl_3$) δ 142.9, 139.3, 131.4, 128.4, 128.2, 125.5, 35.7, 35.3, 23.6, 15.2, –3.4 ppm.

IR (ATR): $\tilde{v}$/cm^{-1} = 3026, 2923, 2854, 2669, 2101, 1938, 1602, 1495, 1453, 1404, 1341, 1247, 1167, 1007, 949, 832, 774, 743, 695.

HRMS (APCI) for $C_{12}H_{19}Si^+$ $[M–C_2H_3]^+$: calculated 191.1251, found 191.1252.

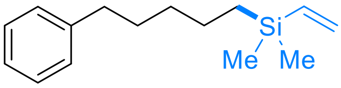

3ga
$C_{15}H_{24}Si$
M = 232.44 g/mol

Dimethyl(5-phenylpentyl)(vinyl)silane (3ga): Prepared from **1g** (33 mg, 0.20 mmol) and **2a** (72 mg, 0.60 mmol) according to **GP**. Purification by flash column chromatography on silica gel using *n*-pentane afforded **3ga** as a colorless oil (44 mg, 94% yield).

R_f = 0.90 (*n*-pentane).

^{1}H NMR (500 MHz, $CDCl_3$) δ 7.31–7.28 (m, 2H), 7.19 (d, *J* = 7.3 Hz, 3H), 6.16 (dd, *J* = 20.4, 14.6 Hz, 1H), 5.96 (dd, *J* = 14.7, 4.0 Hz, 1H), 5.68 (dd, *J* = 20.3, 3.9 Hz, 1H), 2.62 (t, *J* = 7.8 Hz, 2H), 1.66–1.61 (m, 2H), 1.38–1.37 (m, 4H), 0.60–0.57 (m, 2H), 0.07 (s, 6H) ppm.

^{13}C NMR (126 MHz, $CDCl_3$) δ 142.9, 139.3, 131.3, 128.4, 128.2, 125.5, 35.9, 33.1, 31.2, 23.7, 15.3, –3.4 ppm.

IR (ATR): ṽ/cm^{-1} = 3026, 2922, 2853, 2668, 2307, 2091, 1937, 1891, 1602, 1495, 1453, 1404, 1247, 1163, 1074, 1008, 948, 831, 767, 744, 696.

HRMS (APCI) for $C_{13}H_{21}Si^+$ $[M–C_2H_3]^+$: calculated 205.1407, found 205.1407.

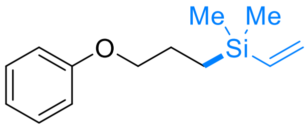

3ha
$C_{13}H_{20}OSi$
M = 220.39 g/mol

Dimethyl(3-phenoxypropyl)(vinyl)silane (3ha): Prepared from **1h** (31 mg, 0.20 mmol) and **2a** (72 mg, 0.60 mmol) according to **GP**. Purification by flash column chromatography on silica gel using *n*-pentane:ethyl acetate = 99:1 afforded **3ha** as a colorless oil (33 mg, 74% yield).

R$_f$ = 0.80 (*n*-pentane:ethyl acetate = 90:10).

^{1}H NMR (500 MHz, $CDCl_3$) δ 7.28 (dd, *J* = 8.8, 7.2 Hz, 2H), 6.92 (dd, *J* = 20.8, 7.5 Hz, 3H), 6.17 (dd, *J* = 20.3, 14.6 Hz, 1H), 5.98 (dd, *J* = 14.7, 3.7 Hz, 1H), 5.71 (dd, *J* = 20.3, 3.7 Hz, 1H), 3.92 (t, *J* = 6.9 Hz, 2H), 1.84–1.78 (m, 2H), 0.71–0.68 (m, 2H), 0.11 (s, 6H) ppm.

^{13}C NMR (126 MHz, $CDCl_3$) δ 159.1, 138.7, 131.8, 129.4, 120.4, 114.5, 70.4, 23.8, 11.4, –3.5 ppm.

IR (ATR): ṽ/cm^{-1} = 3197, 3043, 2934, 1923, 1599, 1495, 1404, 1299, 1241, 1170, 1008, 950, 891, 832, 749, 689.

HRMS (APCI) for $C_{11}H_{17}OSi^+$ $[M–C_2H_3]^+$: calculated 193.1043, found 193.1044.

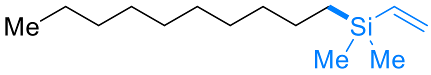

3ia
$C_{14}H_{30}Si$
M = 226.48 g/mol

Decyldimethyl(vinyl)silane (3ia): Prepared from **1i** (32 mg, 0.20 mmol) and **2a** (72 mg, 0.60 mmol) according to **GP**. Purification by flash column chromatography on silica gel using *n*-pentane afforded **3ia** as a colorless oil (33 mg, 73% yield).

R$_f$ = 0.95 (*n*-pentane).

^{1}H NMR (500 MHz, $CDCl_3$) δ 6.14 (dd, *J* = 20.3, 14.7 Hz, 1H), 5.93 (dd, *J* = 14.7, 3.8 Hz, 1H), 5.65 (dd, *J* = 20.3, 3.9 Hz, 1H), 1.29–1.27 (m, 16H), 0.89 (t, *J* = 6.8 Hz, 3H), 0.56 (s, 2H), 0.05(s, 6H) ppm.

^{13}C NMR (126 MHz, $CDCl_3$) δ 139.4, 131.2, 33.6, 31.9, 29.7, 29.6, 29.4, 23.8, 22.7, 15.4, 14.1, –3.4 ppm.

IR (ATR): $\tilde{v}$/cm^{-1} = 3047, 2920, 2852, 2668, 2314, 2066, 2001, 1896, 1592, 1462, 1404, 1248, 1169, 1051, 1007, 948, 833, 772, 704.

HRMS (APCI) for $C_{14}H_{29}Si^+$ $[M–H]^+$: calculated 225.2039, found 225.2031.

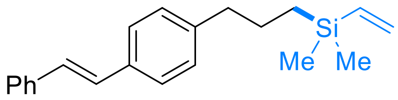

3ja
$C_{21}H_{26}Si$
M = 306.52 g/mol

(*E*)-dimethyl(3-(4-styrylphenyl)propyl)(vinyl)silane (3ja): Prepared from **1j** (48 mg, 0.20 mmol) and **2a** (72 mg, 0.60 mmol) according to **GP**. Purification by flash column chromatography on silica gel using *n*-pentane afforded **3ja** as a white solid (60 mg, 98% yield).

R_f = 0.80 (*n*-pentane).

M.p.: 56–58 °C.

^{1}H NMR (500 MHz, $CDCl_3$) δ 7.54 (d, *J* = 7.0 Hz, 2H), 7.47 (d, *J* = 8.1 Hz, 2H), 7.38 (t, *J* = 7.8 Hz, 2H), 7.30–7.26 (m, 1H), 7.20 (d, *J* = 7.9 Hz, 2H), 7.12 (d, *J* = 3.9 Hz, 2H), 6.18 (dd, *J* = 20.4, 14.7 Hz, 1H), 5.99 (dd, *J* = 14.7, 3.9 Hz, 1H), 5.70 (dd, *J* = 20.3, 3.9 Hz, 1H), 2.66 (t, *J* = 7.6 Hz, 2H), 1.71–1.65 (m, 2H), 0.68–0.65 (m, 2H), 0.10 (s, 6H) ppm.

^{13}C NMR (126 MHz, $CDCl_3$) δ 142.3, 139.0, 137.5, 134.8, 131.5, 128.8, 128.7, 128.6, 127.8, 127.4, 126.4, 126.4, 39.5, 25.9, 15.2, –3.4 ppm.

IR (ATR): $\tilde{v}$/cm^{-1} = 3022, 2918, 2854, 2650, 2305, 2111, 1898, 1794, 1700, 1592, 1510, 1446, 1403, 1340, 1246, 1166, 1083, 1006, 964, 820, 751, 738.

HRMS (APCI) for $C_{21}H_{26}Si^+$ $[M]^+$: calculated 306.1798, found 306.1800.

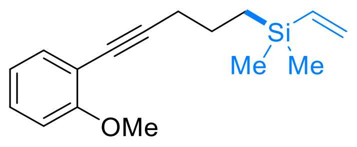

3ka
$C_{16}H_{22}OSi$
M = 258.44 g/mol

(5-(2-Methoxyphenyl)pent-4-yn-1-yl)dimethyl(vinyl)silane (3ka): Prepared from **1k** (39 mg, 0.20 mmol) and **2a** (72 mg, 0.60 mmol) according to **GP**. Purification by flash column chromatography on silica gel using *n*-pentane:ethyl acetate = 98:2 afforded **3ka** as a colorless oil (43 mg, 84% yield).

$\mathbf{R_f}$ = 0.70 (*n*-pentane:ethyl acetate = 90:10).

^{1}H NMR (500 MHz, $CDCl_3$) δ 7.39 (d, *J* = 7.1 Hz, 1H), 7.24 (t, *J* = 7.9 Hz, 1H), 6.88 (dd, *J* = 17.7, 7.9 Hz, 2H), 6.17 (dd, *J* = 20.4, 14.7 Hz, 1H), 5.97 (dd, *J* = 14.6, 3.9 Hz, 1H), 5.71 (dd, *J* = 20.3, 3.9 Hz, 1H), 3.88 (s, 3H), 2.49 (t, *J* = 7.1 Hz, 2H), 1.68–1.65 (m, 2H), 0.80–0.76 (m, 2H), 0.10 (s, 6H) ppm.

^{13}C NMR (126 MHz, $CDCl_3$) δ 159.8, 138.9, 133.6, 131.6, 128.8, 120.3, 113.2, 110.5, 94.5, 55.7, 23.6, 23.4, 15.0, –3.5 ppm.

IR (ATR): $\tilde{\nu}$/cm^{-1} = 3045, 2931, 2225, 2081, 1897, 1699, 1594, 1491, 1460, 1341, 1257, 1179, 1115, 1025, 950, 830, 747, 701.

HRMS (APCI) for $C_{16}H_{23}OSi^+$ [M+H]$^+$: calculated 259.1513, found 259.1514.

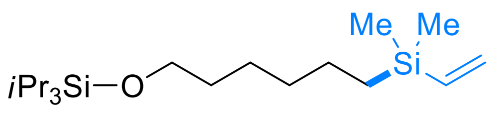

3la
$C_{19}H_{42}OSi_2$
M = 342.71 g/mol

((6-(Dimethyl(vinyl)silyl)hexyl)oxy)triisopropylsilane (3la): Prepared from **1l** (55 mg, 0.20 mmol) and **2a** (72 mg, 0.60 mmol) according to **GP**. Purification by flash column chromatography on silica gel using *n*-pentane afforded **3la** as a colorless oil (44 mg, 64% yield).

$\mathbf{R_f}$ = 0.60 (*n*-pentane).

^{1}H NMR (500 MHz, $CDCl_3$) δ 6.14 (dd, *J* = 20.3, 14.7 Hz, 1H), 5.94 (dd, *J* = 14.7, 4.0 Hz, 1H), 5.66 (dd, *J* = 20.3, 4.0 Hz, 1H), 3.67 (t, *J* = 6.7 Hz, 2H), 1.52 (q, *J* = 6.7 Hz, 2H), 1.32 (d, *J* = 4.8 Hz, 6H), 1.10–1.03 (m, 21H), 0.57–0.53 (m, 2H), 0.05 (s, 6H) ppm.

^{13}C NMR (126 MHz, $CDCl_3$) δ 139.4, 131.2, 63.5, 33.3, 32.9, 25.5, 23.8, 18.0, 15.3, 12.0, –3.4 ppm.

IR (ATR): ṽ/cm^{-1} = 2923, 2864, 2726, 2355, 2120, 1896, 1592, 1462, 1404, 1248, 1103, 1068, 1008, 949, 881, 834, 776, 678.

HRMS (APCI) for $C_{19}H_{43}OSi_2^+$ [M+H]$^+$: calculated 343.2847, found 343.2850.

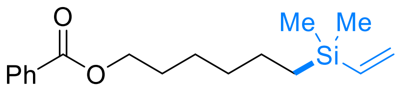

3ma
$C_{17}H_{26}O_2Si$
M = 290.48 g/mol

6-(Dimethyl(vinyl)silyl)hexyl benzoate (3ma): Prepared from **1m** (45 mg, 0.20 mmol) and **2a** (72 mg, 0.60 mmol) according to **GP**. Purification by flash column chromatography on silica gel using *n*-pentane:ethyl acetate = 98:2 afforded **3ma** as a colorless oil (40 mg, 68% yield).

R_f = 0.65 (*n*-pentane:ethyl acetate = 90:10).

^{1}H NMR (500 MHz, $CDCl_3$) δ 8.04 (d, *J* = 6.9 Hz, 2H), 7.56–7.53 (m, 1H), 7.44 (d, *J* = 7.5 Hz, 2H), 6.14 (dd, *J* = 20.4, 14.7 Hz, 1H), 5.94 (dd, *J* = 14.7, 3.9 Hz, 1H), 5.66 (dd, *J* = 20.3, 3.9 Hz, 1H), 4.31 (t, *J* = 6.6 Hz, 2H), 1.76 (t, *J* = 7.3 Hz, 2H), 1.45–1.34 (m, 6H), 0.71–0.28 (m, 2H), 0.05 (s, 6H) ppm.

^{13}C NMR (126 MHz, $CDCl_3$) δ 166.7, 139.2, 132.7, 131.3, 130.6, 129.5, 128.3, 65.1, 33.1, 28.7, 25.7, 23.7, 15.3, –3.4 ppm.

IR (ATR): ṽ/cm^{-1} = 3444, 3045, 2922, 2854, 2340, 2125, 1959, 1718, 1601, 1451, 1404, 1268, 1174, 1110, 1026, 949, 834, 779.

HRMS (APCI) for $C_{17}H_{27}O_2Si^+$ [M+H]$^+$: calculated 291.1775, found 291.1776.

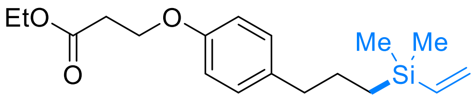

3na
$C_{18}H_{28}O_3Si$
M = 320.50 g/mol

Ethyl 3-(4-(3-(dimethyl(vinyl)silyl)propyl)phenoxy)propanoate (3na): Prepared from **1n** (51 mg, 0.20 mmol) and **2a** (72 mg, 0.60 mmol) according to **GP**. Purification by flash column chromatography on silica gel using *n*-pentane:ethyl acetate = 97:3 afforded **3na** as a colorless oil (33 mg, 52% yield).

R_f = 0.60 (*n*-pentane:ethyl acetate = 90:10).

^{1}H NMR (500 MHz, $CDCl_3$) δ 7.07 (d, *J* = 8.2 Hz, 2H), 6.83 (d, *J* = 8.1 Hz, 2H), 6.13(dd, *J* = 20.3, 14.7 Hz, 1H), 5.94 (dd, *J* = 14.7, 3.9 Hz, 1H), 5.65 (dd, *J* = 20.3, 3.9 Hz, 1H), 4.23 (t, *J* = 6.5 Hz, 2H), 4.18 (q, *J* = 7.2 Hz, 2H), 2.77 (t, *J* = 6.5 Hz, 2H), 2.56 (t, *J* = 7.6 Hz, 2H), 1.62–1.56 (m, 2H), 1.27 (d, *J* = 7.1 Hz, 3H), 0.61–0.58 (m, 2H), 0.05 (s, 6H) ppm.

^{13}C NMR (126 MHz, $CDCl_3$) δ 171.1, 156.6, 139.1, 135.2, 131.4, 129.3, 114.5, 63.6, 60.6, 38.9, 34.7, 26.1, 15.1, 14.2, –3.5 ppm.

IR (ATR): $\tilde{\nu}$/cm^{-1} = 3475, 2924, 2303, 2121, 1876, 1735, 1610, 1510, 1464, 1402, 1242, 1175, 1035, 949, 829, 703.

HRMS (APCI) for $C_{18}H_{28}O_3Si^+$ $[M]^+$: calculated 320.1802, found 320.1803.

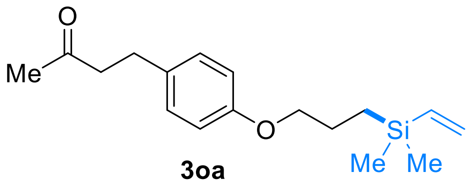

3oa
$C_{17}H_{26}O_2Si$
M = 290.48 g/mol

4-(4-(3-(Dimethyl(vinyl)silyl)propoxy)phenyl)butan-2-one (3oa): Prepared from **1o** (45 mg, 0.20 mmol) and **2a** (72 mg, 0.60 mmol) according to **GP**. Purification by flash column chromatography on silica gel using *n*-pentane:ethyl acetate = 98:2 afforded **3oa** as a colorless oil (41 mg, 70% yield).

R_f = 0.65 (*n*-pentane:ethyl acetate = 90:10).

^{1}H NMR (500 MHz, $CDCl_3$) δ 7.07 (d, *J* = 8.3 Hz, 2H), 6.80 (d, *J* = 8.1 Hz, 2H), 6.16 (dd, *J* = 20.2, 14.6 Hz, 1H), 5.97 (dd, *J* = 14.6, 3.9 Hz, 1H), 5.70 (dd, *J* = 20.3, 3.8 Hz, 1H), 3.88 (t, *J* = 6.8 Hz, 2H), 2.83 (t, *J* = 7.6 Hz, 2H), 2.72 (t, *J* = 7.6 Hz, 2H), 2.12 (s, 3H), 1.78 (dd, *J* = 16.4, 7.3 Hz, 2H), 0.69–0.66(m, 2H), 0.10 (s, 6H) ppm.

^{13}C NMR (126 MHz, $CDCl_3$) δ 208.1, 157.5, 138.7, 132.8, 131.8, 129.1, 114.5, 70.6, 45.4, 30.1, 28.9, 23.8, 11.3, –3.5 ppm.

IR (ATR): ṽ/cm^{-1} = 3424, 2928, 2109, 1881, 1715, 1611, 1510, 1470, 1405, 1360, 1296, 1241, 1175, 1108, 1048, 1009, 950, 892, 833, 770, 704.

HRMS (APCI) for $C_{17}H_{26}O_2Si^+$ [M]$^+$: calculated 290.1697, found 290.1699.

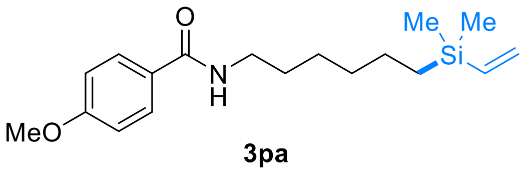

3pa
$C_{18}H_{29}NO_2Si$
M = 319.52 g/mol

***N*-(6-(dimethyl(vinyl)silyl)hexyl)-4-methoxybenzamide (3pa):** Prepared from **1p** (50 mg, 0.20 mmol) and **2a** (72 mg, 0.60 mmol) according to **GP**. Purification by flash column chromatography on silica gel using *n*-pentane:ethyl acetate = 85:15 afforded **3pa** as a colorless oil (49 mg, 76% yield).

R_f = 0.40 (*n*-pentane:ethyl acetate = 2:1).

^{1}H NMR (500 MHz, $CDCl_3$) δ 7.72 (d, *J* = 8.7 Hz, 2H), 6.90 (d, *J* = 8.6 Hz, 2H), 6.13 (dd, *J* = 20.3, 14.7 Hz, 2H), 5.93 (dd, *J* = 14.7, 3.9 Hz, 1H), 5.65 (dd, *J* = 20.3, 3.9 Hz, 1H), 3.83 (s, 3H), 3.41 (q, *J* = 6.7 Hz, 2H), 1.58 (t, *J* = 7.2 Hz, 2H), 1.35 (q, *J* = 7.0, 5.8 Hz, 6H), 0.56–0.53 (m, 2H), 0.04 (s, 6H) ppm.

^{13}C NMR (126 MHz, $CDCl_3$) δ 167.0, 162.0, 139.2, 131.3, 128.6, 127.1, 113.7, 55.3, 40.1, 33.2, 29.7, 26.7, 23.7, 15.3, –3.5 ppm.

IR (ATR): ṽ/cm^{-1} = 3314, 3046, 2920, 2852, 2291, 2054, 1900, 1724, 1630, 1543, 1502, 1460, 1404, 1297, 1249, 1177, 1109, 1032, 948, 835, 766, 686.

HRMS (APCI) for $C_{18}H_{30}NO_2Si^+$ [M+H]$^+$: calculated 320.2040, found 320.2043.

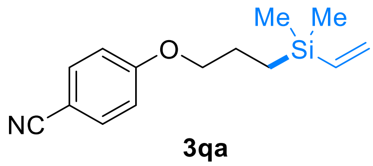

3qa
$C_{14}H_{19}NOSi$
M = 245.40 g/mol

4-(3-(Dimethyl(vinyl)silyl)propoxy)benzonitrile (3qa): Prepared from **1q** (35 mg, 0.20 mmol) and **2a** (72 mg, 0.60 mmol) according to **GP**. Purification by flash column chromatography on silica gel using *n*-pentane:ethyl acetate = 97:3 afforded **3qa** as a colorless oil (40 mg, 81% yield).

R_f = 0.50 (*n*-pentane:ethyl acetate = 90:10).

^{1}H NMR (500 MHz, $CDCl_3$) δ 7.56 (d, *J* = 8.9 Hz, 2H), 6.92 (d, *J* = 8.8 Hz, 2H), 6.04 (dd, *J* = 20.3, 14.7 Hz, 1H), 5.97 (dd, *J* = 14.7, 3.8 Hz, 1H), 5.70 (dd, *J* = 20.3, 3.8 Hz, 1H), 3.95 (t, *J* = 6.8 Hz, 2H), 1.84–1.78 (m, 2H), 0.69–0.66 (m, 2H), 0.10 (s, 6H) ppm.

^{13}C NMR (126 MHz, $CDCl_3$) δ 162.4, 138.4, 133.9, 133.9, 132.0, 119.3, 115.1, 103.7, 70.8, 23.5, 11.3, –3.6 ppm.

IR (ATR): ṽ/cm^{-1} = 3209, 3047, 2941, 2876, 2560, 2329, 2223, 2089, 1898, 1726, 1604, 1506, 1468, 1404, 1300, 1249, 1169, 1112, 1045, 1006, 951, 892, 830, 770, 703.

HRMS (APCI) for $C_{14}H_{20}NOSi^+$ $[M+H]^+$: calculated 246.1309, found 246.1309.

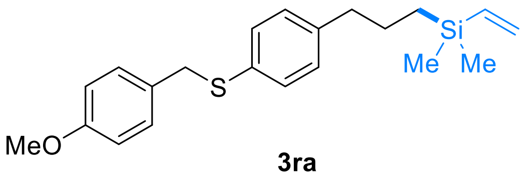

3ra
$C_{21}H_{28}OSSi$
M = 356.60 g/mol

(3-(4-((4-Methoxybenzyl)thio)phenyl)propyl)dimethyl(vinyl)silane (3ra): Prepared from **1r** (58 mg, 0.20 mmol) and **2a** (72 mg, 0.60 mmol) according to **GP**. Purification by flash column chromatography on silica gel using *n*-pentane:ethyl acetate = 98:2 afforded **3ra** as a colorless oil (64mg, 89% yield).

R_f = 0.70 (*n*-pentane:ethyl acetate = 90:10).

^{1}H NMR (500 MHz, $CDCl_3$) δ 7.24 (d, *J* = 8.2 Hz, 2H), 7.20 (d, *J* = 8.4 Hz, 2H), 7.07 (d, *J* = 7.9 Hz, 2H), 6.82 (d, *J* = 8.6 Hz, 2H), 6.13 (dd, *J* = 20.3, 14.7 Hz, 1H), 5.95 (dd, *J* = 14.7, 3.9 Hz,

1H), 5.67 (dd, J = 20.3, 3.9 Hz, 1H), 4.05 (s, 2H), 3.79 (s, 3H), 2.59(t, J = 7.6 Hz, 2H), 1.62–1.59 (m, 2H), 0.61–0.58(m, 2H), 0.06 (s, 6H) ppm.

^{13}C NMR (126 MHz, $CDCl_3$) δ 158.7, 141.1, 139.0, 133.1, 131.5, 130.4, 129.9, 129.7, 129.0, 113.8, 55.2, 39.2, 39.1, 25.8, 15.1, –3.5 ppm.

IR (ATR): ṽ/cm^{-1} = 3003, 2923, 2277, 2111, 2054, 1991, 1887, 1609, 1509, 1460, 1402, 1300, 1245, 1173, 1092, 1034, 950, 827, 700.

HRMS (APCI) for $C_{21}H_{27}OSSi^+$ [M–H]$^+$: calculated 355.1546, found 355.1543.

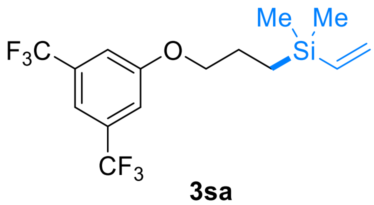

3sa

$C_{15}H_{18}F_6OSi$

M = 356.38 g/mol

(3-(3,5-Bis(trifluoromethyl)phenoxy)propyl)dimethyl(vinyl)silane (3sa): Prepared from **1s** (58 mg, 0.20 mmol) and **2a** (72 mg, 0.60 mmol) according to **GP**. Purification by flash column chromatography on silica gel using *n*-pentane afforded **3sa** as a colorless oil (63 mg, 88% yield).

R$_f$ = 0.65 (*n*-pentane).

^{1}H NMR (500 MHz, $CDCl_3$) δ 7.44 (s, 1H), 7.28 (s, 2H), 6.16 (dd, J = 20.4, 14.8 Hz, 1H), 5.99(dd, J = 14.7, 3.8 Hz, 1H), 5.72 (dd, J = 20.3, 3.8 Hz, 1H), 3.99 (t, J = 6.6 Hz, 2H), 1.86–1.80 (m, 2H), 0.72–0.69 (m, 2H), 0.12 (s, 6H) ppm.

^{13}C NMR (126 MHz, $CDCl_3$) δ 159.7, 138.4, 132.7 (q, J = 33.3 Hz), 132.1, 123.2 (q, J = 272.7 Hz), 114.8 (d, J = 4.1 Hz), 114.0 (p, J = 4.0 Hz), 71.3, 23.5, 11.3, –3.6 ppm.

^{19}F NMR (471 MHz, $CDCl_3$) δ –63.1 ppm.

IR (ATR): ṽ/cm^{-1} = 2944, 2324, 2123, 1983, 1761, 1612, 1462, 1369, 1275, 1171, 1129, 1028, 948, 835, 772, 701.

HRMS (APCI) for $C_{13}H_{15}F_6OSi^+$ [M–C_2H_3]$^+$: calculated 329.0796, found 329.0794.

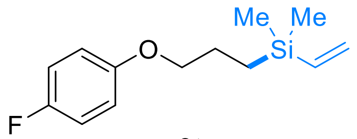

3ta
$C_{13}H_{19}FOSi$
M = 238.38 g/mol

(3-(4-Fluorophenoxy)propyl)dimethyl(vinyl)silane (3ta): Prepared from **1t** (34 mg, 0.20 mmol) and **2a** (72 mg, 0.60 mmol) according to **GP**. Purification by flash column chromatography on silica gel using *n*-pentane:ethyl acetate = 99:1 afforded **3ta** as a colorless oil (34 mg, 72% yield).

R_f = 0.60 (*n*-pentane).

^{1}H NMR (500 MHz, $CDCl_3$) δ 6.96 (t, *J* = 8.7 Hz, 2H), 6.84–6.81 (m, 2H), 6.16 (dd, *J* = 20.3, 14.7 Hz, 1H), 5.98 (dd, *J* = 14.8, 3.8 Hz, 1H), 5.71 (dd, *J* = 20.2, 3.9 Hz, 1H), 3.88 (t, *J* = 6.8 Hz, 2H), 1.82–1.76 (m, 2H), 0.70–0.67 (m, 2H), 0.11 (s, 6H) ppm.

^{13}C NMR (126 MHz, $CDCl_3$) δ 157.1 (d, *J* = 237.8 Hz), 155.2, 138.6, 131.9, 115.7 (d, *J* = 23.2 Hz), 115.4 (d, *J* = 7.8 Hz), 71.2, 23.8, 11.3, –3.5 ppm.

^{19}F NMR (471 MHz, $CDCl_3$) δ –124.4 ppm.

IR (ATR): $\tilde{\nu}$/cm^{-1} = 3049, 2938, 2874, 2246, 2114, 1951, 1854, 1598, 1503, 1404, 1289, 1246, 1207, 1095, 1008, 951, 893, 824, 760, 705.

HRMS (APCI) for $C_{11}H_{16}FOSi^+$ $[M–C_2H_3]^+$: calculated 211.0949, found 211.0952.

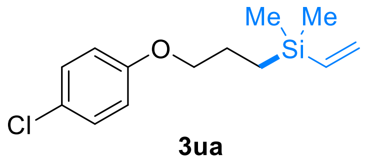

3ua
$C_{13}H_{19}ClOSi$
M = 254.83 g/mol

(3-(4-Chlorophenoxy)propyl)dimethyl(vinyl)silane (3ua): Prepared from **1u** (37 mg, 0.20 mmol) and **2a** (72 mg, 0.60 mmol) according to **GP**. Purification by flash column chromatography on silica gel using *n*-pentane:ethyl acetate = 99:1 afforded **3ua** as a colorless oil (38 mg, 75% yield).

R_f = 0.40 (*n*-pentane).

^{1}H NMR (500 MHz, $CDCl_3$) δ 7.22 (d, *J* = 9.0 Hz, 2H), 6.81 (d, *J* = 9.0 Hz, 2H), 6.15 (dd, *J* = 20.3, 14.7 Hz, 1H), 5.97 (dd, *J* = 14.7, 3.8 Hz, 1H), 5.70 (dd, *J* = 20.3, 3.8 Hz, 1H), 3.88 (t, *J* = 6.8 Hz, 2H), 1.82–1.76 (m, 2H), 0.69–0.66 (m, 2H), 0.10 (s, 6H) ppm.

^{13}C NMR (126 MHz, $CDCl_3$) δ 157.7, 138.6, 131.9, 129.2, 129.2, 125.3, 115.7, 115.7, 70.8, 23.7, 11.3, –3.5 ppm.

IR (ATR): ṽ/cm^{-1} = 3046, 2926, 2306, 2088, 1934, 1867, 1741, 1959, 1490, 1404, 1283, 1240, 1168, 1091, 1006, 950, 891, 820, 768, 707, 661.

HRMS (APCI) for $C_{11}H_{16}ClOSi^{+}$ $[M–C_2H_3]^{+}$: calculated 227.0653, found 227.0654.

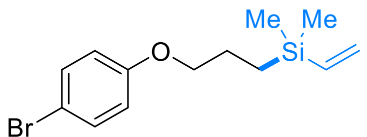

3va

$C_{13}H_{19}BrOSi$

M = 299.28 g/mol

(3-(4-Bromophenoxy)propyl)dimethyl(vinyl)silane (3va): Prepared from **1v** (46 mg, 0.20 mmol) and **2a** (72 mg, 0.60 mmol) according to **GP**. Purification by flash column chromatography on silica gel using *n*-pentane:ethyl acetate = 99:1 afforded **3va** as a colorless oil (41 mg, 68% yield).

R_f = 0.40 (*n*-pentane).

^{1}H NMR (500 MHz, $CDCl_3$) δ 7.36 (d, *J* = 8.9 Hz, 2H), 6.77 (d, *J* = 8.9 Hz, 2H), 6.16 (dd, *J* = 20.2, 14.7 Hz, 1H), 5.98(dd, *J* = 14.7, 3.8 Hz, 1H), 5.71 (dd, *J* = 20.4, 3.8 Hz, 1H), 3.88(t, *J* = 6.8 Hz, 2H), 1.82–1.76 (m, 2H), 0.69–0.66 (m, 2H), 0.10 (s, 6H) ppm.

^{13}C NMR (126 MHz, $CDCl_3$) δ 158.2, 138.6, 132.2, 131.9, 116.3, 112.6, 70.8, 23.7, 11.3, –3.5 ppm.

IR (ATR): ṽ/cm^{-1} = 3046, 2937, 2534, 2300, 2078, 1869, 1737, 1590, 1487, 1403, 1283, 1238, 1169, 1100, 1071, 1002, 950, 891, 817, 767, 706.

HRMS (APCI) for $C_{11}H_{16}BrOSi^{+}$ $[M–C_2H_3]^{+}$: calculated 271.0148, found 271.0147.

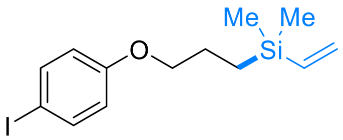

3wa
$C_{13}H_{19}IOSi$
M = 346.28 g/mol

(3-(4-Iodophenoxy)propyl)dimethyl(vinyl)silane (3wa): Prepared from **1w** (56 mg, 0.20 mmol) and **2a** (72 mg, 0.60 mmol) according to **GP**. Purification by flash column chromatography on silica gel using *n*-pentane:ethyl acetate = 99:1 afforded **3wa** as a colorless oil (33 mg, 47% yield).

R_f = 0.40 (*n*-pentane).

^{1}H NMR (500 MHz, $CDCl_3$) δ 7.23 (d, *J* = 24.7 Hz, 2H), 6.86 (d, *J* = 23.6 Hz, 2H), 6.13 (dd, *J* = 20.2, 14.7 Hz, 1H), 5.94 (dd, *J* = 14.6, 3.8 Hz, 1H), 5.67 (dd, *J* = 20.4, 3.8 Hz, 1H), 3.89 (t, *J* = 6.9 Hz, 2H), 1.77 (dt, *J* = 14.1, 7.1 Hz, 2H), 0.68–0.64 (m, 2H), 0.08 (s, 6H) ppm.

^{13}C NMR (126 MHz, $CDCl_3$) δ 138.7, 131.8, 129.4, 127.3, 120.5, 114.5, 70.4, 23.8, 11.4, –3.5 ppm.

IR (ATR): ṽ/cm^{-1} = 3045, 2935, 2187, 1602, 1509, 1404, 1243, 1173, 1112, 1051, 1009, 951, 893, 833, 771, 690.

HRMS (APCI) for $C_{13}H_{19}OSi^+$ [M–I]$^+$: calculated 219.1200, found 219.1200.

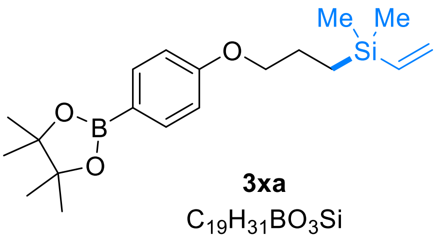

3xa
$C_{19}H_{31}BO_3Si$
M = 346.35 g/mol

Dimethyl(3-(4-(4,4,5,5-tetramethyl-1,3,2-dioxaborolan-2-yl)phenoxy)propyl)(vinyl)silane (3xa): Prepared from **1x** (56 mg, 0.20 mmol) and **2a** (72 mg, 0.60 mmol) according to **GP**. Purification by flash column chromatography on silica gel using *n*-pentane:ethyl acetate = 99:1 afforded **3xa** as a colorless oil (50 mg, 72% yield).

R_f = 0.40 (*n*-pentane).

^{1}H NMR (500 MHz, $CDCl_3$) δ 7.74 (d, *J* = 8.3 Hz, 2H), 6.88 (d, *J* = 8.6 Hz, 2H), 6.15 (dd, *J* = 20.3, 14.7 Hz, 1H), 5.97 (dd, *J* = 14.6, 3.8 Hz, 1H), 5.70 (dd, *J* = 20.3, 3.8 Hz, 1H), 3.94 (t, *J* = 6.6 Hz, 2H), 1.83–1.77 (m, 2H), 1.33 (s, 12H), 0.70–0.67 (m, 2H), 0.10 (s, 6H) ppm.

^{13}C NMR (126 MHz, $CDCl_3$) δ 161.7, 138.6, 136.5, 131.8, 113.8, 83.5, 70.3, 24.8, 23.7, 11.3, –3.5 ppm.

IR (ATR): ṽ/cm^{-1} = 3205, 2976, 2928, 2256, 2121, 2098, 1950, 1908, 1738, 1603, 1515, 1467, 1357, 1316, 1242, 1141, 1090, 1048, 1009, 960, 891, 831, 768.

HRMS (APCI) for $C_{17}H_{28}BO_3Si^+$ $[M–C_2H_3]^+$: calculated 319.1895, found 319.1896.

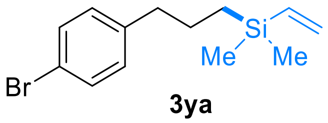

3ya

$C_{13}H_{19}BrSi$

M = 283.28 g/mol

(3-(4-Bromophenyl)propyl)dimethyl(vinyl)silane (3ya): Prepared from **1y** (43 mg, 0.20 mmol) and **2a** (72 mg, 0.60 mmol) according to **GP**. Purification by flash column chromatography on silica gel using *n*-pentane afforded **3ya** as a colorless oil (42 mg, 73% yield).

R_f = 0.95 (*n*-pentane).

^{1}H NMR (500 MHz, $CDCl_3$) δ 7.39 (d, *J* = 8.4 Hz, 2H), 7.04 (d, *J* = 8.4 Hz, 2H), 6.12 (dd, *J* = 20.3, 14.7 Hz, 1H), 5.95 (dd, *J* = 14.7, 3.8 Hz, 1H), 5.66 (dd, *J* = 20.3, 3.9 Hz, 1H), 2.58 (t, *J* = 7.6 Hz, 2H), 1.63–1.57 (m, 2H), 0.61–0.57 (m, 2H), 0.06 (s, 6H) ppm.

^{13}C NMR (126 MHz, $CDCl_3$) δ 141.5, 138.9, 131.6, 131.2, 130.2, 119.3, 39.1, 25.8, 15.1, –3.5 ppm.

IR (ATR): ṽ/cm^{-1} = 3045, 2924, 2856, 2665, 2291, 2092, 2000, 1891, 1591, 1486, 1402, 1342, 1247, 1169, 1071, 1009, 950, 831, 781, 701.

HRMS (APCI) for $C_{11}H_{16}BrSi^+$ $[M–C_2H_3]^+$: calculated 255.0199, found 255.0198.

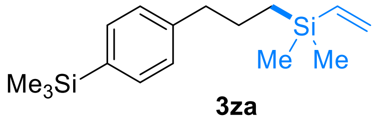

3za
$C_{16}H_{28}Si_2$
M = 276.57 g/mol

(4-(3-(Dimethyl(vinyl)silyl)propyl)phenyl)trimethylsilane (3za): Prepared from **1z** (42 mg, 0.20 mmol) and **2a** (72 mg, 0.60 mmol) according to **GP**. Purification by flash column chromatography on silica gel using *n*-pentane afforded **3za** as a colorless oil (47 m**g,** 84% yield).

R_f = 0.95 (*n*-pentane).

^{1}H NMR (500 MHz, $CDCl_3$) δ 7.48 (d, *J* = 7.8 Hz, 2H), 7.21 (d, *J* = 7.5 Hz, 2H), 6.17 (dd, *J* = 20.3, 14.7 Hz, 1H), 5.98 (dd, *J* = 14.7, 3.9 Hz, 1H), 5.70 (dd, *J* = 20.3, 3.9 Hz, 1H), 2.66 (t, *J* = 7.8 Hz, 2H), 1.71–1.65 (m, 2H), 0.70–0.66 (m, 2H), 0.30 (s, 9H), 0.10 (s, 6H) ppm.

^{13}C NMR (126 MHz, $CDCl_3$) δ 143.3, 139.1, 137.1, 133.3, 131.5, 128.0, 125.2, 39.9, 25.9, 15.4, –1.0, –3.4 ppm.

IR (ATR): ṽ/cm^{-1} = 3444, 3045, 2922, 2854, 2340, 2125, 1959, 1718, 1601, 1451, 1404, 1268, 1174, 1110, 1026, 949, 834, 779.

HRMS (APCI) for $C_{14}H_{25}Si_2^+$ $[M–C_2H_3]^+$: calculated 249.1489, found 249.1491.

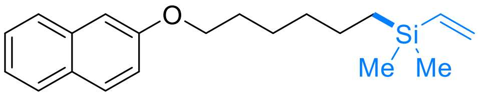

5aa
$C_{20}H_{28}OSi$
M = 312.53 g/mol

Dimethyl(6-(naphthalen-2-yloxy)hexyl)(vinyl)silane (5aa): Prepared from **4a** (49 mg, 0.20 mmol) and **2a** (72 mg, 0.60 mmol) according to **GP**. Purification by flash column chromatography on silica gel using *n*-pentane:ethyl acetate = 99:1 afforded **5aa** as a colorless oil (56 mg, 89% yield).

R_f = 0.45 (*n*-pentane).

^{1}H NMR (500 MHz, $CDCl_3$) δ 7.78–7.72 (m, 3H), 7.44 (t, *J* = 7.5 Hz, 1H), 7.33 (t, *J* = 7.5 Hz, 1H), 7.17–7.14(m, 2H), 6.16 (dd, *J* = 20.4, 14.7 Hz, 1H), 5.96 (dd, *J* = 14.7, 3.9 Hz, 1H), 5.68

(dd, *J* = 20.3, 3.9 Hz, 1H), 4.08 (t, *J* = 6.6 Hz, 2H), 1.88–1.82 (m, 2H), 1.55–1.49 (m, 2H), 1.46–1.36 (m, 4H), 0.62–0.58 (m, 2H), 0.07 (s, 6H) ppm.

^{13}C NMR (126 MHz, $CDCl_3$) δ 157.1, 139.3, 134.6, 131.3, 129.3, 128.9, 127.6, 126.7, 126.2, 123.4, 119.0, 106.6, 68.0, 33.2, 29.2, 25.8, 23.7, 15.3, –3.4 ppm.

IR (ATR): ṽ/cm^{-1} = 3048, 2929, 2853, 2271, 2080, 1896, 1628, 1599, 1510, 1463, 1389, 1355, 1255, 1215, 1179, 1119, 1007, 948, 831, 743, 704.

HRMS (APCI) for $C_{20}H_{28}OSi^+$ [M]$^+$: calculated 312.1904, found 312.1905.

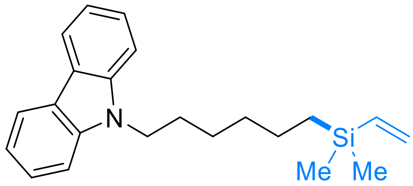

5ba
$C_{22}H_{29}NSi$
M = 335.57 g/mol

9-(6-(Dimethyl(vinyl)silyl)hexyl)-9H-carbazole (5ba): Prepared from **4b** (54 mg, 0.20 mmol) and **2a** (72 mg, 0.60 mmol) according to **GP**. Purification by flash column chromatography on silica gel using *n*-pentane:ethyl acetate =m99:1 afforded **5ba** as a yellow oil (56 mg, 83% yield).

R_f = 0.35 (*n*-pentane).

^{1}H NMR (500 MHz, $CDCl_3$) δ 8.12 (d, *J* = 7.7 Hz, 2H), 7.48 (t, *J* = 7.6 Hz, 2H), 7.41 (d, *J* = 8.2 Hz, 2H), 7.23 (d, *J* = 7.8 Hz, 2H), 6.13 (dd, *J* = 20.3, 14.7 Hz, 1H), 5.95 (dd, *J* = 14.6, 3.9 Hz, 1H), 5.67 (dd, *J* = 20.3, 3.9 Hz, 1H), 4.30 (t, *J* = 7.3 Hz, 2H), 1.89–1.86 (m, 2H), 1.39 (t, *J* = 3.9 Hz, 4H), 1.30 (dd, *J* = 12.3, 4.5 Hz, 2H), 0.56–0.53 (m, 2H), 0.05 (s, 6H) ppm.

^{13}C NMR (126 MHz, $CDCl_3$) δ 140.4, 139.2, 131.4, 125.5, 122.8, 120.3, 118.7, 108.6, 43.0, 33.2, 28.8, 26.9, 23.6, 15.3, –3.4 ppm.

IR (ATR): ṽ/cm^{-1} = 3046, 2920, 2851, 2647, 2096, 1880, 1595, 1483, 1451, 1403, 1324, 1244, 1151, 1120, 1066, 1004, 948, 833, 745, 719.

HRMS (APCI) for $C_{22}H_{30}NSi^+$ [M+H]$^+$: calculated 336.2142, found 336.2142.

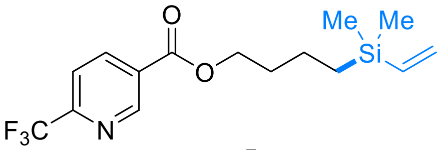

5ca
$C_{15}H_{20}F_3NO_2Si$
M = 331.41 g/mol

4-(Dimethyl(vinyl)silyl)butyl 6-(trifluoromethyl)nicotinate (5ca): Prepared from **4c** (53 mg, 0.20 mmol) and **2a** (72 mg, 0.60 mmol) according to **GP**. Purification by flash column chromatography on silica gel using *n*-pentane:ethyl acetate = 98:2 afforded **5ca** as a yellow oil (40 mg, 60% yield).

R_f = 0.60 (*n*-pentane:ethyl acetate = 90:10).

^{1}H NMR (500 MHz, $CDCl_3$) δ 9.29 (s, 1H), 8.47 (d, *J* = 8.3 Hz, 1H), 7.78 (d, *J* = 8.2 Hz, 1H), 6.12 (dd, *J* = 20.2, 14.7 Hz, 1H), 5.94 (dd, *J* = 14.7, 3.9 Hz, 1H), 5.66 (dd, *J* = 20.2, 3.9 Hz, 1H), 4.39 (t, *J* = 6.6 Hz, 2H), 1.85–1.78 (m, 2H), 1.50–1.42 (m, 2H), 0.65–0.61 (m, 2H), 0.06 (s, 6H) ppm.

^{13}C NMR (126 MHz, $CDCl_3$) δ 164.0, 151.2 (q, *J* = 35.2 Hz), 151.0, 138.7, 138.7, 131.8, 128.8, 121.1 (q, *J* = 274.6 Hz) 120.2 (q, *J* = 2.9 Hz), 65.8, 32.1, 20.3, 15.0, –3.5 ppm.

^{19}F NMR (471 MHz, $CDCl_3$) δ –68.3 ppm.

IR (ATR): $\tilde{v}$/cm^{-1} = 3450, 2955, 2352, 2210, 2117, 1727, 1600, 1467, 1391, 1332, 1281, 1117, 1084, 1023, 950, 833, 789, 704.

HRMS (APCI) for $C_{15}H_{21}F_3NO_2Si^+$ $[M+H]^+$: calculated 332.1288, found 332.1291.

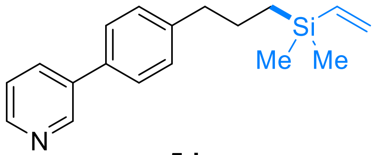

5da
$C_{18}H_{23}NSi$
M = 281.47 g/mol

3-(4-(3-(Dimethyl(vinyl)silyl)propyl)phenyl)pyridine (5da): Prepared from **4d** (43 mg, 0.20 mmol) and **2a** (72mg, 0.60 mmol) according to **GP**. Purification by flash column chromatography on silica gel using *n*-pentane:ethyl acetate = 90:10 afforded **5da** as a colorless oil (29 mg, 51% yield).

R_f = 0.30 (*n*-pentane:ethyl acetate = 4:1).

^{1}H NMR (500 MHz, $CDCl_3$) δ 8.85 (s, 1H), 8.56 (d, *J* = 2.9 Hz, 1H), 7.86 (d, *J* = 8.0 Hz, 1H), 7.50 (d, *J* = 8.2 Hz, 2H), 7.34 (dd, *J* = 7.9, 4.6 Hz, 1H), 7.28 (d, *J* = 8.2 Hz, 2H), 6.14 (dd, *J* = 20.3, 14.7 Hz, 1H), 5.95 (dd, *J* = 14.8, 3.7 Hz, 1H), 5.67 (dd, *J* = 20.3, 3.7 Hz, 1H), 2.68 (t, *J* = 7.7 Hz, 2H), 1.69–1.66 (m, 2H), 0.66–0.63 (m, 2H), -0.07 (s, 6H) ppm.

^{13}C NMR (126 MHz, $CDCl_3$) δ 148.2, 148.2, 142.8, 139.0, 136.6, 135.2, 134.2, 129.2, 127.0, 123.5, 39.4, 25.9, 15.3, –3.5 ppm.

IR (ATR): ṽ/cm^{-1} = 3027, 2922, 2854, 2653, 2319, 2083, 1899, 1724, 1590, 1515, 1472, 1399, 1341, 1246, 1183, 1126, 1003, 949, 832, 793, 708.

HRMS (APCI) for $C_{18}H_{24}NSi^{+}$ [M+H]$^{+}$: calculated 282.1673, found 282.1671.

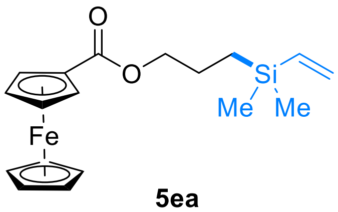

5ea

$C_{18}H_{24}FeO_2Si$

M = 356.32 g/mol

3-(Dimethyl(vinyl)silyl)propyl ferrocene carboxylate (5ea): Prepared from **4e** (58 mg, 0.20 mmol) and **2a** (72 mg, 0.60 mmol) according to **GP**. Purification by flash column chromatography on silica gel using *n*-pentane:ethyl acetate = 98:2 afforded **5ea** as a red brown oil (68mg, 95% yield).

R_f = 0.75 (*n*-pentane).

^{1}H NMR (500 MHz, $CDCl_3$) δ 6.16 (dd, *J* = 20.3, 14.8 Hz, 1H), 5.98 (dd, *J* = 14.7, 3.8 Hz, 1H), 5.71 (dd, *J* = 20.3, 3.9 Hz, 1H), 4.80 (t, *J* = 1.9 Hz, 2H), 4.38 (t, *J* = 1.9 Hz, 2H), 4.18 (d, *J* = 10.3 Hz, 7H), 1.71 (dd, *J* = 16.3, 7.8 Hz, 2H), 0.68–0.65 (m, 2H), -0.11(s, 6H) ppm.

^{13}C NMR (126 MHz, $CDCl_3$) δ 171.6, 138.5, 132.0,71.6, 71.1, 70.1, 69.7, 66.6, 23.5, 11.4, –3.5 ppm.

IR (ATR): ṽ/cm^{-1} = 3419, 3096, 3046, 2951, 2104, 1946, 1708, 1592, 1458, 1405, 1270, 1192, 1130, 1003, 951, 915, 816, 770, 705.

HRMS (APCI) for $C_{18}H_{24}FeO_2Si^{+}$ [M]$^{+}$: calculated 356.0884, found 356.0891.

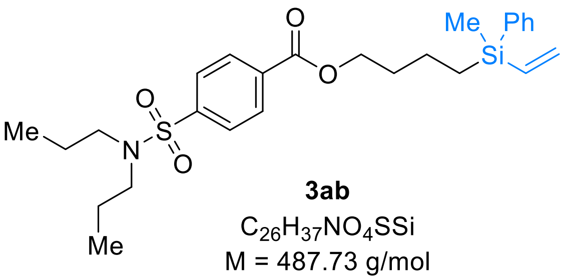

3ab
$C_{26}H_{37}NO_4SSi$
M = 487.73 g/mol

4-(Methyl(phenyl)(vinyl)silyl)butyl 4-(*N*,*N*-dipropylsulfamoyl)benzoate (3ab): Prepared from **1a** (72 mg, 0.20 mmol) and **2b** (110 mg, 0.60 mmol) according to **GP**. Purification by flash column chromatography on silica gel using *n*-pentane:ethyl acetate = 97:3 afforded **3ab** as a colorless oil (67 mg, 69% yield).

$\mathbf{R_f}$ = 0.40 (*n*-pentane:ethyl acetate = 4:1).

^{1}H NMR (500 MHz, $CDCl_3$) δ 7.89 (d, *J* = 8.2 Hz, 2H), 7.64 (d, *J* = 8.0 Hz, 2H), 7.29 (d, *J* = 5.0 Hz, 2H), 7.12 (d, *J* = 6.6 Hz, 3H), 6.07 (dd, *J* = 20.4, 14.7 Hz, 1H), 5.87 (dd, *J* = 14.7, 3.8 Hz, 1H), 5.55 (dd, *J* = 20.3, 3.8 Hz, 1H), 4.12 (t, *J* = 6.6 Hz, 2H), 2.88 (t, *J* = 7.7 Hz, 4H), 1.59 (p, *J* = 6.9 Hz, 2H), 1.35–1.27 (m, 6H), 0.65 (t, *J* = 7.4 Hz, 8H), 0.14 (s, 3H) ppm.

^{13}C NMR (126 MHz, $CDCl_3$) δ 165.2, 144.1, 137.1, 136.5, 134.0, 133.7, 133.6, 130.1, 129.1, 127.8, 126.9, 65.1, 49.9, 32.1, 21.9, 20.2, 13.7, 11.1, -5.1 ppm.

IR (ATR): $\tilde{\nu}$/cm^{-1} = 3531, 3445, 3047, 2962, 2933, 2874, 2694, 2299, 2115, 1880, 1720, 1596, 1463, 1398, 1342, 1270, 1157, 1107, 990, 863, 792, 734, 696.

HRMS (APCI) for $C_{26}H_{38}NO_4SSi^+$ $[M+H]^+$: calculated 488.2285, found 488.2285.

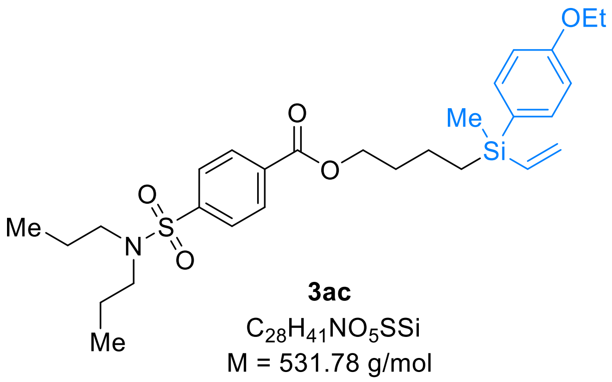

3ac
$C_{28}H_{41}NO_5SSi$
M = 531.78 g/mol

4-((4-Ethoxyphenyl)(methyl)(vinyl)silyl)butyl 4-(*N*,*N*-dipropylsulfamoyl)benzoate (3ac): Prepared from **1a** (72 mg, 0.20 mmol) and **2c** (136 mg, 0.60 mmol) according to **GP**. Purification by flash column chromatography on silica gel using *n*-pentane:ethyl acetate = 96:4 afforded **3ac** as a colorless oil (38 mg, 36% yield).

R_f = 0.30 (*n*-pentane:ethyl acetate = 90:10).

^{1}H NMR (500 MHz, $CDCl_3$) δ 7.91 (d, *J* = 8.1 Hz, 2H), 7.67 (d, *J* = 8.0 Hz, 2H), 7.22 (d, *J* = 7.9 Hz, 2H), 6.69 (d, *J* = 8.0 Hz, 2H), 6.08 (dd, *J* = 20.3, 14.7 Hz, 1H), 5.87 (dd, *J* = 14.9, 3.7 Hz, 1H), 5.55 (dd, *J* = 20.2, 3.8 Hz, 1H), 4.14 (t, *J* = 6.6 Hz, 2H), 3.84 (q, *J* = 7.0 Hz, 2H), 2.91 (t, *J* = 7.6 Hz, 4H), 1.61 (t, *J* = 7.2 Hz, 2H), 1.35 (dd, *J* = 15.1, 7.5 Hz, 6H), 1.22 (t, *J* = 7.0 Hz, 3H), 0.68 (t, *J* = 7.5 Hz, 8H), 0.13 (s, 3H) ppm.

^{13}C NMR (126 MHz, $CDCl_3$) δ 165.3, 159.9, 144.2, 137.0, 135.5, 133.8, 133.3, 130.1, 127.6, 127.0, 114.2, 65.2, 63.2, 49.9, 32.1, 21.9, 20.3, 14.8, 13.9, 11.1, -4.8 ppm.

IR (ATR): ṽ/cm^{-1} = 3531, 3445, 3047, 2962, 2933, 2874, 2694, 2299, 2115, 1880, 1720, 1596, 1463, 1398, 1342, 1270, 1157, 1107, 990, 863, 792, 734, 696.

HRMS (APCI) for $C_{28}H_{42}NO_5SSi^+$ $[M+H]^+$: calculated 532.2547, found 532.2548.

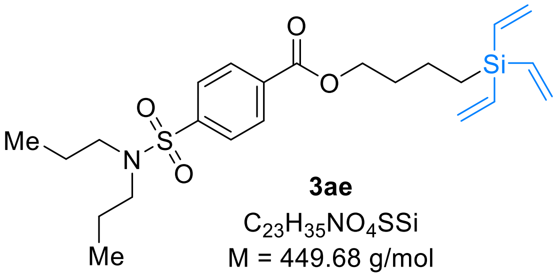

3ae
$C_{23}H_{35}NO_4SSi$
M = 449.68 g/mol

4-(Trivinylsilyl)butyl 4-(*N*,*N*-dipropylsulfamoyl)benzoate (3ae): Prepared from **1a** (72 mg, 0.20 mmol) and **2e** (87 mg, 0.60 mmol) according to **GP**. Purification by flash column chromatography on silica gel using *n*-pentane:ethyl acetate = 97:3 afforded **3ae** as a colorless oil (43 mg, 48% yield).

R_f = 0.40 (*n*-pentane:ethyl acetate = 90:10).

^{1}H NMR (500 MHz, $CDCl_3$) δ 8.07 (d, *J* = 8.0 Hz, 2H), 7.80 (d, *J* = 8.1 Hz, 2H), 6.12–5.97 (m, 6H), 5.71 (dd, *J* = 18.9, 5.0 Hz, 3H), 4.28 (t, *J* = 6.6 Hz, 2H), 3.06–3.00 (m, 4H), 1.79–1.70 (m, 2H), 1.48 (q, *J* = 7.5 Hz, 6H), 0.80 (t, *J* = 7.3 Hz, 8H) ppm.

^{13}C NMR (126 MHz, $CDCl_3$) δ 165.3, 144.2, 134.6, 134.4, 133.8, 130.1, 126.9, 65.2, 49.9, 49.9, 32.1, 21.9, 20.1, 12.3, 11.1 ppm.

IR (ATR): ṽ/cm^{-1} = 3447, 3049, 2929, 2873, 2682, 2298, 2086, 1900, 1722, 1593, 1461, 1400, 1344, 1269, 1158, 1105, 992, 954, 862.

HRMS (APCI) for $C_{23}H_{36}NO_4SSi^+$ $[M+H]^+$: calculated 450.2129, found 450.2126.

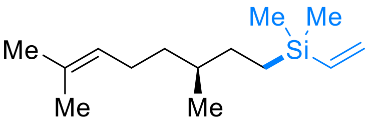

7aa
$C_{14}H_{28}Si$
M = 224.46 g/mol

(*S*)-(3,7-dimethyloct-6-en-1-yl)dimethyl(vinyl)silane (7aa): Prepared from (*S*)-(-)-β-Citronellol (31 mg, 0.20 mmol) and **2a** (72 mg, 0.60 mmol) according to **GP**. Purification by flash column chromatography on silica gel using *n*-pentane afforded **7aa** as a colorless oil (31 mg, 70% yield).

$\mathbf{R_f}$ = 0.95 (*n*-pentane).

^{1}H NMR (500 MHz, $CDCl_3$) δ 6.14(dd, *J* = 20.3, 14.6 Hz, 1H), 5.94 (dd, *J* = 14.6, 3.9 Hz, 1H), 5.66 (dd, *J* = 20.4, 3.9 Hz, 1H), 5.11 (dd, *J* = 8.5, 5.8 Hz, 1H), 2.01–1.88(m, 2H), 1.69 (s, 3H), 1.61 (s, 3H), 1.35–1.26 (m, 3H), 1.15–1.09 (m, 2H), 0.86 (d, *J* = 6.0 Hz, 3H), 0.60–0.46 (m, 2H), 0.05 (s, 6H) ppm.

^{13}C NMR (126 MHz, $CDCl_3$) δ 139.3, 131.3, 130.9, 125.1, 36.5, 35.1, 30.6, 25.7, 25.6, 19.1, 17.6, 12.1, –3.5 ppm.

IR (ATR): $\tilde{\nu}$/cm^{-1} = 2954, 2912, 2601, 2300, 2112, 1896, 1592, 1456, 1404, 1376, 1249, 1182, 1050, 1007, 949, 887, 834, 770, 705.

HRMS (APCI) for $C_{14}H_{27}Si^+$ $[M–H]^+$: calculated 223.1882, found 223.1879.

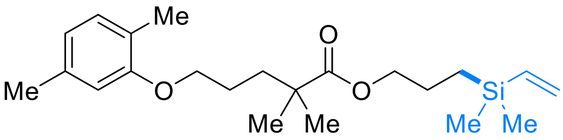

7ba
$C_{22}H_{36}O_3Si$
M = 376.61 g/mol

3-(Dimethyl(vinyl)silyl)propyl 5-(2,5-dimethylphenoxy)-2,2-dimethylpentanoate (7ba): Prepared from **6b** (62 mg, 0.20 mmol) and **2a** (72 mg, 0.60 mmol) according to **GP**. Purification by flash column chromatography on silica gel using *n*-pentane:ethyl acetate = 98:2 afforded **7ba** as a colorless oil (58 mg, 77% yield).

$\mathbf{R_f}$ = 0.65 (*n*-pentane:ethyl acetate = 90:10).

^{1}H NMR (500 MHz, $CDCl_3$) δ 7.01 (d, *J* = 7.5 Hz, 1H), 6.67 (d, *J* = 7.5 Hz, 1H), 6.62 (s, 1H), 6.13 (dd, *J* = 20.3, 14.7 Hz, 1H), 5.97 (dd, *J* = 14.7, 3.8 Hz, 1H), 5.69 (dd, *J* = 20.3, 3.8 Hz, 1H), 4.03 (t, *J* = 6.9 Hz, 2H), 3.93 (t, *J* = 5.6 Hz, 2H), 2.32 (s, 3H), 2.19 (s, 3H), 1.76–1.72 (m, 4H), 1.67–1.61 (m, 2H), 1.23 (s, 6H), 0.61–0.57 (m, 2H), 0.09 (s, 6H) ppm.

^{13}C NMR (126 MHz, $CDCl_3$) δ 177.8, 156.9, 138.5, 136.4, 131.9, 130.2, 123.5, 120.6, 111.9, 67.9, 66.9, 42.1, 37.1, 25.2, 23.2, 21.4, 15.7, 11.2, –3.6 ppm.

IR (ATR): ṽ/cm^{-1} = 3451, 2951, 2676, 2356, 2121, 2072, 1862, 1725, 1585, 1508, 1472, 1411, 1309, 1260, 1191, 1143, 1046, 1007, 950, 835, 801, 769, 707.

HRMS (APCI) for $C_{22}H_{37}O_3Si^+$ $[M+H]^+$: calculated 377.2506, found 377.2511.

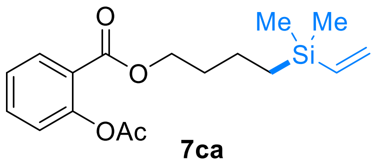

7ca

$C_{17}H_{24}O_4Si$

M = 320.46 g/mol

4-(Dimethyl(vinyl)silyl)butyl 2-acetoxybenzoate (7ca): Prepared from **6c** (51 mg, 0.20 mmol) and **2a** (72 mg, 0.60 mmol) according to **GP**. Purification by flash column chromatography on silica gel using *n*-pentane:ethyl acetate = 98:2 afforded **7ca** as a colorless oil (40 mg, 63% yield).

R_f = 0.75 (*n*-pentane:ethyl acetate = 90:10).

^{1}H NMR (500 MHz, $CDCl_3$) δ 8.01(dd, *J* = 7.9, 1.7 Hz, 1H), 7.57–7.53 (m, 1H), 7.32 (td, *J* = 7.6, 1.2 Hz, 1H), 7.10 (dd, *J* = 8.1, 1.2 Hz, 1H), 6.13 (dd, *J* = 20.2, 14.7 Hz, 1H), 5.95 (dd, *J* = 14.7, 3.9 Hz, 1H), 5.67 (dd, *J* = 20.2, 3.9 Hz, 1H), 4.27 (t, *J* = 6.7 Hz, 2H), 2.35 (s, 3H), 1.75 (p, *J* = 7.0 Hz, 2H), 1.46–1.41 (m, 2H), 0.64–0.59 (m, 2H), 0.07 (s, 6H) ppm.

^{13}C NMR (126 MHz, $CDCl_3$) δ 169.6, 164.5, 150.6, 138.8, 133.7, 131.7, 131.7, 125.9, 123.7, 123.5, 64.9, 32.2, 21.0, 20.3, 15.0, –3.5 ppm.

IR (ATR): ṽ/cm^{-1} = 3543, 3442, 2953, 2233, 2115, 1770, 1719, 1606, 1451, 1404, 1366, 1292, 1250, 1188, 1132, 1081, 1007, 951, 914, 832, 750, 702.

HRMS (APCI) for $C_{17}H_{23}O_4Si^+$ $[M–H]^+$: calculated 319.1366, found 319.1362.

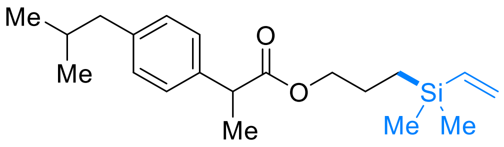

7da
$C_{20}H_{32}O_2Si$
M = 332.56 g/mol

3-(Dimethyl(vinyl)silyl)propyl 2-(4-isobutylphenyl)propanoat (7da): Prepared from **6d** (53 mg, 0.20 mmol) and **2a** (72 mg, 0.60 mmol) according to **GP**. Purification by flash column chromatography on silica gel using *n*-pentane:ethyl acetate = 98:2 afforded **7da** as a colorless oil (63 mg, 94% yield).

$\mathbf{R_f}$ = 0.40 (*n*-pentane).

^{1}**H NMR** (500 MHz, $CDCl_3$) δ 7.21 (d, *J* = 7.8 Hz, 2H), 7.09 (d, *J* = 7.8 Hz, 2H), 6.09 (dd, *J* = 20.2, 14.7 Hz, 1H), 5.95 (dd, *J* = 14.7, 3.9 Hz, 1H), 5.65 (dd, *J* = 20.3, 3.9 Hz, 1H), 4.03 (td, *J* = 6.8, 2.9 Hz, 2H), 3.69 (q, *J* = 7.2 Hz, 1H), 2.45 (d, *J* = 7.2 Hz, 2H), 1.88–1.82 (m, 1H), 1.61–1.55 (m, 2H), 1.50 (d, *J* = 7.3 Hz, 3H), 0.91 (d, *J* = 6.7 Hz, 6H), 0.50–0.47 (m, 2H), 0.04 (s, 6H) ppm.

^{13}**C NMR** (126 MHz, $CDCl_3$) δ 174.7, 140.4, 138.5, 137.9, 131.8, 129.2, 129.2, 127.1, 127.1, 67.0, 45.2, 45.0, 30.1, 23.1, 22.4, 22.3, 18.4, 11.1, –3.6 ppm.

IR (ATR): $\tilde{\nu}/cm^{-1}$ = 3459, 2953, 2392, 2307, 2092, 1899, 1733, 1592, 1511, 1461, 1329, 1247, 1201, 1160, 1069, 1007, 951, 834, 770, 706.

HRMS (APCI) for $C_{20}H_{33}O_2Si^+$ $[M+H]^+$: calculated 333.2244, found 333.2249.

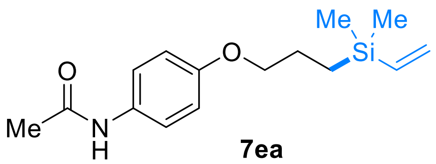

7ea
$C_{15}H_{23}NO_2Si$
M = 277.44 g/mol

***N*-(4-(3-(dimethyl(vinyl)silyl)propoxy)phenyl)acetamide (7ea):** Prepared from **6e** (42 mg, 0.20 mmol) and **2a** (72 mg, 0.60 mmol) according to **GP**. Purification by flash column chromatography on silica gel using *n*-pentane:ethyl acetate = 1:1 afforded **7ea** as a white solid (28 mg, 51% yield).

$\mathbf{R_f}$ = 0.40 (*n*-pentane:ethyl acetate = 1:3).

M.p.: 43–45 °C.

^{1}H NMR (500 MHz, $CDCl_3$) δ 7.39–7.35 (m, 2H), 6.82 (d, *J* = 8.6 Hz, 2H), 6.14 (dd, *J* = 20.3, 14.7 Hz, 1H), 5.96 (dd, *J* = 14.7, 3.8 Hz, 1H), 5.69 (dd, *J* = 20.3, 3.9 Hz, 1H), 3.88 (t, *J* = 6.8 Hz, 2H), 2.13 (s, 3H), 1.80–1.74 (m, 2H), 0.68–0.65 (m, 2H), 0.09 (s, 6H) ppm.

^{13}C NMR (126 MHz, $CDCl_3$) δ 168.3, 155.9, 138.6, 131.8, 130.8, 121.9, 114.7, 70.8, 24.2, 23.7, 11.3, –3.5 ppm.

IR (ATR): ṽ/cm^{-1} = 3296, 3096, 3132, 3047, 2936, 2867, 2322, 2090, 1890, 1654, 1606, 1508, 1405, 1365, 1239, 1168, 1108, 1048, 1010, 950, 893, 827, 765, 705.

HRMS (APCI) for $C_{15}H_{24}NO_2Si^+$ $[M+H]^+$: calculated 278.1571, found 278.1569.

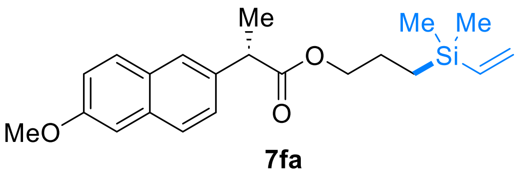

7fa
$C_{21}H_{28}O_3Si$
M = 356.54 g/mol

4-(Dimethyl(vinyl)silyl)butyl (*S*)-2-(6-methoxynaphthalen-2-yl)propanoate (7fa): Prepared from **6f** (58 mg, 0.20 mmol) and **2a** (72 mg, 0.60 mmol) according to **GP**. Purification by flash column chromatography on silica gel using *n*-pentane:ethyl acetate = 98:2 afforded **7fa** as a white solid (69 mg, 96% yield).

R_f = 0.50 (*n*-pentane:ethyl acetate = 90:10).

M.p.: 43–45 °C.

^{1}H NMR (500 MHz, $CDCl_3$) δ 7.72–7.69 (m, 3H), 7.43 (d, *J* = 8.4 Hz, 1H), 7.16–7.12 (m, 2H), 6.05 (dd, *J* = 20.1, 14.6 Hz, 1H), 5.92 (dd, *J* = 14.7, 4.0 Hz, 1H), 5.61 (dd, *J* = 20.2, 3.9 Hz, 1H), 4.05 (t, *J* = 6.8 Hz, 2H), 3.91 (s, 3H), 3.86 (t, *J* = 7.1 Hz, 1H), 1.59 (d, *J* = 7.2 Hz, 3H), 0.91–0.86 (m, 2H), 0.52–0.38 (m, 2H), 0.47–0.44 (m, 2H), 0.01 (s, 6H) ppm.

^{13}C NMR (126 MHz, $CDCl_3$) δ 174.6, 157.6, 138.4, 135.8, 133.6, 131.8, 129.2, 128.9, 127.0, 126.2, 125.9, 118.9, 105.6, 67.1, 55.2, 45.5, 23.0, 18.4, 11.0, –3.7 ppm.

IR (ATR): ṽ/cm^{-1} = 3746, 3449, 3048, 2953, 2157, 2018, 1972, 1728, 1605, 1459, 1391, 1324, 1262, 1155, 1069, 1032, 952, 834, 769, 704.

HRMS (APCI) for $C_{21}H_{28}O_3Si^+$ $[M]^+$: calculated 356.1802, found 356.1804.

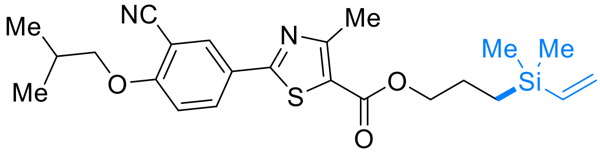

7ga
$C_{23}H_{30}N_2O_3SSi$
M = 442.65 g/mol

3-(Dimethyl(vinyl)silyl)propyl 2-(3-cyano-4-isobutoxyphenyl)-4-methylthiazole-5-carboxylate (7ga): Prepared from **6g** (75 mg, 0.20 mmol) and **2a** (72 mg, 0.60 mmol) according to **GP**. Purification by flash column chromatography on silica gel using *n*-pentane:ethyl acetate = 95:5 afforded **7ga** as a white solid (69 mg, 78% yield).

R$_f$ = 0.90 (*n*-pentane:ethyl acetate = 4:1).

M.p.: 83–85 °C.

^{1}H NMR (500 MHz, $CDCl_3$) δ 8.15 (s, 1H), 8.08 (d, *J* = 8.8 Hz, 1H), 7.00 (d, *J* = 8.9 Hz, 1H), 6.13 (dd, *J* = 20.3, 14.7 Hz, 1H), 5.97 (dd, *J* = 14.7, 3.8 Hz, 1H), 5.69 (dd, *J* = 20.3, 3.8 Hz, 1H), 4.24 (t, *J* = 6.9 Hz, 2H), 3.88 (d, *J* = 6.5 Hz, 2H), 2.75 (s, 3H), 2.18 (dt, *J* = 13.3, 6.6 Hz, 1H), 1.74 (dd, *J* = 16.6, 7.4 Hz, 2H), 1.08 (d, *J* = 6.8 Hz, 6H), 0.65–0.62 (m, 2H), 0.10 (s, 6H) ppm.

^{13}C NMR (126 MHz, $CDCl_3$) δ 167.0, 162.4, 162.0, 160.9, 138.3, 132.5, 132.0, 132.0, 126.0, 121.9, 115.3, 112.6, 102.9, 75.6, 67.8, 28.1, 23.2, 19.0, 19.0, 17.4, 11.3, –3.6 ppm.

IR (ATR): $\tilde{\nu}$/cm^{-1} = 3359, 3205, 2957, 2928, 2602, 2293, 2227, 2114, 2077, 1906, 1686, 1602, 1508, 1467, 1432, 1391, 1330, 1273, 1174, 1095, 1042, 1002, 950, 924, 826, 759, 724.

HRMS (APCI) for $C_{23}H_{31}N_2O_3SSi^+$ [M+H]$^+$: calculated 443.1819, found 443.1816.

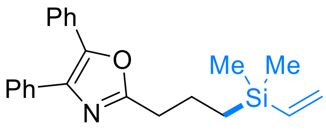

7ha
$C_{22}H_{25}NOSi$
M = 347.53 g/mol

2-(3-(Dimethyl(vinyl)silyl)propyl)-4,5-diphenyloxazol (7ha): Prepared from **6h** (56 mg, 0.20 mmol) and **2a** (72 mg, 0.60 mmol) according to **GP**. Purification by flash column

chromatography on silica gel using *n*-pentane:ethyl acetate = 98:2 afforded **7ha** as a colorless oil (67 mg, 96% yield).

R$_f$ = 0.50 (*n*-pentane:ethyl acetate = 90:10).

^{1}H NMR (500 MHz, $CDCl_3$) δ 7.66 (d, *J* = 6.9 Hz, 2H), 7.60 (d, *J* = 6.9 Hz, 2H), 7.37–7.31 (m, 6H), 6.16 (dd, *J* = 20.3, 14.7 Hz, 1H), 5.98 (dd, *J* = 14.7, 3.8 Hz, 1H), 5.71 (dd, *J* = 20.3, 3.9 Hz, 1H), 2.88 (t, *J* = 7.6 Hz, 2H), 1.92–1.86 (m, 2H), 0.75–0.72 (m, 2H), 0.11 (s, 6H) ppm.

^{13}C NMR (126 MHz, $CDCl_3$) δ 163.6, 145.0, 138.6, 134.9, 132.6, 131.8, 129.2, 128.6, 128.5, 128.2, 127.9, 127.9, 126.4, 126.4, 31.8, 22.0, 15.4, –3.5 ppm.

IR (ATR): ṽ/cm^{-1} = 3047, 2953, 2655, 2096, 1946, 1890, 1805, 1725, 1569, 1501, 1445, 1403, 1349, 1247, 1215, 1167, 1120, 1058, 1007, 959, 914, 832, 760, 690.

HRMS (APCI) for $C_{22}H_{26}NOSi^{+}$ $[M+H]^{+}$: calculated 348.1778, found 348.1781.

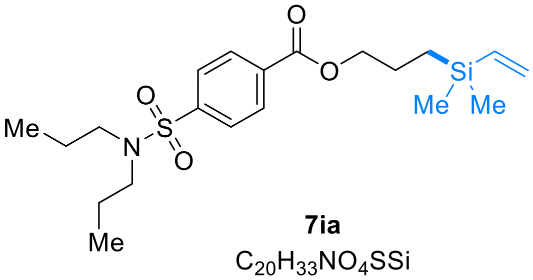

7ia
$C_{20}H_{33}NO_4SSi$
M = 411.63 g/mol

(Dimethyl(vinyl)silyl)propyl 4-(*N*,*N*-dipropylsulfamoyl)benzoate (7ia): Prepared from **6i** (69 mg, 0.20 mmol) and **2a** (72 mg, 0.60 mmol) according to **GP**. Purification by flash column chromatography on silica gel using *n*-pentane:ethyl acetate = 97:3 afforded **7ia** as a colorless oil (68 mg, 83% yield).

R$_f$ = 0.40 (*n*-pentane:ethyl acetate = 90:10).

^{1}H NMR (500 MHz, $CDCl_3$) δ 8.14 (d, *J* = 8.1 Hz, 2H), 7.86 (d, *J* = 8.1 Hz, 2H), 6.13 (dd, *J* = 20.2, 14.7 Hz, 1H), 5.97 (dd, *J* = 14.7, 3.8 Hz, 1H), 5.69 (dd, *J* = 20.1, 3.8 Hz, 1H), 4.29 (t, *J* = 7.0 Hz, 2H), 3.09 (t, *J* = 7.7 Hz, 4H), 1.80–1.74 (m, 2H), 1.53 (q, *J* = 7.6 Hz, 4H), 0.85 (t, *J* = 7.5 Hz, 6H), 0.66–0.63 (m, 2H), 0.09 (s, 6H) ppm.

^{13}C NMR (126 MHz, $CDCl_3$) δ 165.2, 144.1, 138.3, 133.7, 132.0, 130.1, 126.9, 68.0, 49.9, 49.8, 23.2, 21.9, 11.2, 11.1, –3.6 ppm.

IR (ATR): ṽ/cm^{-1} = 3443, 2959, 2875, 2299, 2116, 1720, 1598, 1463, 1398, 1344, 1269, 1158, 1105, 991, 835, 764, 739, 705.

HRMS (APCI) for $C_{20}H_{34}NO_4SSi^+$ [M+H]$^+$: calculated 412.1972, found 412.1971.

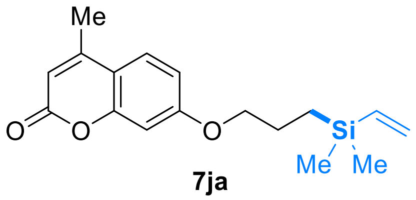

7ja
$C_{17}H_{22}O_3Si$
M = 302.45 g/mol

7-(3-(Dimethyl(vinyl)silyl)propoxy)-4-methyl-2H-chromen-2-one (7ja): Prepared from **6j** (47 mg, 0.20 mmol) and **2a** (72 mg, 0.60 mmol) according to **GP**. Purification by flash column chromatography on silica gel using *n*-pentane:ethyl acetate = 95:5 afforded **7ja** as a colorless oil (35 mg, 58% yield).

R$_f$ = 0.50 (*n*-pentane:ethyl acetate = 4:1).

M.p.: 60–62 °C.

^{1}H NMR (500 MHz, $CDCl_3$) δ 7.47 (d, *J* = 8.8 Hz, 1H), 6.83 (dd, *J* = 8.8, 2.5 Hz, 1H), 6.77 (d, *J* = 2.5 Hz, 1H), 6.19–6.10 (m, 2H), 5.97 (dd, *J* = 14.7, 3.9 Hz, 1H), 5.70 (dd, *J* = 20.2, 3.9 Hz, 1H), 3.96 (t, *J* = 6.8 Hz, 2H), 2.38 (d, *J* = 1.2 Hz, 3H), 1.86–1.78 (m, 2H), 0.71–0.66 (m, 2H), 0.10 (s, 6H) ppm.

^{13}C NMR (126 MHz, $CDCl_3$) δ 162.1, 161.3, 155.3, 152.5, 138.4, 132.0, 125.4, 113.4, 112.6, 111.7, 101.3, 71.0, 23.5, 18.6, 11.3, –3.5 ppm.

IR (ATR): ṽ/cm^{-1} = 3425, 3208, 3052, 2941, 2874, 2622, 2479, 2394, 2296,2208, 2083, 1988, 1890, 1720, 1603, 1509, 1467, 1386, 1344, 1257, 1196, 1138, 1067, 1011, 981, 947, 833, 804, 769, 705.

HRMS (APCI) for $C_{17}H_{23}O_3Si^+$ [M+H]$^+$: calculated 303.1411 found 303.1412.

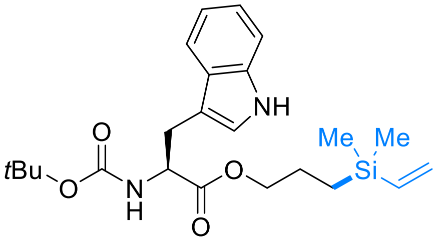

7ka

$C_{23}H_{34}N_2O_4Si$

M = 430.62 g/mol

3-(Dimethyl(vinyl)silyl)propyl (*tert*-butoxycarbonyl)-*D*-tryptophanate (7ka): Prepared from **6k** (73 mg, 0.20 mmol) and **2a** (72 mg, 0.60 mmol) according to **GP**. Purification by flash column chromatography on silica gel using *n*-pentane:ethyl acetate = 6:1 afforded **7ka** as a white solid (37 mg, 43% yield).

R_f = 0.40 (*n*-pentane:ethyl acetate = 4:1).

^{1}H NMR (500 MHz, $CDCl_3$) δ 8.18 (s, 1H), 7.57 (d, *J* = 7.9 Hz, 1H), 7.33 (d, *J* = 8.1 Hz, 1H), 7.18 (t, *J* = 7.6 Hz, 1H), 7.11 (t, *J* = 7.5 Hz, 1H), 6.99 (s, 1H), 6.11 (dd, *J* = 20.2, 14.7 Hz, 1H), 5.97 (dd, *J* = 14.7, 3.9 Hz, 1H), 5.67 (dd, *J* = 20.2, 3.9 Hz, 1H), 5.09 (d, *J* = 8.2 Hz, 1H), 4.63 (d, *J* = 7.2 Hz, 1H), 4.05–3.96 (m, 2H), 3.28 (s, 2H), 1.57–1.51 (m, 2H), 1.43 (s, 9H), 0.48–0.45 (m, 2H), 0.06 (s, 6H) ppm.

^{13}C NMR (126 MHz, $CDCl_3$) δ 172.4, 155.2, 138.4, 136.1, 132.0, 127.7, 122.7, 122.1, 119.5, 118.8, 111.1, 110.3, 79.7, 67.8, 54.2, 29.7, 28.3, 22.9, 11.0, –3.6 ppm.

IR (ATR): $\tilde{\nu}$/cm^{-1} = 3303, 3045, 2926, 2108, 1727, 1683, 1519, 1458, 1391, 1355, 1280, 1223, 1157, 1059, 1008, 949, 833, 765, 740, 683.

HRMS (APCI) for $C_{23}H_{35}N_2O_4Si^+$ $[M+H]^+$: calculated 431.2361, found 431.2355.

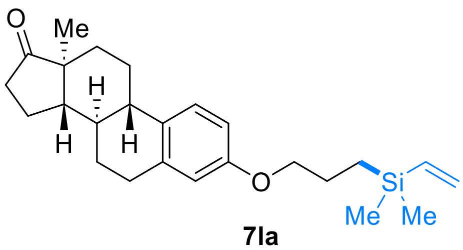

7la

$C_{25}H_{36}O_2Si$

M = 396.65 g/mol

(8*R*,9*S*,13*S*,14*S*)-3-(3-(Dimethyl(vinyl)silyl)propoxy)-13-methyl-6,7,8,9,11,12,13,14,15,16-decahydro-17*H*-cyclopenta[a]phenanthren-17-one (7la): Prepared from **6l** (66 mg, 0.20

mmol) and **2a** (72 mg, 0.60 mmol) according to **GP**. Purification by flash column chromatography on silica gel using *n*-pentane:ethyl acetate = 98:2 afforded **7la** as a white solid (73 mg, 92% yield).

R$_f$ = 0.55 (*n*-pentane:ethyl acetate = 90:10).

M.p.: 101–103 °C.

^{1}H NMR (500 MHz, $CDCl_3$) δ 7.19 (d, *J* = 8.6 Hz, 1H), 6.70 (d, *J* = 8.6 Hz, 1H), 6.64 (s, 1H), 6.16 (dd, *J* = 20.3, 14.7 Hz, 1H), 5.97 (dd, *J* = 14.7, 3.9 Hz, 1H), 5.70 (dd, *J* = 20.3, 3.9 Hz, 1H), 3.89 (t, *J* = 6.8 Hz, 2H), 2.89 (dd, *J* = 10.3, 6.3 Hz, 2H), 2.50 (dd, *J* = 19.0, 8.7 Hz, 1H), 2.39 (dd, *J* = 9.4, 5.5 Hz, 1H), 2.24 (q, *J* = 6.5 Hz, 1H), 2.15 (dt, *J* = 18.5, 8.8 Hz, 1H), 2.08–1.95 (m, 3H), 1.79 (dq, *J* = 11.9, 6.9 Hz, 2H), 1.66–1.56 (m, 2H), 1.54–1.41 (m, 4H), 0.91 (s, 3H), 0.70–0.66 (m, 2H), 0.10 (s, 6H) ppm.

^{13}C NMR (126 MHz, $CDCl_3$) δ 220.7, 157.1, 138.7, 137.6, 131.8, 126.2, 114.5, 112.1, 70.4, 50.4, 47.9, 43.9, 38.4, 35.8, 31.6, 29.6, 26.5, 25.9, 23.8, 21.5, 13.8, 11.3, –3.5 ppm.

IR (ATR): $\tilde{\nu}$/cm^{-1} = 3452, 3046, 2930, 2873, 2669, 2286, 2087, 1894, 1732, 1609, 1570, 1495, 1468, 1403, 1373, 1339, 1246, 1159, 1114, 1053, 1004, 947, 899, 832, 766, 706.

HRMS (APCI) for $C_{25}H_{35}O_2Si^+$ [M–H]$^+$: calculated 395.2406, found 395.2403.

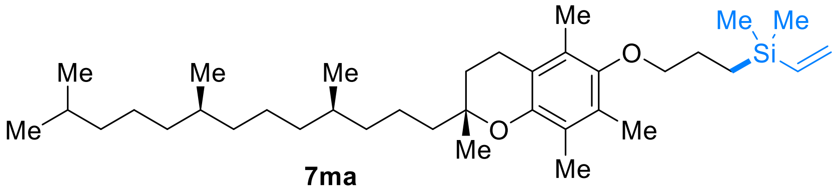

7ma
$C_{36}H_{64}O_2Si$
M = 556.99 g/mol

Dimethyl(3-(((*R*)-2,5,7,8-tetramethyl-2-((4*R*,8*R*)-4,8,12-trimethyltridecyl)chroman-6-yl)oxy)propyl)(vinyl)silane (7ma): Prepared from **6m** (98 mg, 0.20 mmol) and **2a** (72 mg, 0.60 mmol) according to **GP**. Purification by flash column chromatography on silica gel using *n*-pentane:ethyl acetate = 99:1 afforded **7ma** as a colorless oil (91 mg, 82% yield).

R$_f$ = 0.75 (*n*-pentane:ethyl acetate = 90:10).

^{1}H NMR (500 MHz, $CDCl_3$) δ 6.19 (dd, *J* = 20.4, 14.7 Hz, 1H), 6.00 (dd, *J* = 14.7, 3.9 Hz, 1H), 5.73 (dd, *J* = 20.3, 3.9 Hz, 1H), 3.62 (t, *J* = 6.9 Hz, 2H), 2.59 (t, *J* = 6.9 Hz, 2H), 2.19 (s, 3H), 2.15 (s, 3H), 2.11 (s, 3H), 1.86–1.76 (m, 4H), 1.59–1.53 (m, 4H), 1.48–1.38 (m, 4H), 1.37–1.28

(m, 9H), 1.19–1.15 (m, 3H), 1.12–1.08 (m, 4H), 0.88 (t, *J* = 6.0 Hz, 12H), 0.74–0.71 (m, 2H), 0.13 (s, 6H) ppm.

^{13}C NMR (126 MHz, $CDCl_3$) δ 148.4, 147.6, 138.8, 131.7, 127.8, 125.8, 122.7, 117.4, 75.7, 74.7, 40.1, 39.4, 37.5, 37.5, 37.4, 37.3, 32.8, 32.7, 31.3, 28.0, 24.8, 24.6, 24.4, 23.9, 22.7, 22.6, 21.0, 20.6, 19.7, 19.7, 12.8, 11.9, 11.8, 11.4, –3.5 ppm.

IR (ATR): ṽ/cm^{-1} = 2924, 2865, 2305, 2114, 2086, 1927, 1575, 1457, 1413, 1375, 1249, 1158, 1087, 1060, 1007, 949, 835, 769, 705.

HRMS (APCI) for $C_{36}H_{64}O_2Si^+$ [M]$^+$: calculated 556.4670, found 556.467.

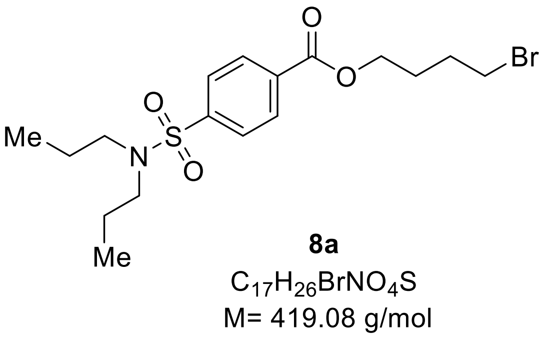

8a
$C_{17}H_{26}BrNO_4S$
M= 419.08 g/mol

4-Bromobutyl 4-(*N*,*N*- dipropylsulfamoyl)benzoate (8a): Prepared from **1a** (84 mg, 0.20 mmol) according to the first step of **GP**. Purification by flash column chromatography on silica gel using *n*-pentane:ethyl acetate = 97:3 afforded **8a** as a yellow oil (76 mg, 91% yield).

R_f = 0.75 (*n*-pentane:ethyl acetate = 95:5).

^{1}H NMR (500 MHz, $CDCl_3$) δ 8.12 (d, *J* = 8.4 Hz, 2H), 7.85 (d, *J* = 8.4 Hz, 2H), 4.37 (t, *J* = 6.2 Hz, 2H), 3.46 (t, *J* = 6.4 Hz, 2H), 3.09 – 3.06 (m, 4H), 2.03 – 2.00 (m, 2H), 1.95 – 1.92 (m, 2H), 1.54–1.50 (m, 4H), 0.84 (t, *J* = 7.4 Hz, 6H) ppm.

^{13}C NMR (126 MHz, $CDCl_3$) δ 165.1, 144.2, 133.4, 130.1, 126.9, 64.5, 49.8, 32.9, 29.2, 27.2, 21.8, 11.1 ppm.

IR (ATR): ṽ/cm^{-1} = 3445, 2964, 2874, 2683, 2292, 2124, 1915, 1719, 1599, 1463, 1398, 1340, 1269, 1156, 1106, 990, 861, 738, 708.

HRMS (APCI) for $C_{17}H_{27}BrNO_4S$ [M+H]$^+$: calculated 420.0839, found 420.0840.

6 NMR Spectra of Products

Figure S1. ^{1}H NMR (500 MHz, $CDCl_3$, 298 K) of ***n*-Bu₂phen**.

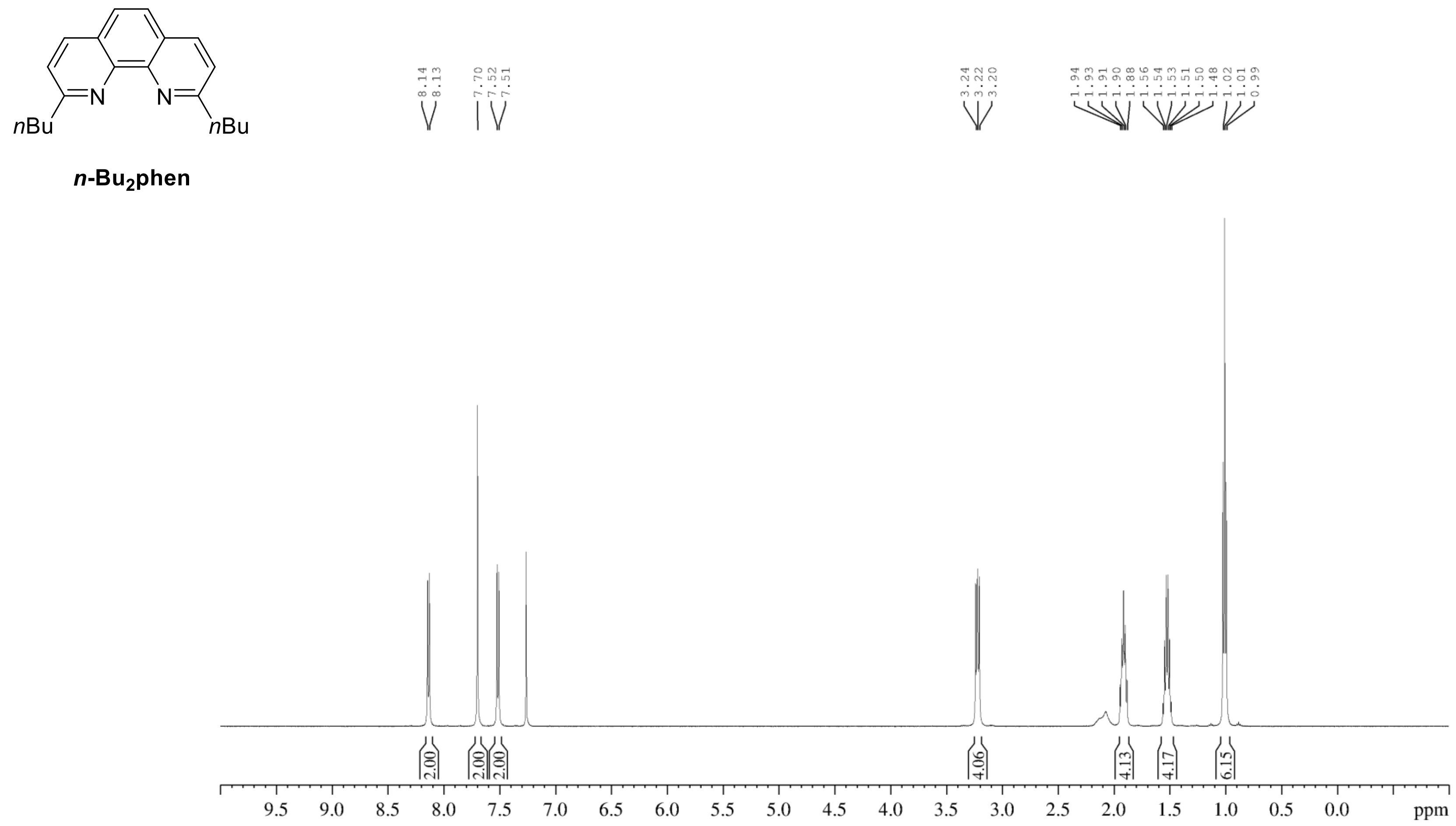

Figure S2. ^{13}C NMR (126 MHz, $CDCl_3$, 298 K) of ***n*-Bu₂phen**.

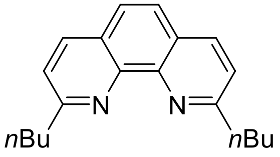

***n*-Bu$_2$phen**

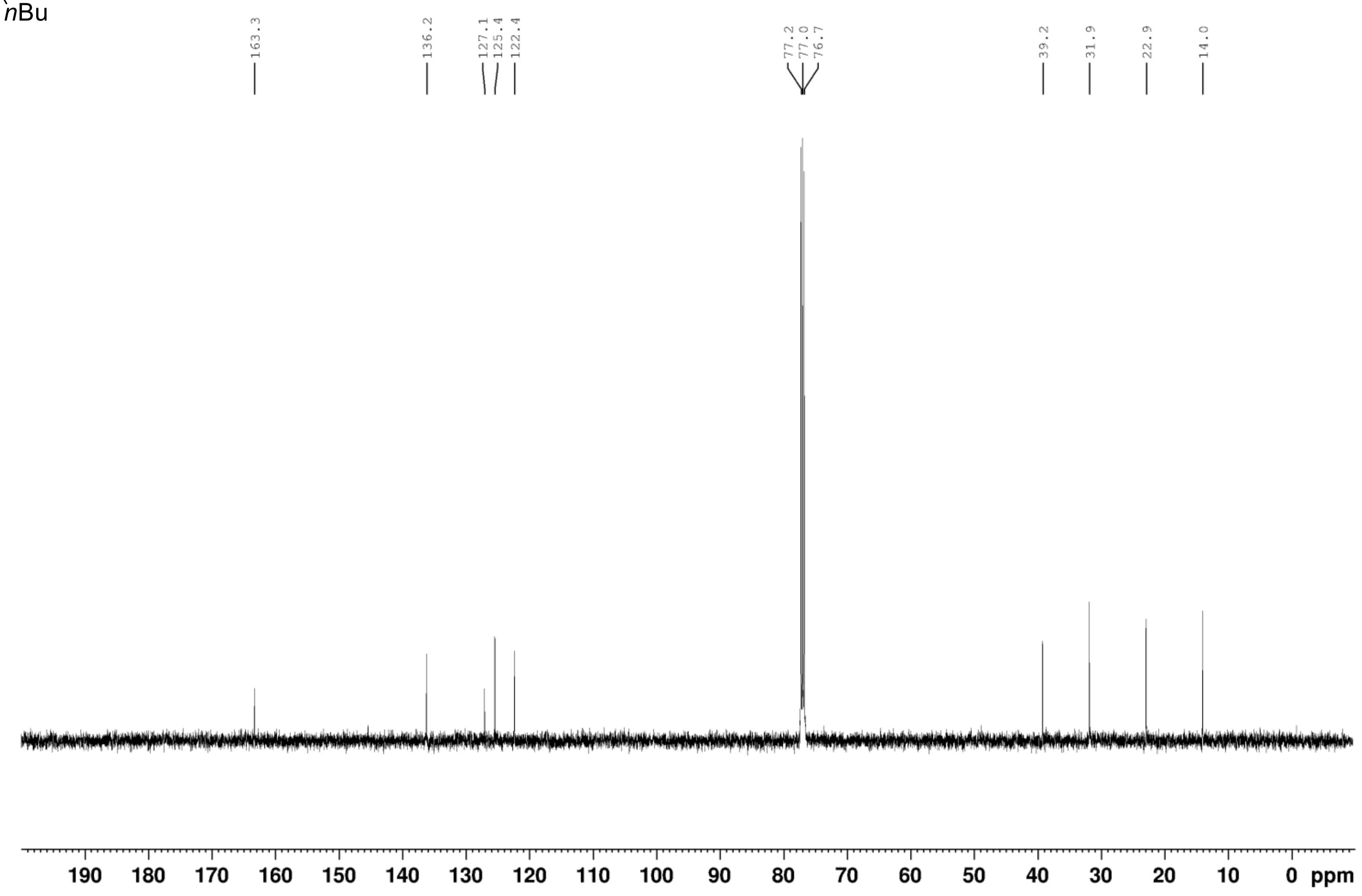

Figure S3. ^{1}H NMR (500 MHz, $CDCl_3$, 298 K) of **1a**.

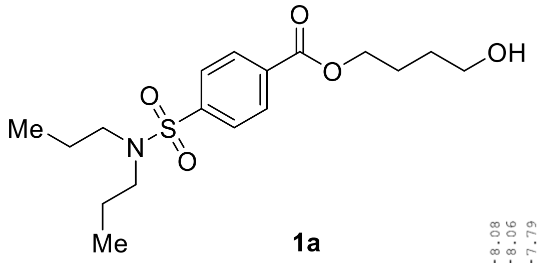

1a

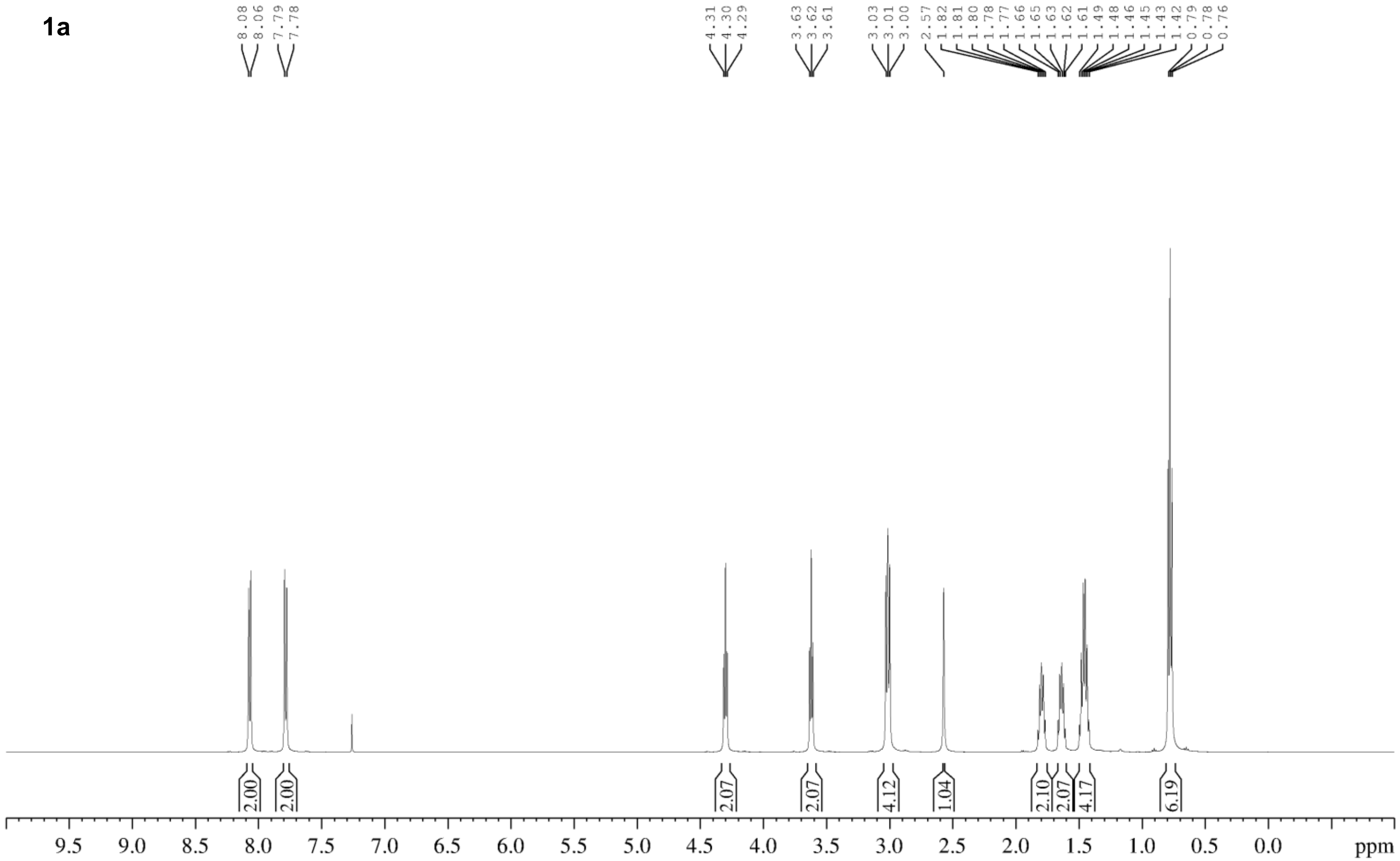

Figure S4. ^{13}C NMR (126 MHz, $CDCl_3$, 298 K) of **1a**.

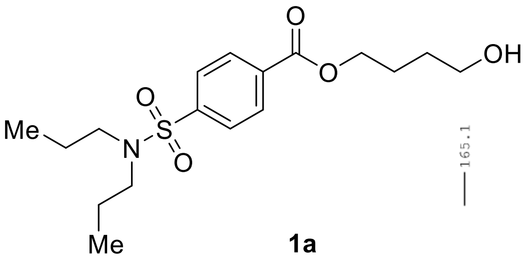

1a

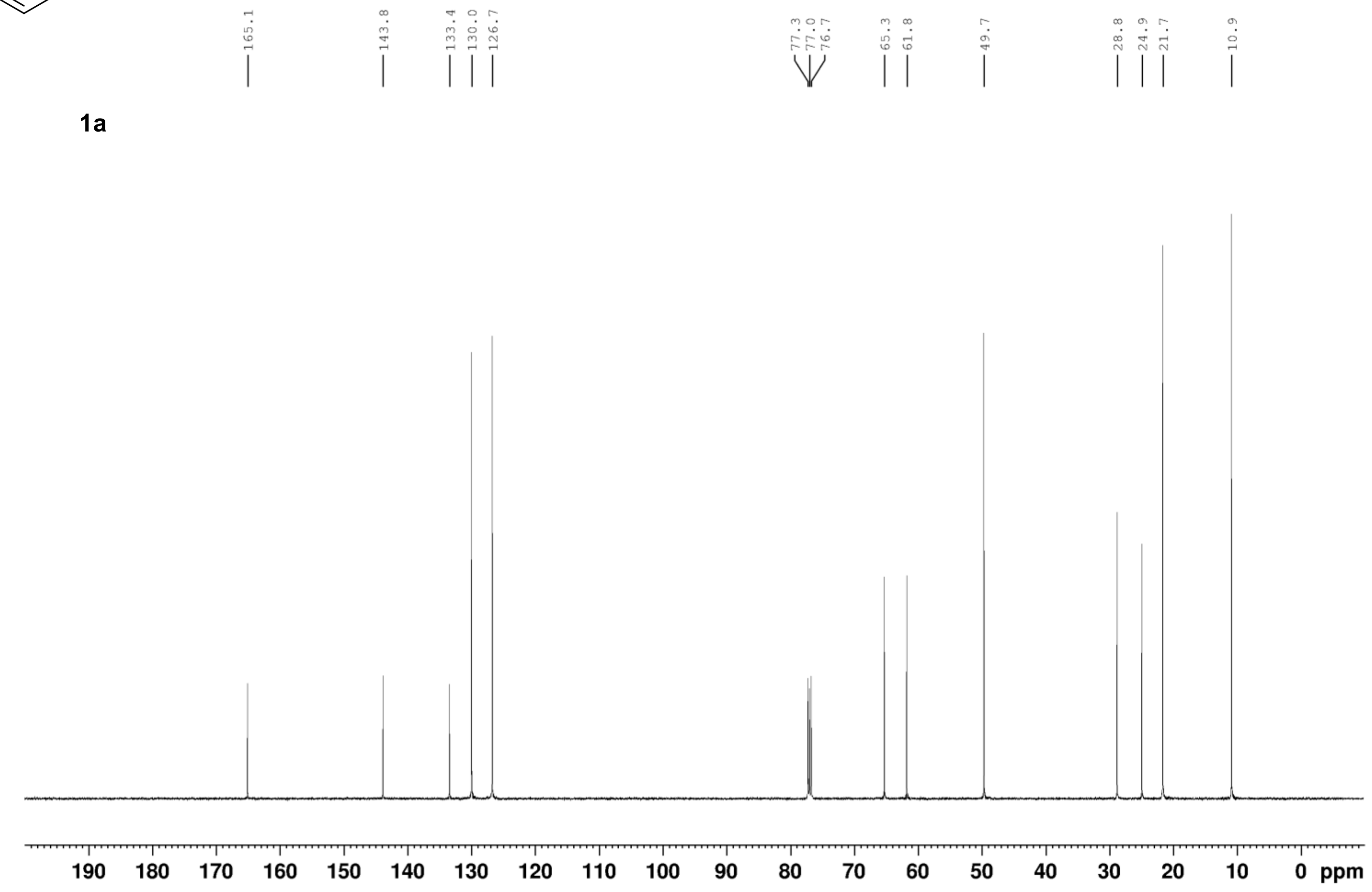

Figure S5. ^{1}H NMR (500 MHz, $CDCl_3$, 298 K) of **1c**.

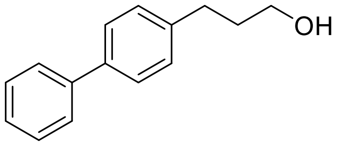

1c

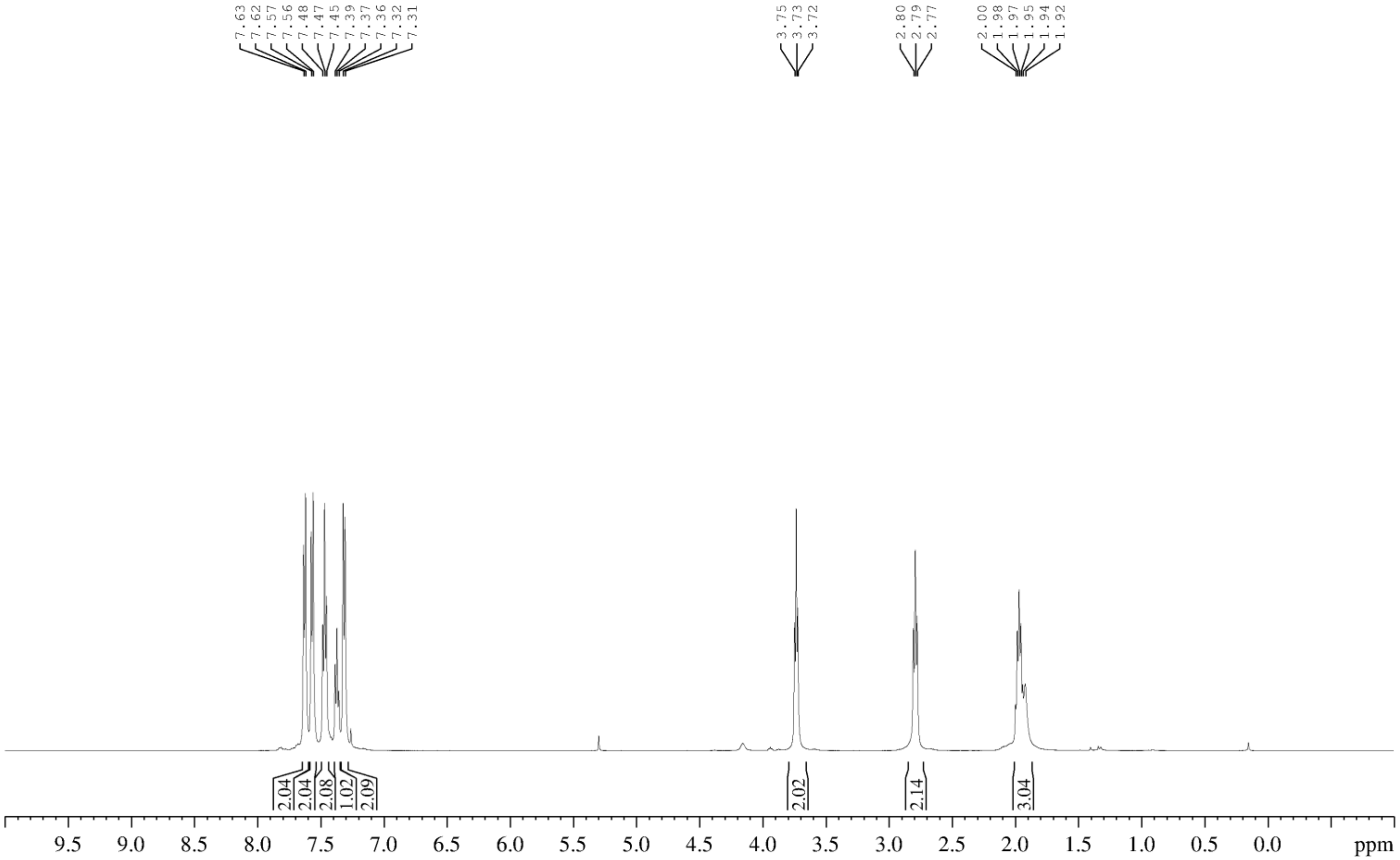

Figure S6. ^{13}C NMR (126 MHz, $CDCl_3$, 298 K) of **1c**.

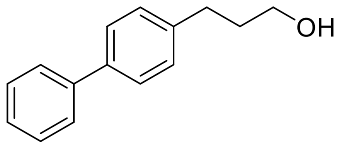

1c

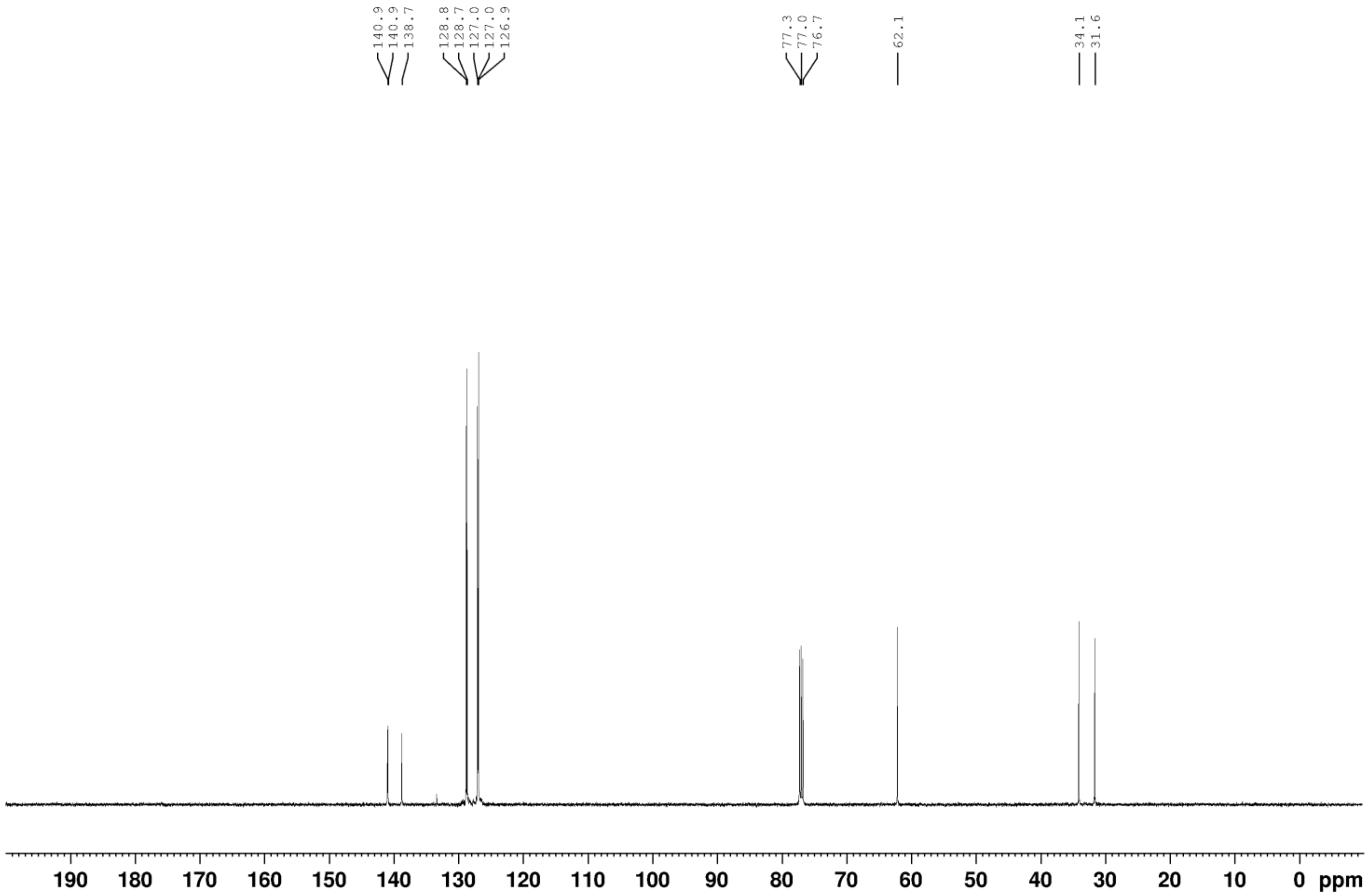

Figure S7. ^{1}H NMR (500 MHz, $CDCl_3$, 298 K) of **1h**.

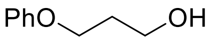

1h

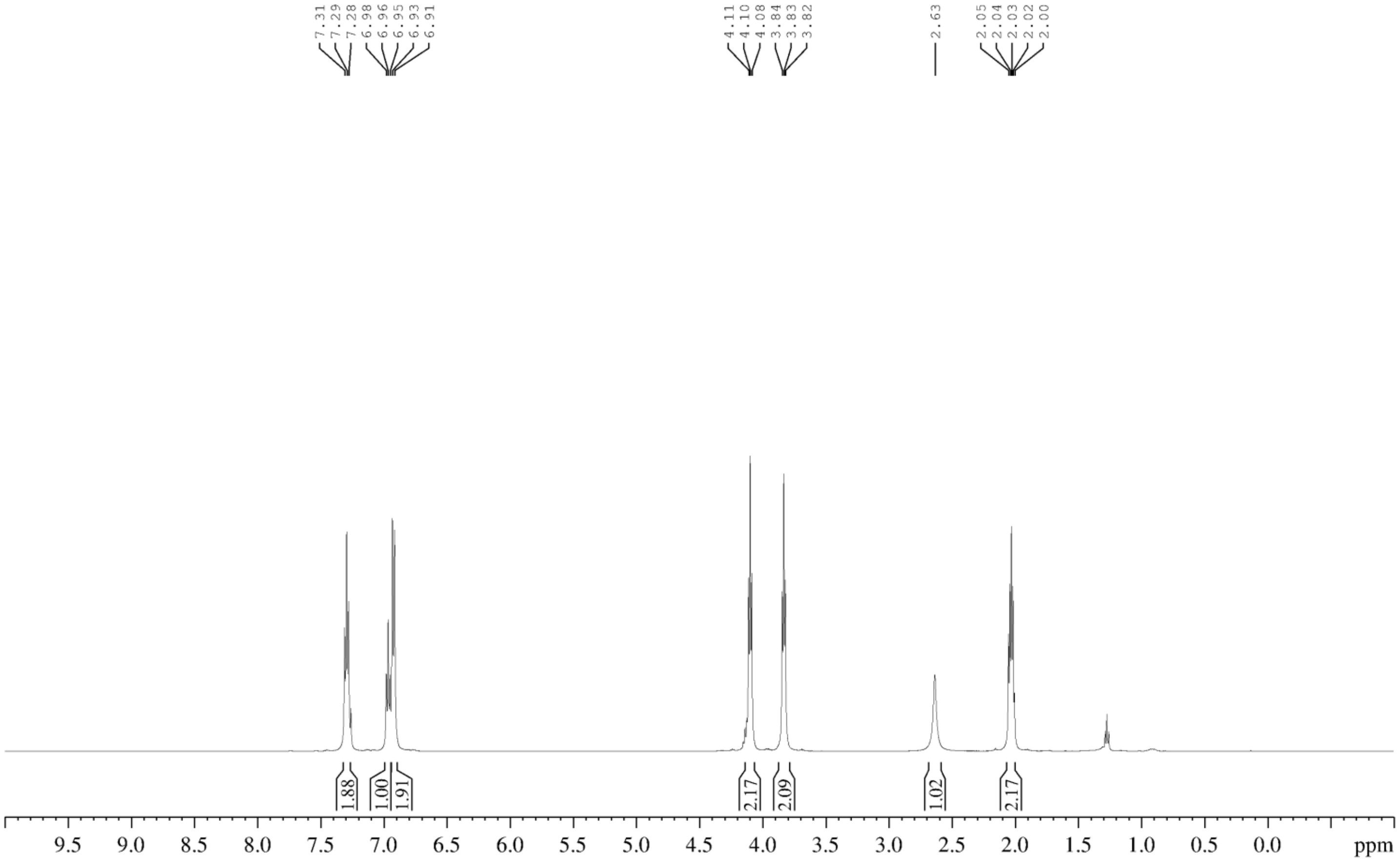

Figure S8. ^{13}C NMR (126 MHz, $CDCl_3$, 298 K) of **1h**.

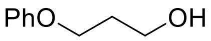

1h

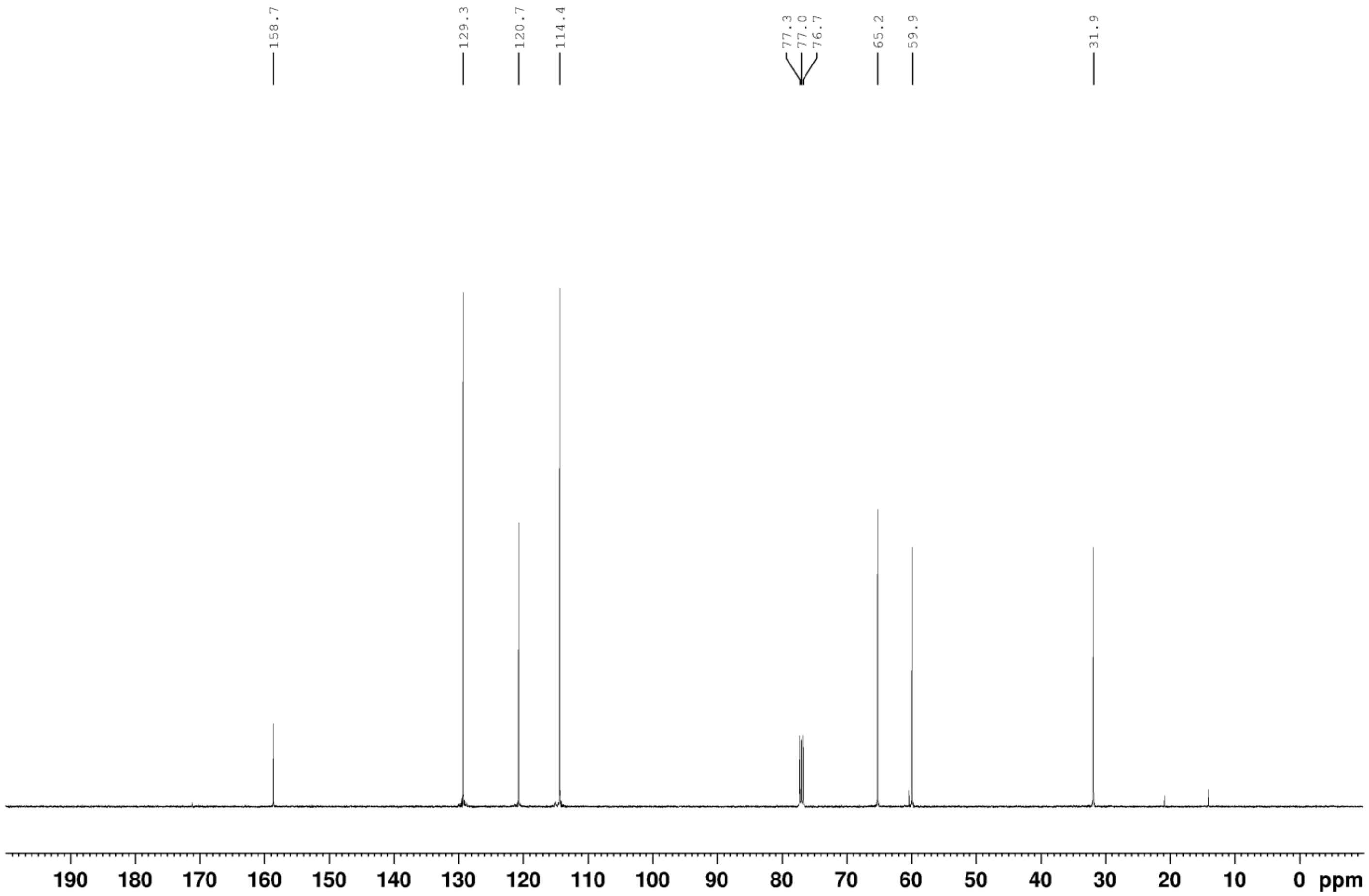

Figure S9. ^{1}H NMR (500 MHz, $CDCl_3$, 298 K) of **1j-s**.

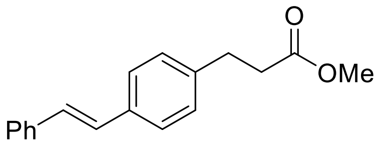

1j-s

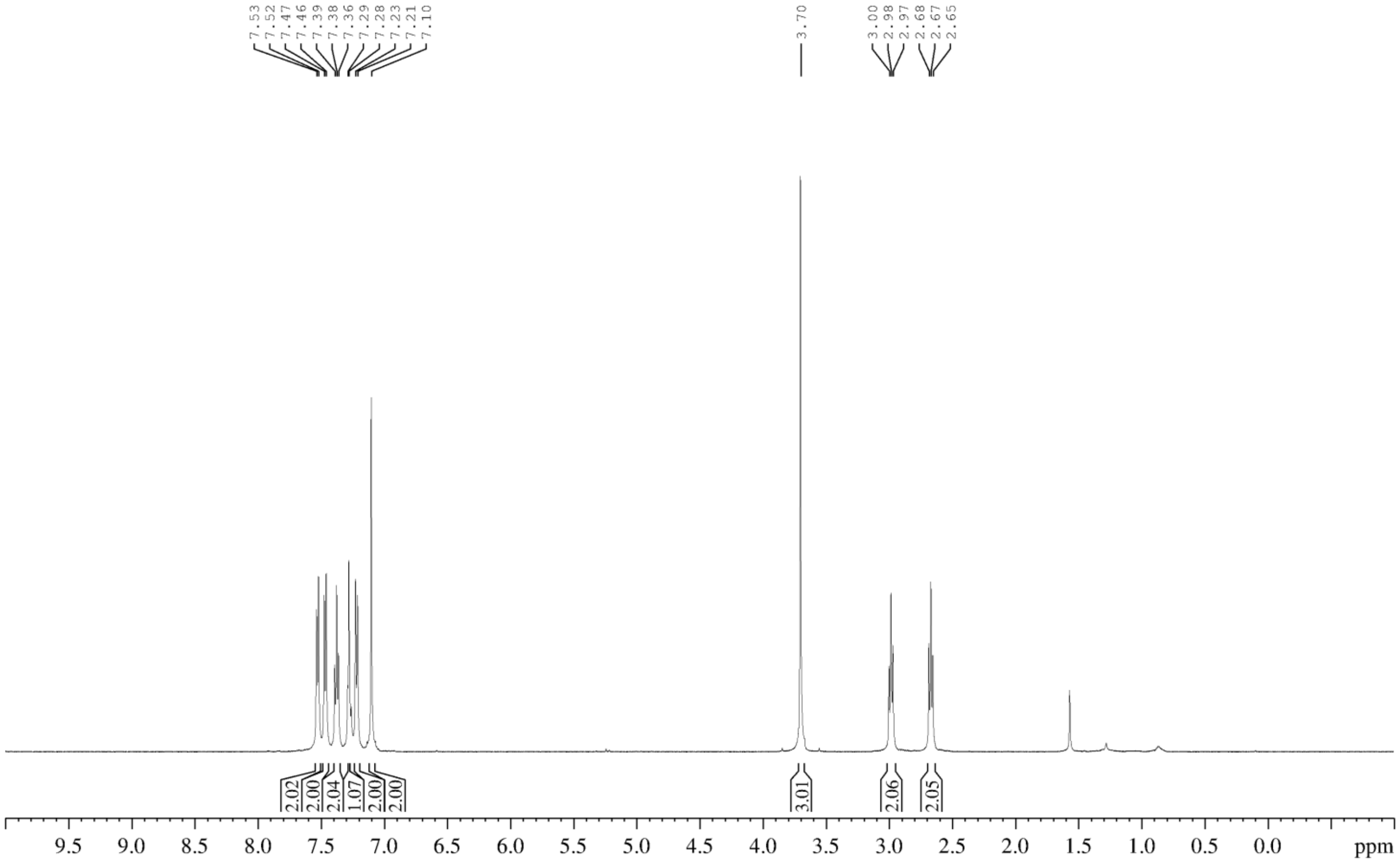

Figure S10. ^{13}C NMR (126 MHz, $CDCl_3$, 298 K) of **1j-s**.

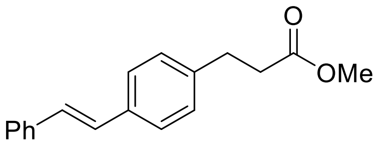

1j-s

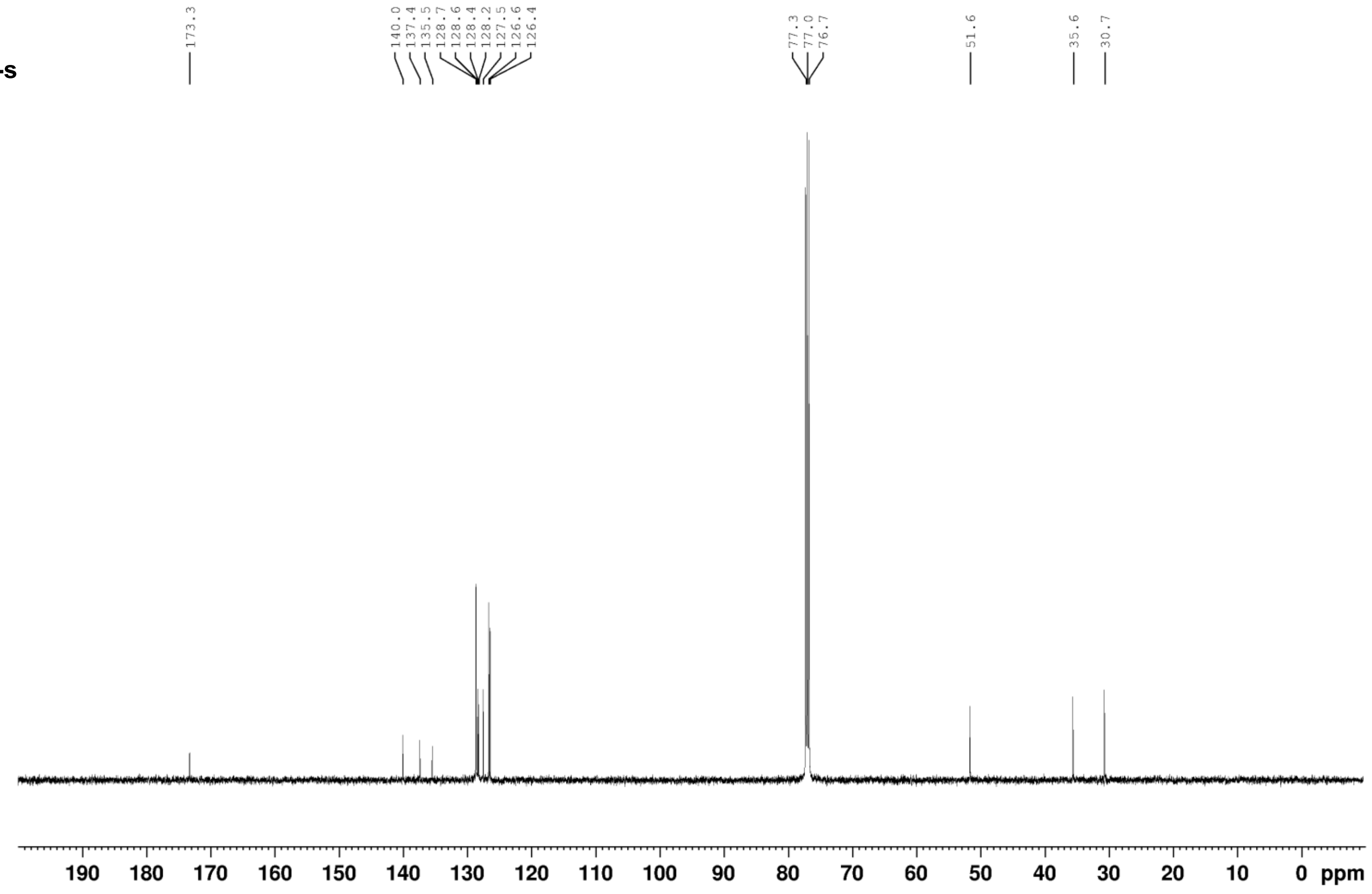

Figure S11. ^{1}H NMR (500 MHz, $CDCl_3$, 298 K) of **1j**.

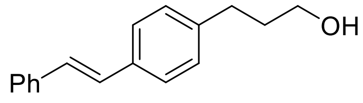

1j

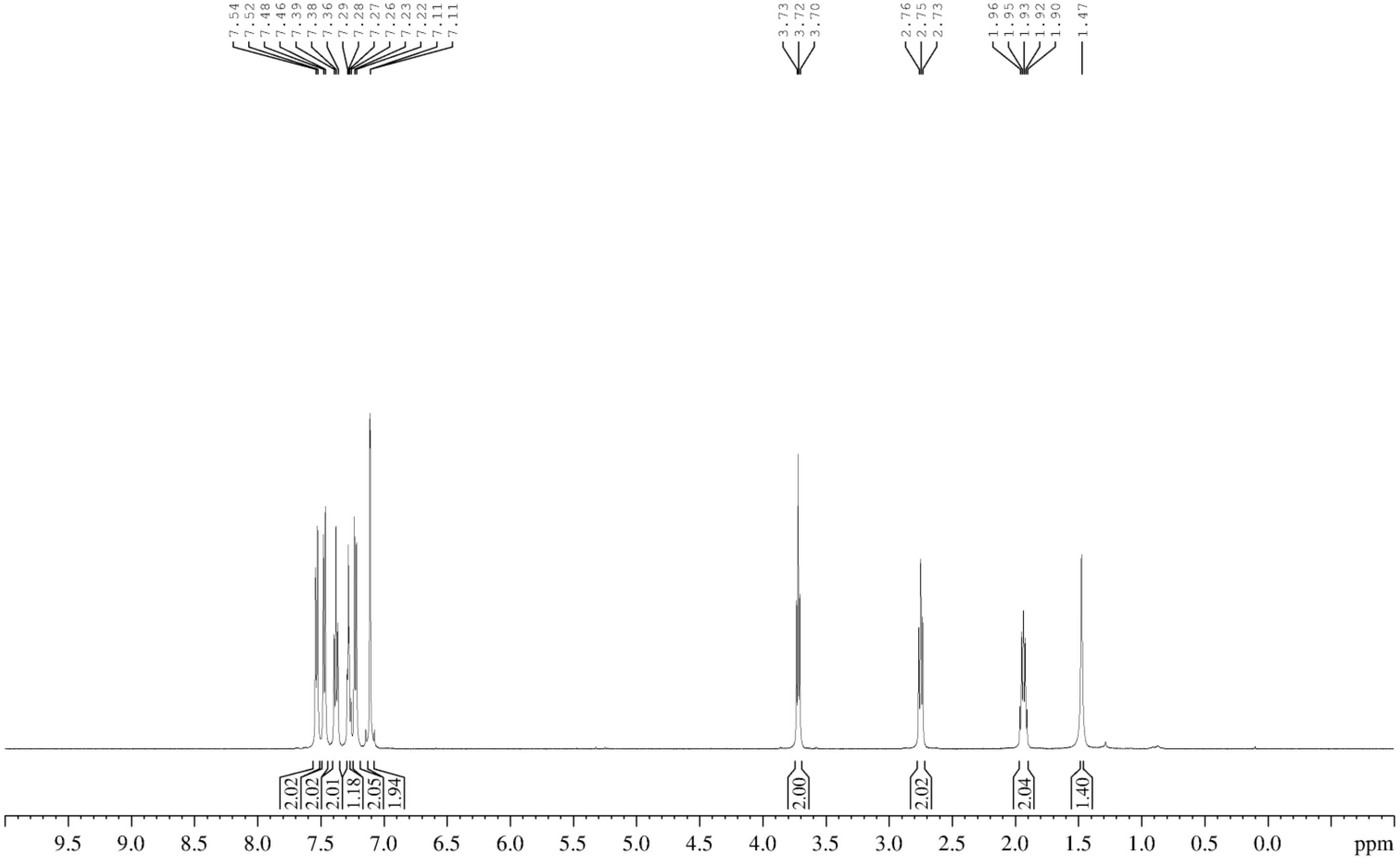

Figure S12. ^{13}C NMR (126 MHz, $CDCl_3$, 298 K) of **1j**.

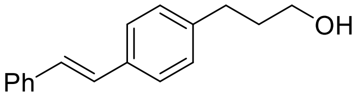

1j

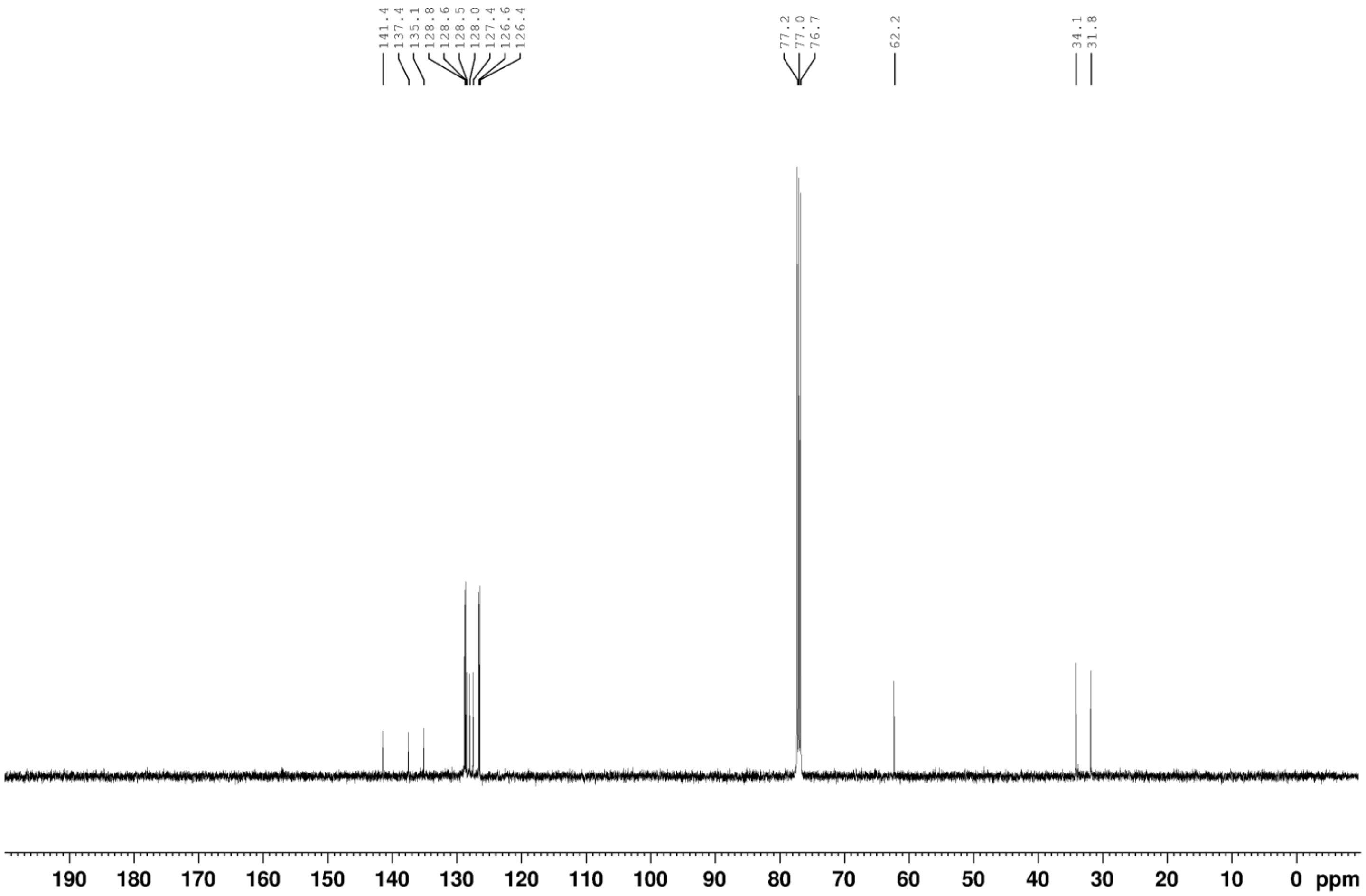

Figure S13. ^{1}H NMR (500 MHz, $CDCl_3$, 298 K) of **1k**.

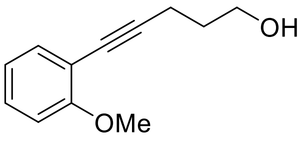

1k

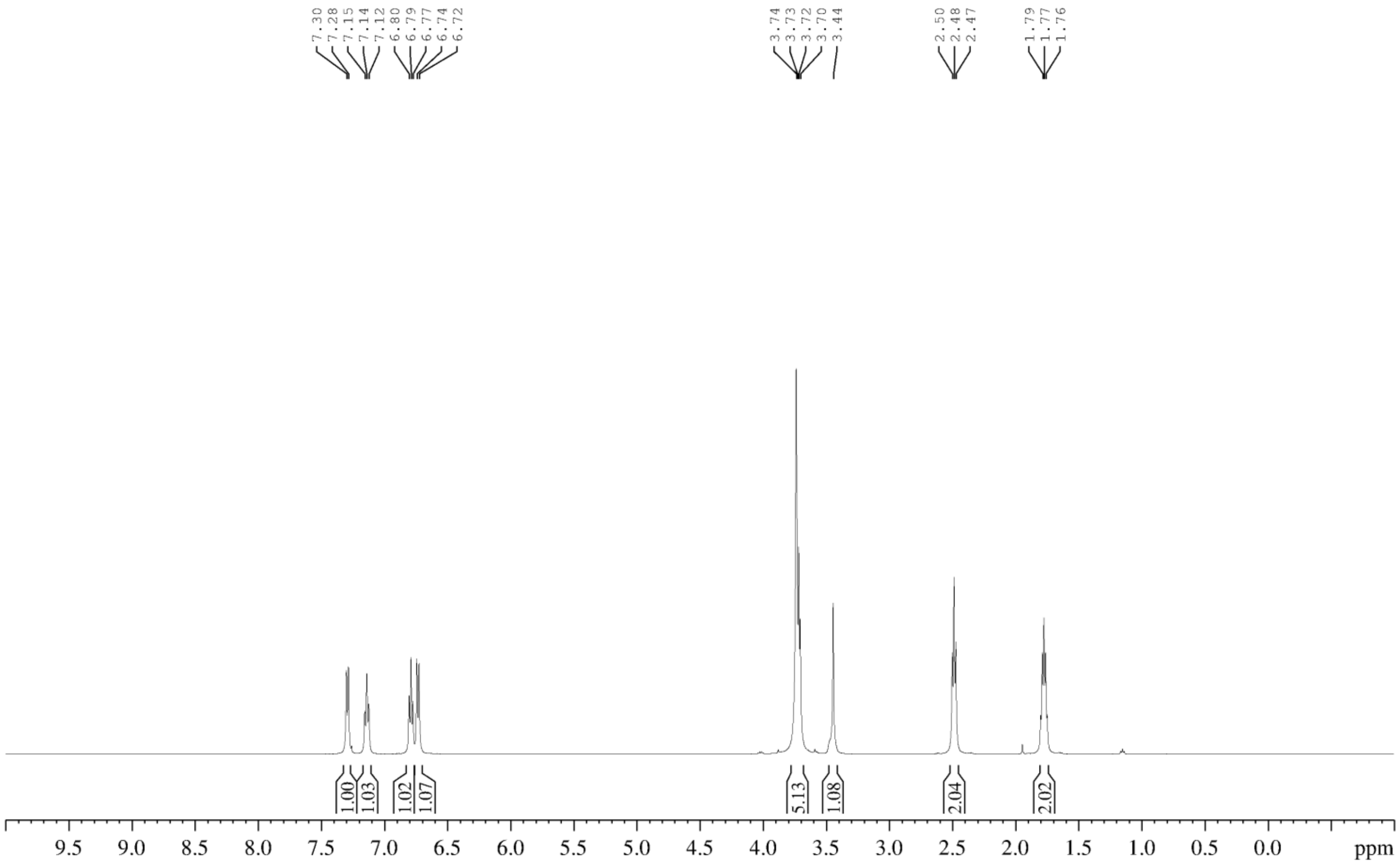

Figure S14. ^{13}C NMR (126 MHz, $CDCl_3$, 298 K) of **1k**.

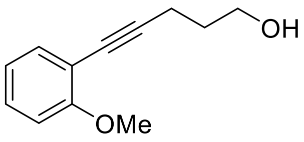

1k

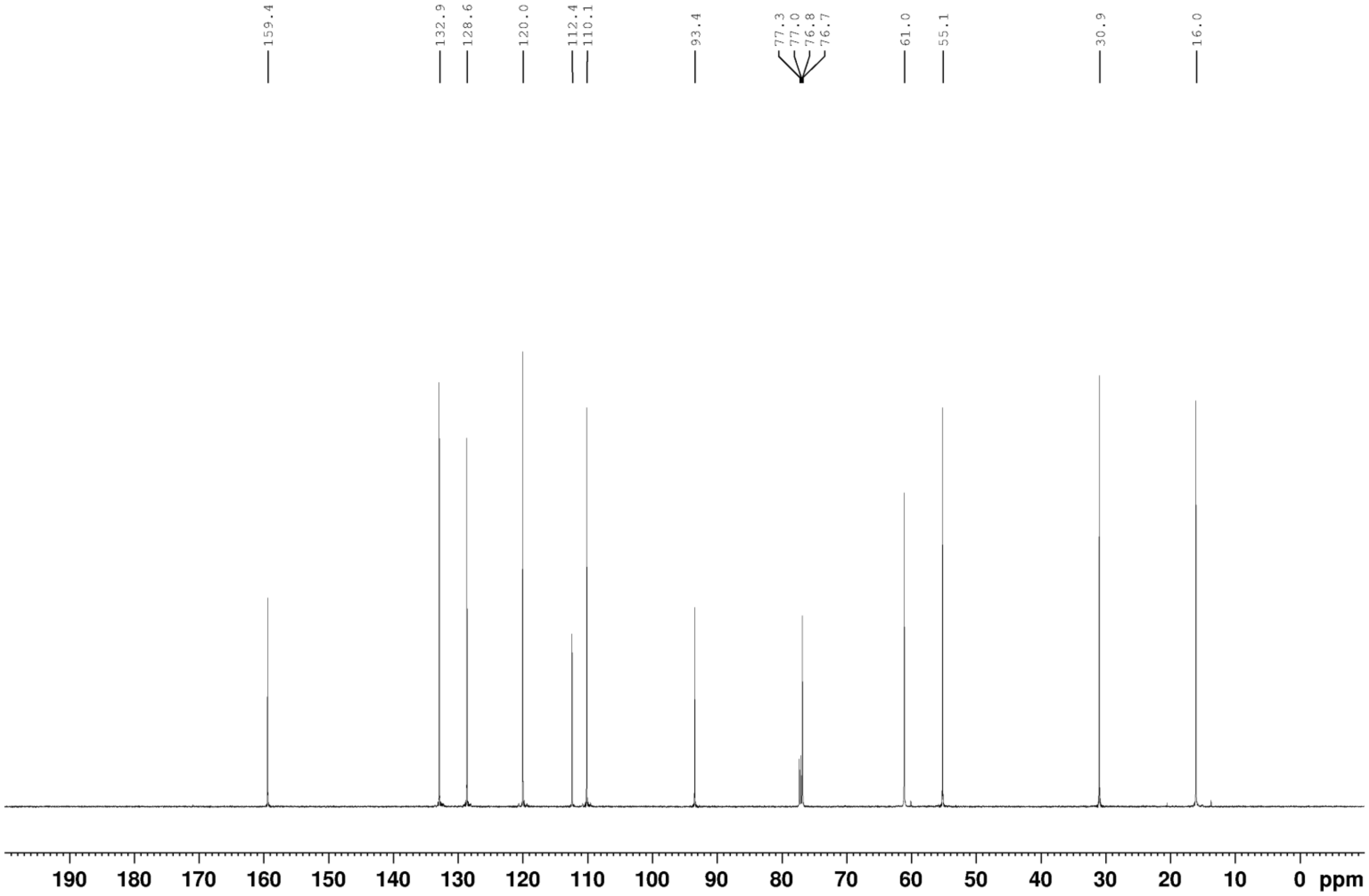

Figure S15. ^{1}H NMR (500 MHz, $CDCl_3$, 298 K) of **1l**.

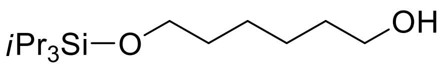

1l

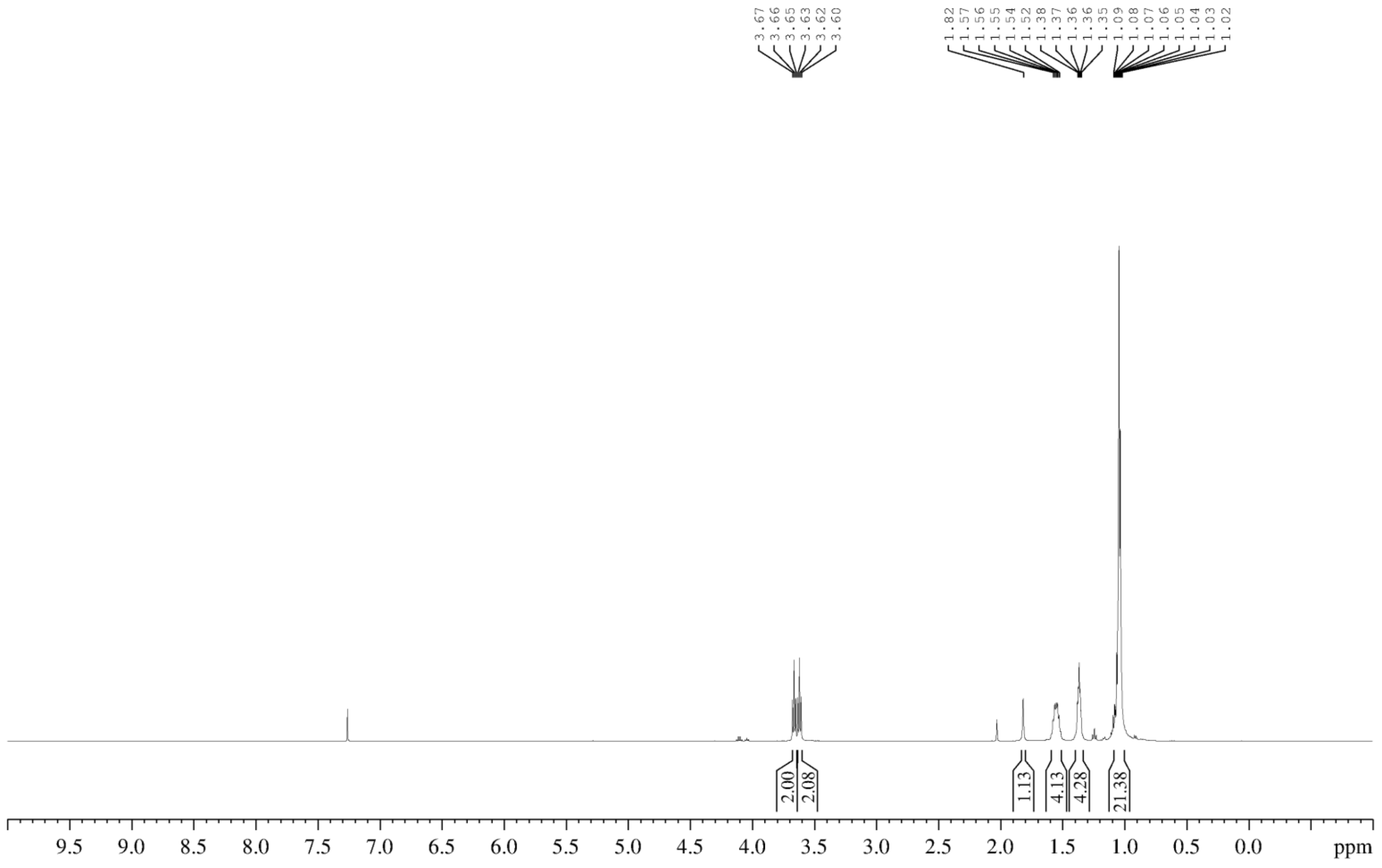

Figure S16. ^{13}C NMR (126 MHz, $CDCl_3$, 298 K) of **1l**.

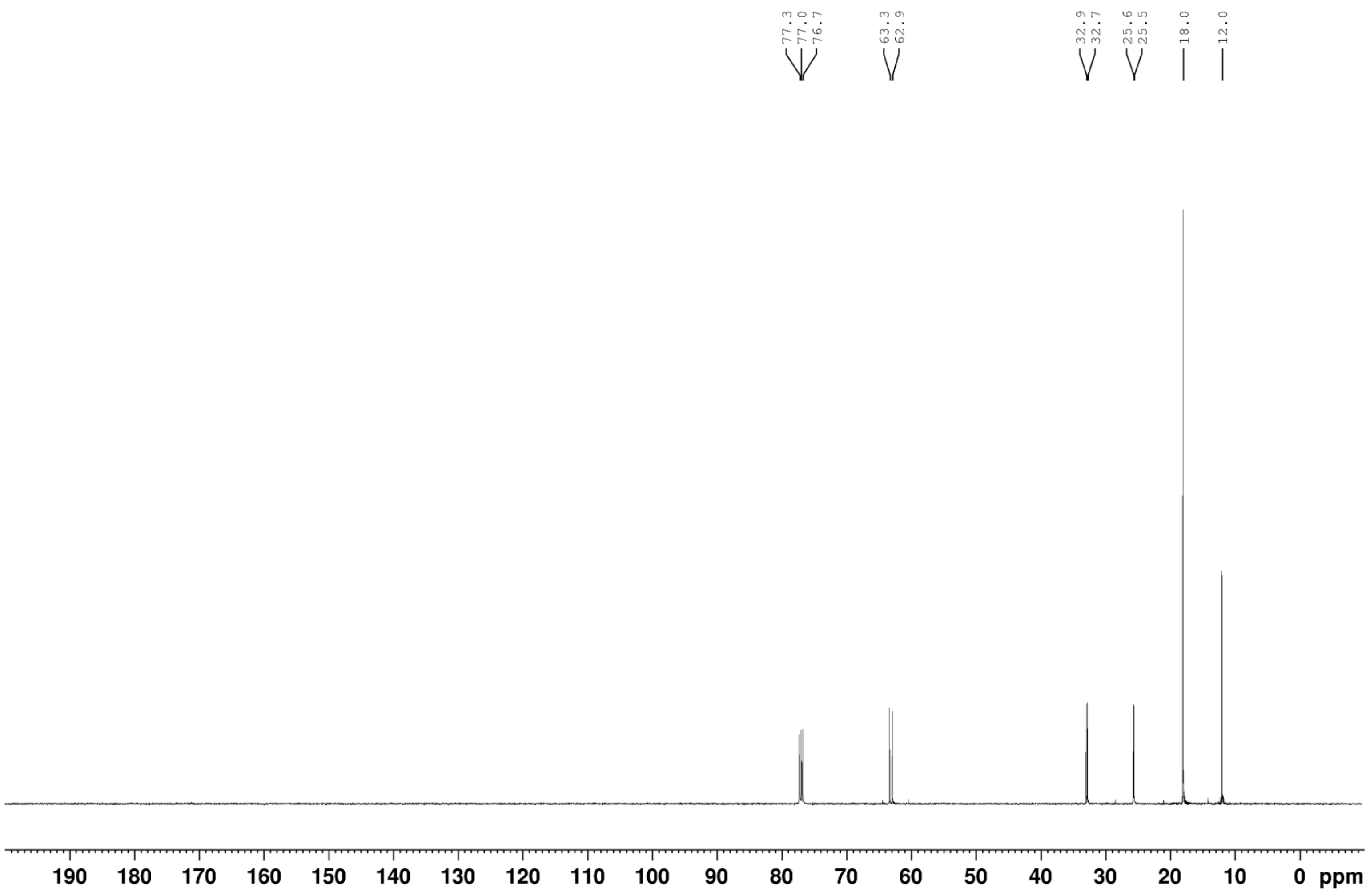

Figure S17. ^{1}H/^{29}Si HMQC NMR (500/99 MHz, $CDCl_3$, 298 K, optimized for *J* = 7 Hz) of **1l**.

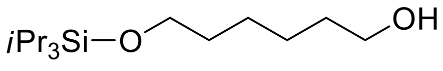

1l

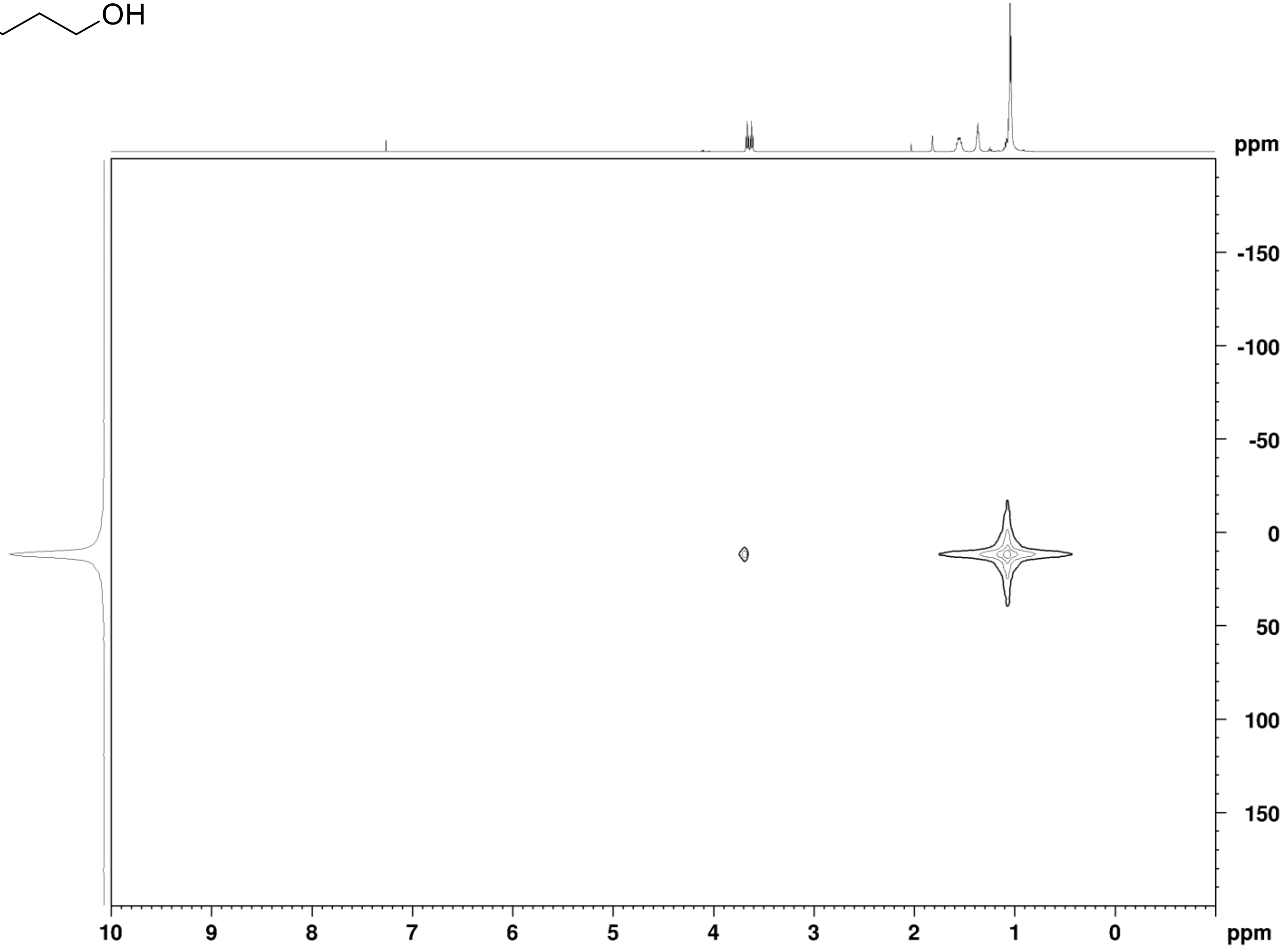

Figure S18. ^{1}H NMR (500 MHz, $CDCl_3$, 298 K) of **1m**.

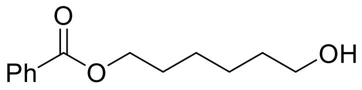

1m

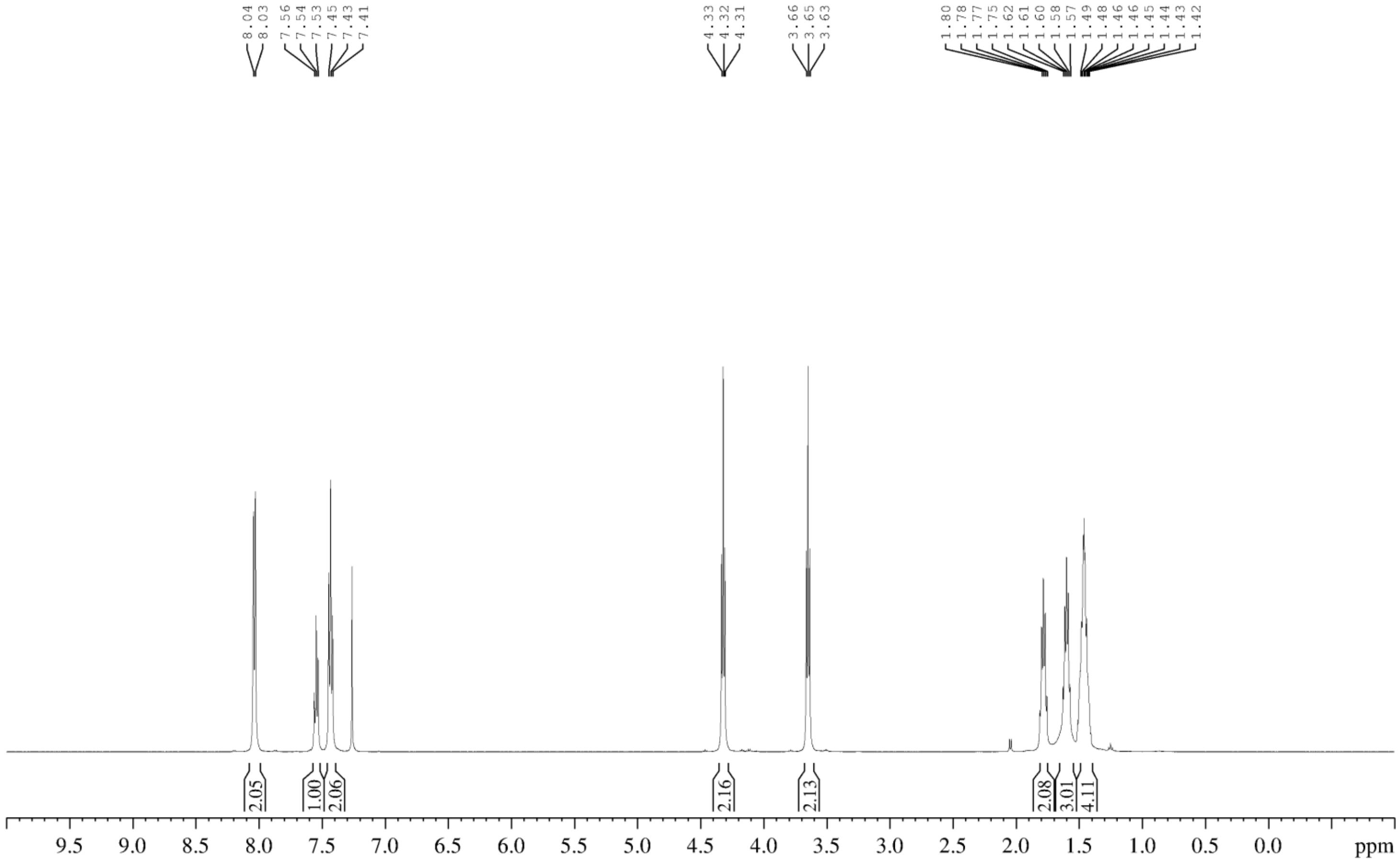

Figure S19. ^{13}C NMR (126 MHz, $CDCl_3$, 298 K) of **1m**.

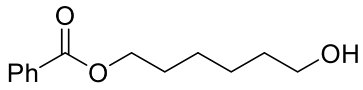

1m

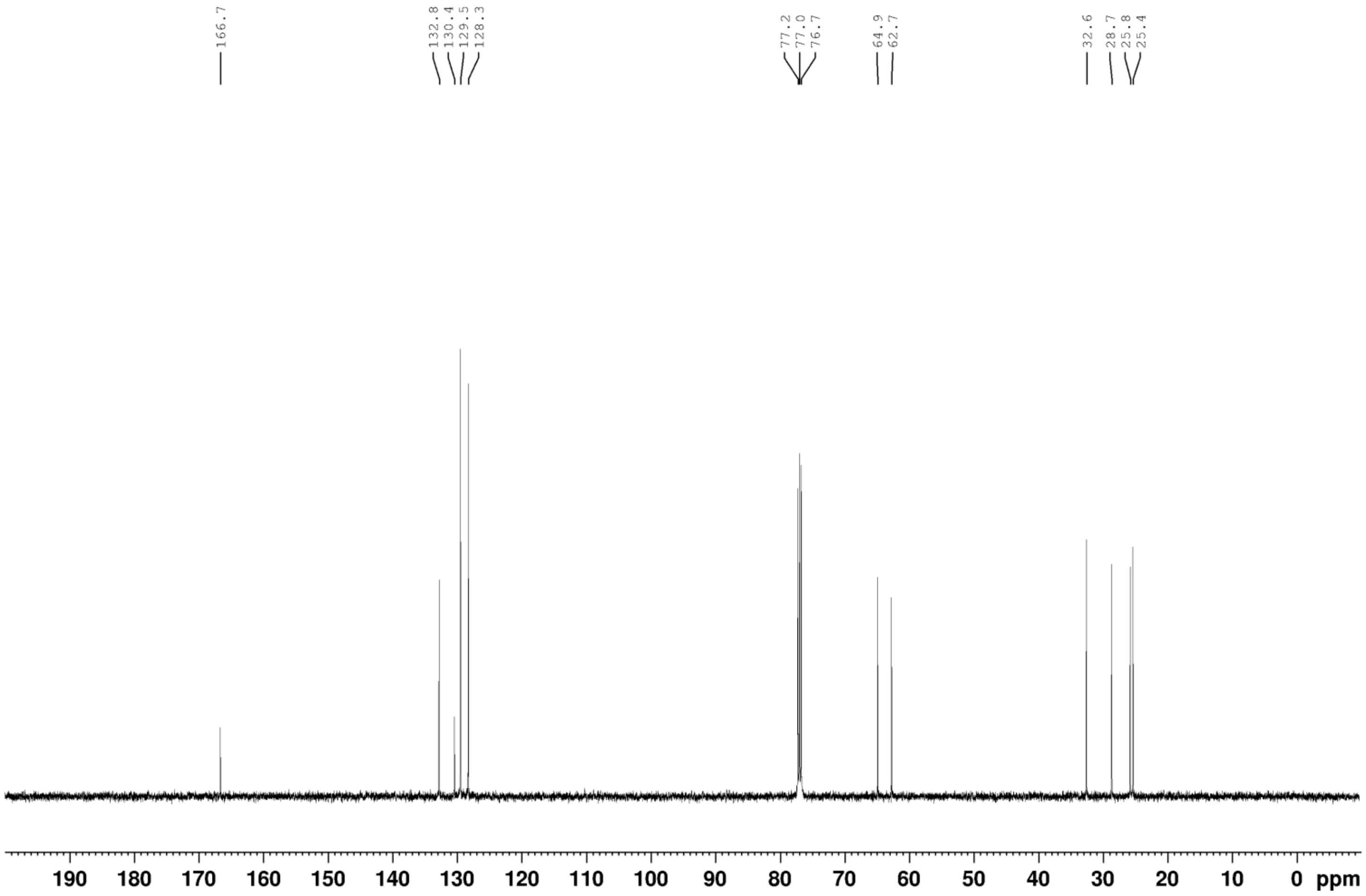

Figure S20. ^{1}H NMR (500 MHz, $CDCl_3$, 298 K) of **1n**.

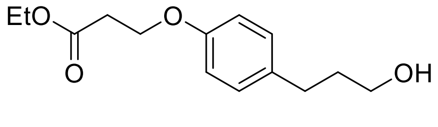

1n

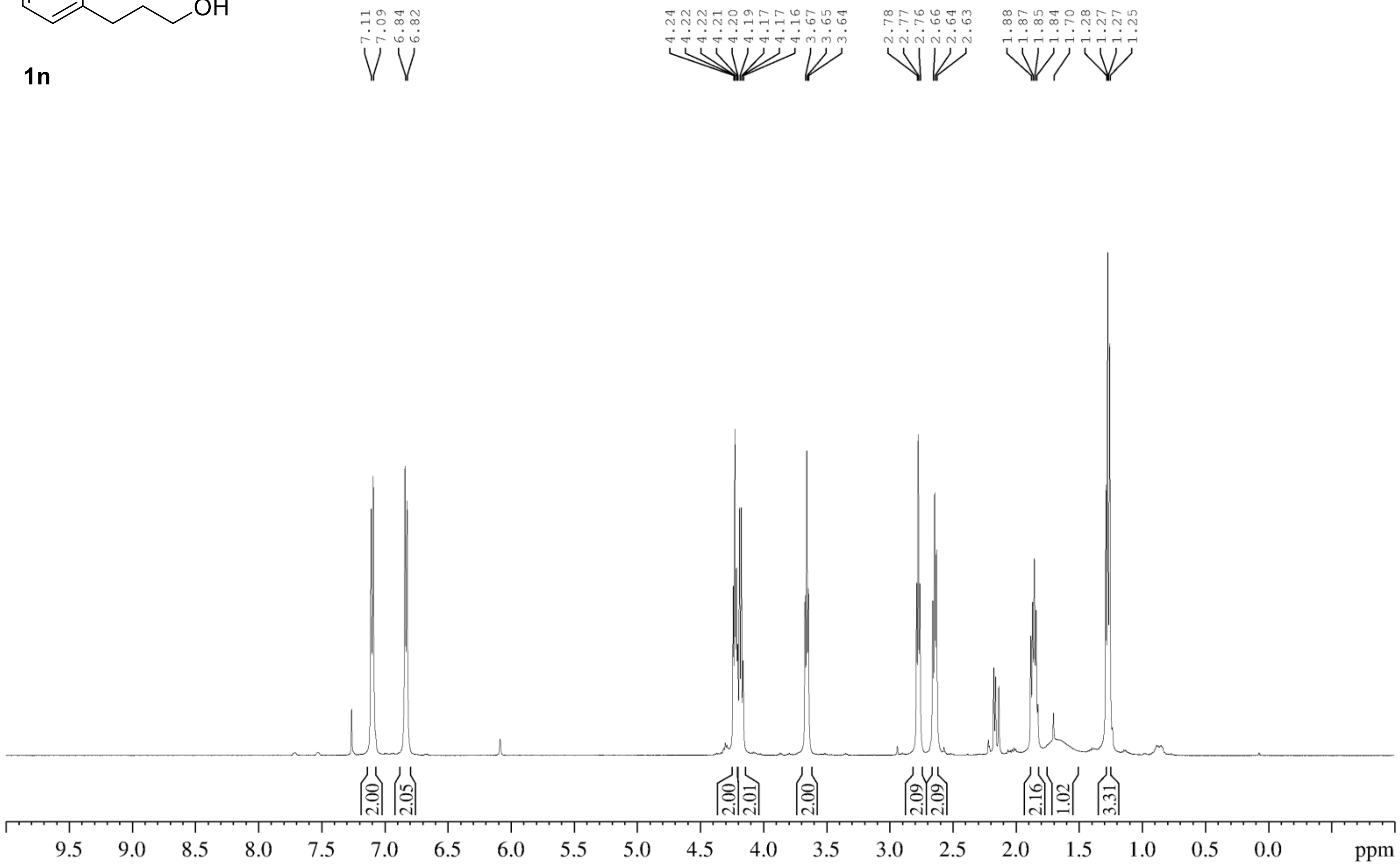

Figure S21. ^{13}C NMR (126 MHz, $CDCl_3$, 298 K) of **1n**.

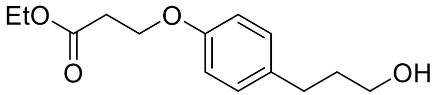

1n

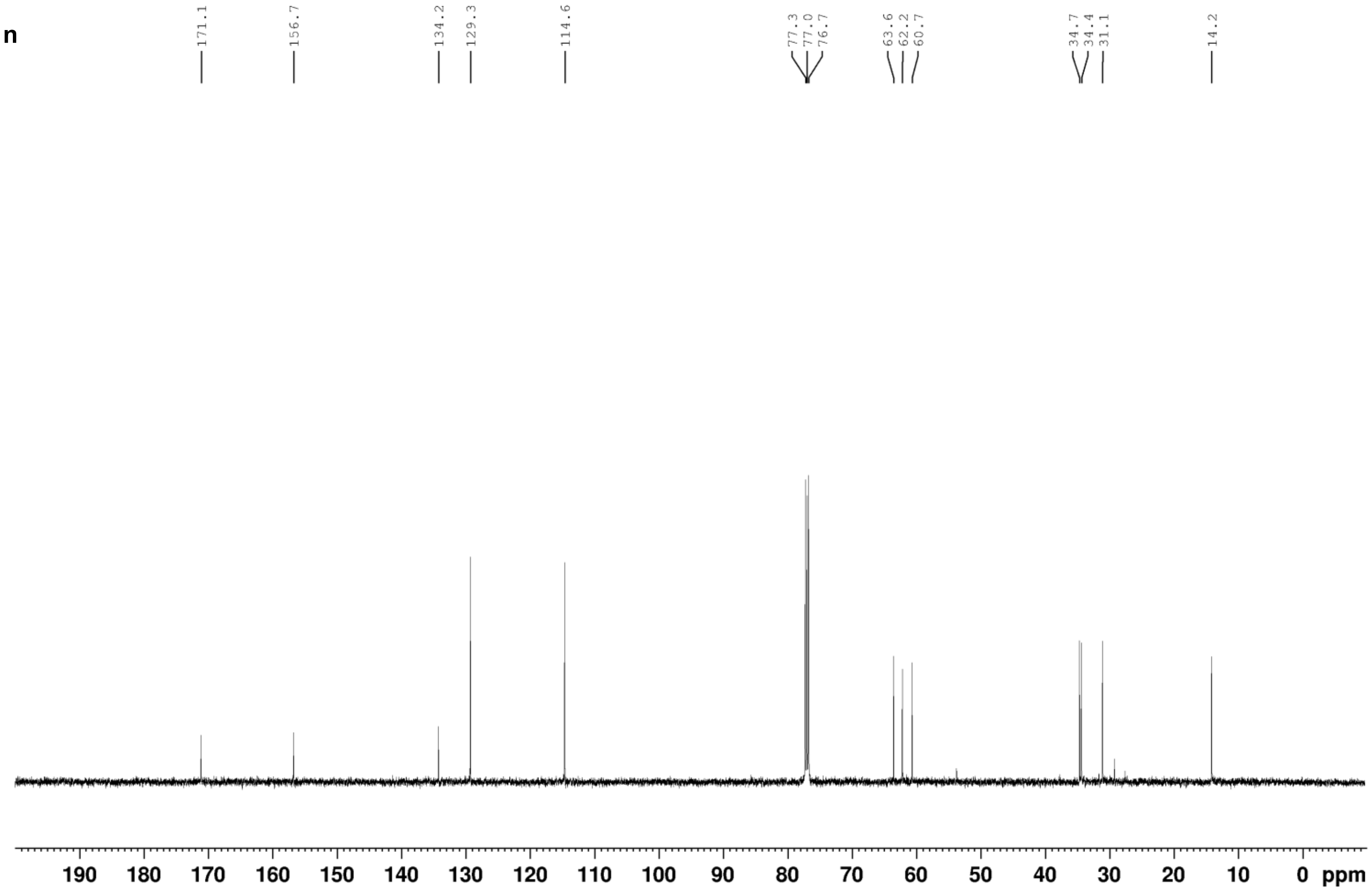

Figure S22. ^1H NMR (500 MHz, $CDCl_3$, 298 K) of **1o**.

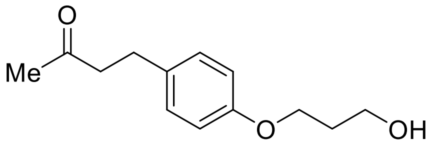

1o

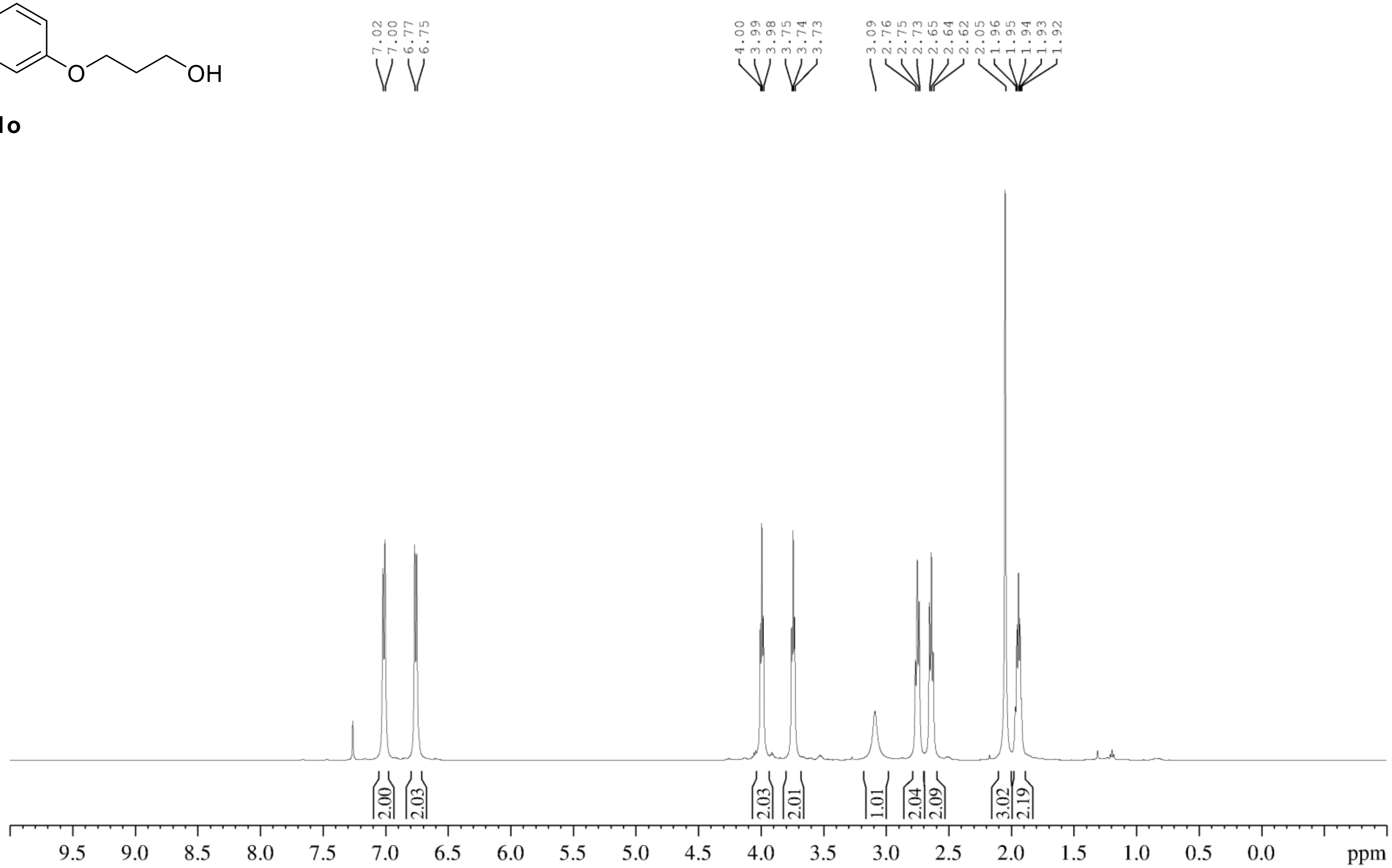

Figure S23. ^{13}C NMR (126 MHz, $CDCl_3$, 298 K) of **1o**.

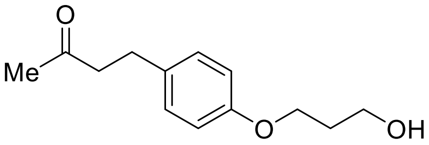

1o

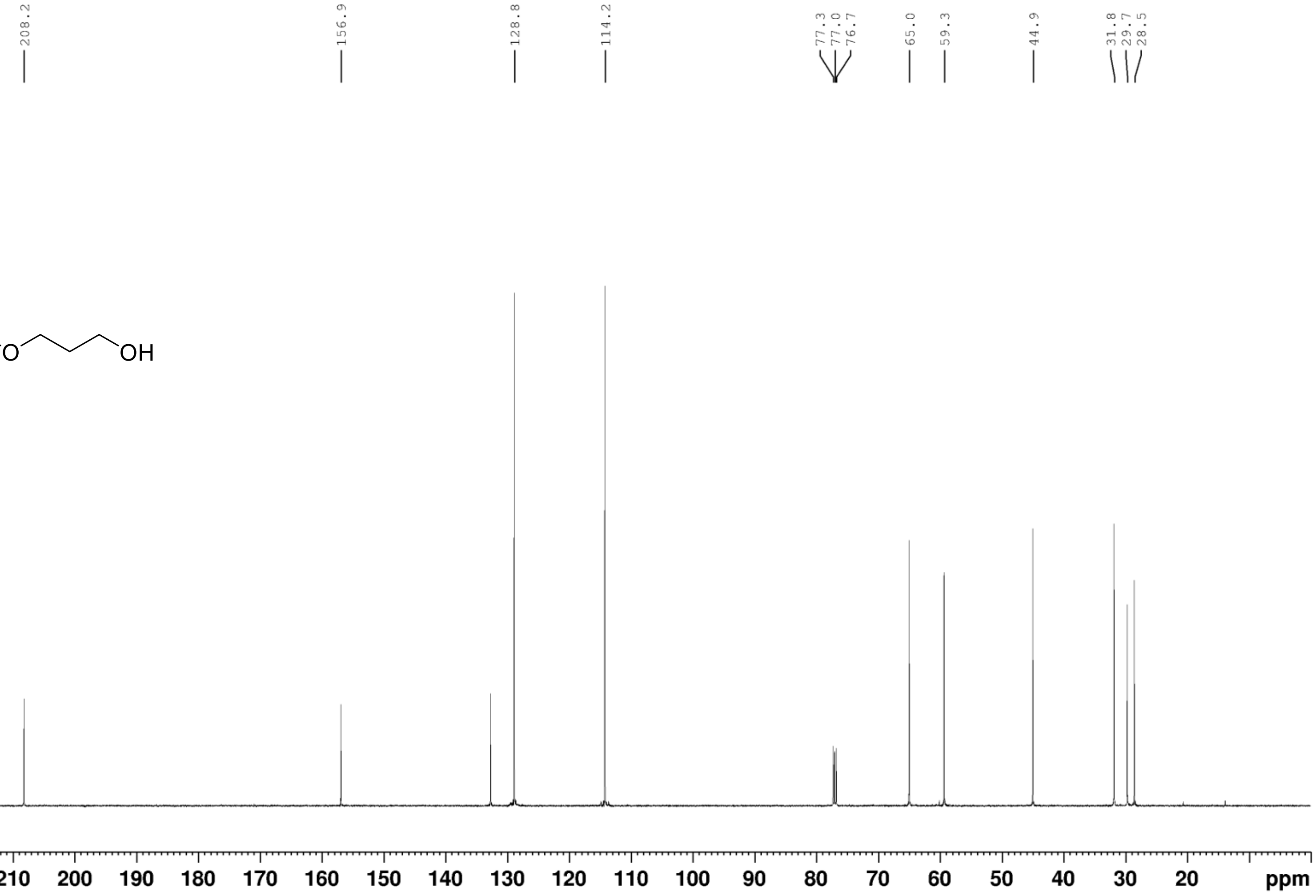

Figure S24. ^{1}H NMR (500 MHz, $CDCl_3$, 298 K) of **1p**.

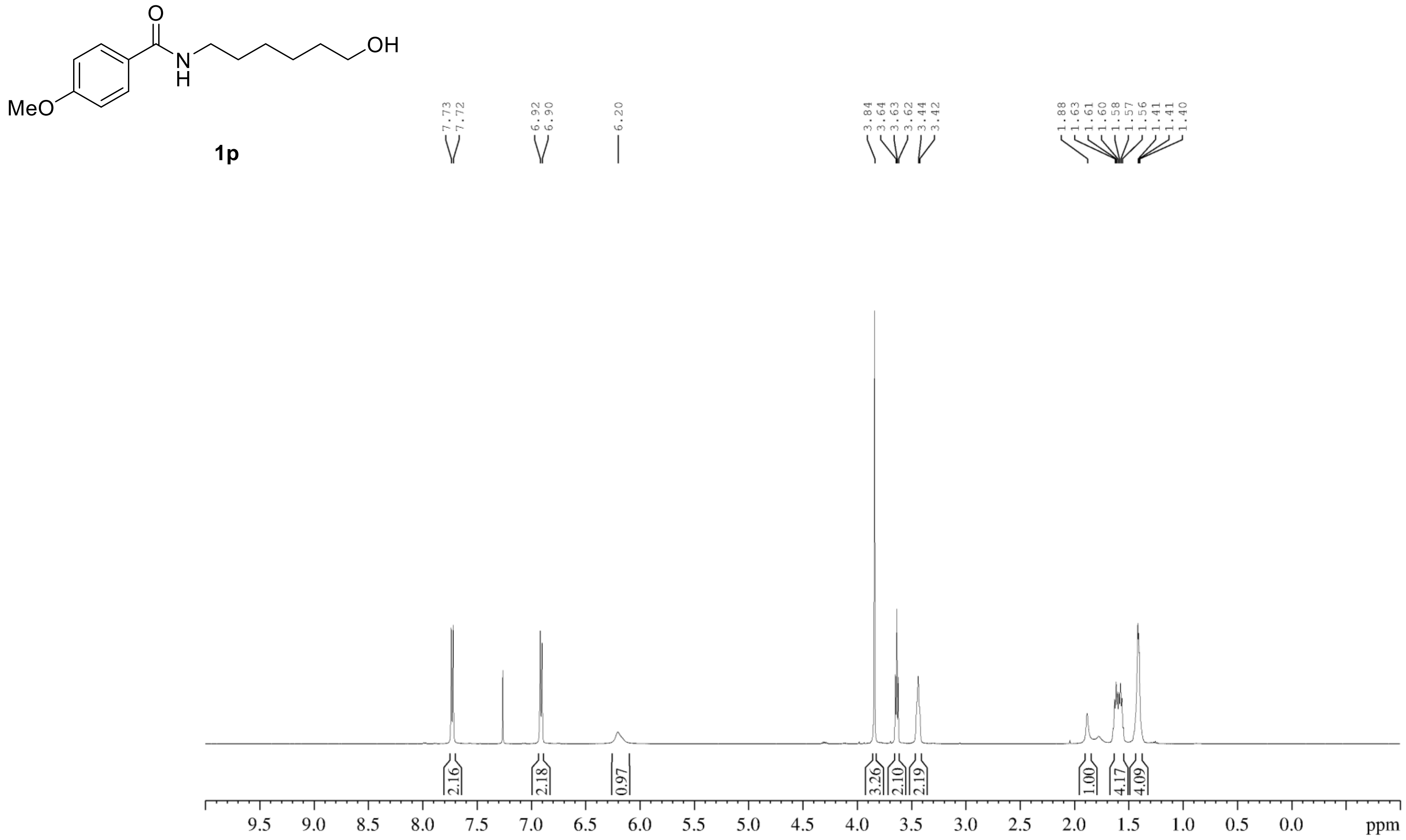

Figure S25. ^{13}C NMR (126 MHz, $CDCl_3$, 298 K) of **1p**.

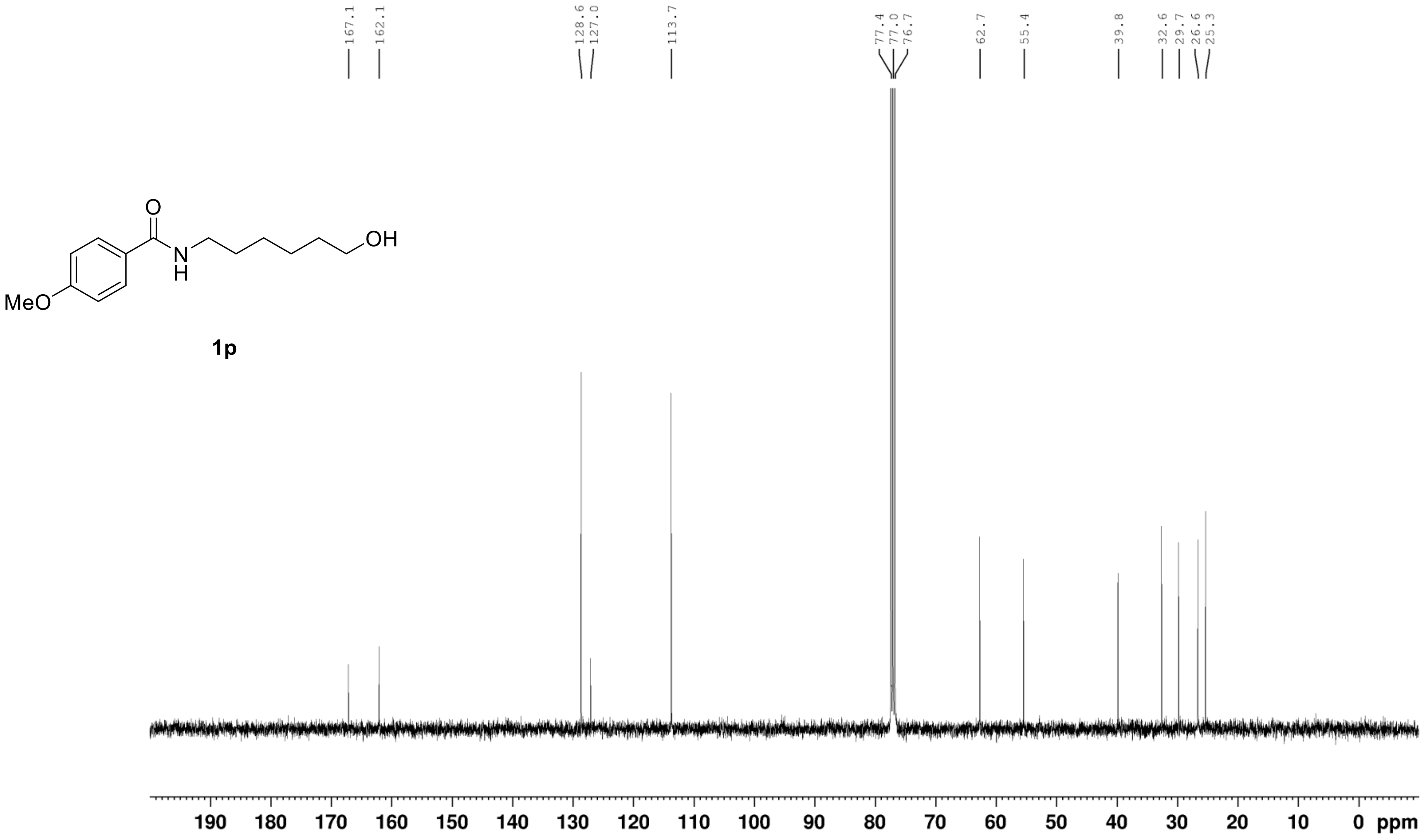

Figure S26. ^{1}H NMR (500 MHz, $CDCl_3$, 298 K) of **1q**.

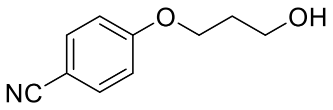

1q

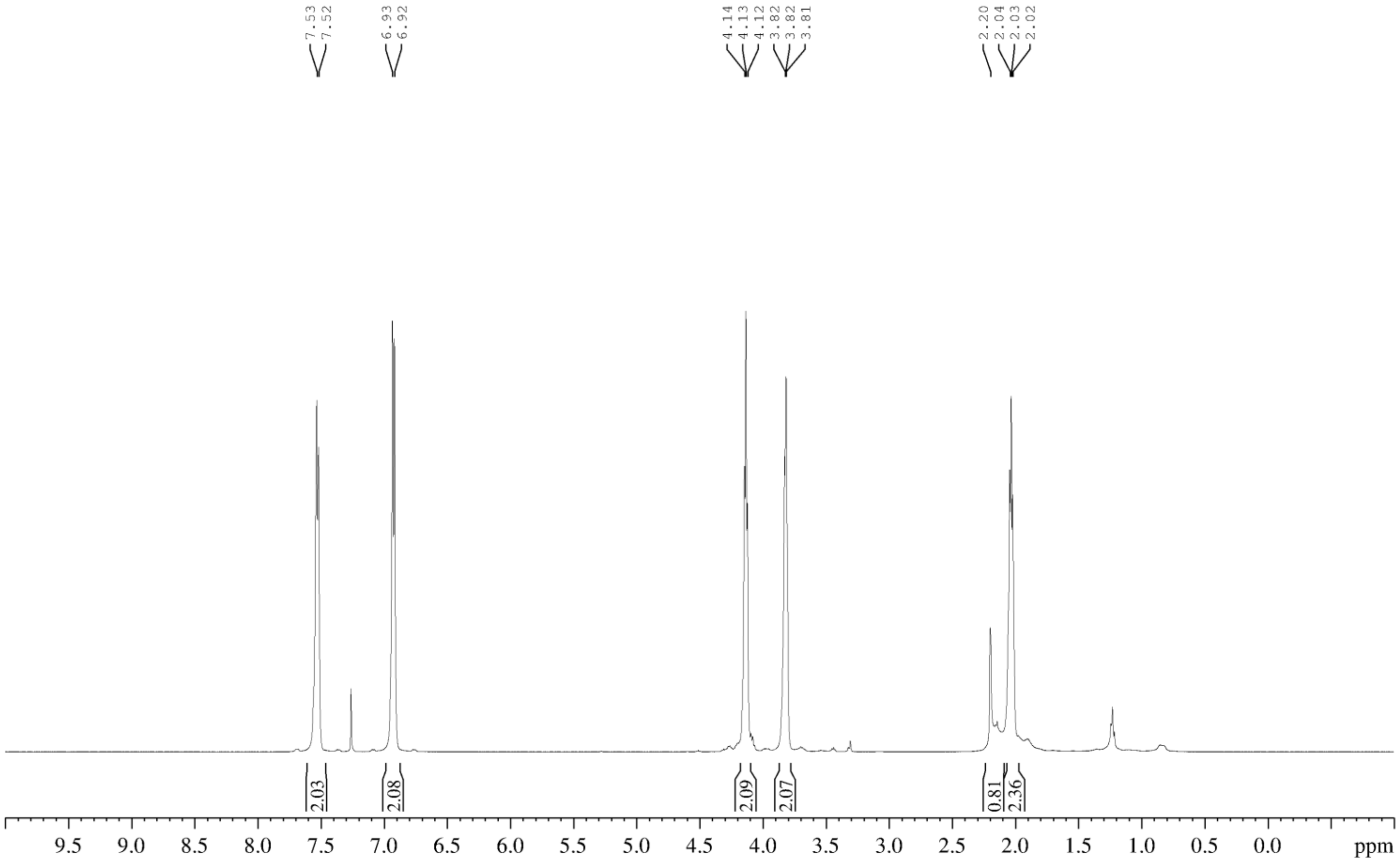

Figure S27. ^{13}C NMR (126 MHz, $CDCl_3$, 298 K) of **1q**.

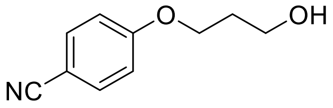

1q

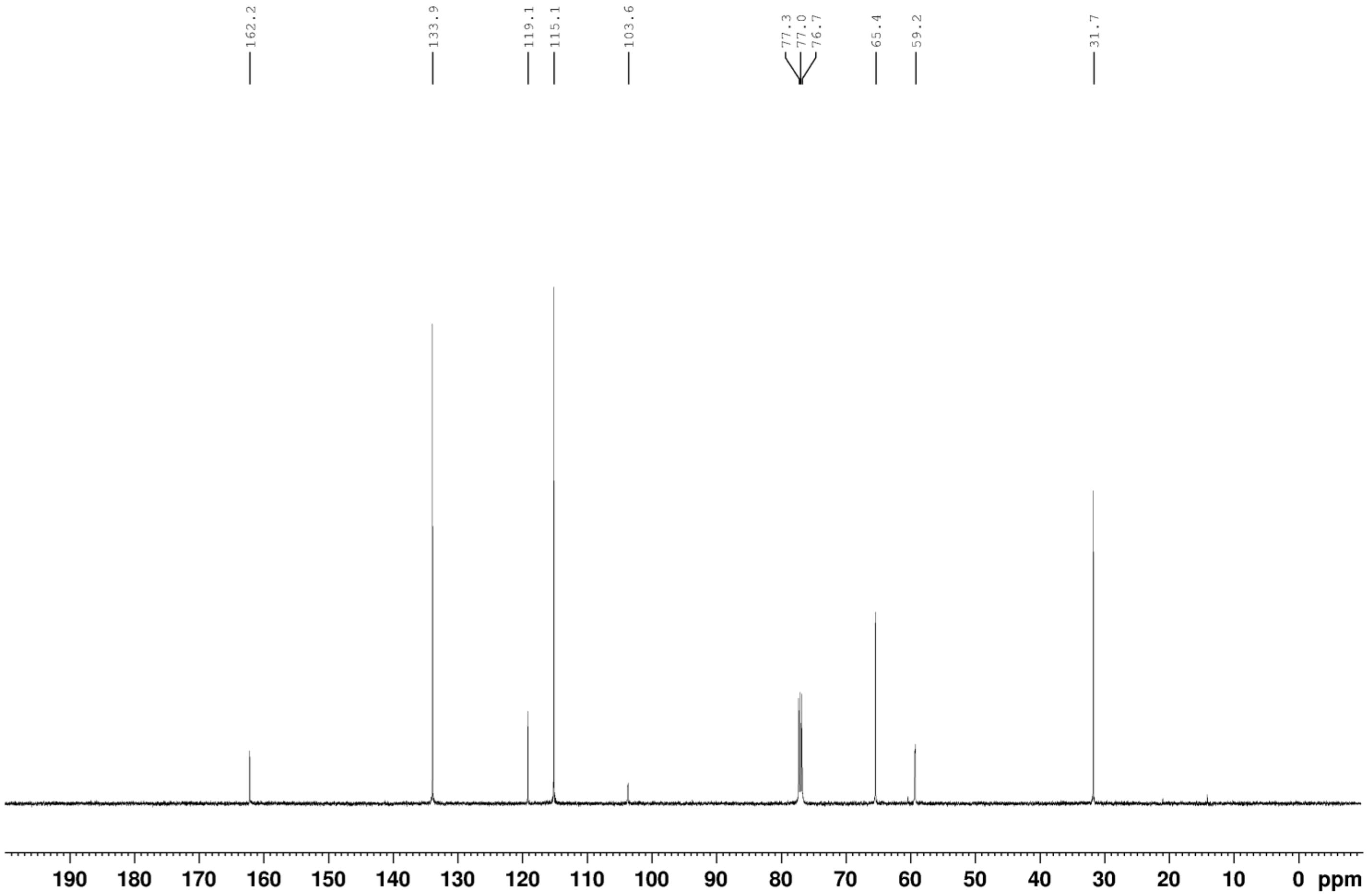

Figure S28. ^{1}H NMR (500 MHz, $CDCl_3$, 298 K) of **1r**.

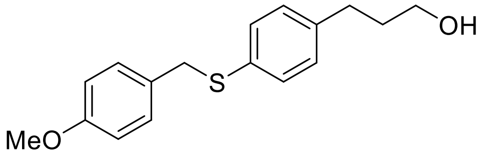

1r

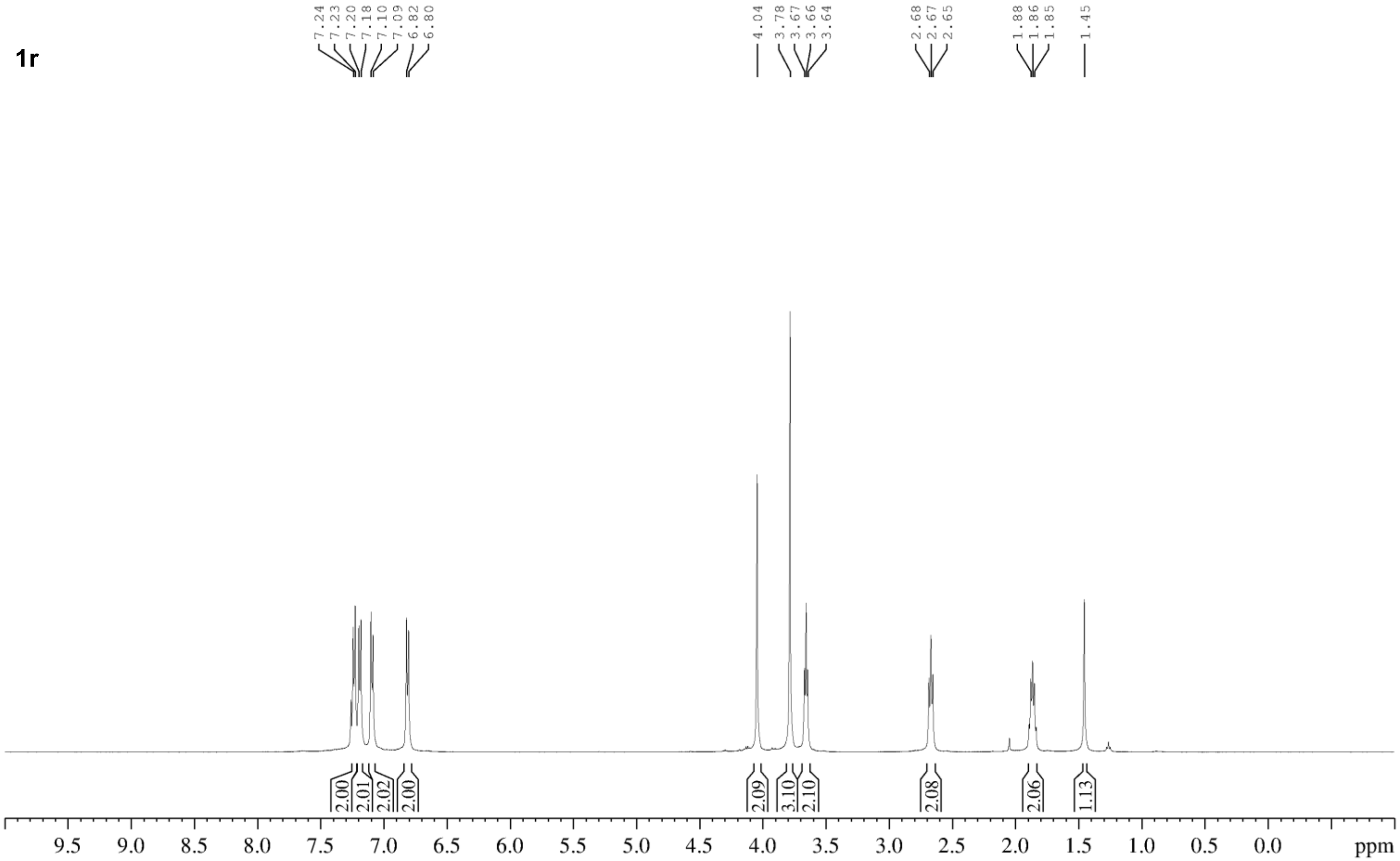

Figure S29. ^{13}C NMR (126 MHz, $CDCl_3$, 298 K) of **1r**.

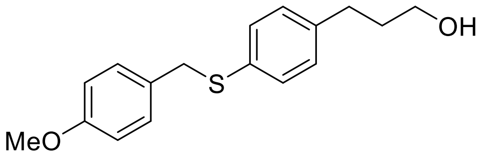

1r

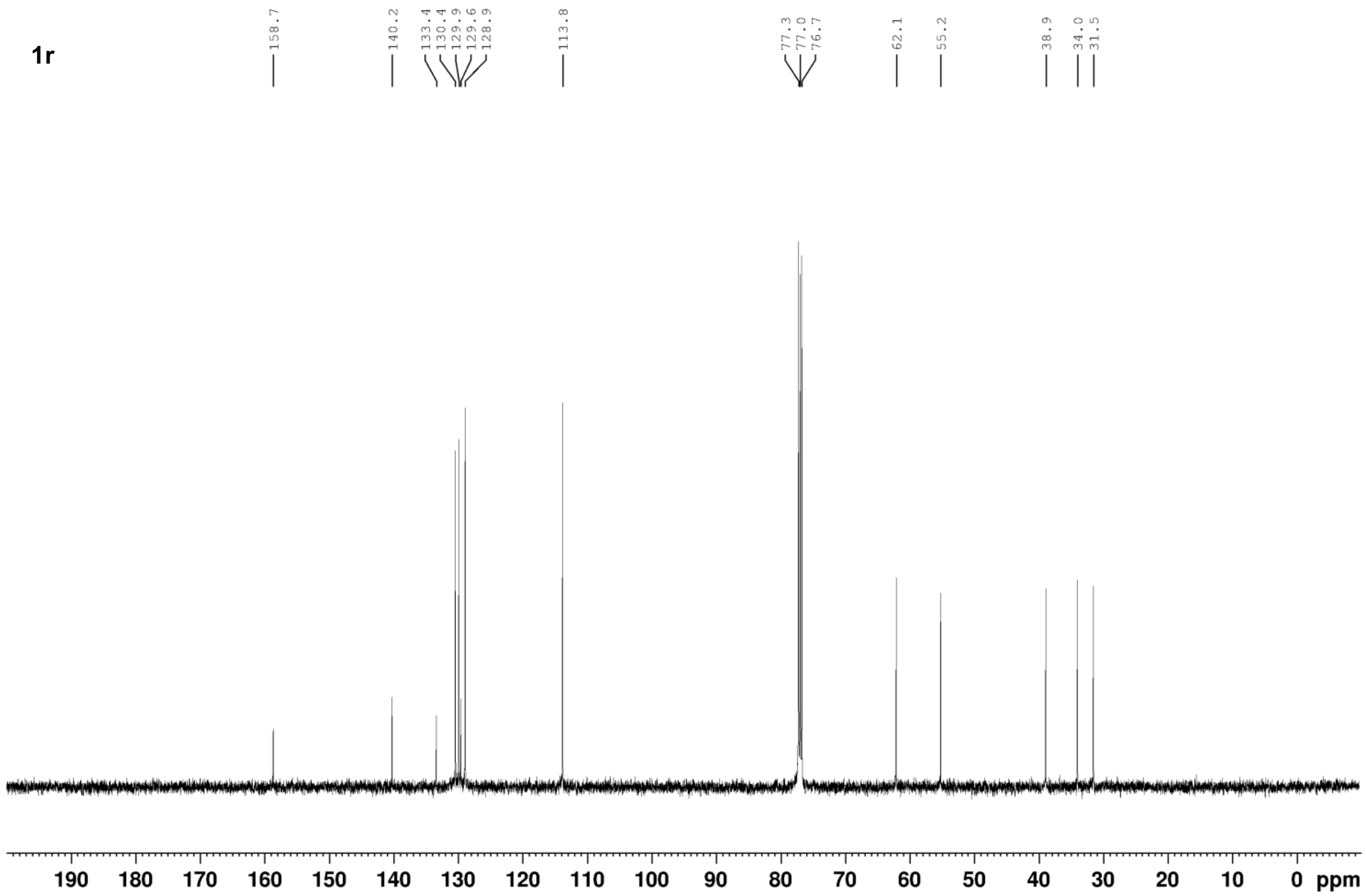

Figure S30. ^{1}H NMR (500 MHz, $CDCl_3$, 298 K) of **1s**.

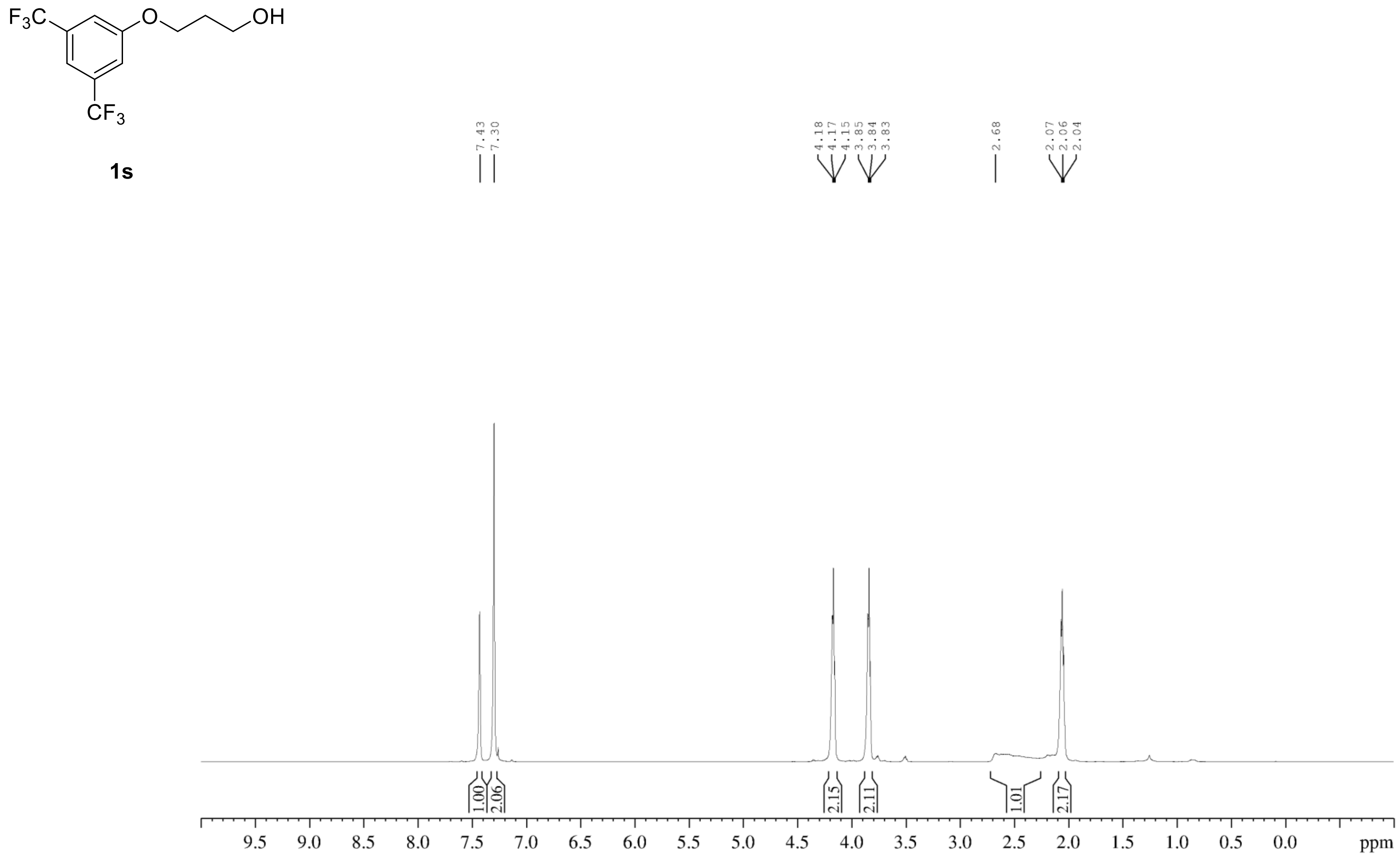

Figure S31. ^{13}C NMR (126 MHz, $CDCl_3$, 298 K) of **1s**.

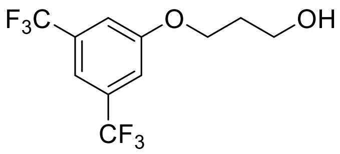

1s

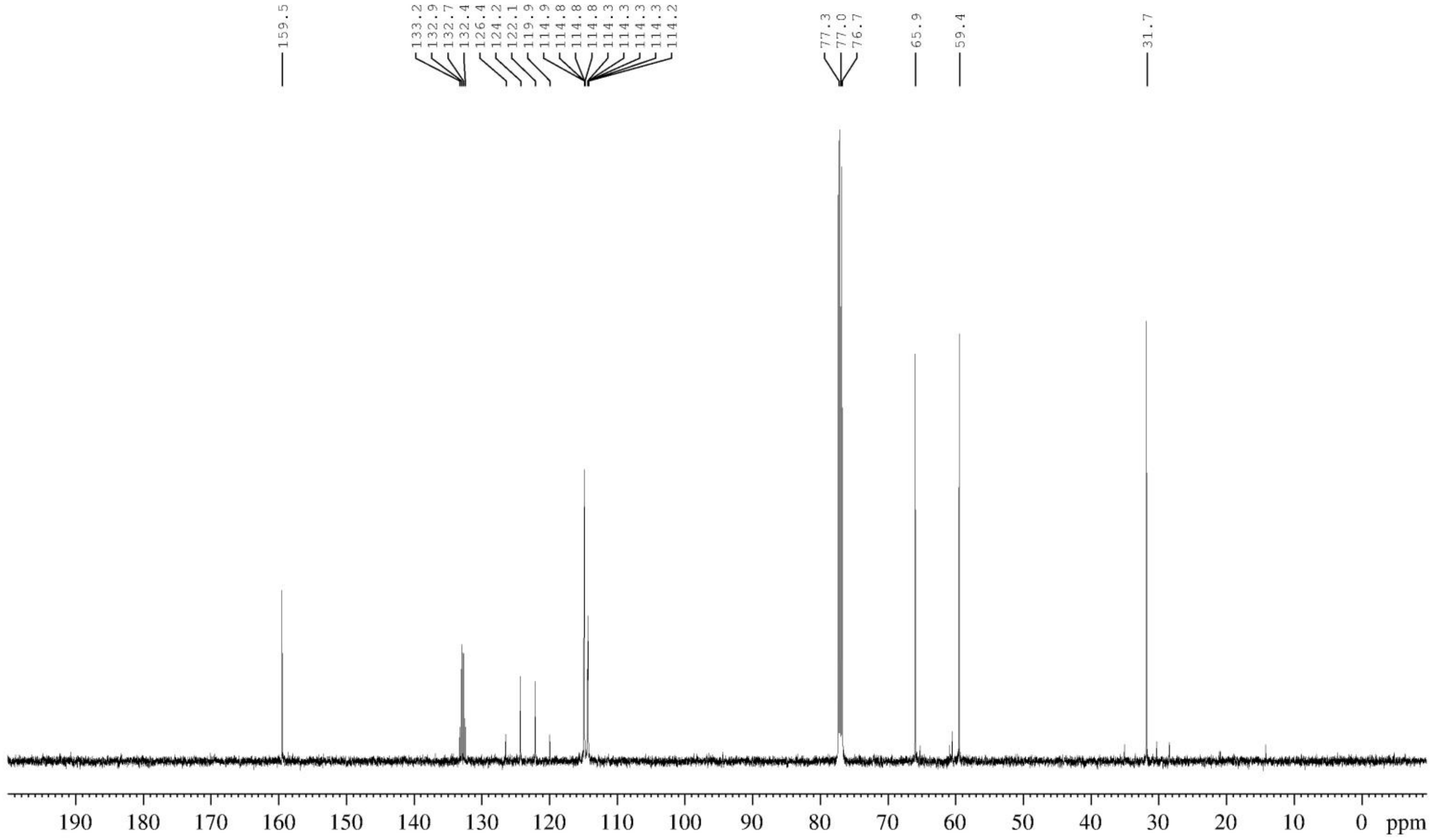

Figure S32. ^{19}F NMR (471 MHz, $CDCl_3$, 298 K) of **1s**.

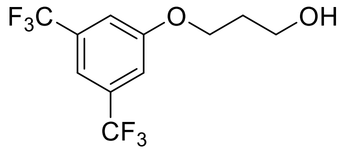

1s

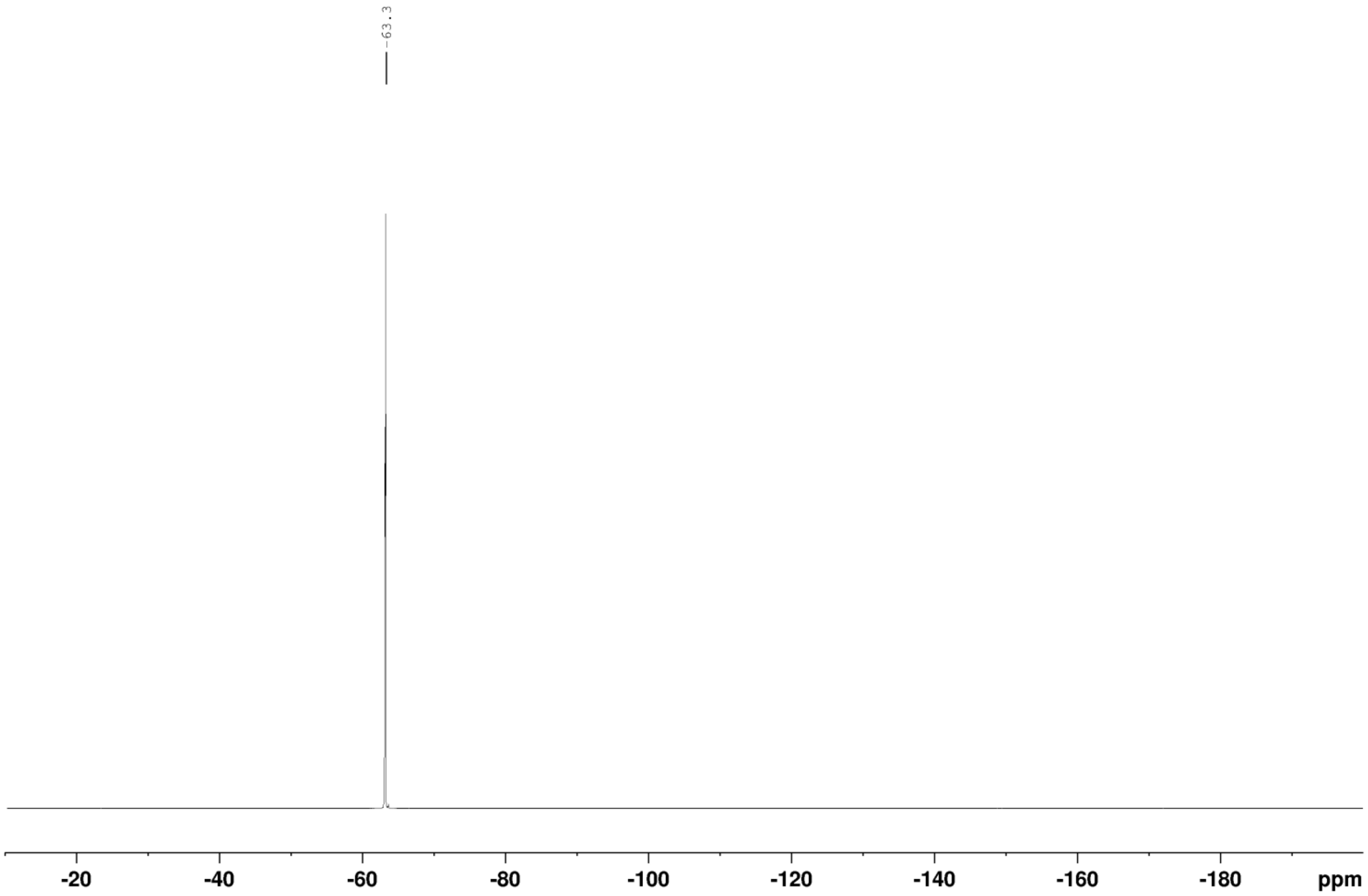

Figure S33. ^{1}H NMR (500 MHz, $CDCl_3$, 298 K) of **1t**.

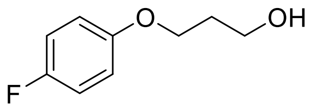

1t

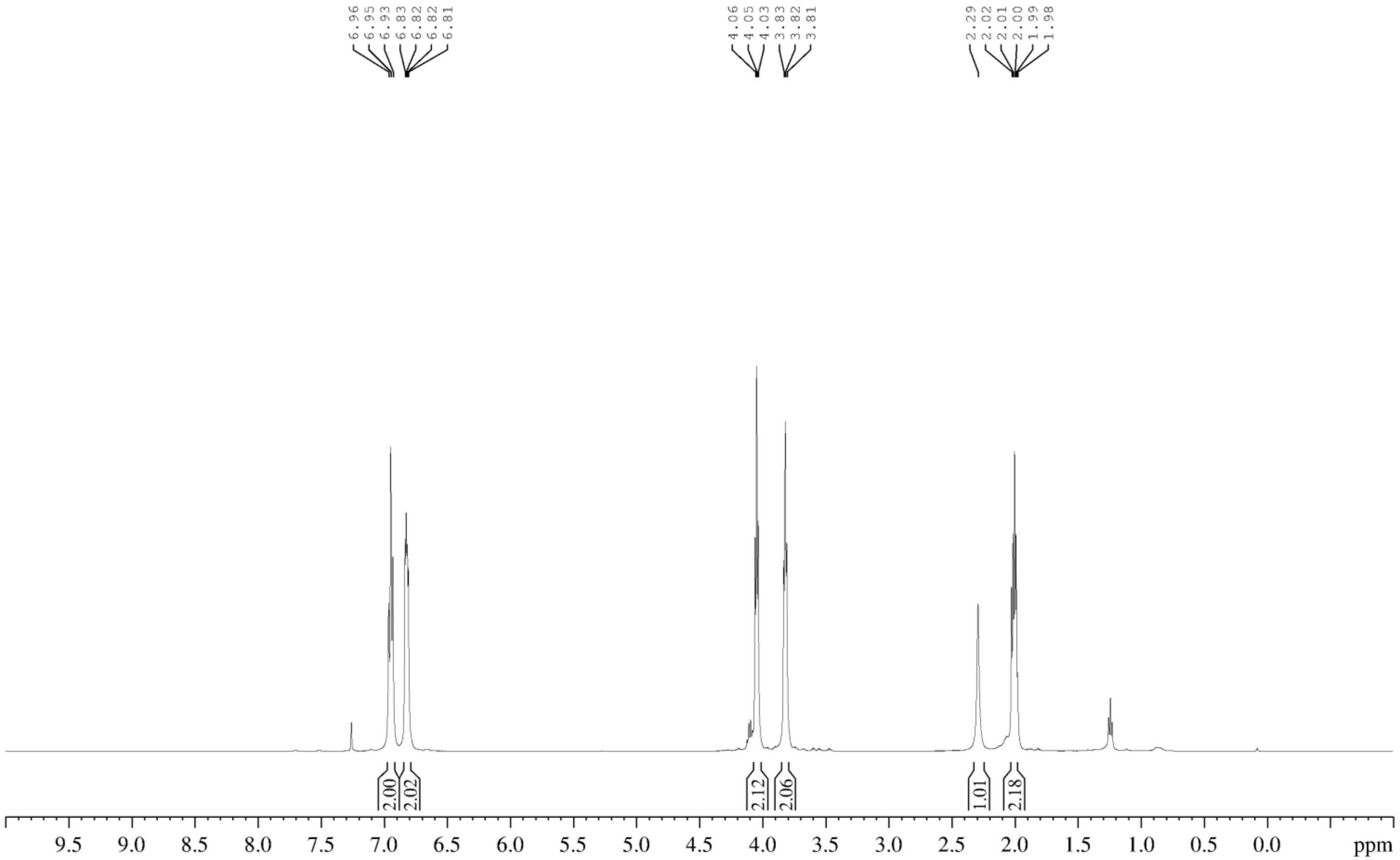

Figure S34. ^{13}C NMR (126 MHz, $CDCl_3$, 298 K) of **1t**.

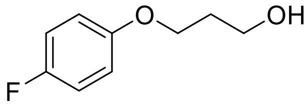

1t

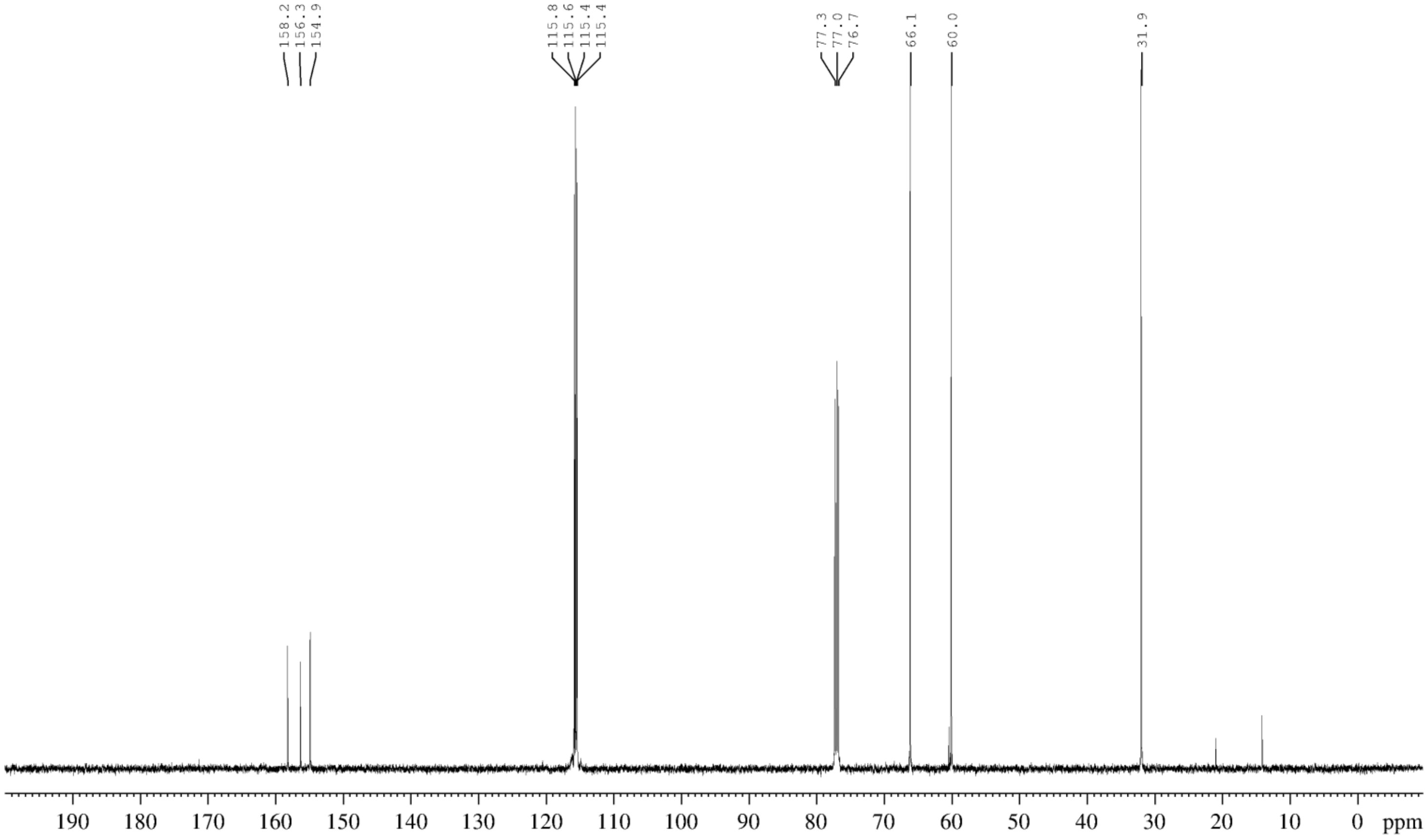

Figure S35. ^{19}F NMR (471 MHz, $CDCl_3$, 298 K) of **1t**.

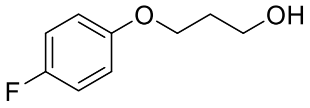

1t

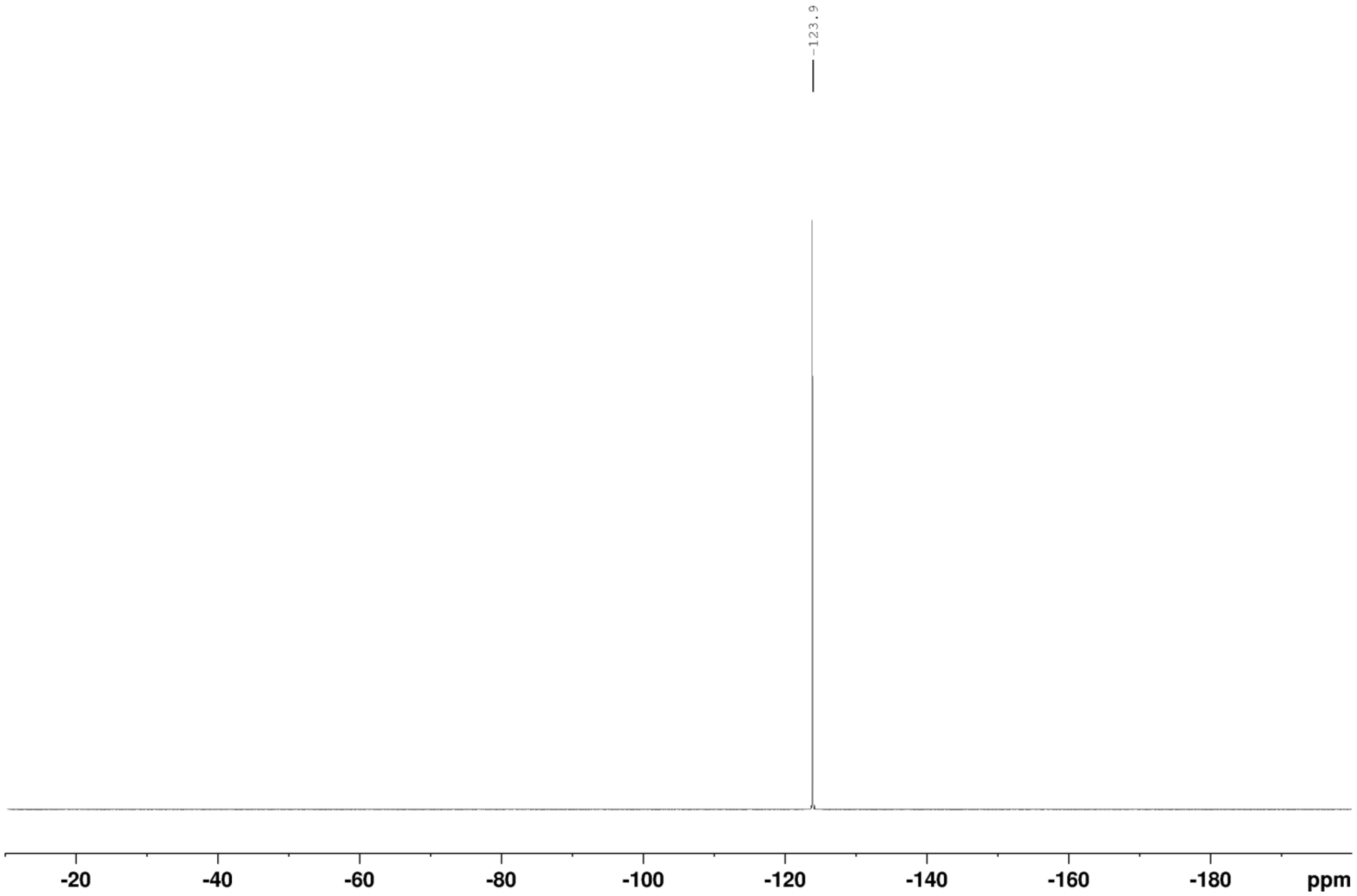

Figure S36. ^{1}H NMR (500 MHz, $CDCl_3$, 298 K) of **1u**.

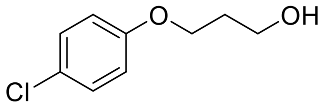

1u

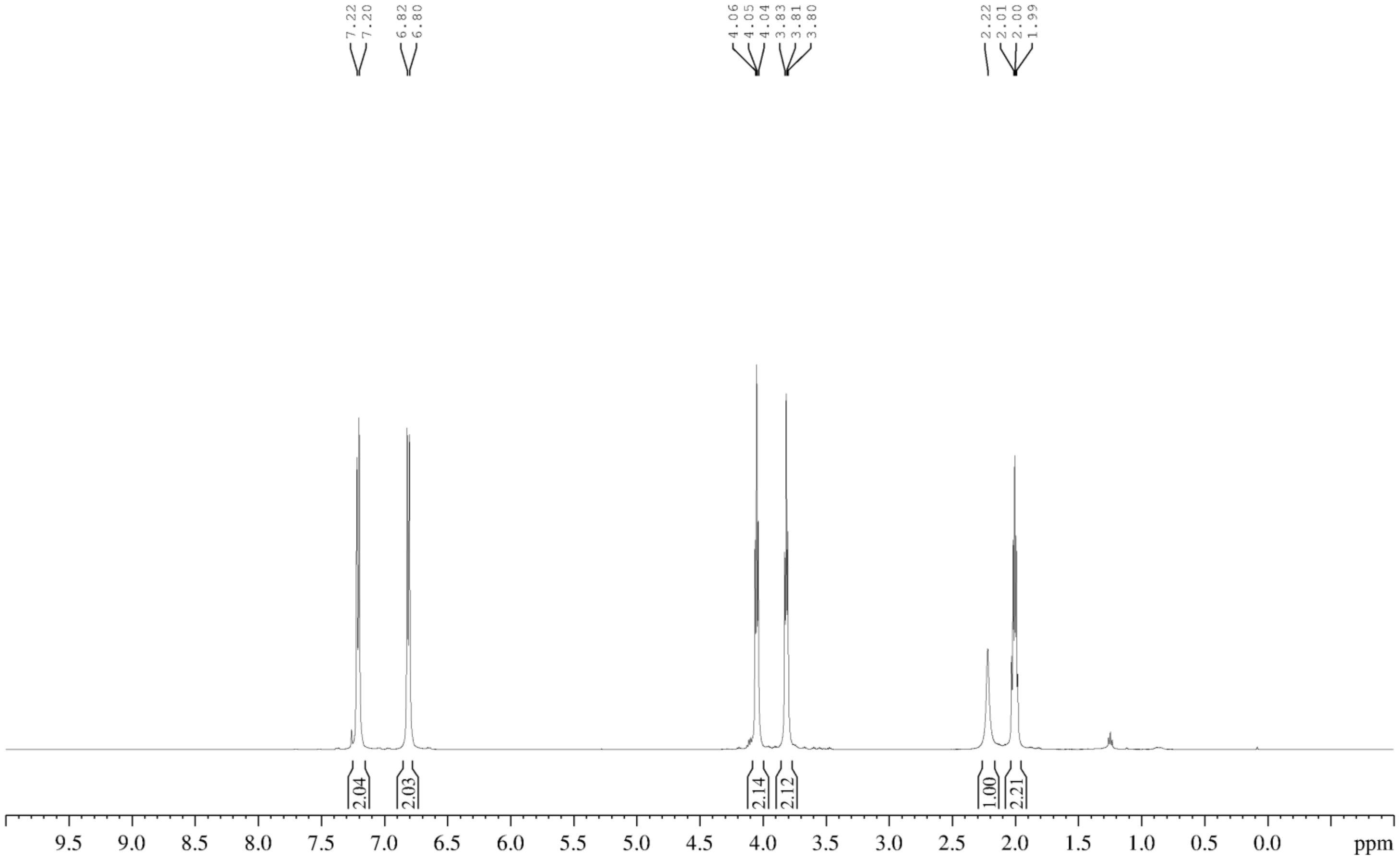

Figure S37. ^{13}C NMR (126 MHz, $CDCl_3$, 298 K) of **1u**.

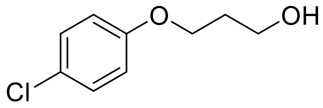

1u

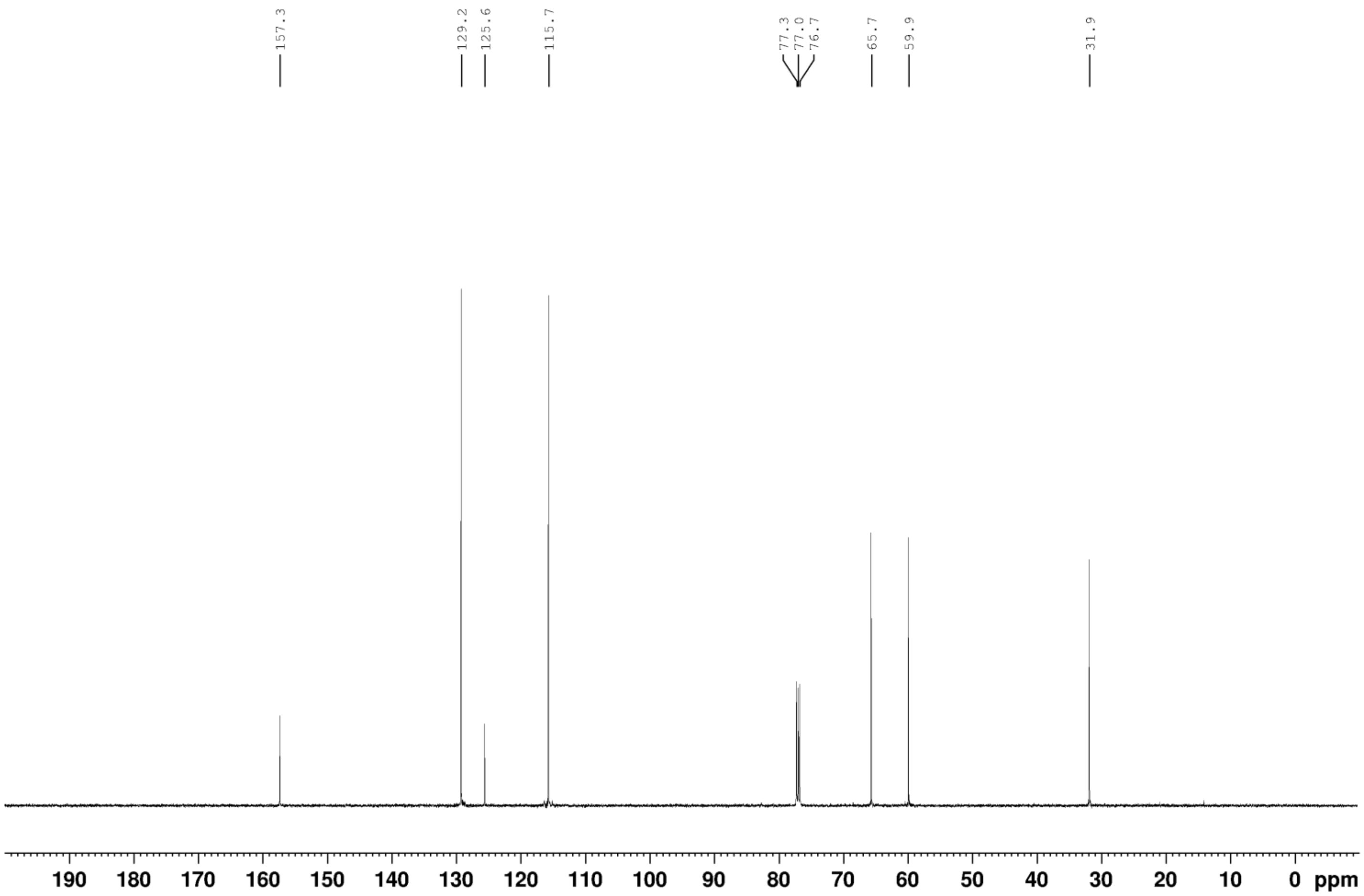

Figure S38. ^{1}H NMR (500 MHz, $CDCl_3$, 298 K) of **1v**.

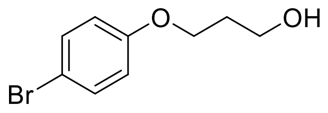

1v

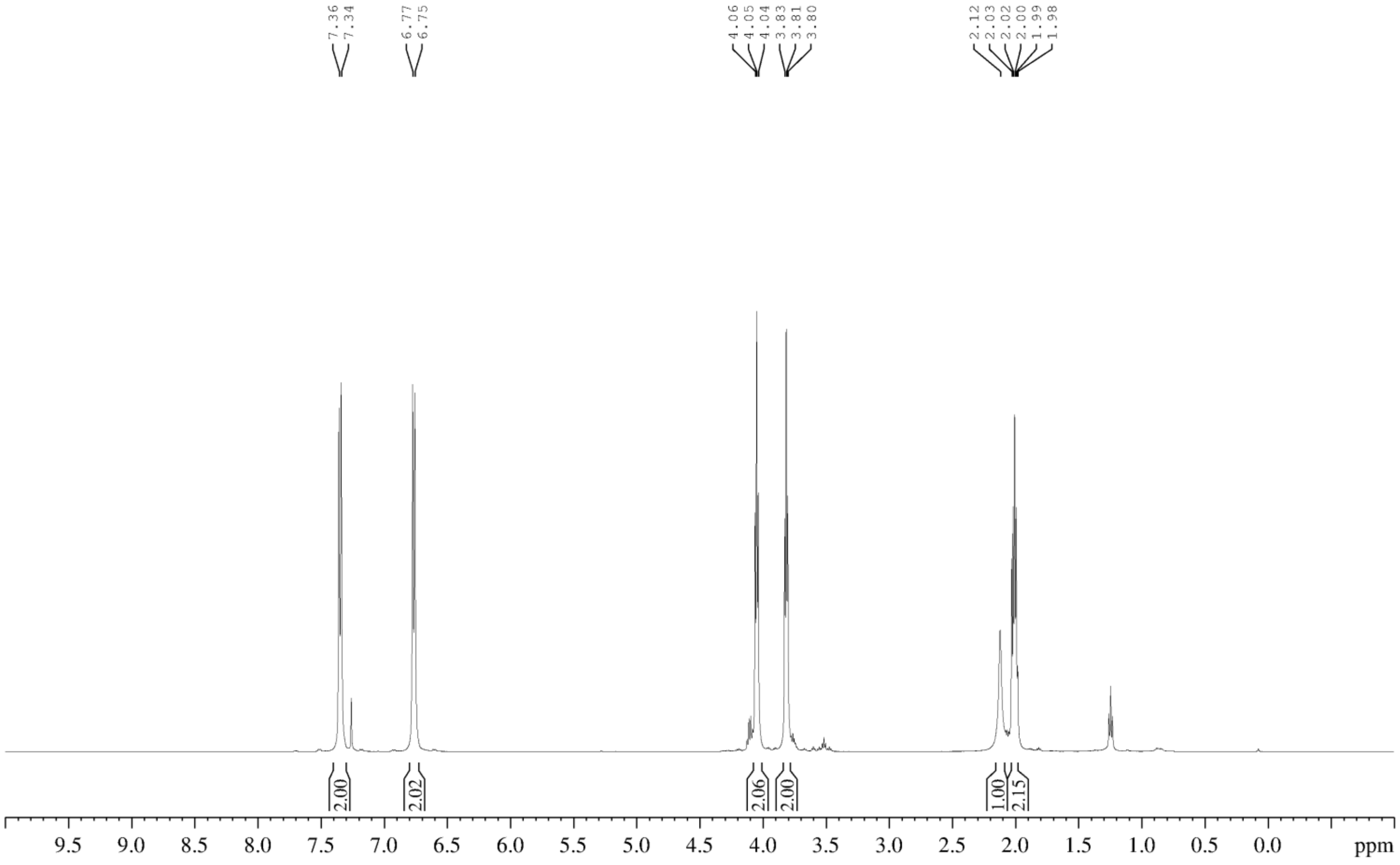

Figure S39. ^{13}C NMR (126 MHz, $CDCl_3$, 298 K) of **1v**.

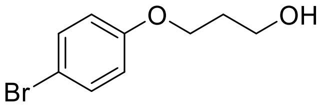

1v

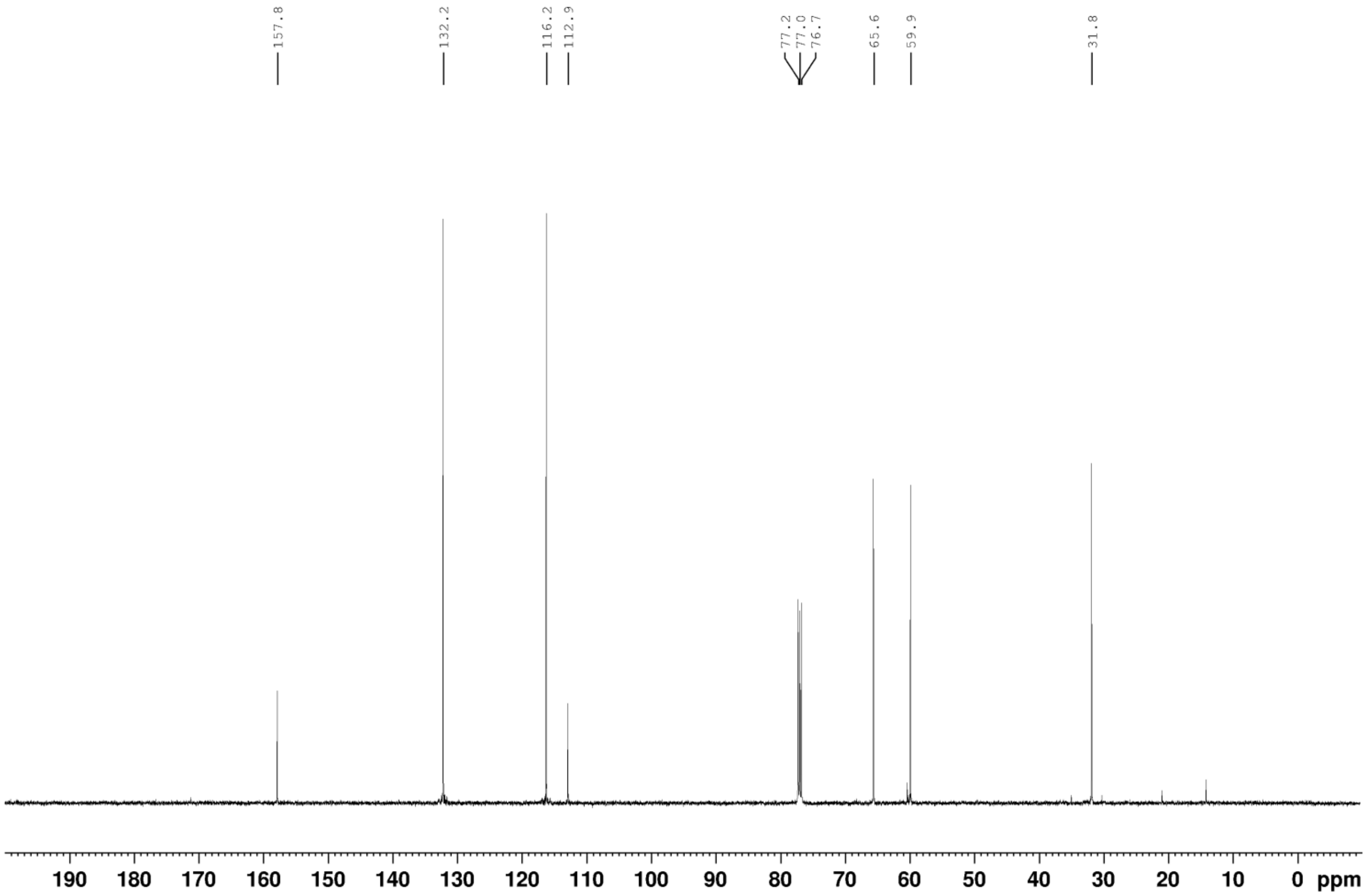

Figure S40. ^{1}H NMR (500 MHz, $CDCl_3$, 298 K) of **1w**.

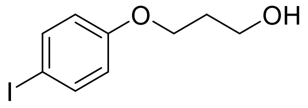

1w

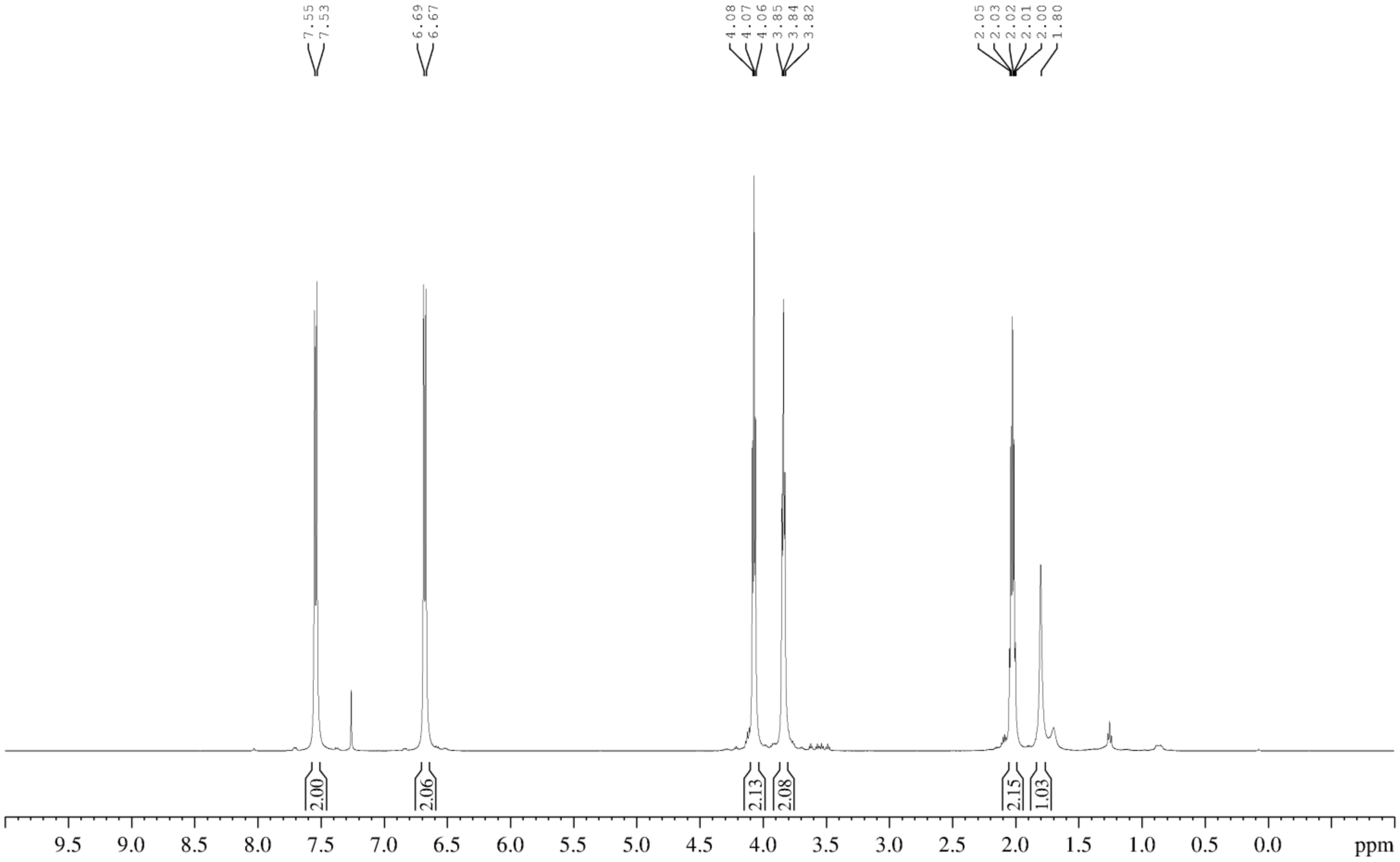

Figure S41. ^{13}C NMR (126 MHz, $CDCl_3$, 298 K) of **1w**.

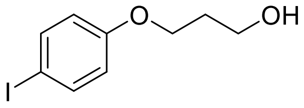

1w

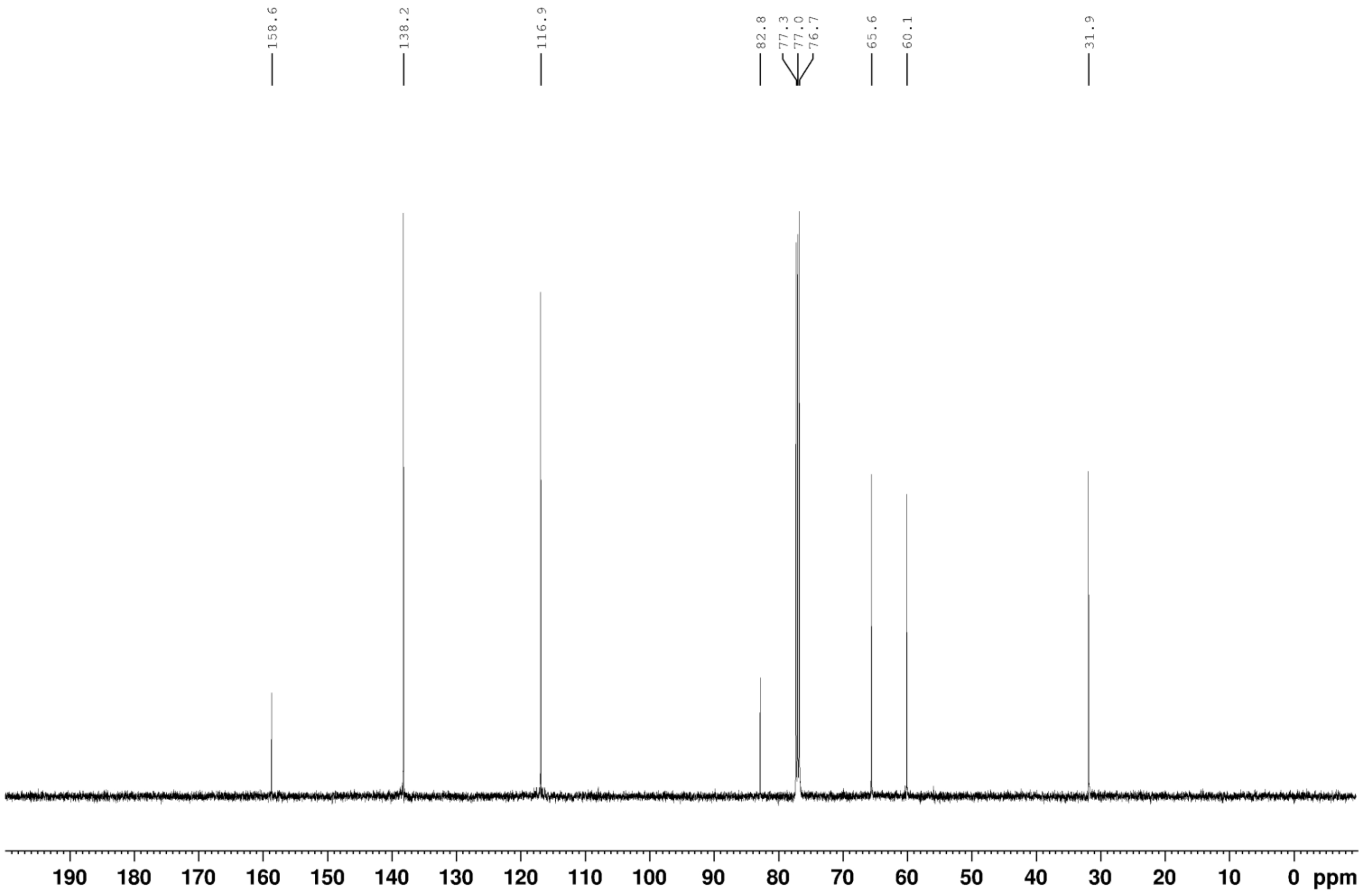

Figure S42. ^{1}H NMR (500 MHz, $CDCl_3$, 298 K) of **1x**.

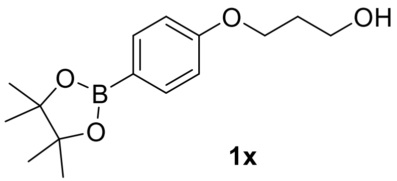

1x

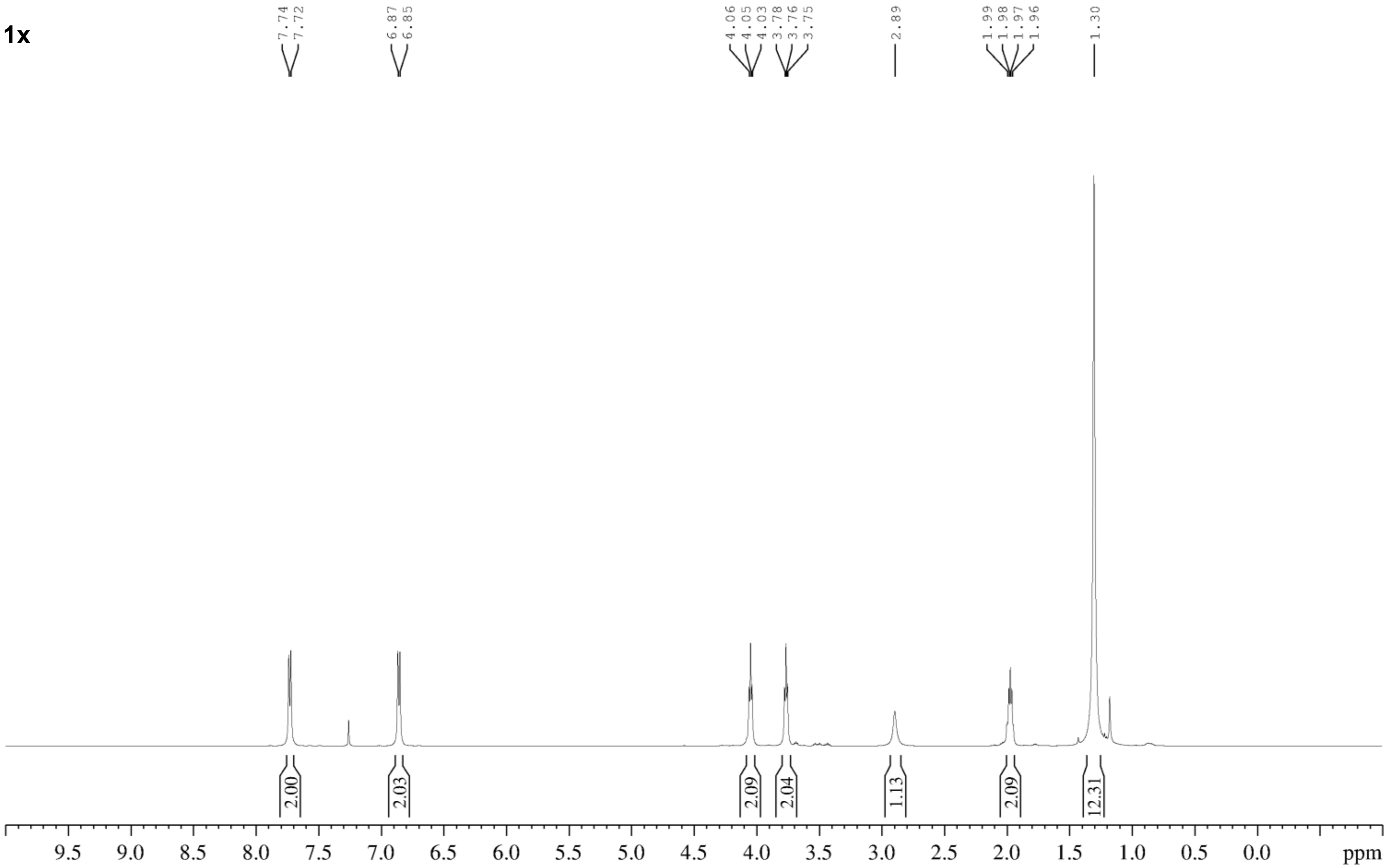

Figure S43. ^{13}C NMR (126 MHz, $CDCl_3$, 298 K) of **1x**.

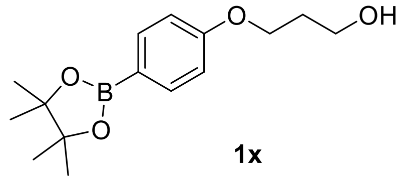

1x

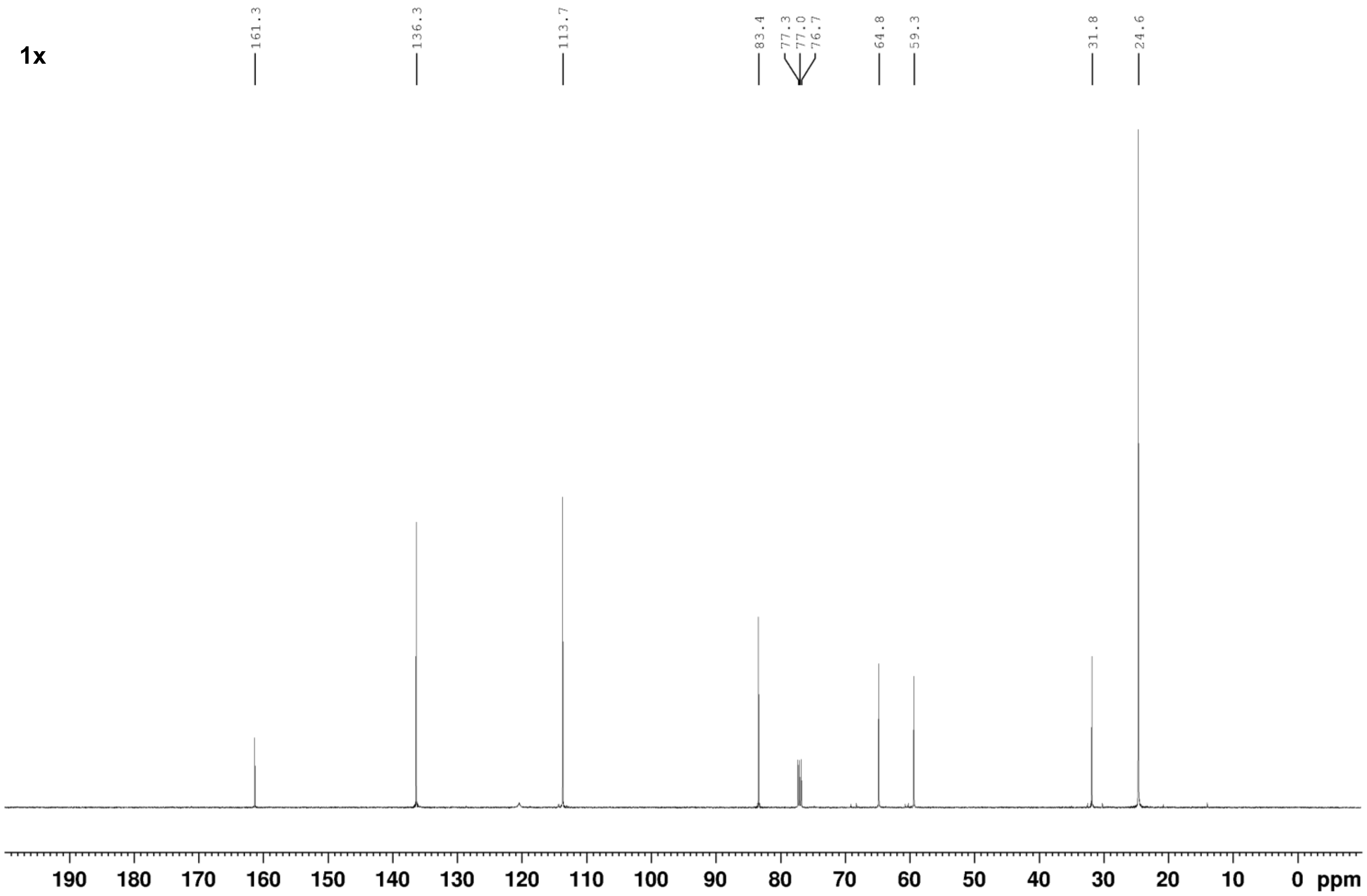

Figure S44. ^{1}H NMR (500 MHz, $CDCl_3$, 298 K) of **1z**.

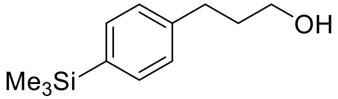

1z

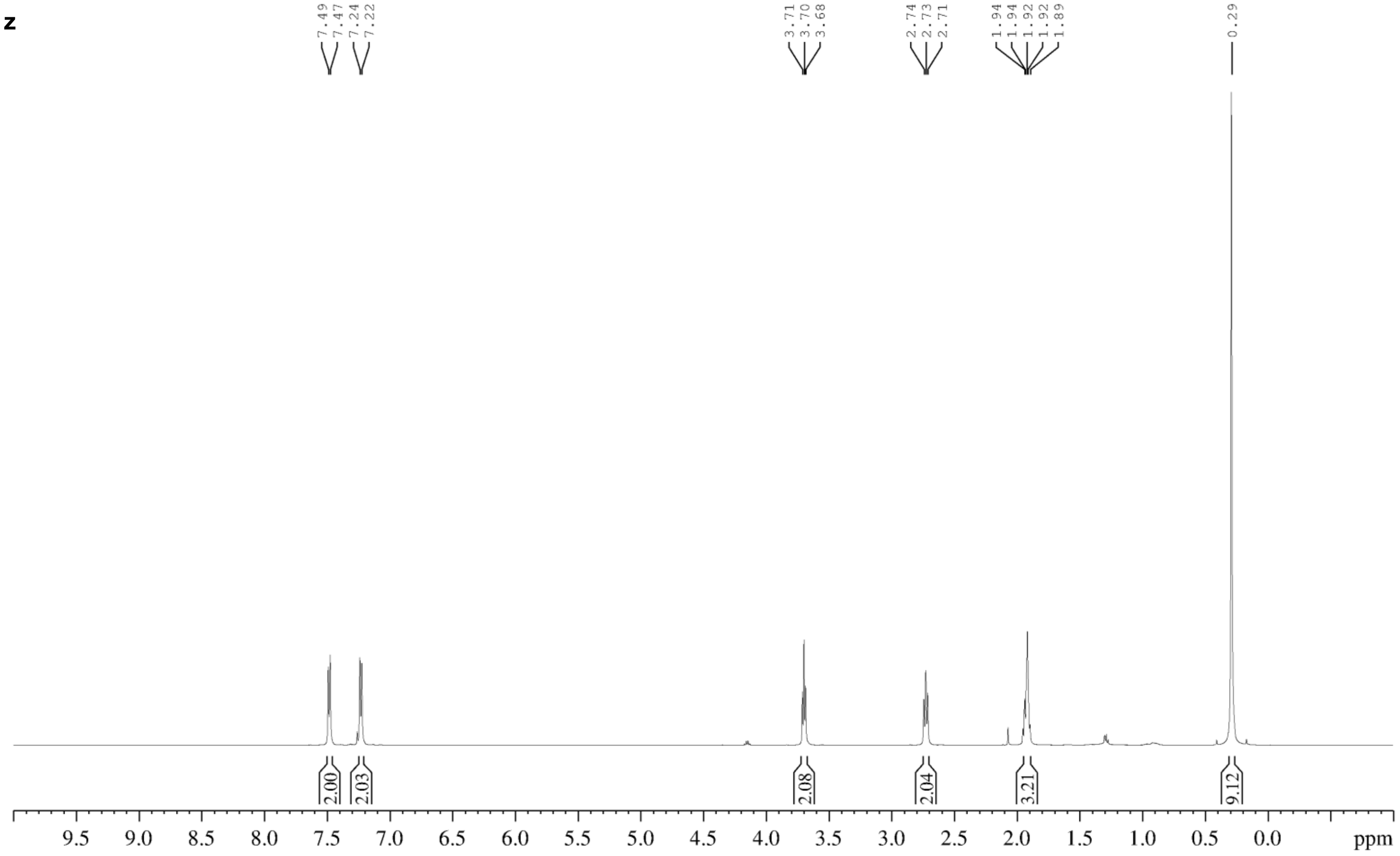

Figure S45. ^{13}C NMR (126 MHz, $CDCl_3$, 298 K) of **1z**.

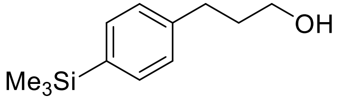

1z

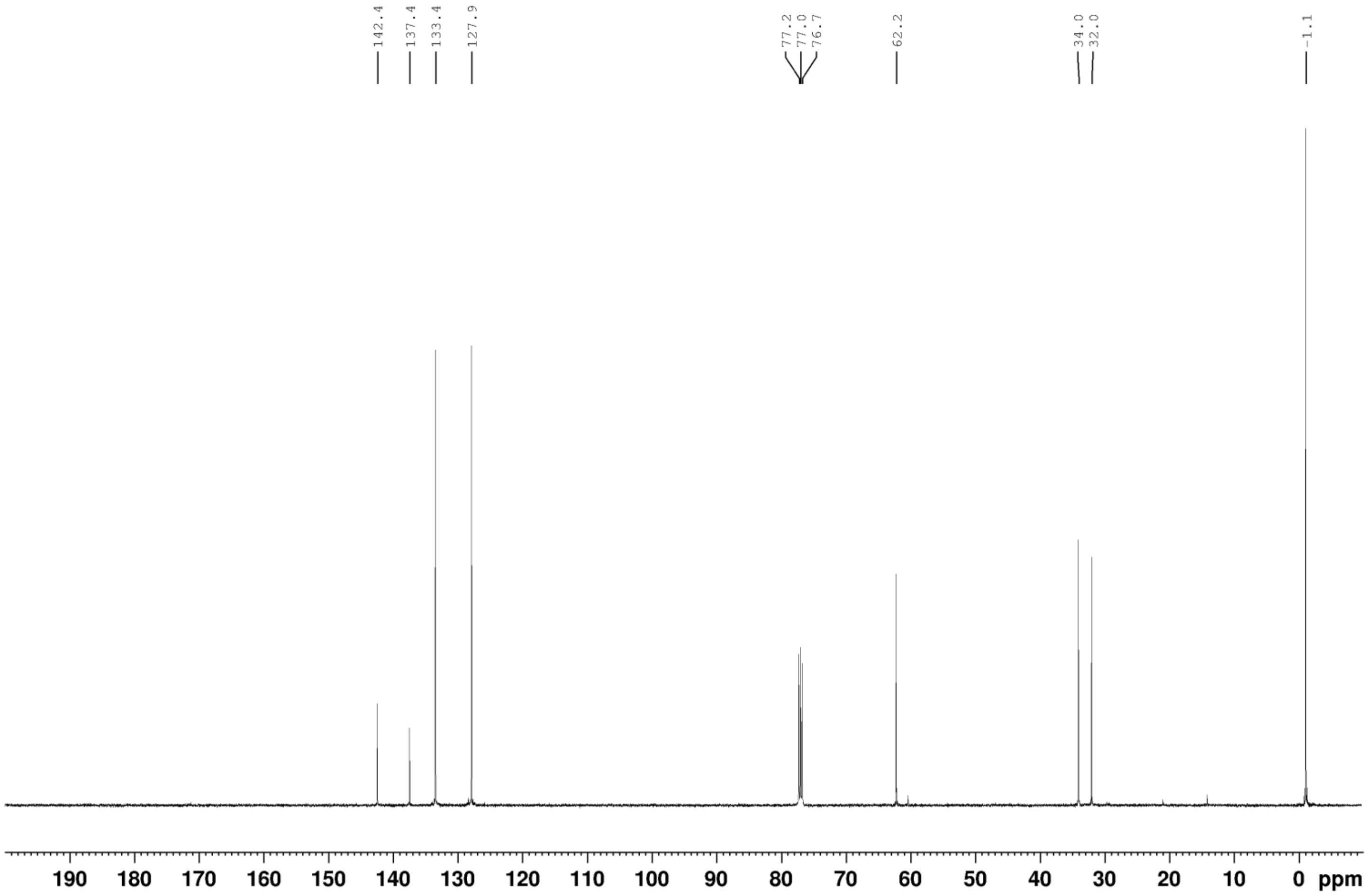

Figure S46. $^{1}H/^{29}Si$ HMQC NMR (500/99 MHz, $CDCl_3$, 298 K, optimized for J = 7 Hz) of **1z**.

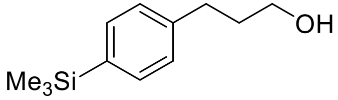

1z

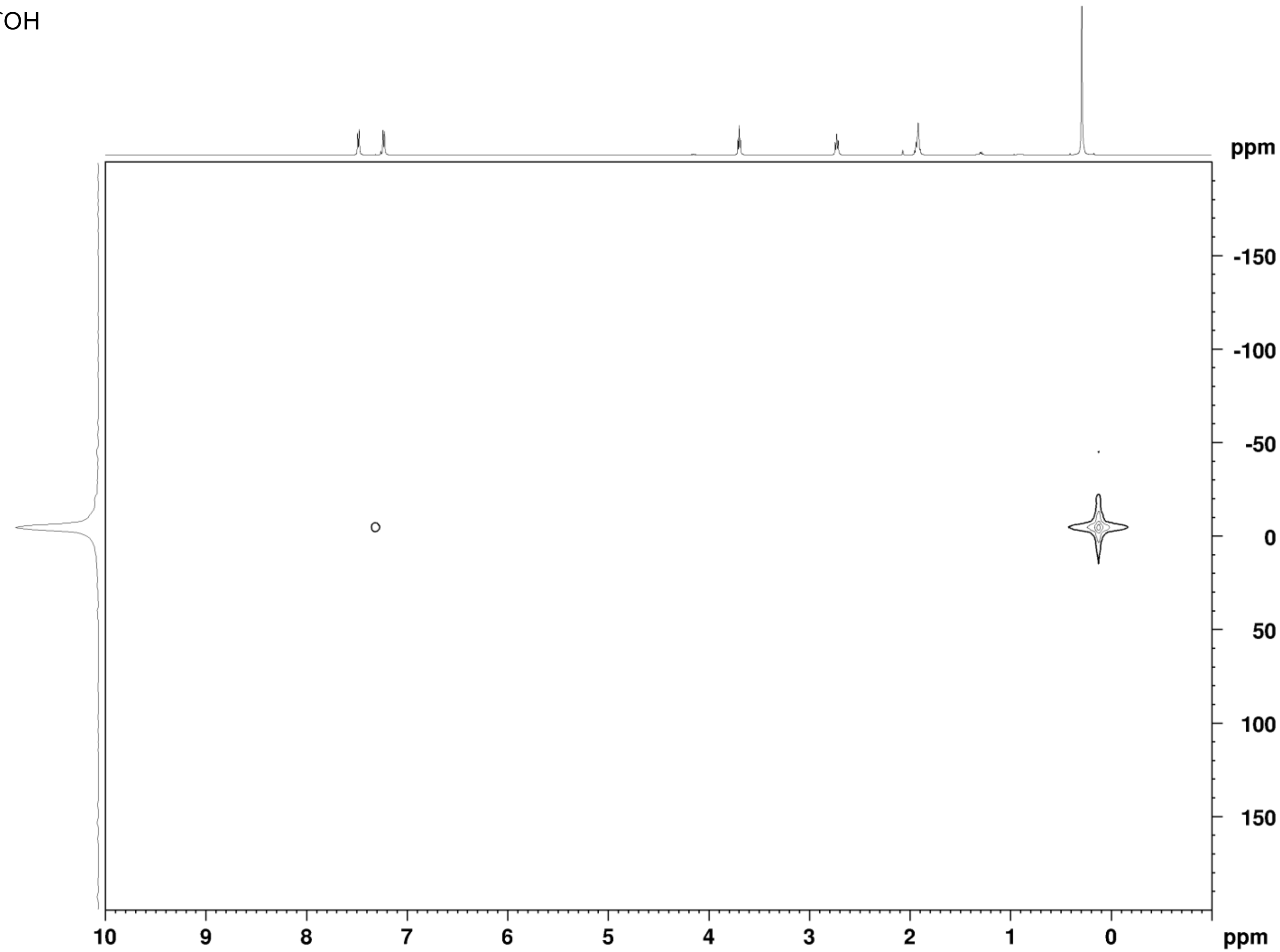

Figure S47. ^1H NMR (500 MHz, $CDCl_3$, 298 K) of **4a**.

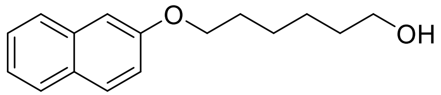

4a

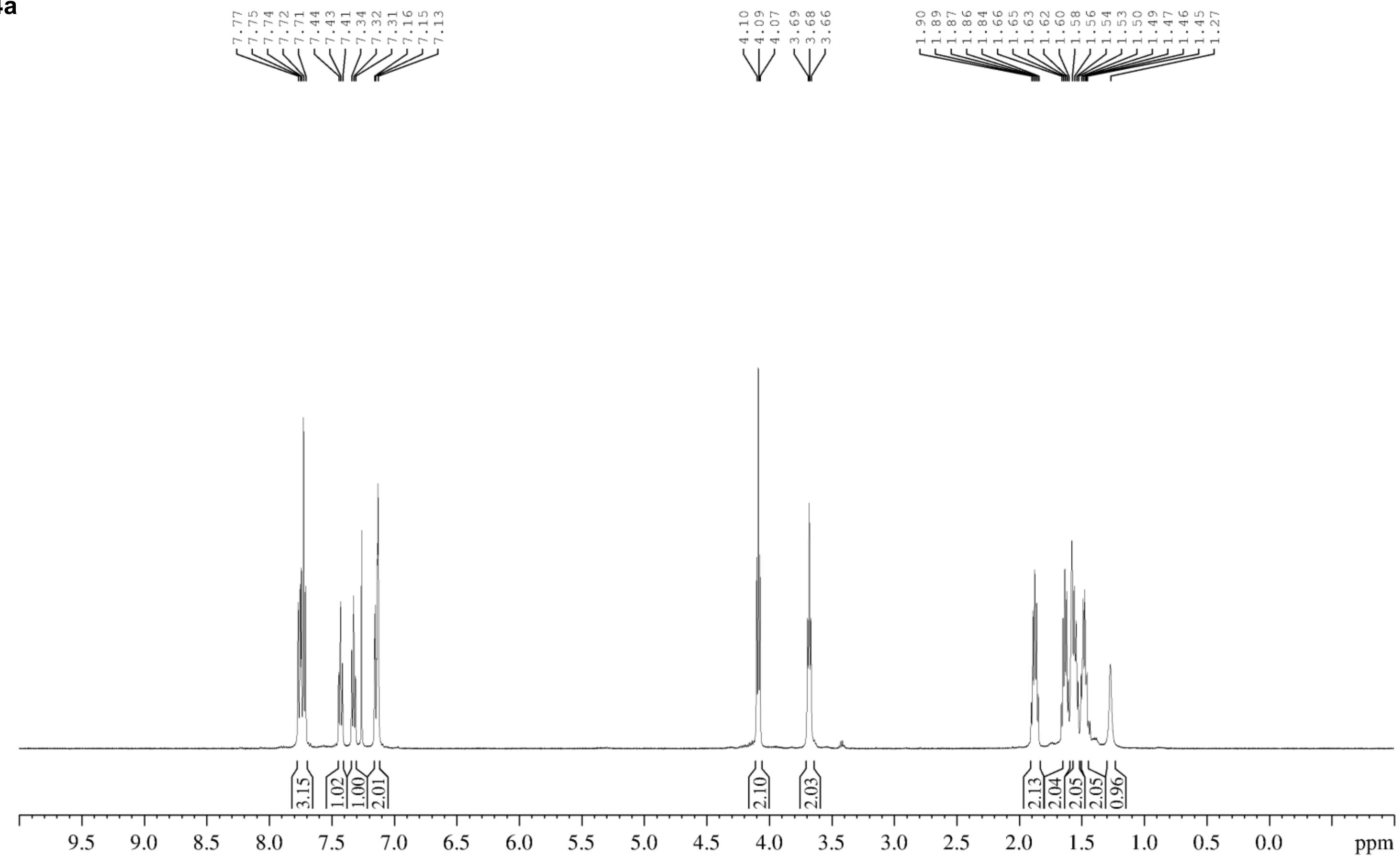

Figure S48. ^{13}C NMR (126 MHz, $CDCl_3$, 298 K) of **4a**.

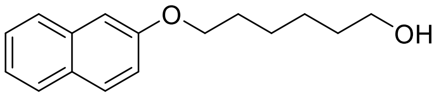

4a

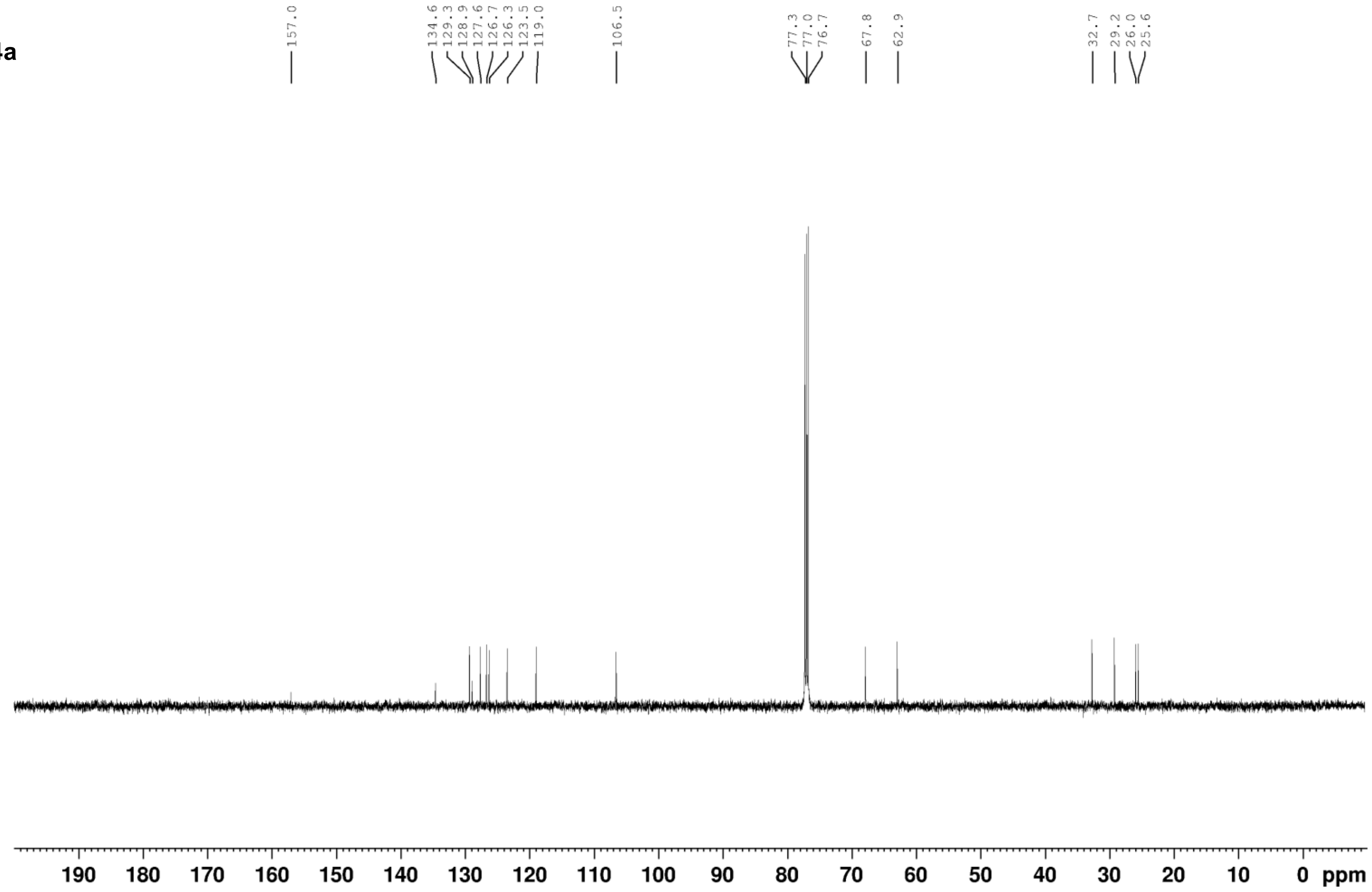

Figure S49. ^{1}H NMR (500 MHz, $CDCl_3$, 298 K) of **4b**.

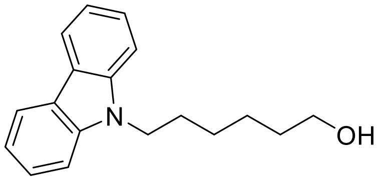

4b

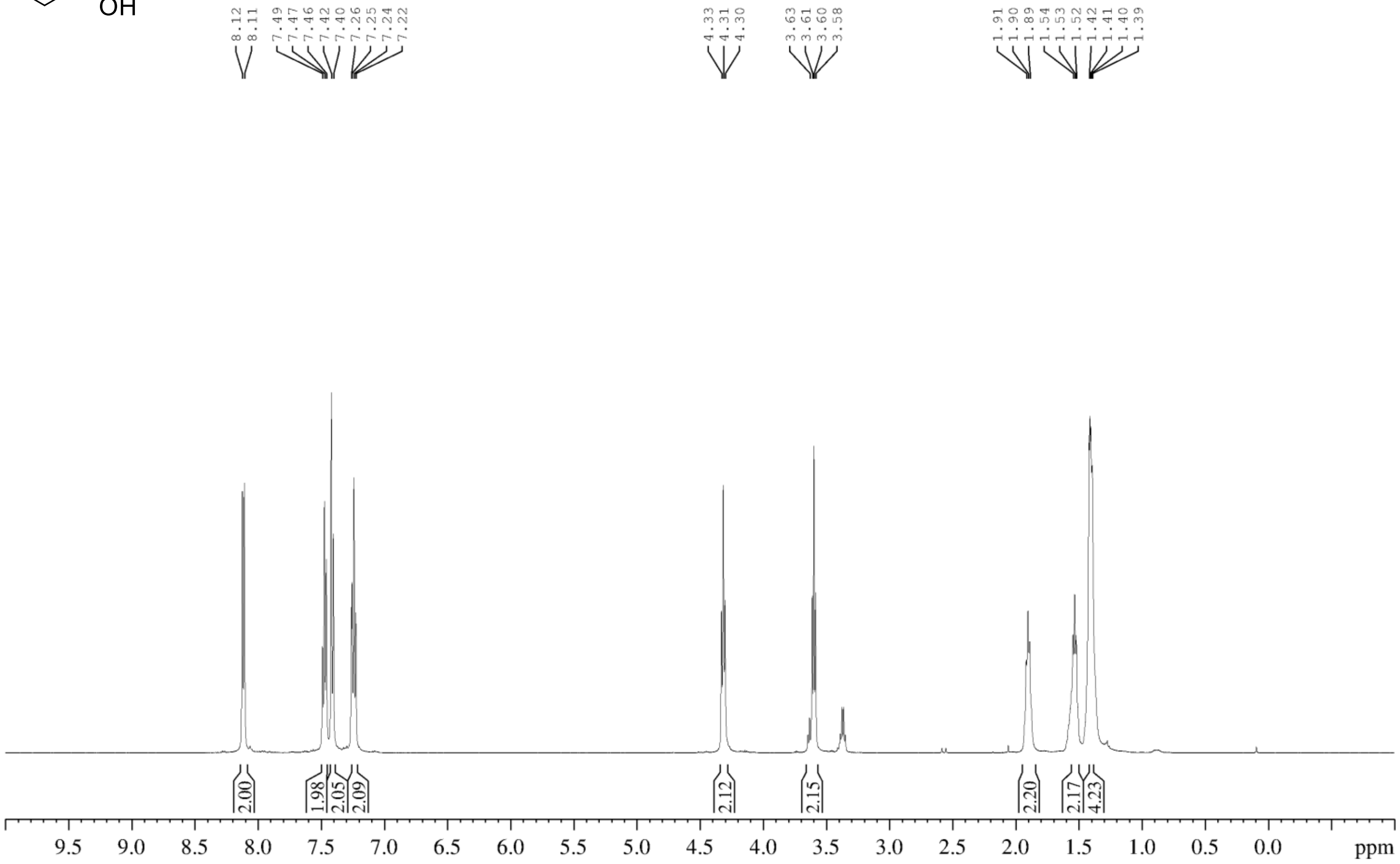

Figure S50. ^{13}C NMR (126 MHz, $CDCl_3$, 298 K) of **4b**.

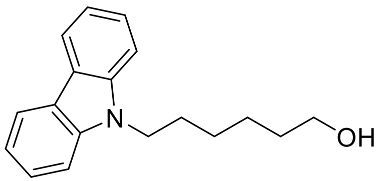

4b

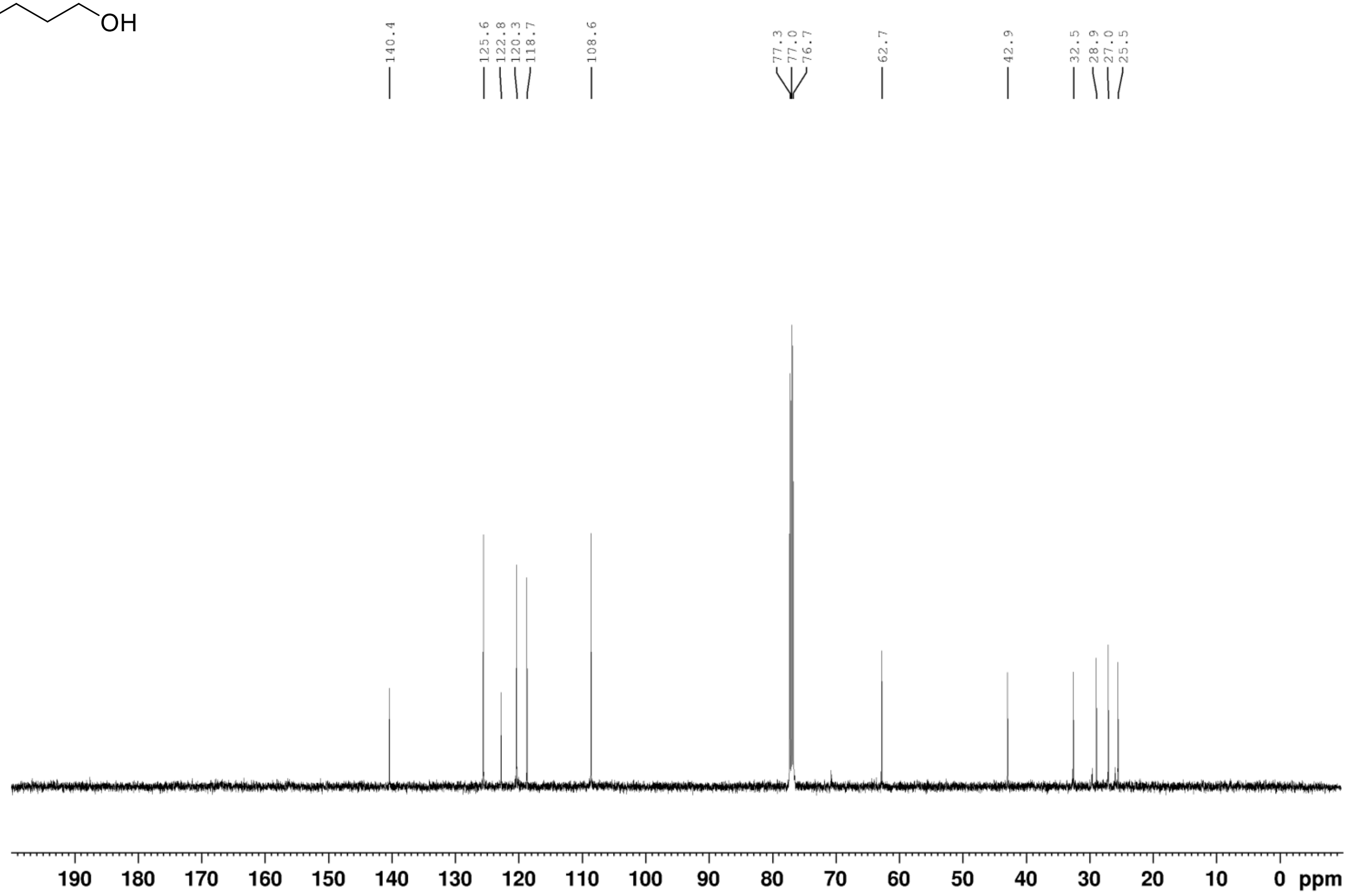

Figure S51. ^{1}H NMR (500 MHz, $CDCl_3$, 298 K) of **4c**.

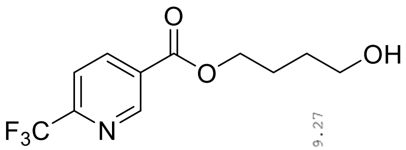

4c

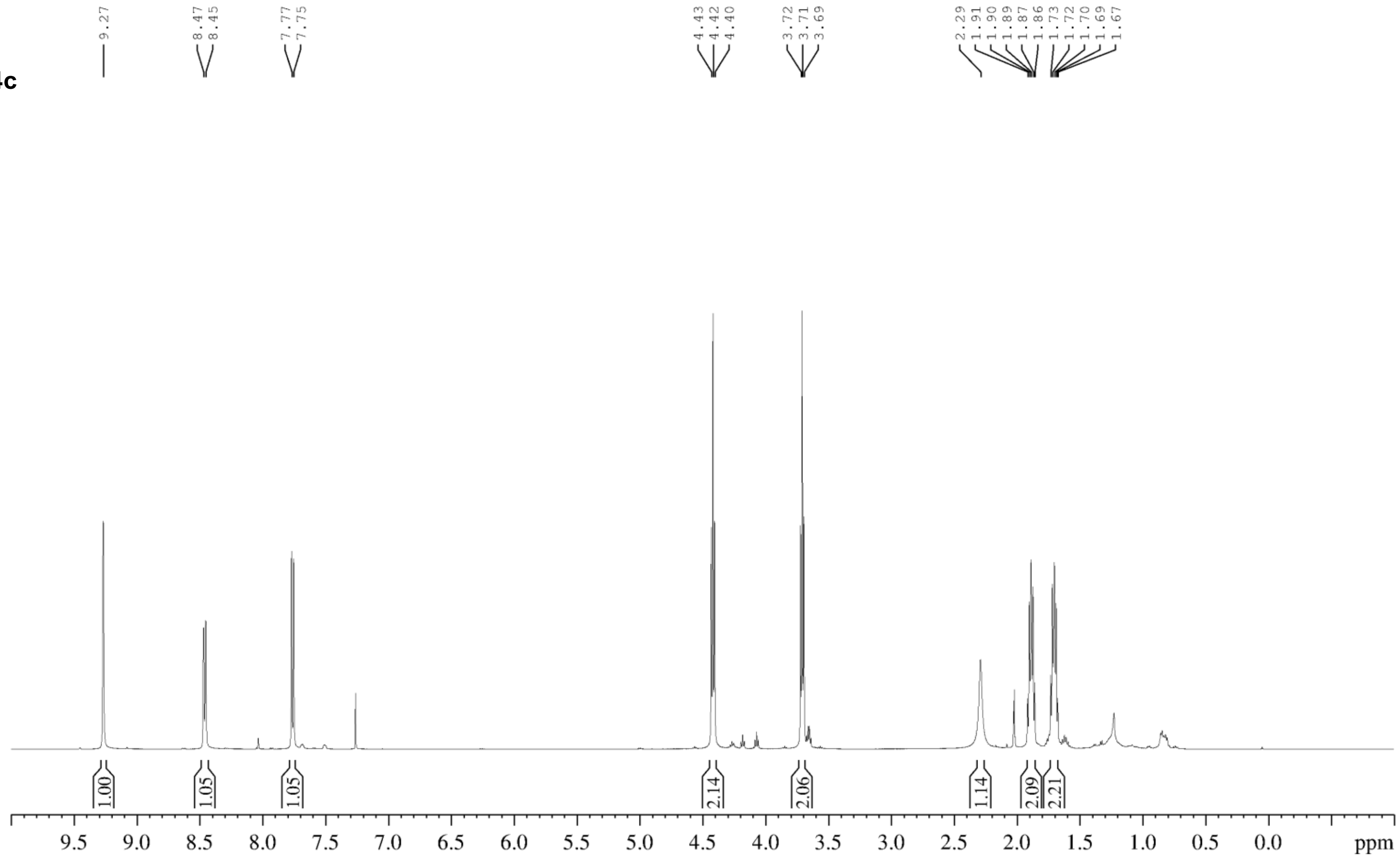

Figure S52. ^{13}C NMR (126 MHz, $CDCl_3$, 298 K) of **4c**.

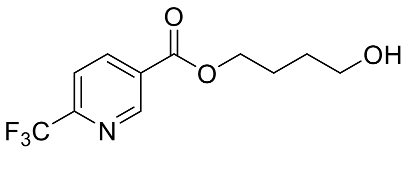

4c

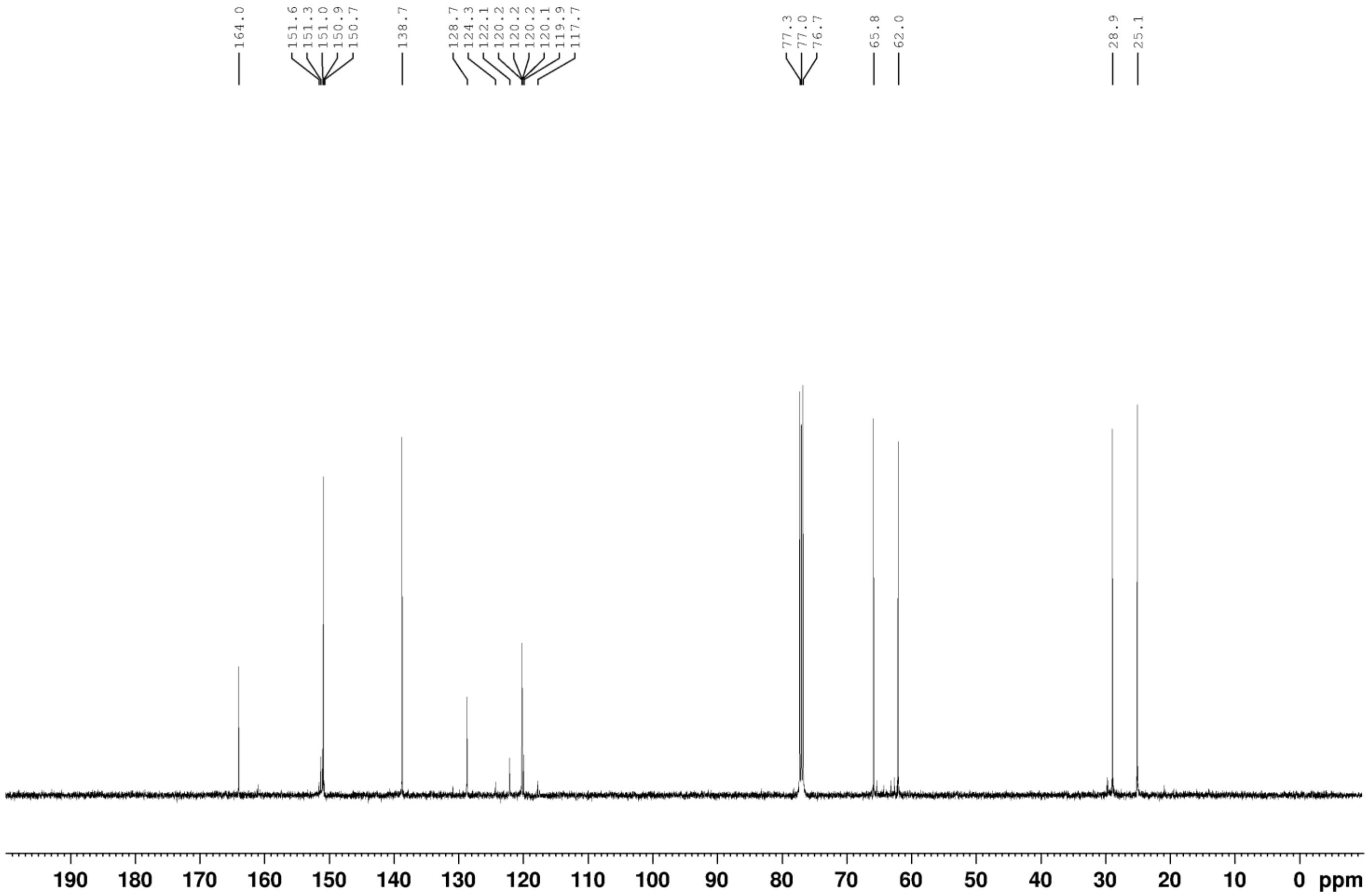

Figure S53. ^{19}F NMR (471 MHz, $CDCl_3$, 298 K) of **4c**.

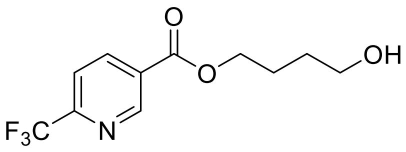

4c

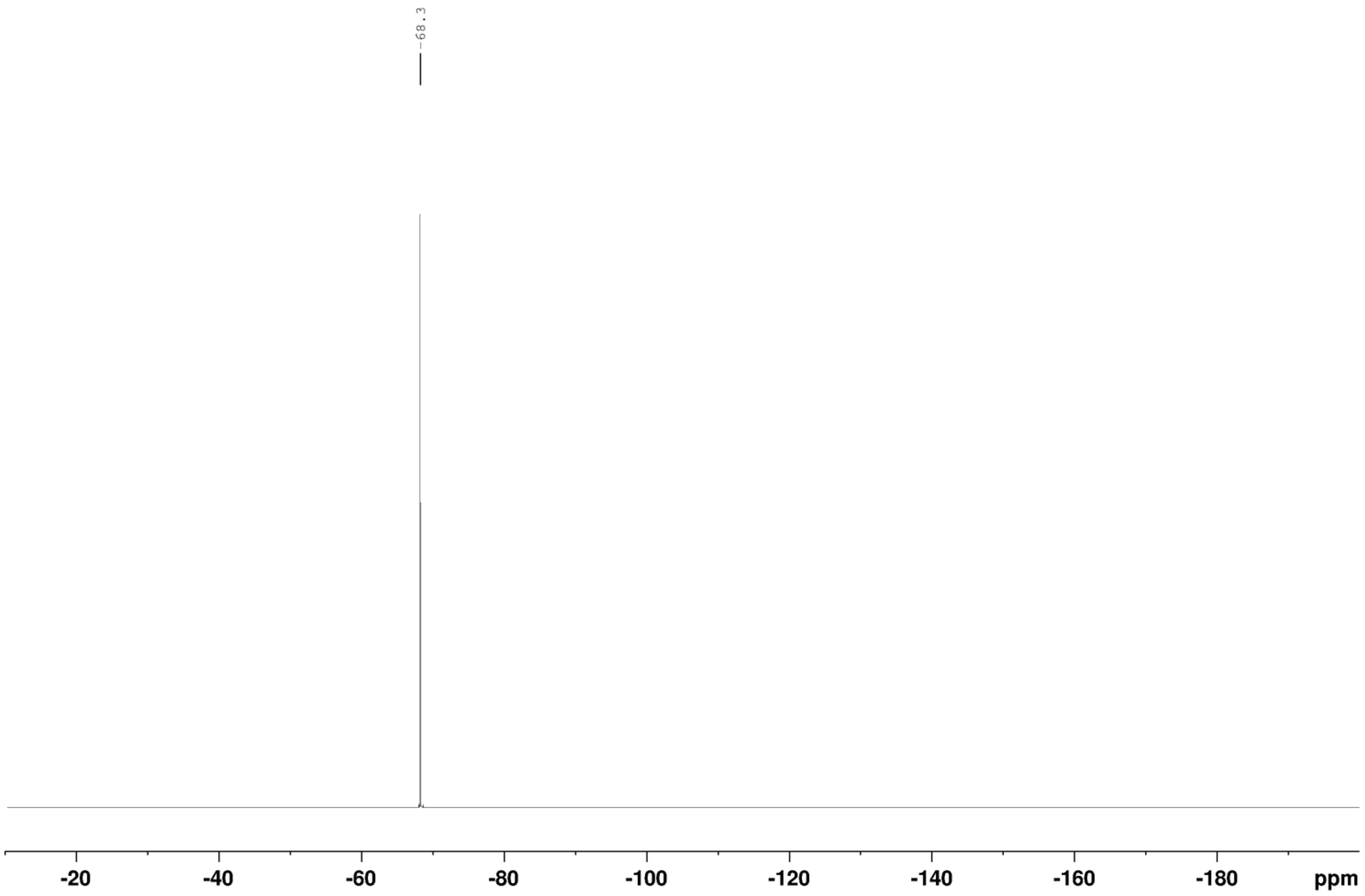

Figure S54. ^{1}H NMR (500 MHz, $CDCl_3$, 298 K) of **4d**.

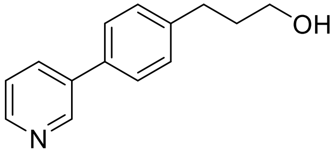

4d

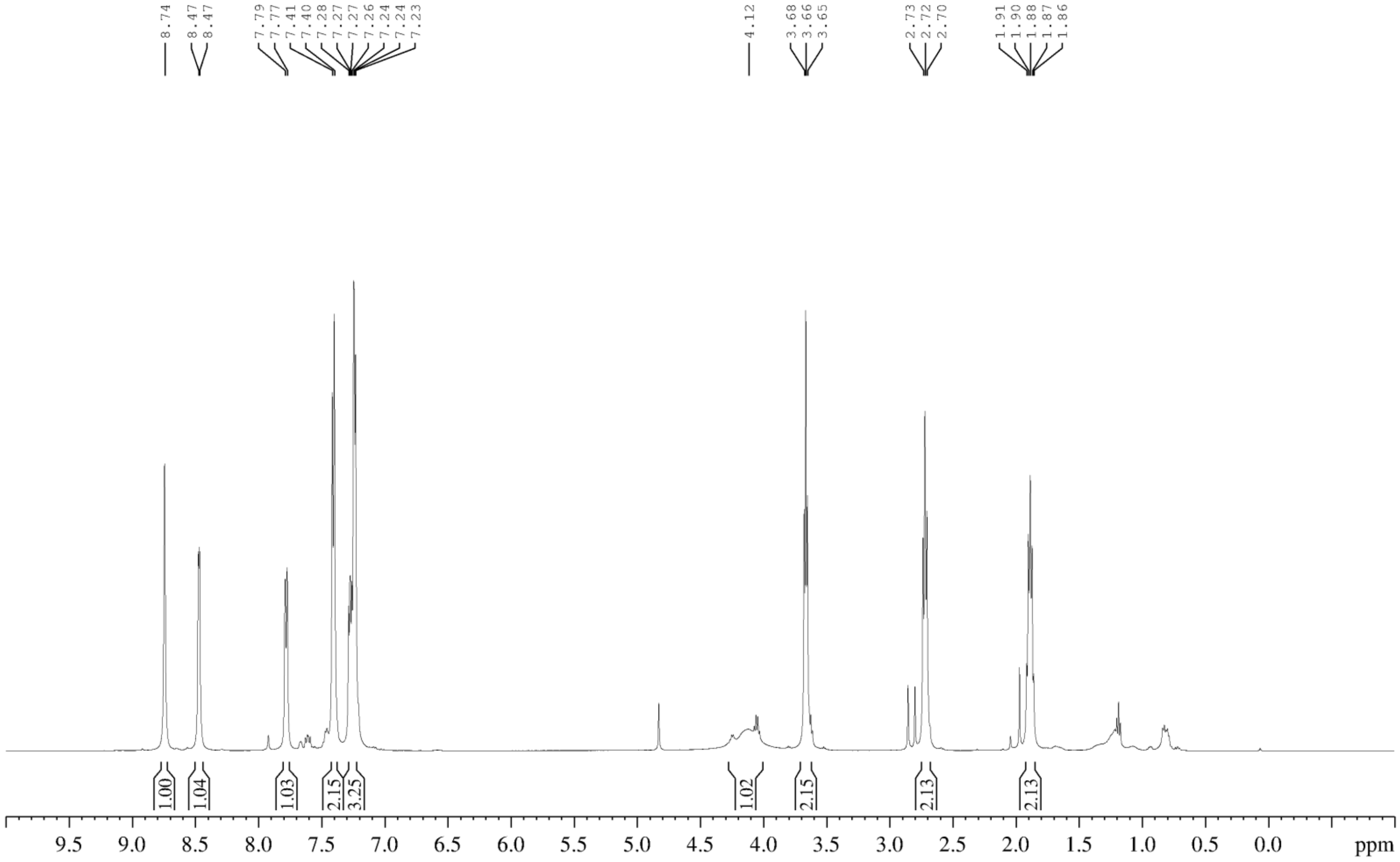

Figure S55. ^{13}C NMR (126 MHz, $CDCl_3$, 298 K) of **4d**.

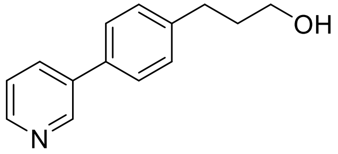

4d

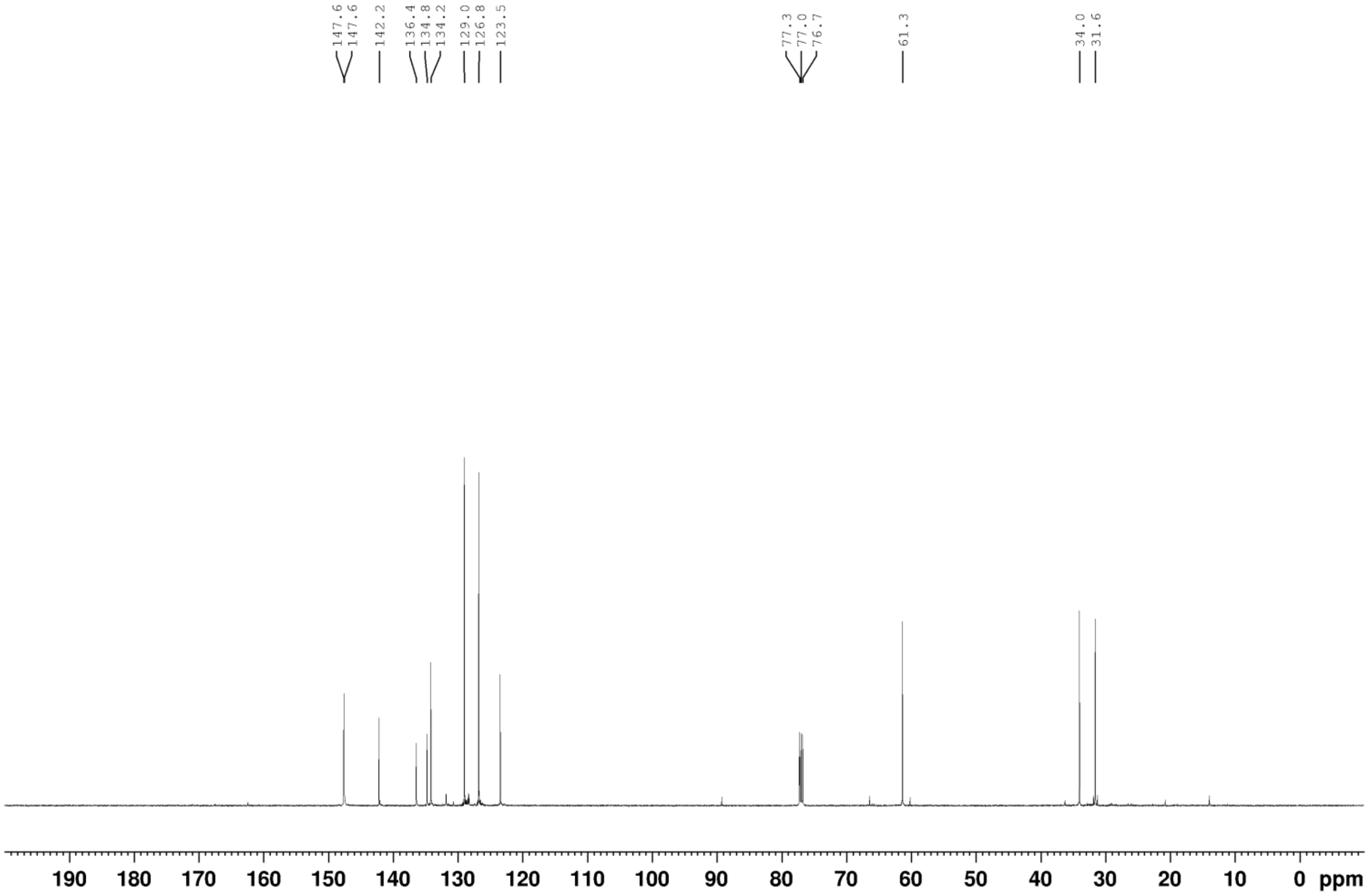

Figure S56. ^{1}H NMR (500 MHz, $CDCl_3$, 298 K) of **4e**.

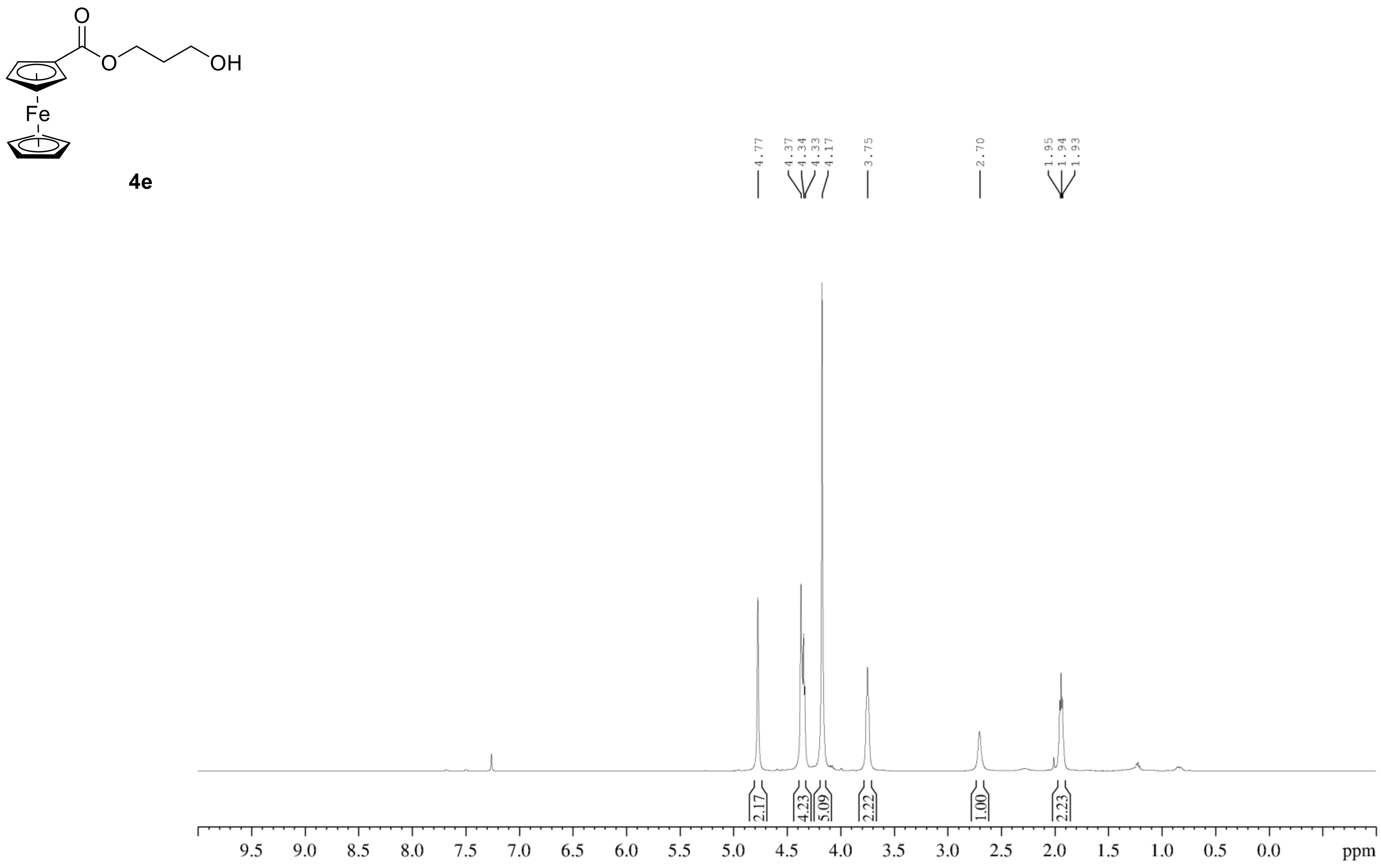

Figure S57. ^{13}C NMR (126 MHz, $CDCl_3$, 298 K) of **4e**.

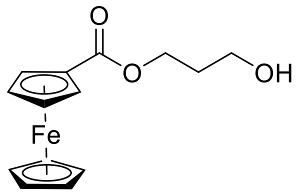

4e

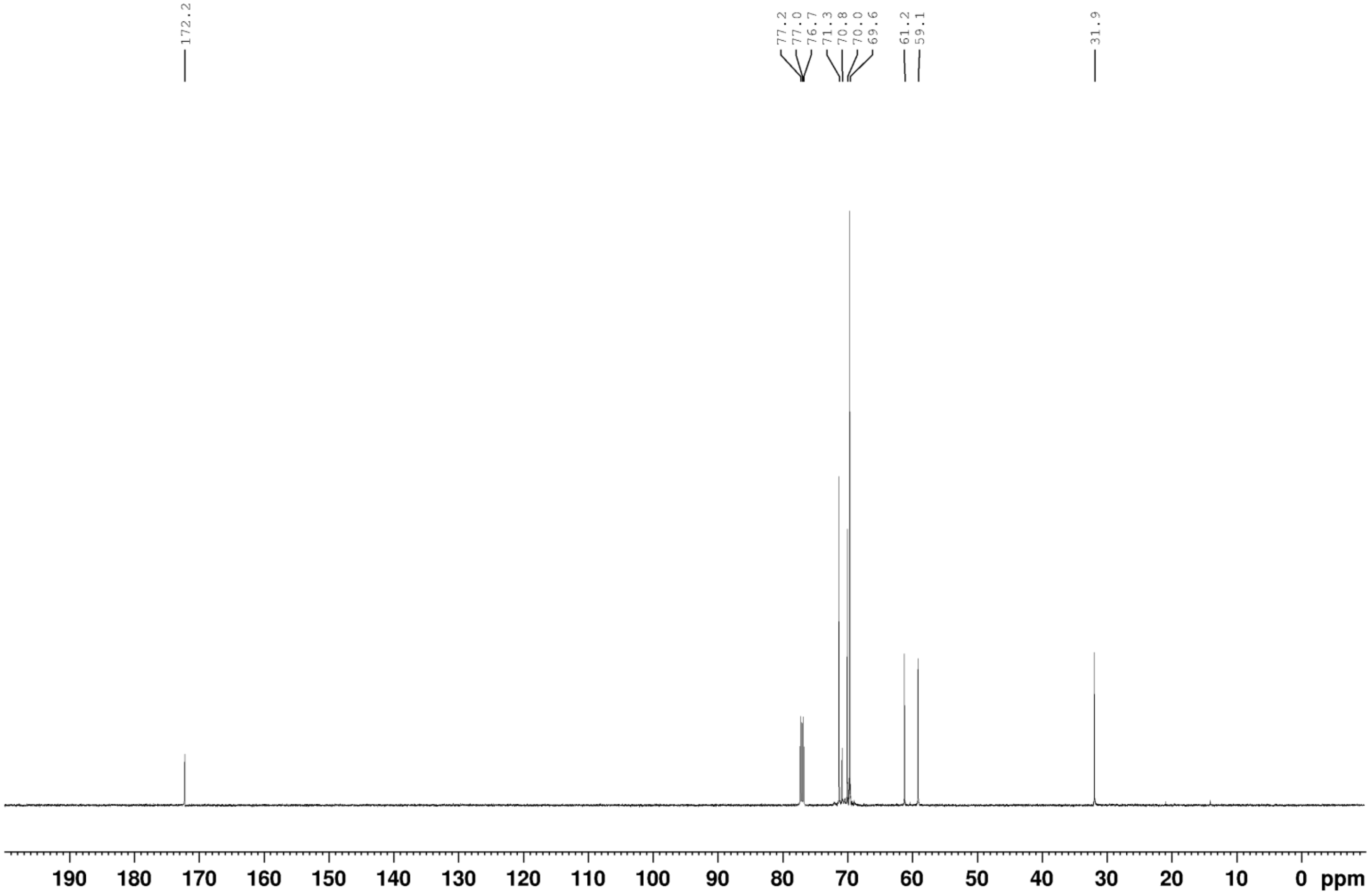

Figure S58. ^{1}H NMR (500 MHz, $CDCl_3$, 298 K) of **6b**.

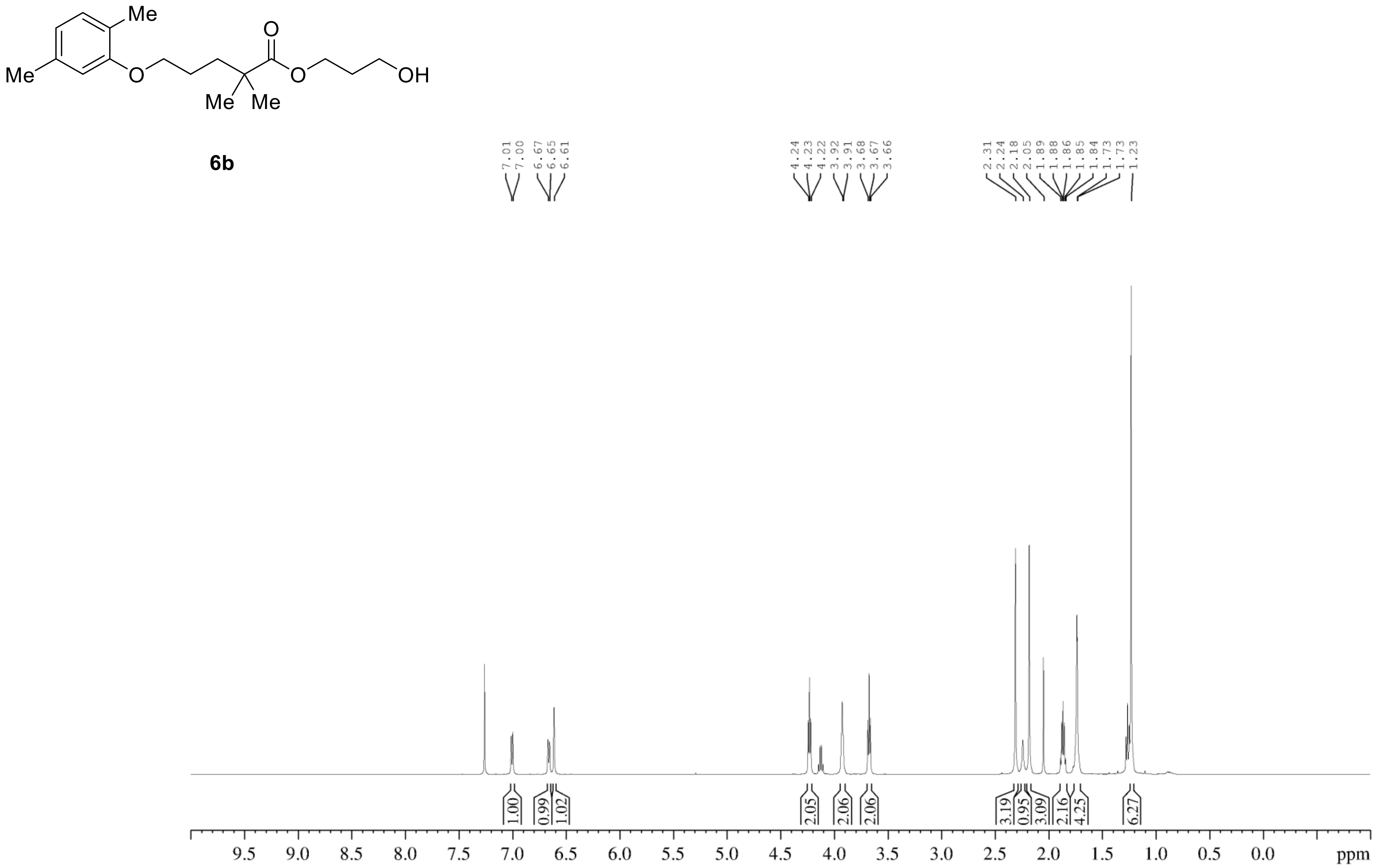

6b

Figure S59. ^{13}C NMR (126 MHz, $CDCl_3$, 298 K) of **6b**.

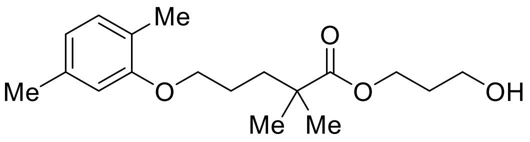

6b

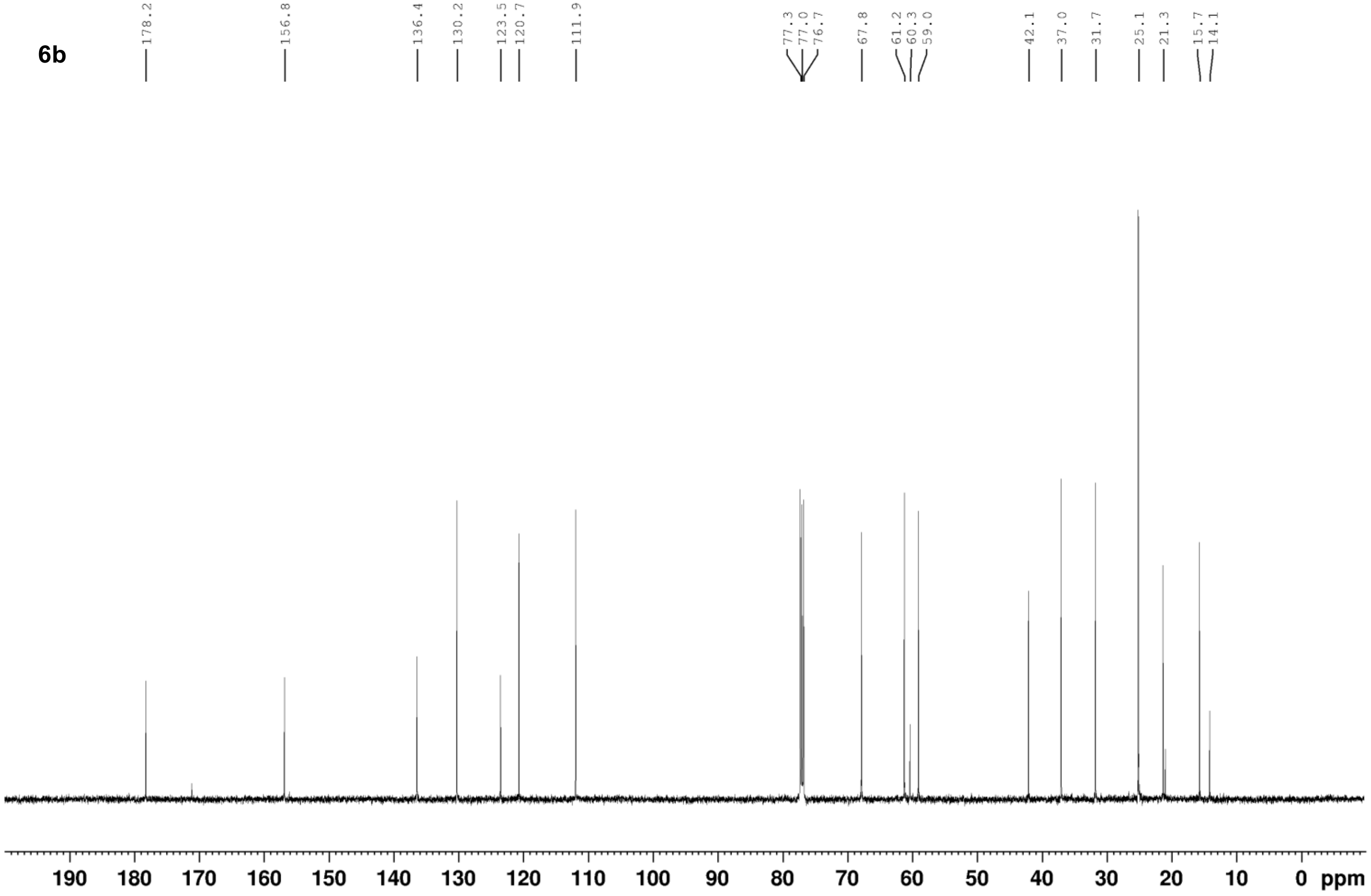

Figure S60. ^{1}H NMR (500 MHz, $CDCl_3$, 298 K) of **6c**.

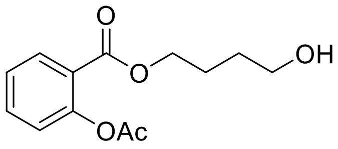

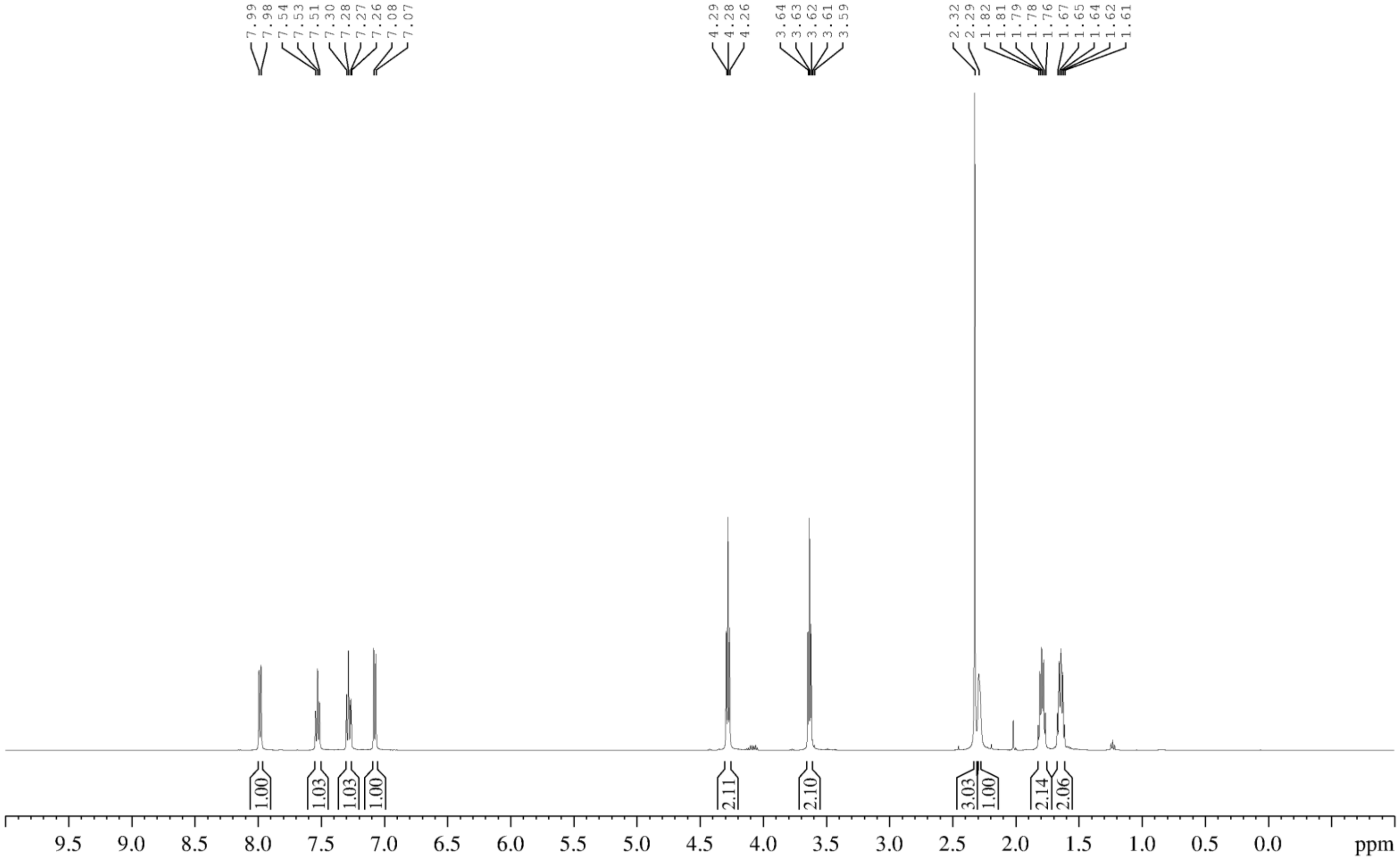

Figure S61. ^{13}C NMR (126 MHz, $CDCl_3$, 298 K) of **6c**.

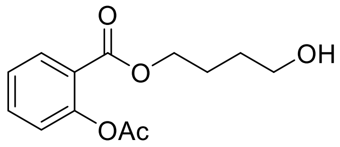

6c

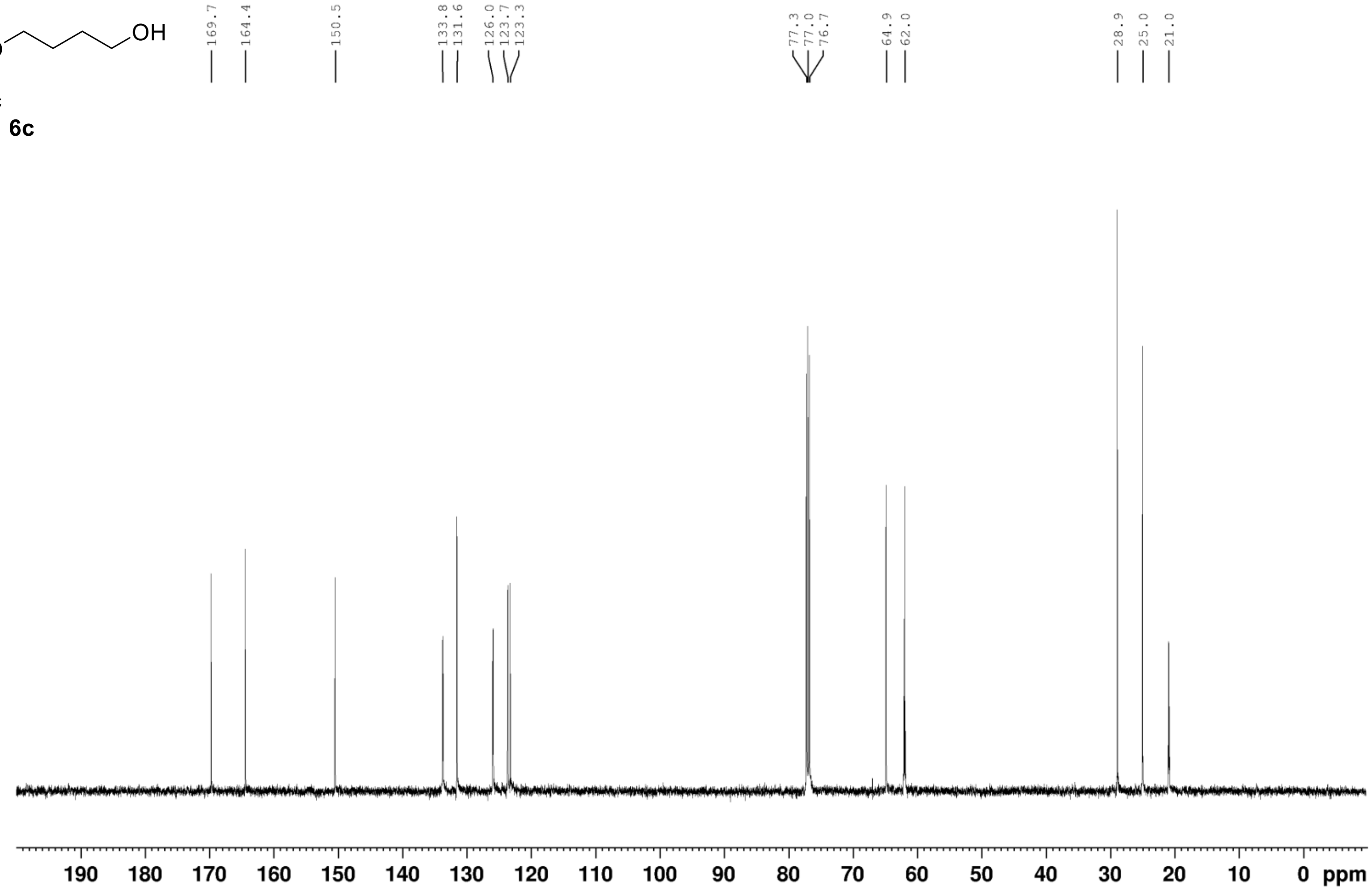

Figure S62. ^{1}H NMR (500 MHz, $CDCl_3$, 298 K) of **6d**.

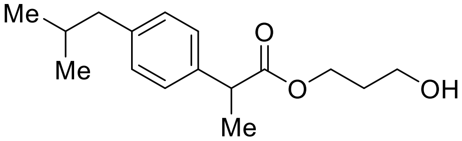

6d

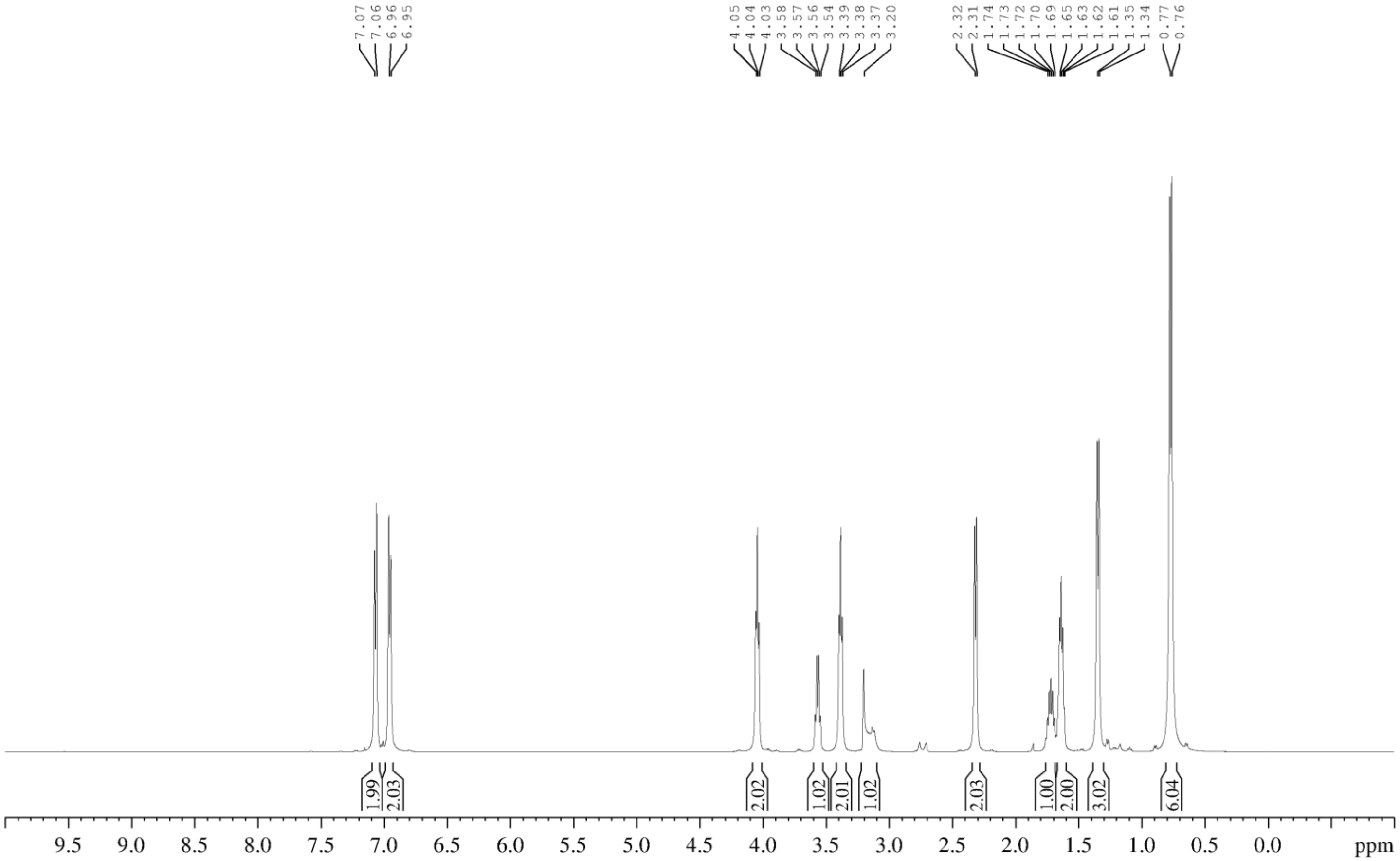

Figure S63. ^{13}C NMR (126 MHz, $CDCl_3$, 298 K) of **6d**.

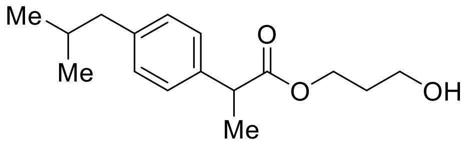

6d

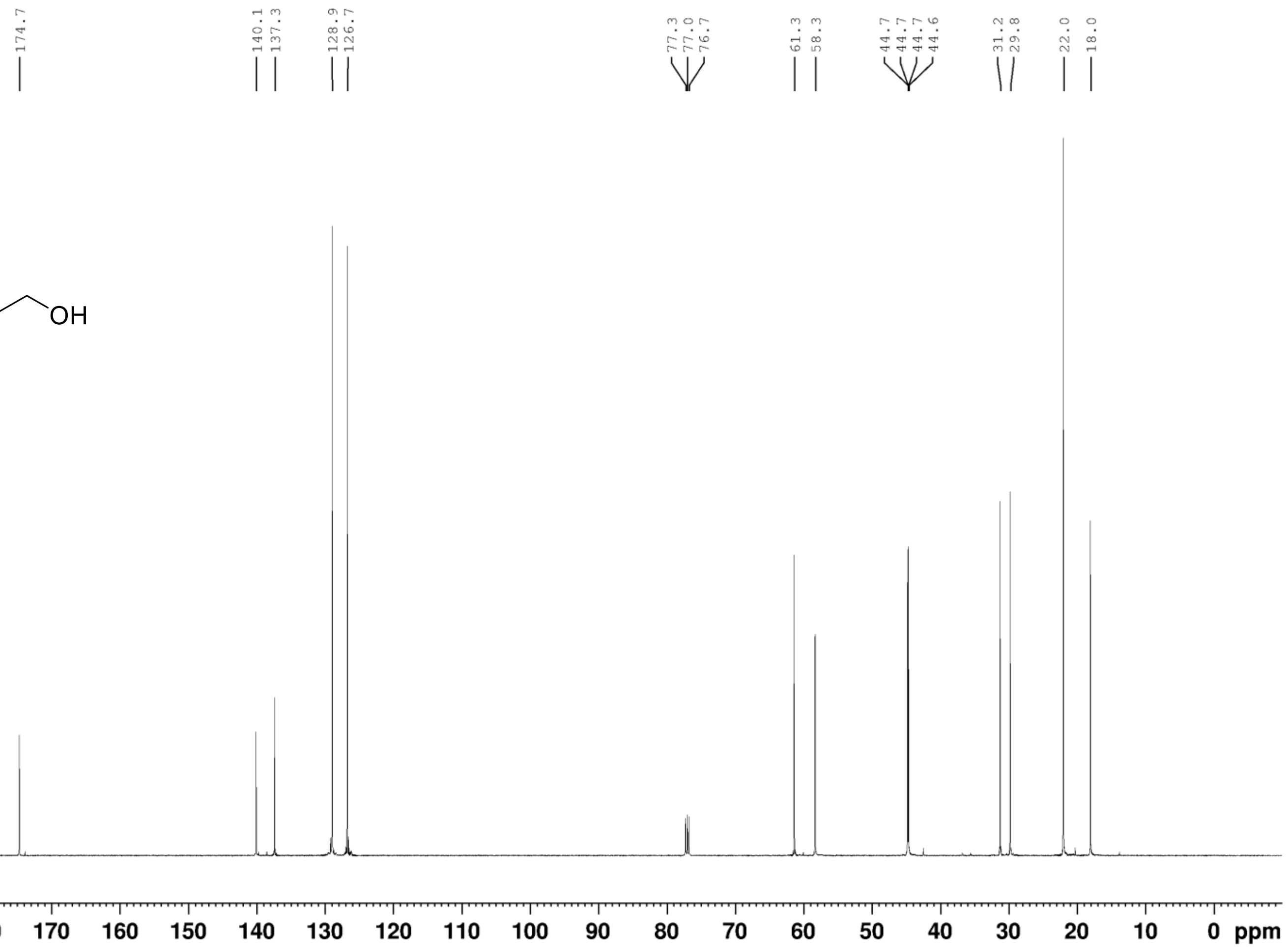

Figure S64. ^{1}H NMR (500 MHz, $CDCl_3$, 298 K) of **6e**.

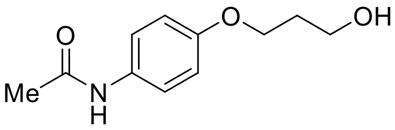

6e

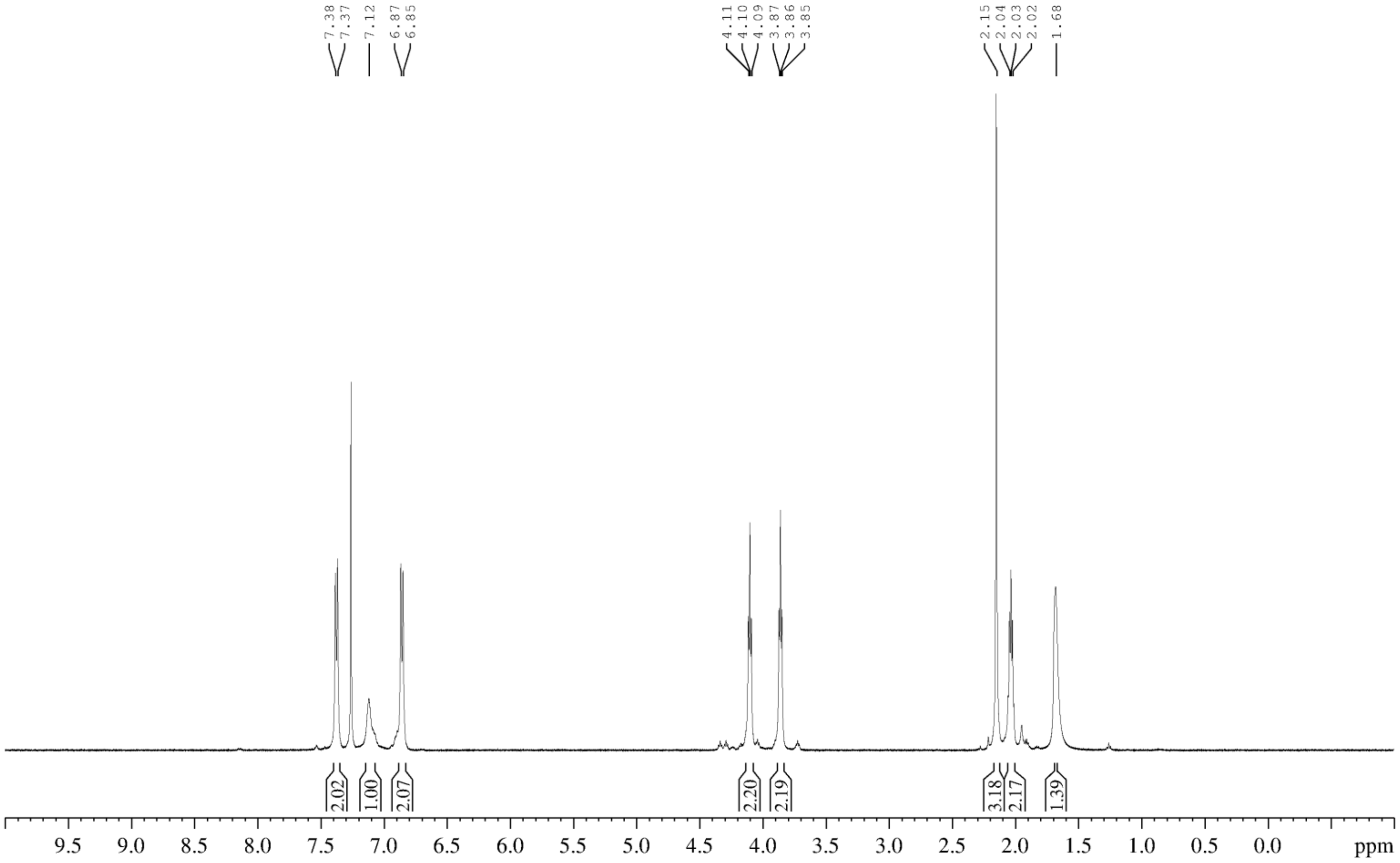

Figure S65. ^{13}C NMR (126 MHz, $CDCl_3$, 298 K) of **6e**.

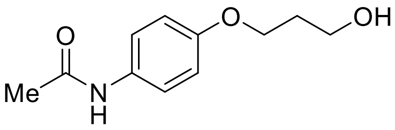

6e

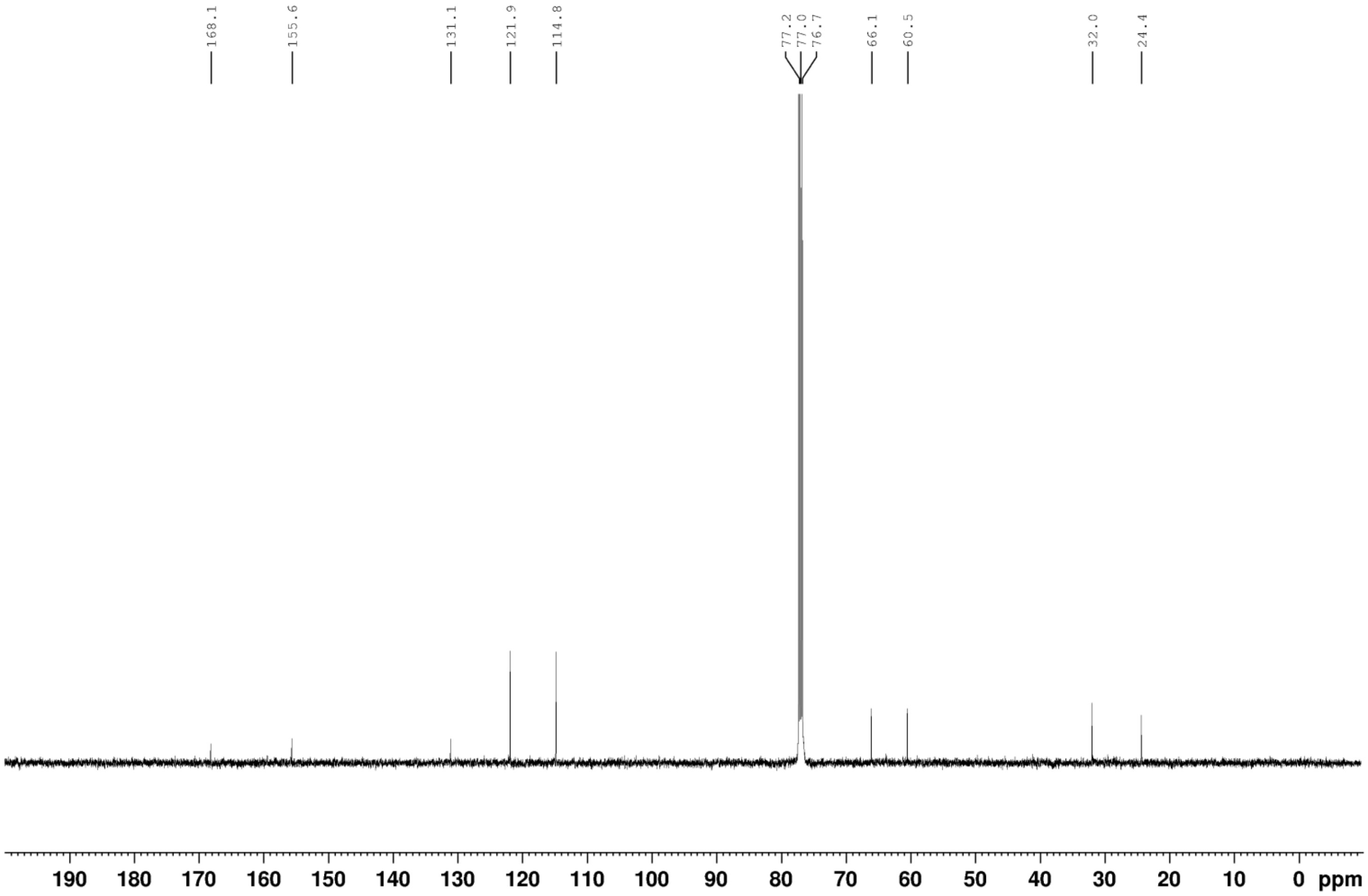

Figure S66. ^{1}H NMR (500 MHz, $CDCl_3$, 298 K) of **6f**.

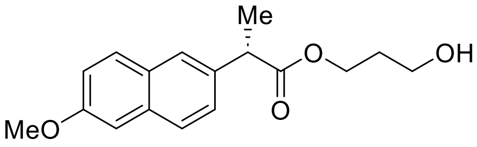

6f

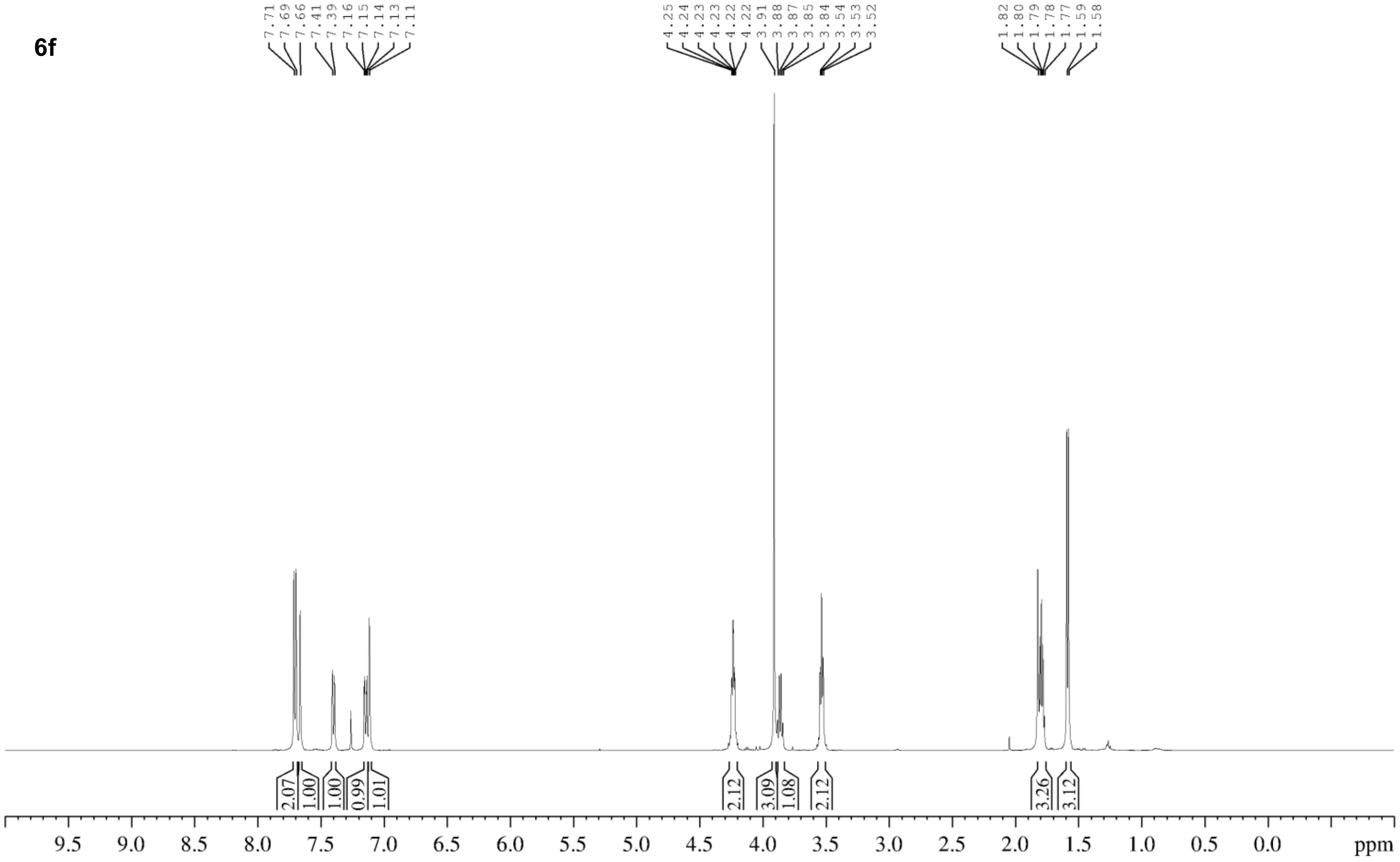

Figure S66. ^{13}C NMR (126 MHz, $CDCl_3$, 298 K) of **6f**.

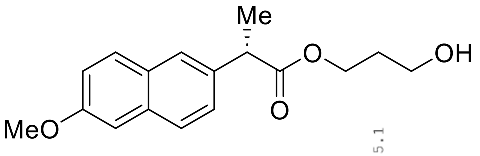

6f

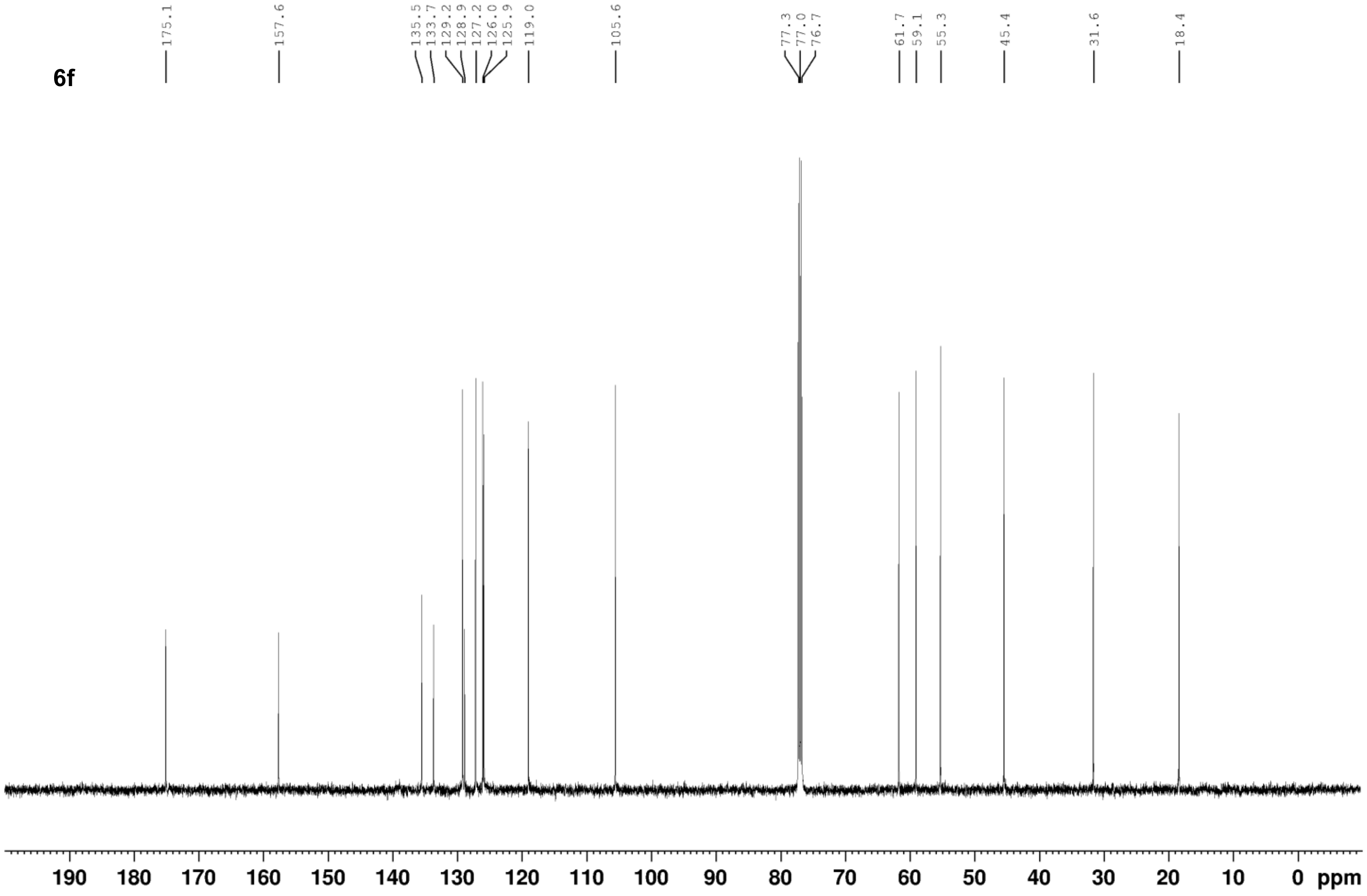

Figure S67. ^{1}H NMR (500 MHz, $CDCl_3$, 298 K) of **6g**.

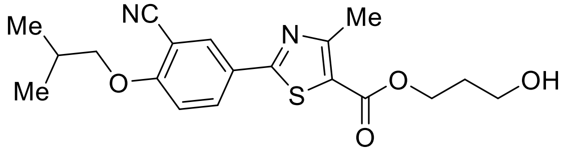

6g

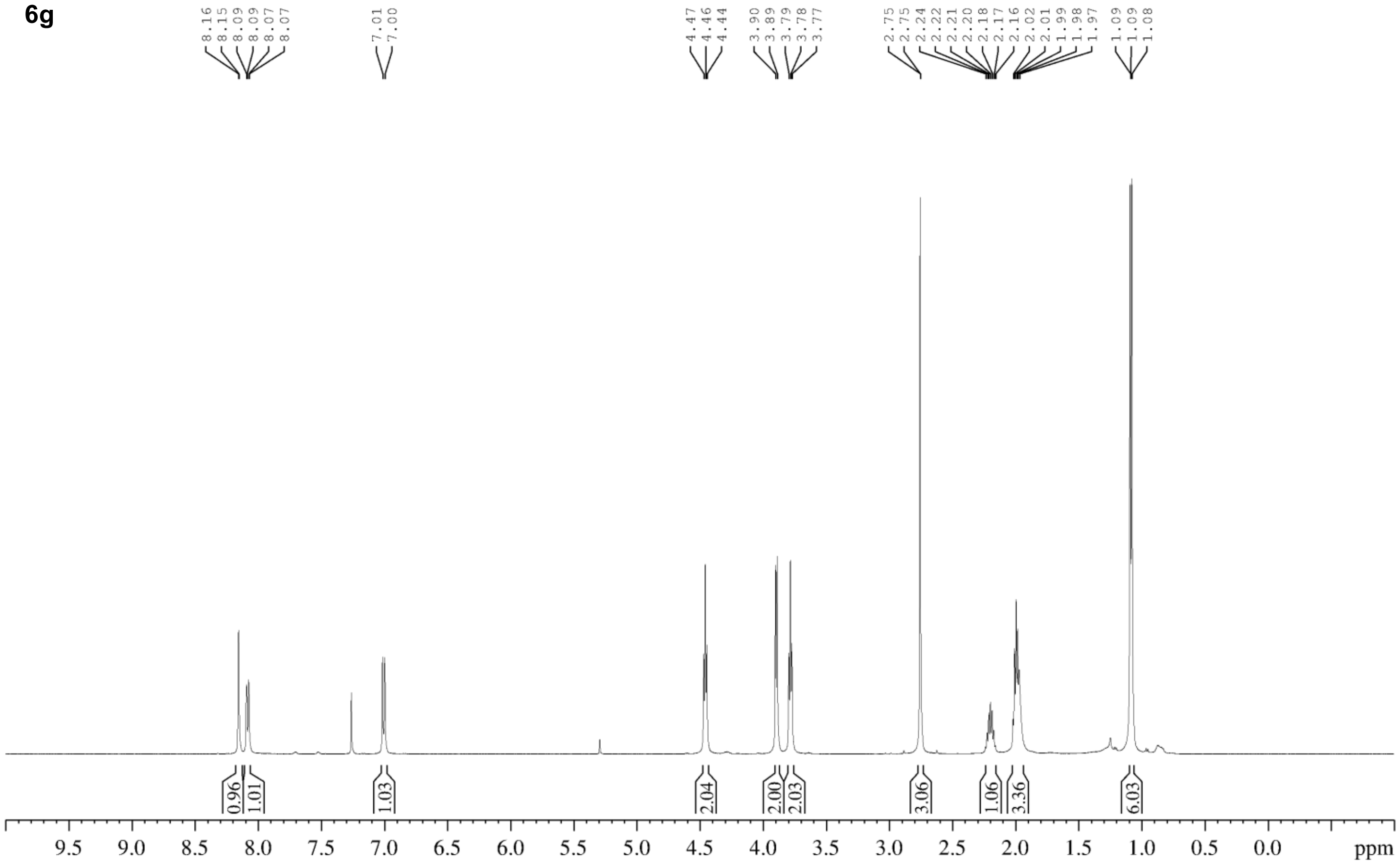

Figure S68. ^{13}C NMR (126 MHz, $CDCl_3$, 298 K) of **6g**.

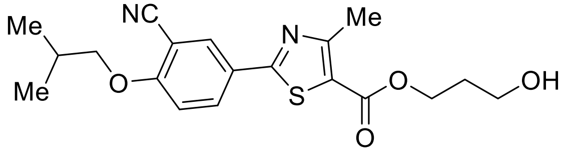

6g

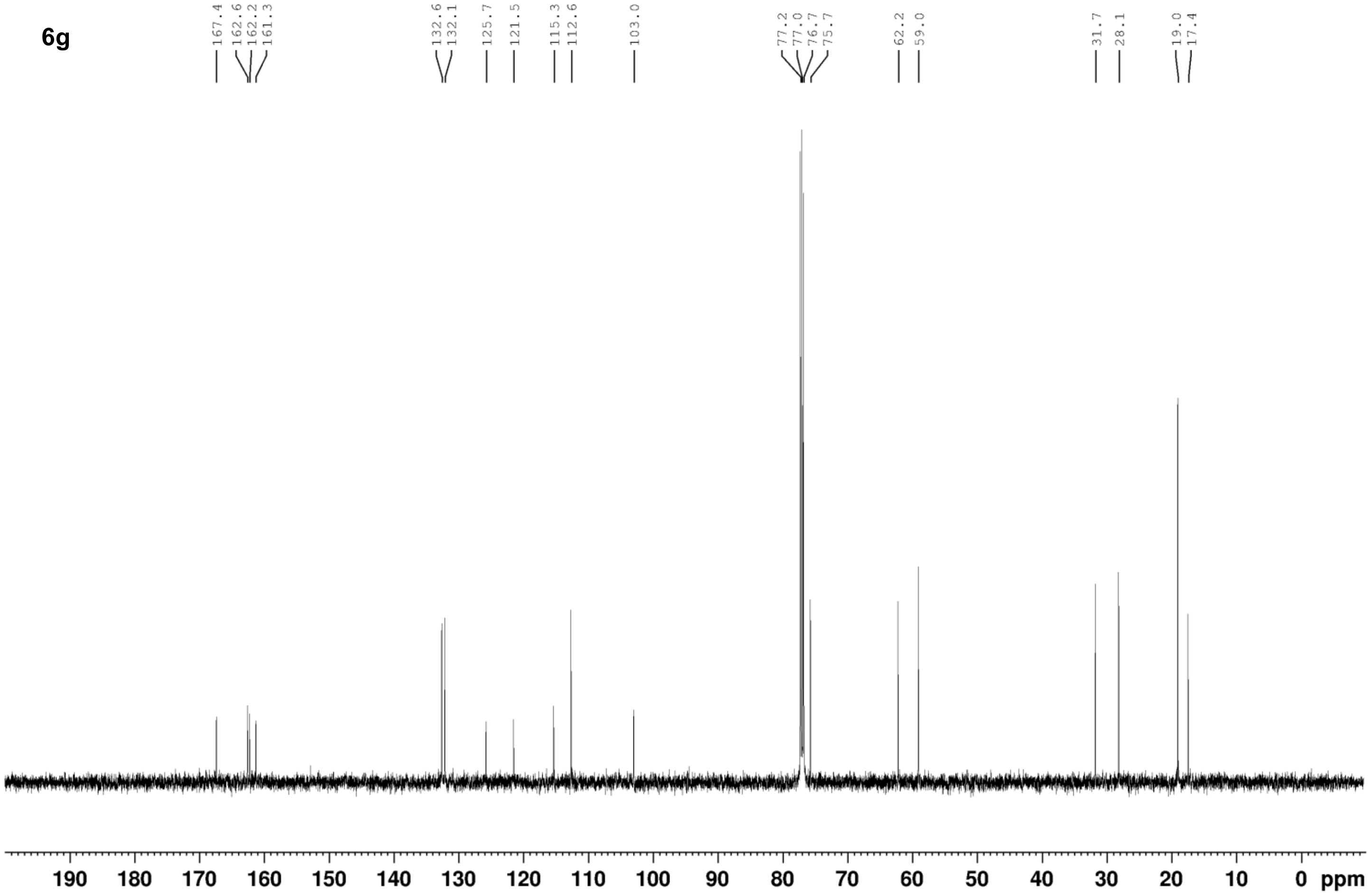

Figure S69. ^{1}H NMR (500 MHz, $CDCl_3$, 298 K) of **6h**.

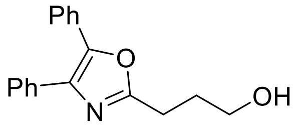

6h

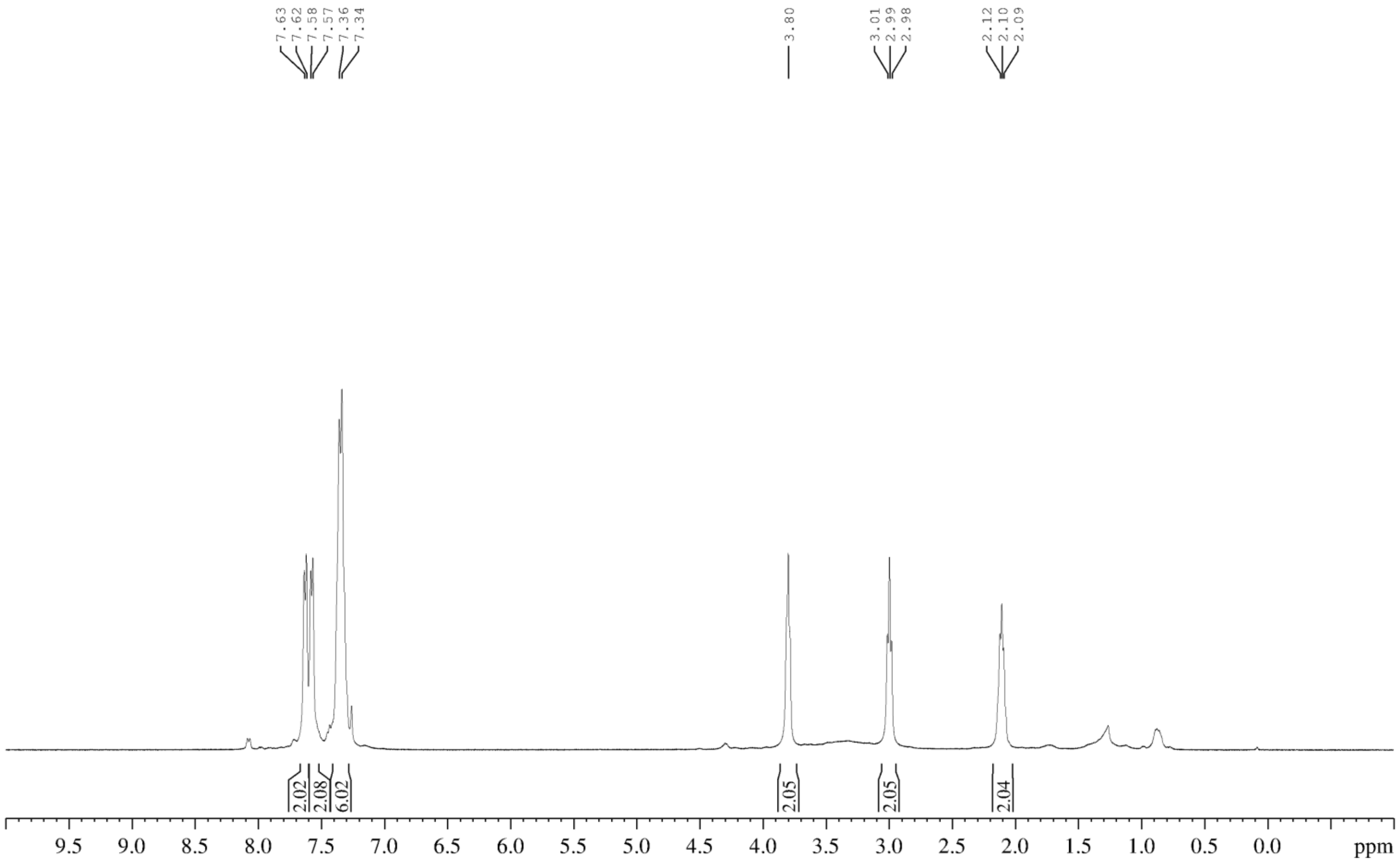

Figure S70. ^{13}C NMR (126 MHz, $CDCl_3$, 298 K) of **6h**.

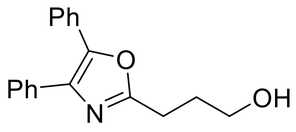

6h

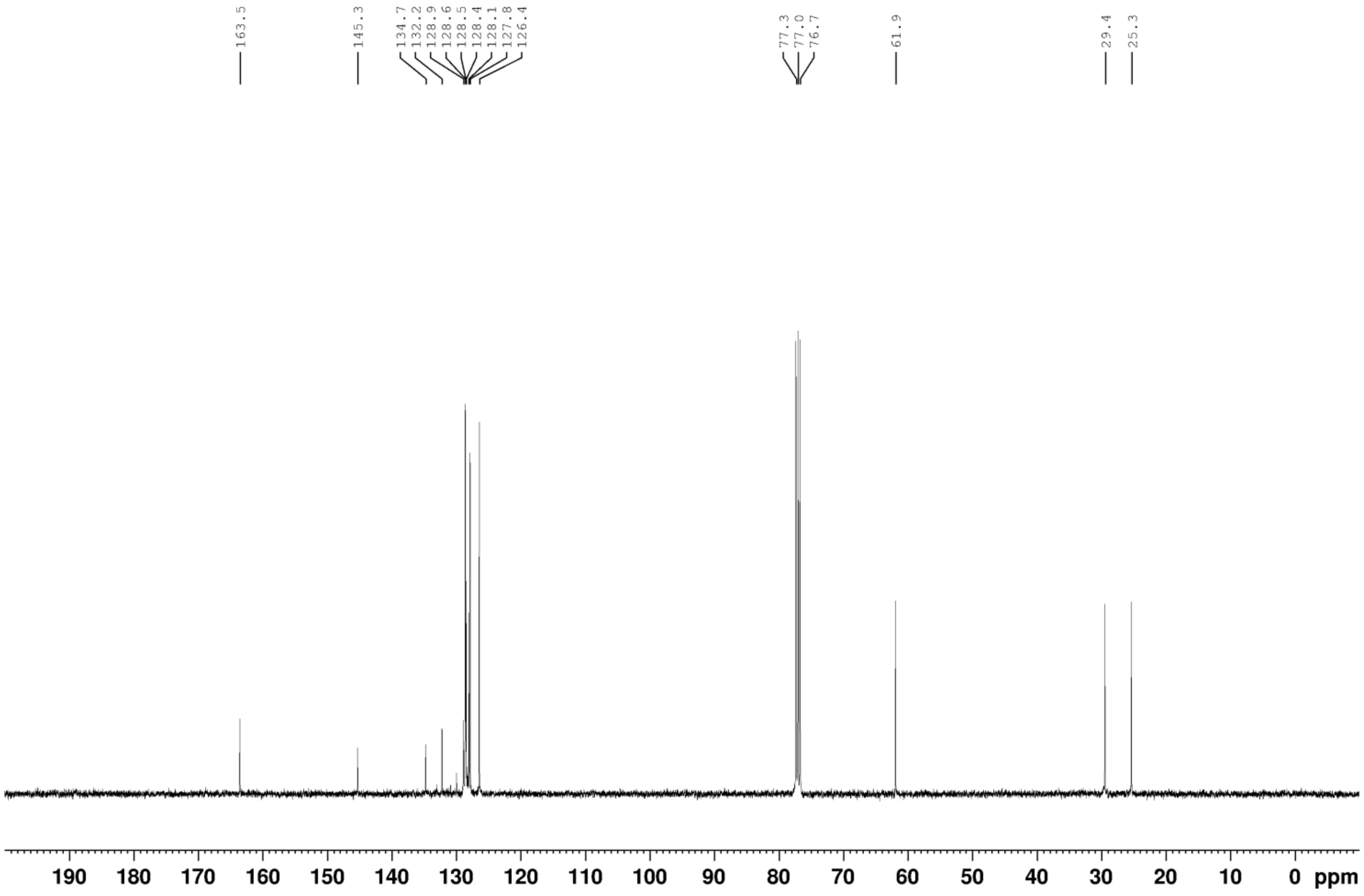

Figure S71. ^{1}H NMR (500 MHz, $CDCl_3$, 298 K) of **6i**.

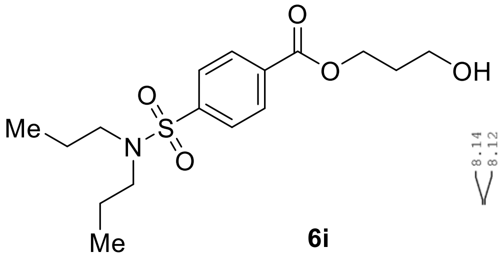

6i

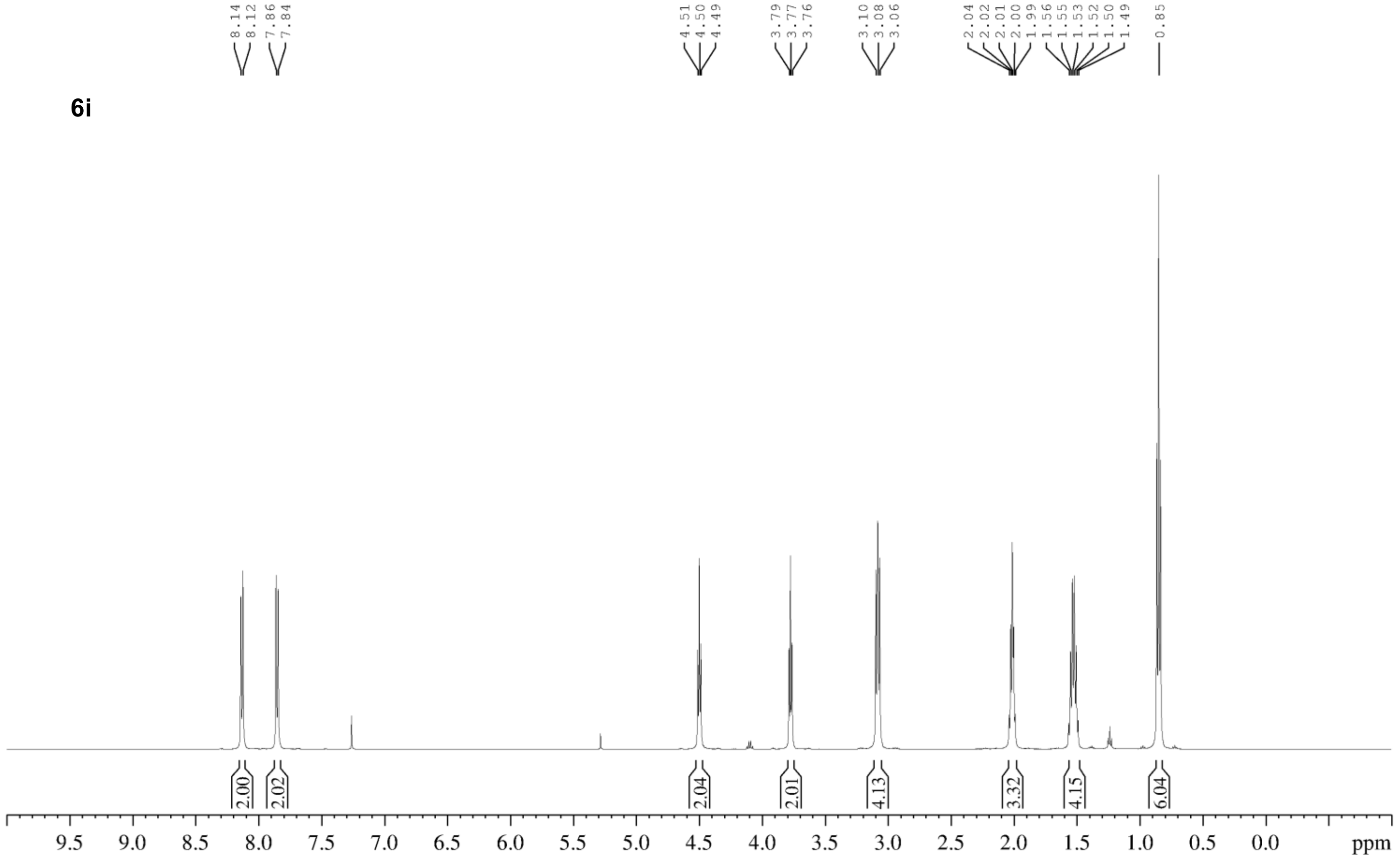

Figure S72. ^{13}C NMR (126 MHz, $CDCl_3$, 298 K) of **6i**.

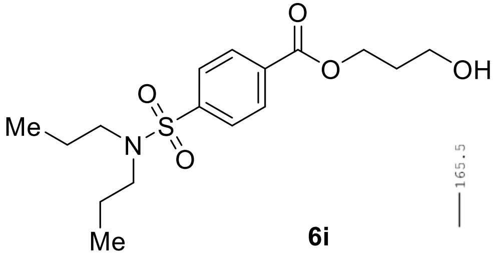

6i

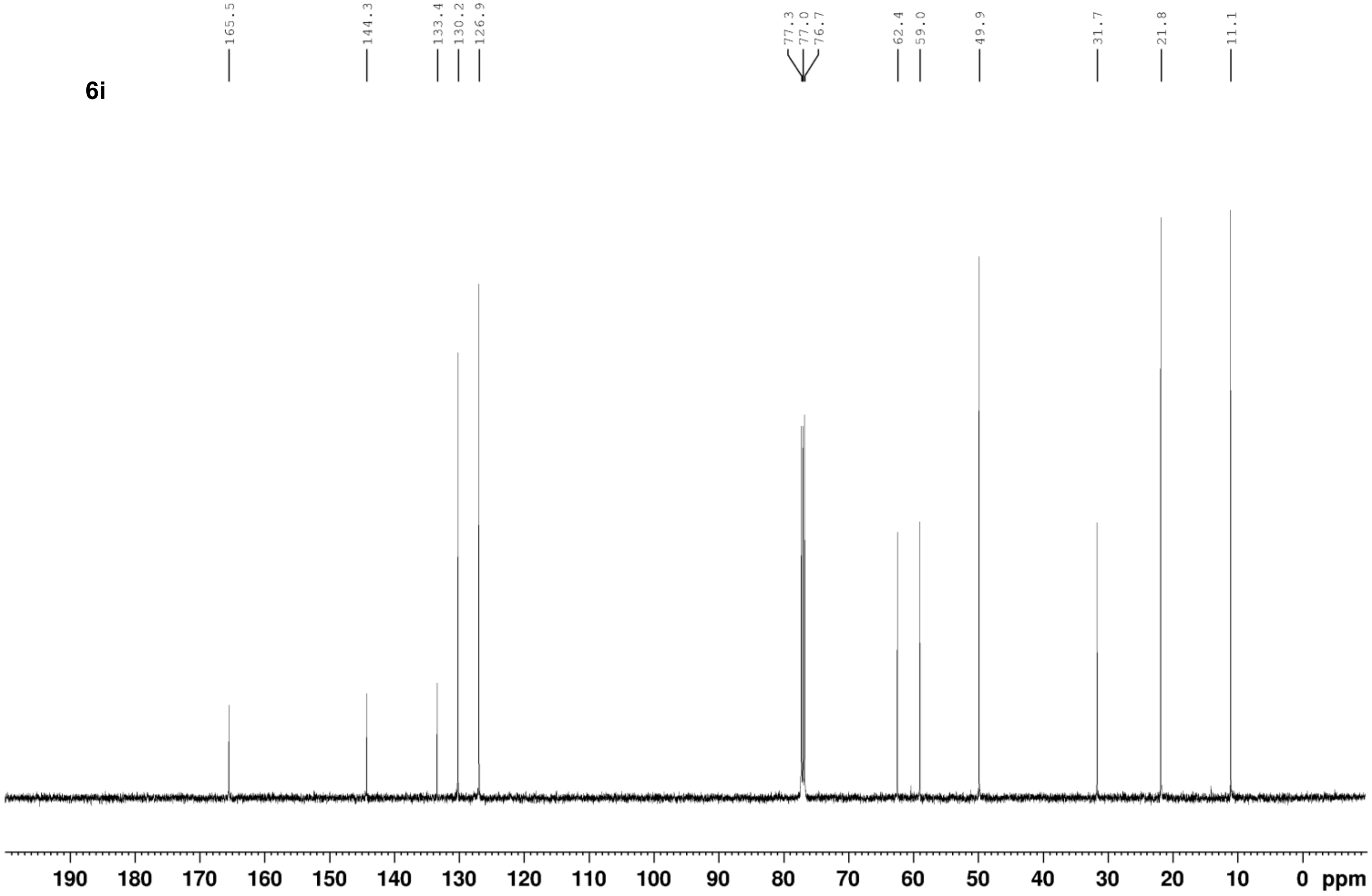

Figure S73. ^{1}H NMR (500 MHz, $CDCl_3$, 298 K) of **6j**.

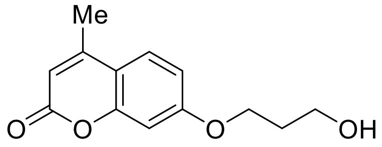

6j

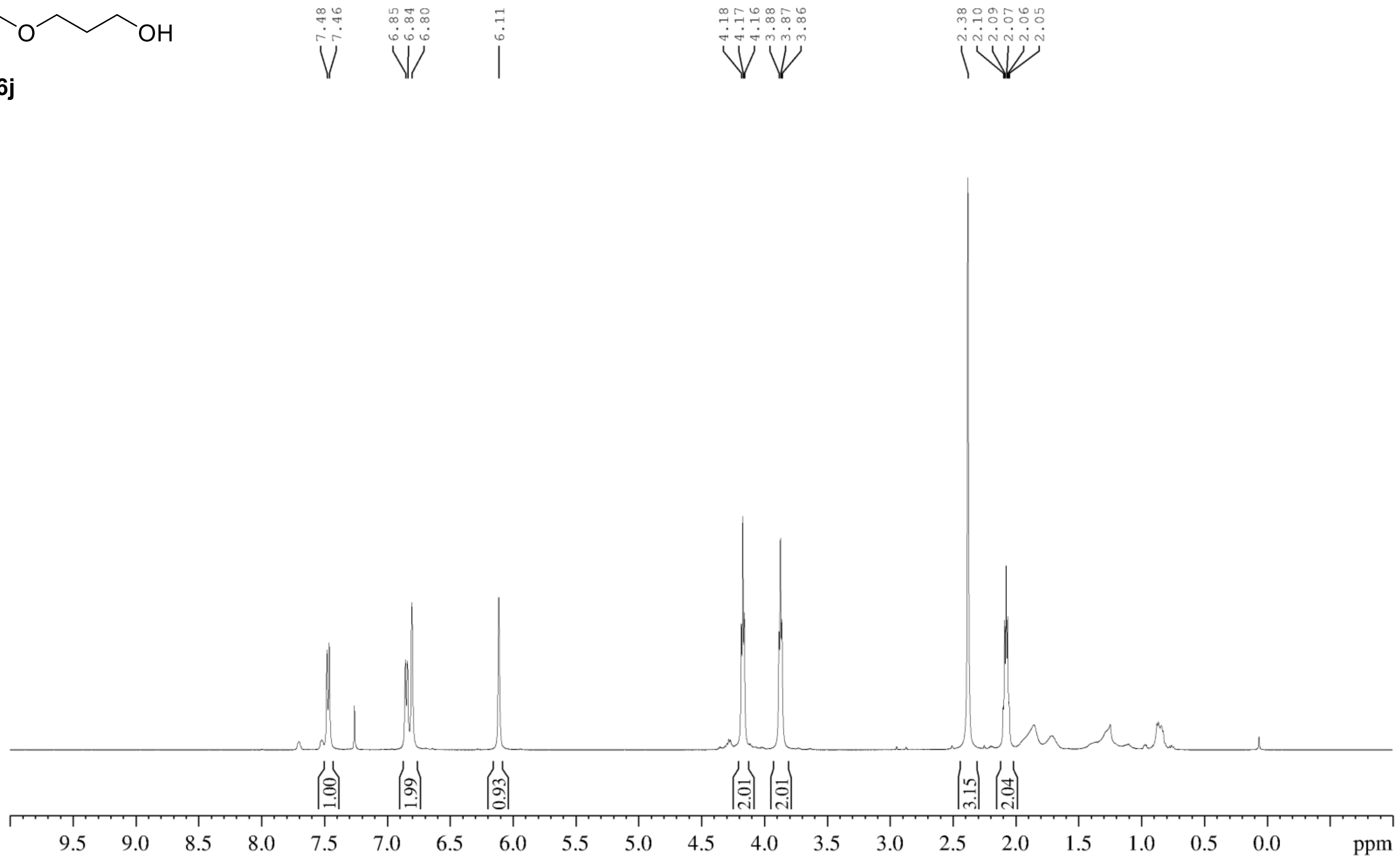

Figure S74. ^{13}C NMR (126 MHz, $CDCl_3$, 298 K) of **6j**.

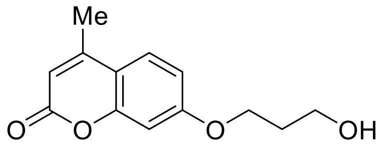

6j

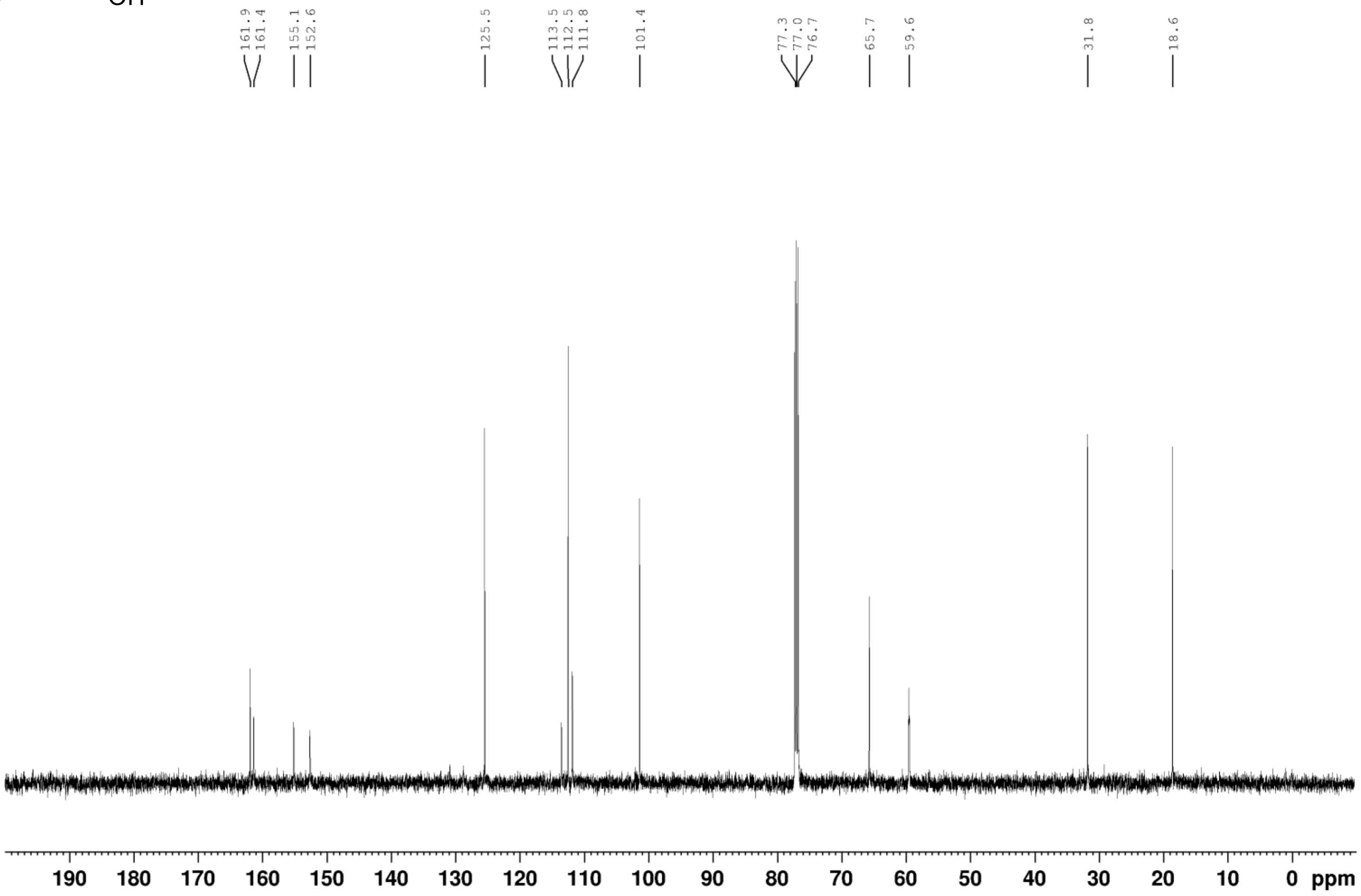

Figure S75. ^{1}H NMR (500 MHz, $CDCl_3$, 298 K) of **6k**.

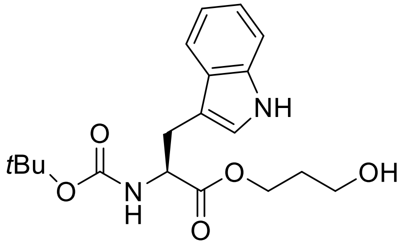

6k

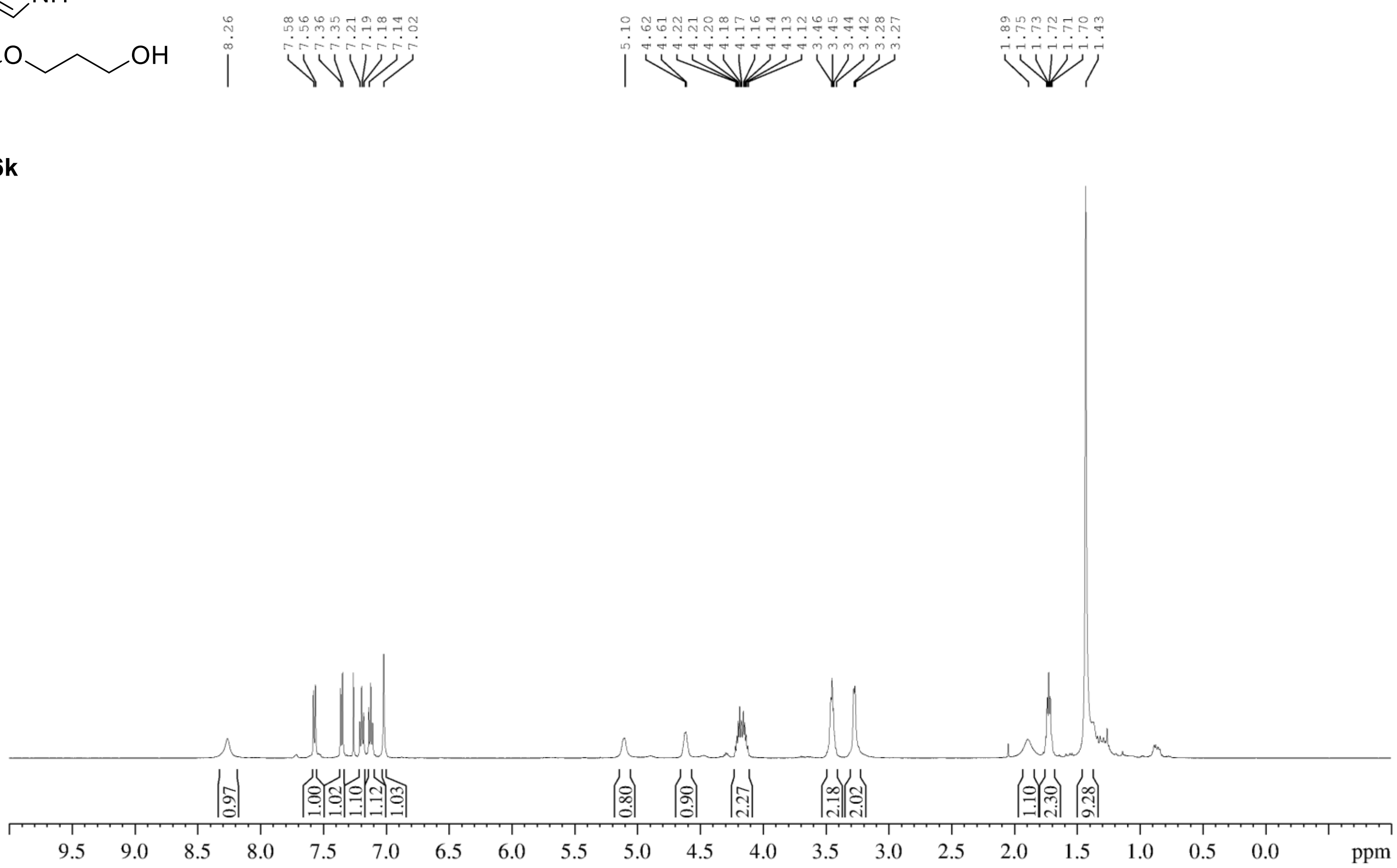

Figur S76. ^{13}C NMR (126 MHz, $CDCl_3$, 298 K) of **6k**.

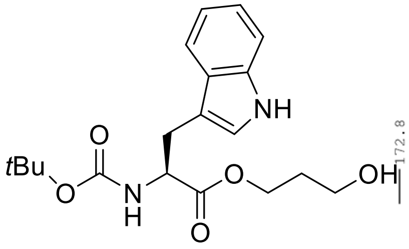

6k

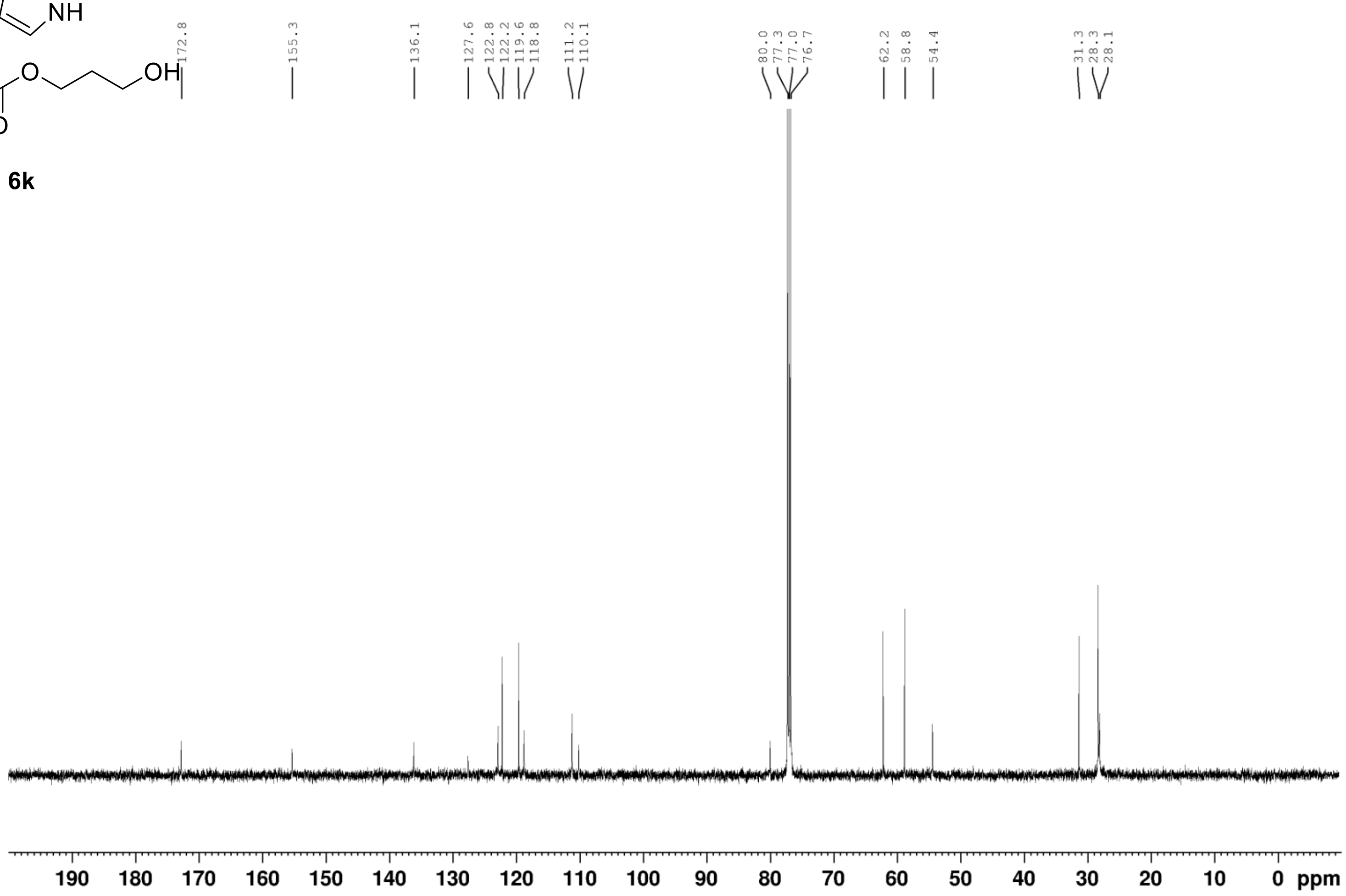

Figure S77. ^{1}H NMR (500 MHz, $CDCl_3$, 298 K) of **6l**.

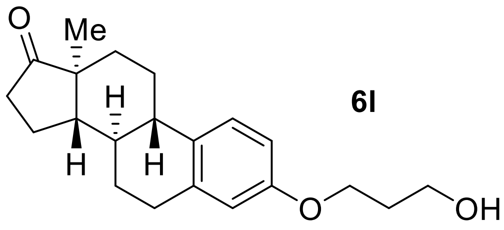

6l

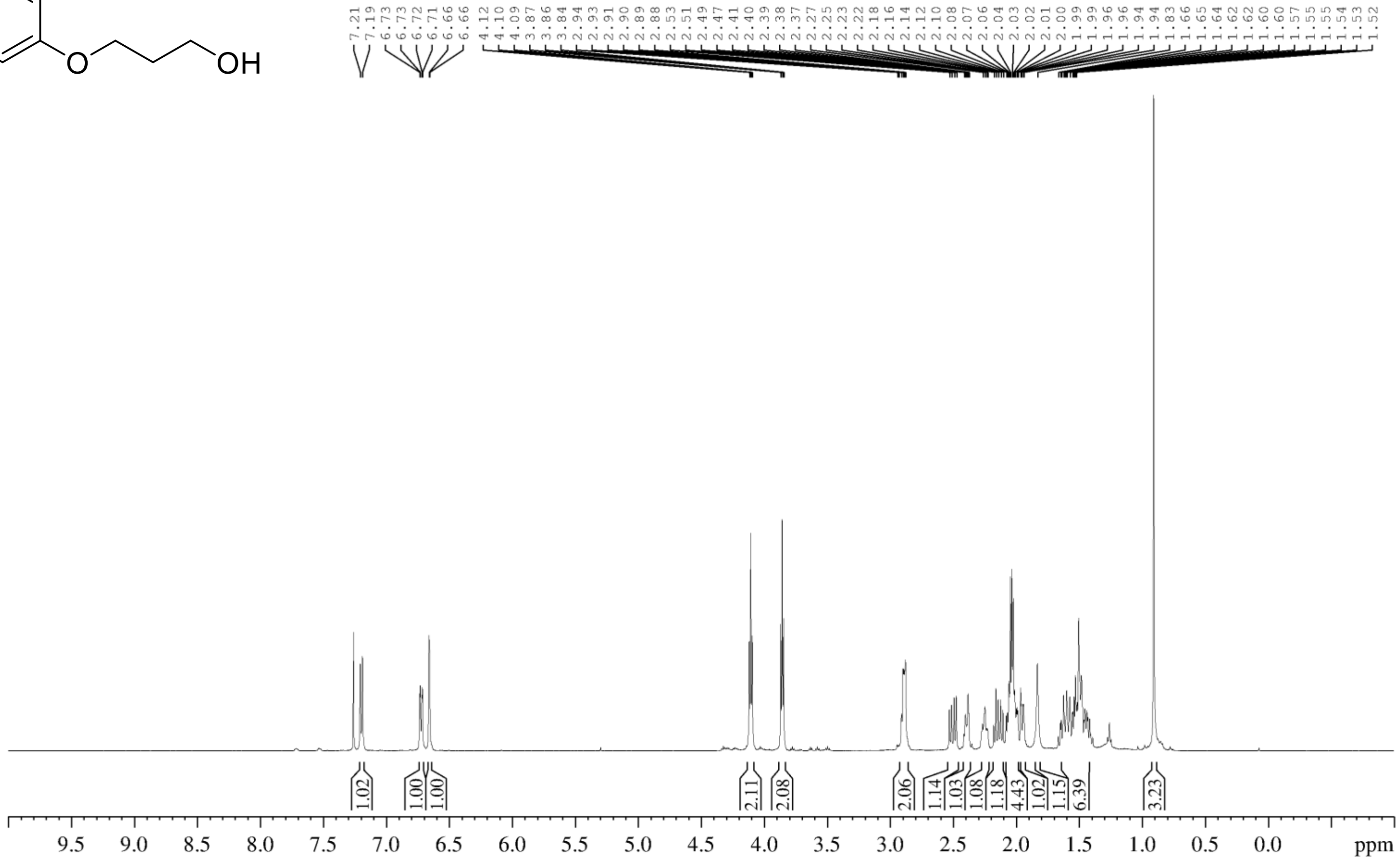

Figure S78. ^{13}C NMR (126 MHz, $CDCl_3$, 298 K) of **6l**.

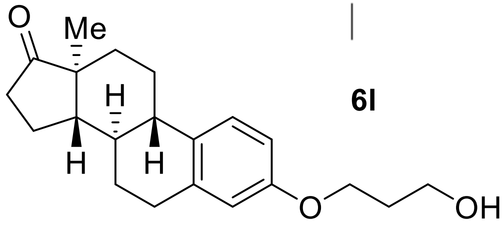

6l

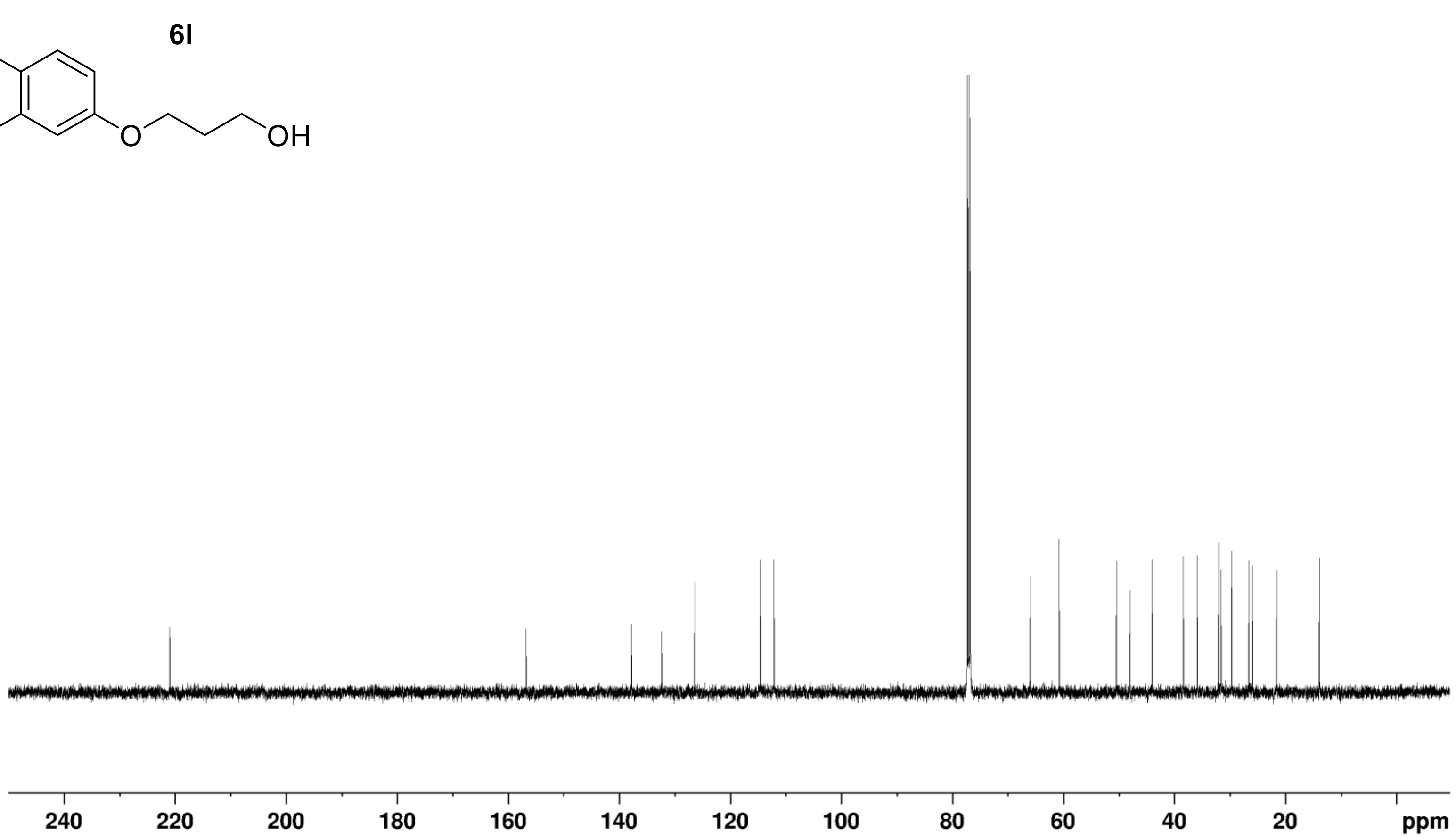

Figure S79. ^{1}H NMR (500 MHz, $CDCl_3$, 298 K) of **6m**.

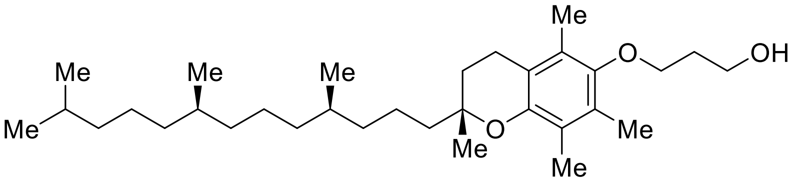

6m

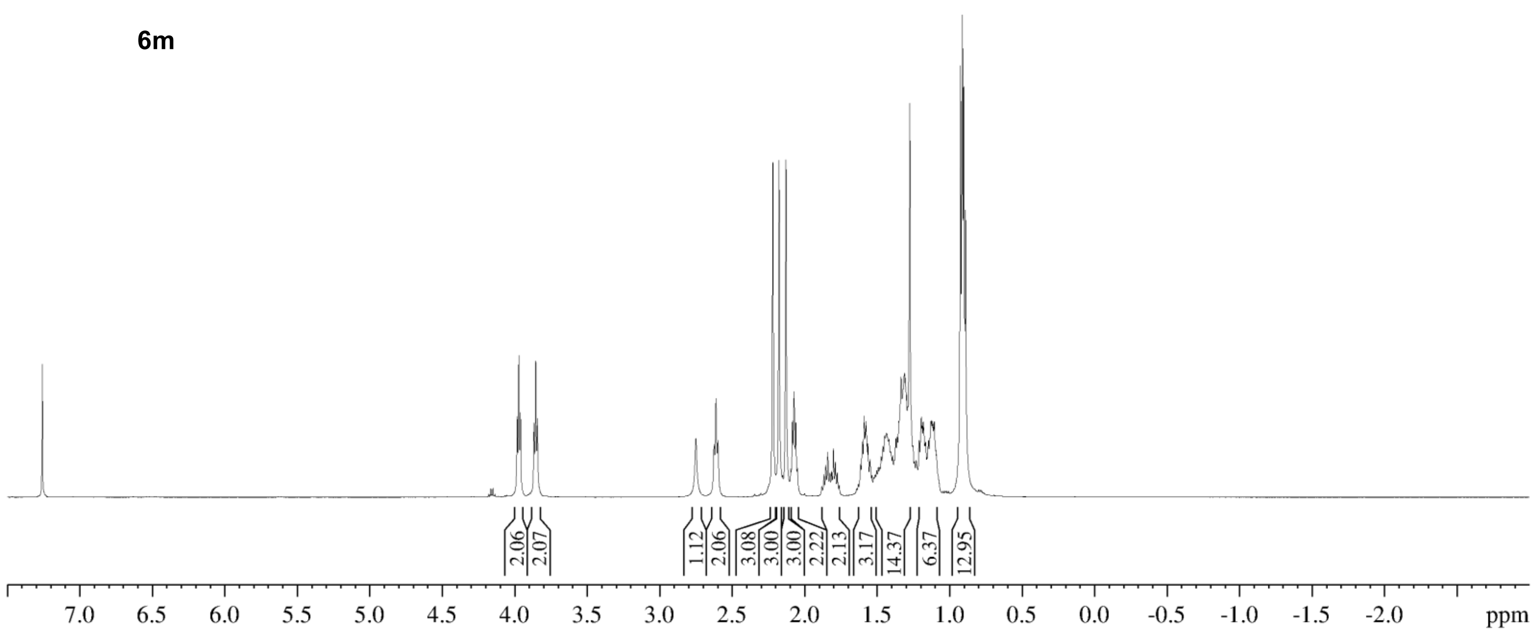

Figure S80. ^{13}C NMR (126 MHz, $CDCl_3$, 298 K) of **6m**.

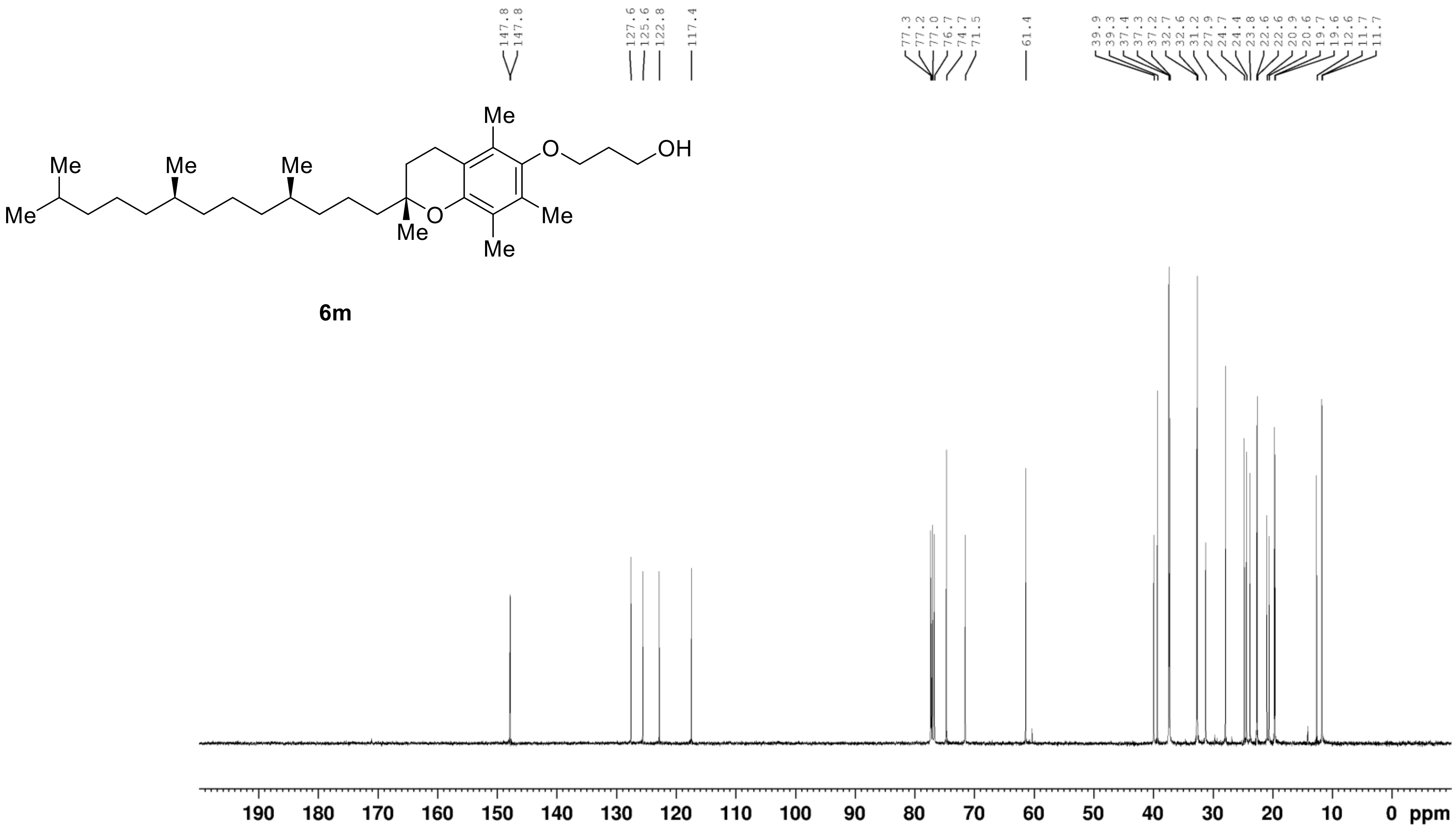

Figure S81. ^{1}H NMR (500 MHz, $CDCl_3$, 298 K) of **2c**.

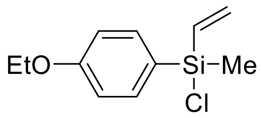

2c

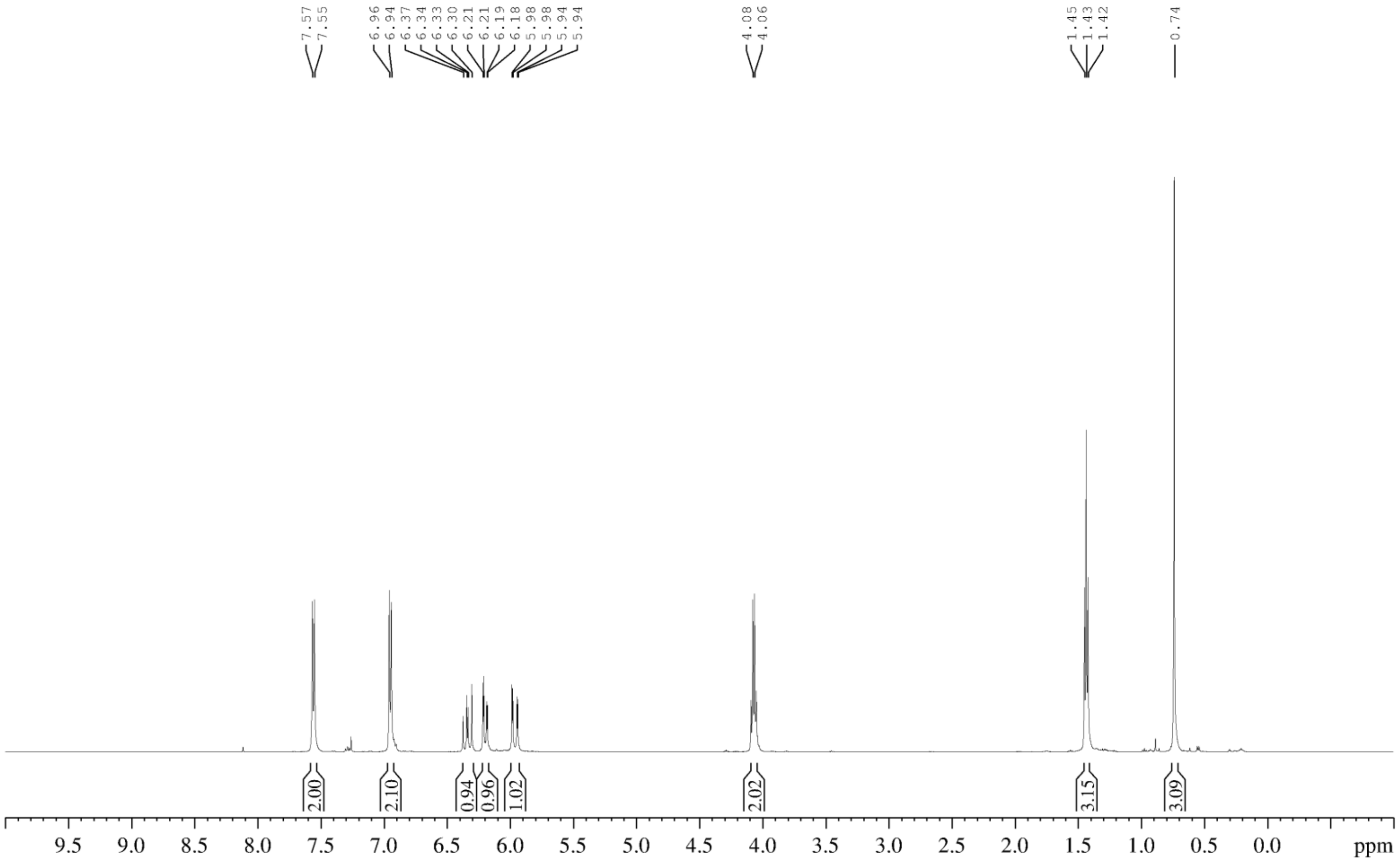

Figure S82. ^{13}C NMR (126 MHz, $CDCl_3$, 298 K) of **2c**.

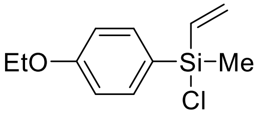

2c

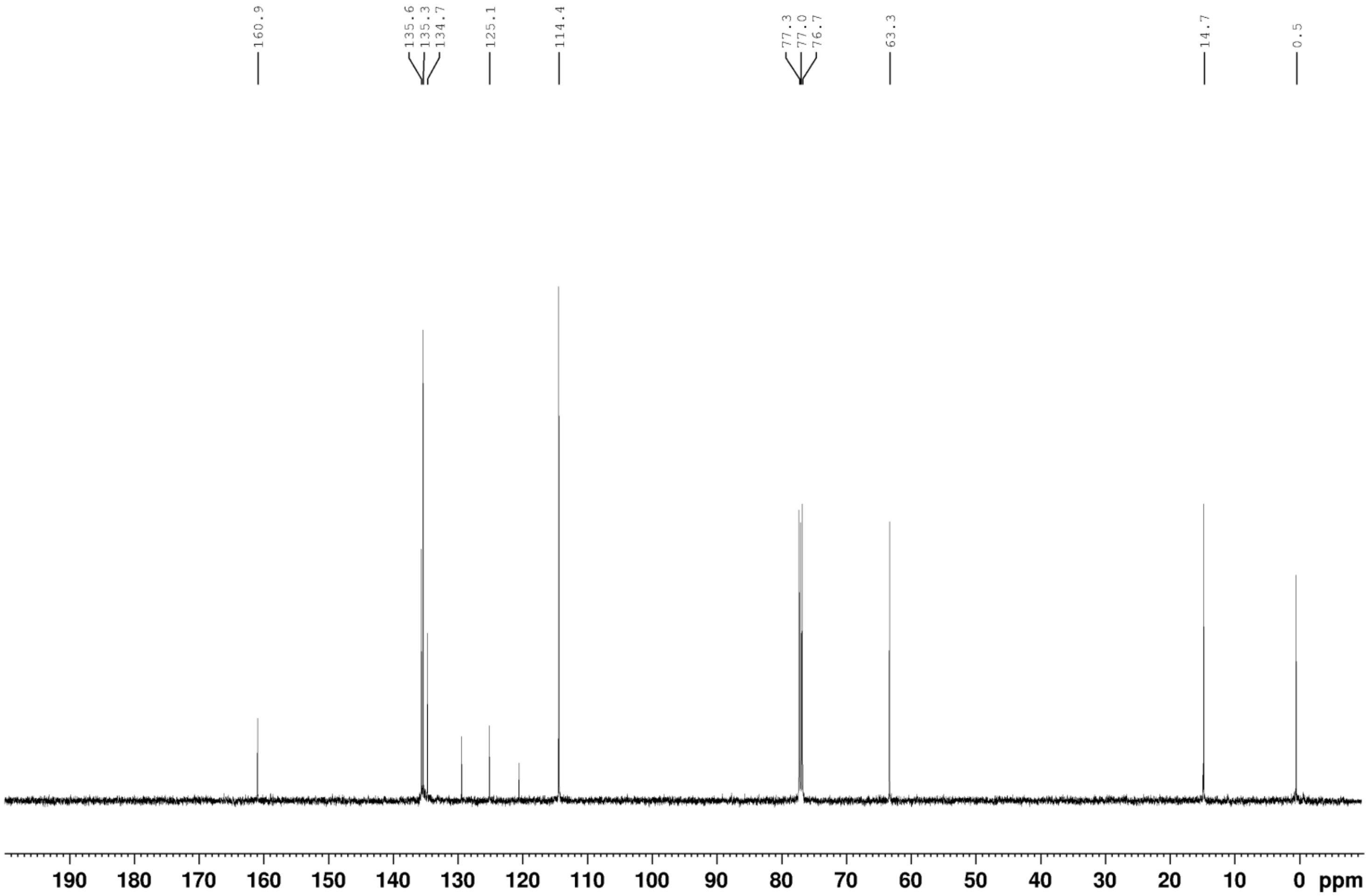

Figure S83. $^1H/^{29}Si$ HMQC NMR (500/99 MHz, $CDCl_3$, 298 K, optimized for J = 7 Hz) of **2c**.

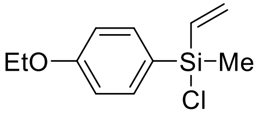

2c

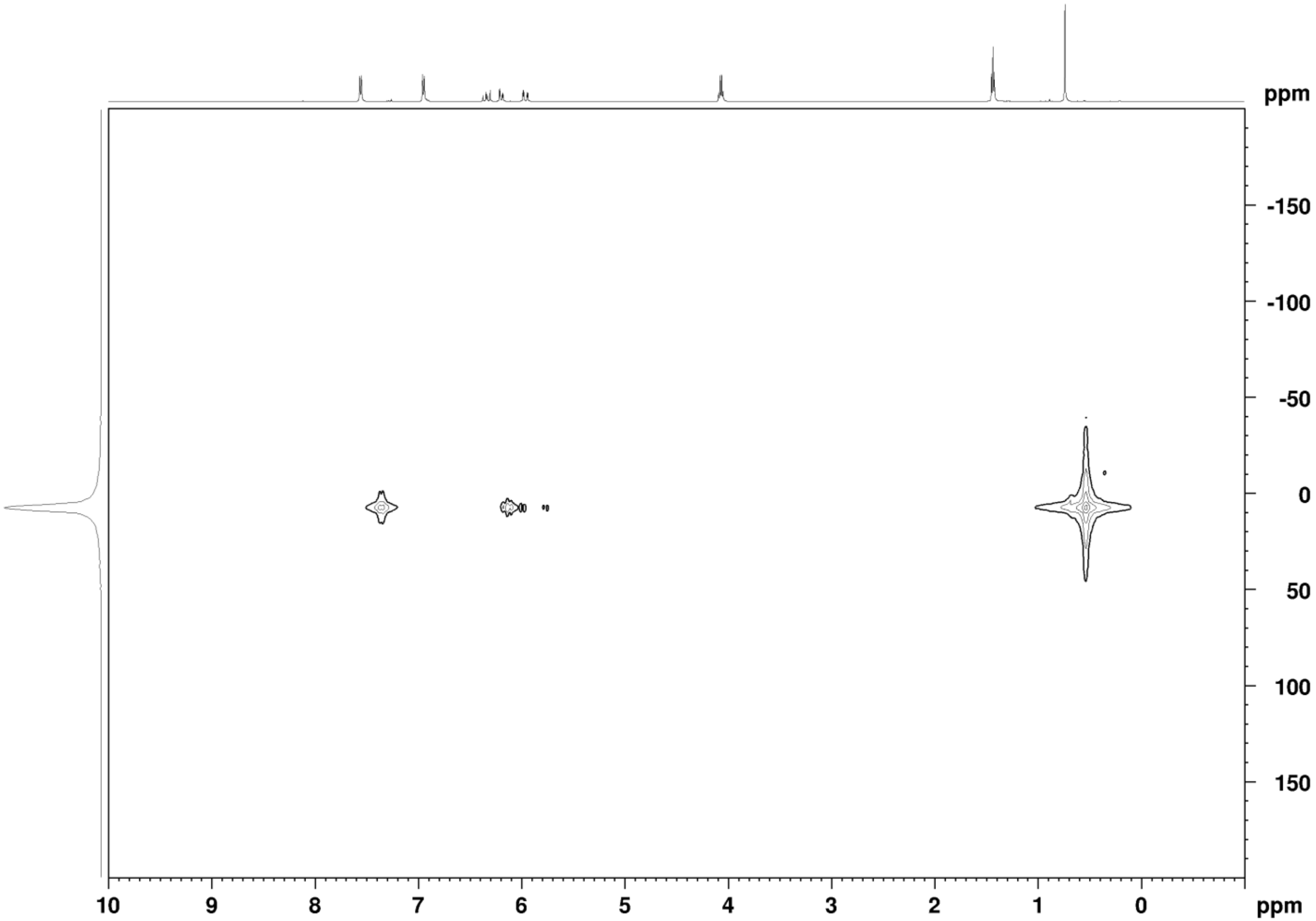

Figure S84. ^{1}H NMR (500 MHz, $CDCl_3$, 298 K) of **3aa**.

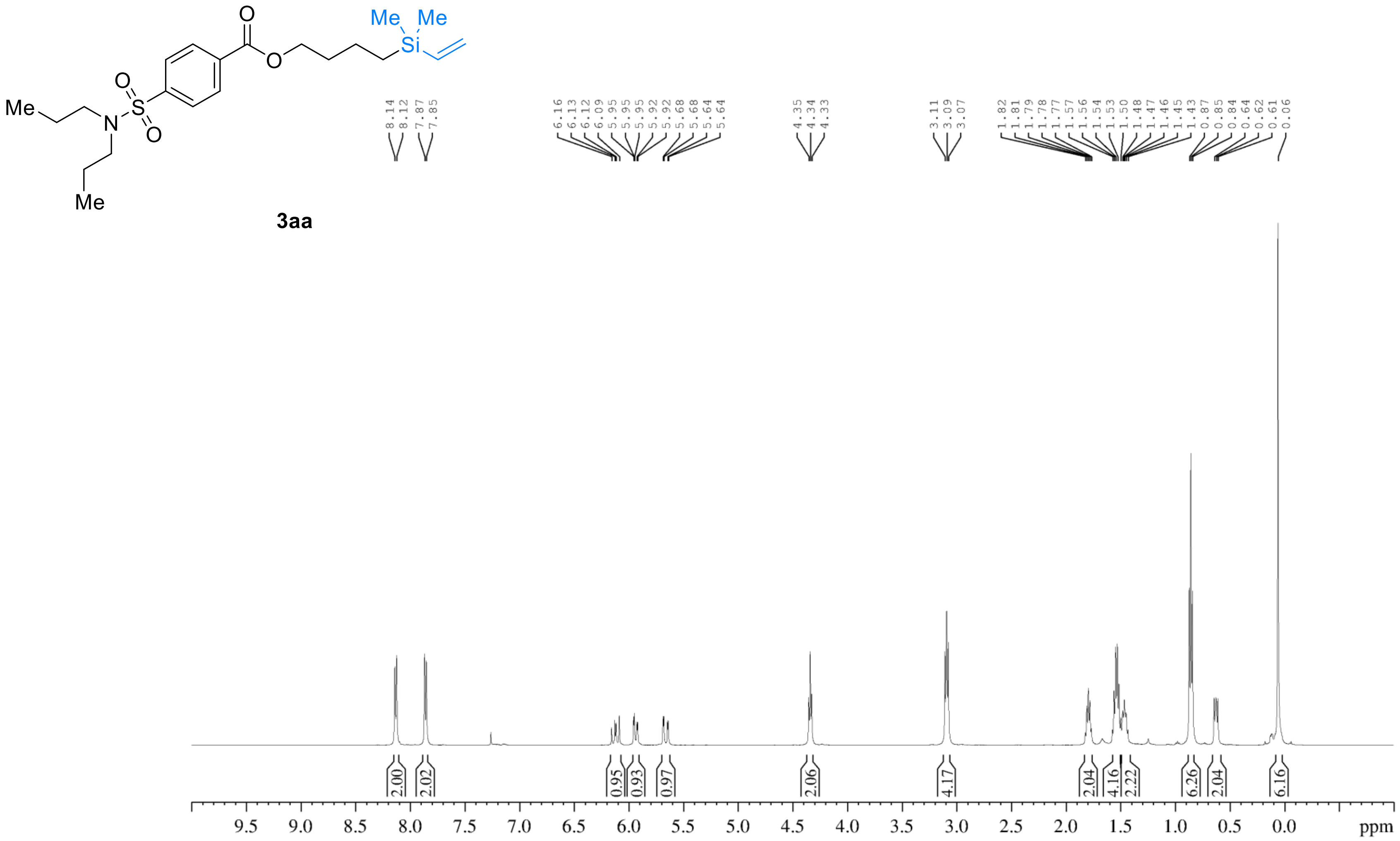

Figure S85. ^{13}C NMR (126 MHz, $CDCl_3$, 298 K) of **3aa**.

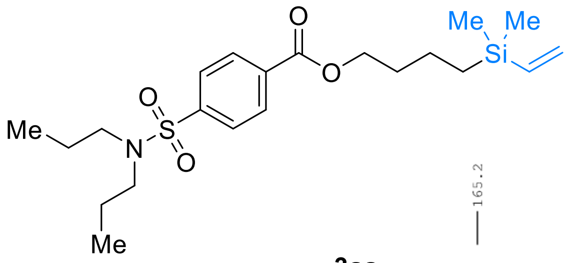

3aa

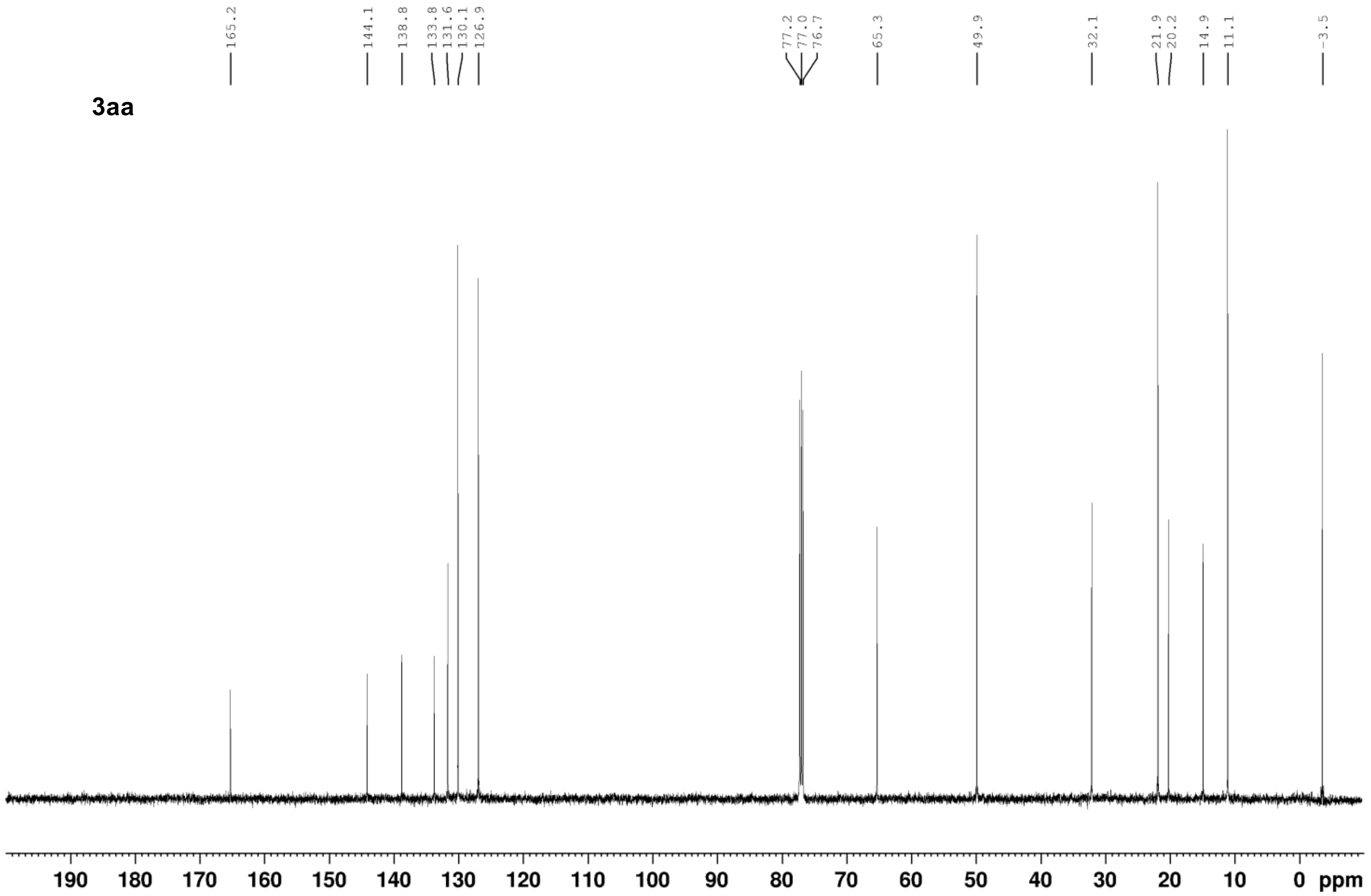

Figure S86. $^{1}H/^{29}Si$ HMQC NMR (500/99 MHz, $CDCl_3$, 298 K, optimized for J = 7 Hz) of **3aa**.

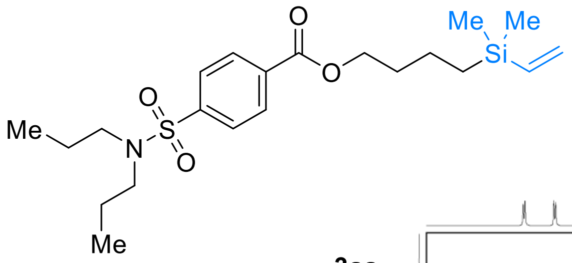

3aa

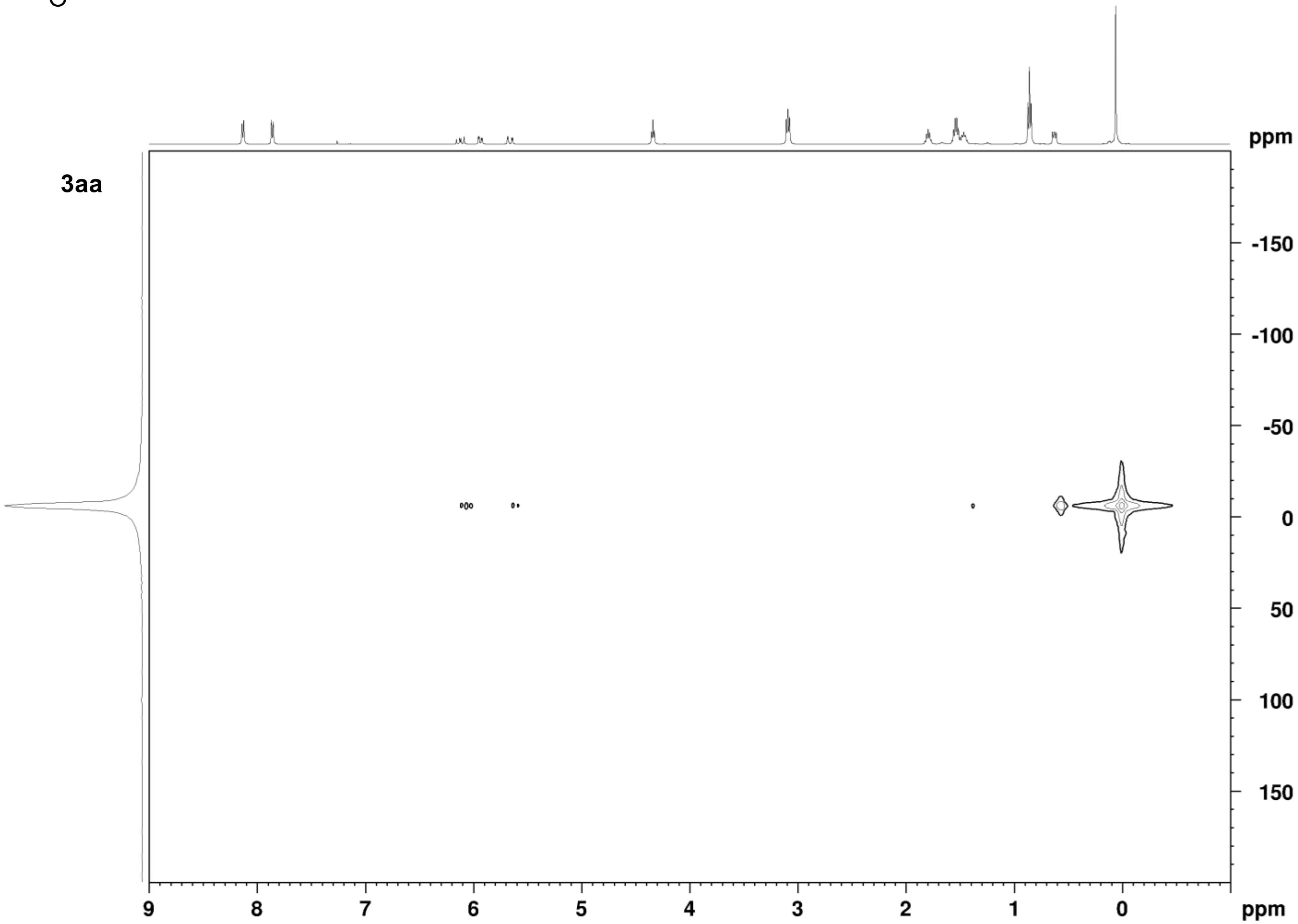

Figure S87. ^{1}H NMR (500 MHz, $CDCl_3$, 298 K) of **3ba**.

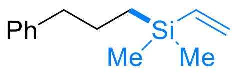

3ba

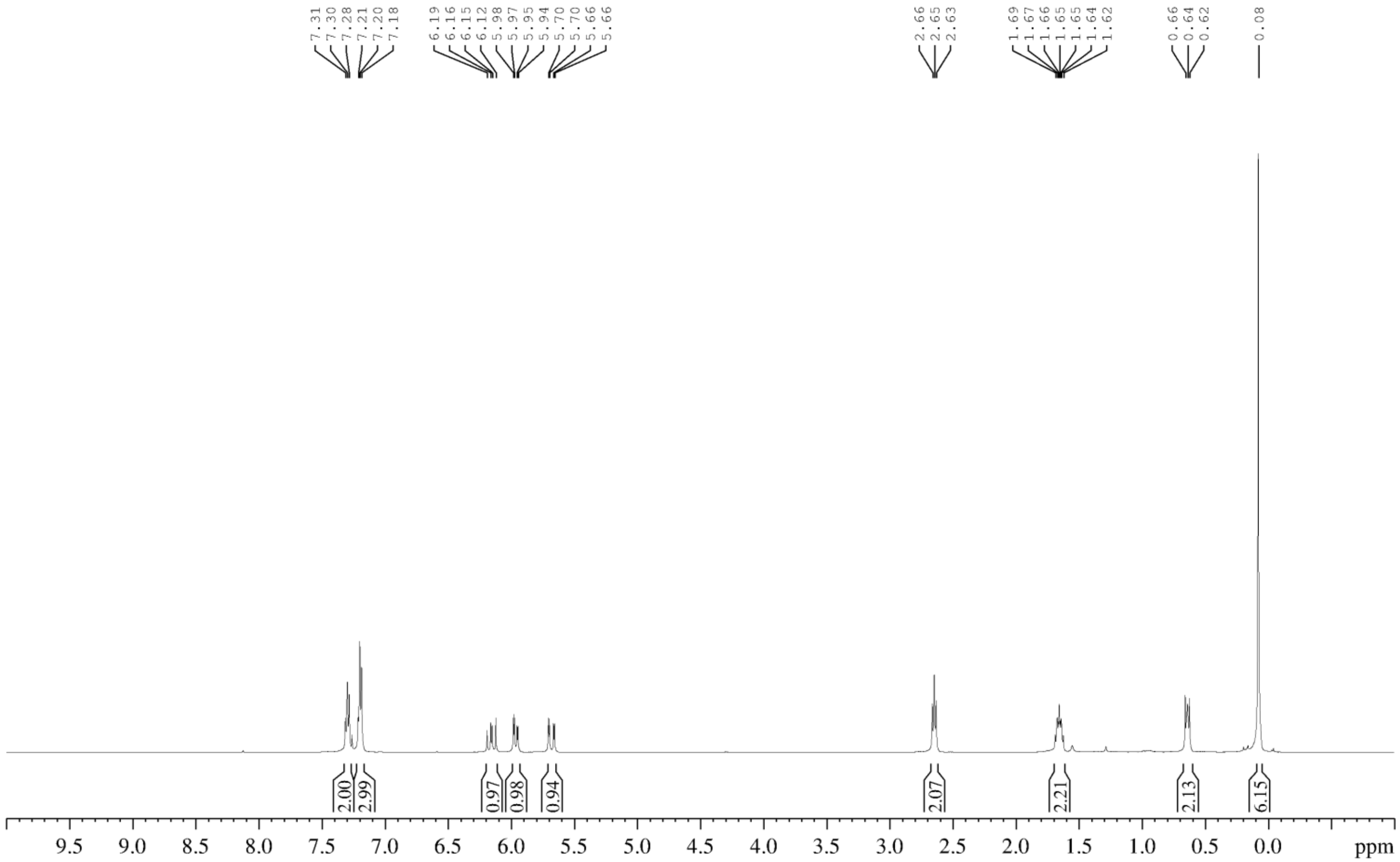

Figure S88. ^{13}C NMR (126 MHz, $CDCl_3$, 298 K) of **3ba**.

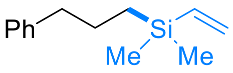

3ba

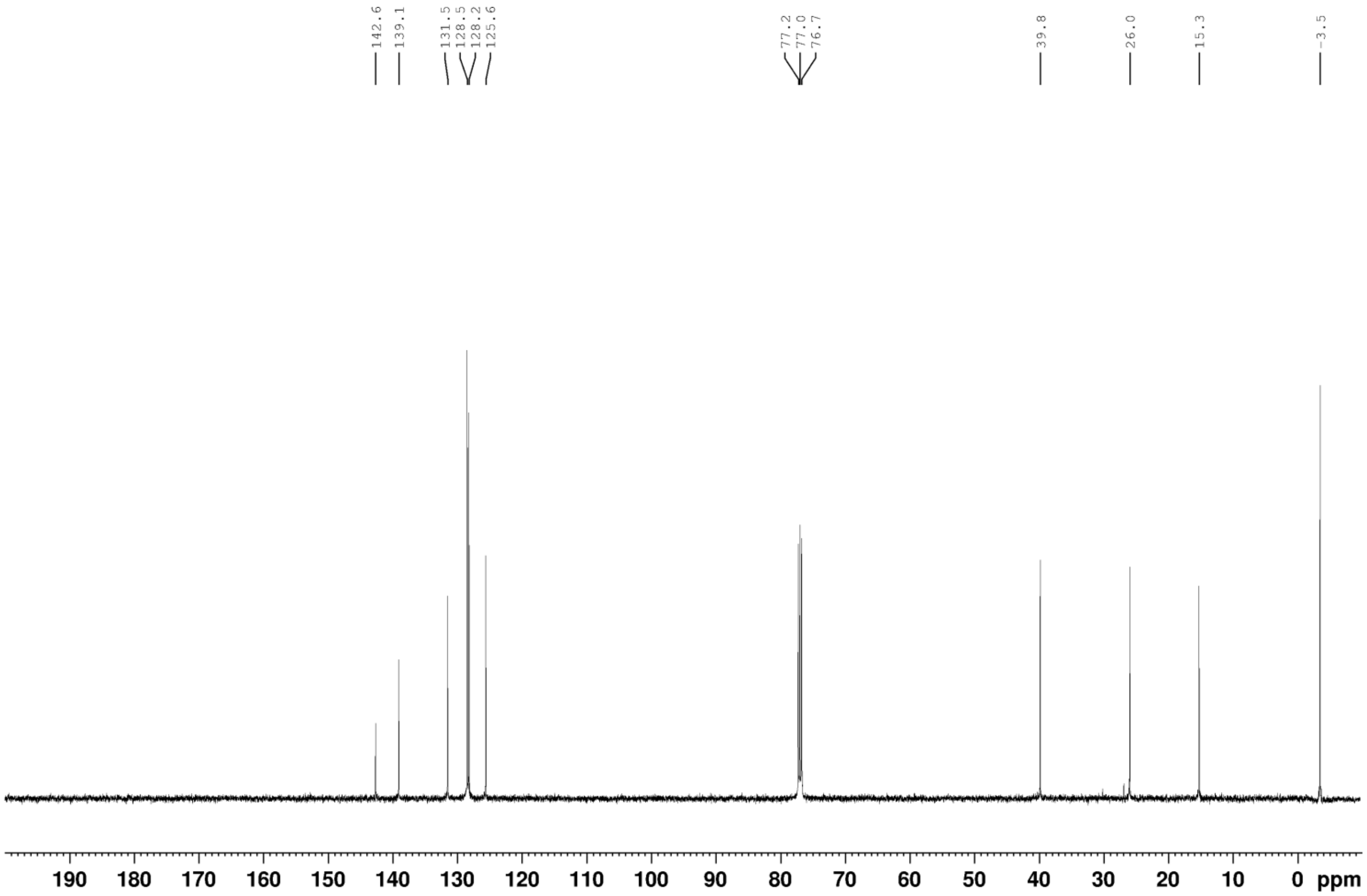

Figure S89. $^{1}H/^{29}Si$ HMQC NMR (500/99 MHz, $CDCl_3$, 298 K, optimized for J = 7 Hz) of **3ba**.

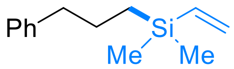

3ba

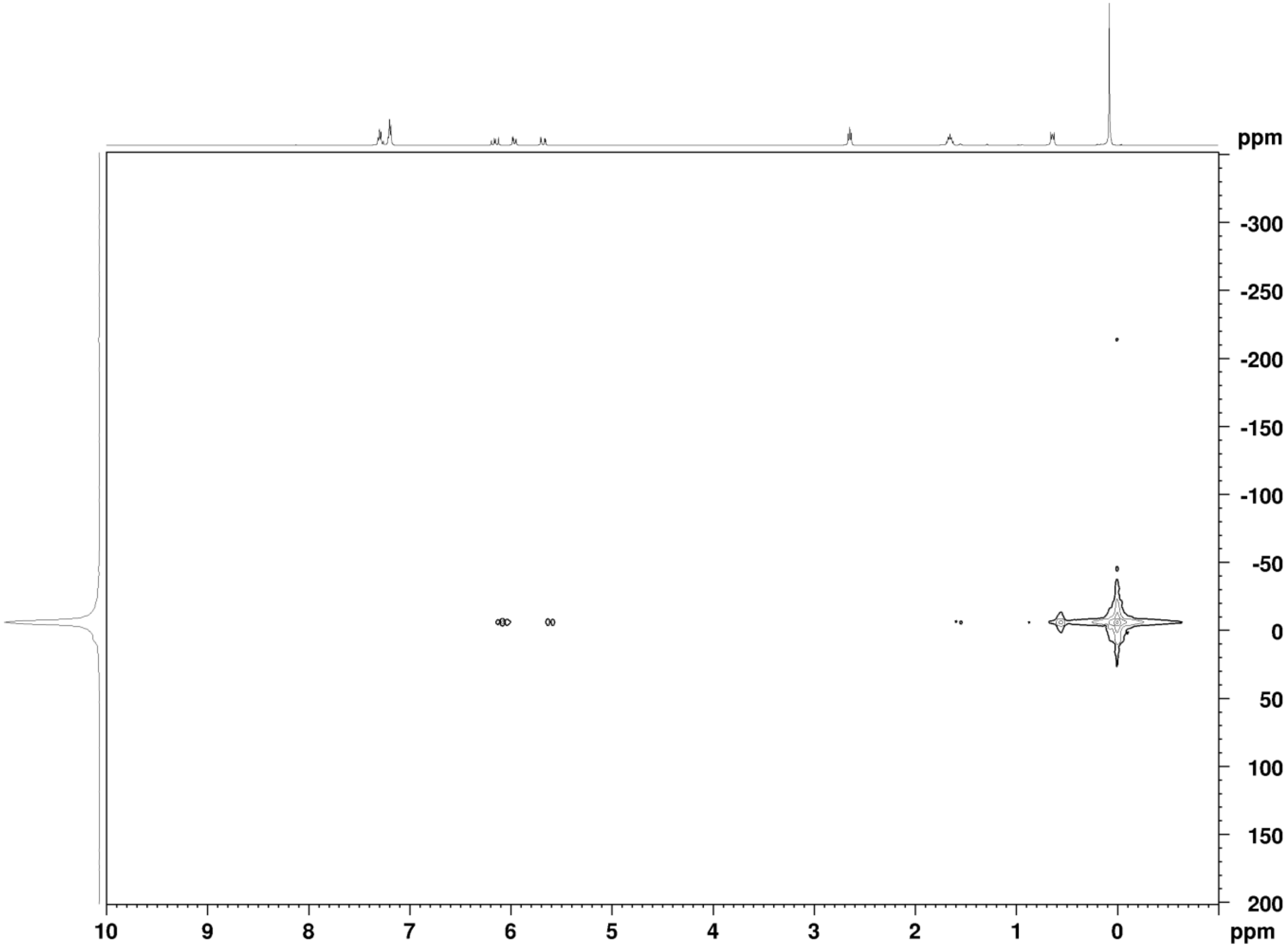

Figure S90. ^{1}H NMR (500 MHz, $CDCl_3$, 298 K) of **3ca**.

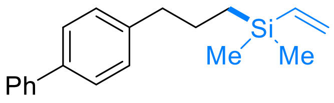

3ca

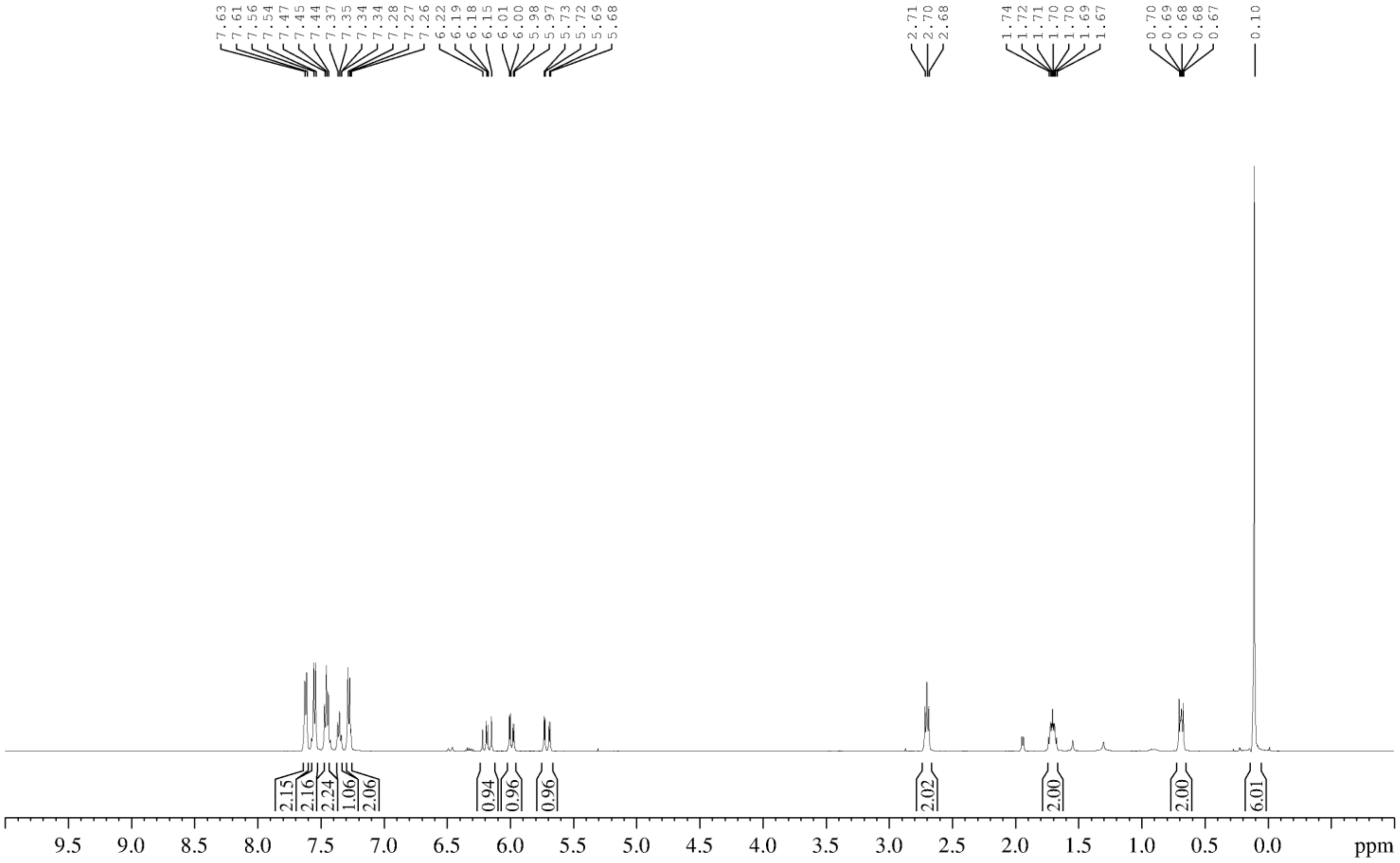

Figure S91. ^{13}C NMR (126 MHz, $CDCl_3$, 298 K) of **3ca**.

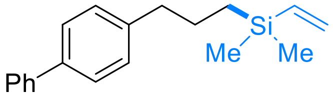

3ca

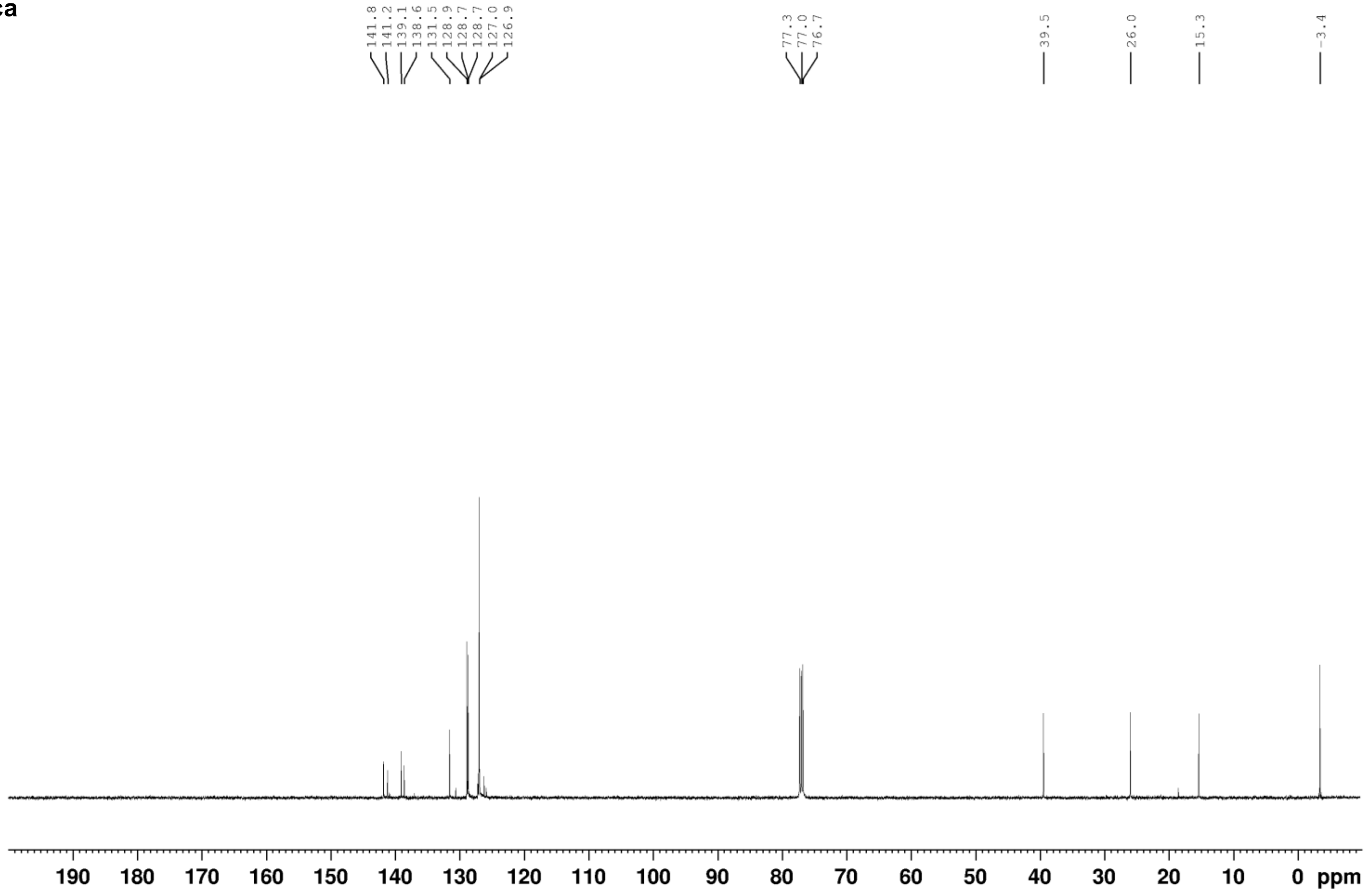

Figure S92. $^{1}H/^{29}Si$ HMQC NMR (500/99 MHz, $CDCl_3$, 298 K, optimized for J = 7 Hz) of **3ca**.

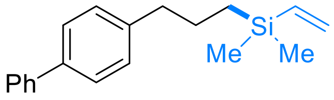

3ca

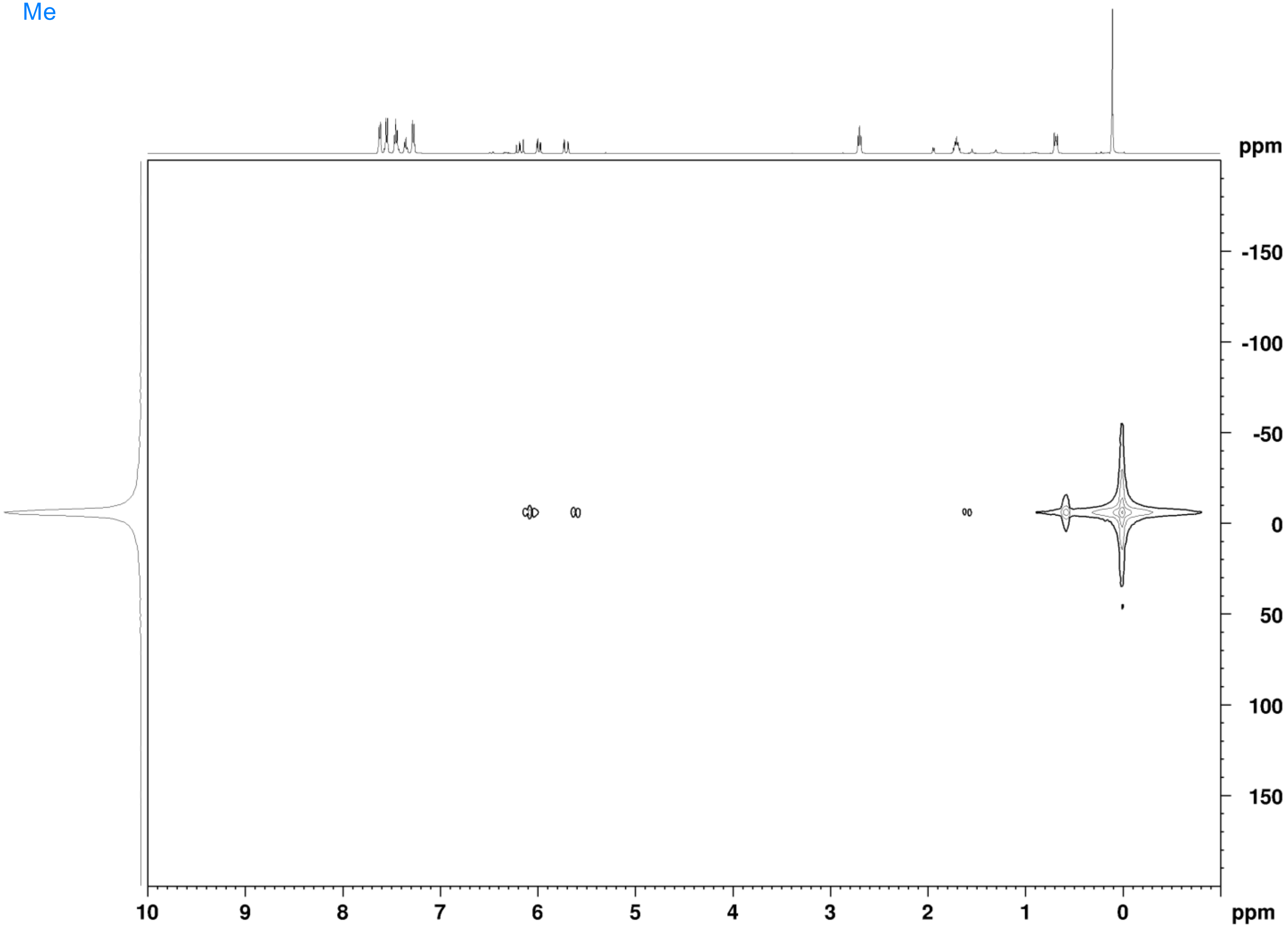

Figure S93. ^{1}H NMR (500 MHz, $CDCl_3$, 298 K) of **3da**.

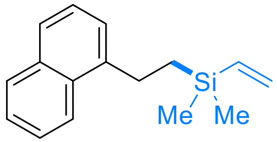

3da

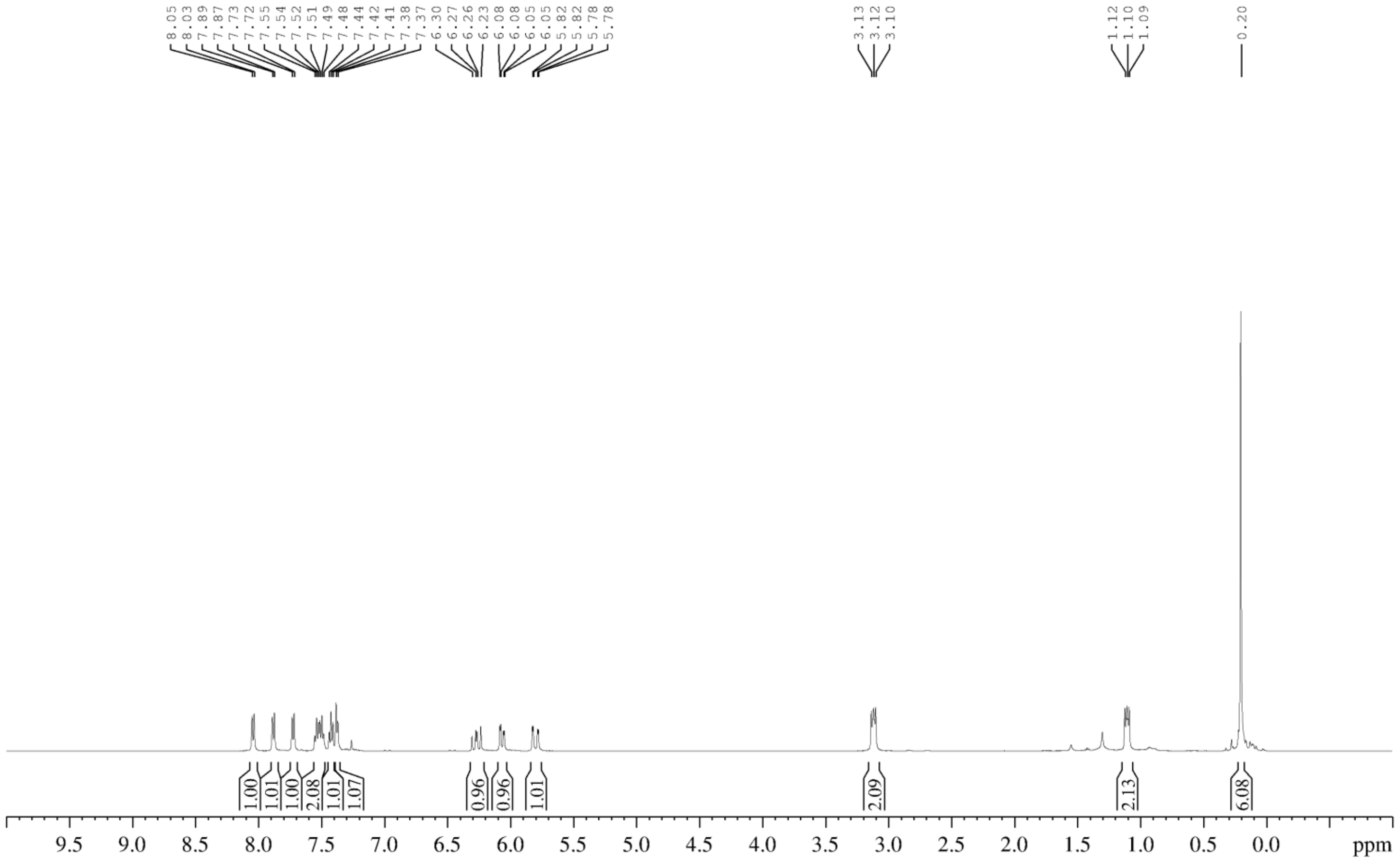

Figure S94. ^{13}C NMR (126 MHz, $CDCl_3$, 298 K) of **3da**.

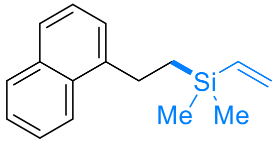

3da

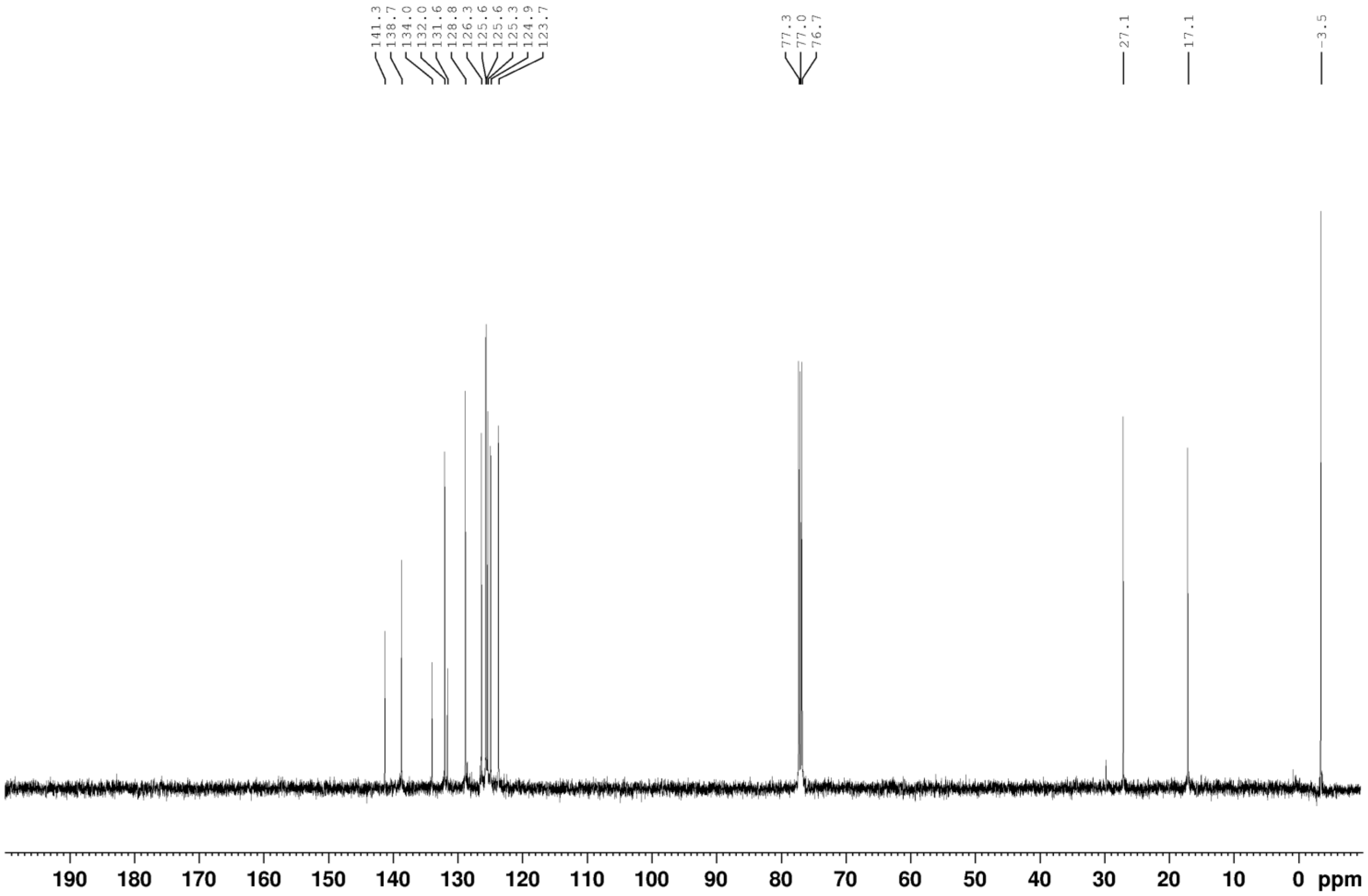

Figure S95. $^{1}H/^{29}Si$ HMQC NMR (500/99 MHz, $CDCl_3$, 298 K, optimized for *J* = 7 Hz) of **3da**.

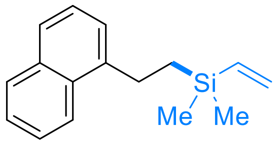

3da

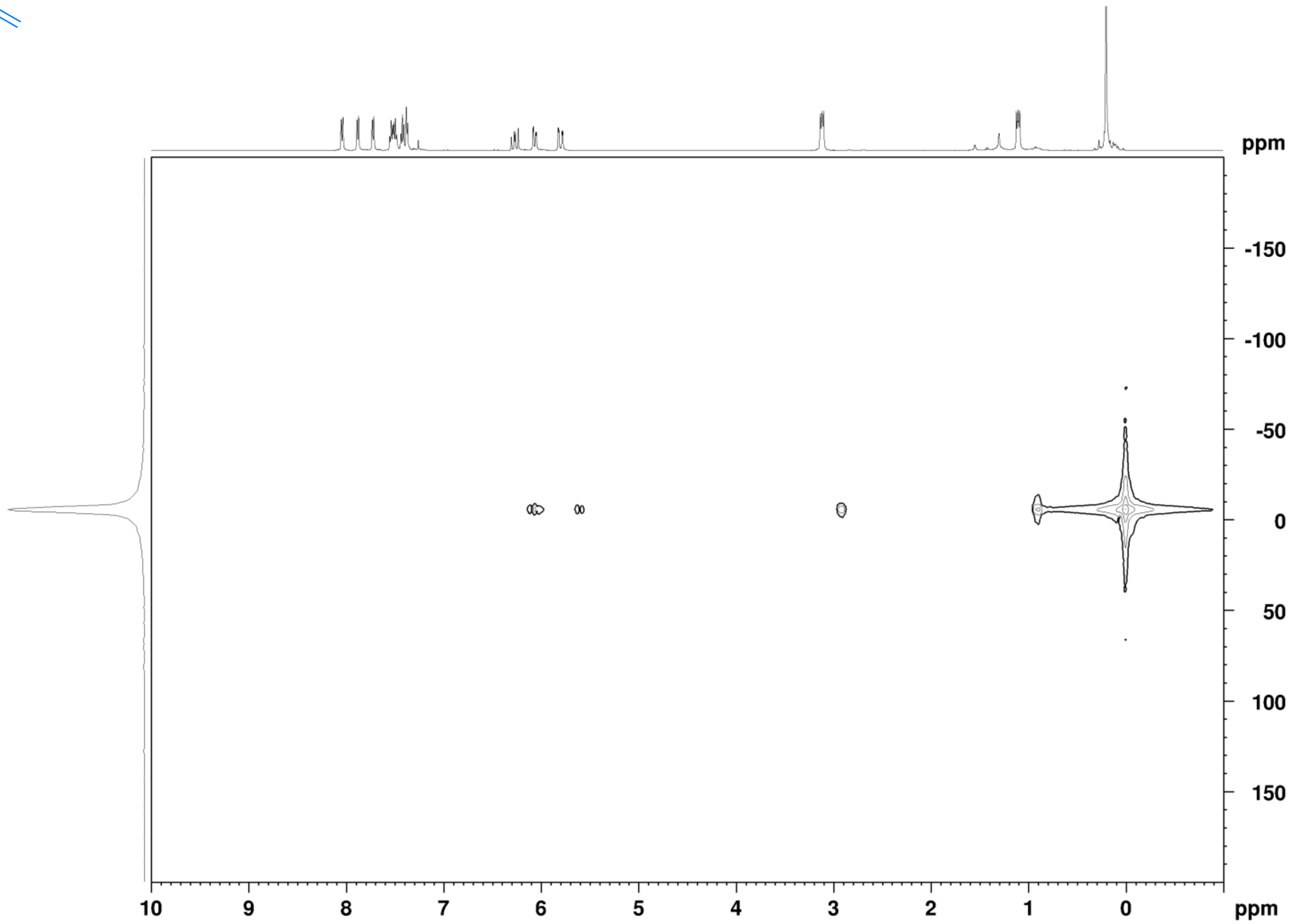

Figure S96. ^{1}H NMR (500 MHz, $CDCl_3$, 298 K) of **3ea**.

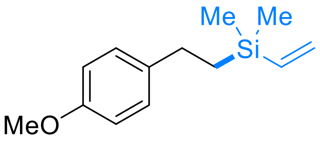

3ea

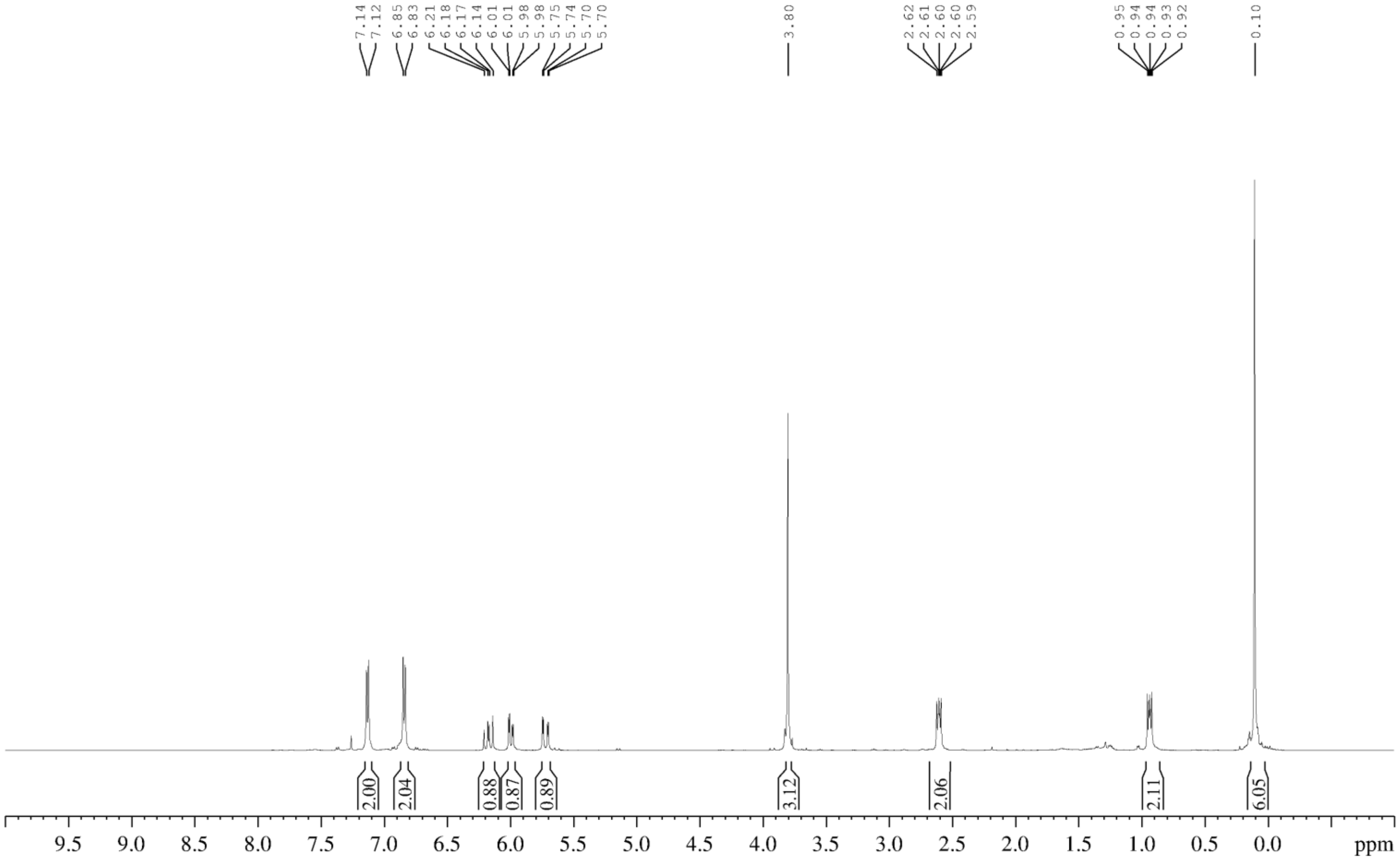

Figure S97. ^{13}C NMR (126 MHz, $CDCl_3$, 298 K) of **3ea**.

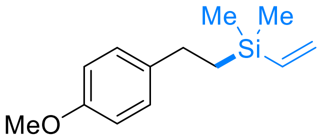

3ea

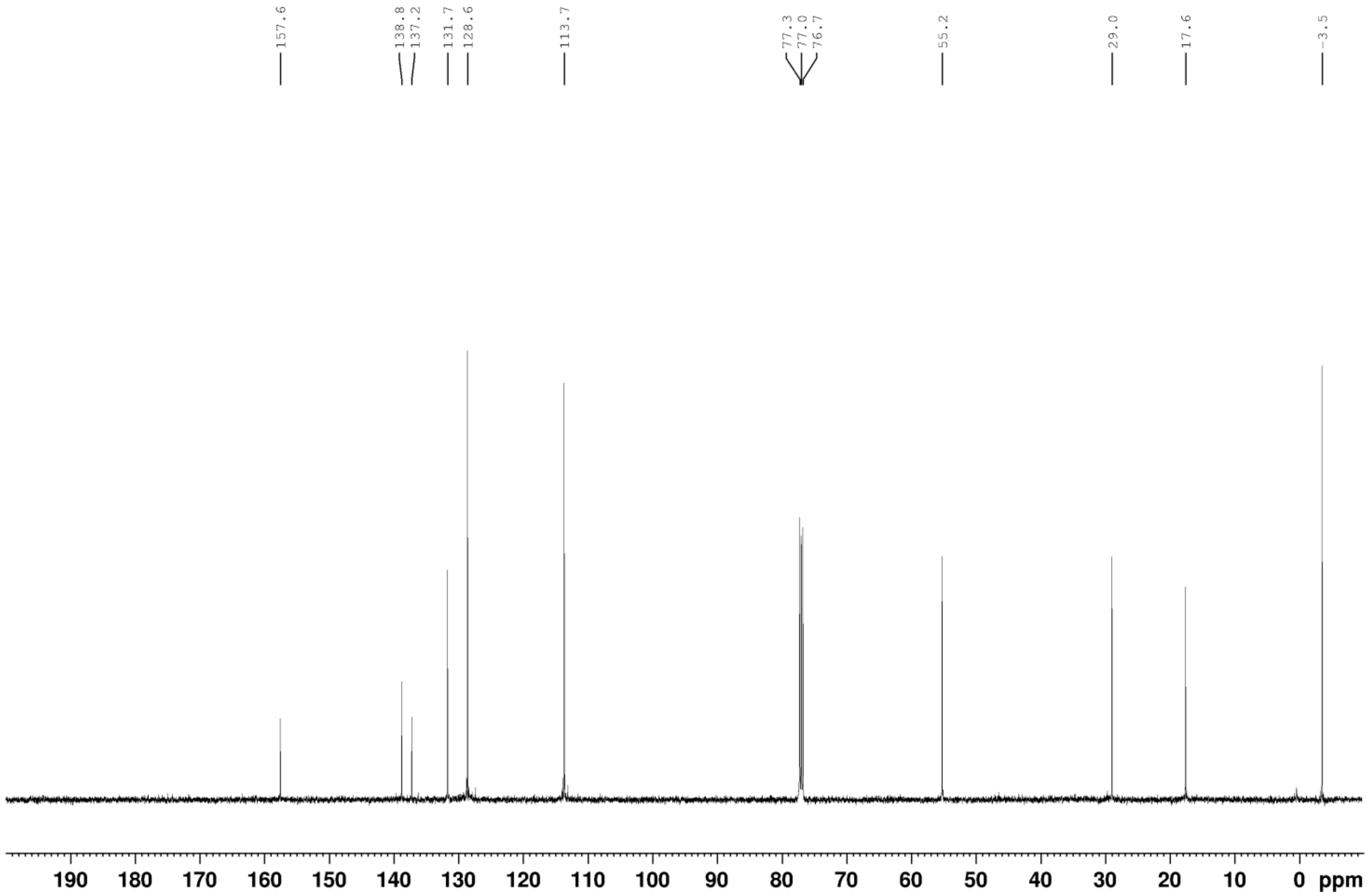

Figure S98. $^{1}H/^{29}Si$ HMQC NMR (500/99 MHz, $CDCl_3$, 298 K, optimized for J = 7 Hz) of **3ea**.

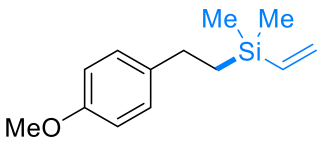

3ea

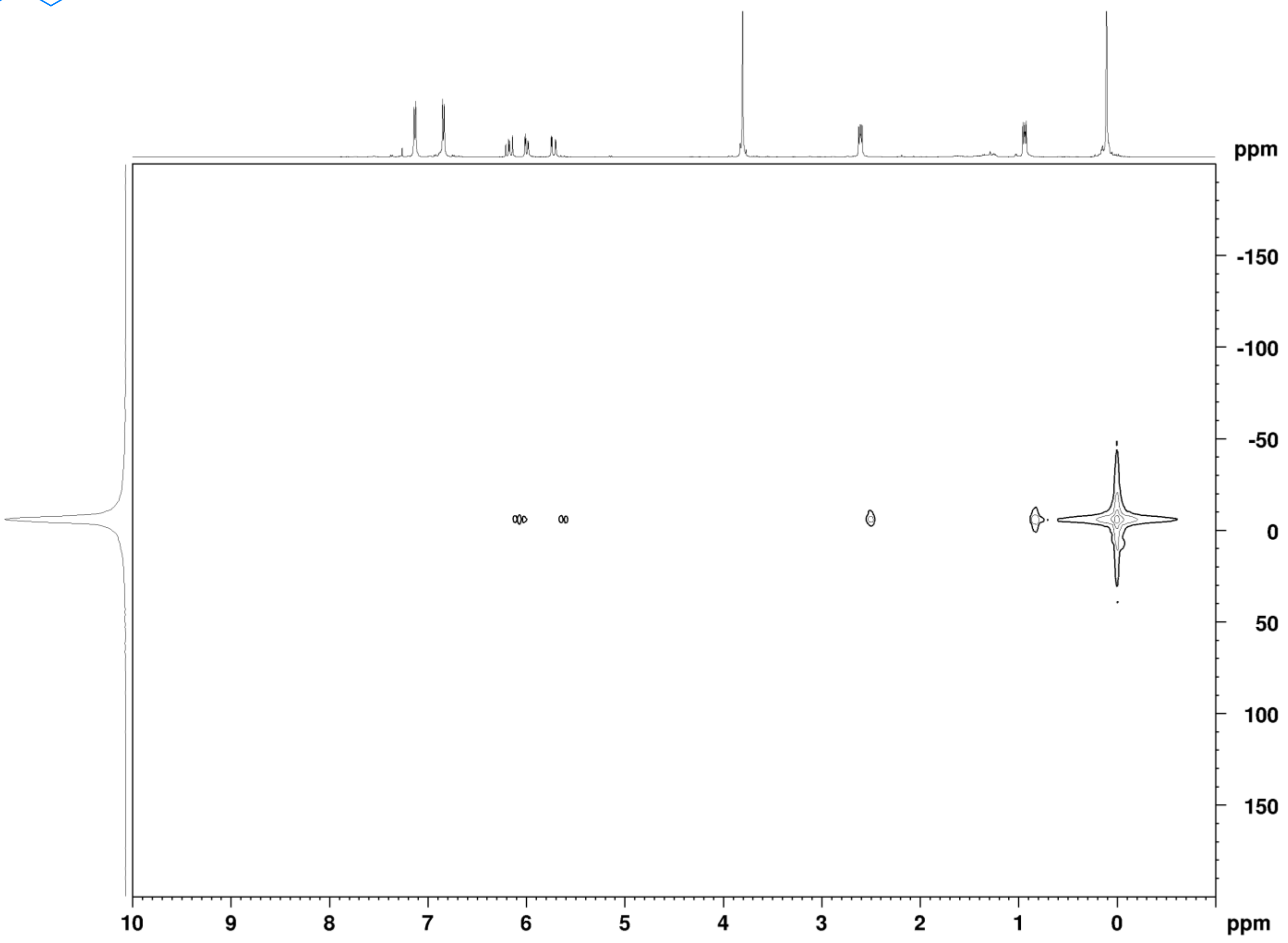

Figure S99. ^{1}H NMR (500 MHz, $CDCl_3$, 298 K) of **3fa**.

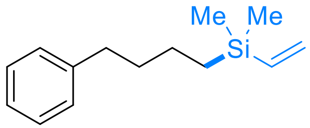

3fa

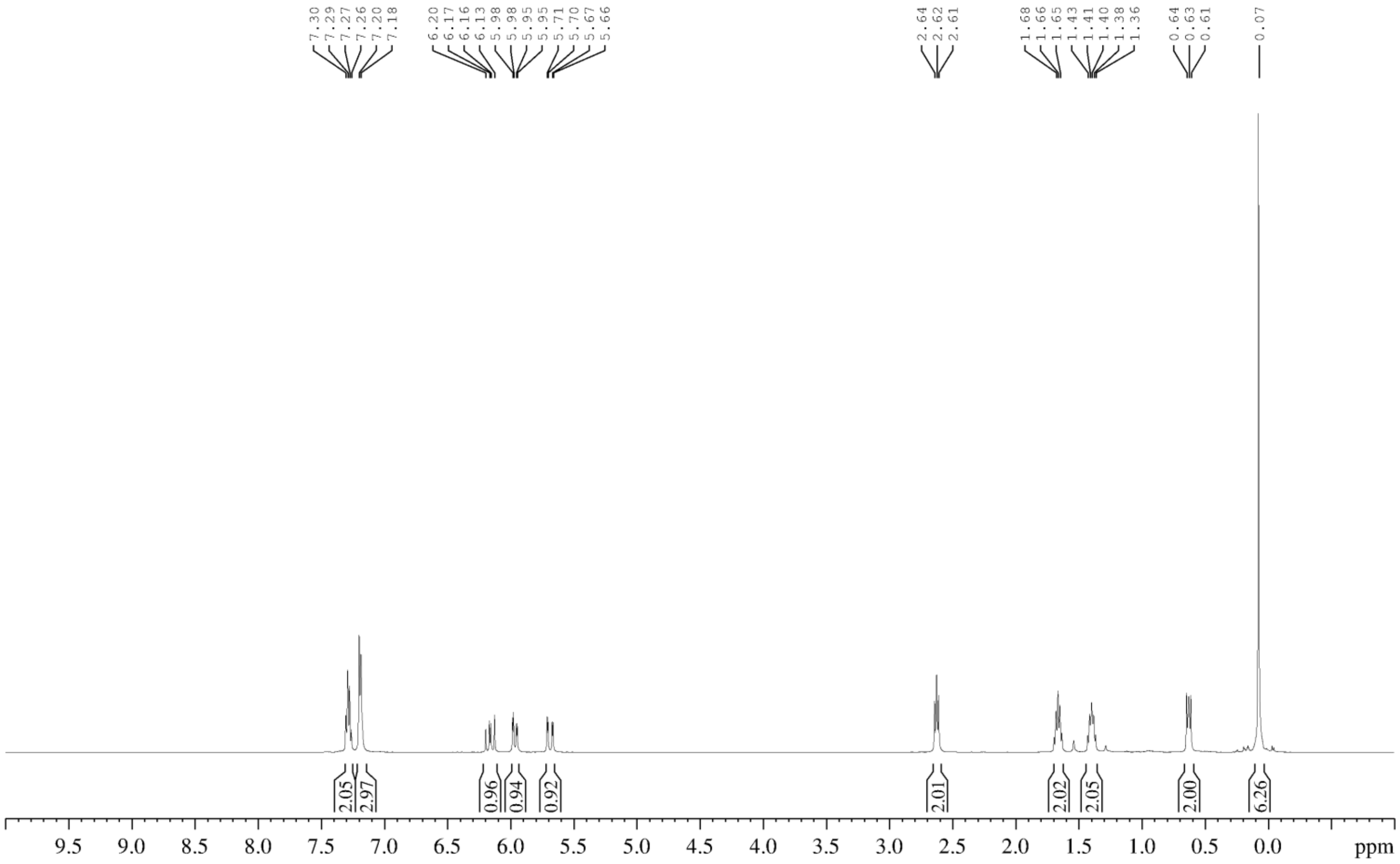

Figure S100. ^{13}C NMR (126 MHz, $CDCl_3$, 298 K) of **3fa**.

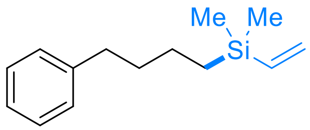

3fa

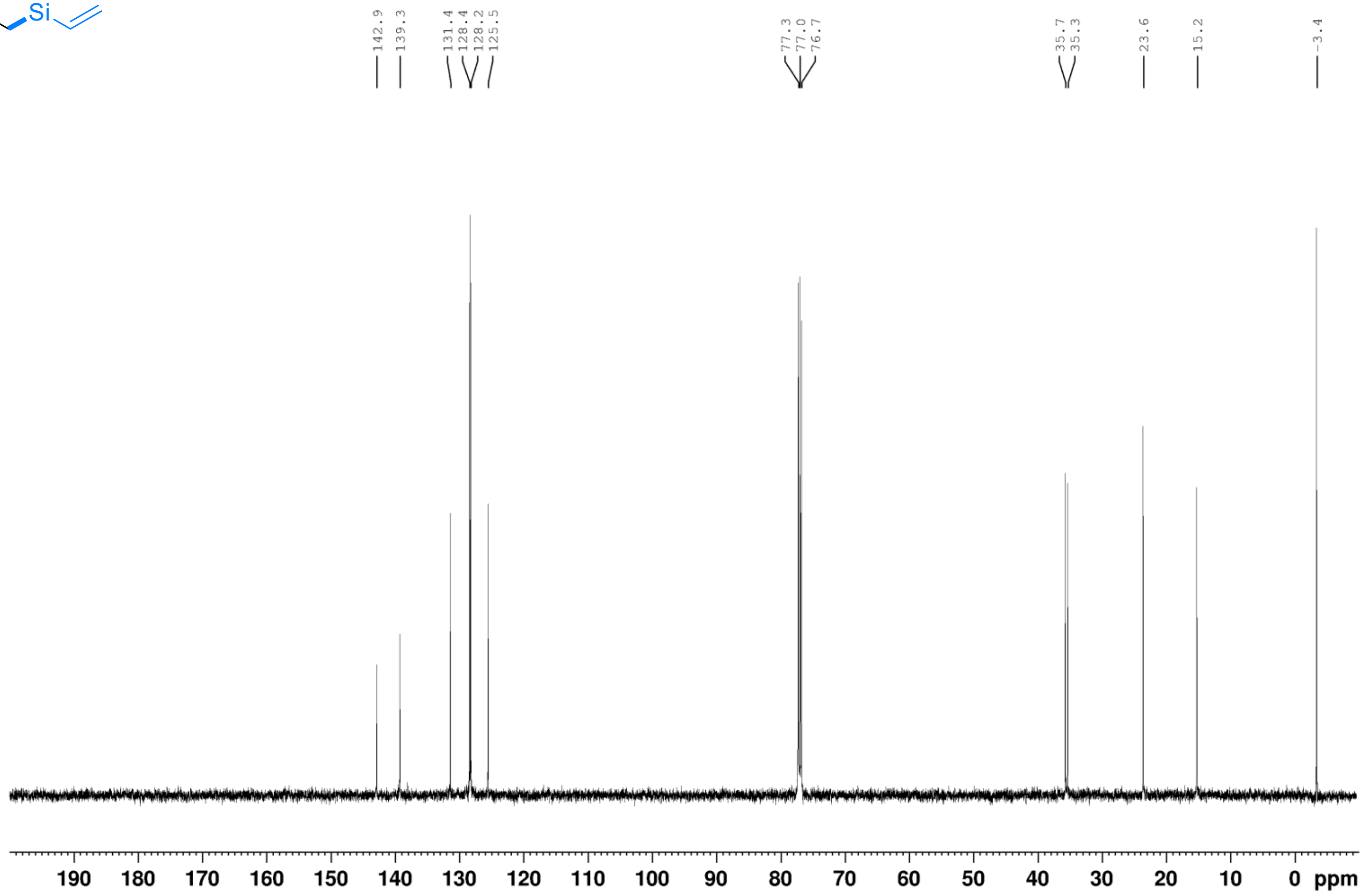

Figure S101. $^{1}H/^{29}Si$ HMQC NMR (500/99 MHz, $CDCl_3$, 298 K, optimized for J = 7 Hz) of **3fa**.

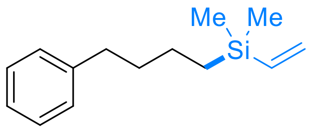

3fa

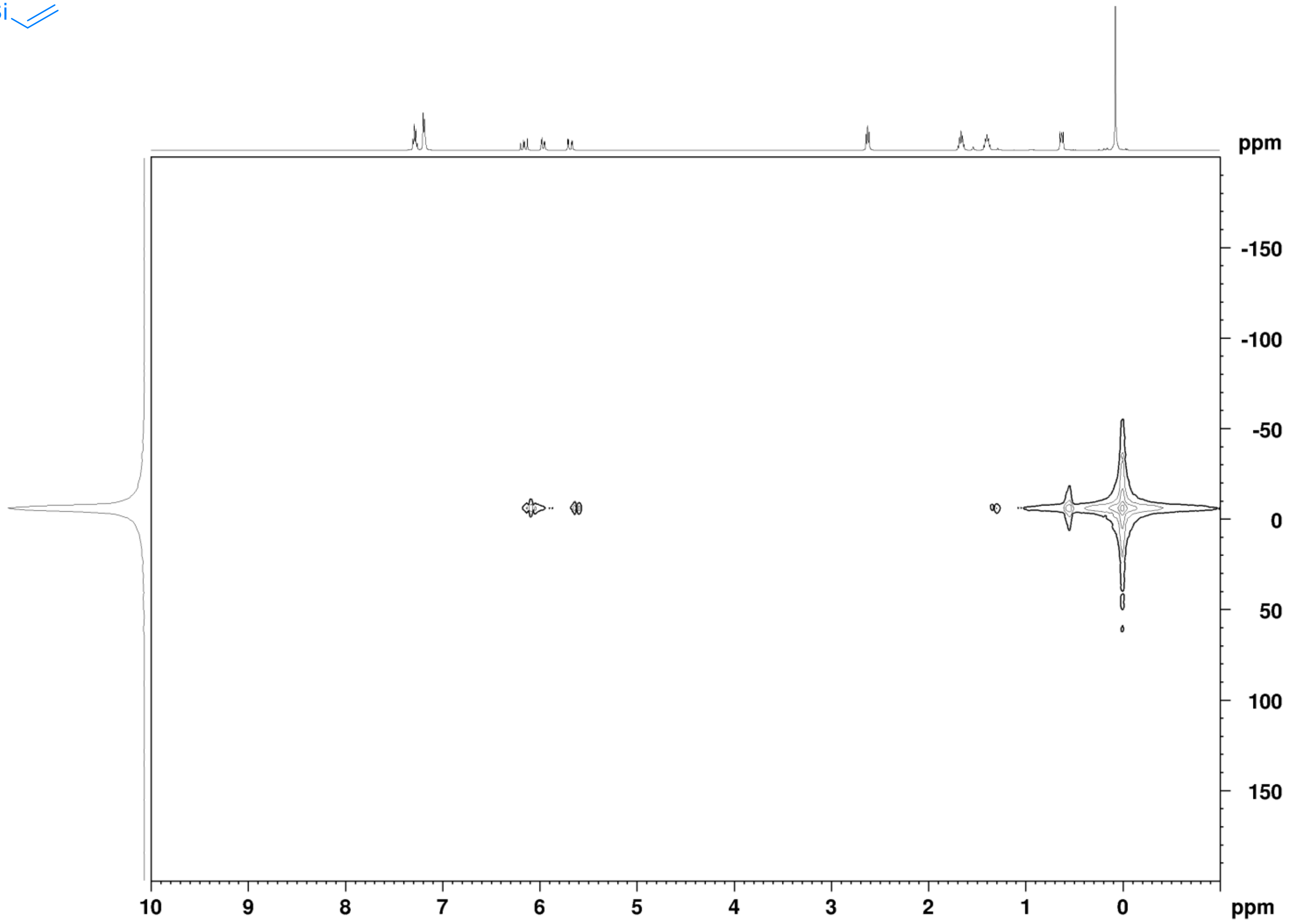

Figure S102. ^{1}H NMR (500 MHz, $CDCl_3$, 298 K) of **3ga**.

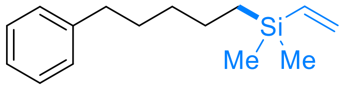

3ga

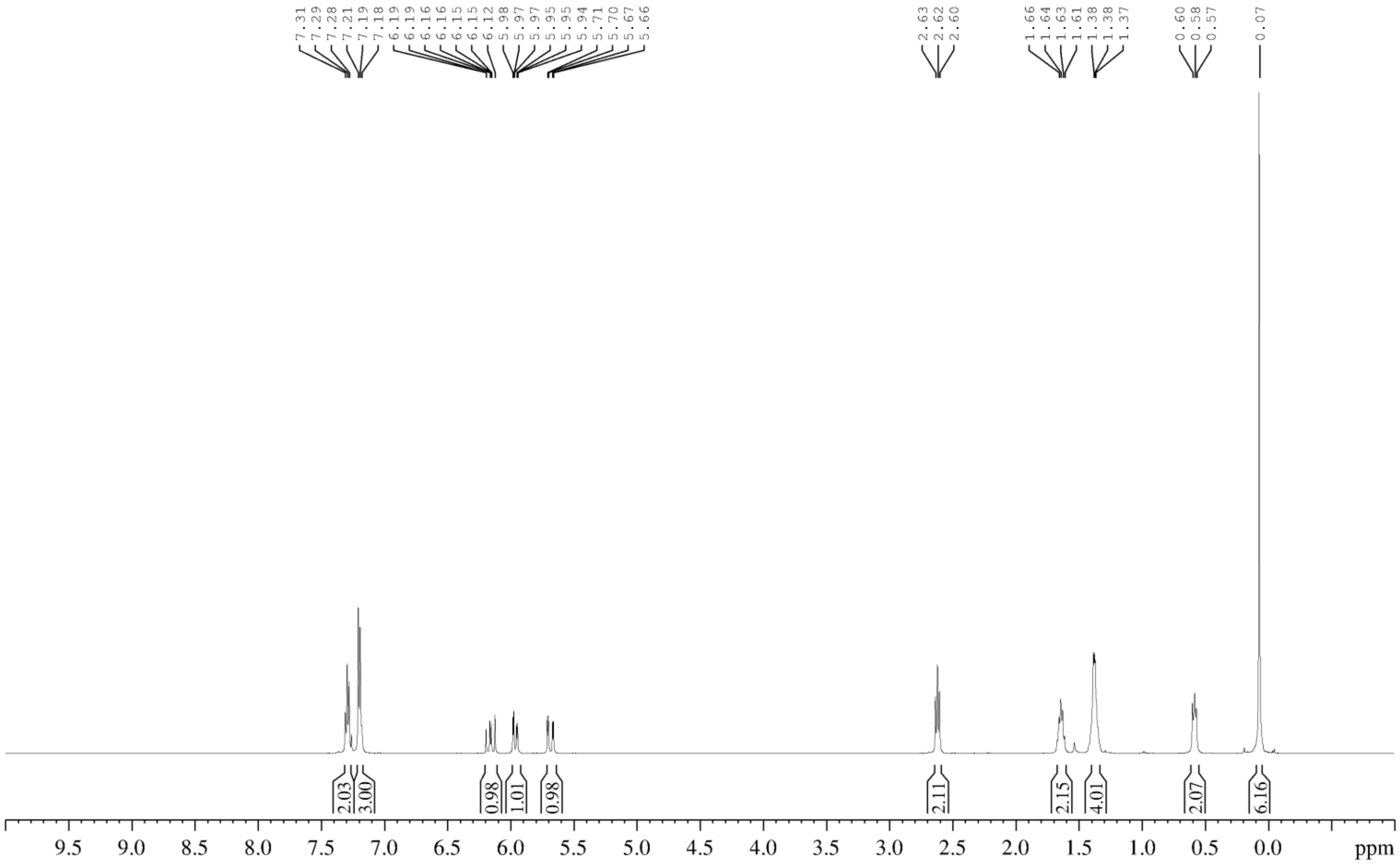

Figure S103. ^{13}C NMR (126 MHz, $CDCl_3$, 298 K) of **3ga**.

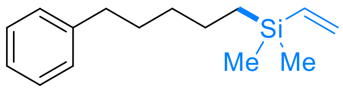

3ga

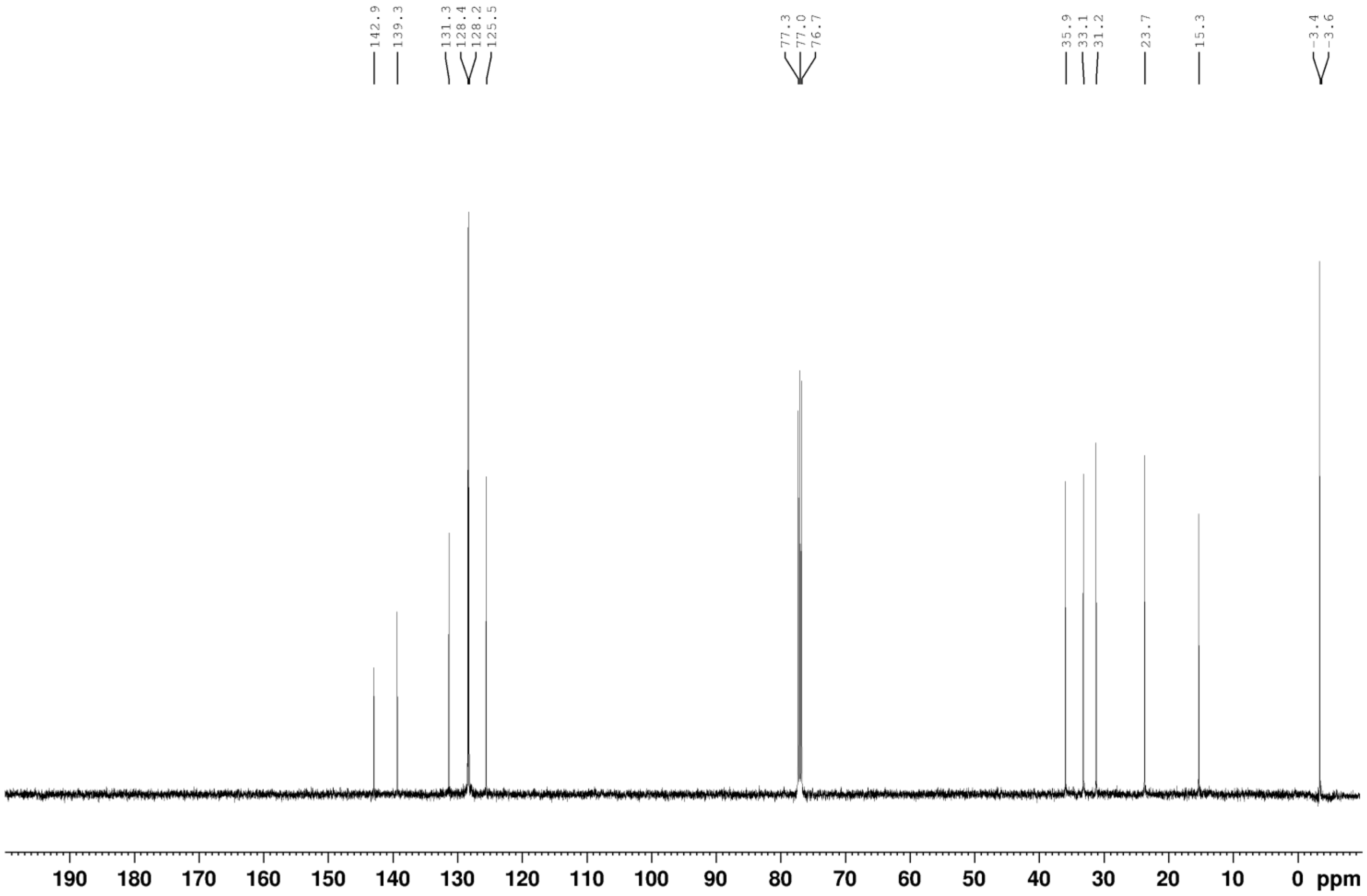

Figure S104. $^{1}H/^{29}Si$ HMQC NMR (500/99 MHz, $CDCl_3$, 298 K, optimized for J = 7 Hz) of **3ga**.

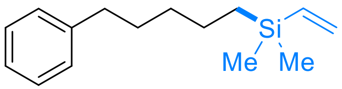

3ga

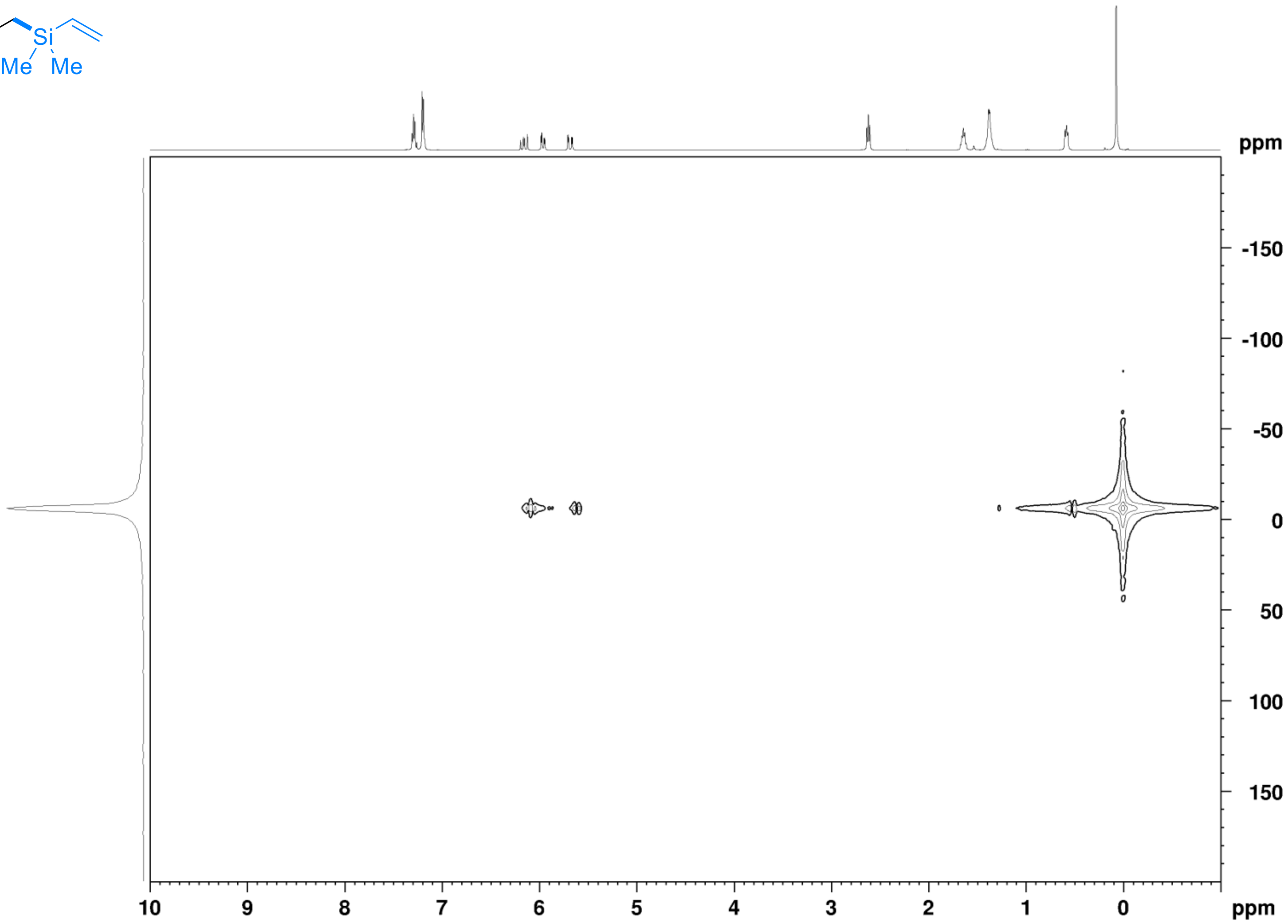

Figure S105. ^{1}H NMR (500 MHz, $CDCl_3$, 298 K) of **3ha**.

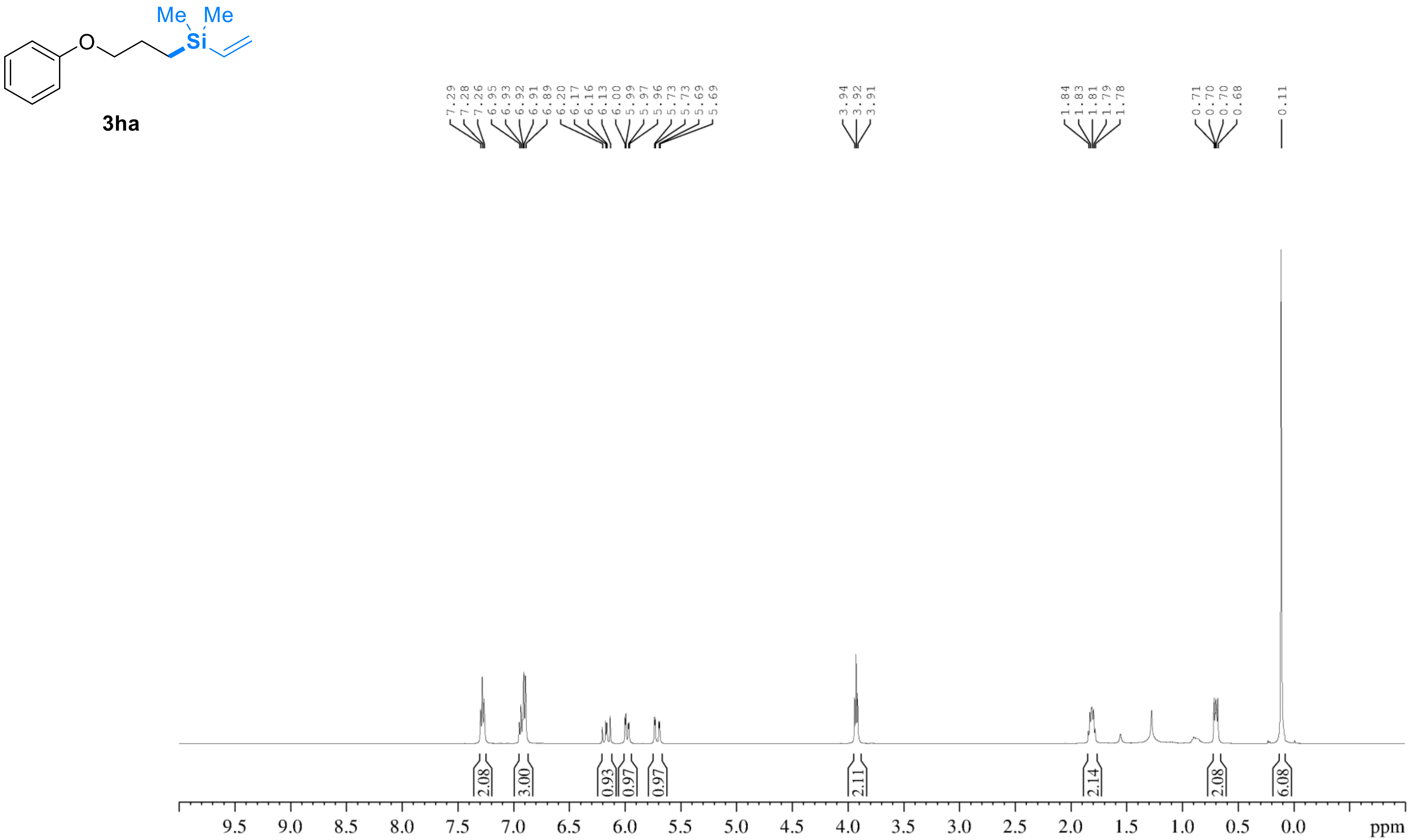

Figure S106. ^{13}C NMR (126 MHz, $CDCl_3$, 298 K) of **3ha**.

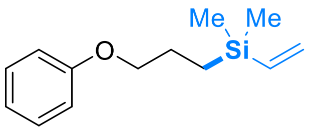

3ha

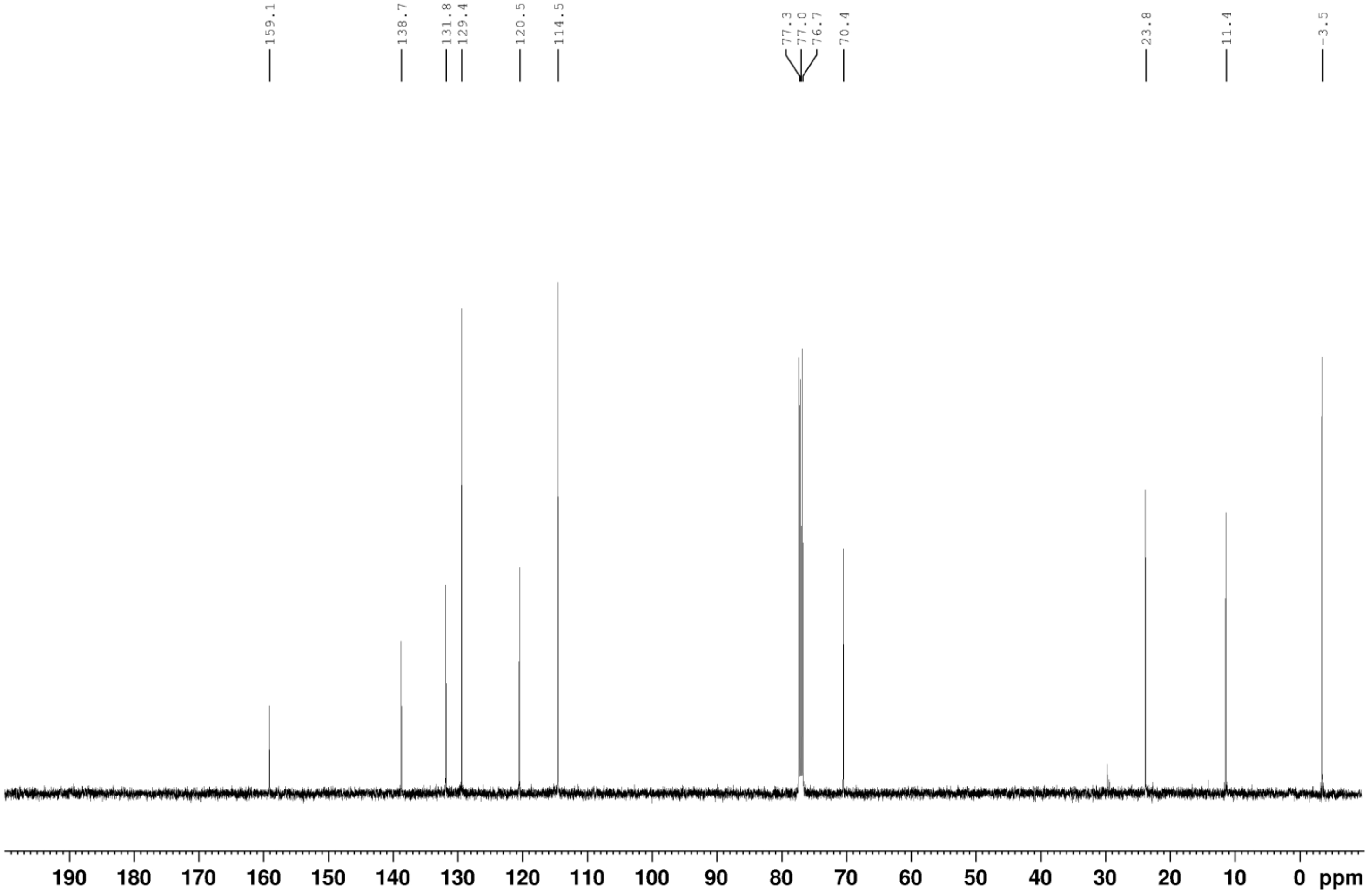

Figure S107. $^{1}H/^{29}Si$ HMQC NMR (500/99 MHz, $CDCl_3$, 298 K, optimized for J = 7 Hz) of **3ha**.

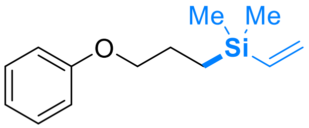

3ha

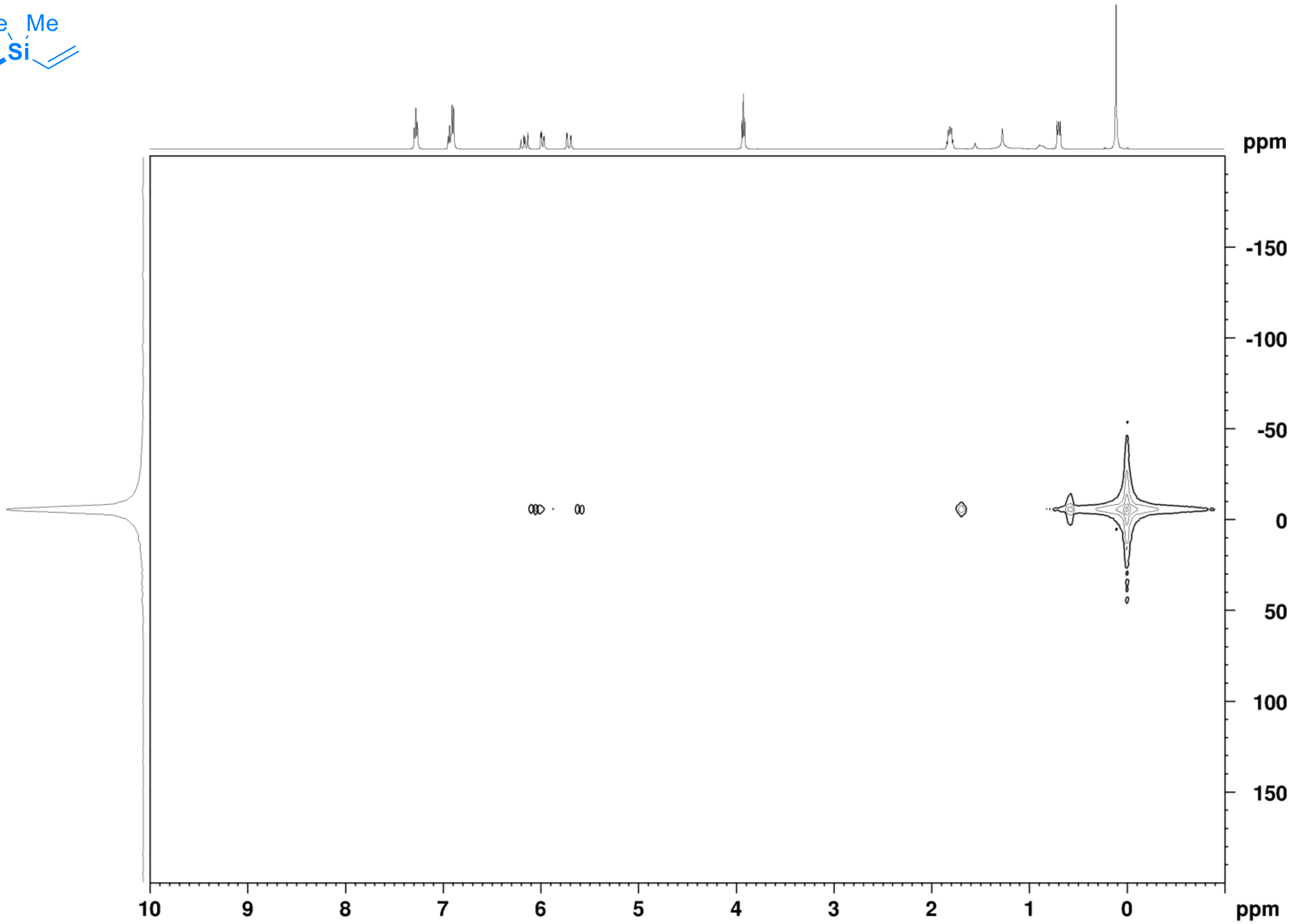

Figure S108. ^{1}H NMR (500 MHz, $CDCl_3$, 298 K) of **3ia**.

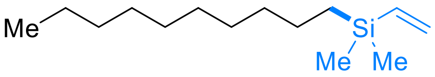

3ia

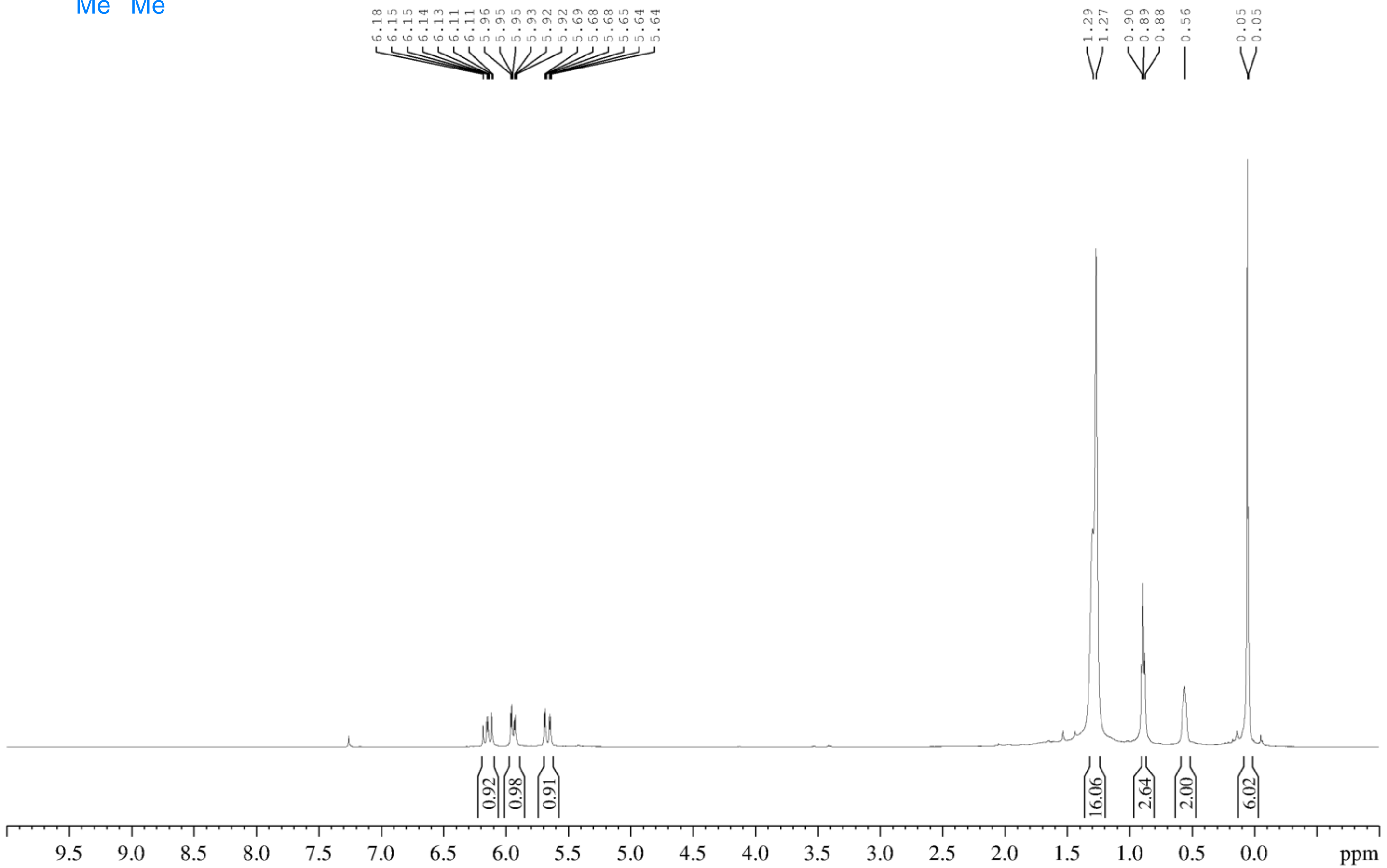

Figure S109. ^{13}C NMR (126 MHz, $CDCl_3$, 298 K) of **3ia**.

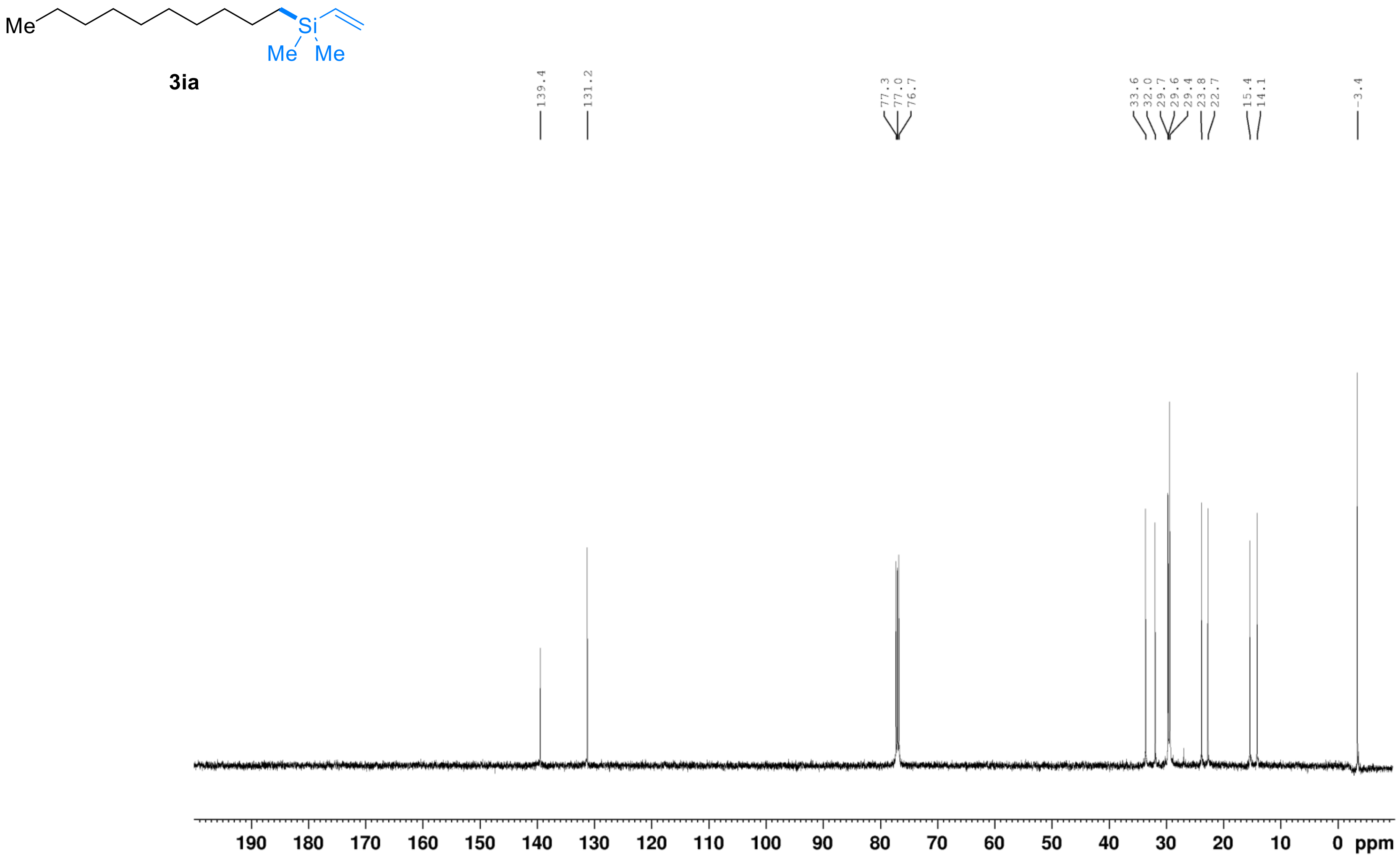

Figure S110. $^{1}H/^{29}Si$ HMQC NMR (500/99 MHz, $CDCl_3$, 298 K, optimized for J = 7 Hz) of **3ia**.

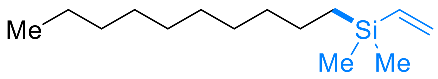

3ia

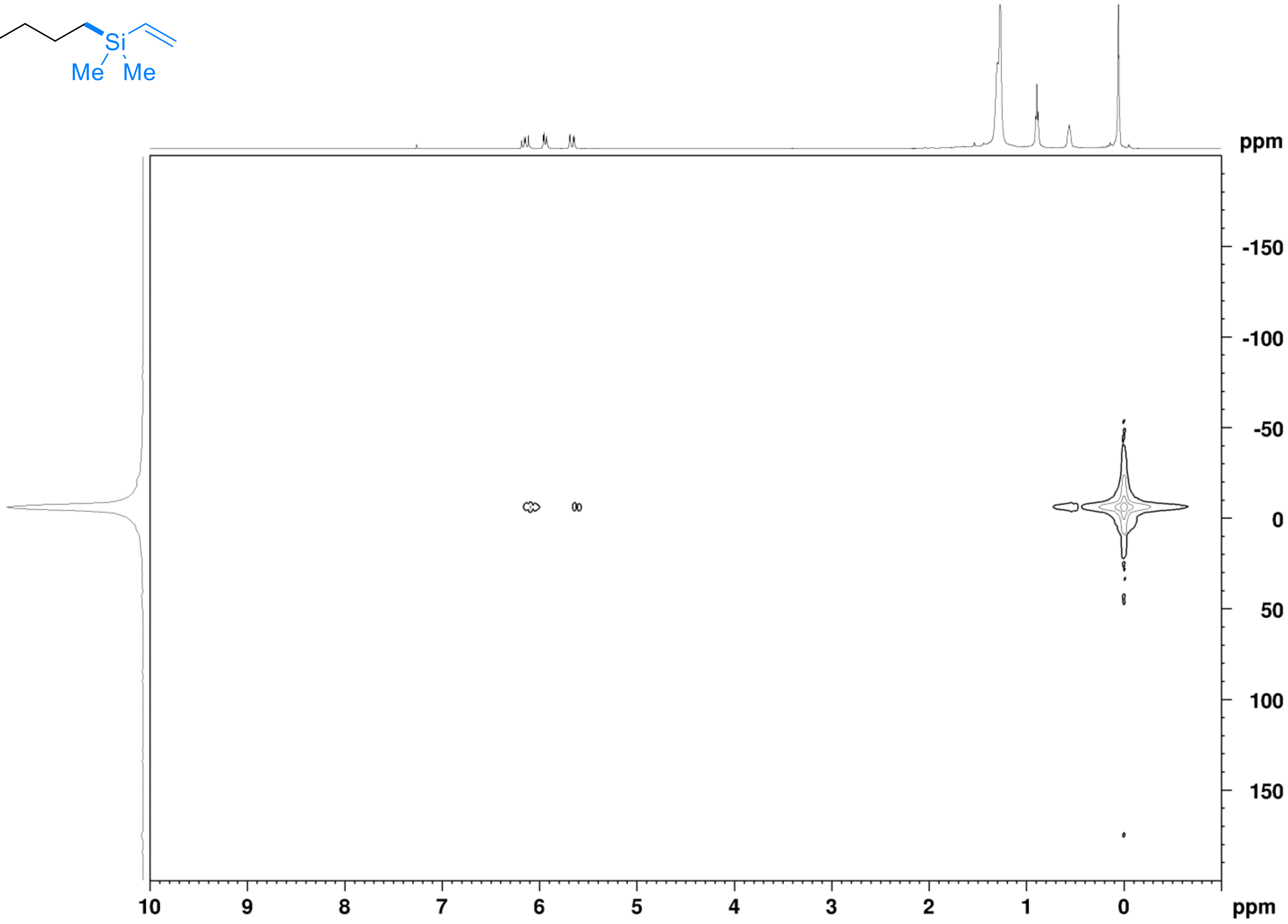

Figure S111. ^{1}H NMR (500 MHz, $CDCl_3$, 298 K) of **3ja**.

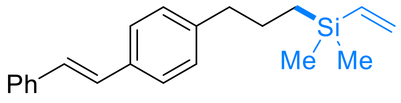

3ja

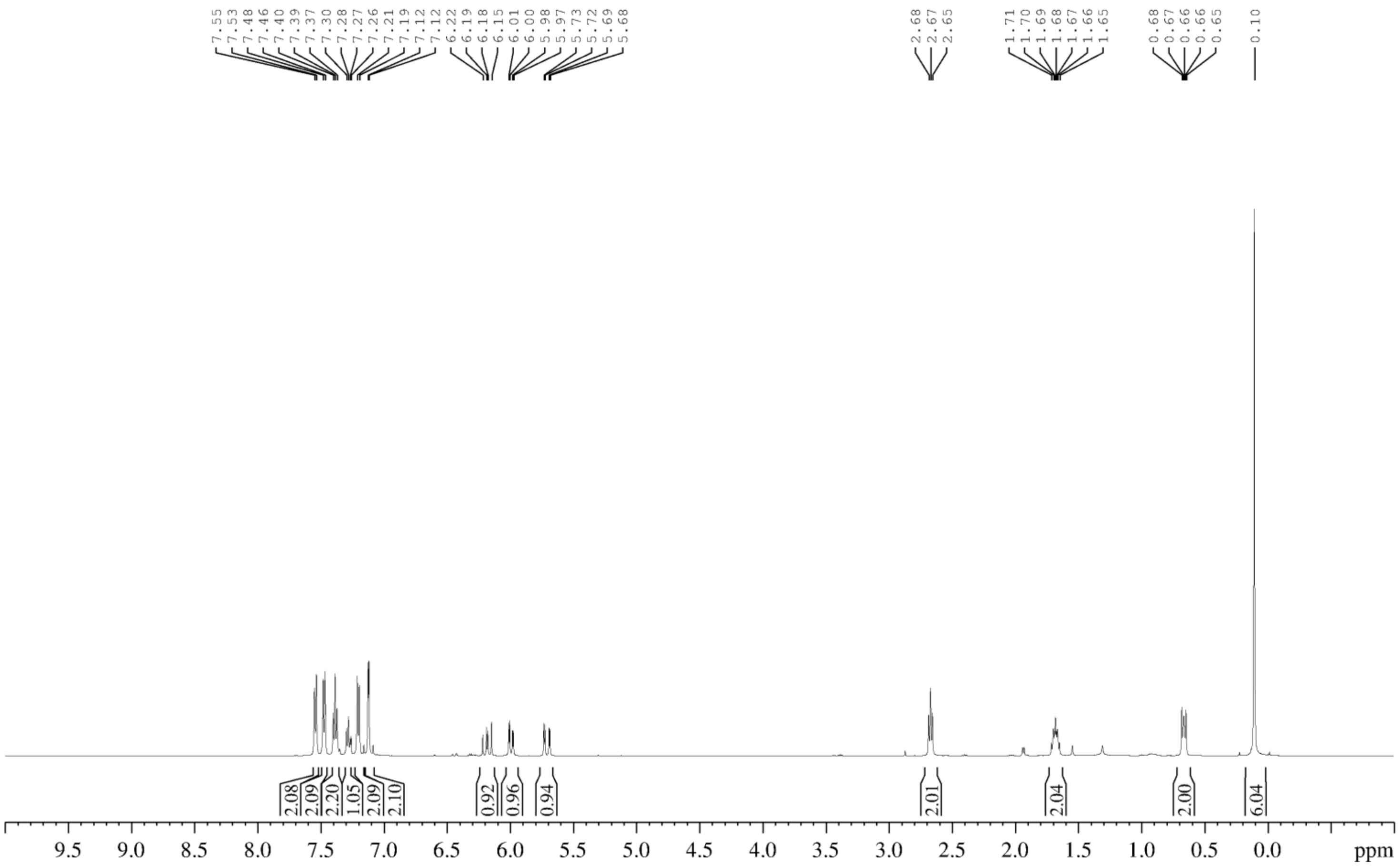

Figure S112. ^{13}C NMR (126 MHz, $CDCl_3$, 298 K) of **3ja**.

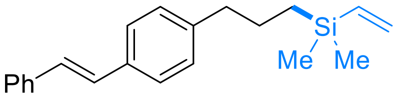

3ja

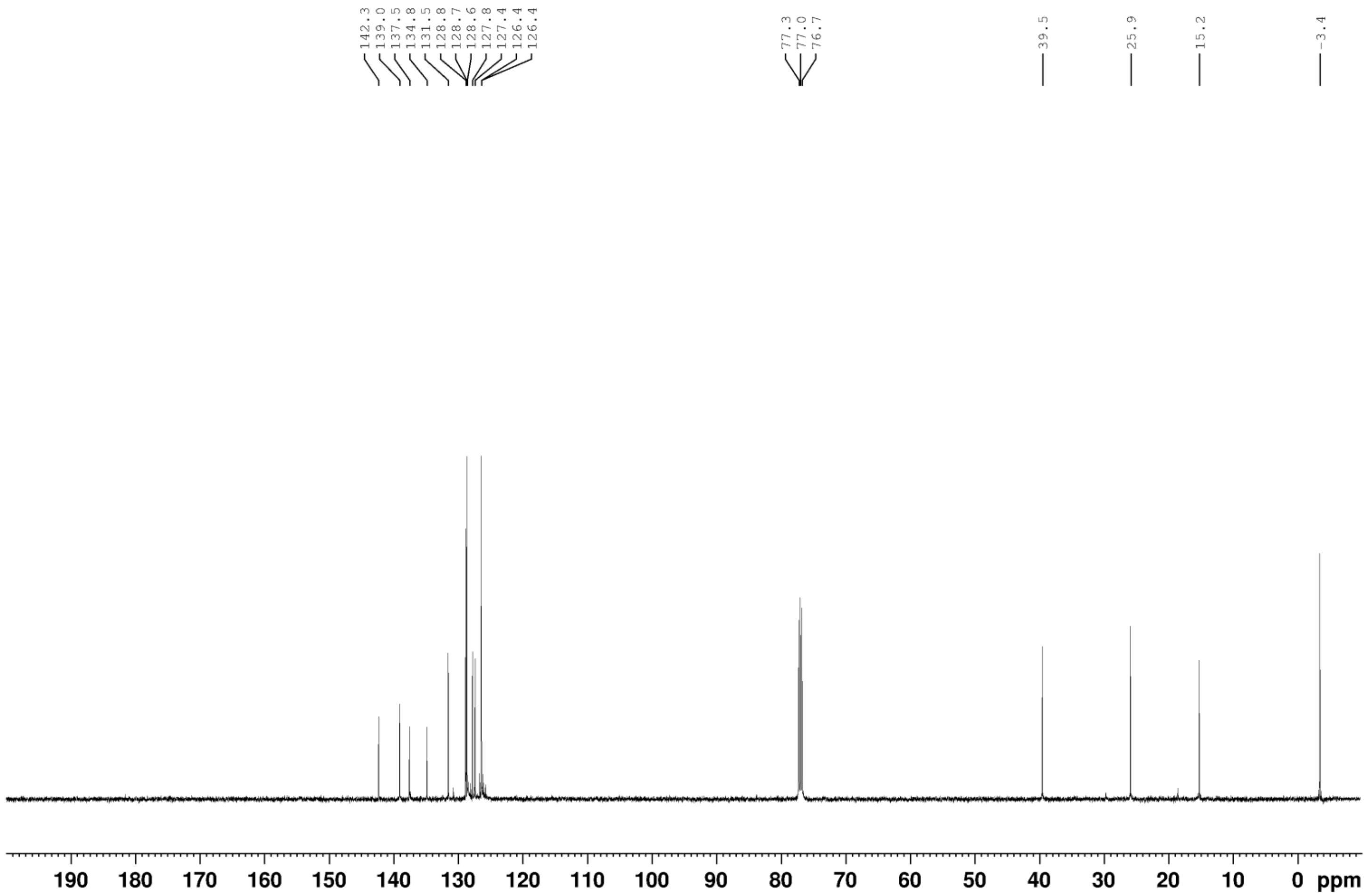

Figure S113. ^{1}H/^{29}Si HMQC NMR (500/99 MHz, $CDCl_3$, 298 K, optimized for J = 7 Hz) of **3ja**.

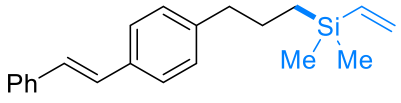

3ja

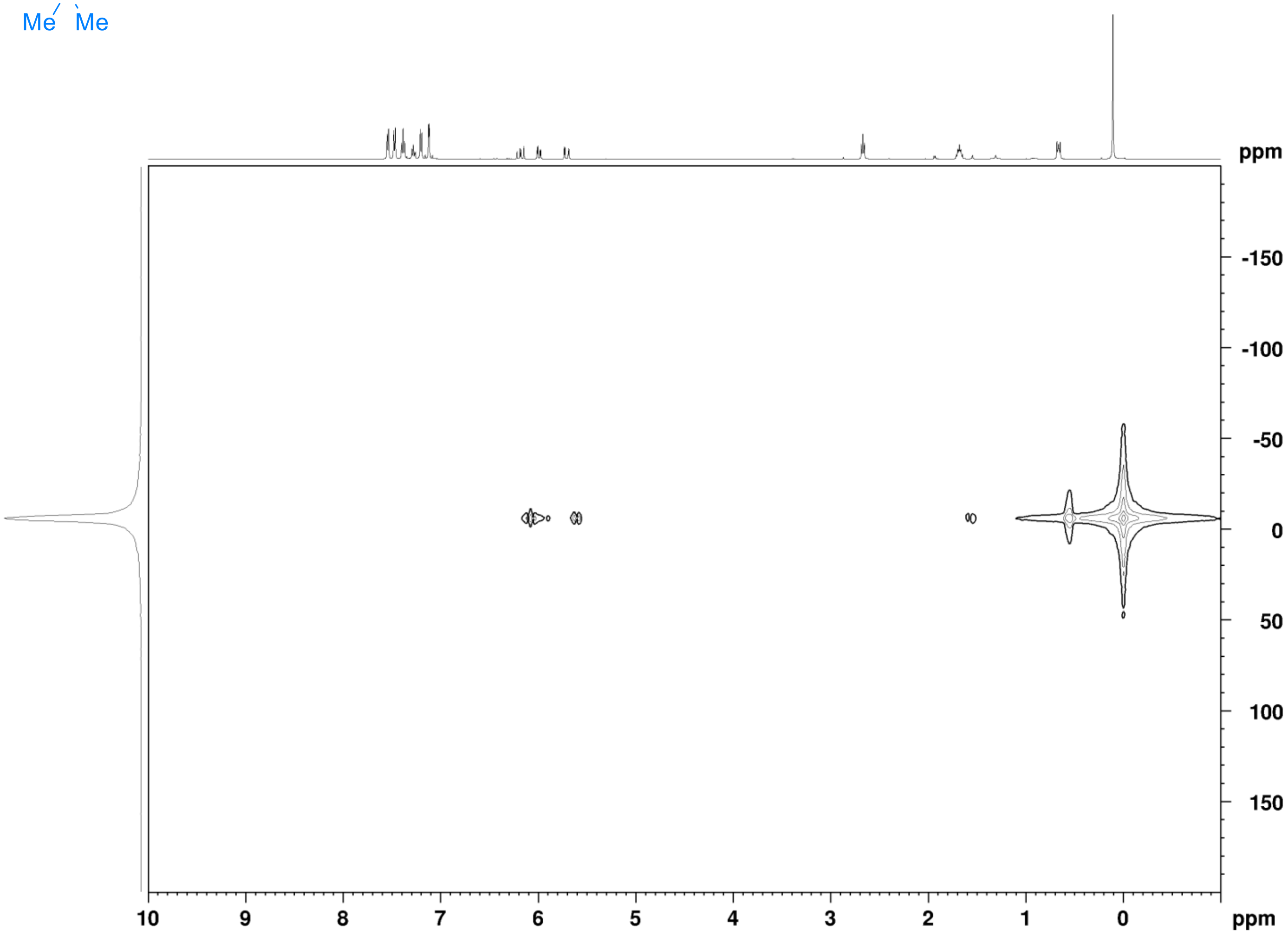

Figure S114. ^{1}H NMR (500 MHz, $CDCl_3$, 298 K) of **3ka**.

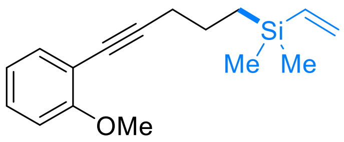

3ka

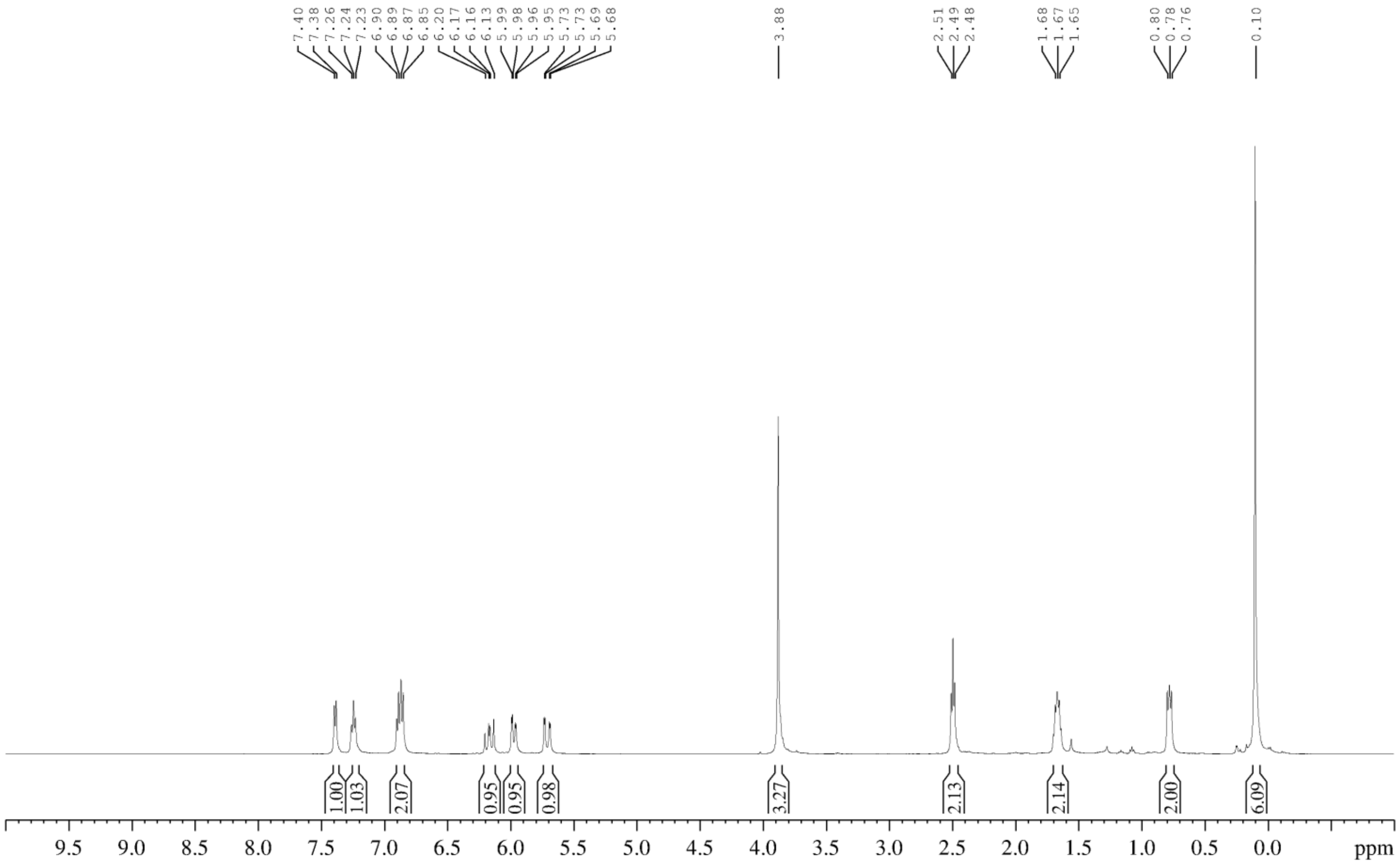

Figure S115. ^{13}C NMR (126 MHz, $CDCl_3$, 298 K) of **3ka**.

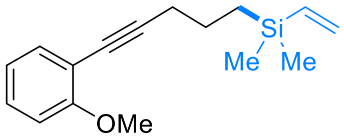

3ka

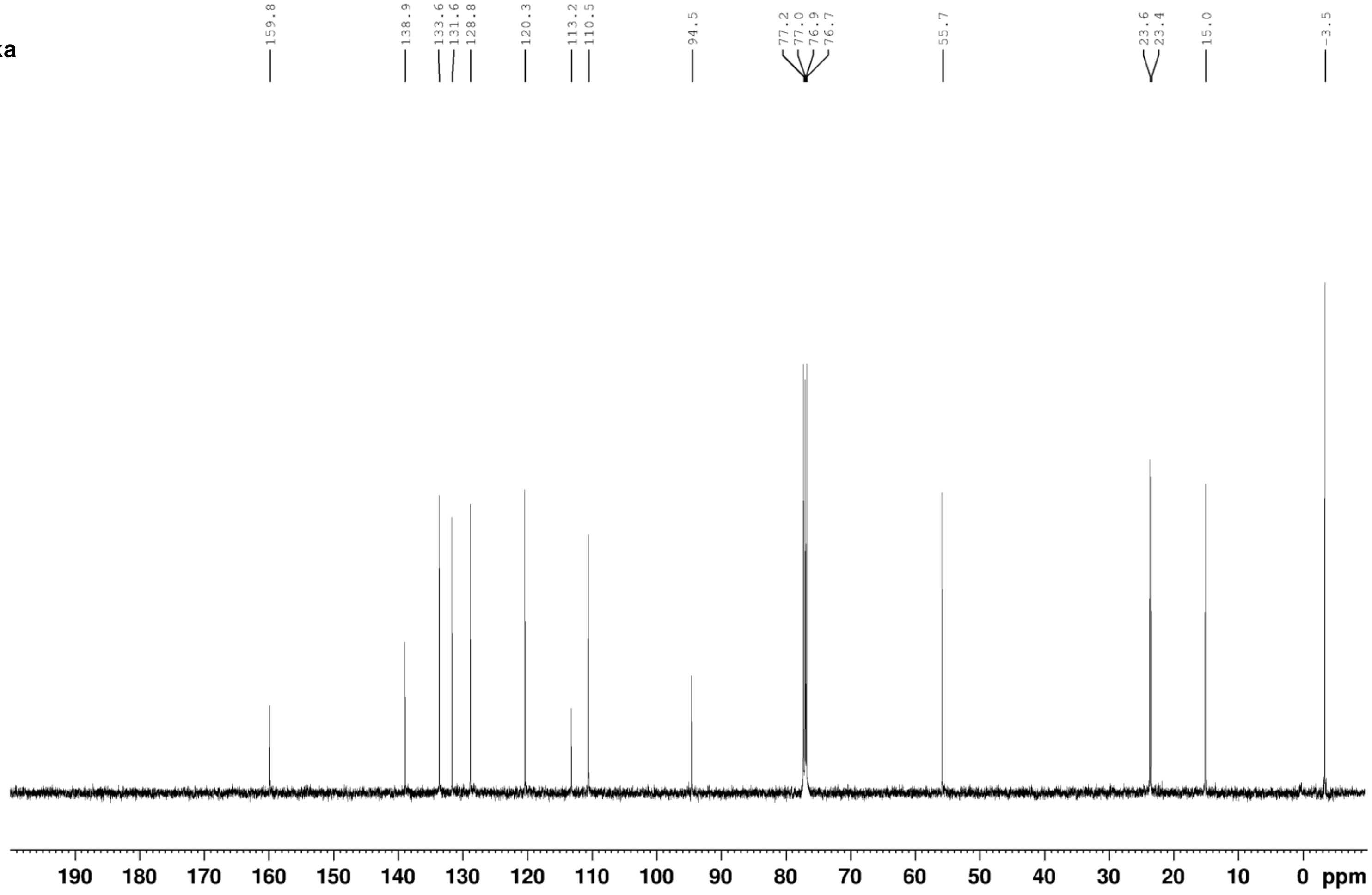

Figure S116. $^{1}H/^{29}Si$ HMQC NMR (500/99 MHz, $CDCl_3$, 298 K, optimized for J = 7 Hz) of **3ka**.

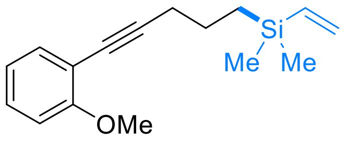

3ka

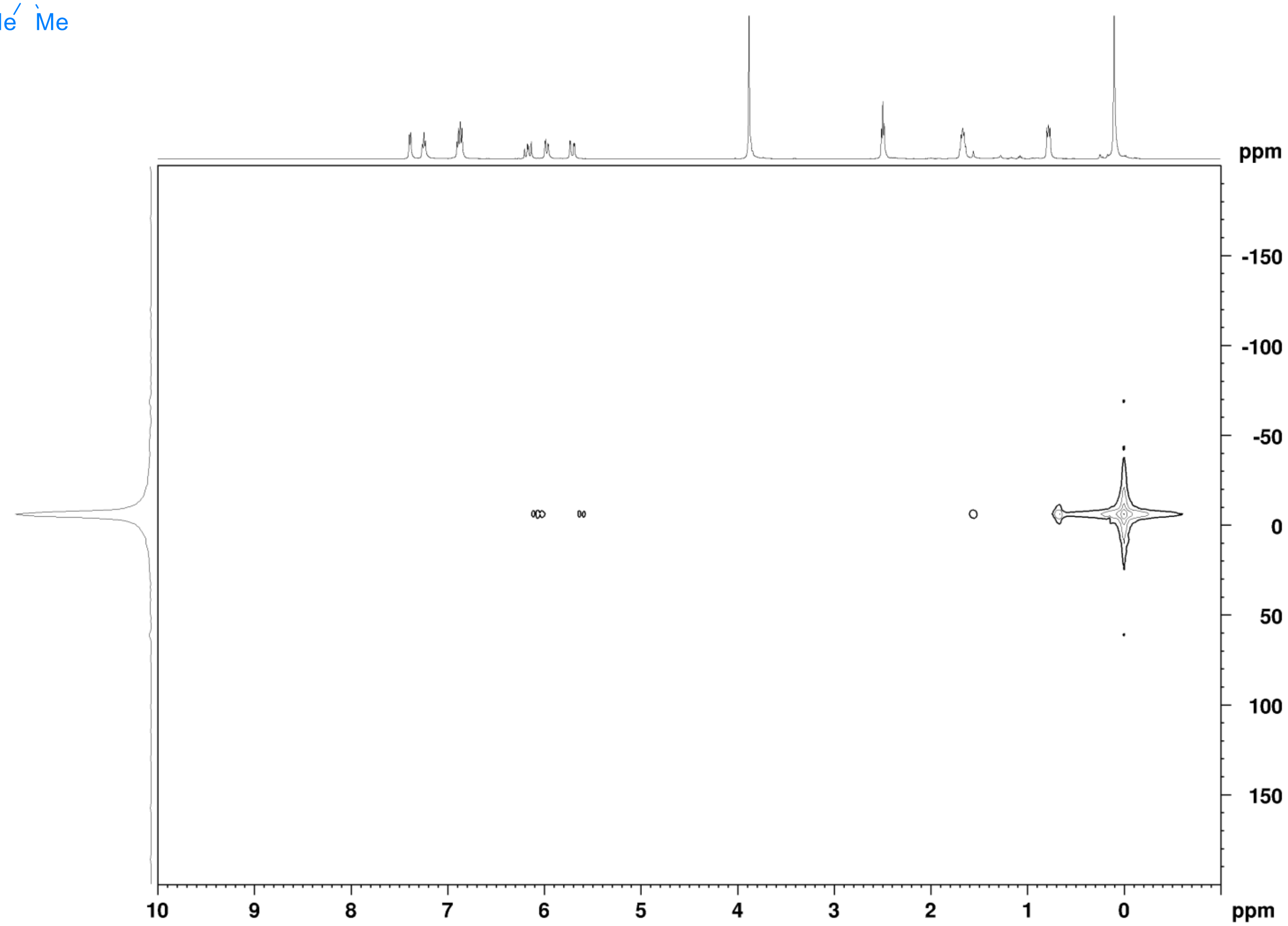

Figure S117. ^{1}H NMR (500 MHz, $CDCl_3$, 298 K) of **3la**.

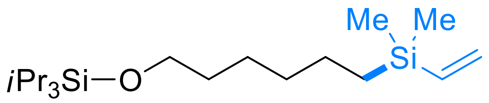

3la

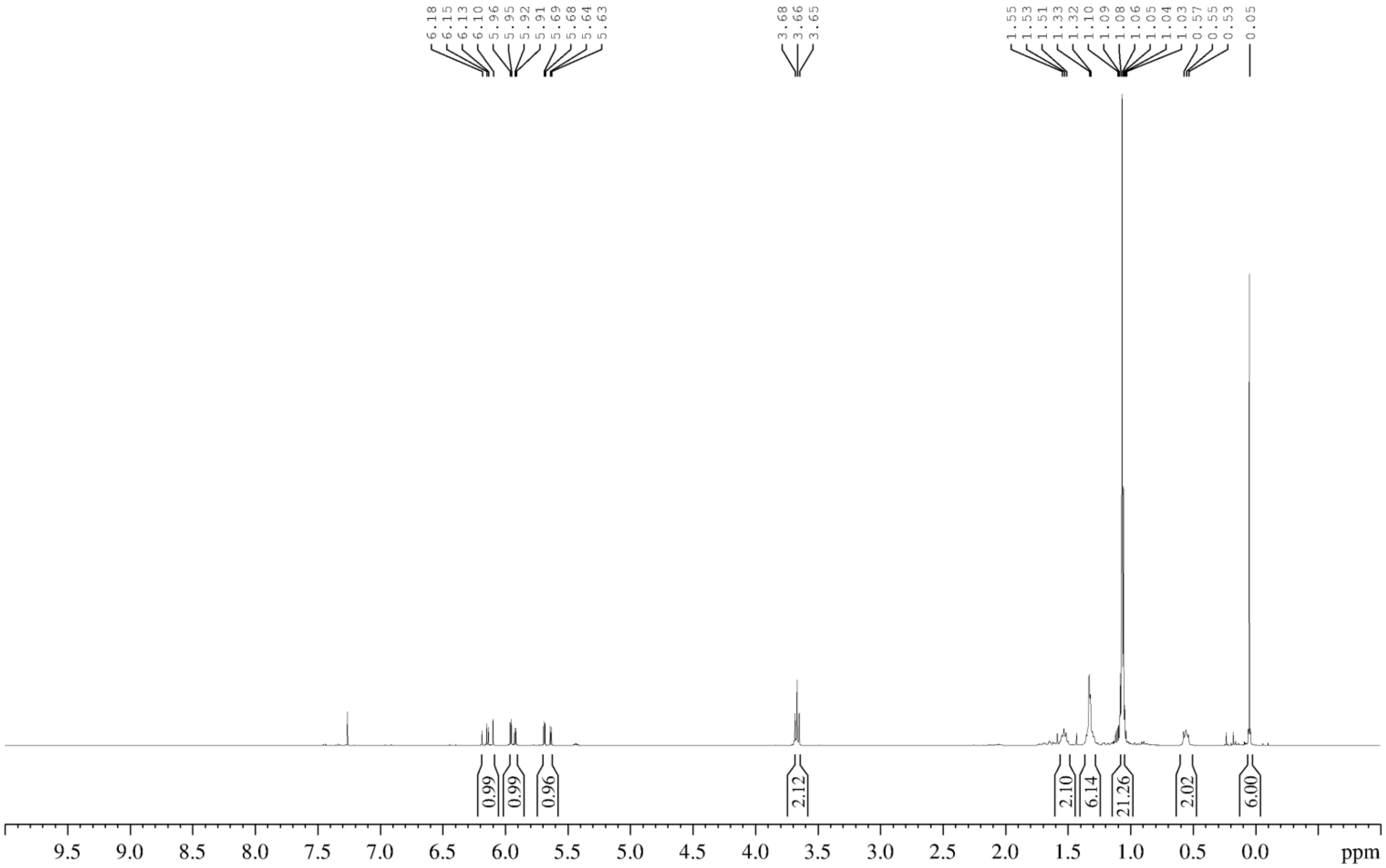

Figure S118. ^{13}C NMR (126 MHz, CDCl$_3$, 298 K) of **3la**.

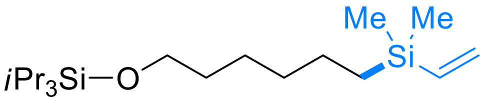

3la

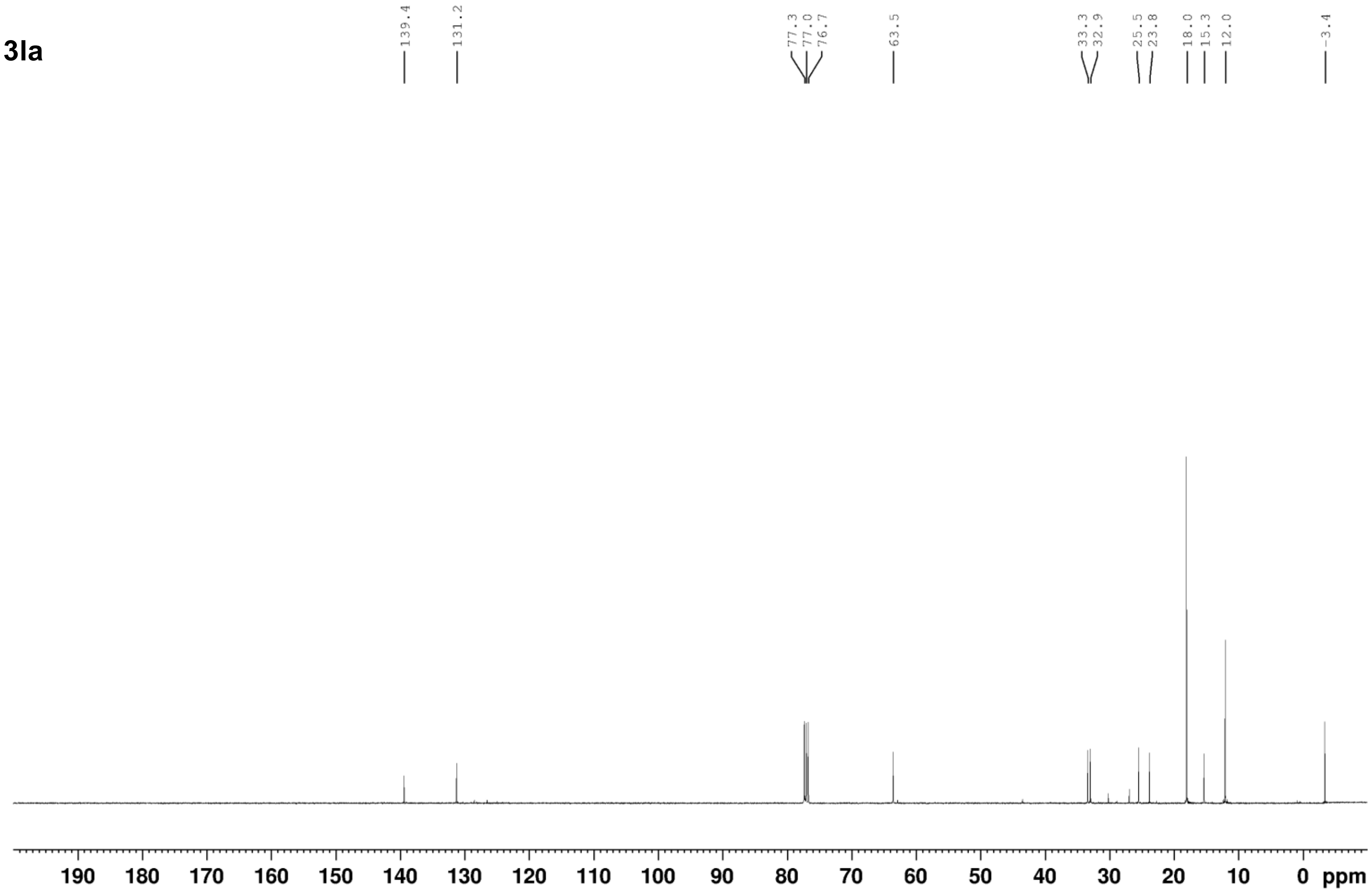

Figure S119. $^{1}H/^{29}Si$ HMQC NMR (500/99 MHz, $CDCl_3$, 298 K, optimized for J = 7 Hz) of **3la**.

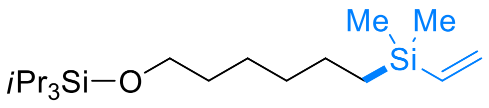

3la

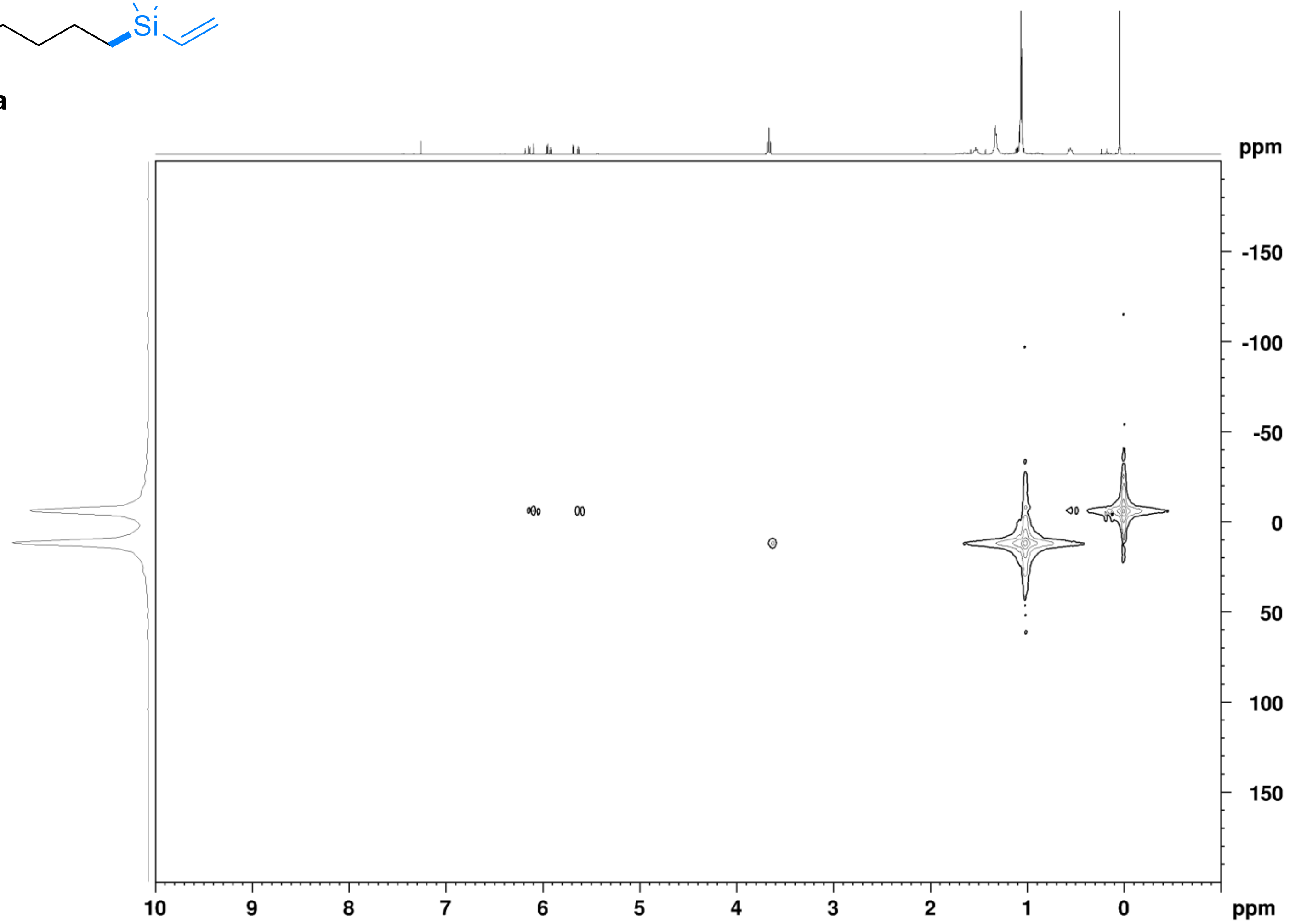

Figure S120. ^{1}H NMR (500 MHz, $CDCl_3$, 298 K) of **3ma**.

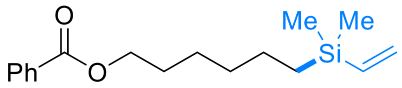

3ma

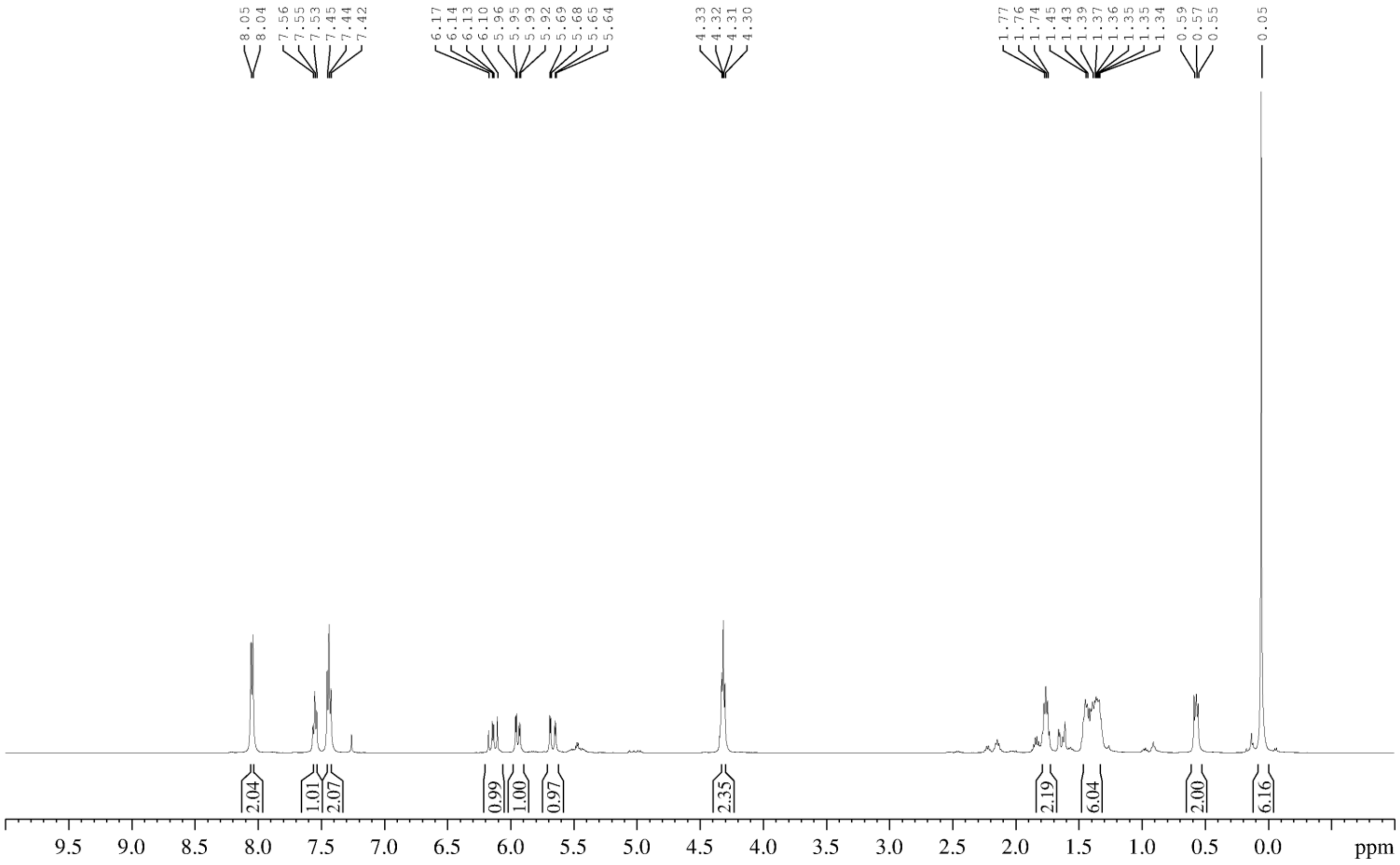

Figure S121. ^{13}C NMR (126 MHz, $CDCl_3$, 298 K) of **3ma**.

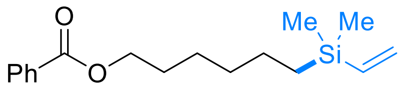

3ma

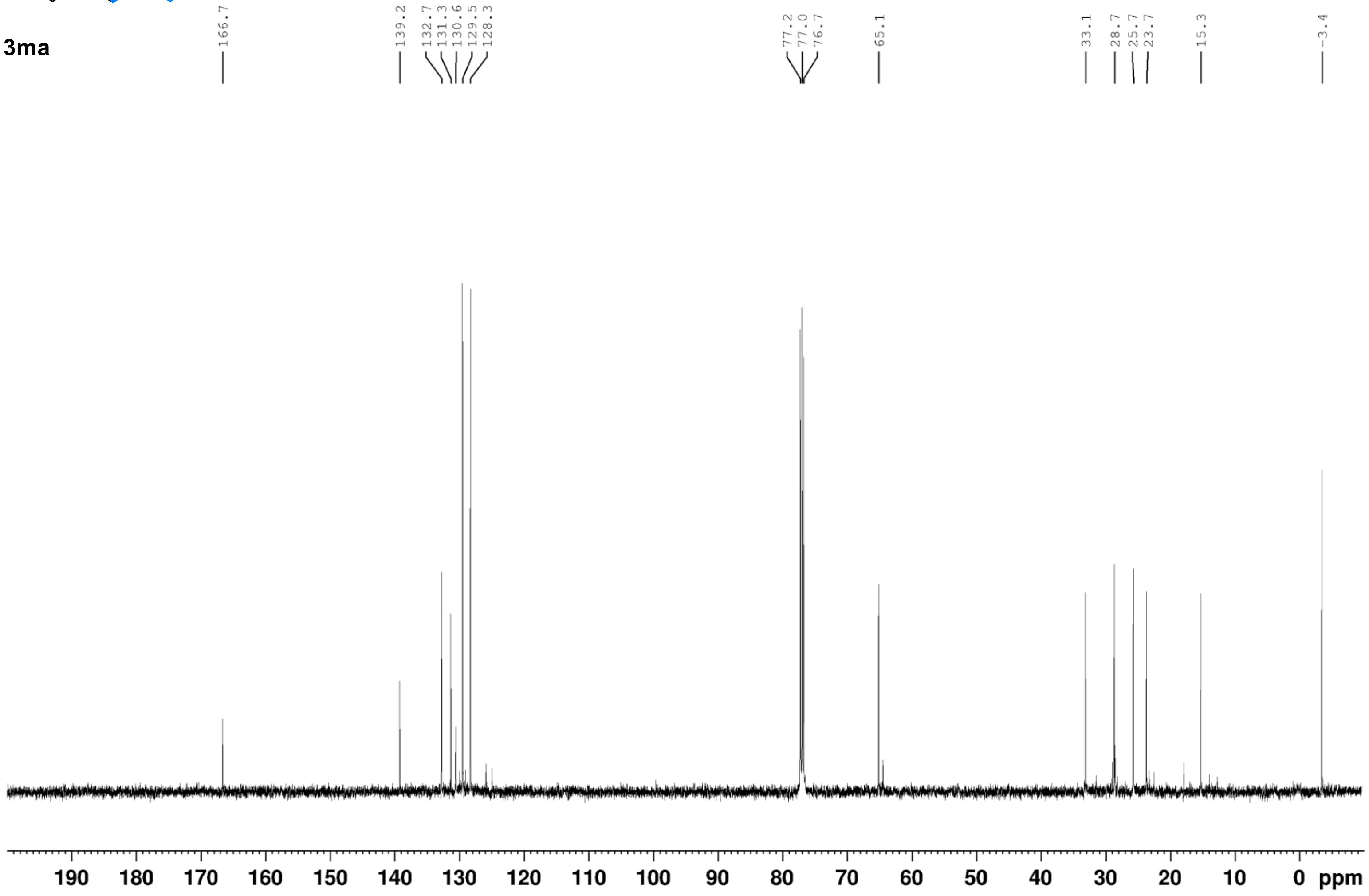

Figure S122. $^{1}H/^{29}Si$ HMQC NMR (500/99 MHz, $CDCl_3$, 298 K, optimized for J = 7 Hz) of **3ma**.

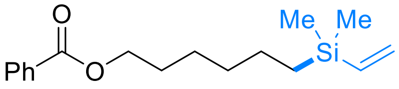

3ma

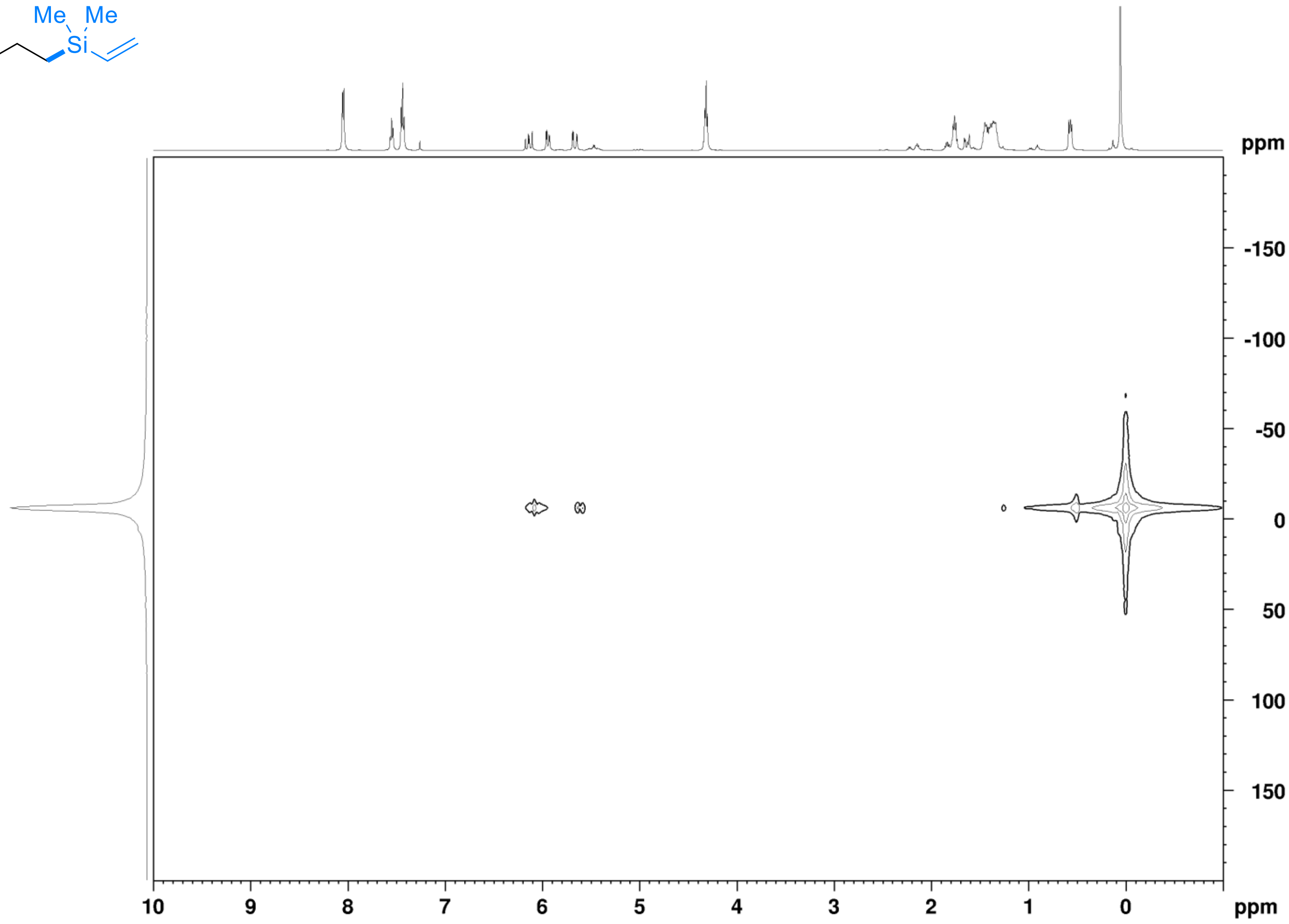

Figure S123. ^{1}H NMR (500 MHz, $CDCl_3$, 298 K) of **3na**.

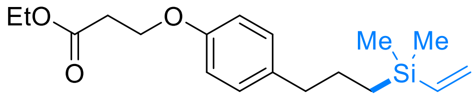

3na

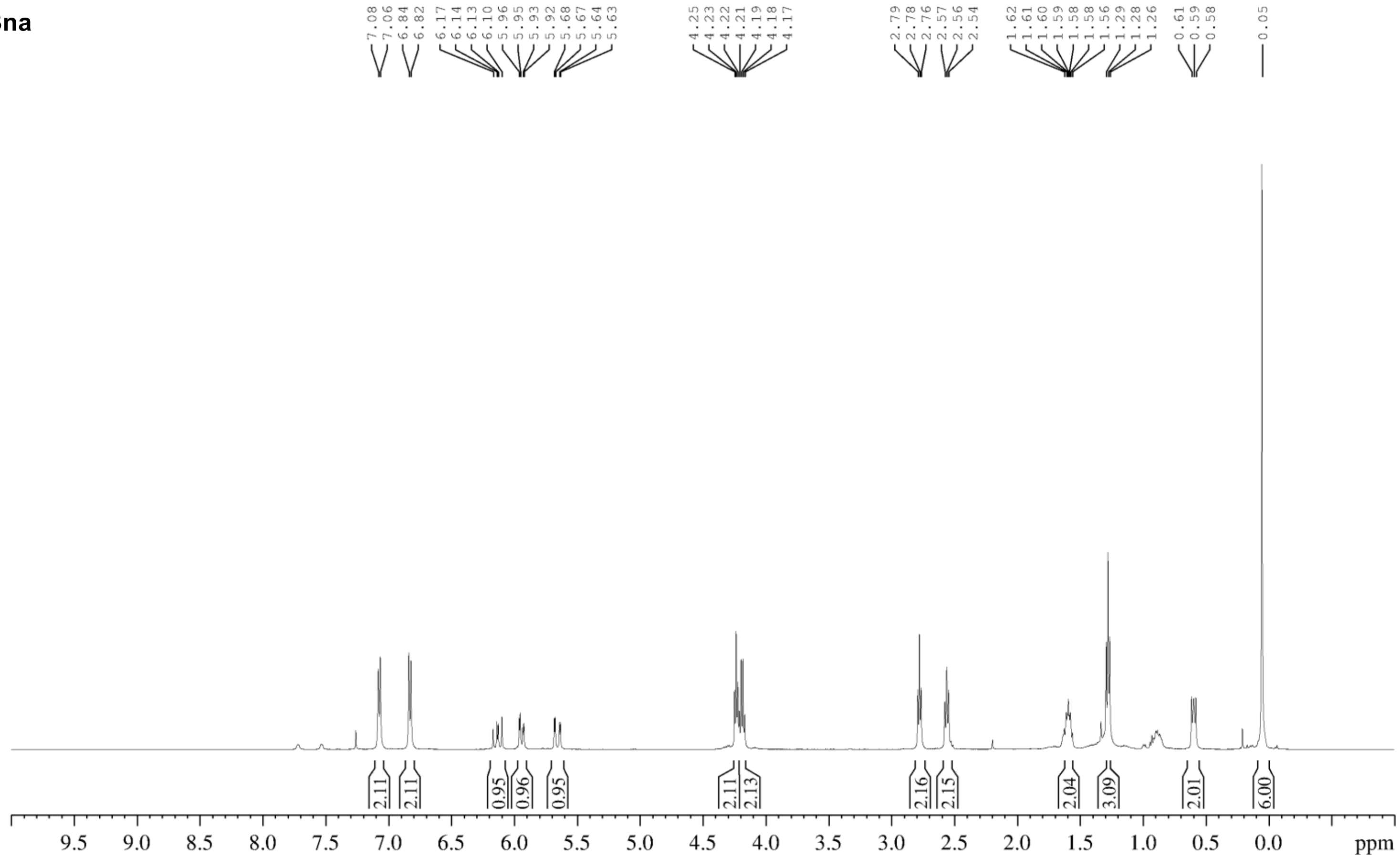

Figure S124. ^{13}C NMR (126 MHz, $CDCl_3$, 298 K) of **3na**.

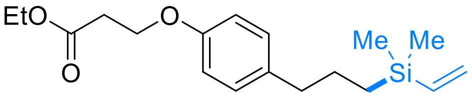

3na

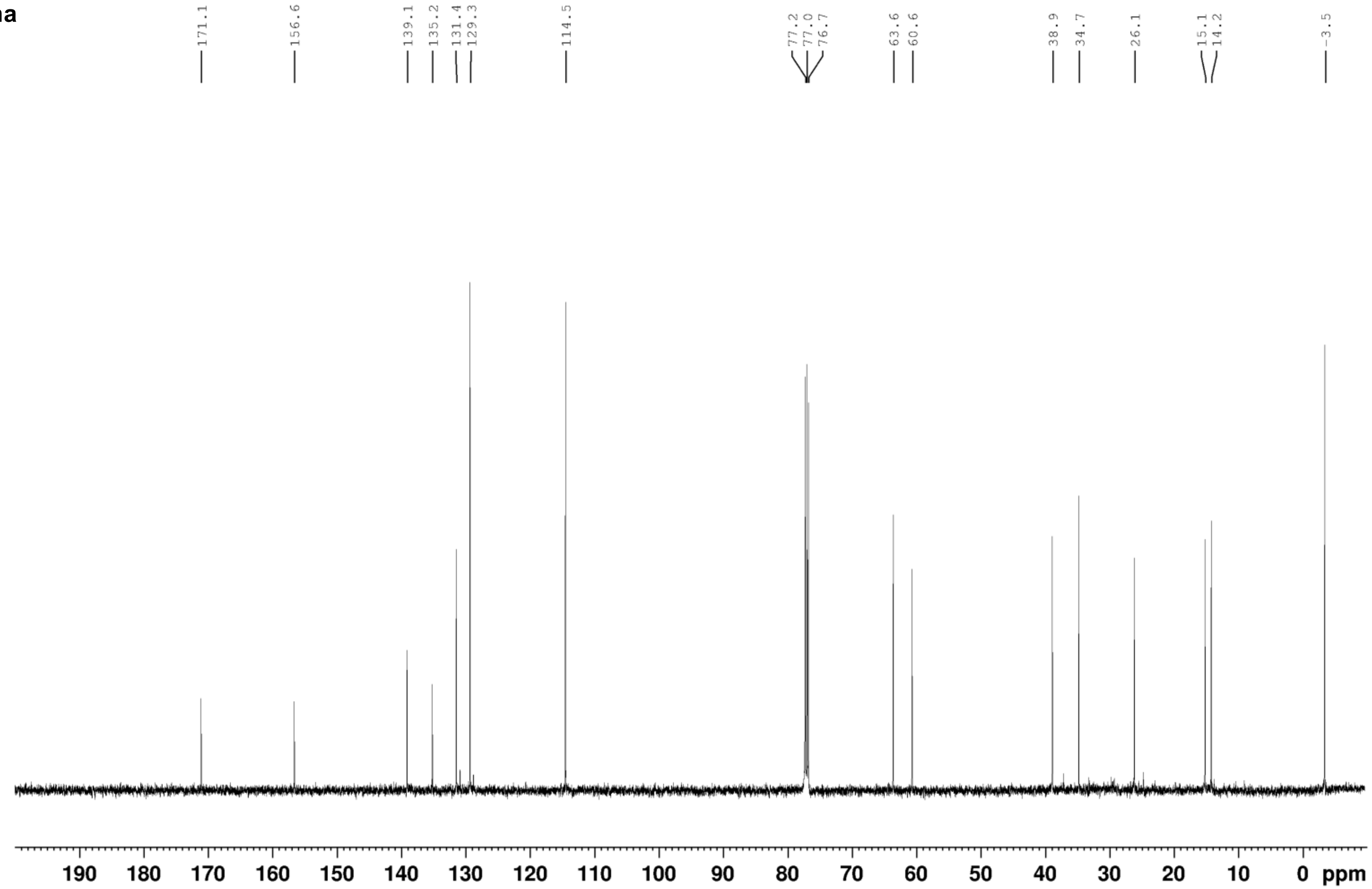

Figure S125. $^{1}H/^{29}Si$ HMQC NMR (500/99 MHz, $CDCl_3$, 298 K, optimized for J = 7 Hz) of **3na**.

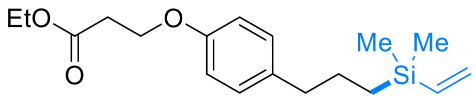

3na

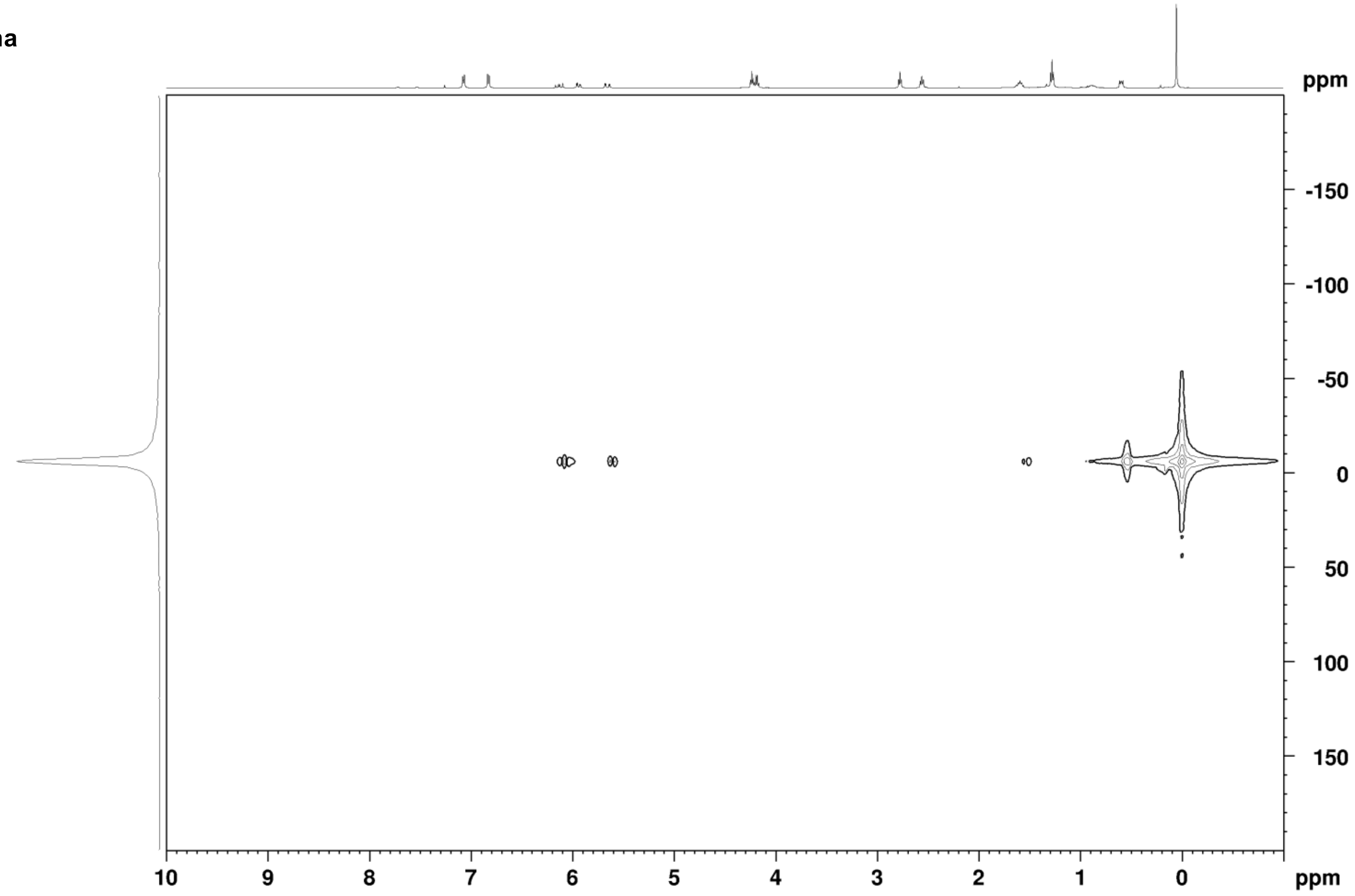

Figure S126. ^{1}H NMR (500 MHz, $CDCl_3$, 298 K) of **3oa**.

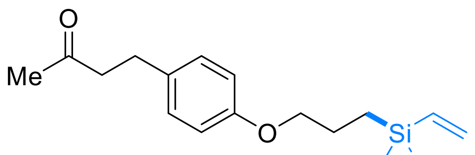

3oa

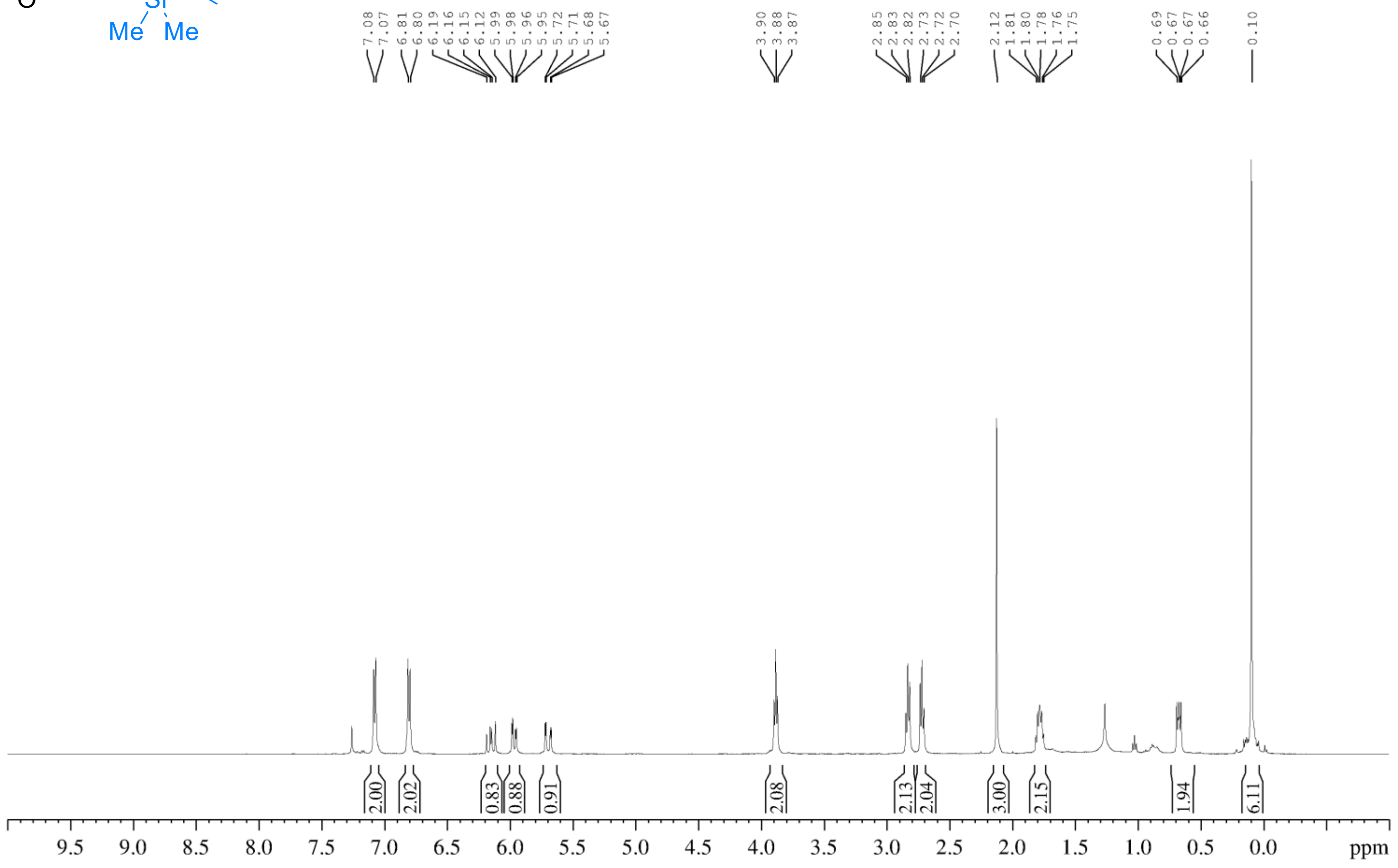

Figure S127. ^{13}C NMR (126 MHz, $CDCl_3$, 298 K) of **3oa**.

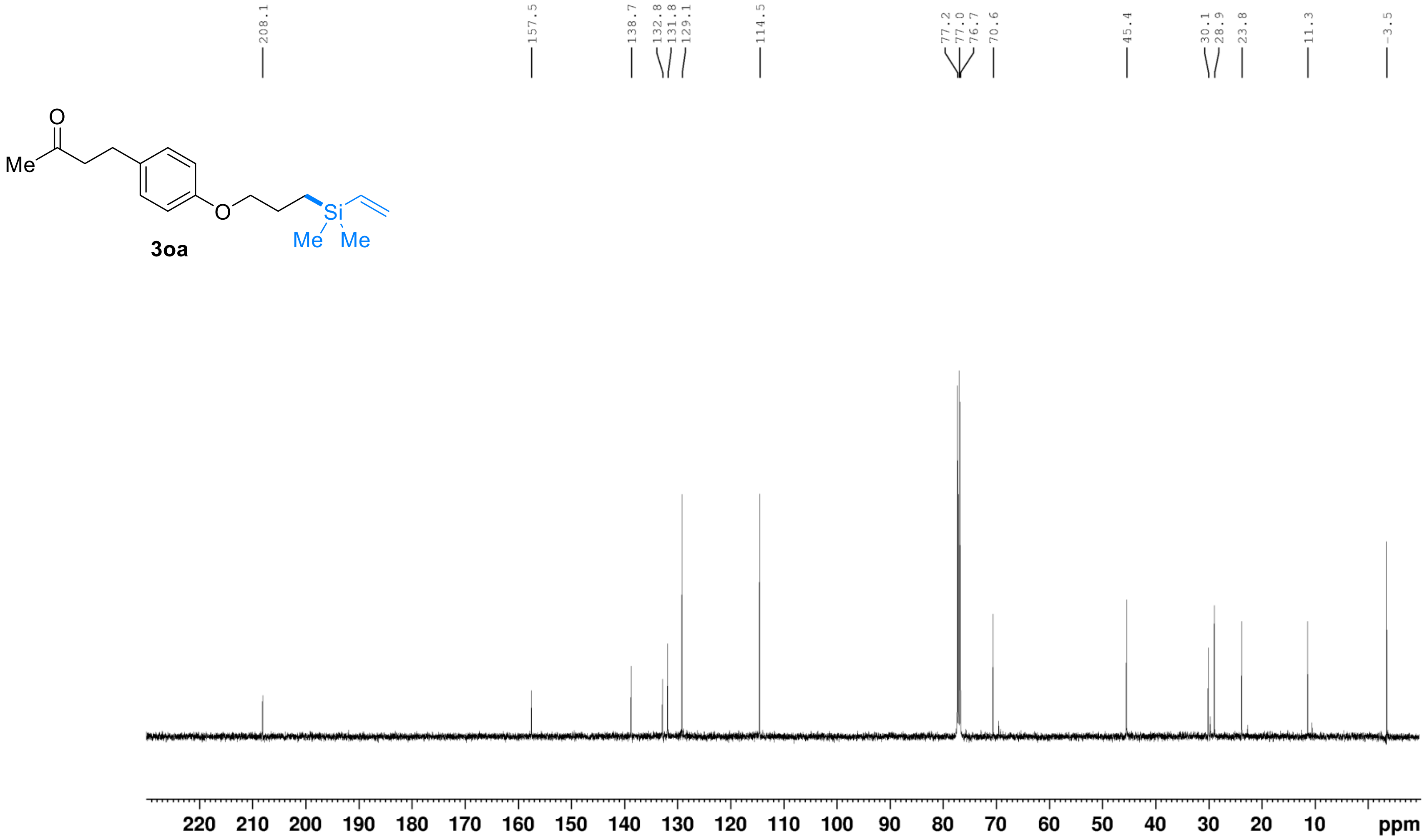

Figure S128. $^{1}H/^{29}Si$ HMQC NMR (500/99 MHz, $CDCl_3$, 298 K, optimized for J = 7 Hz) of **3oa**.

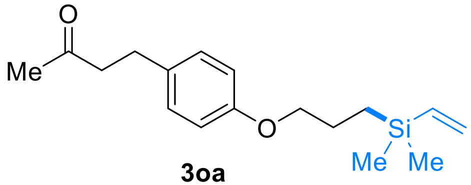

3oa

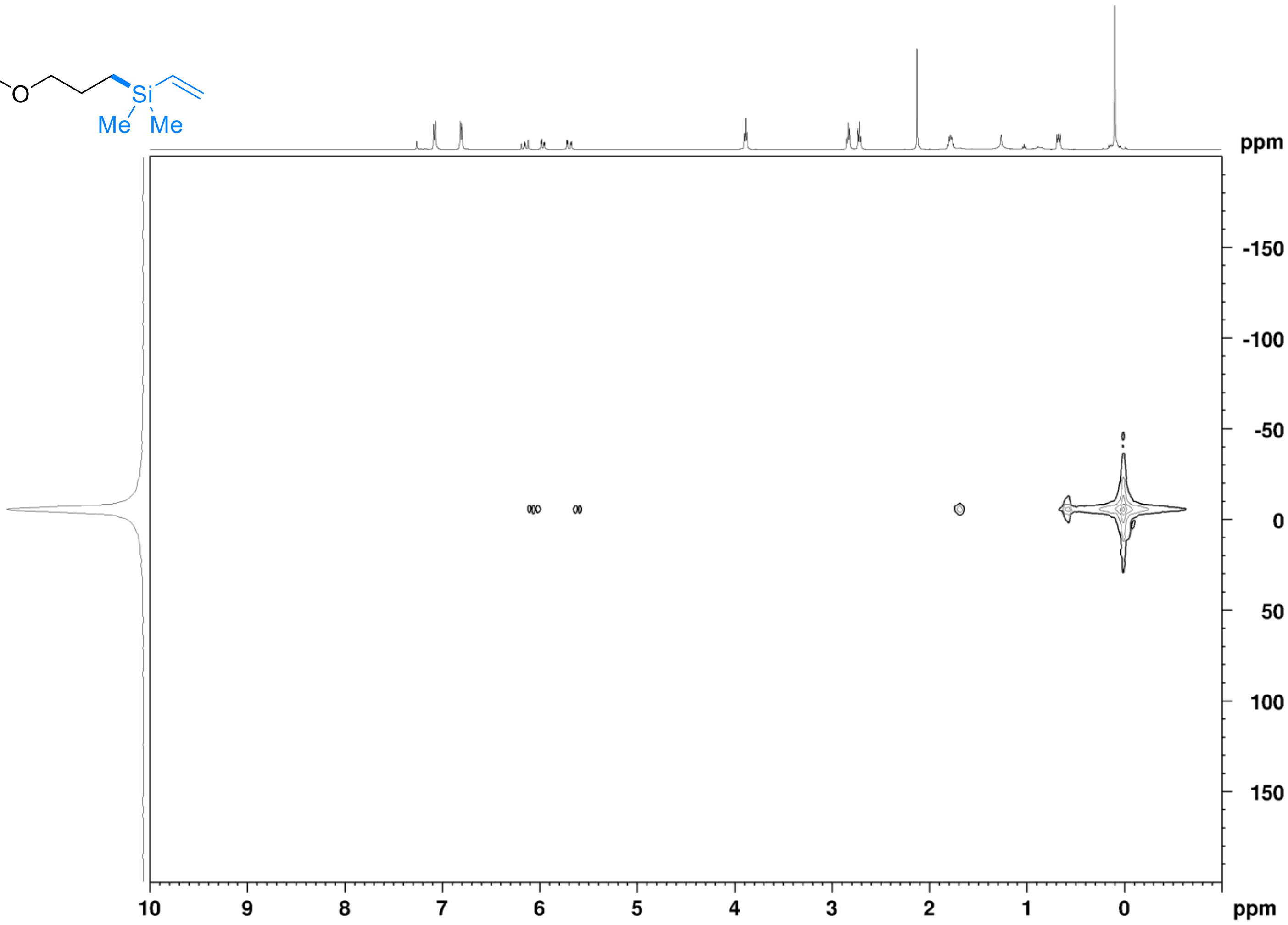

Figure S129. ^{1}H NMR (500 MHz, $CDCl_3$, 298 K) of **3pa**.

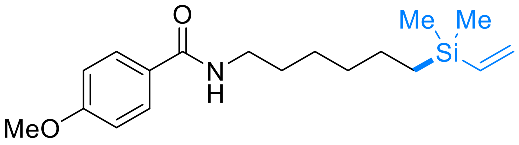

3pa

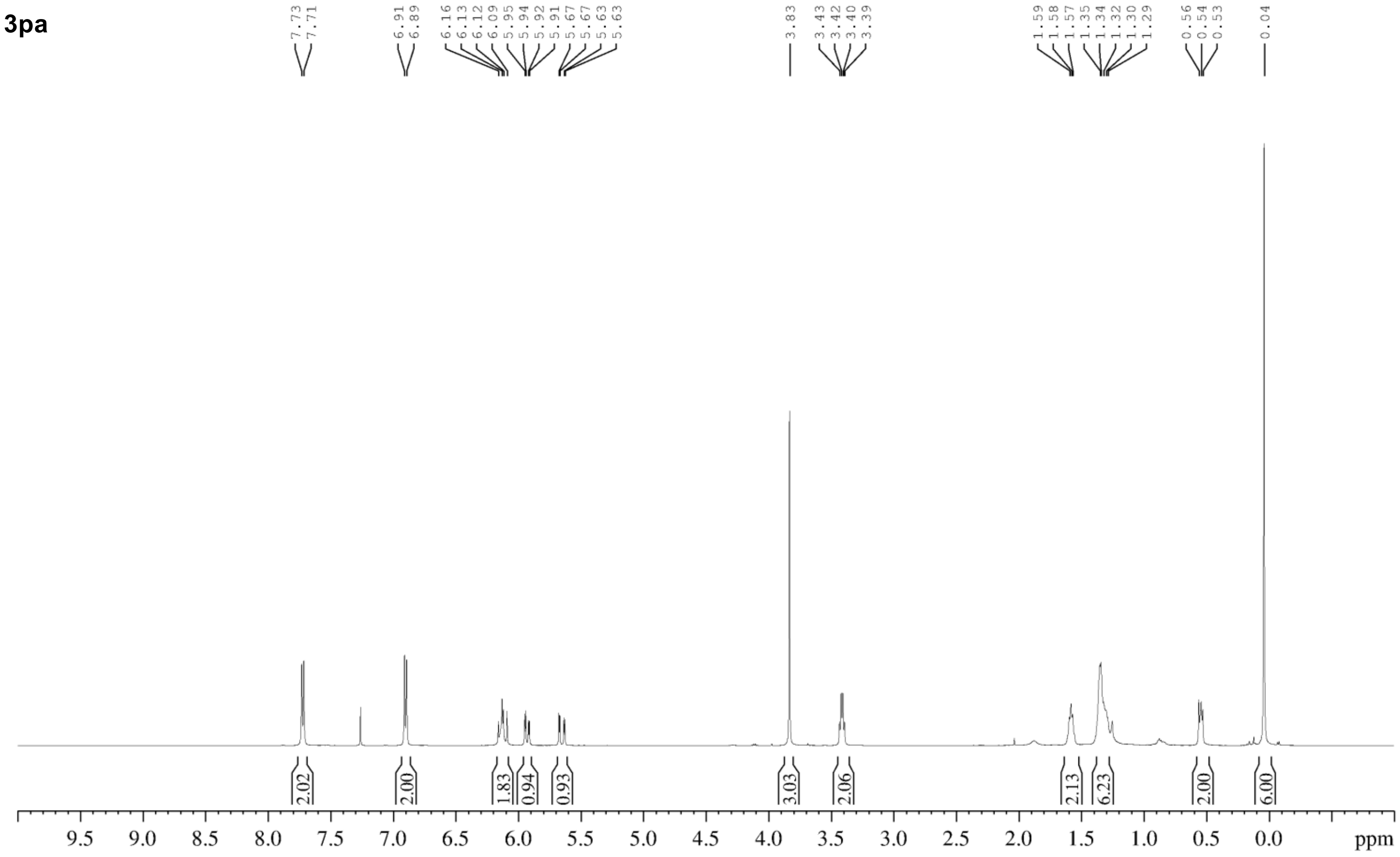

Figure S130. ^{13}C NMR (126 MHz, $CDCl_3$, 298 K) of **3pa**.

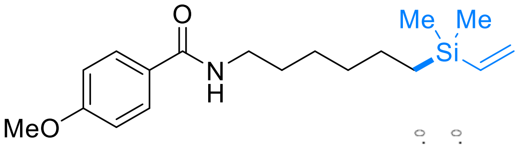

3pa

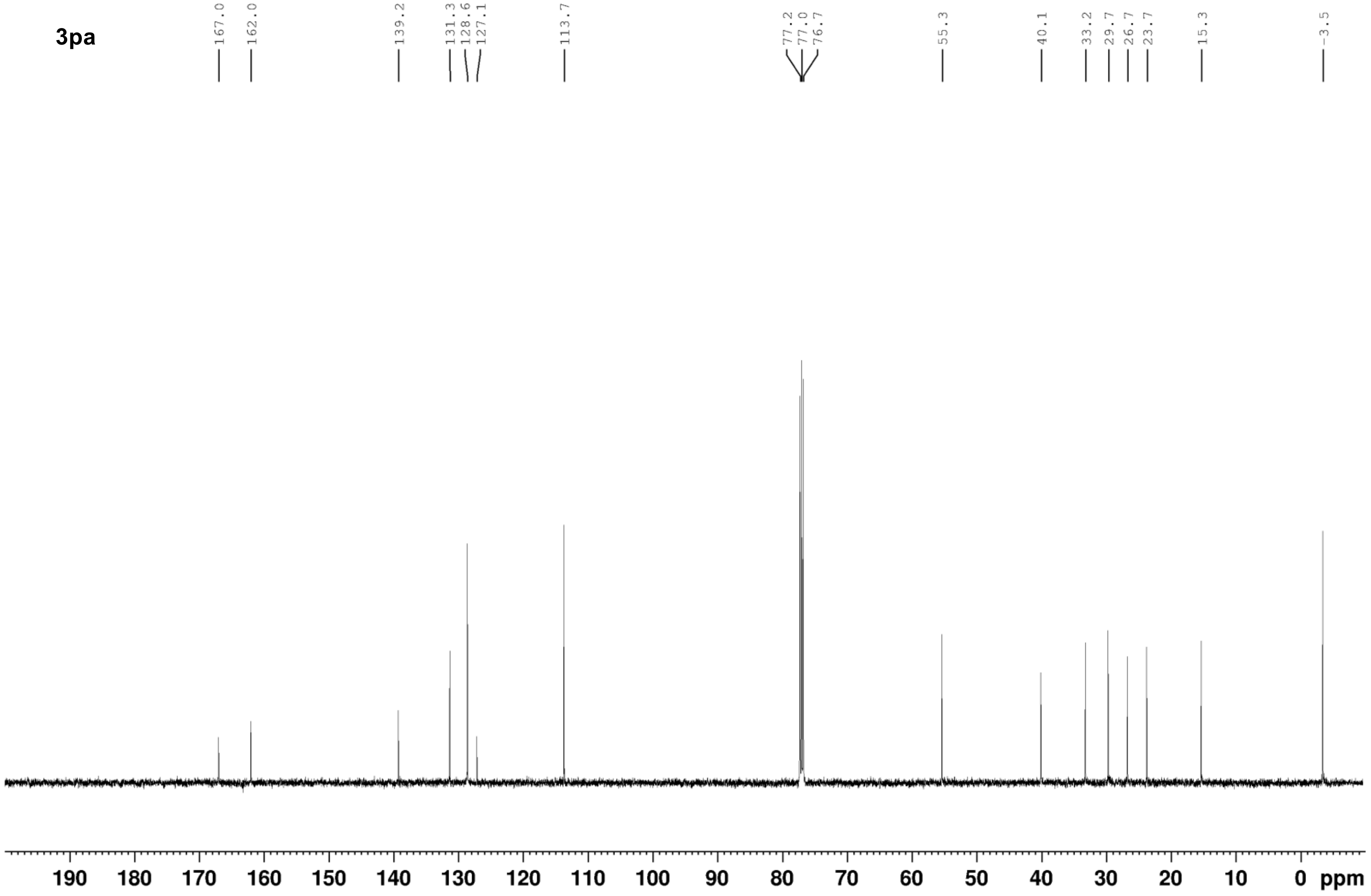

Figure S131. $^{1}H/^{29}Si$ HMQC NMR (500/99 MHz, $CDCl_3$, 298 K, optimized for J = 7 Hz) of **3pa**.

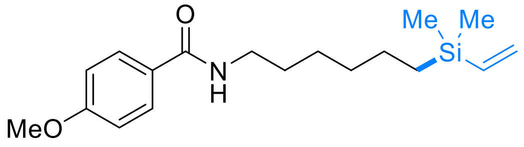

3pa

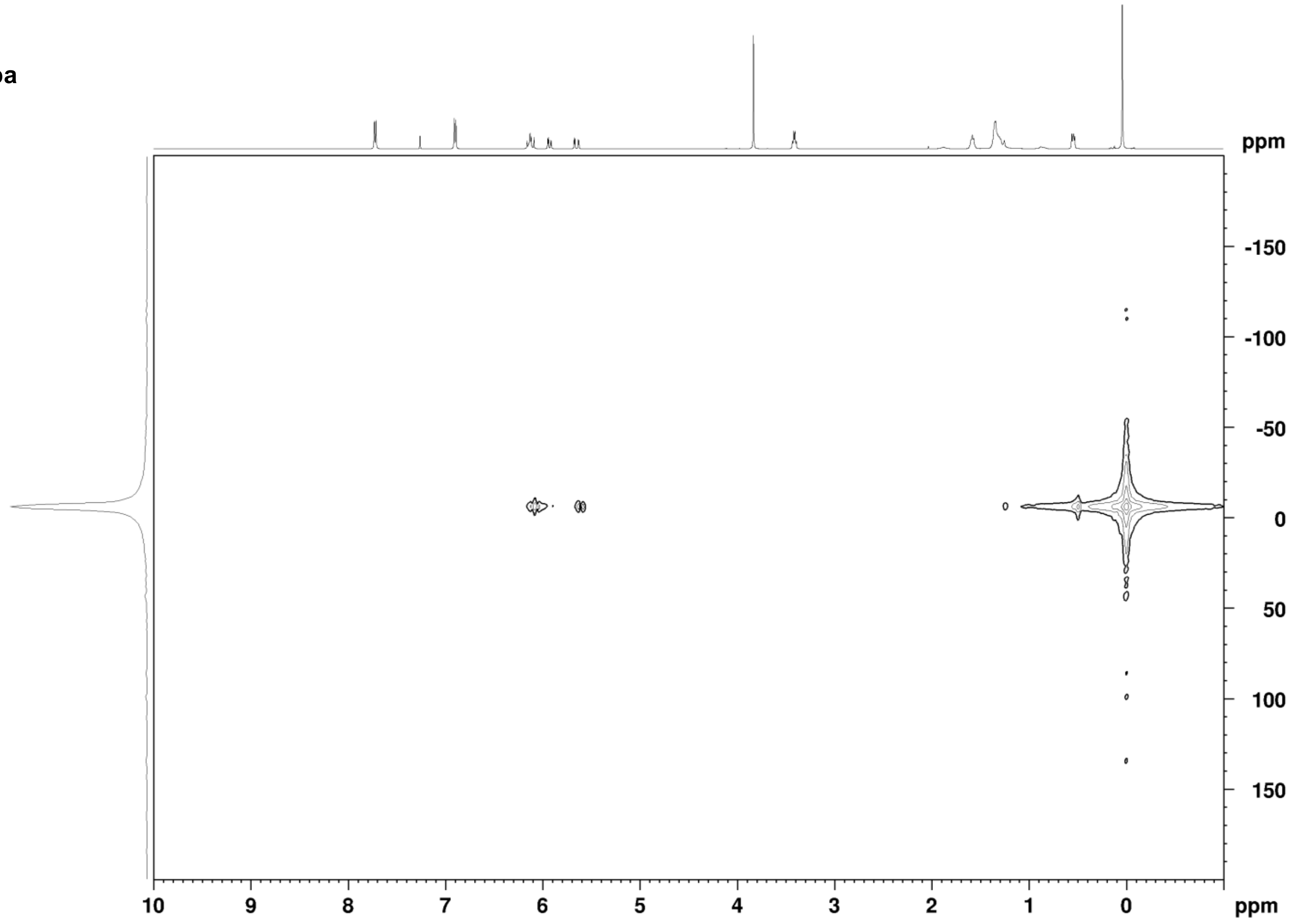

Figure S132. ^{1}H NMR (500 MHz, $CDCl_3$, 298 K) of **3qa**.

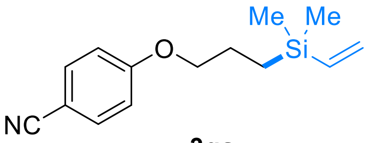

3qa

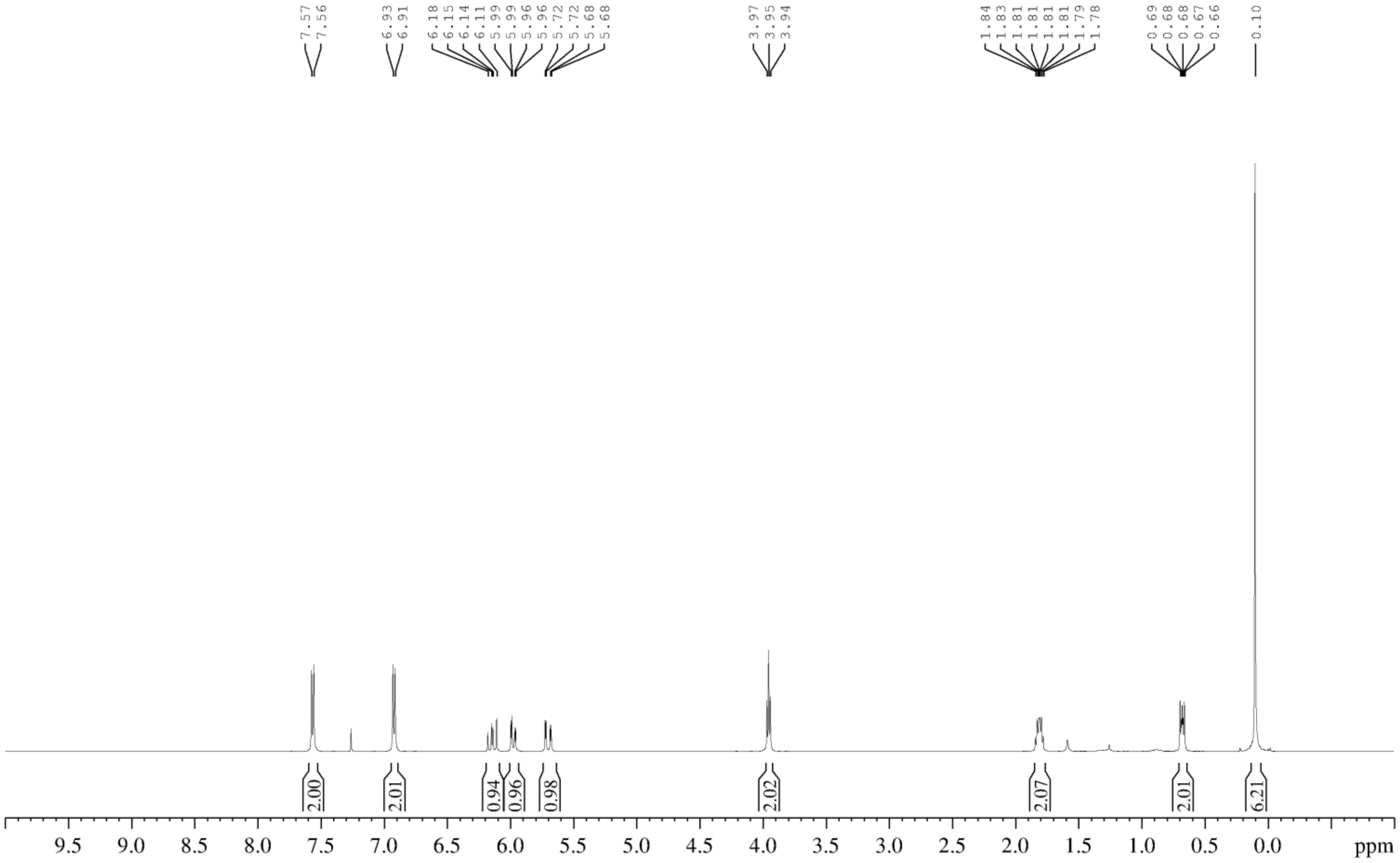

Figure S133. ^{13}C NMR (126 MHz, $CDCl_3$, 298 K) of **3qa**.

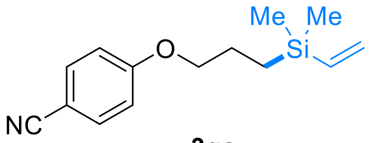

3qa

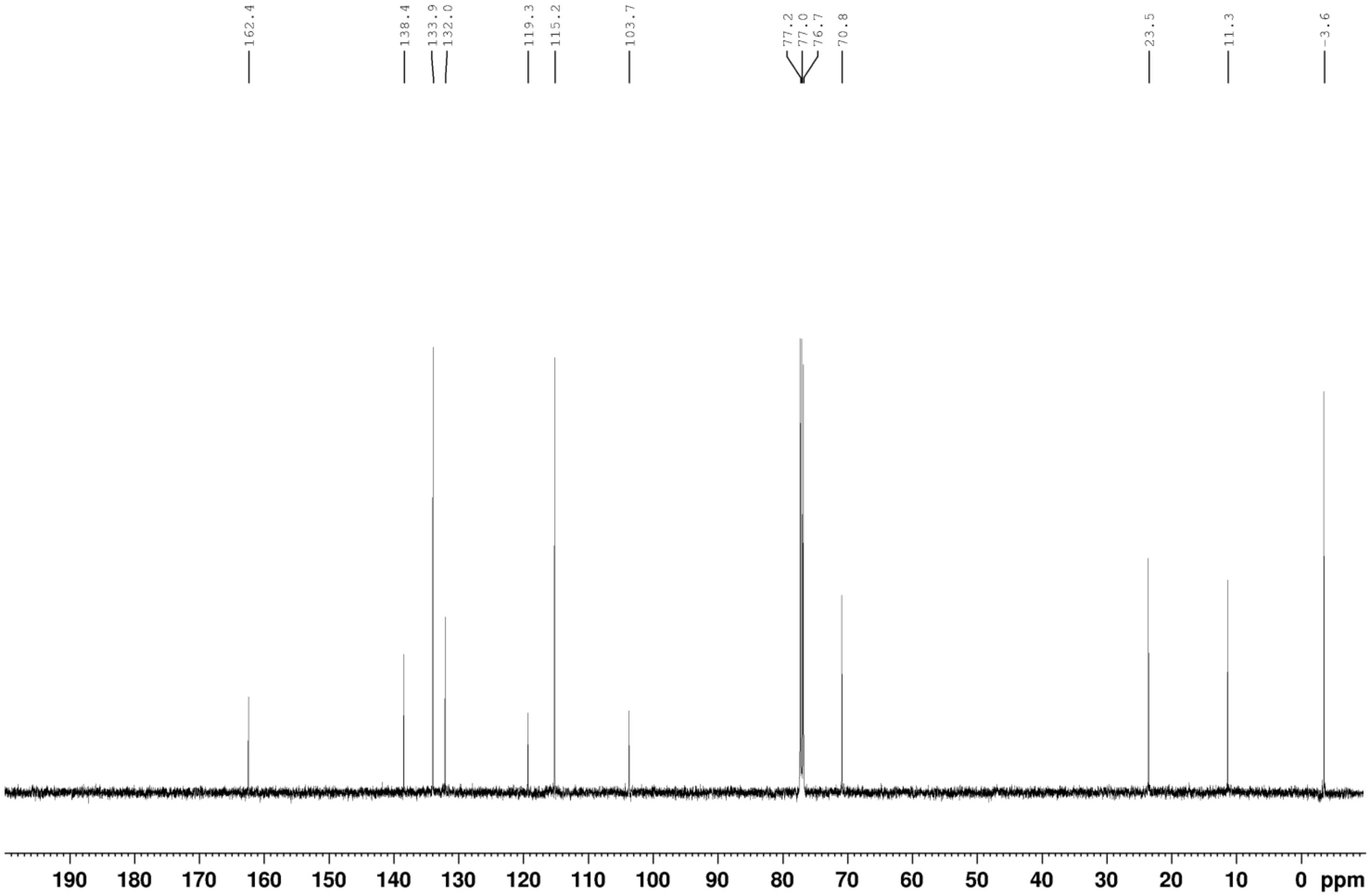

Figure S134. $^{1}H/^{29}Si$ HMQC NMR (500/99 MHz, $CDCl_3$, 298 K, optimized for J = 7 Hz) of **3qa**.

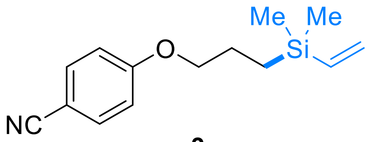

3qa

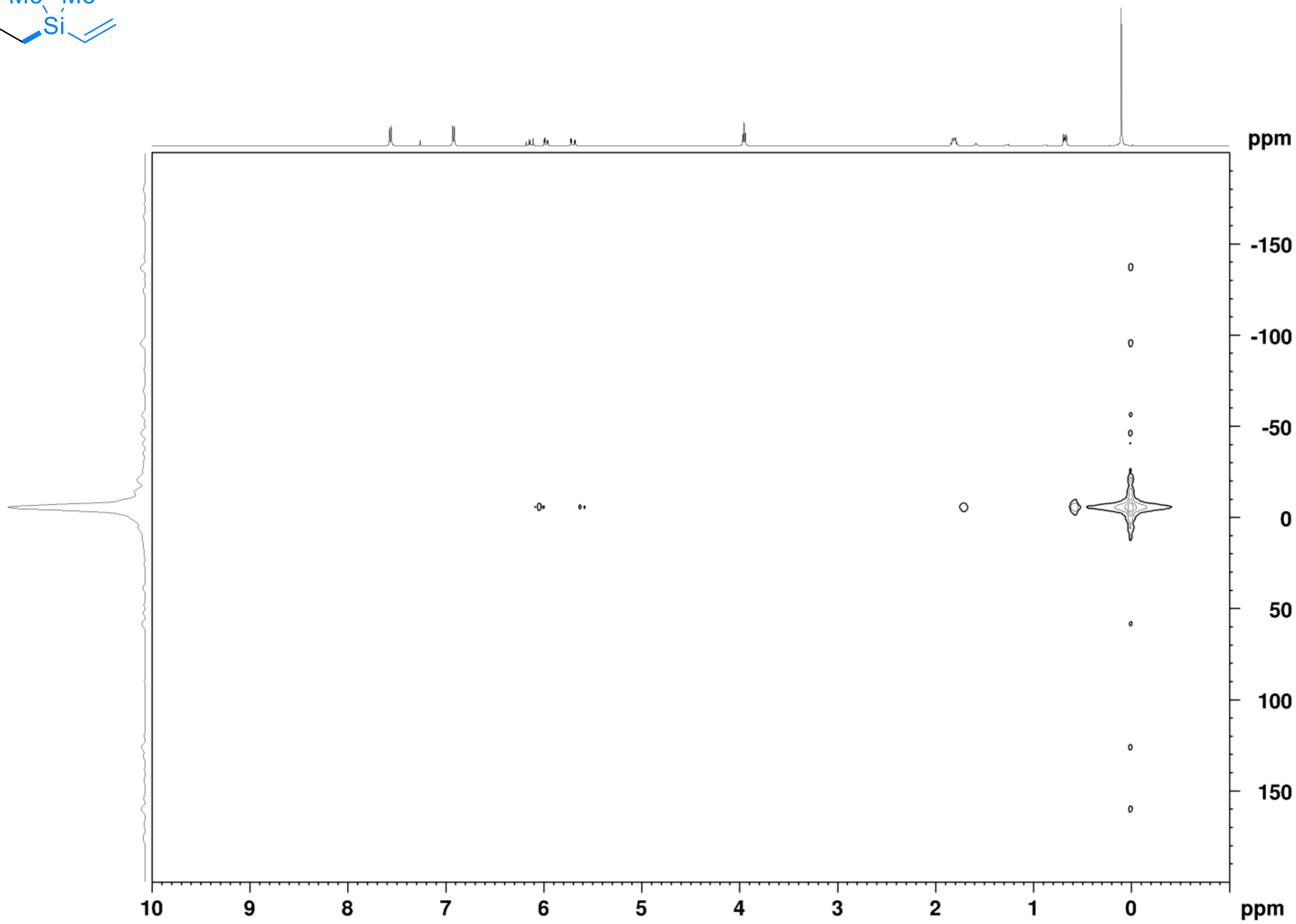

Figure S135. ^{1}H NMR (500 MHz, $CDCl_3$, 298 K) of **3ra**.

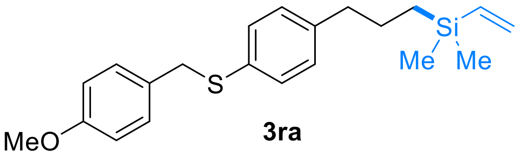

3ra

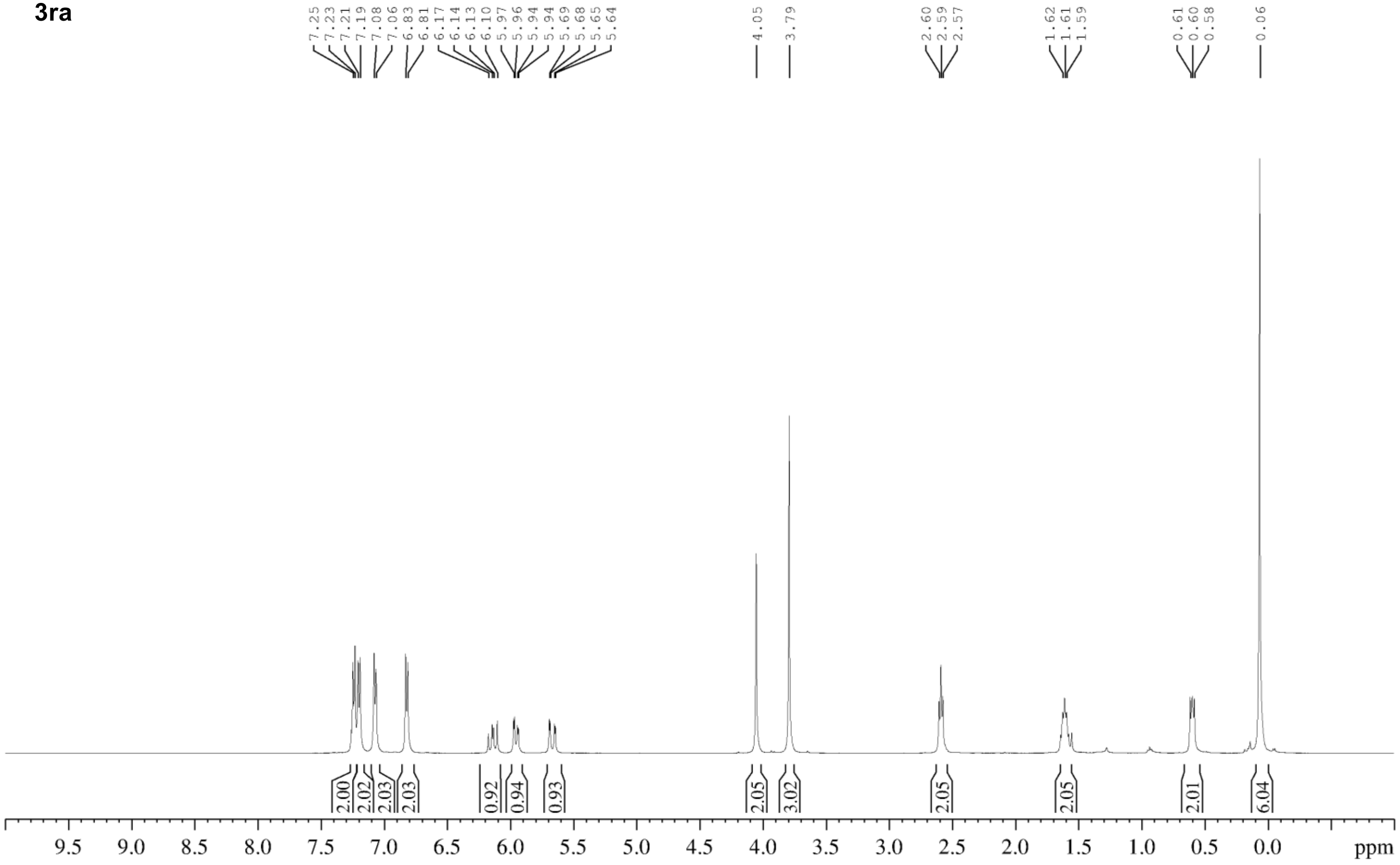

Figure S136. ^{13}C NMR (126 MHz, $CDCl_3$, 298 K) of **3ra**.

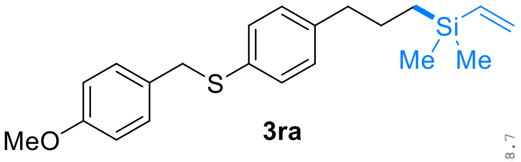

3ra

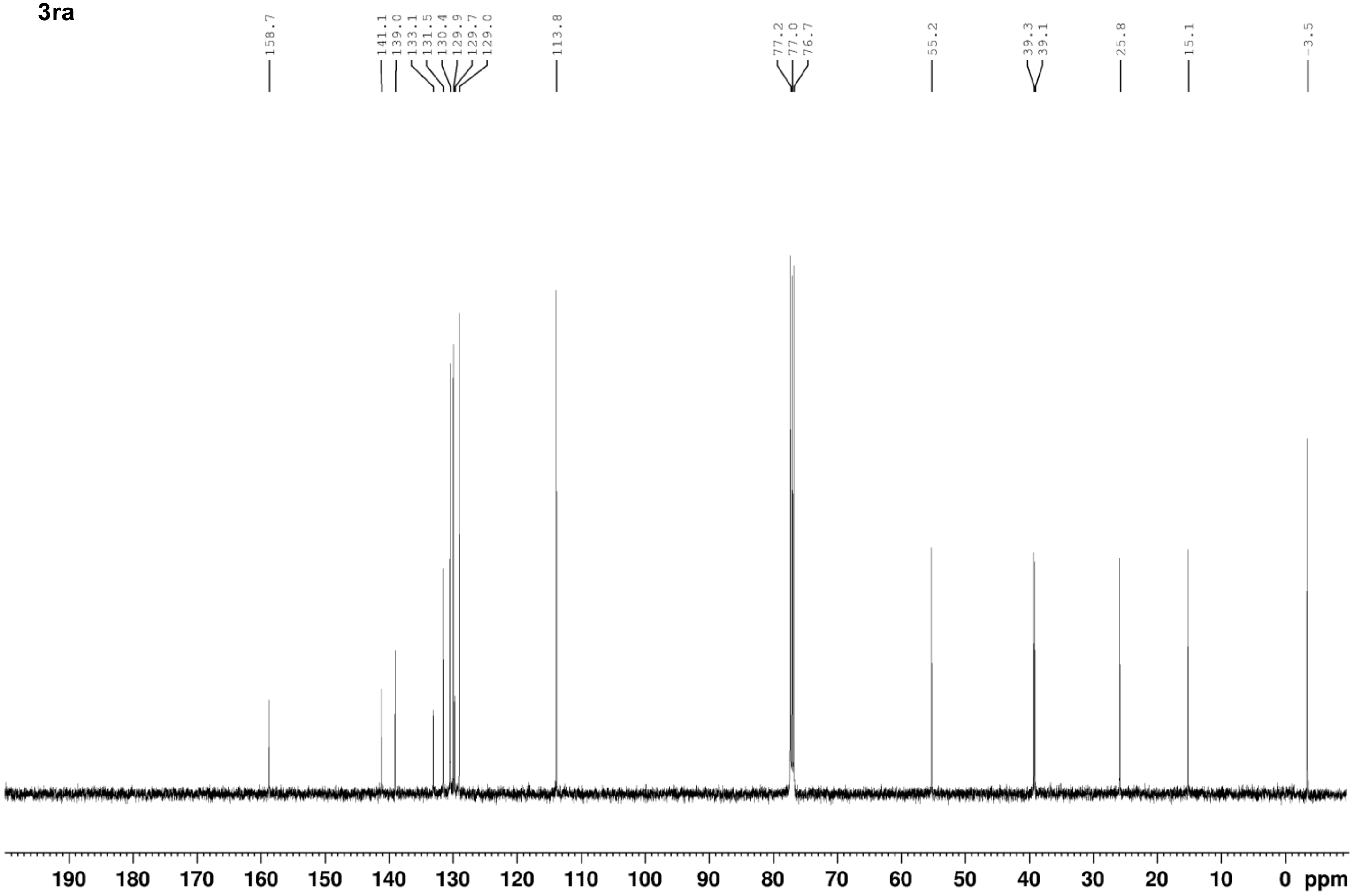

Figure S137. $^{1}H/^{29}Si$ HMQC NMR (500/99 MHz, $CDCl_3$, 298 K, optimized for J = 7 Hz) of **3ra**.

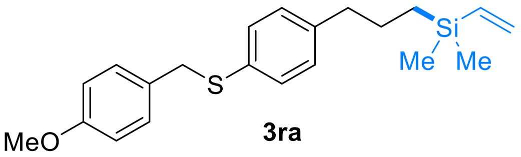

3ra

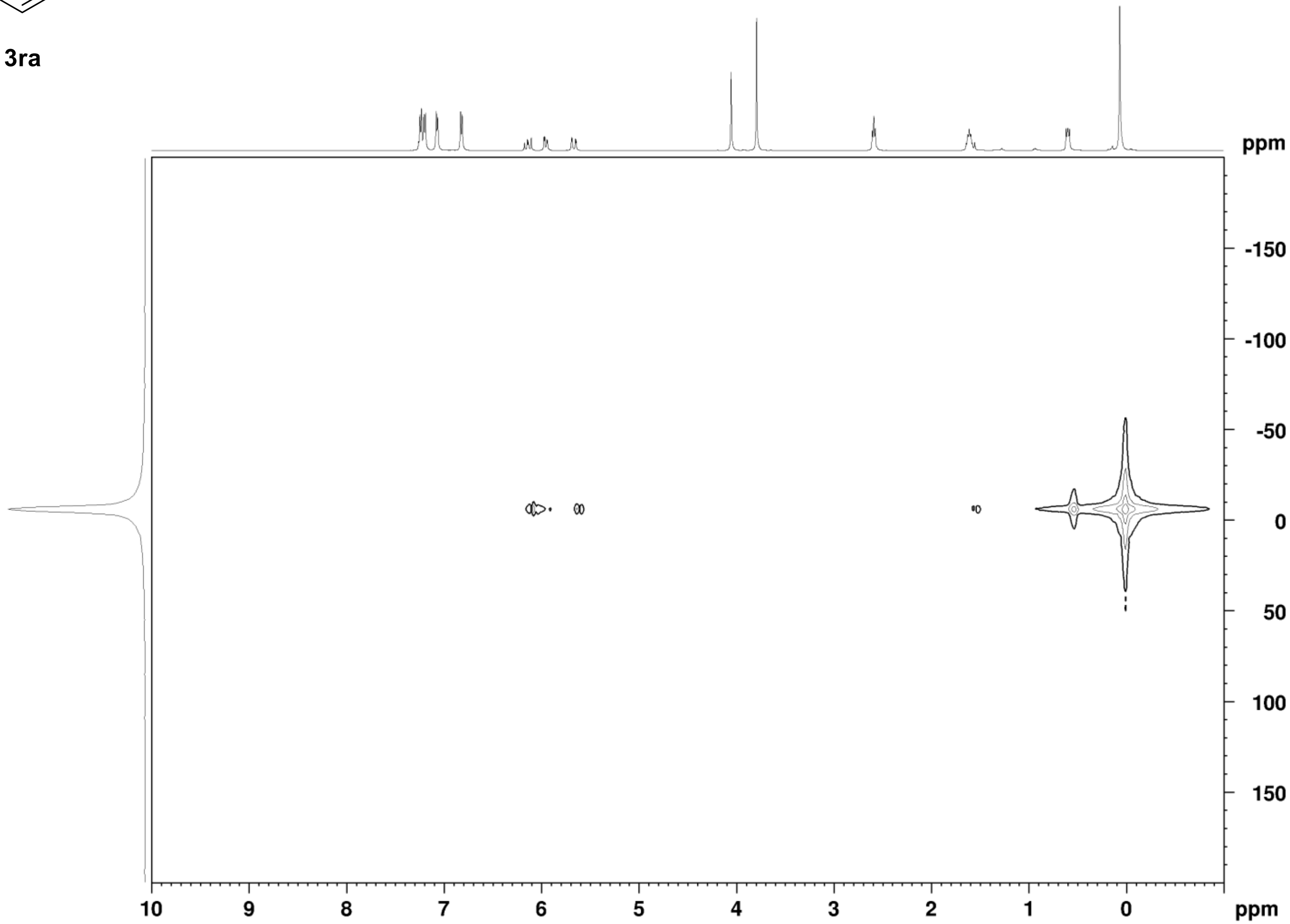

Figure S138. ^{1}H NMR (500 MHz, $CDCl_3$, 298 K) of **3sa**.

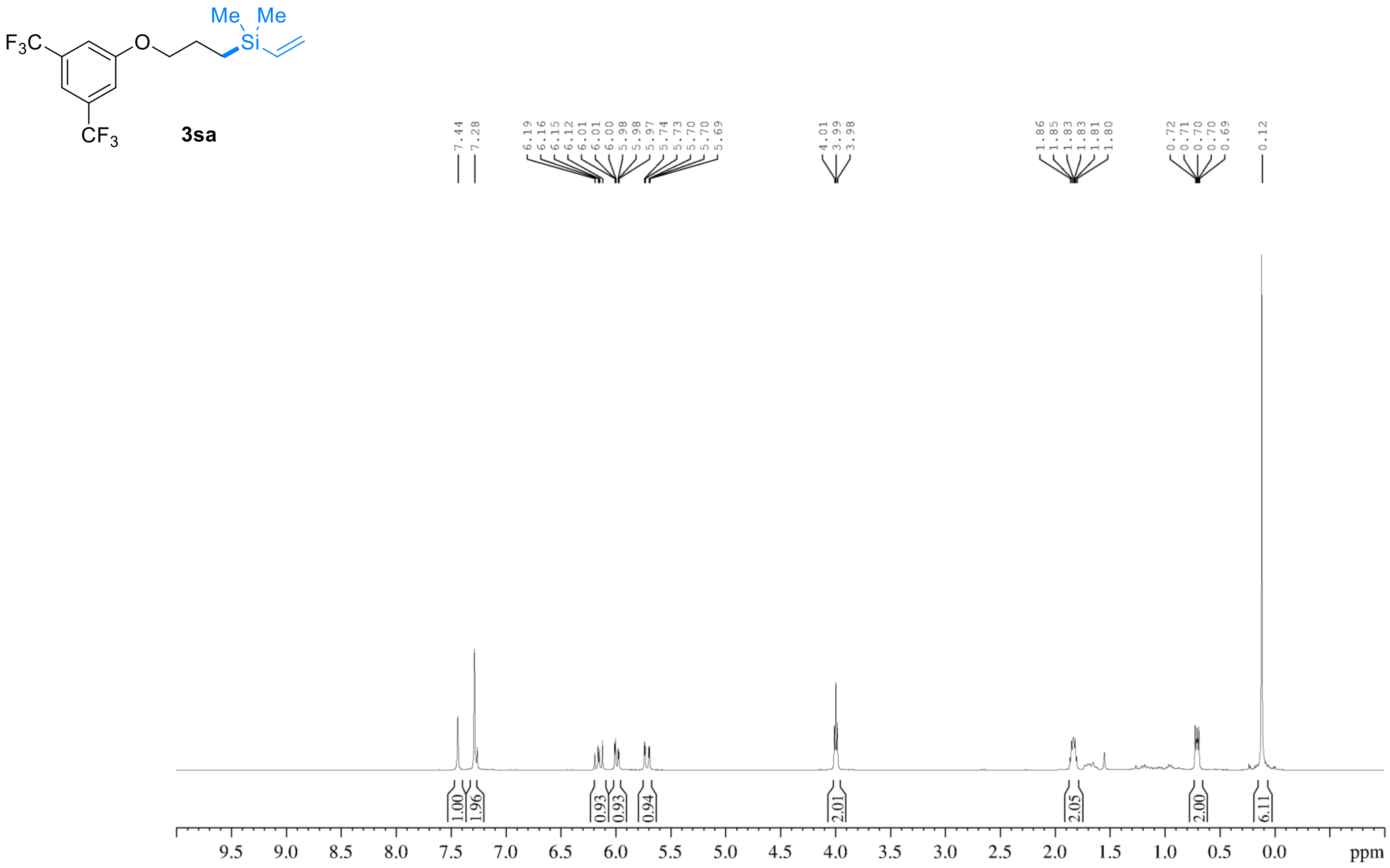

Figure S139. ^{13}C NMR (126 MHz, $CDCl_3$, 298 K) of **3sa**.

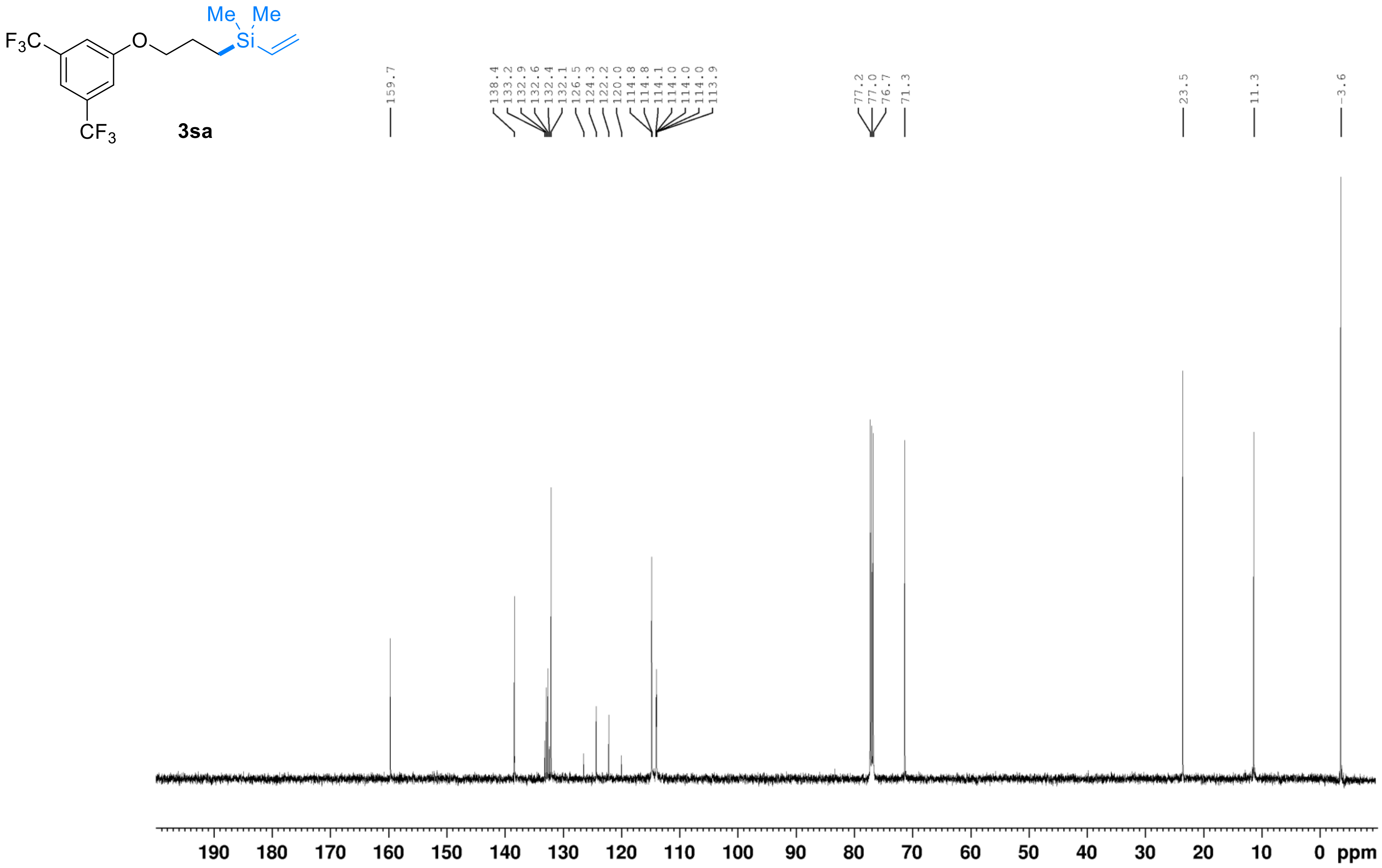

Figure S140. ^{19}F NMR (471 MHz, $CDCl_3$, 298 K) of **3sa**.

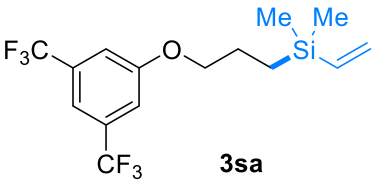

3sa

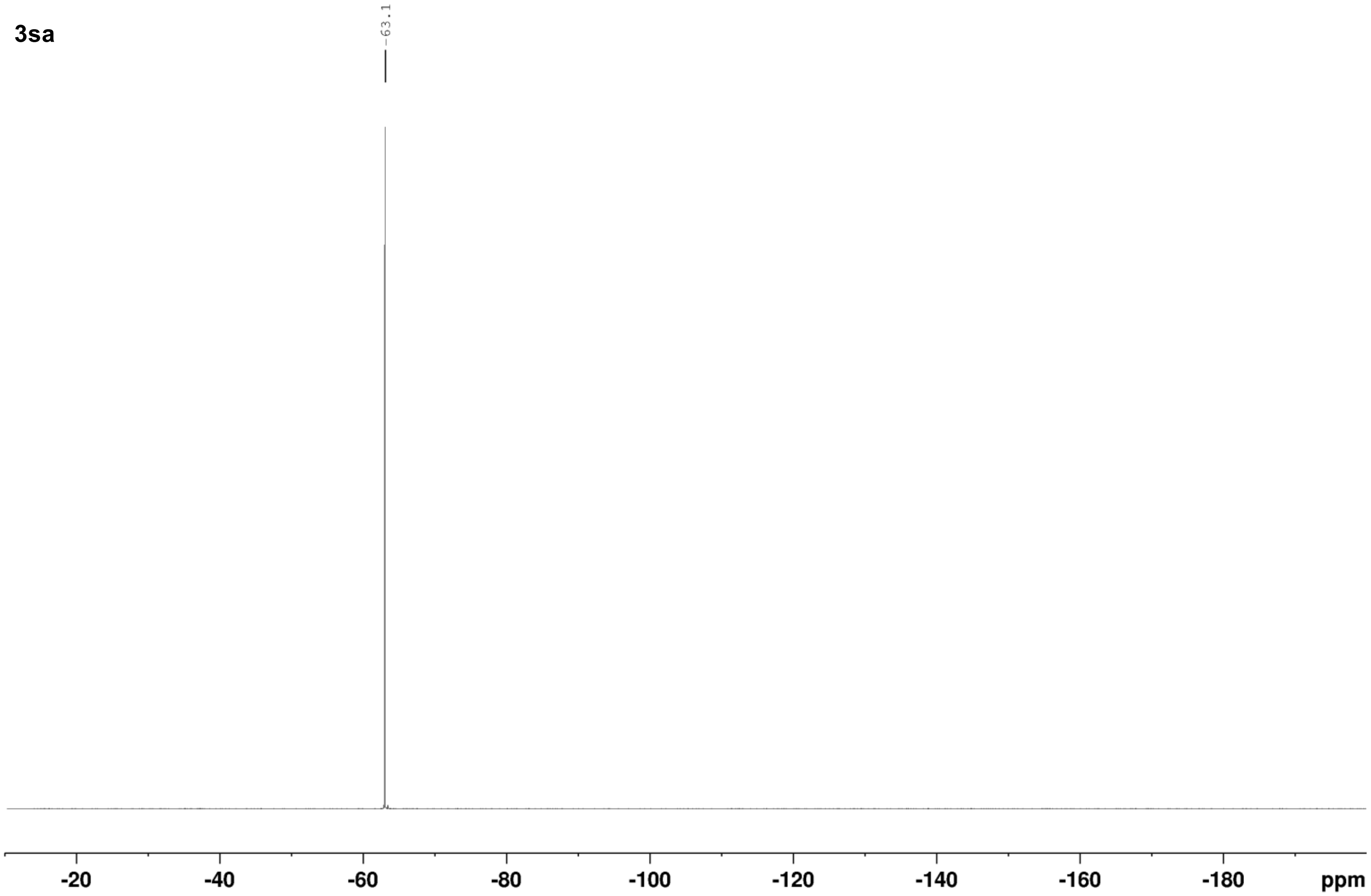

Figure S141. $^{1}H/^{29}Si$ HMQC NMR (500/99 MHz, $CDCl_3$, 298 K, optimized for J = 7 Hz) of **3sa**.

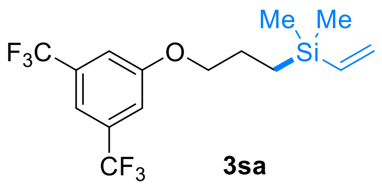

3sa

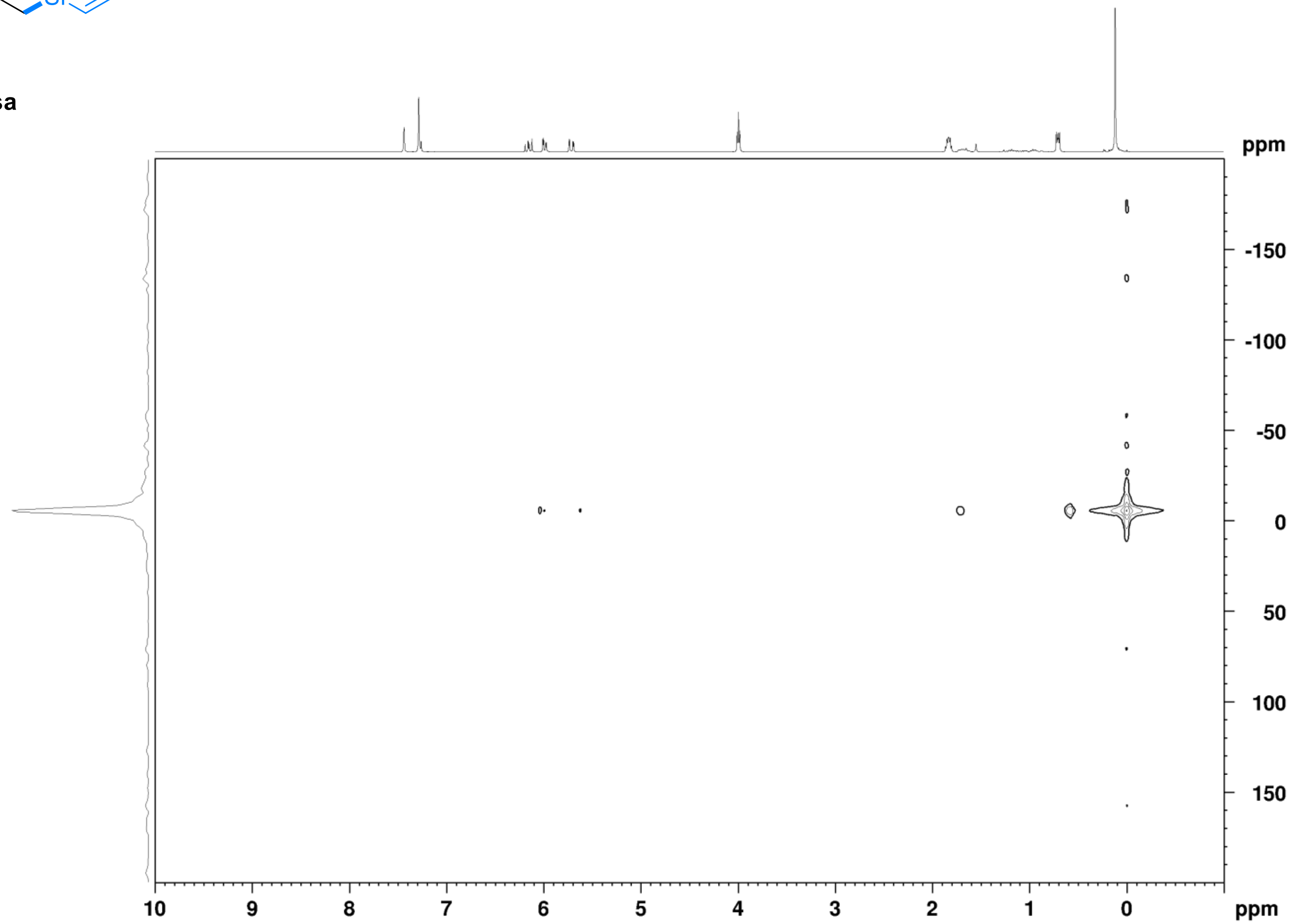

Figure S142. ^{1}H NMR (500 MHz, $CDCl_3$, 298 K) of **3ta**.

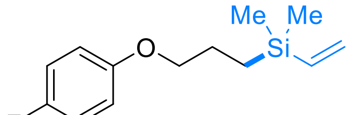

3ta

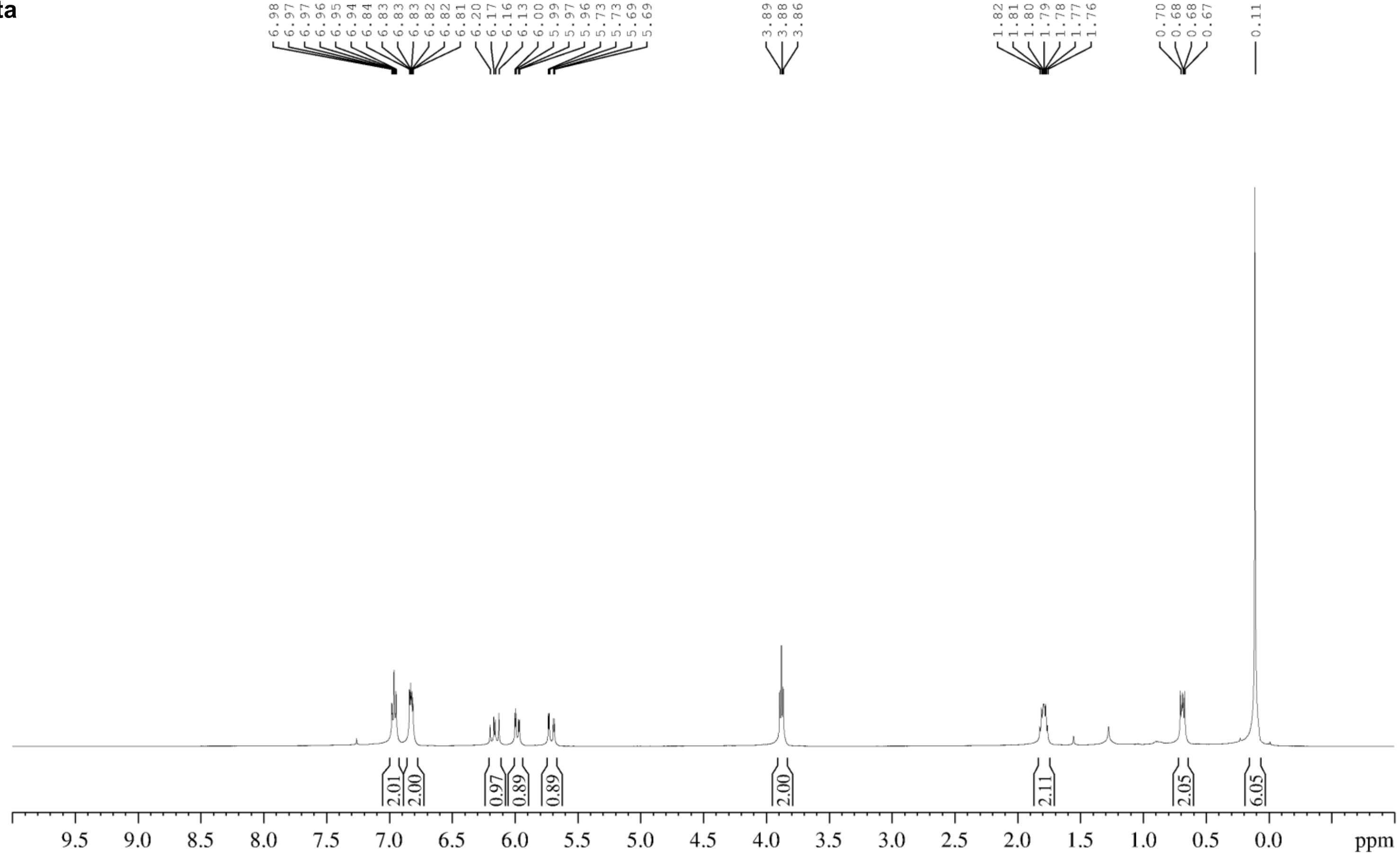

Figure S143. ^{13}C NMR (126 MHz, $CDCl_3$, 298 K) of **3ta**.

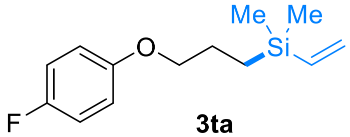

3ta

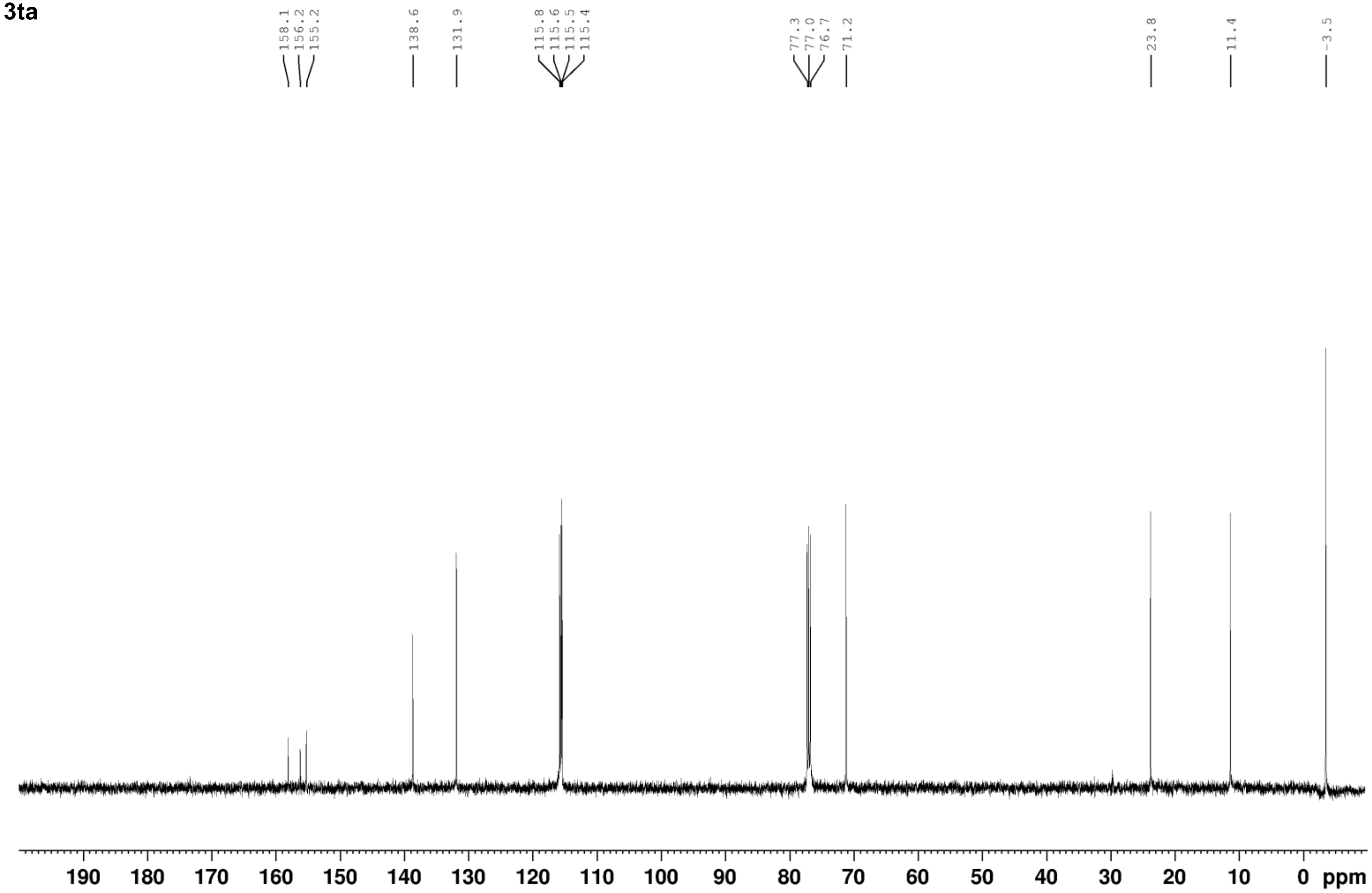

Figure S144. ^{19}F NMR (471 MHz, $CDCl_3$, 298 K) of **3ta**.

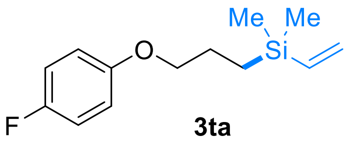

3ta

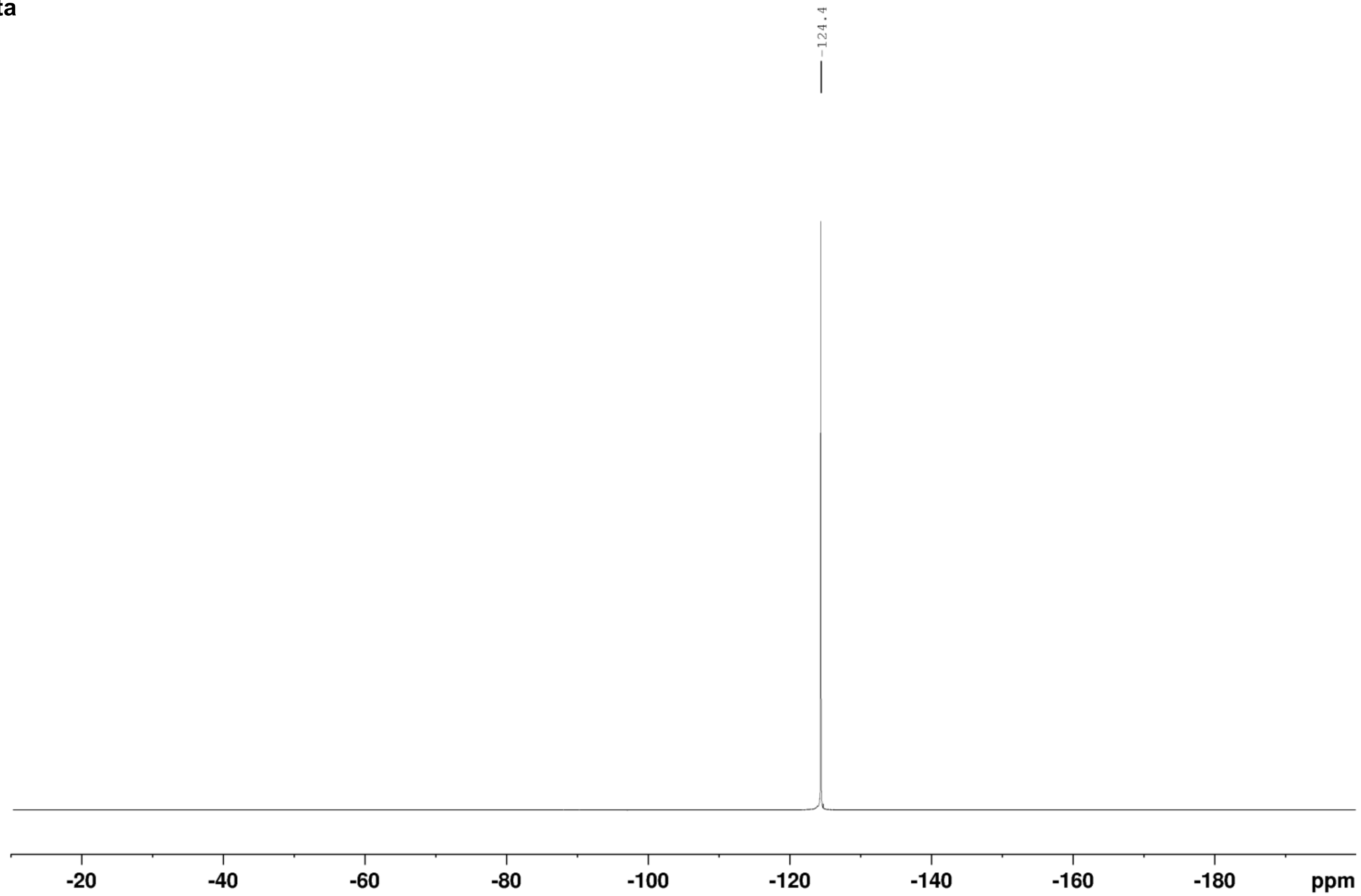

Figure S145. $^{1}H/^{29}Si$ HMQC NMR (500/99 MHz, $CDCl_3$, 298 K, optimized for J = 7 Hz) of **3ta**.

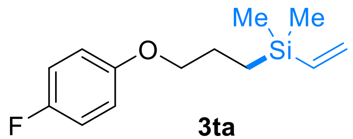

3ta

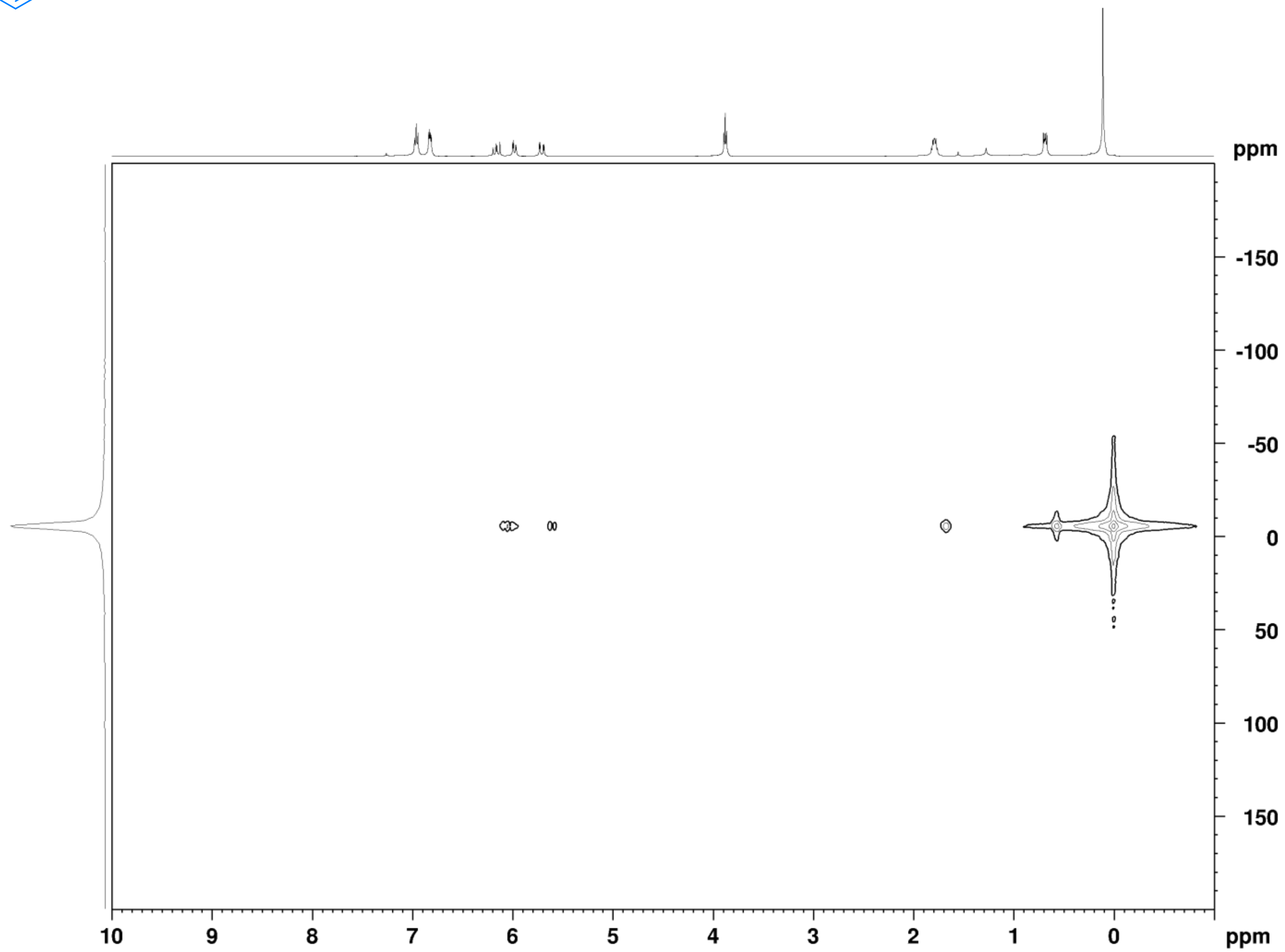

Figure S146. ^{1}H NMR (500 MHz, $CDCl_3$, 298 K) of **3ua**.

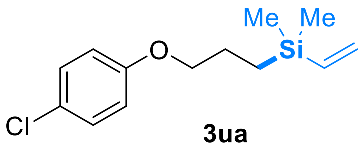

3ua

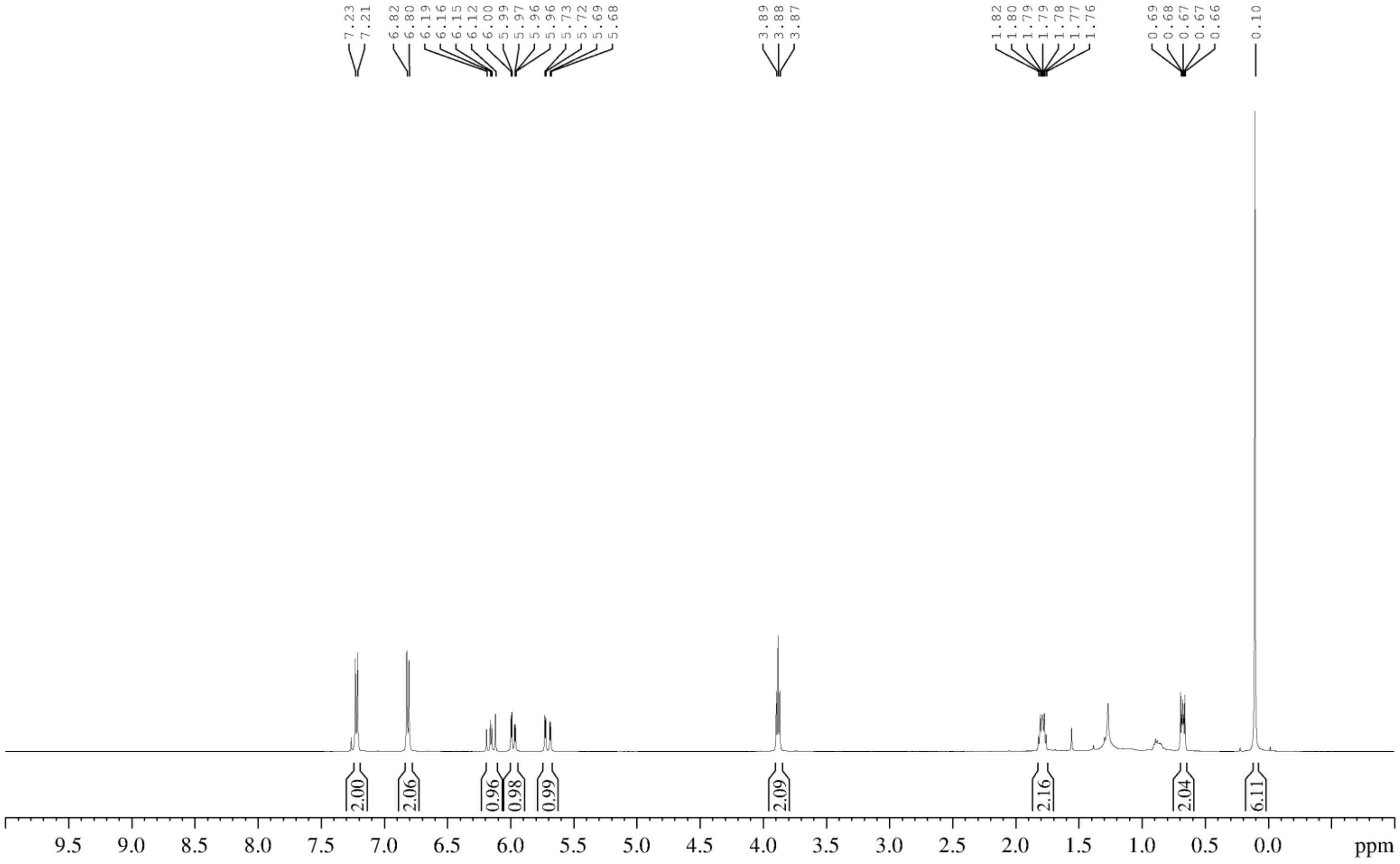

Figure S147. ^{13}C NMR (126 MHz, $CDCl_3$, 298 K) of **3ua**.

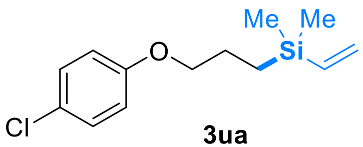

3ua

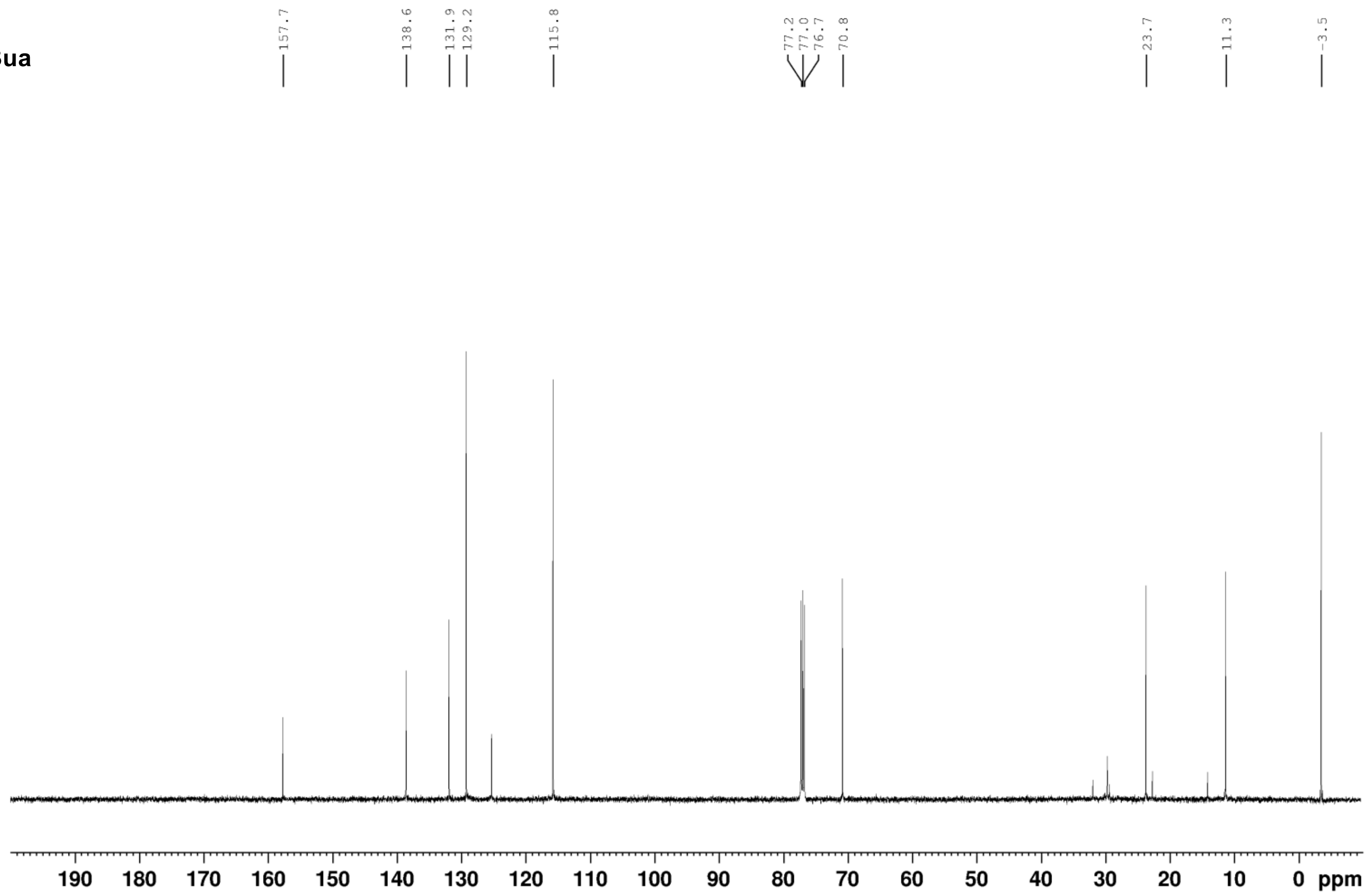

Figure S148. $^{1}H/^{29}Si$ HMQC NMR (500/99 MHz, $CDCl_3$, 298 K, optimized for J = 7 Hz) of **3ua**.

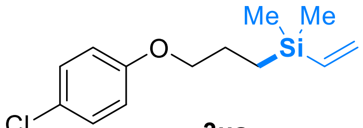

3ua

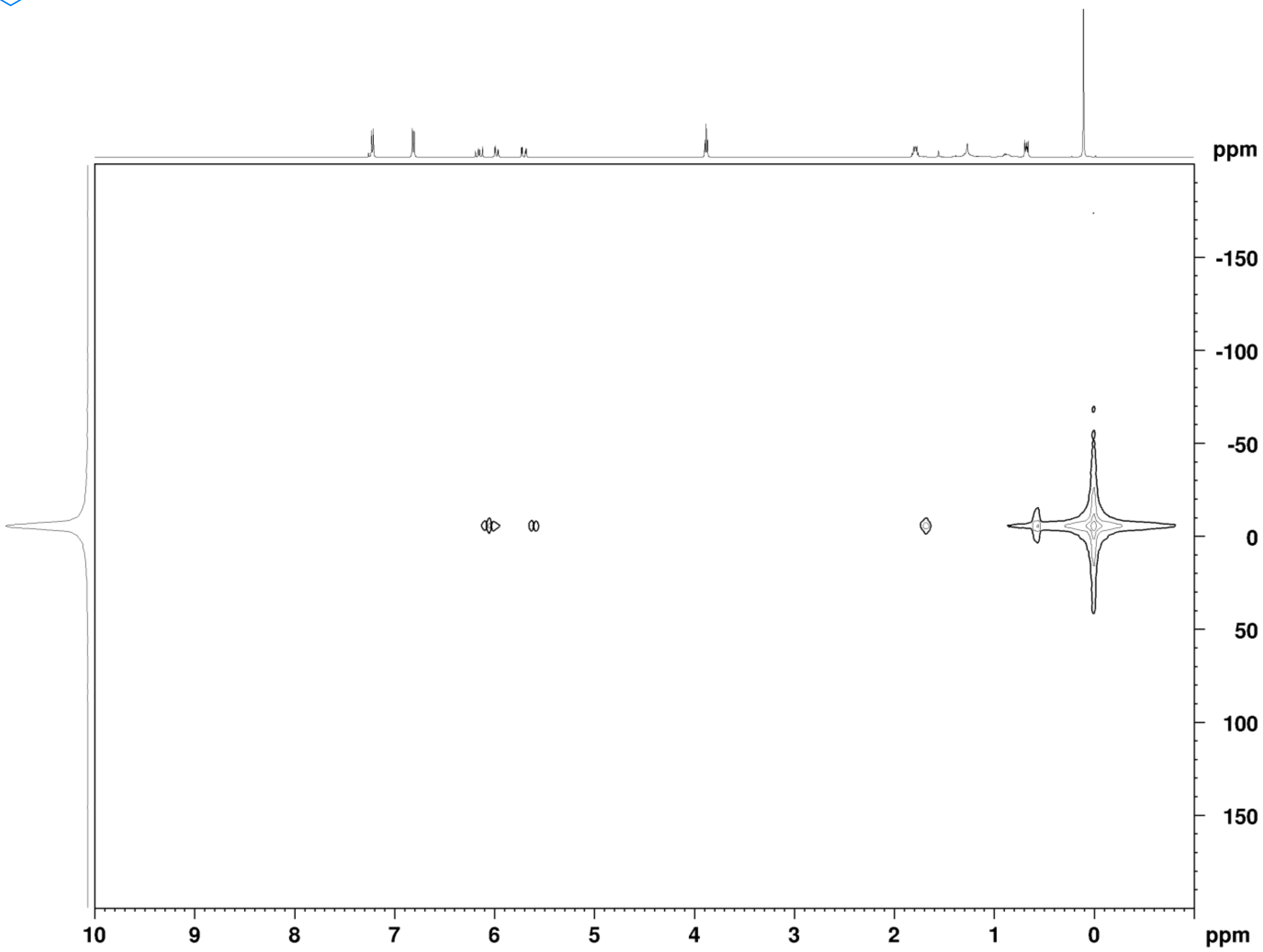

Figure S149. ^{1}H NMR (500 MHz, $CDCl_3$, 298 K) of **3va**.

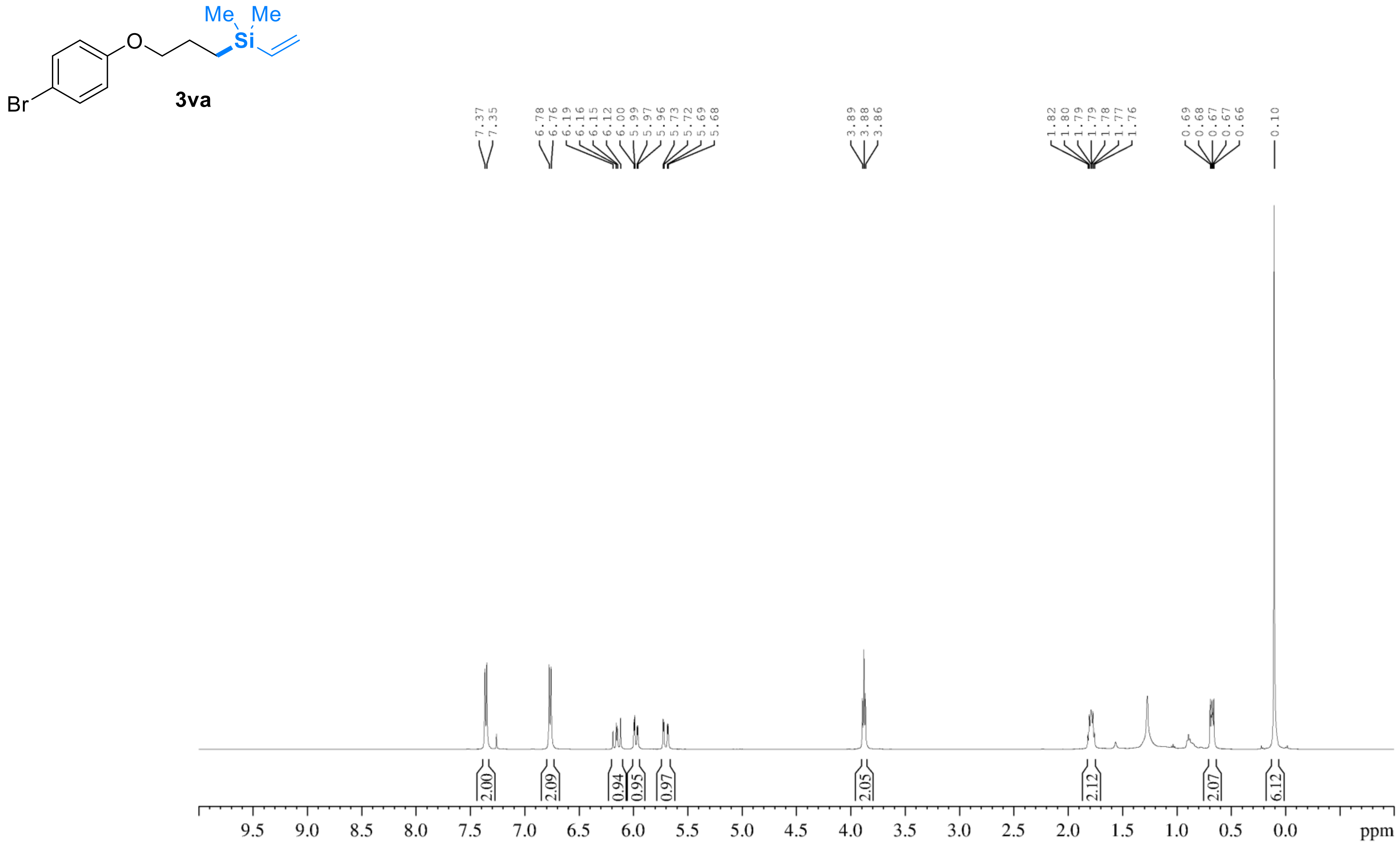

Figure S150. ^{13}C NMR (126 MHz, $CDCl_3$, 298 K) of **3va**.

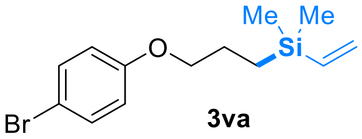

3va

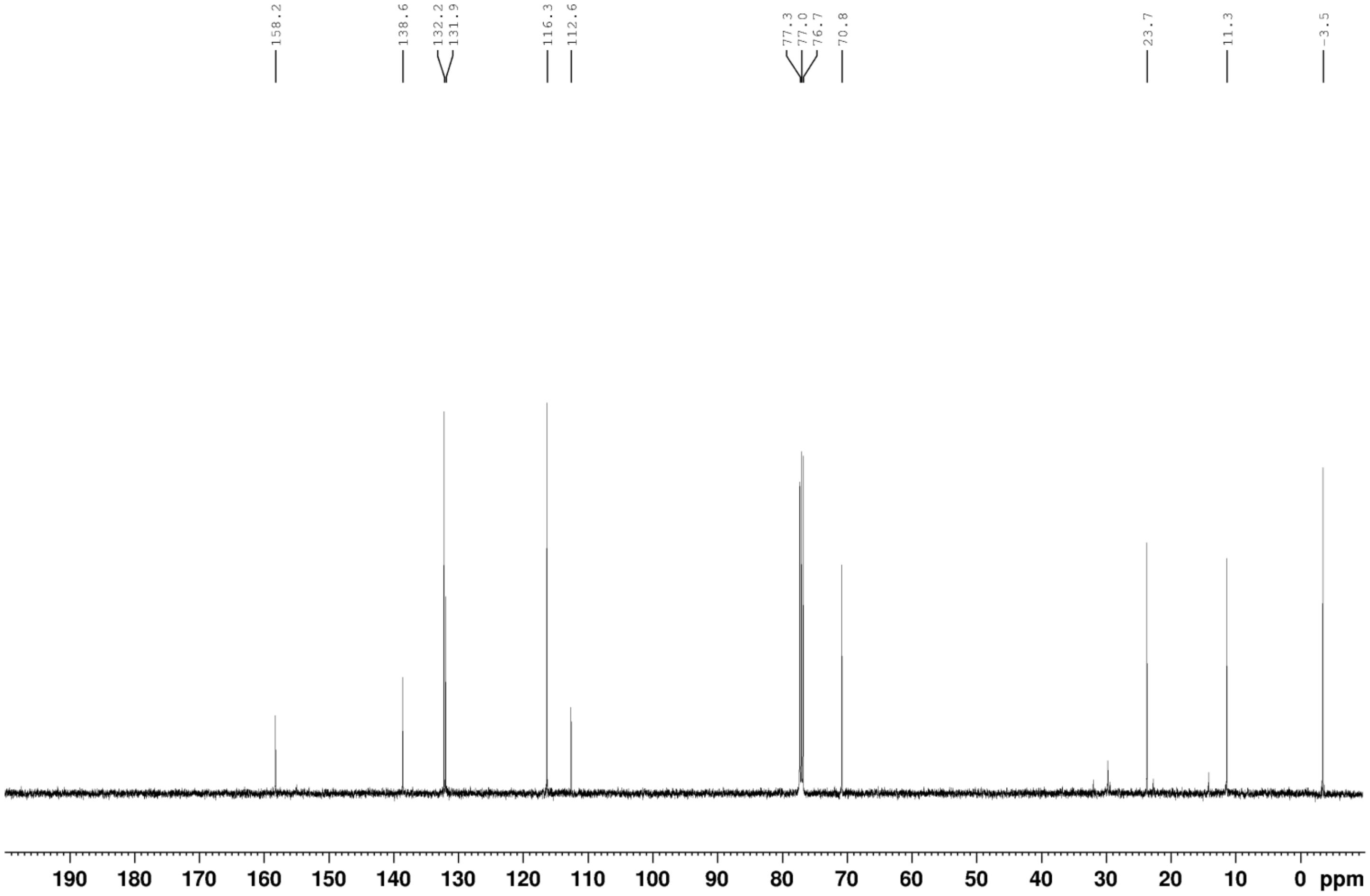

Figure S151. $^{1}H/^{29}Si$ HMQC NMR (500/99 MHz, $CDCl_3$, 298 K, optimized for J = 7 Hz) of **3va**.

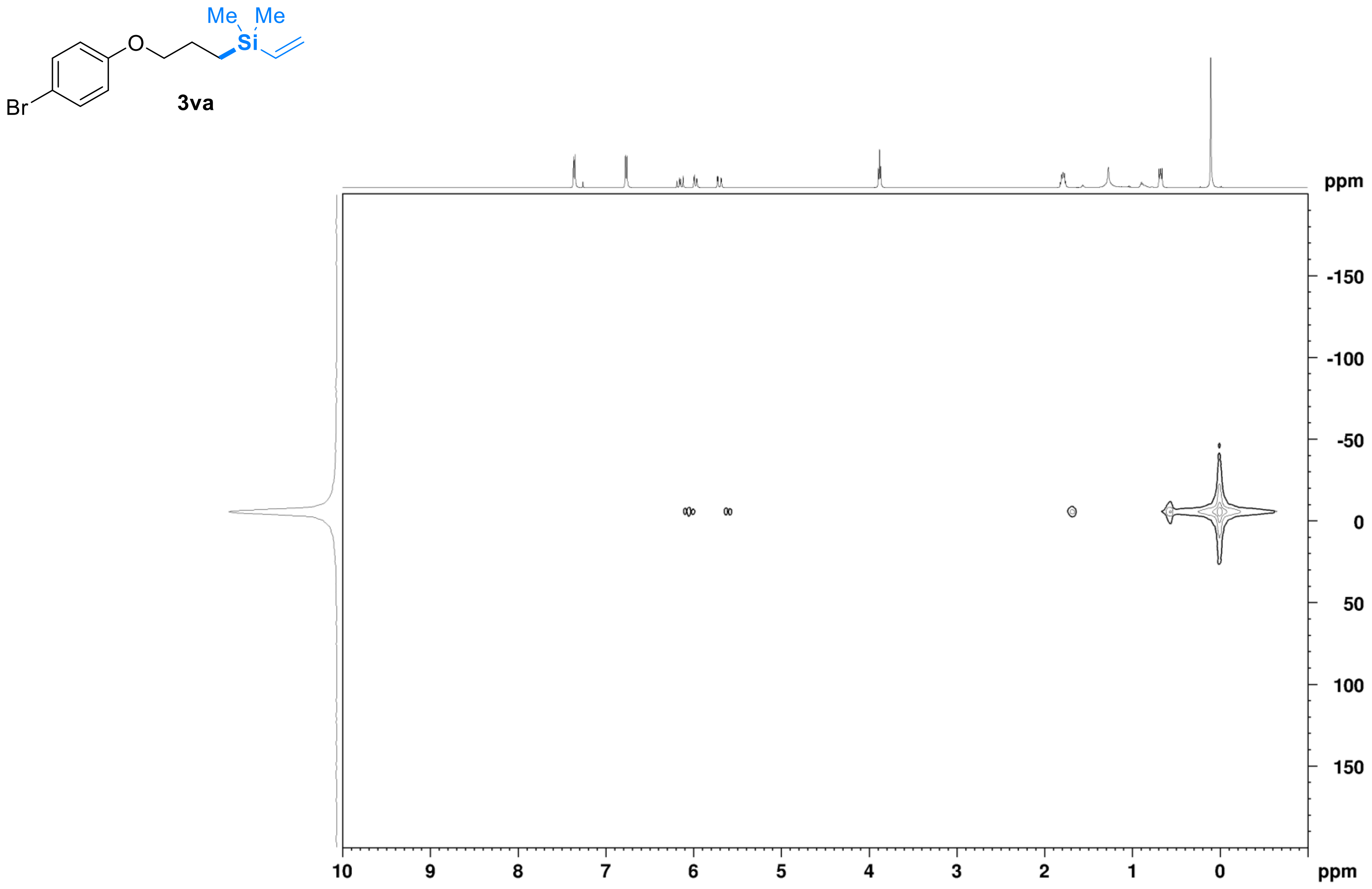

Figure S152. ^{1}H NMR (500 MHz, $CDCl_3$, 298 K) of **3wa**.

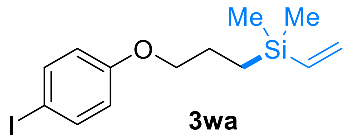

3wa

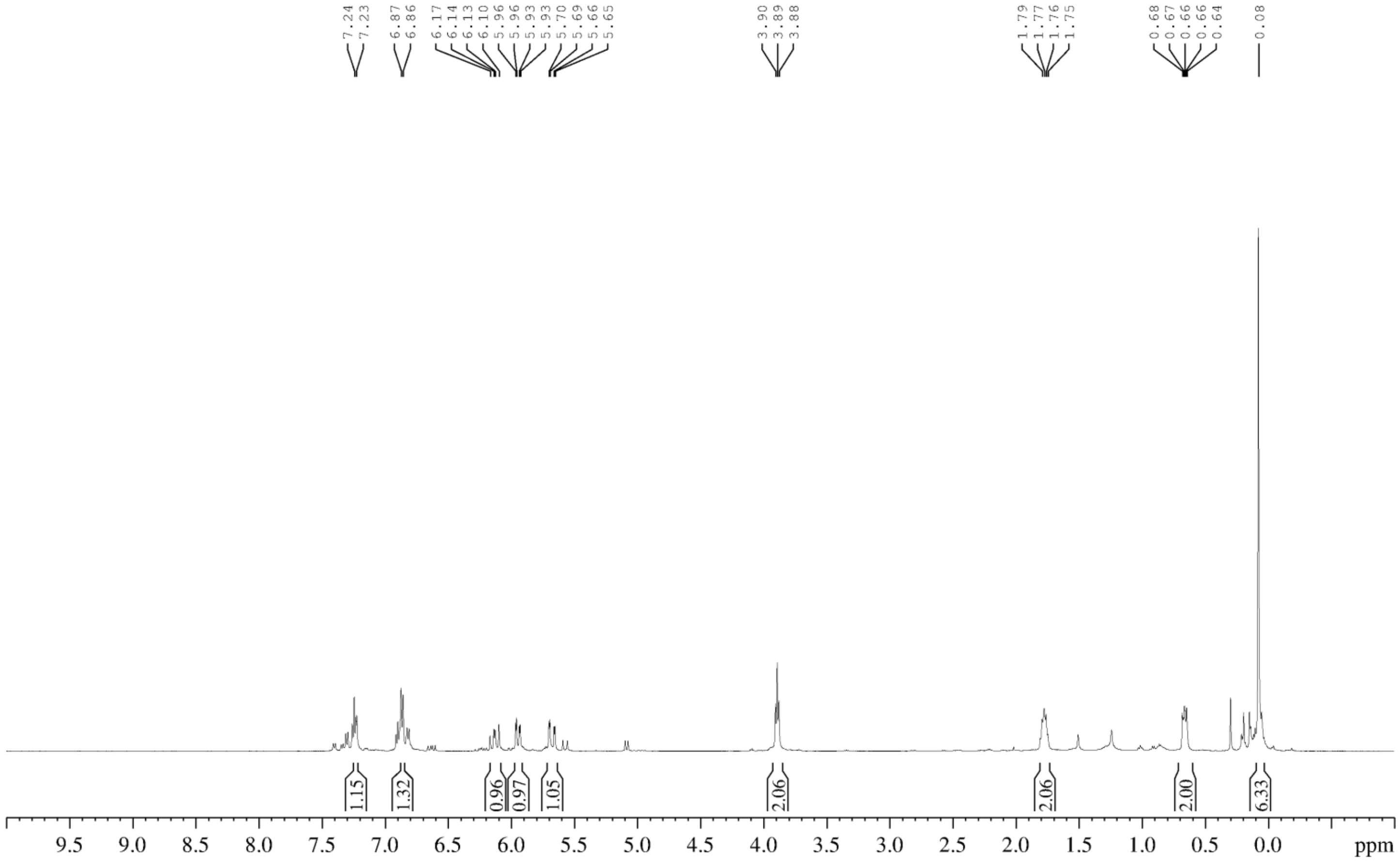

Figure S153. ^{13}C NMR (126 MHz, $CDCl_3$, 298 K) of **3wa**.

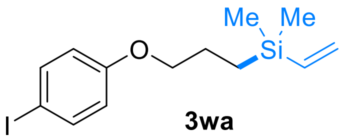

3wa

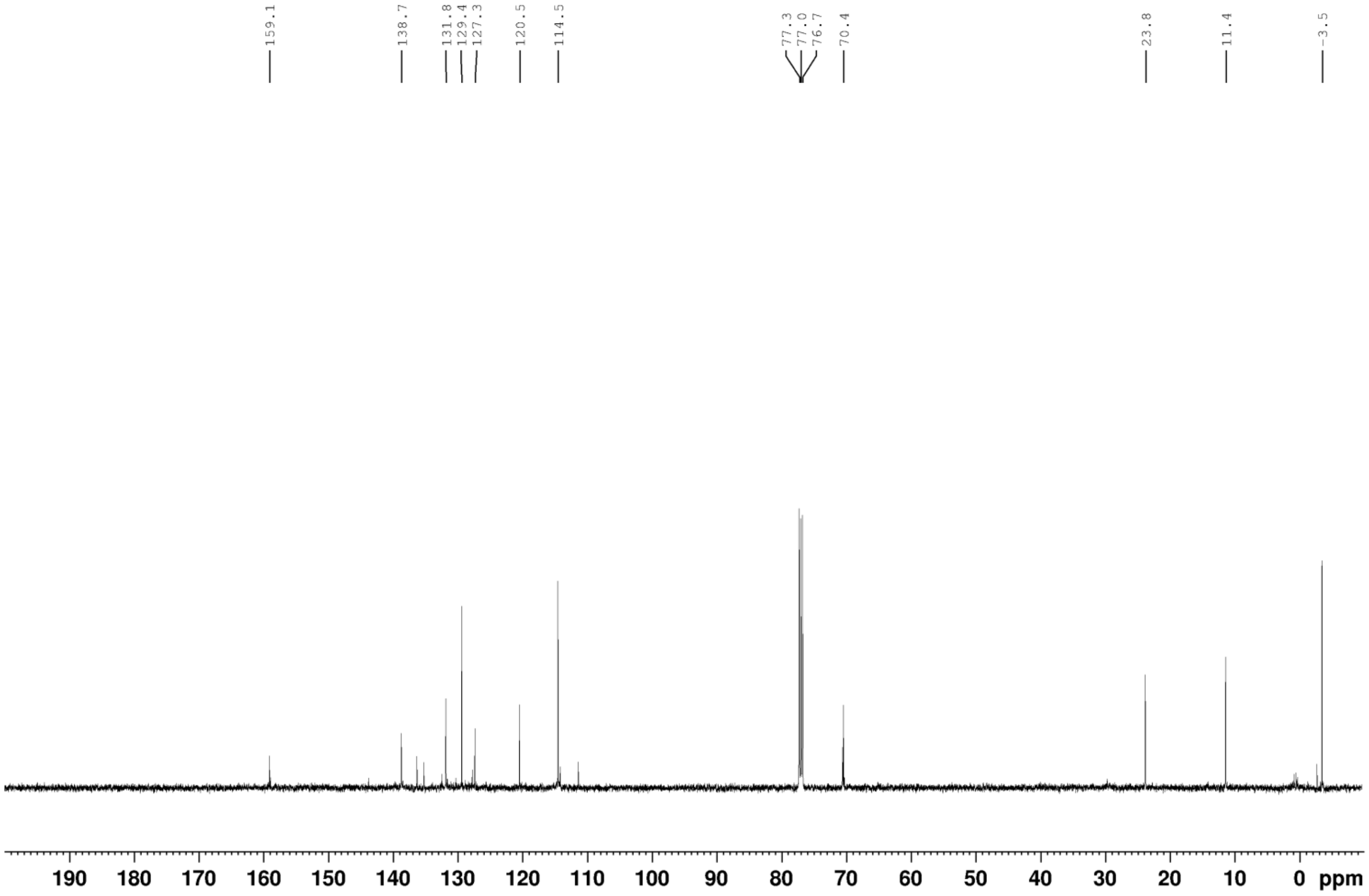

Figure S154. $^{1}H/^{29}Si$ HMQC NMR (500/99 MHz, $CDCl_3$, 298 K, optimized for J = 7 Hz) of **3wa**.

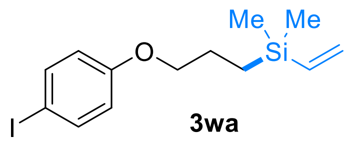

3wa

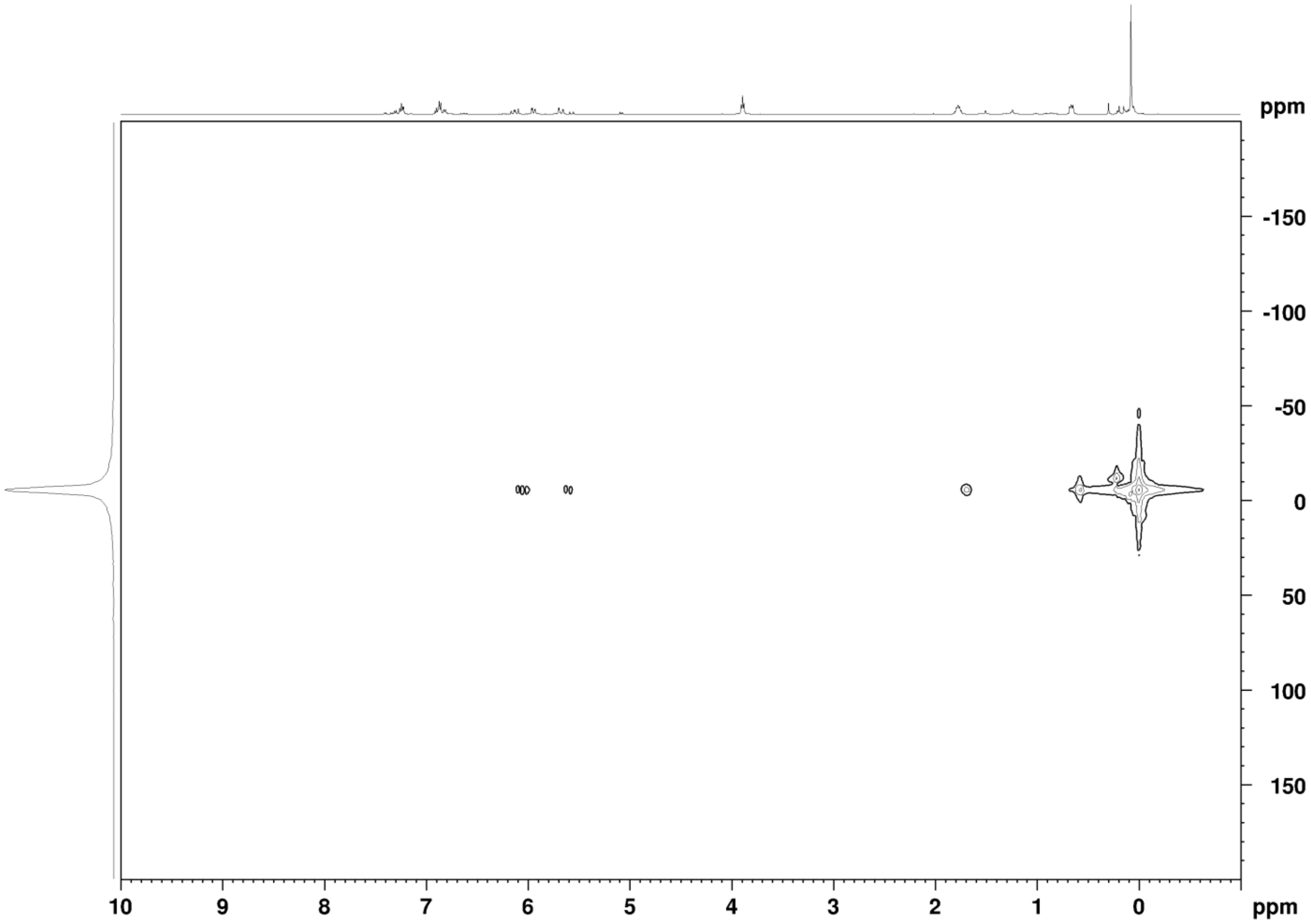

Figure S155. ^{1}H NMR (500 MHz, $CDCl_3$, 298 K) of **3xa**.

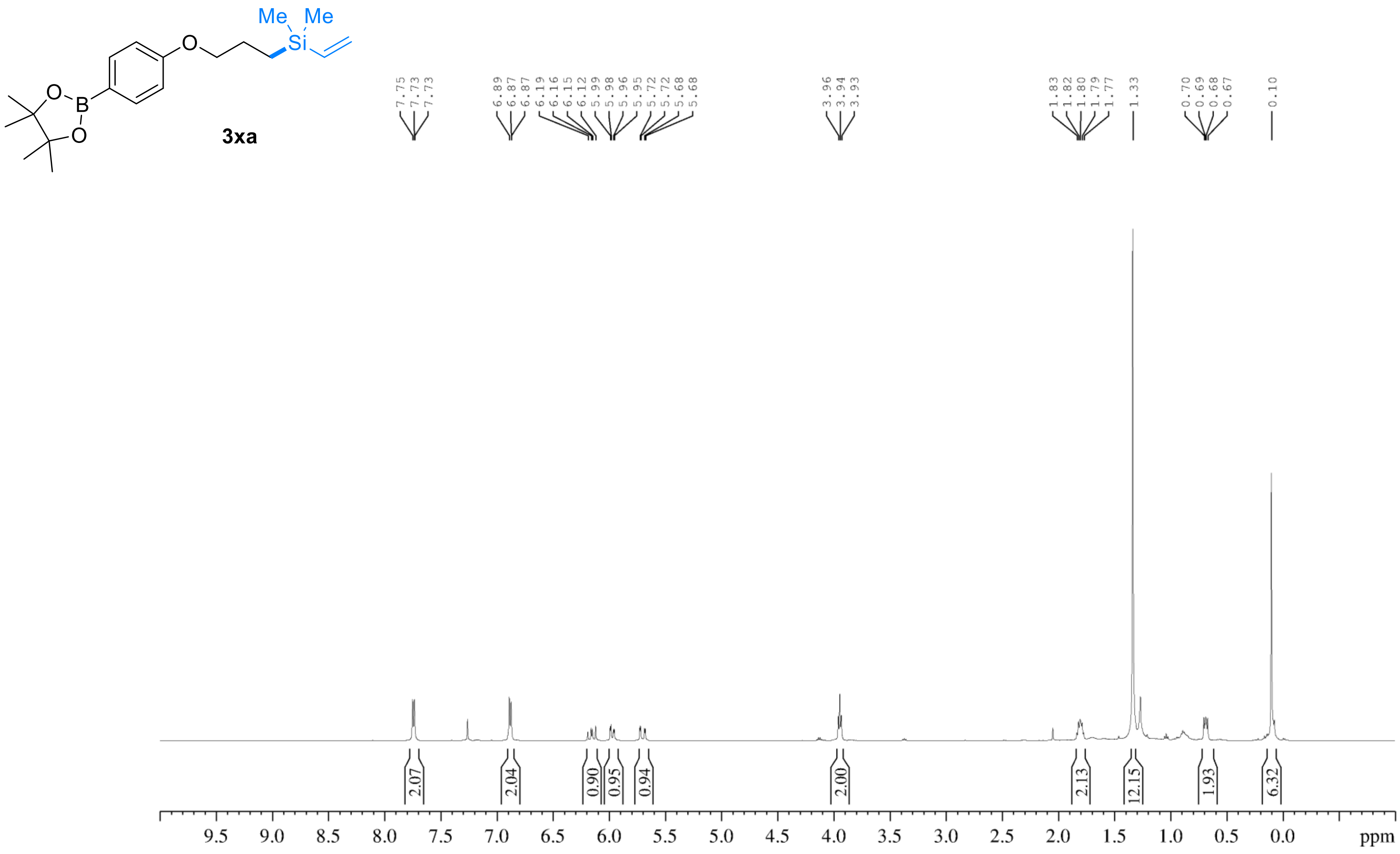

Figure S156. ^{13}C NMR (126 MHz, $CDCl_3$, 298 K) of **3xa**.

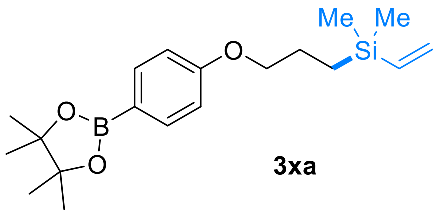

3xa

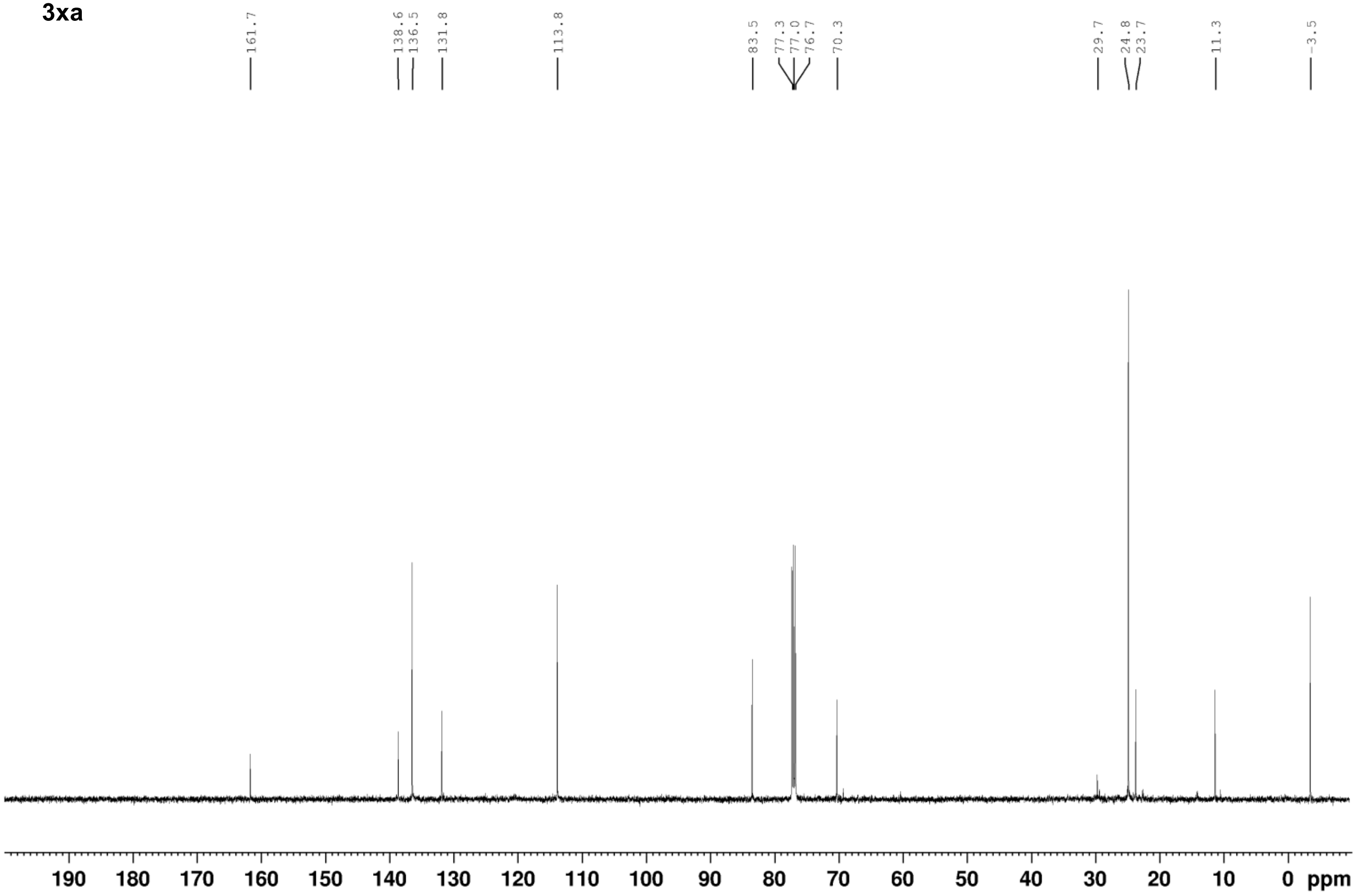

Figure S157. $^{1}H/^{29}Si$ HMQC NMR (500/99 MHz, $CDCl_3$, 298 K, optimized for J = 7 Hz) of **3xa**.

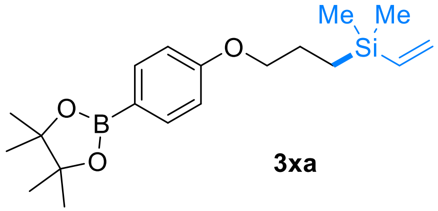

3xa

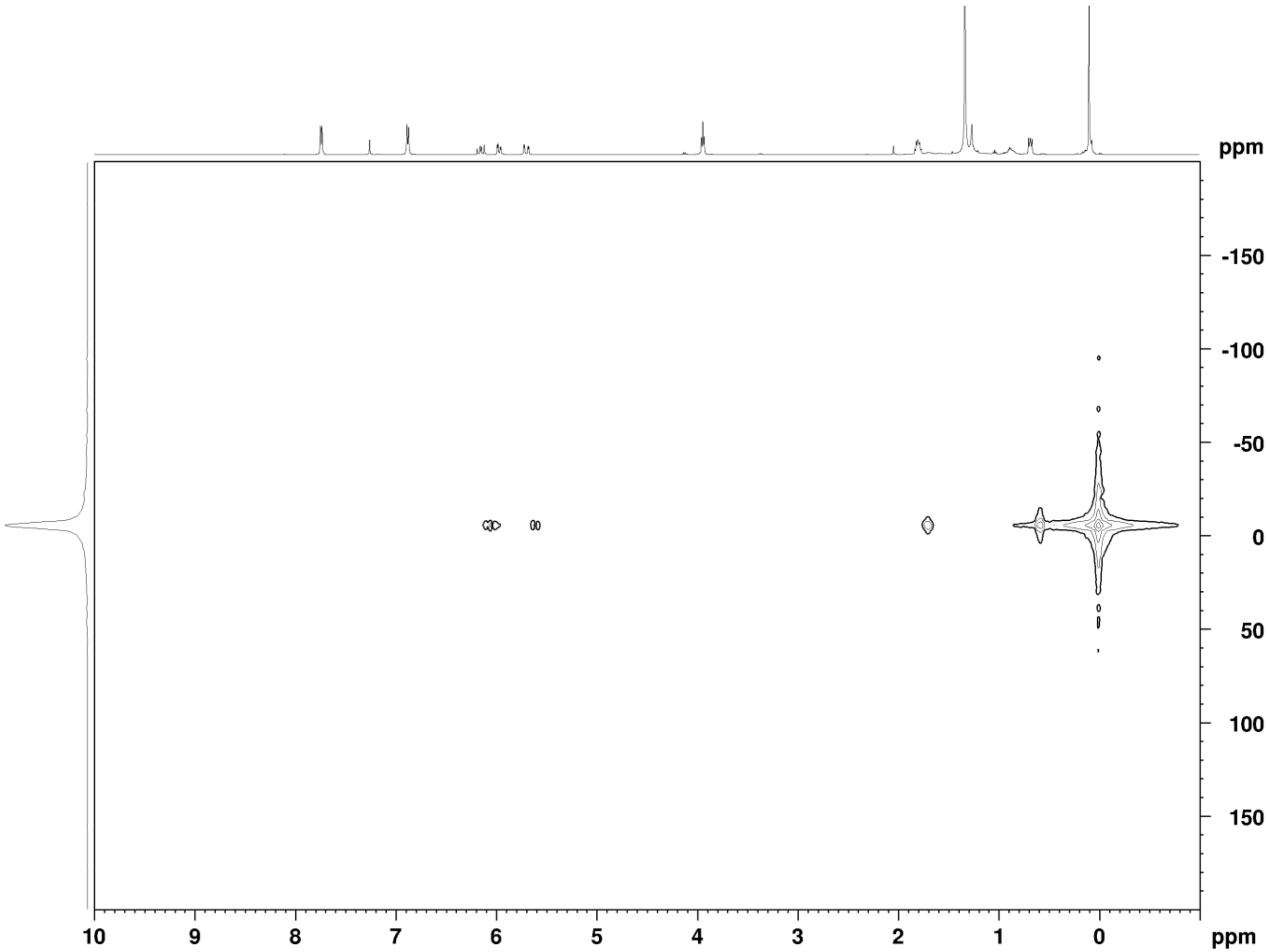

Figure S158. ^{1}H NMR (500 MHz, $CDCl_3$, 298 K) of **3ya**.

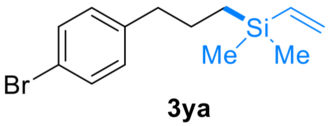

3ya

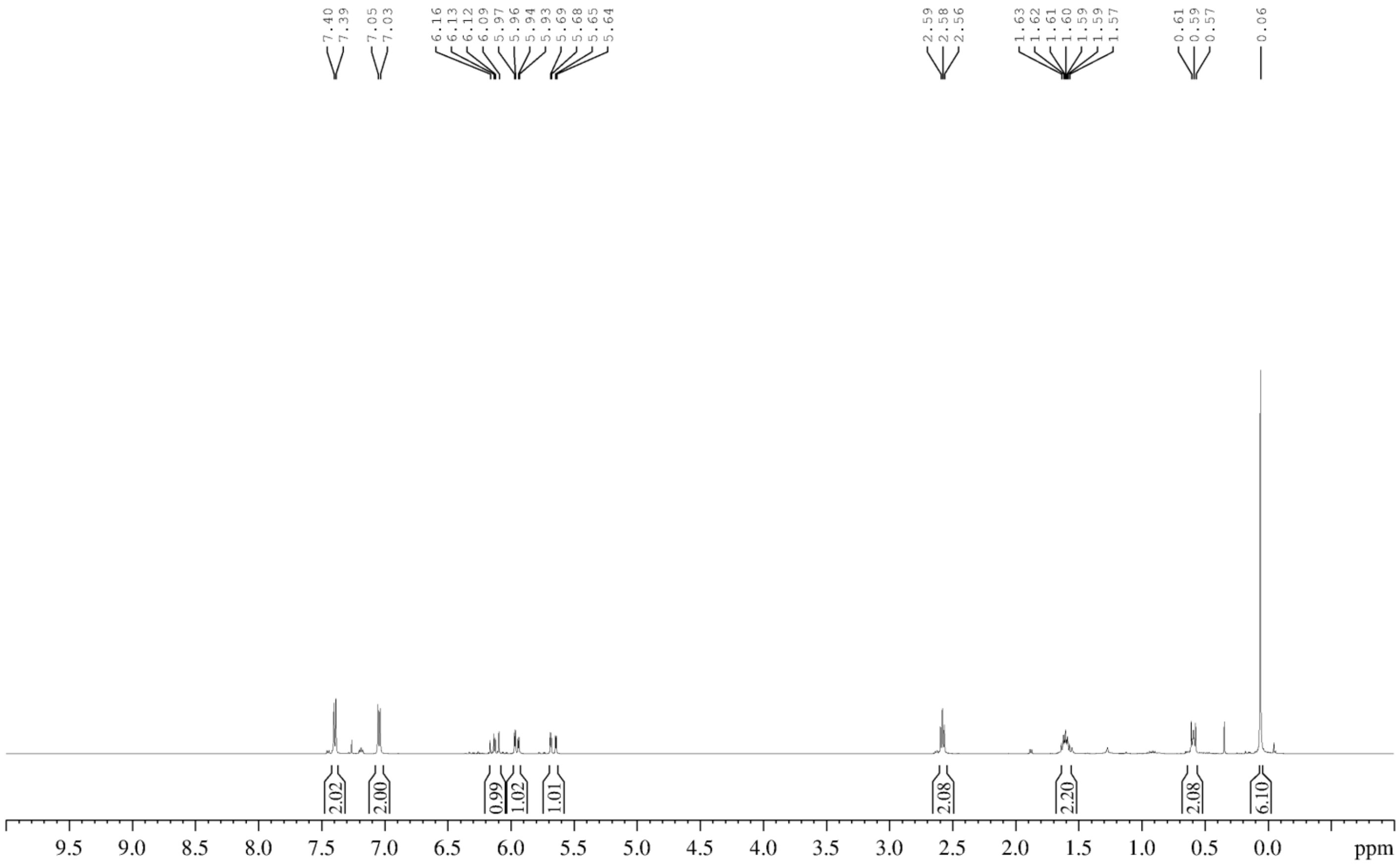

Figure S159. ^{13}C NMR (126 MHz, $CDCl_3$, 298 K) of **3ya**.

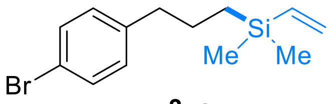

3ya

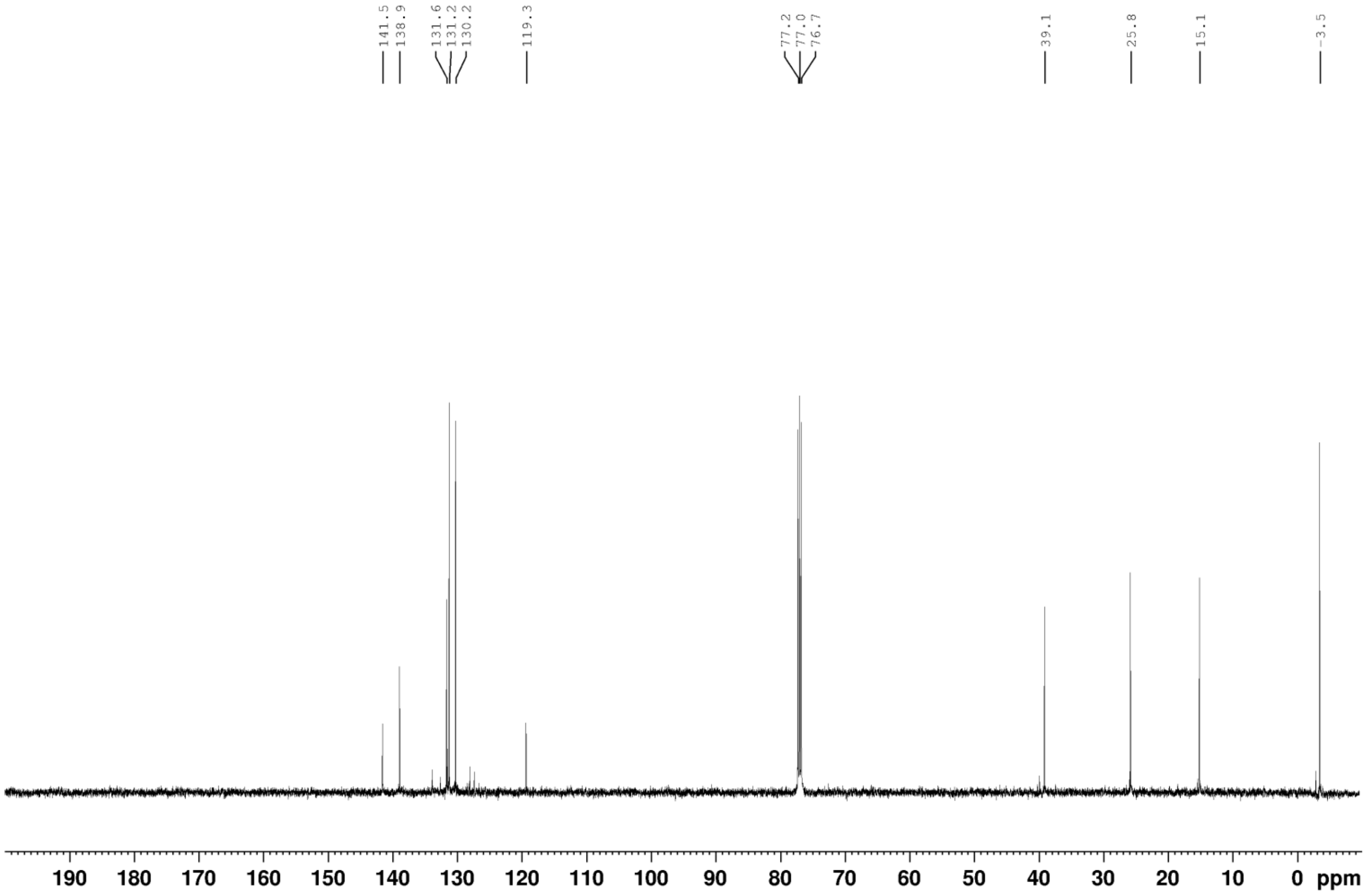

Figure S160. $^{1}H/^{29}Si$ HMQC NMR (500/99 MHz, $CDCl_3$, 298 K, optimized for J = 7 Hz) of **3ya**.

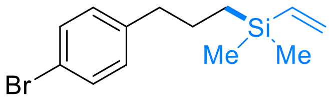

3ya

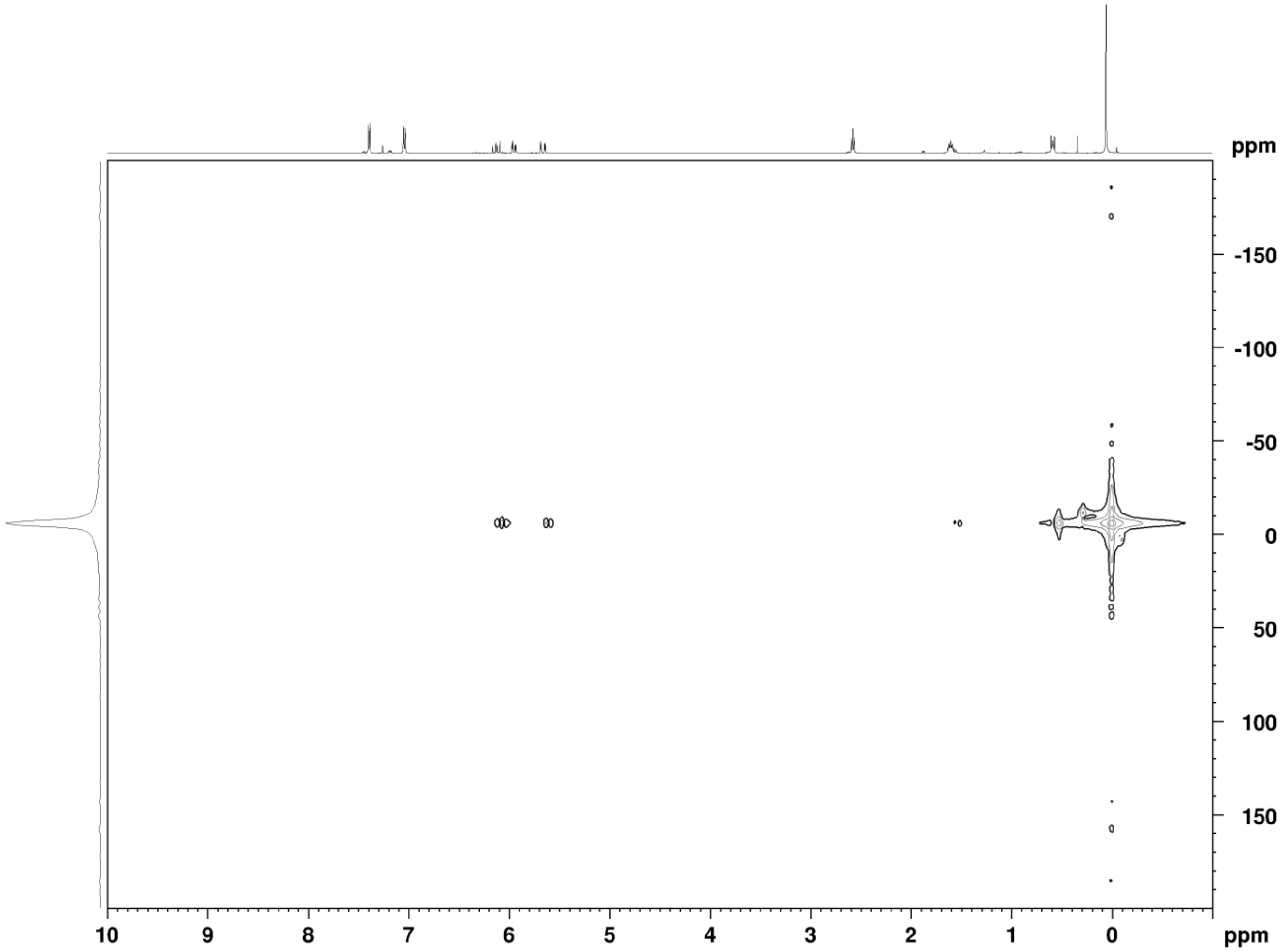

Figure S161. ^{1}H NMR (500 MHz, $CDCl_3$, 298 K) of **3za**.

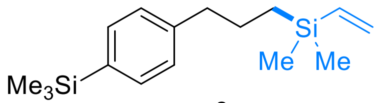

3za

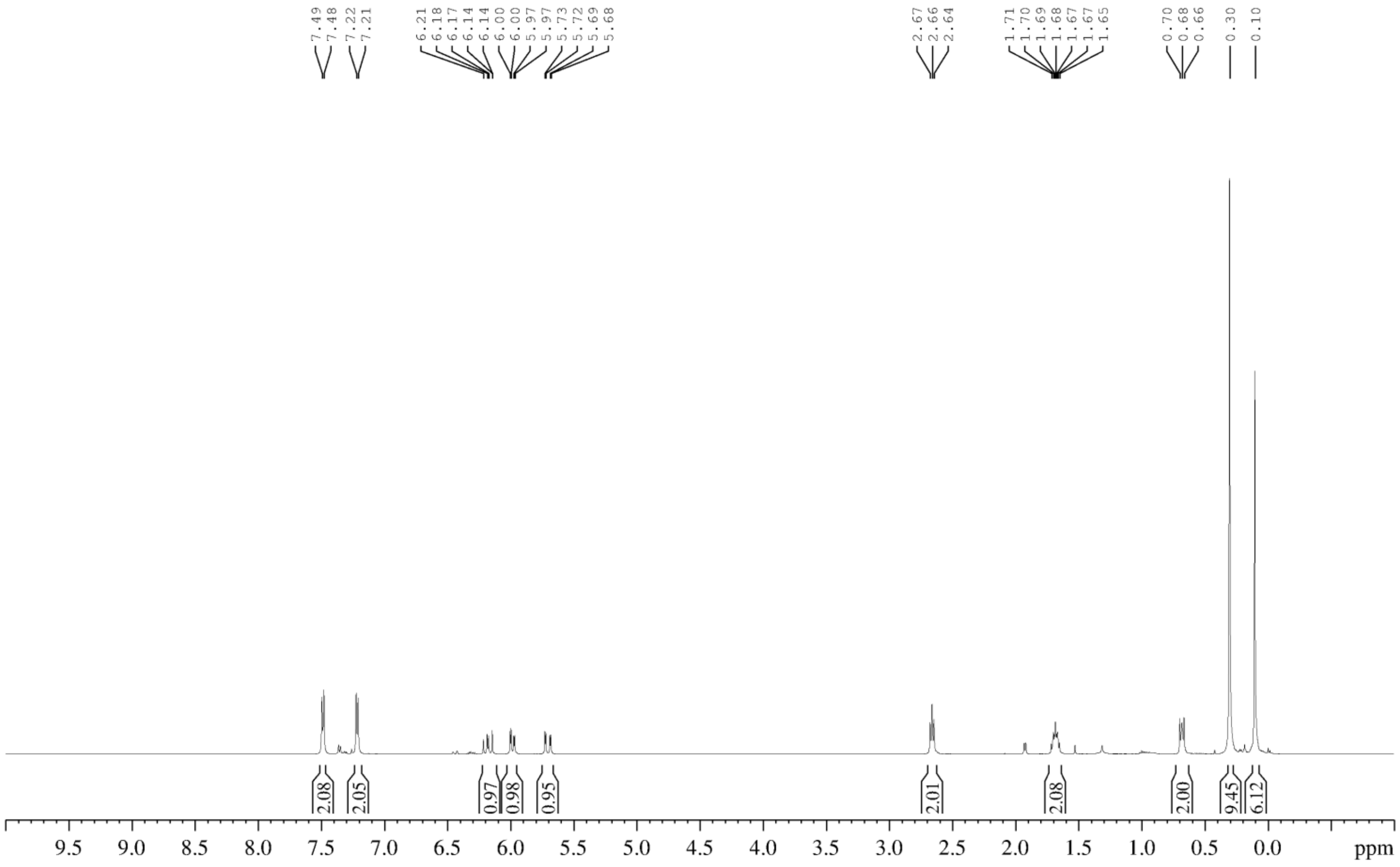

Figure S162. ^{13}C NMR (126 MHz, $CDCl_3$, 298 K) of **3za**.

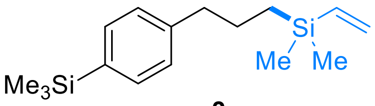

3za

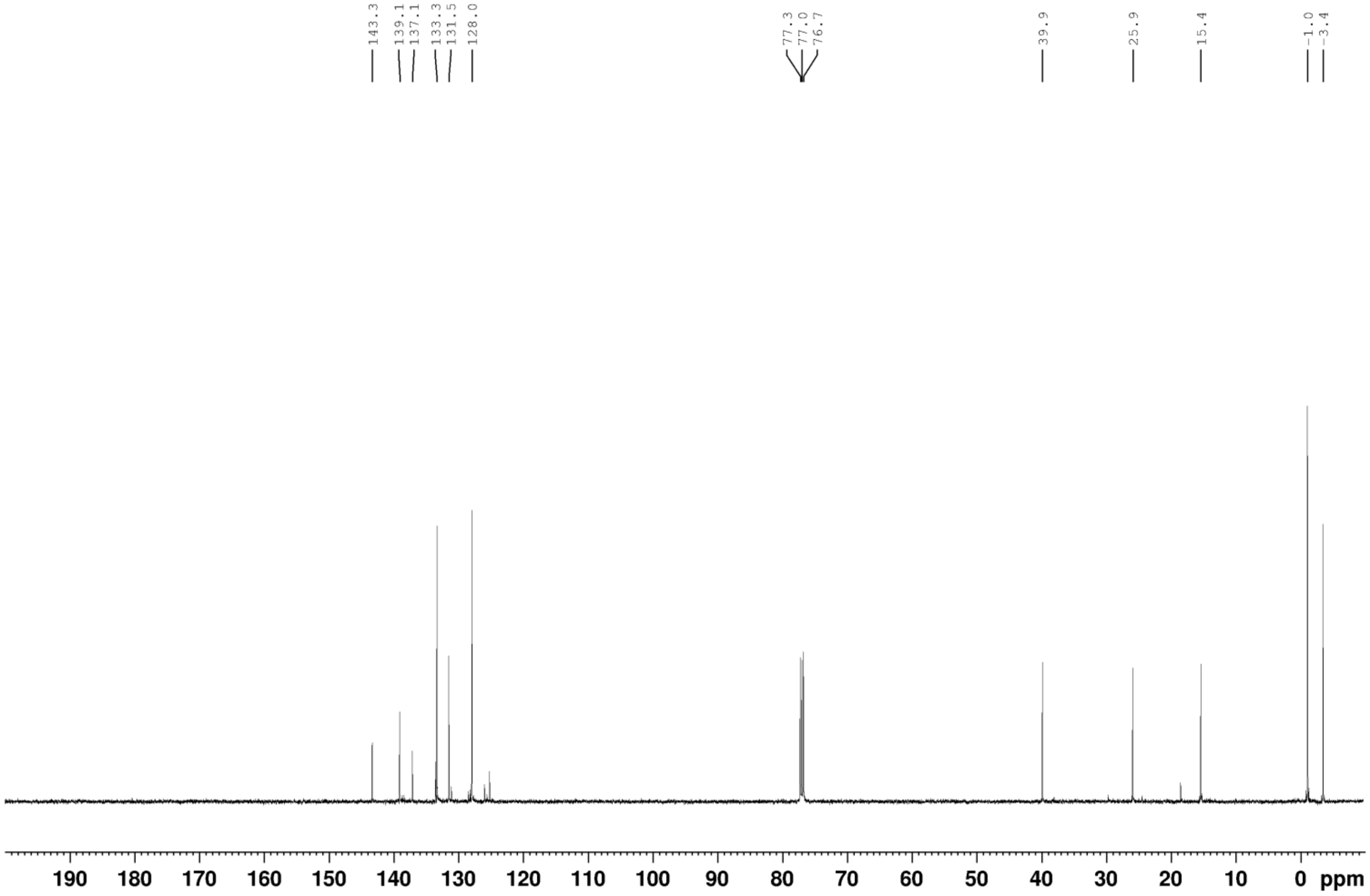

Figure S163. $^{1}H/^{29}Si$ HMQC NMR (500/99 MHz, $CDCl_3$, 298 K, optimized for J = 7 Hz) of **3za**.

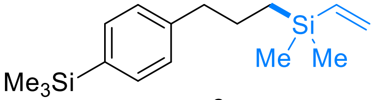

3za

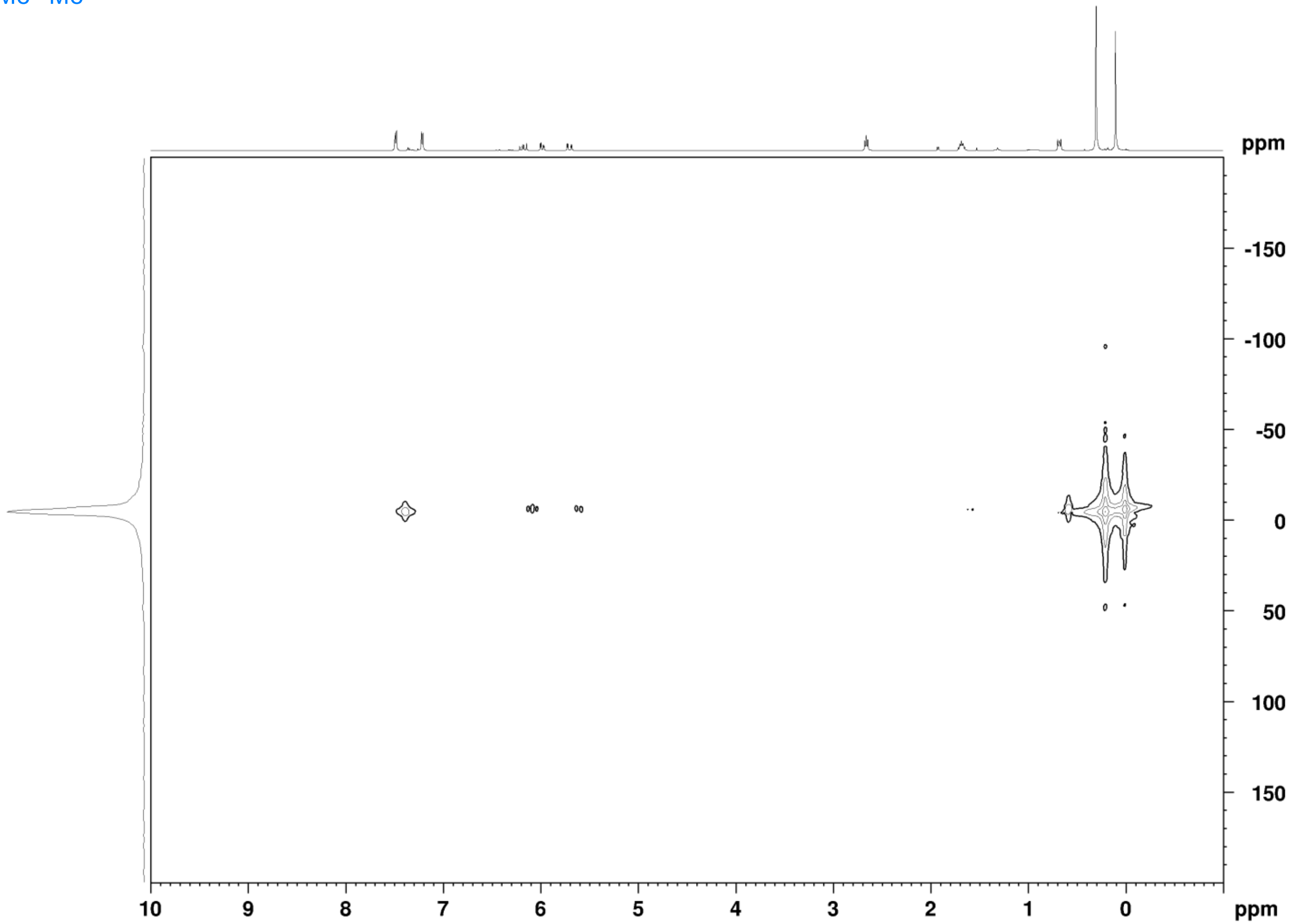

Figure S164. ^{1}H NMR (500 MHz, $CDCl_3$, 298 K) of **5aa**.

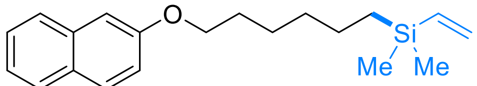

5aa

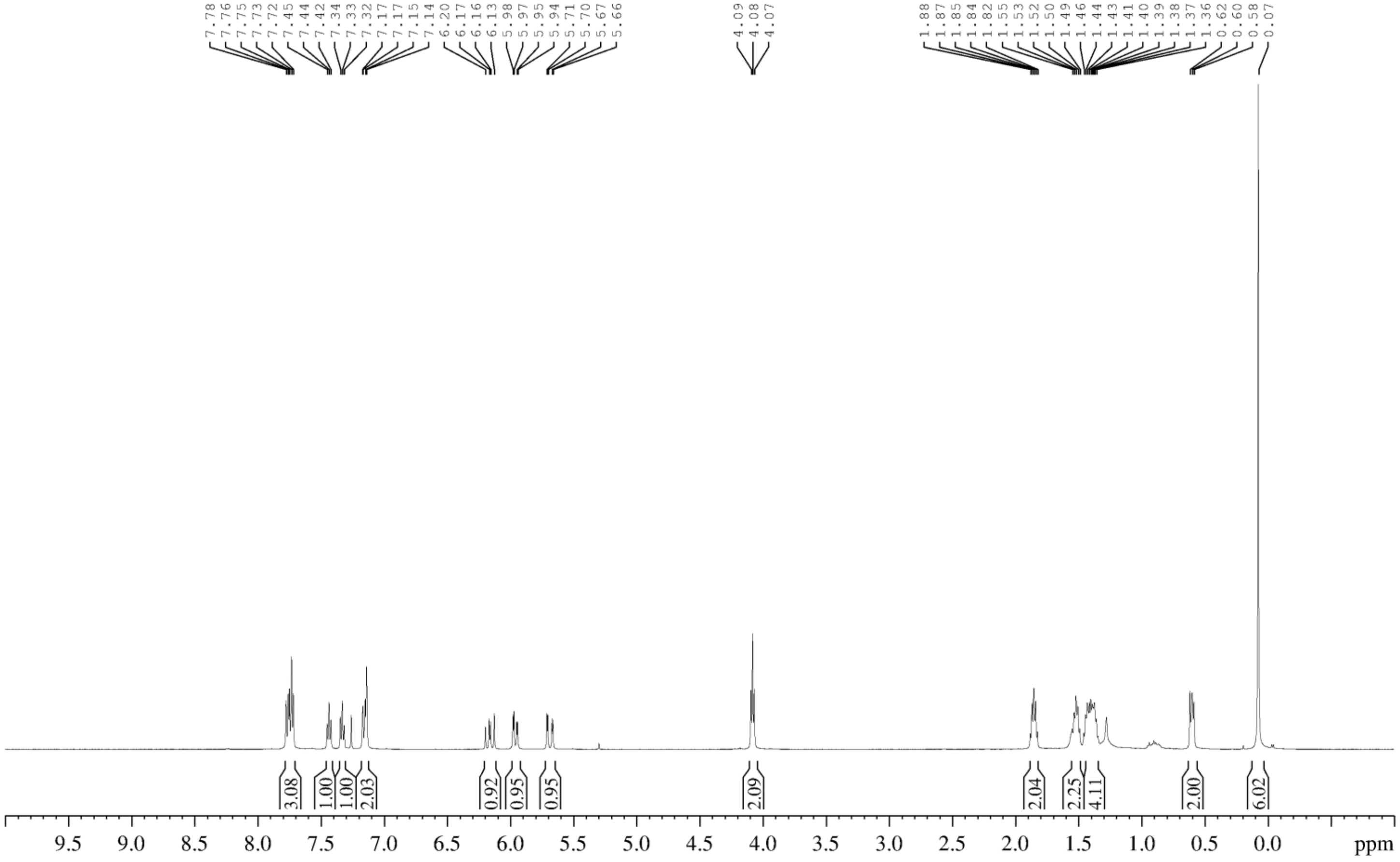

Figure S165. ^{13}C NMR (126 MHz, $CDCl_3$, 298 K) of **5aa**.

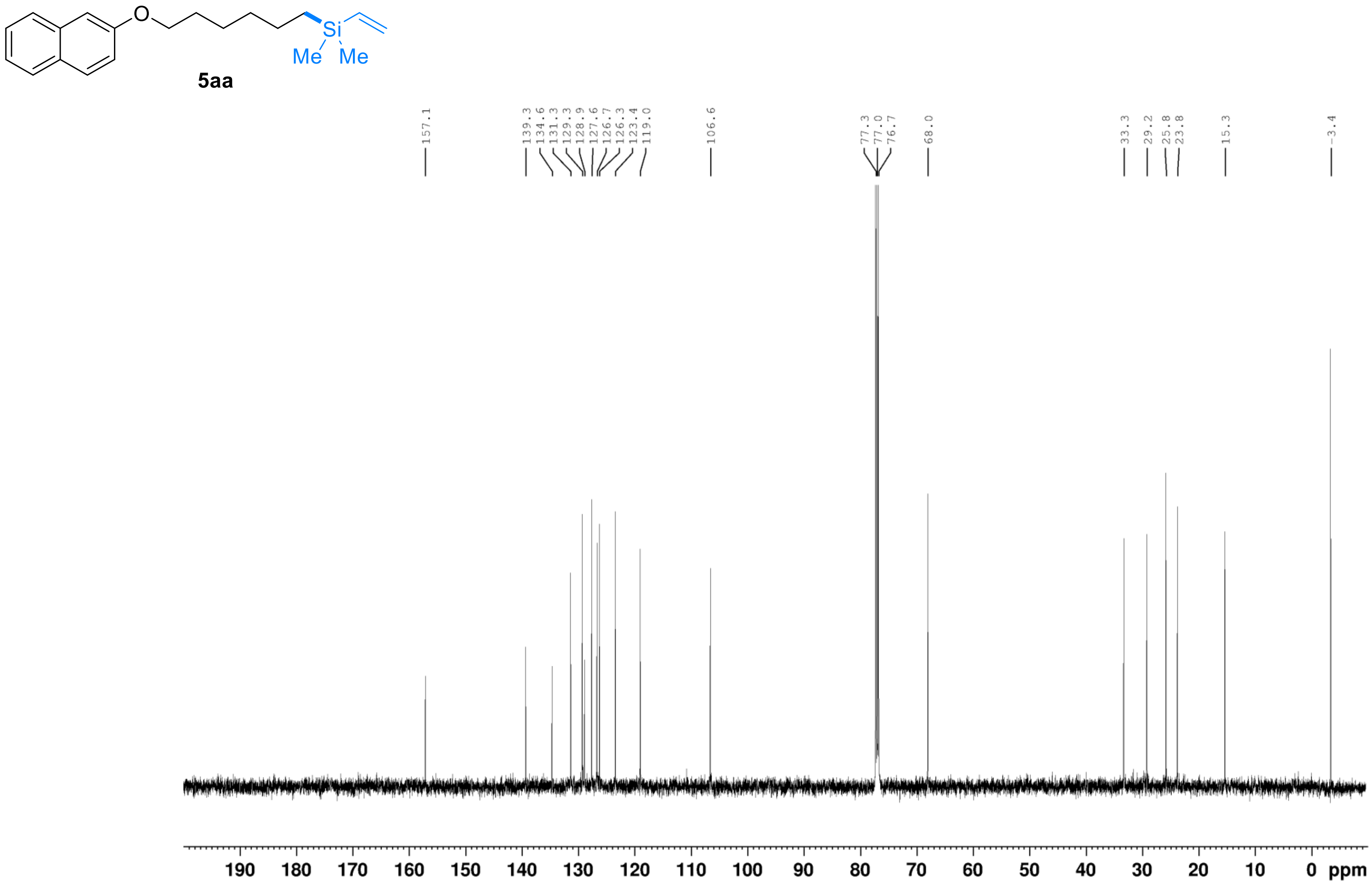

Figure S166. $^{1}H/^{29}Si$ HMQC NMR (500/99 MHz, $CDCl_3$, 298 K, optimized for J = 7 Hz) of **5aa**.

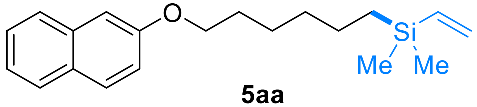

5aa

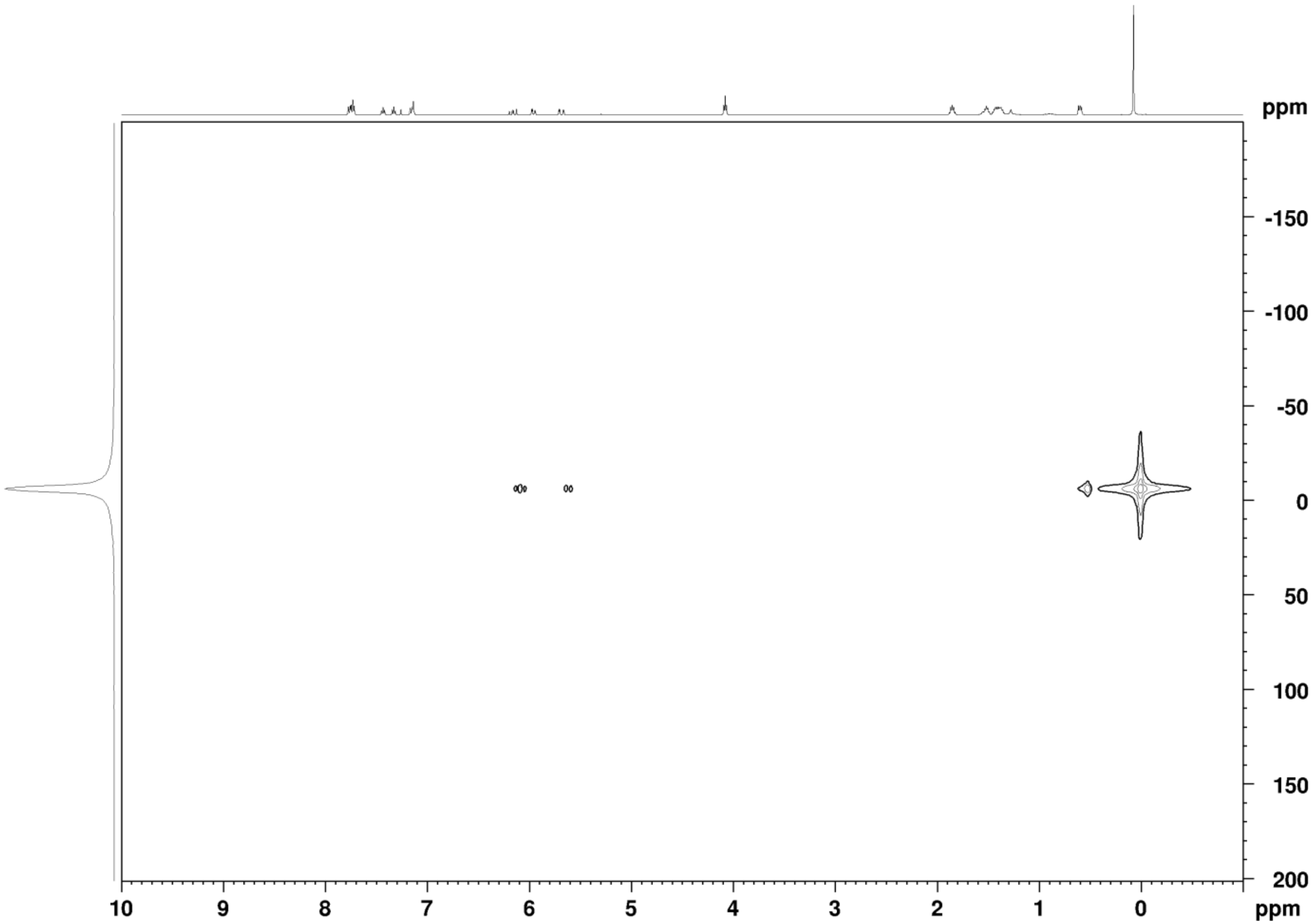

Figure S167. ^{1}H NMR (500 MHz, $CDCl_3$, 298 K) of **5ba**.

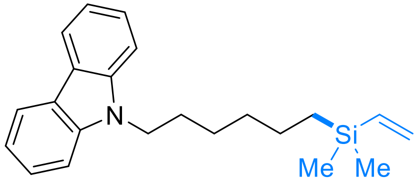

5ba

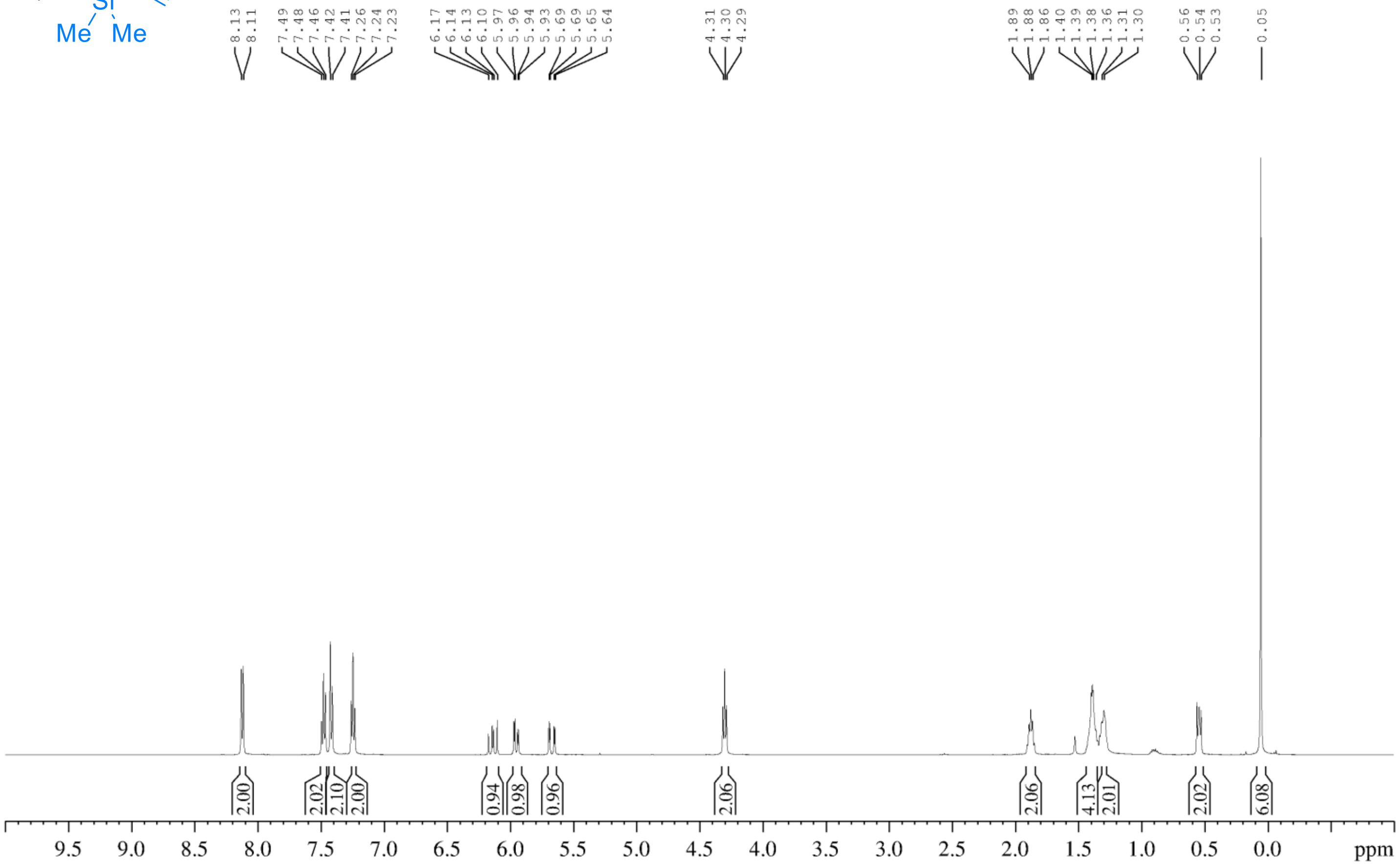

Figure S168. ^{13}C NMR (126 MHz, $CDCl_3$, 298 K) of **5ba**.

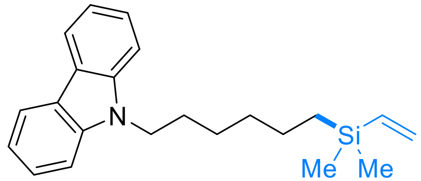

5ba

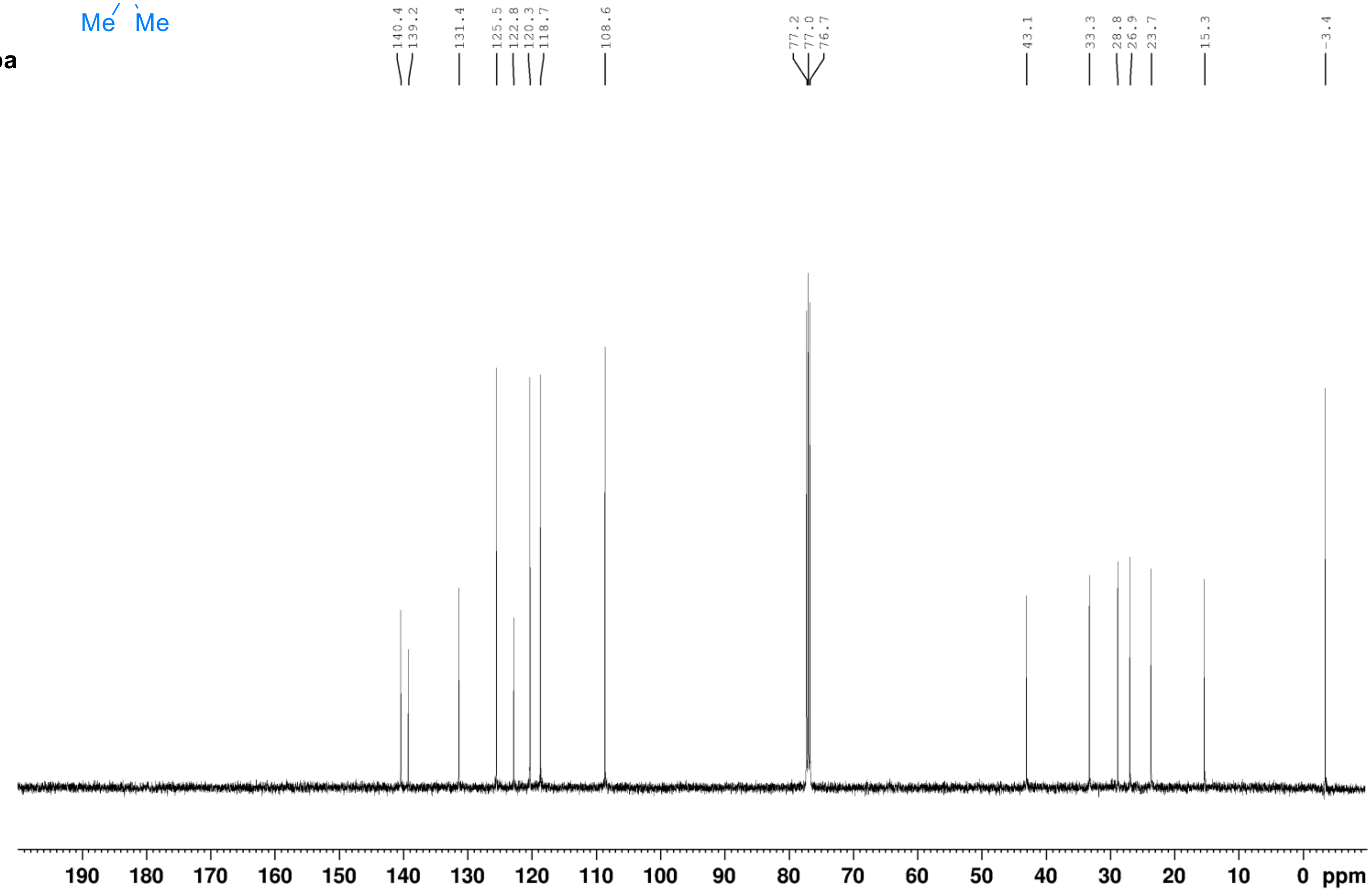

Figure S169. $^{1}H/^{29}Si$ HMQC NMR (500/99 MHz, $CDCl_3$, 298 K, optimized for J = 7 Hz) of **5ba**.

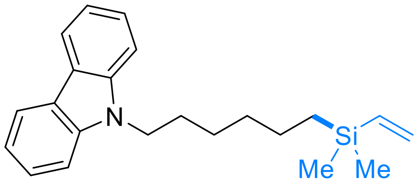

5ba

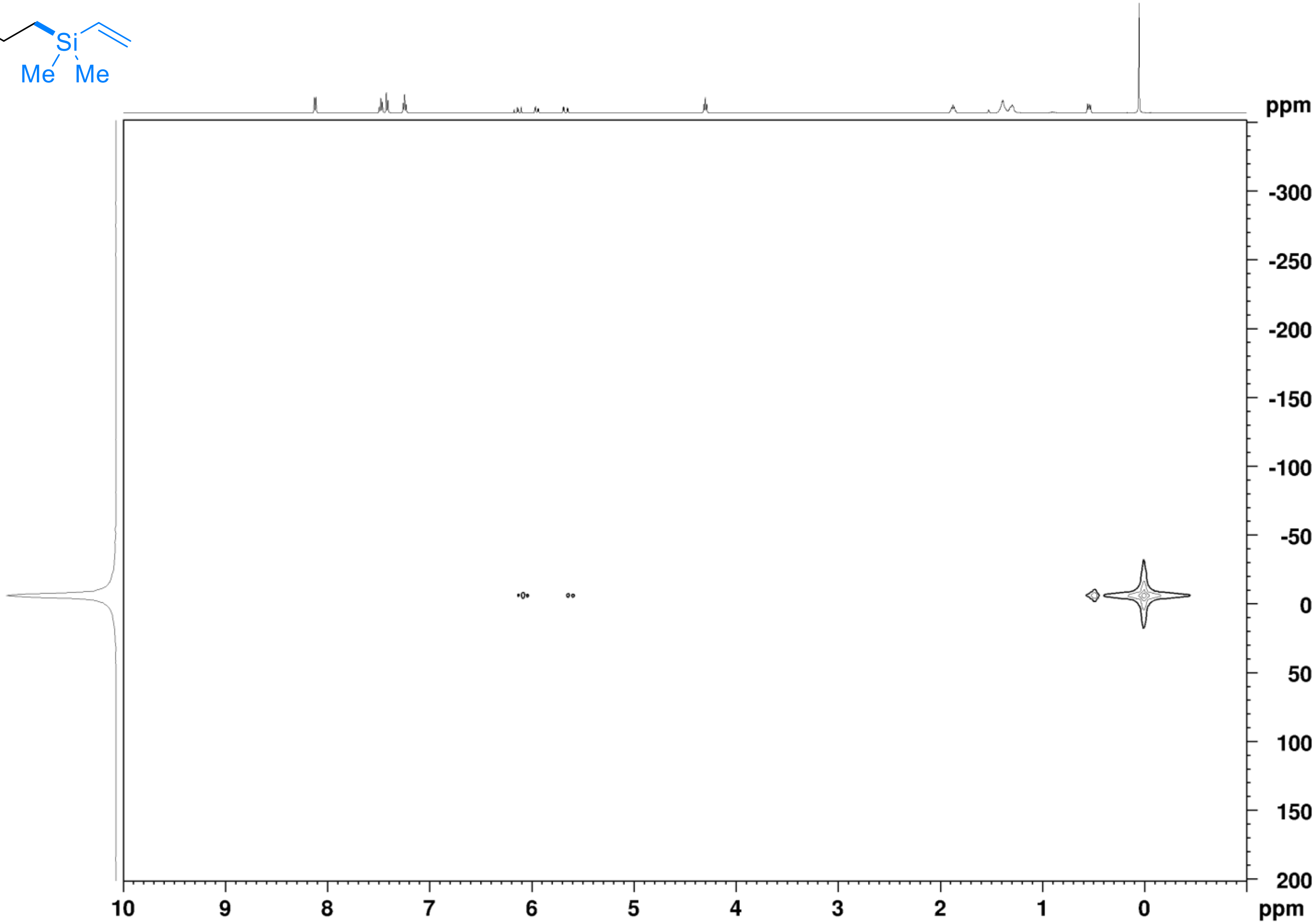

Figure S170. ^{1}H NMR (500 MHz, $CDCl_3$, 298 K) of **5ca**.

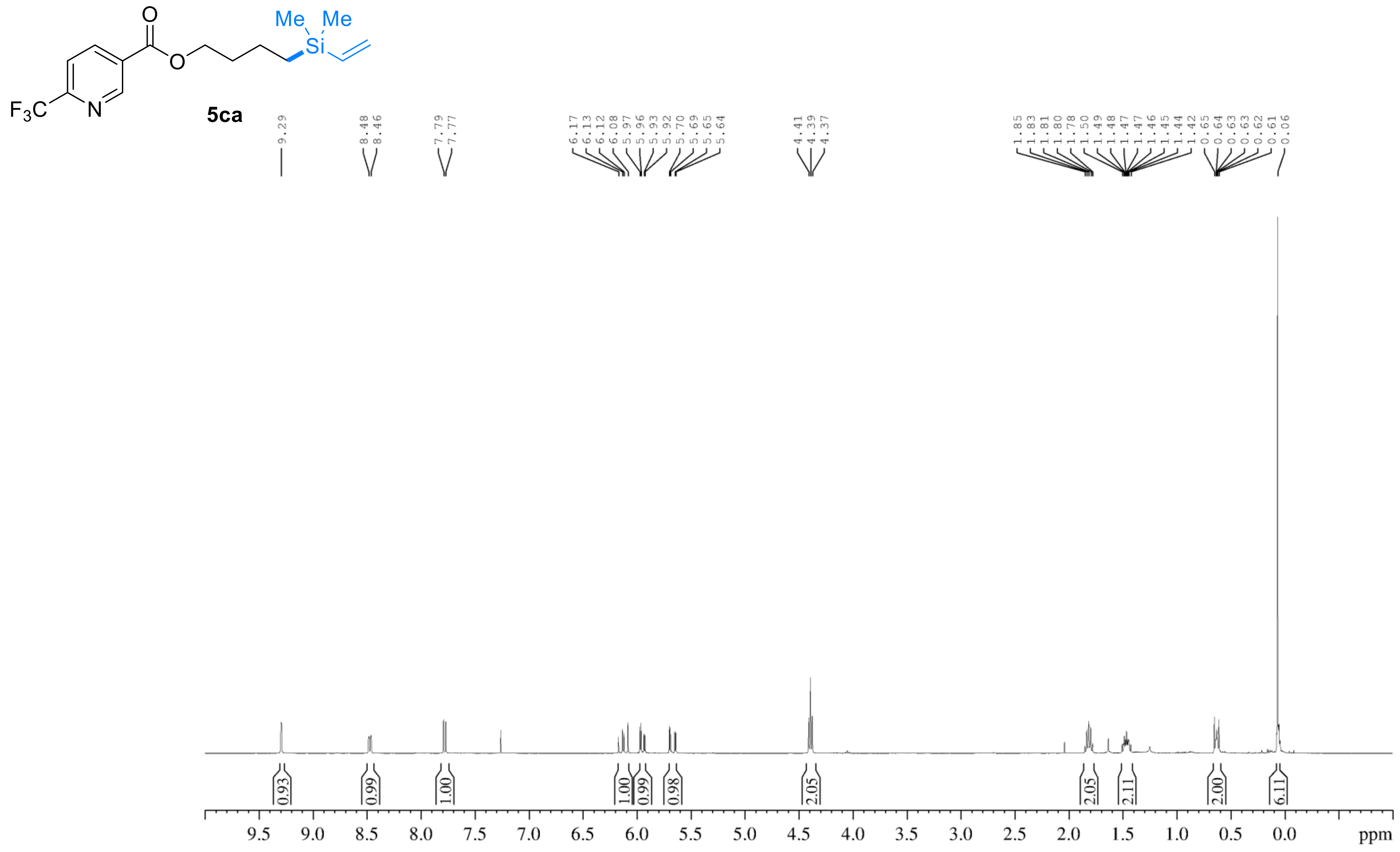

Figure S171. ^{13}C NMR (126 MHz, $CDCl_3$, 298 K) of **5ca**.

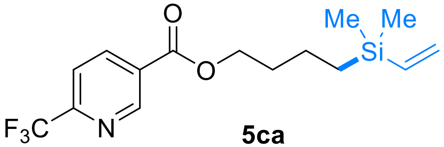

5ca

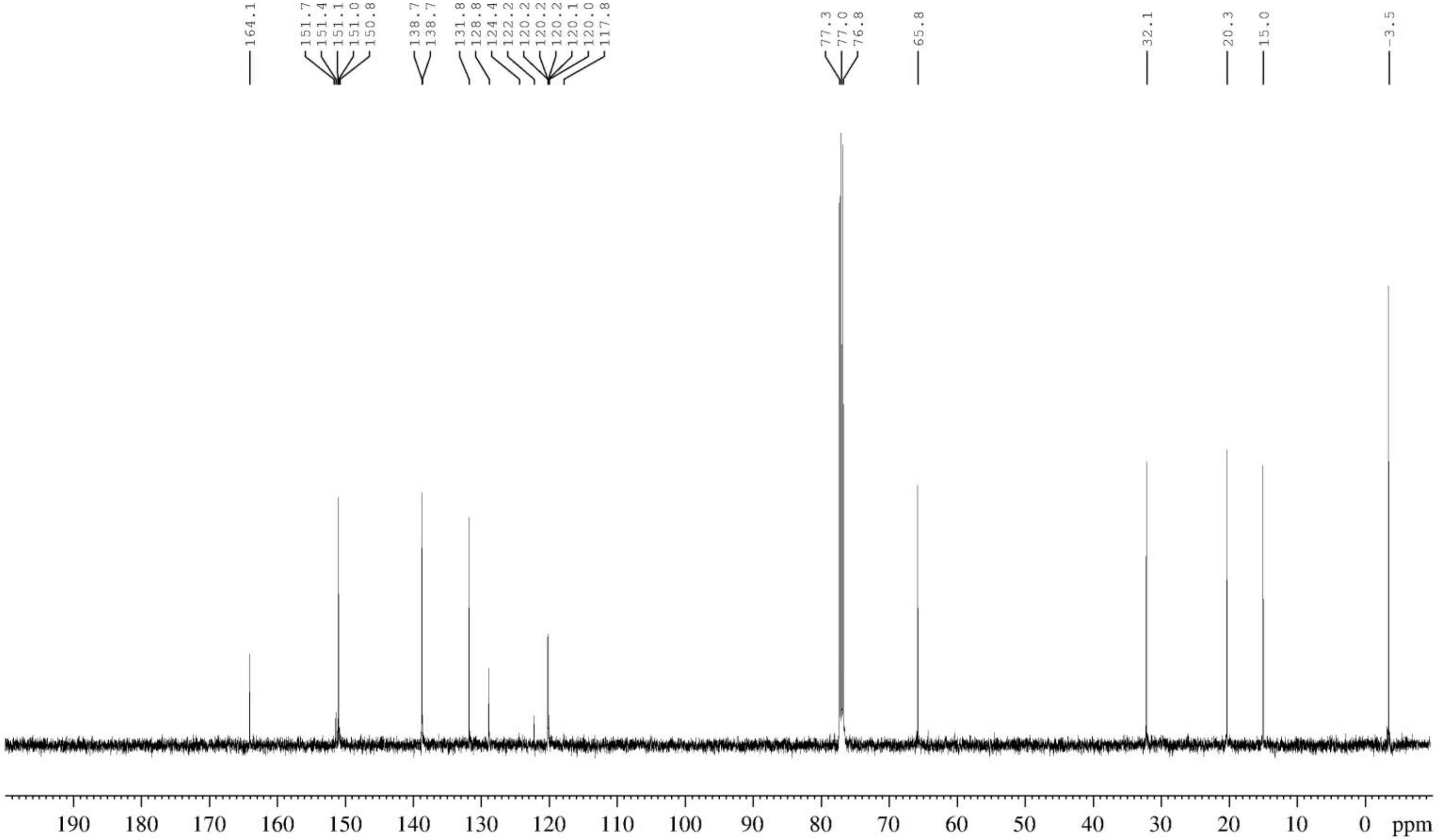

Figure S172. ^{19}F NMR (471 MHz, $CDCl_3$, 298 K) of **5ca**.

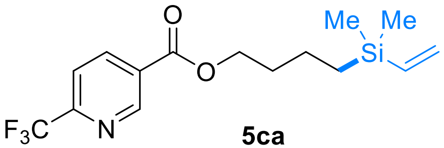

5ca

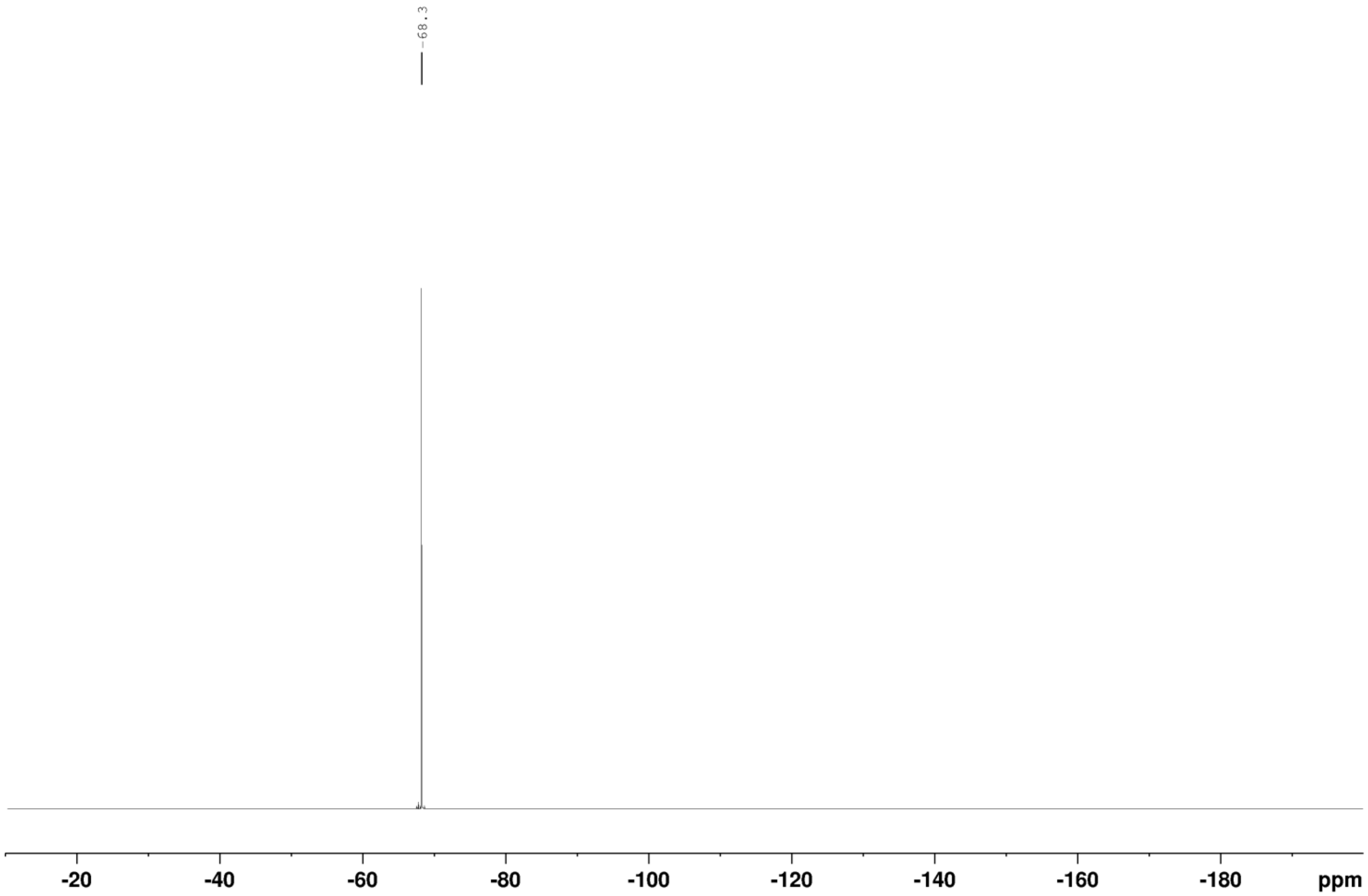

Figure S173. $^{1}H/^{29}Si$ HMQC NMR (500/99 MHz, $CDCl_3$, 298 K, optimized for J = 7 Hz) of **5ca**.

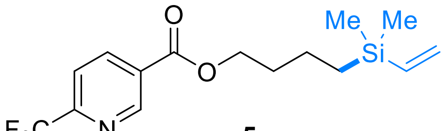

5ca

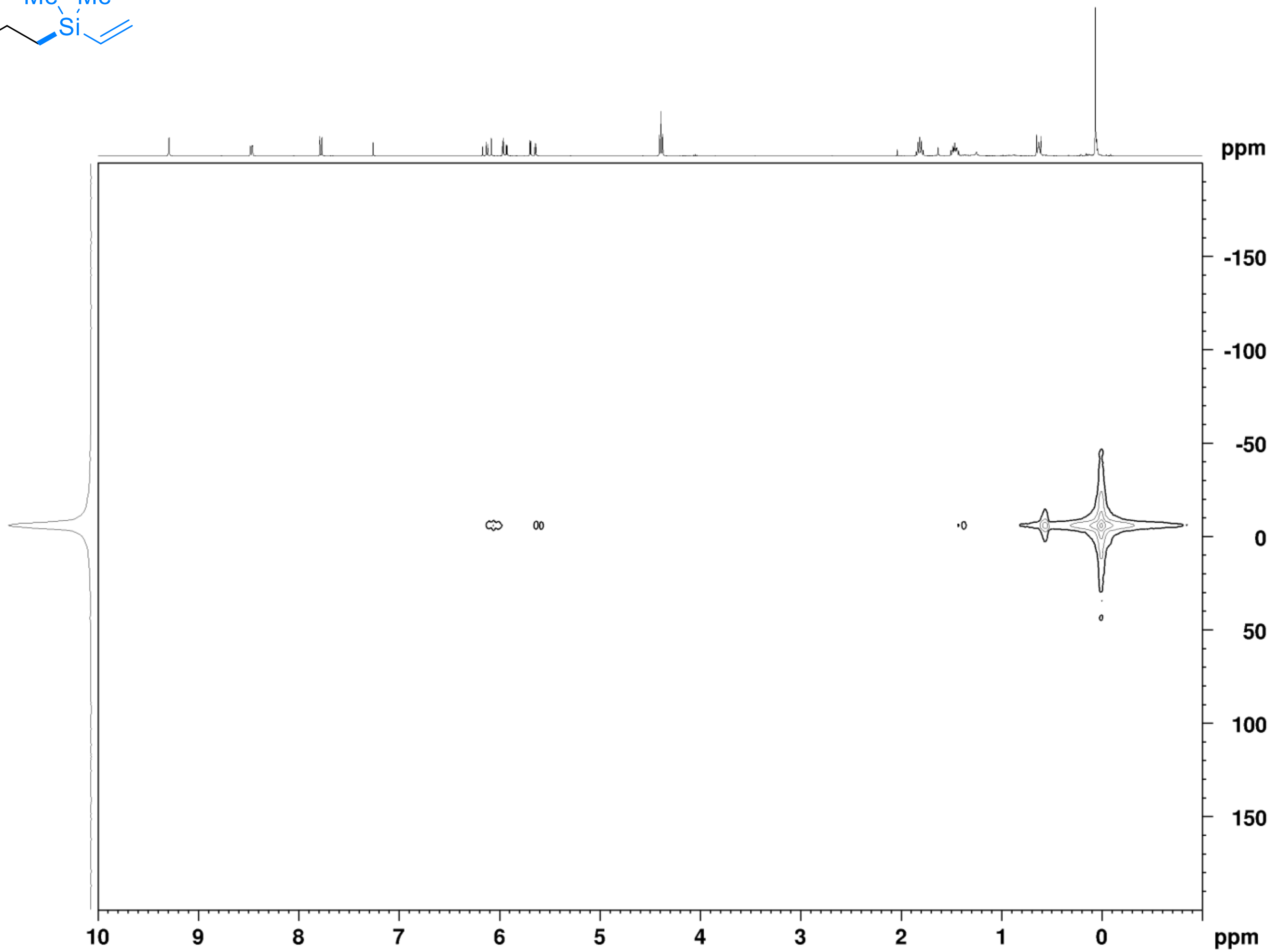

Figure S174. ^{1}H NMR (500 MHz, $CDCl_3$, 298 K) of **5da**.

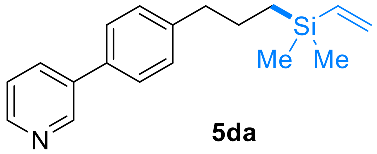

5da

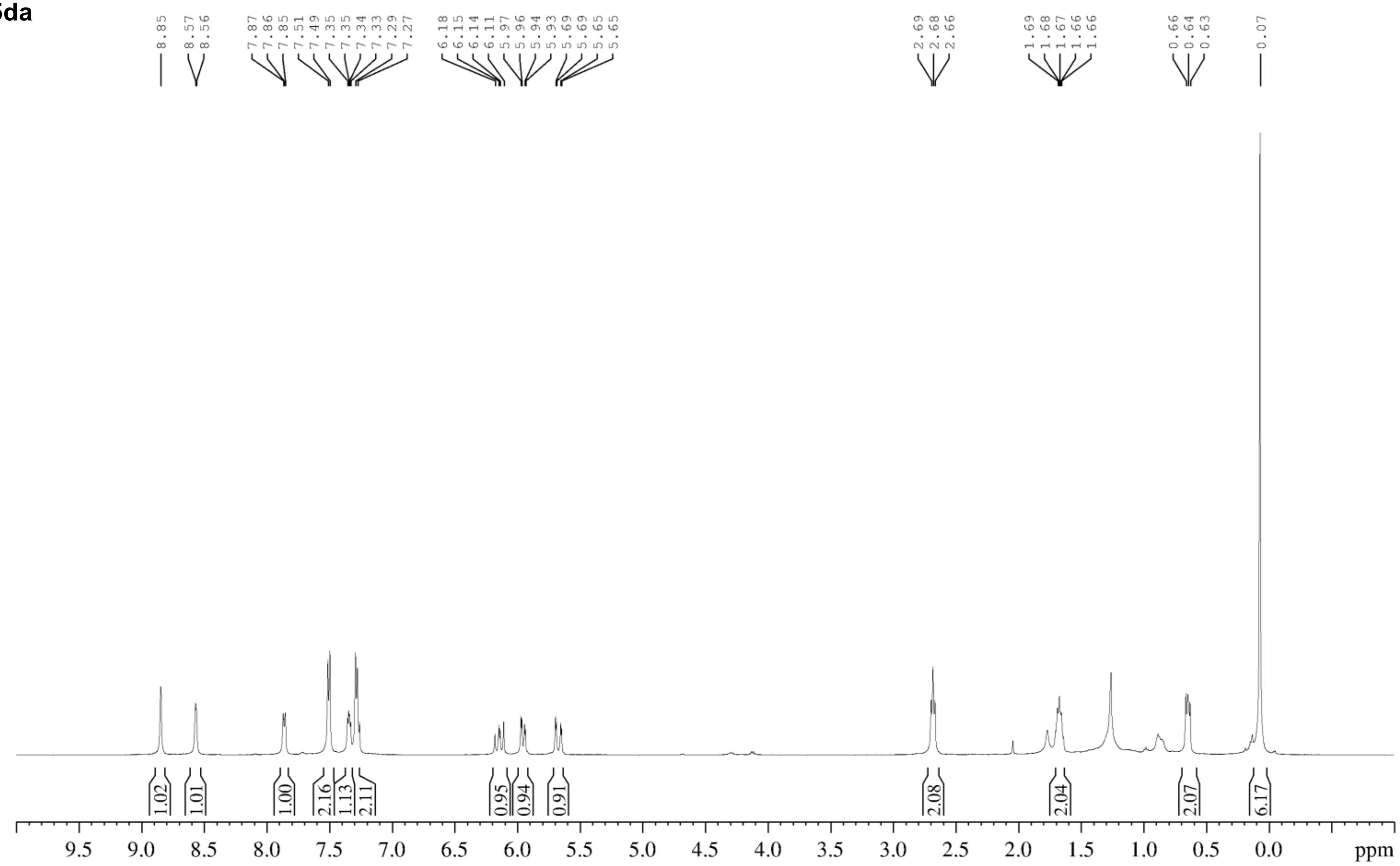

Figure S175. ^{13}C NMR (126 MHz, $CDCl_3$, 298 K) of **5da**.

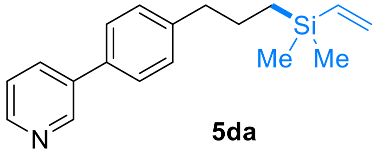

5da

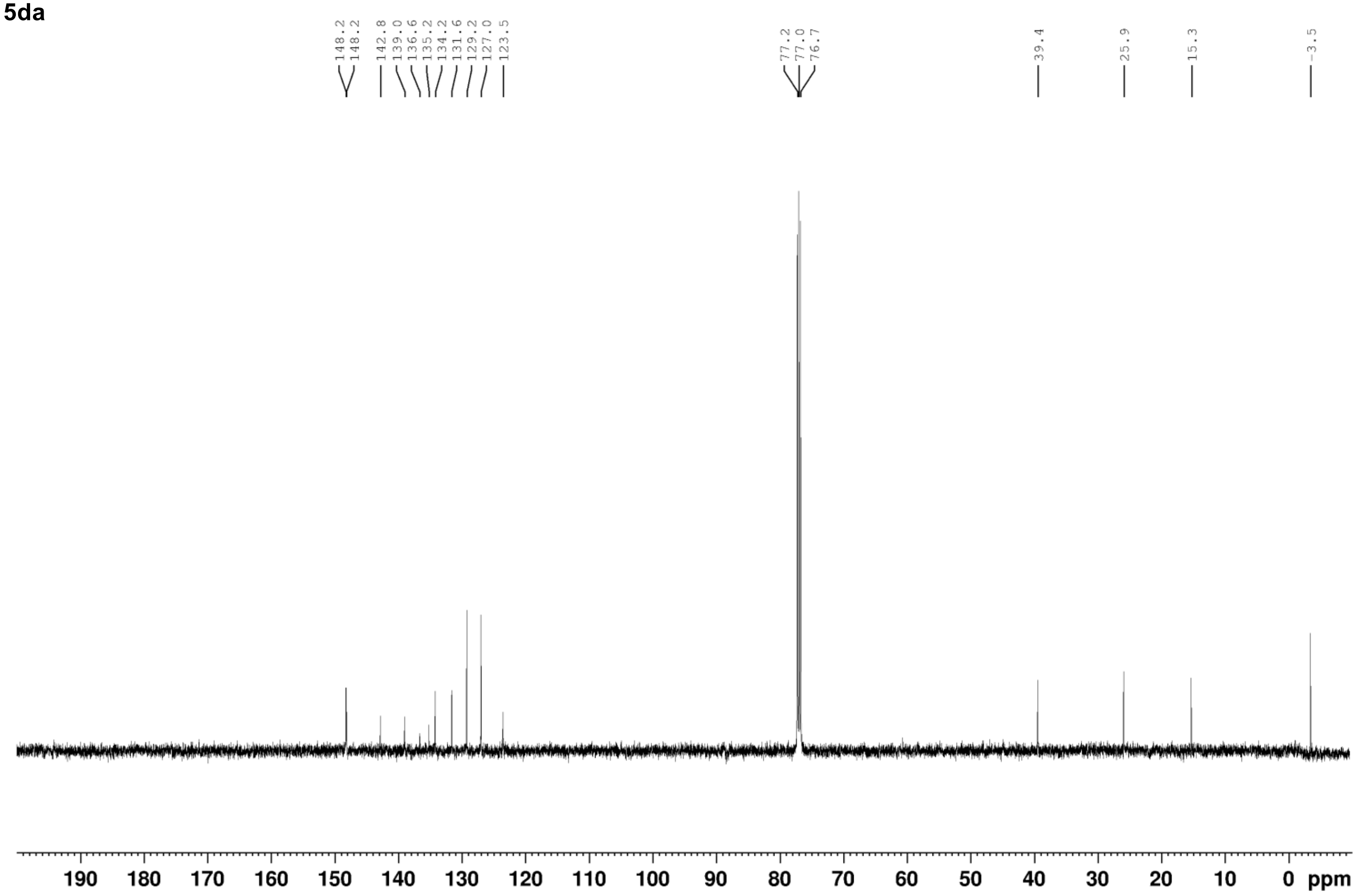

Figure S176. ^{1}H/^{29}Si HMQC NMR (500/99 MHz, $CDCl_3$, 298 K, optimized for J = 7 Hz) of **5da**.

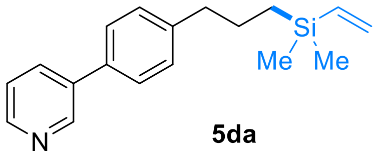

5da

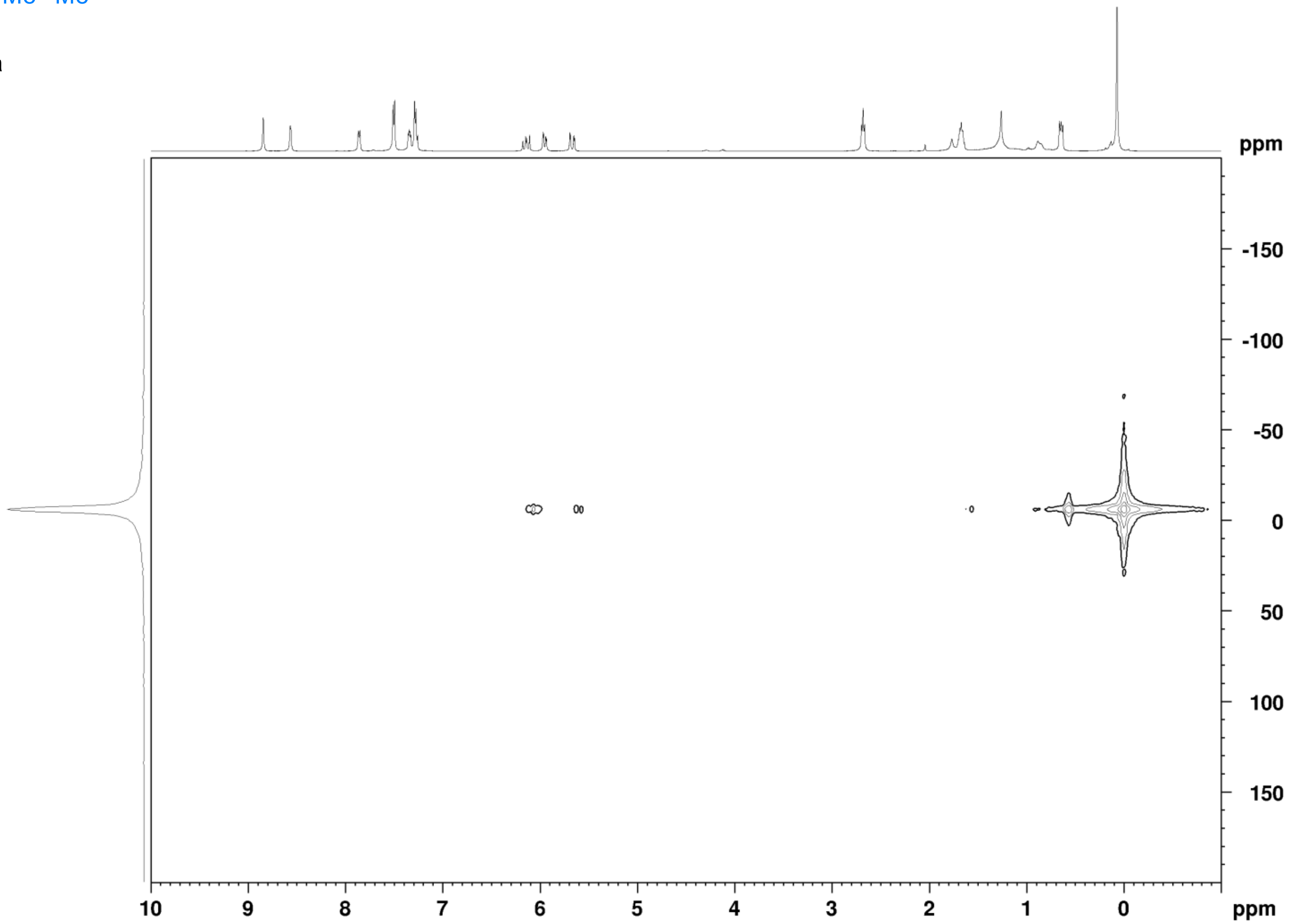

Figure S177. ^{1}H NMR (500 MHz, $CDCl_3$, 298 K) of **5ea**.

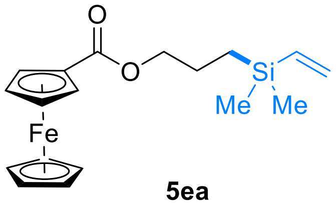

5ea

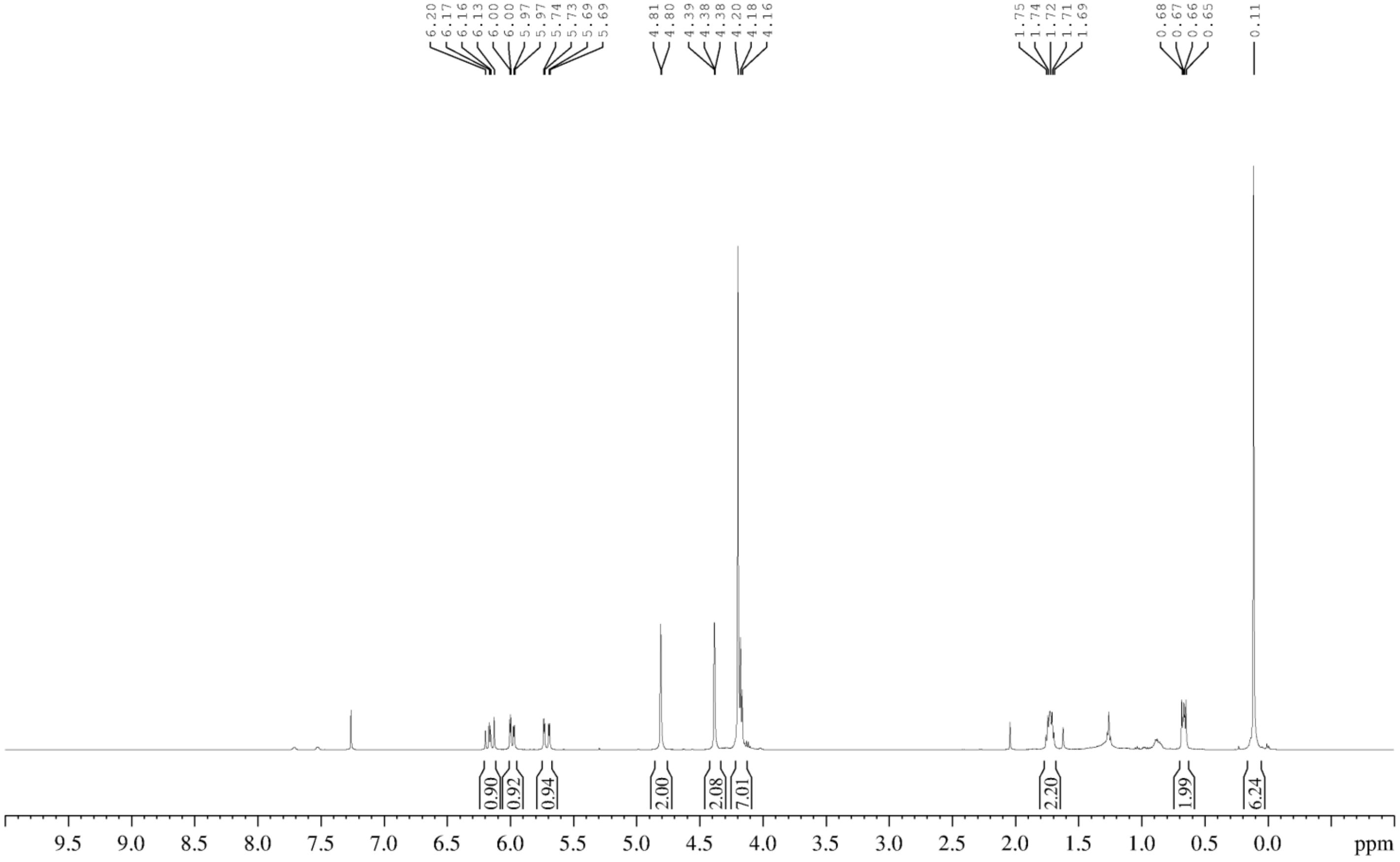

Figure S178. ^{13}C NMR (126 MHz, $CDCl_3$, 298 K) of **5ea**.

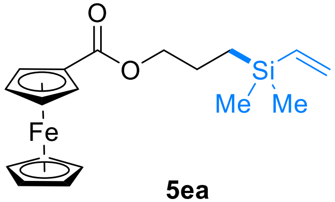

5ea

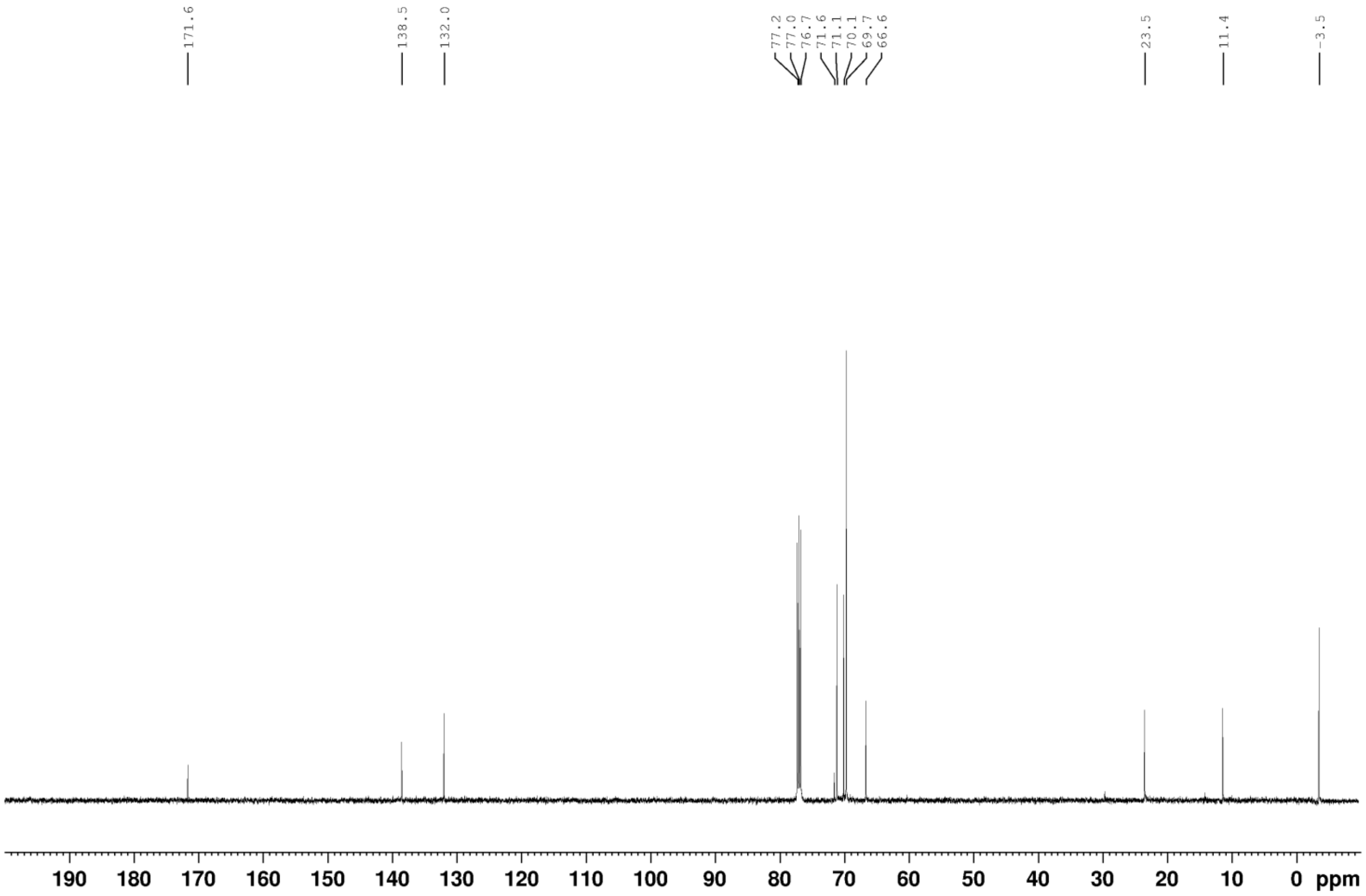

Figure S179. $^{1}H/^{29}Si$ HMQC NMR (500/99 MHz, $CDCl_3$, 298 K, optimized for J = 7 Hz) of **5ea**.

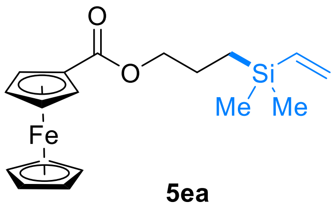

5ea

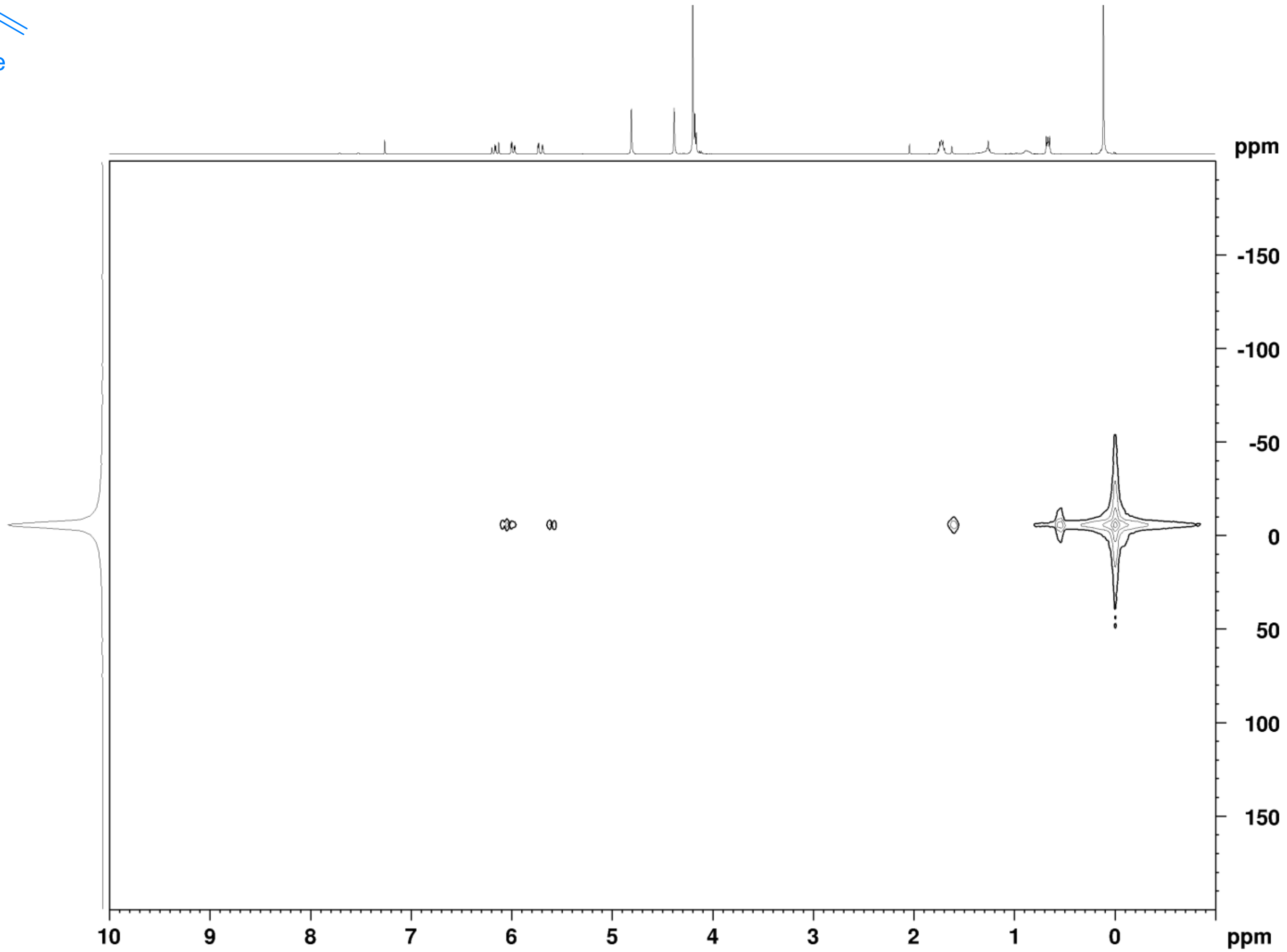

Figure S180. ^{1}H NMR (500 MHz, $CDCl_3$, 298 K) of **3ab**.

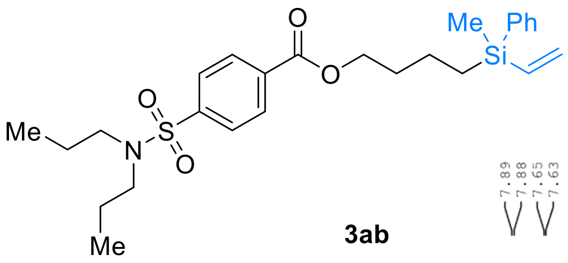

3ab

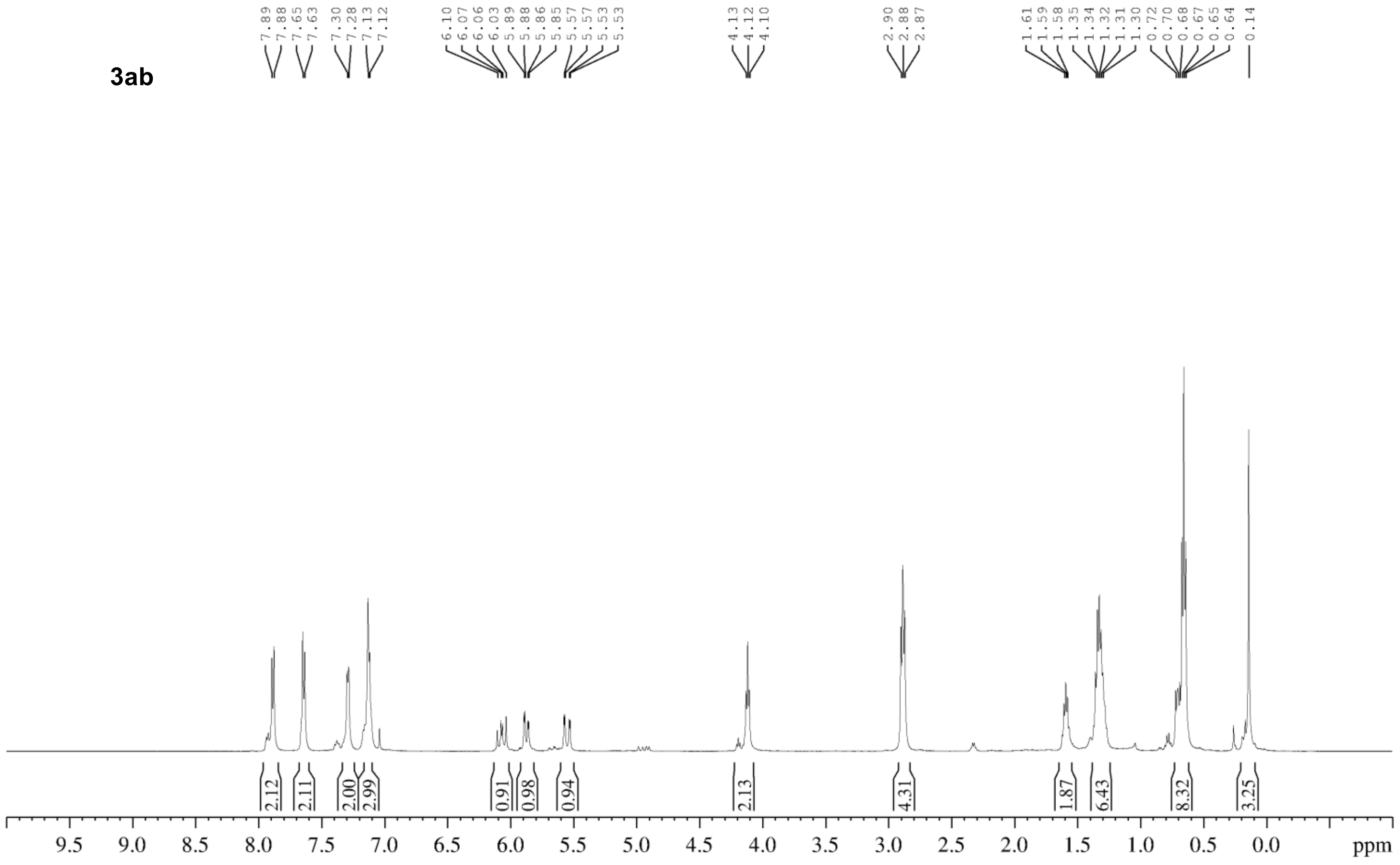

Figure S181. ^{13}C NMR (126 MHz, $CDCl_3$, 298 K) of **3ab**.

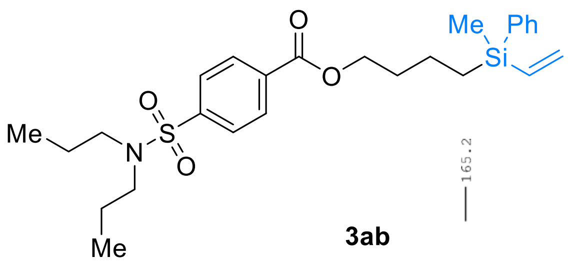

3ab

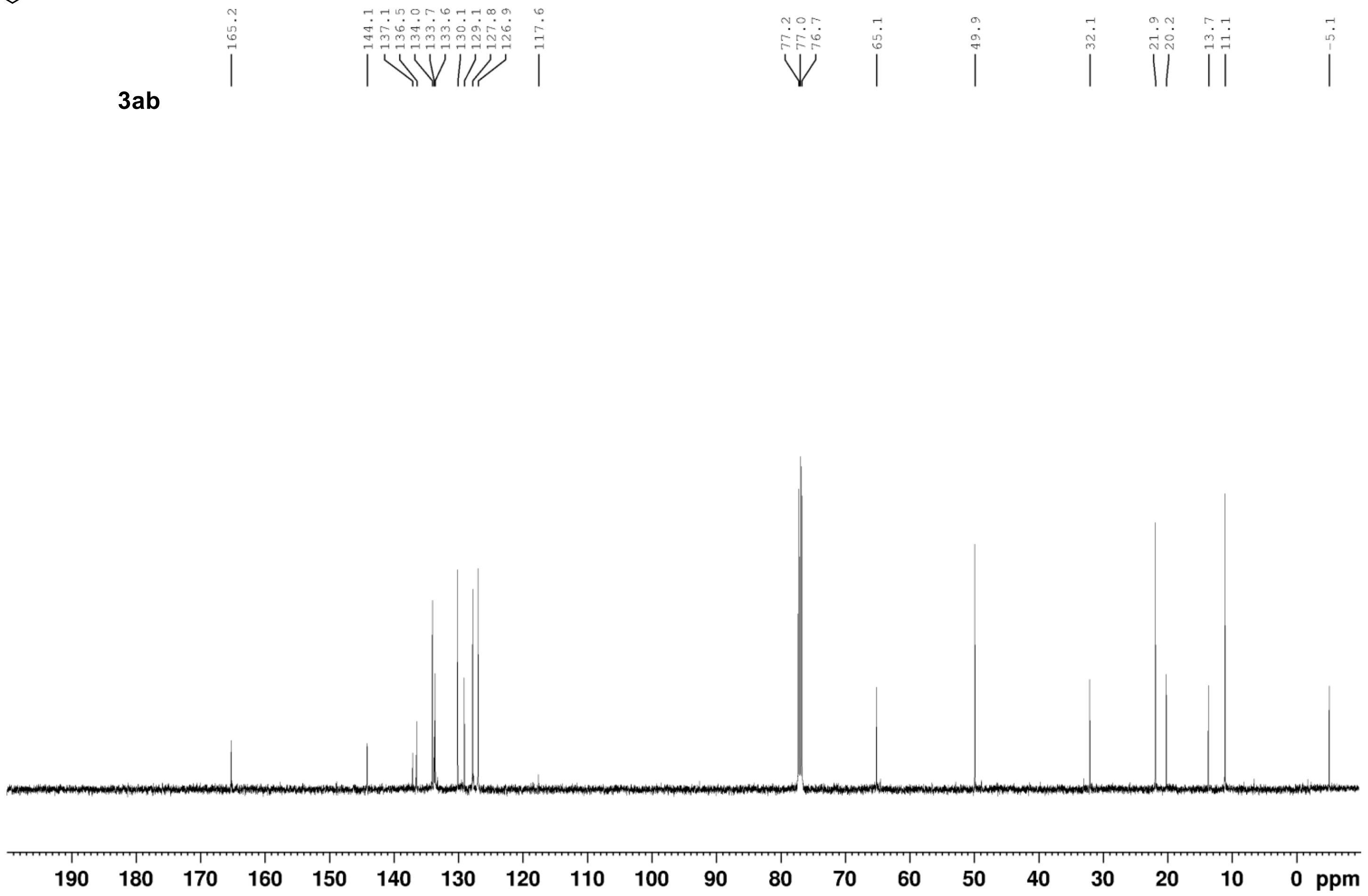

Figure S182. $^{1}H/^{29}Si$ HMQC NMR (500/99 MHz, $CDCl_3$, 298 K, optimized for J = 7 Hz) of **3ab**.

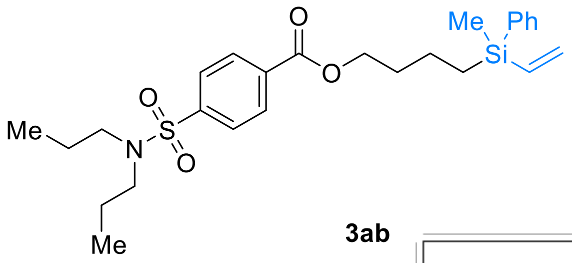

3ab

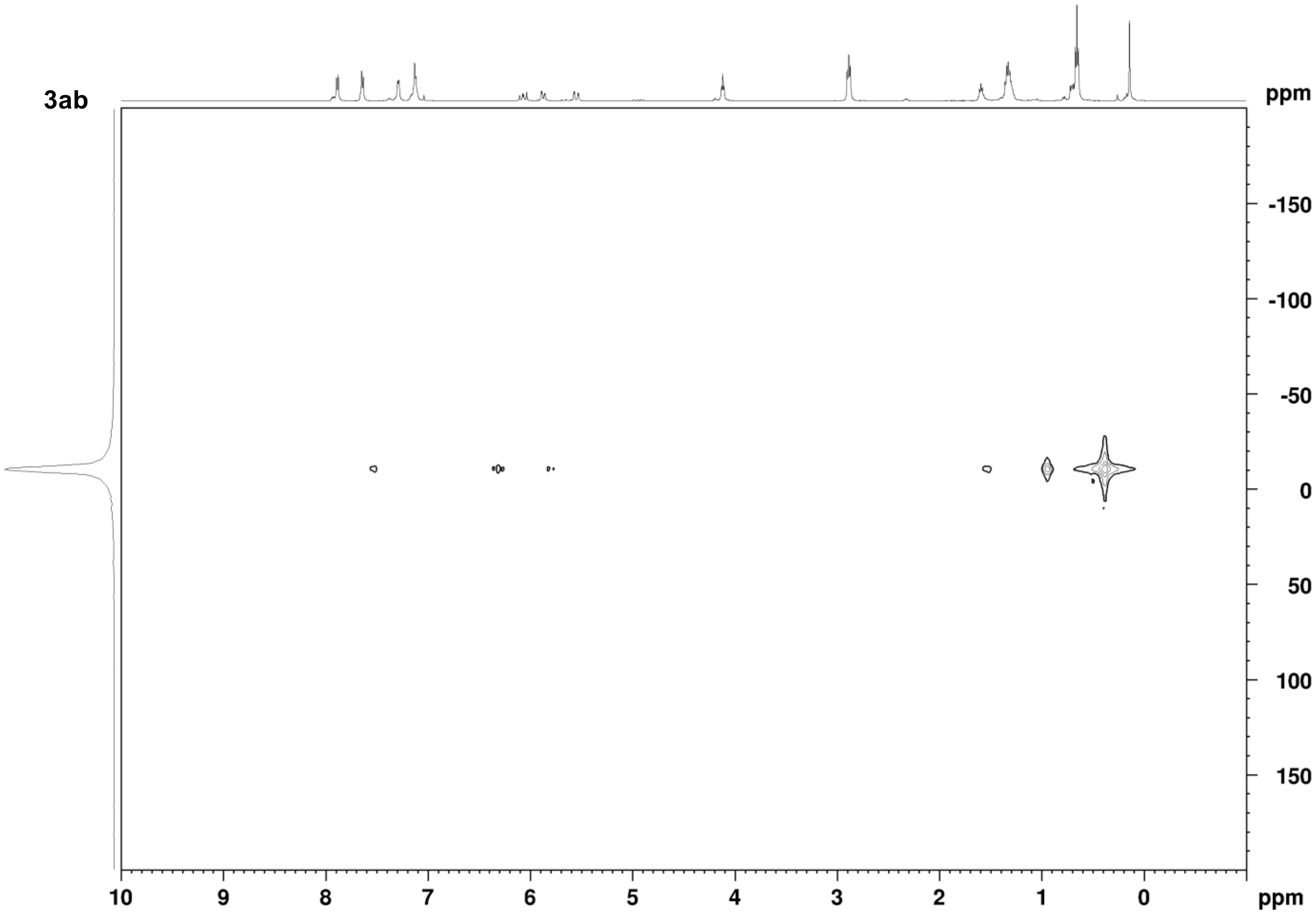

Figure S183. ^{1}H NMR (500 MHz, $CDCl_3$, 298 K) of **3ac**

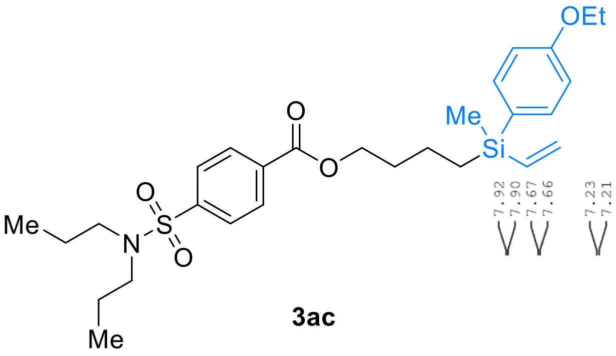

3ac

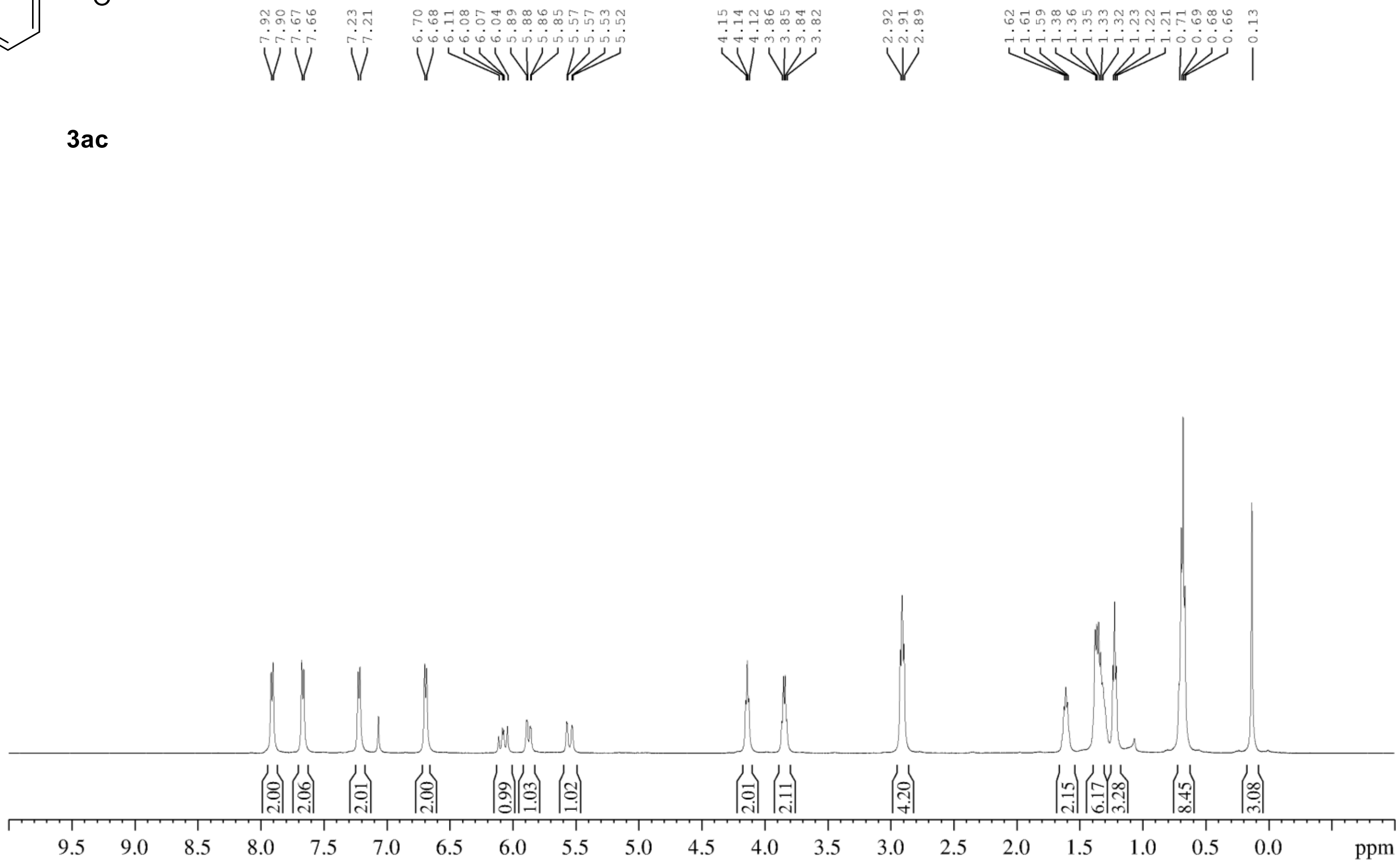

Figure S184. ^{13}C NMR (126 MHz, $CDCl_3$, 298 K) of **3ac**.

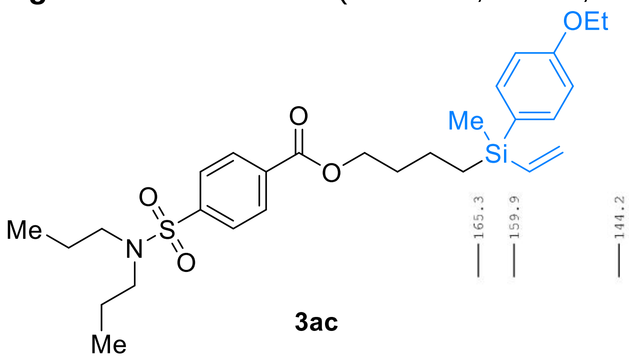

3ac

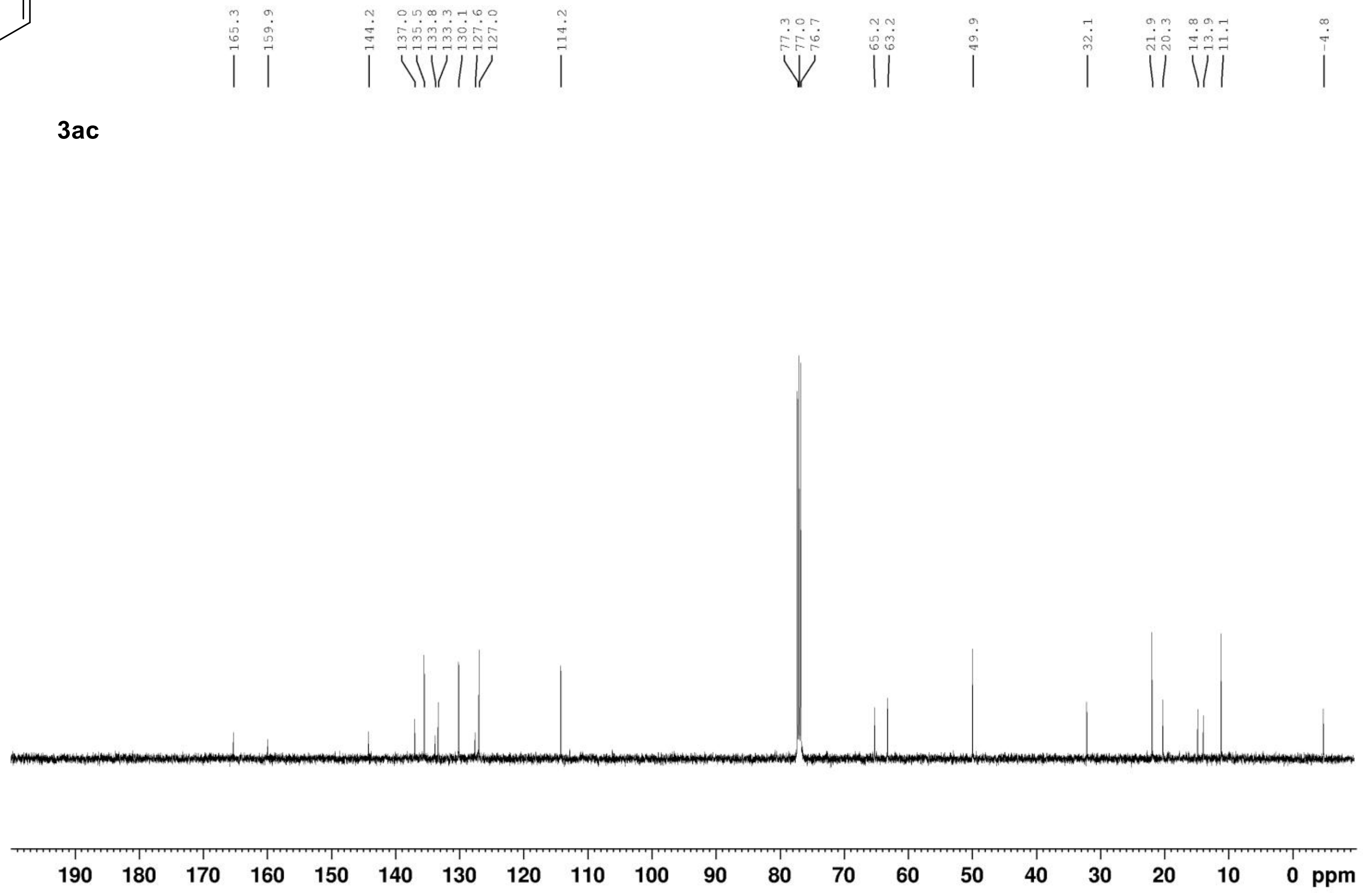

Figure S185. $^{1}H/^{29}Si$ HMQC NMR (500/99 MHz, $CDCl_3$, 298 K, optimized for J = 7 Hz) of **3ac**.

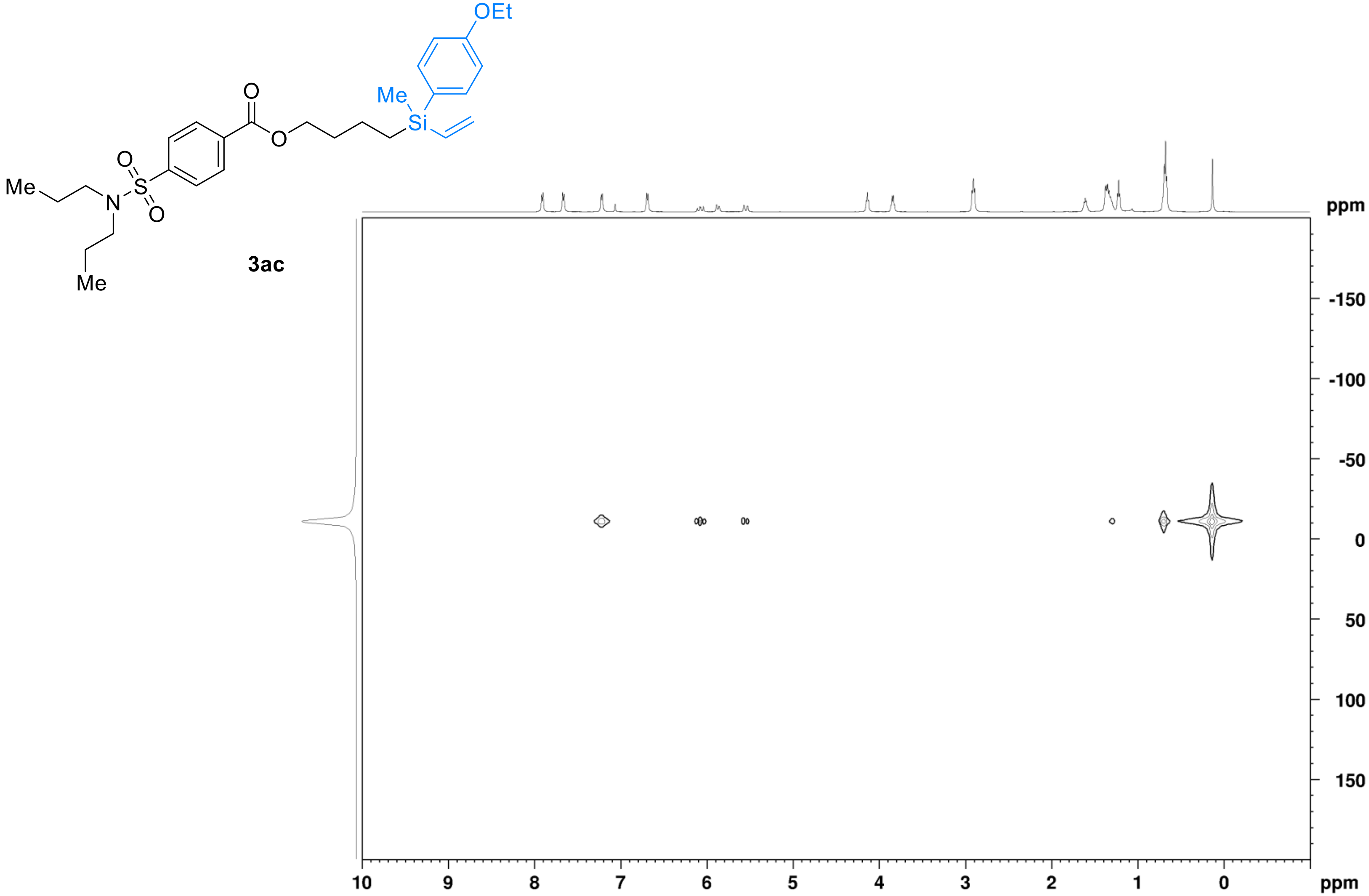

Figure S186. ^{1}H NMR (500 MHz, $CDCl_3$, 298 K) of **3ae**.

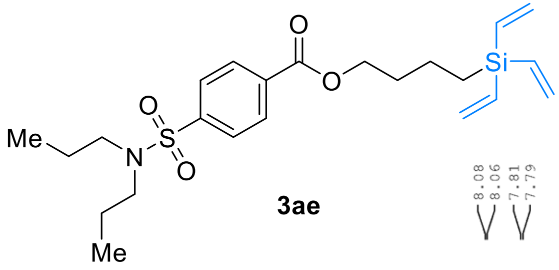

3ae

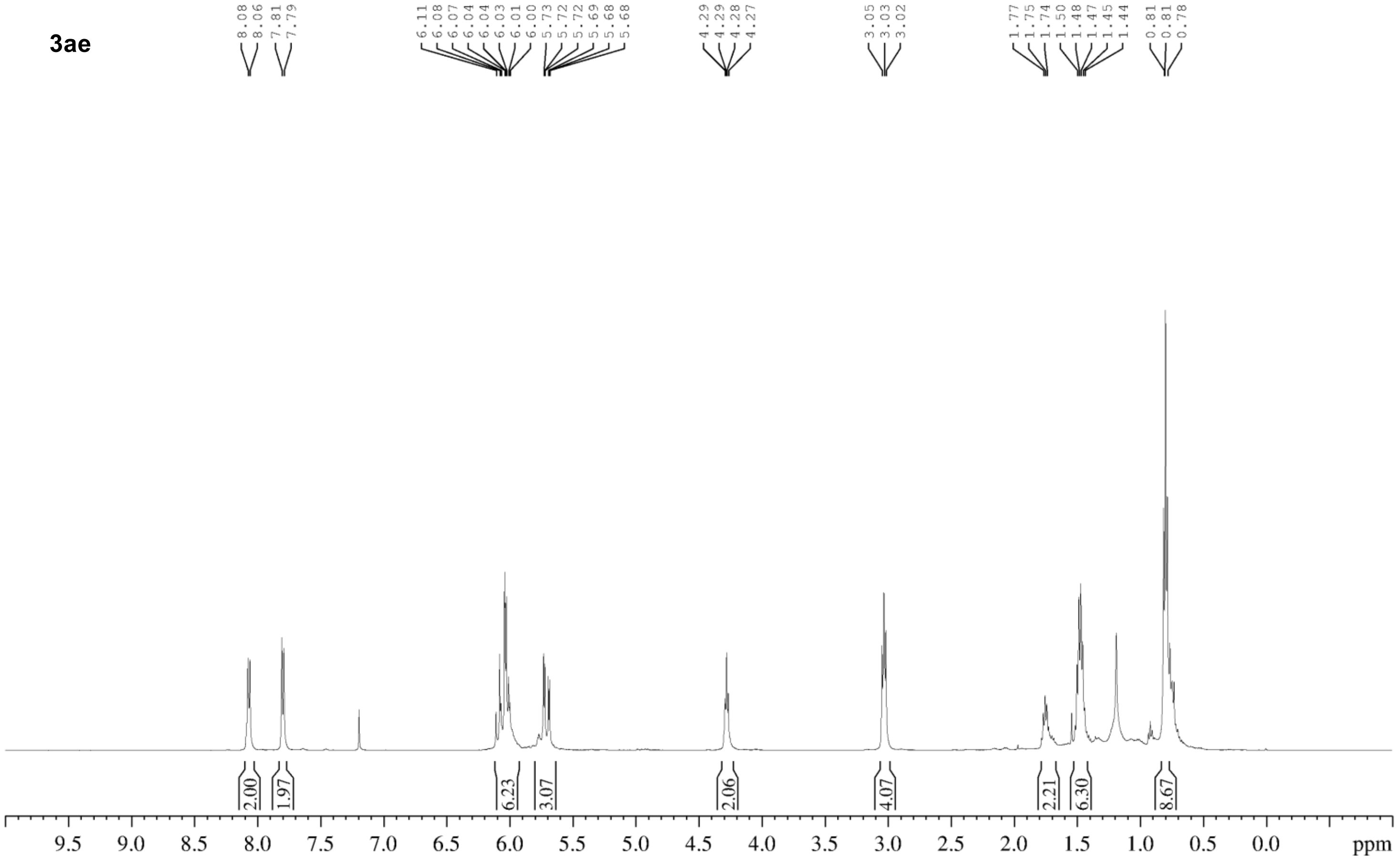

Figure S187. ^{13}C NMR (126 MHz, $CDCl_3$, 298 K) of **3ae**.

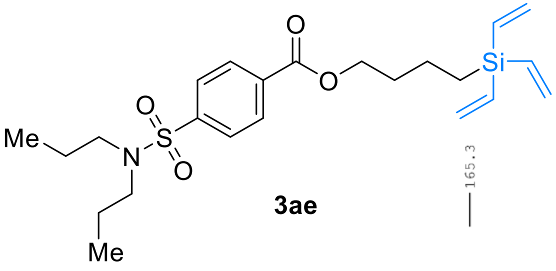

3ae

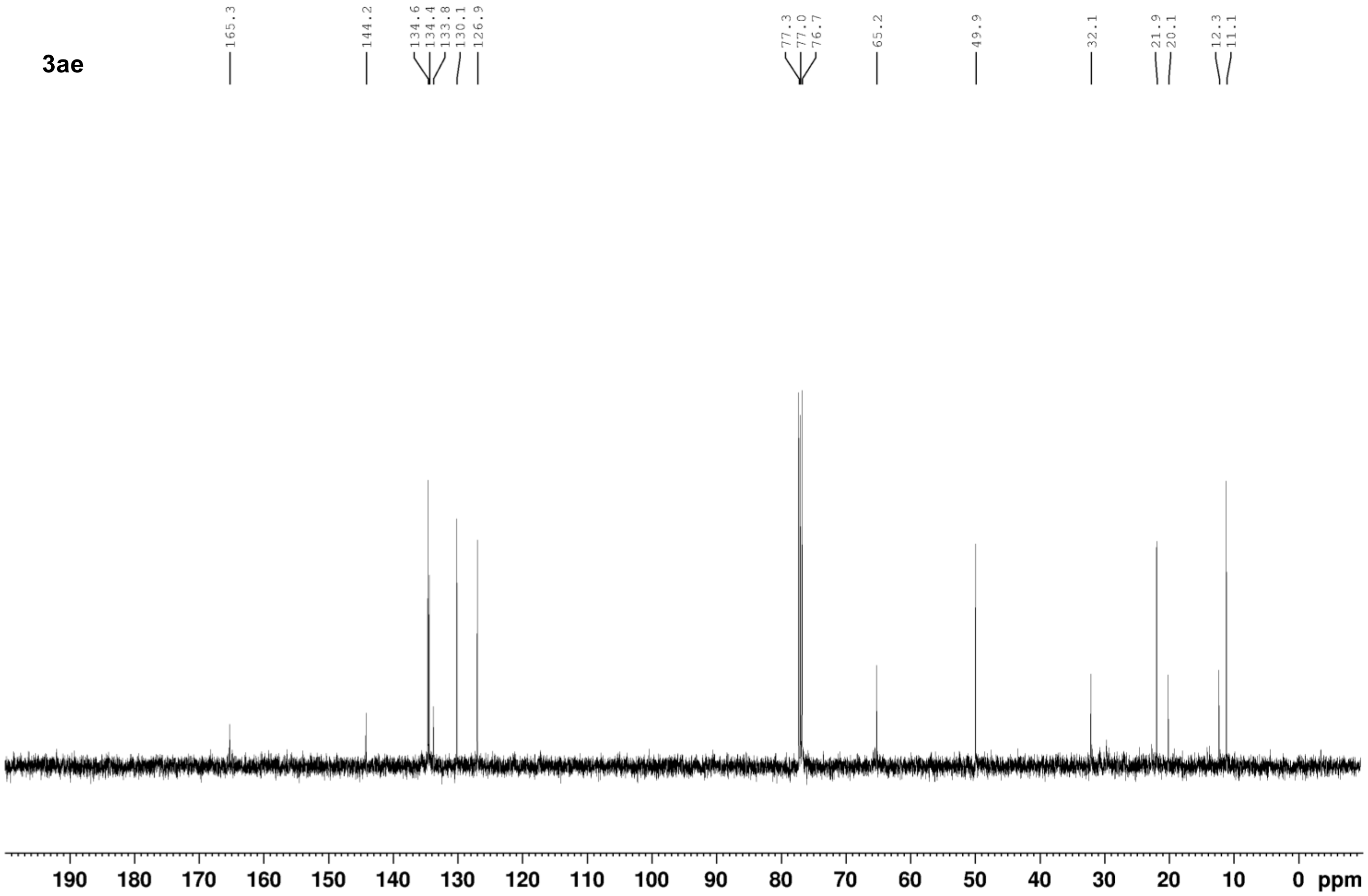

Figure S188. $^{1}H/^{29}Si$ HMQC NMR (500/99 MHz, $CDCl_3$, 298 K, optimized for J = 7 Hz) of **3ae**.

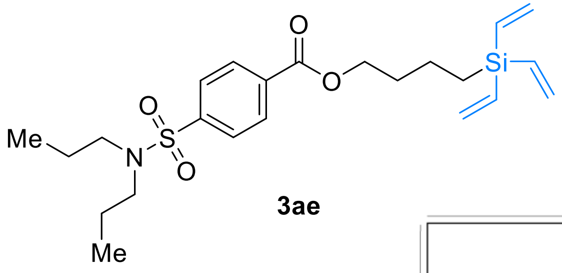

3ae

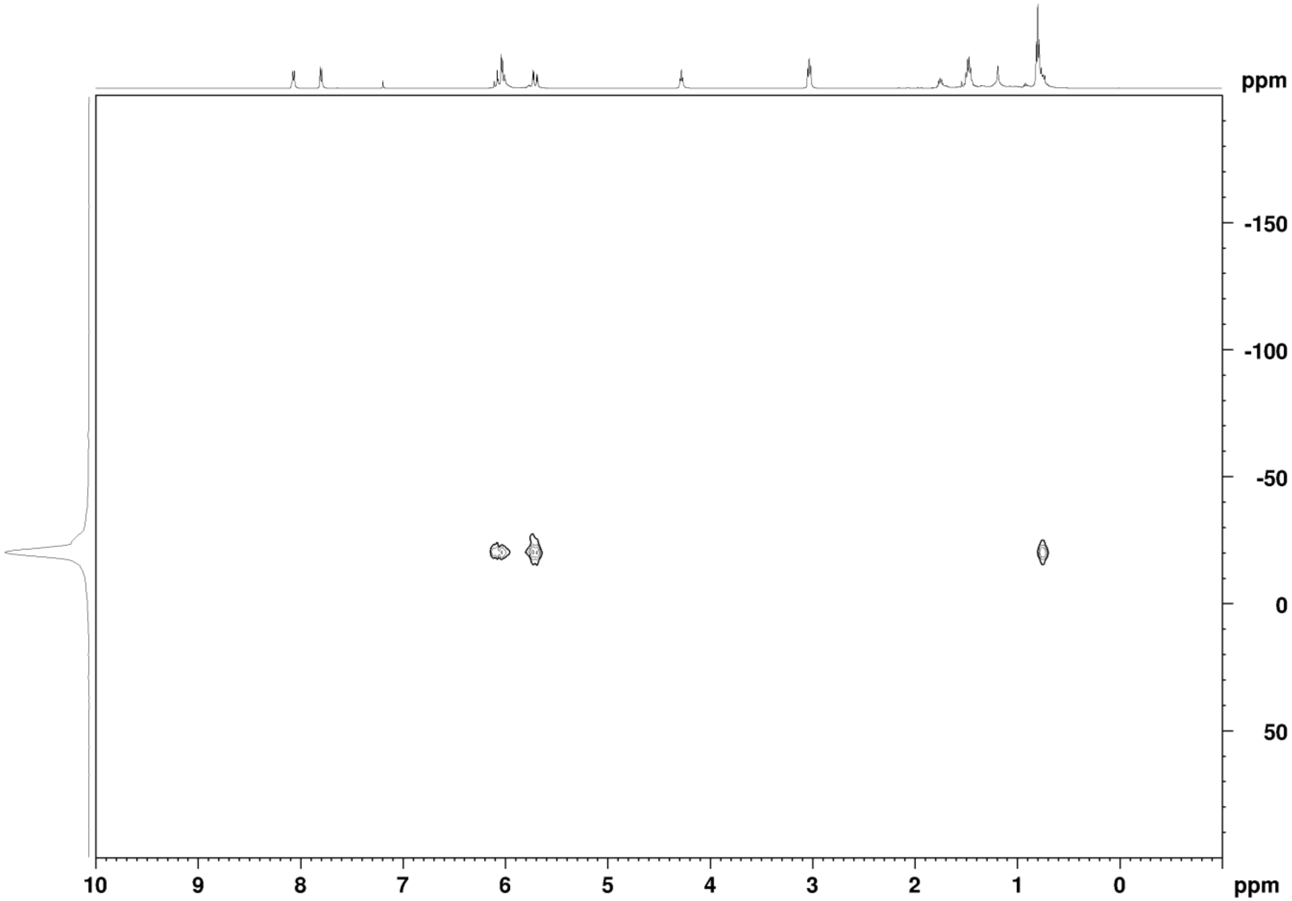

Figure S189. ^{1}H NMR (500 MHz, $CDCl_3$, 298 K) of **7aa**.

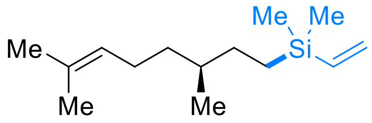

7aa

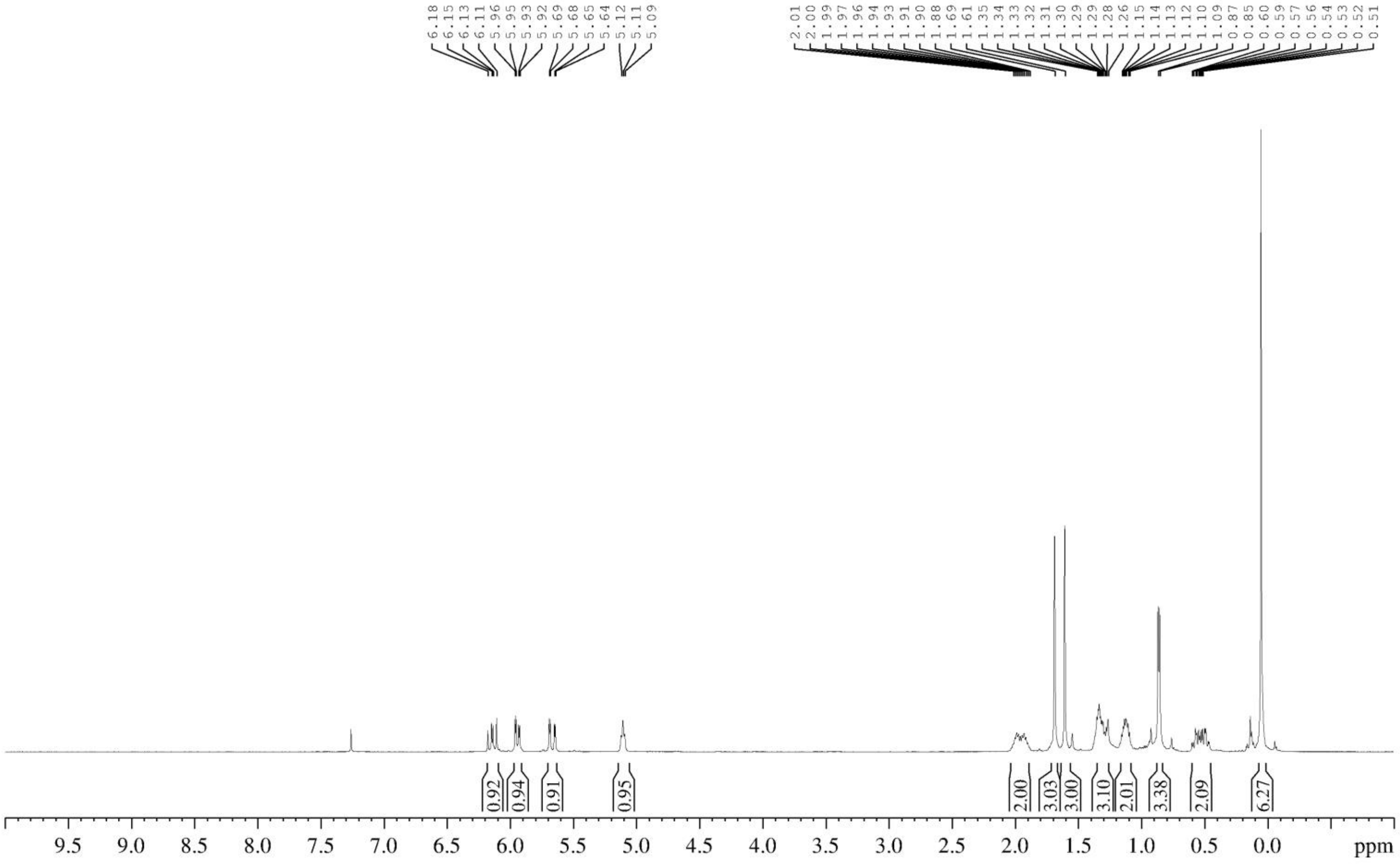

Figure S190. ^{13}C NMR (126 MHz, $CDCl_3$, 298 K) of **7aa**.

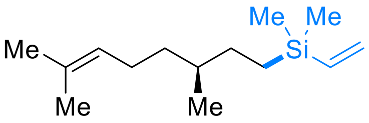

7aa

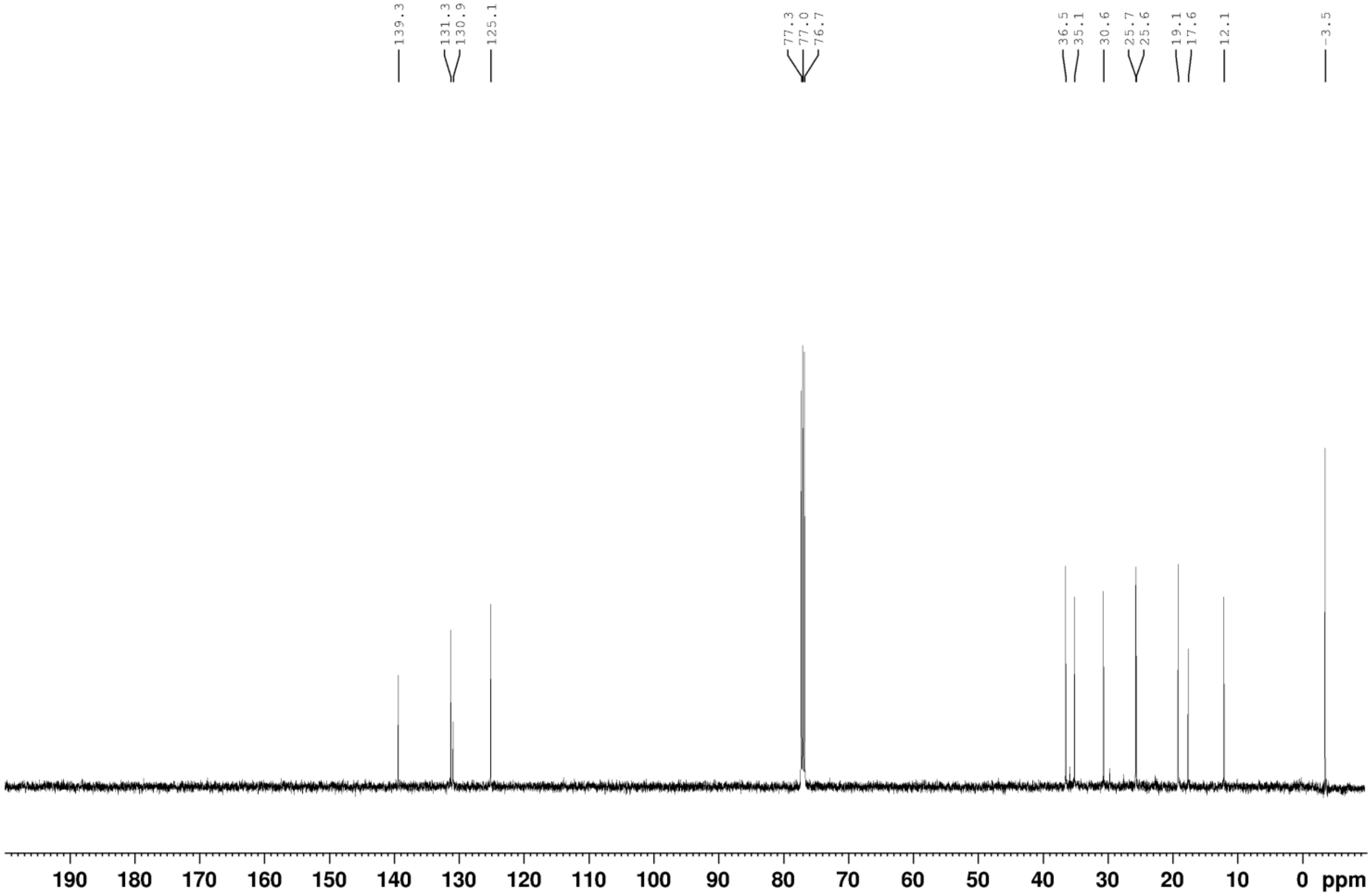

Figure S191. $^{1}H/^{29}Si$ HMQC NMR (500/99 MHz, $CDCl_3$, 298 K, optimized for J = 7 Hz) of **7aa**.

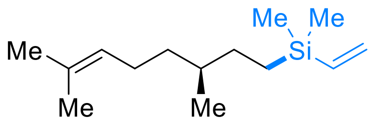

7aa

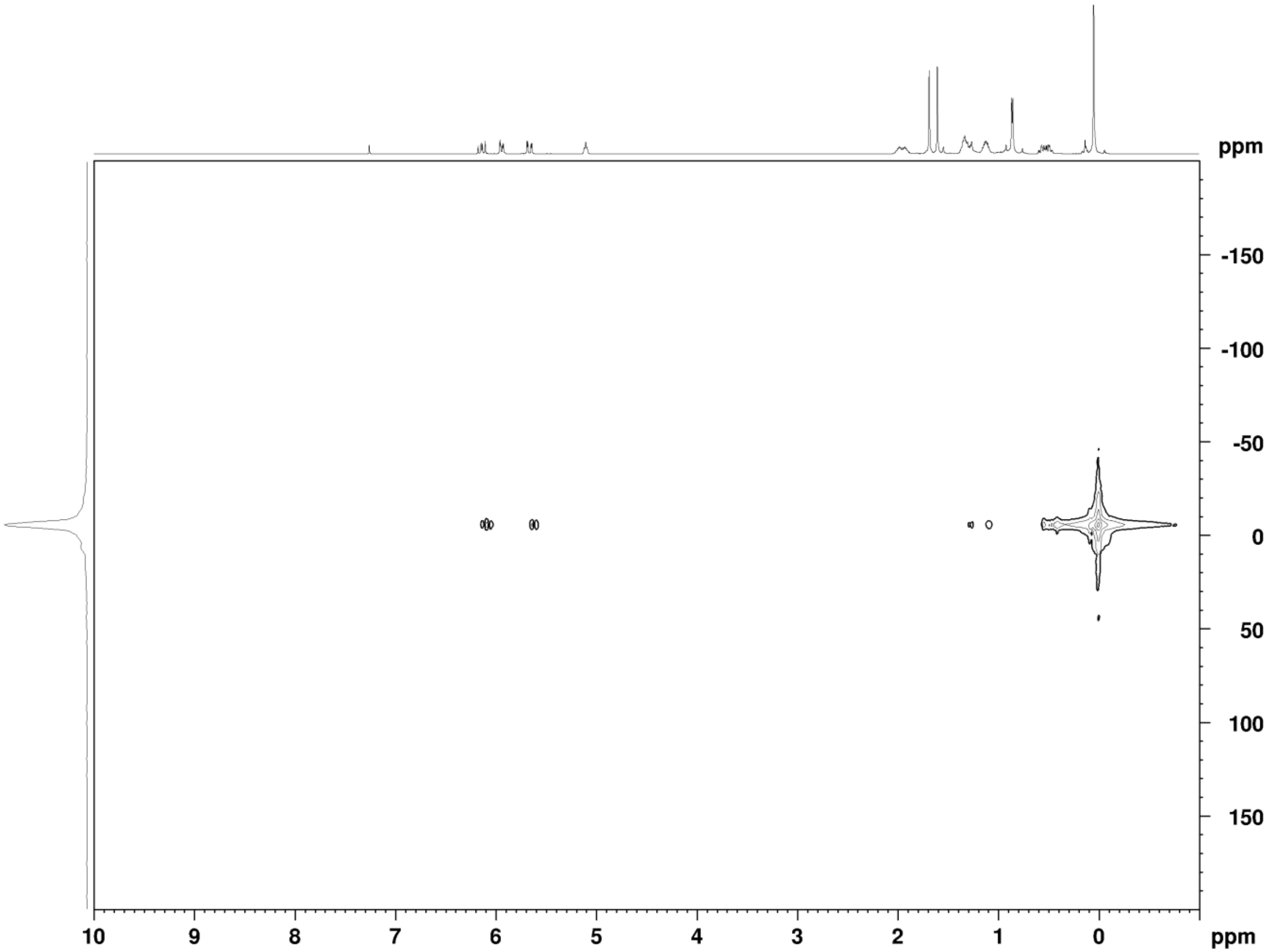

Figure S192. ^{1}H NMR (500 MHz, $CDCl_3$, 298 K) of **7ba**.

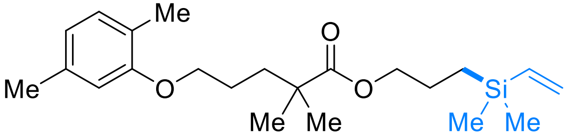

7ba

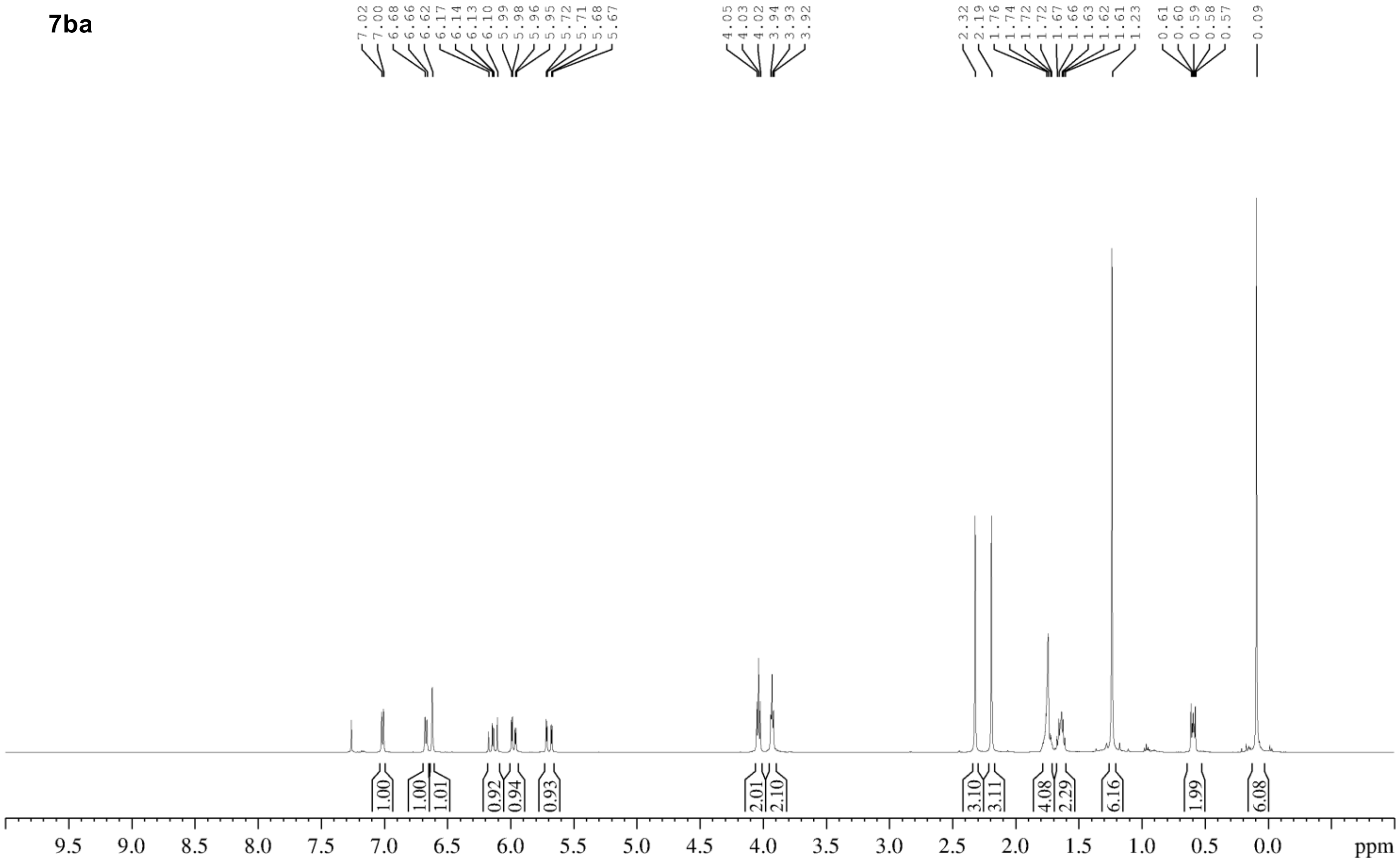

Figure S193. ^{13}C NMR (126 MHz, CDCl$_3$, 298 K) of **7ba**.

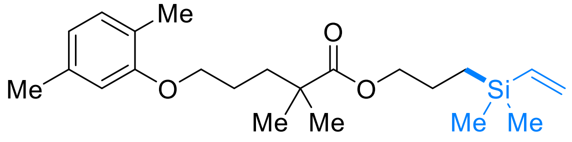

7ba

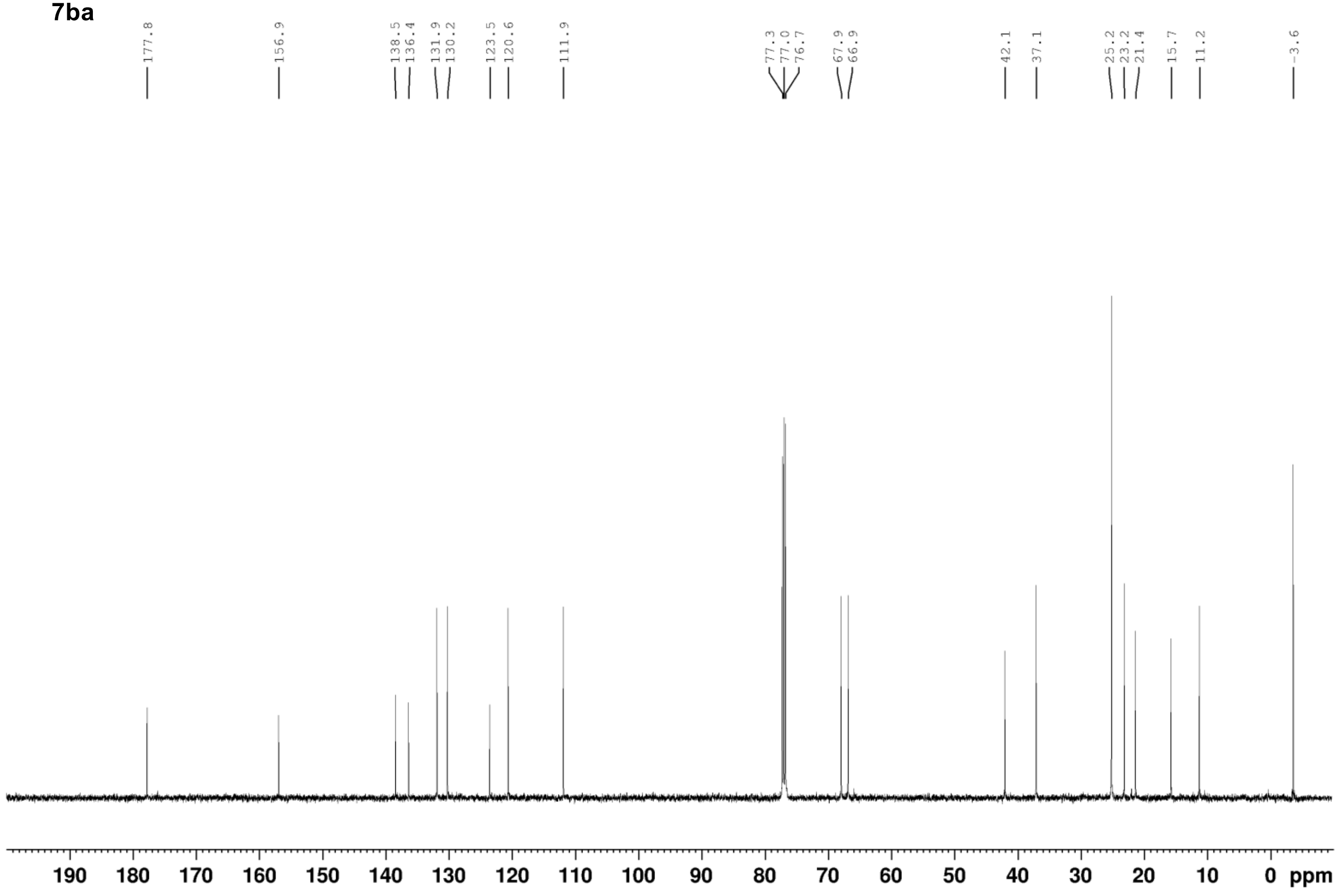

Figure S194. $^{1}H/^{29}Si$ HMQC NMR (500/99 MHz, $CDCl_3$, 298 K, optimized for J = 7 Hz) of **7ba**.

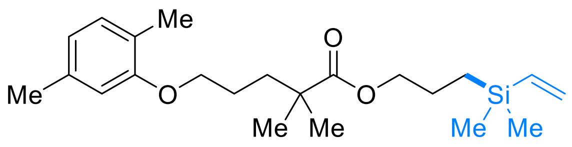

7ba

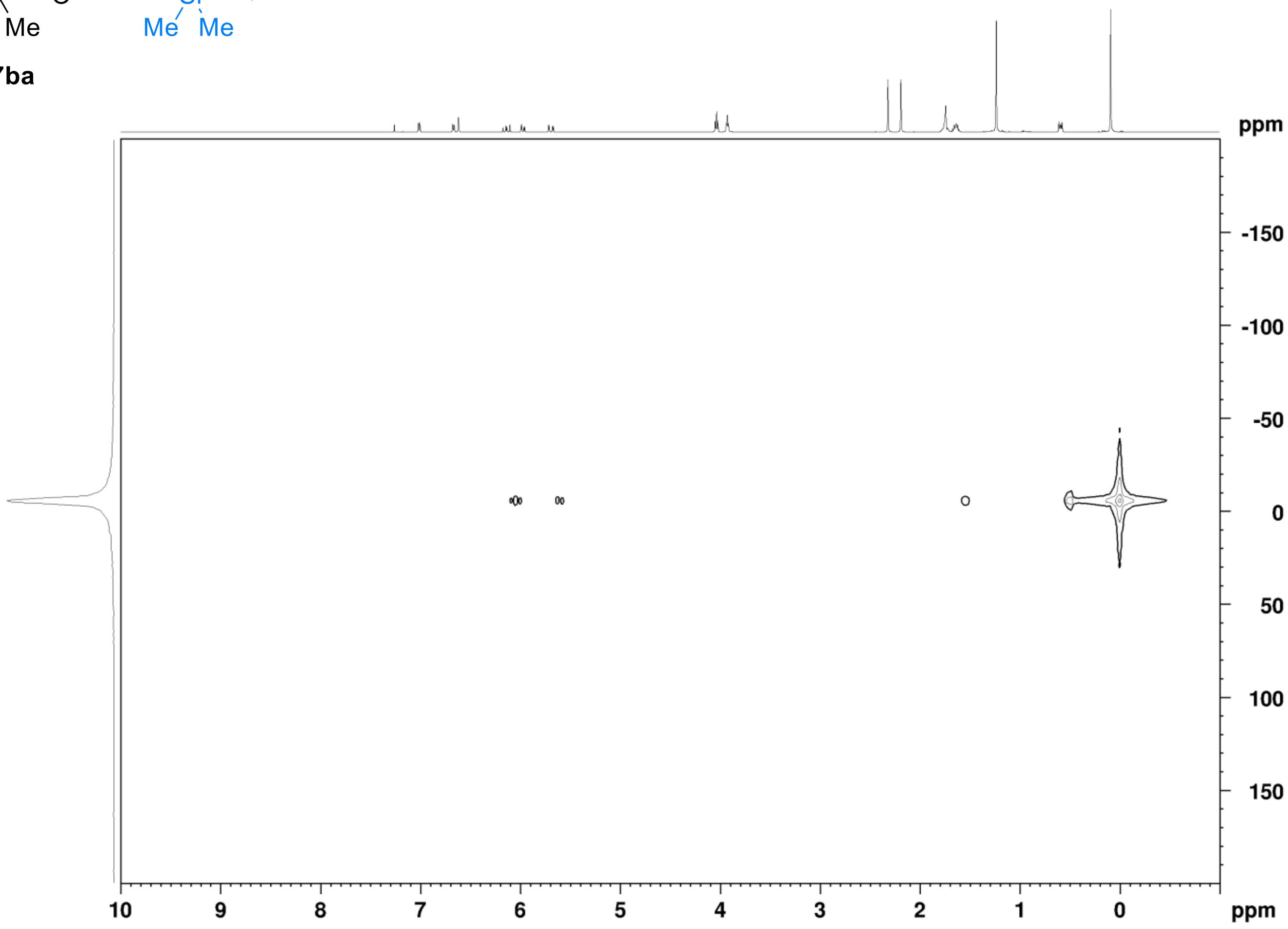

Figure S195. ^{1}H NMR (500 MHz, CDCl$_3$, 298 K) of **7ca**.

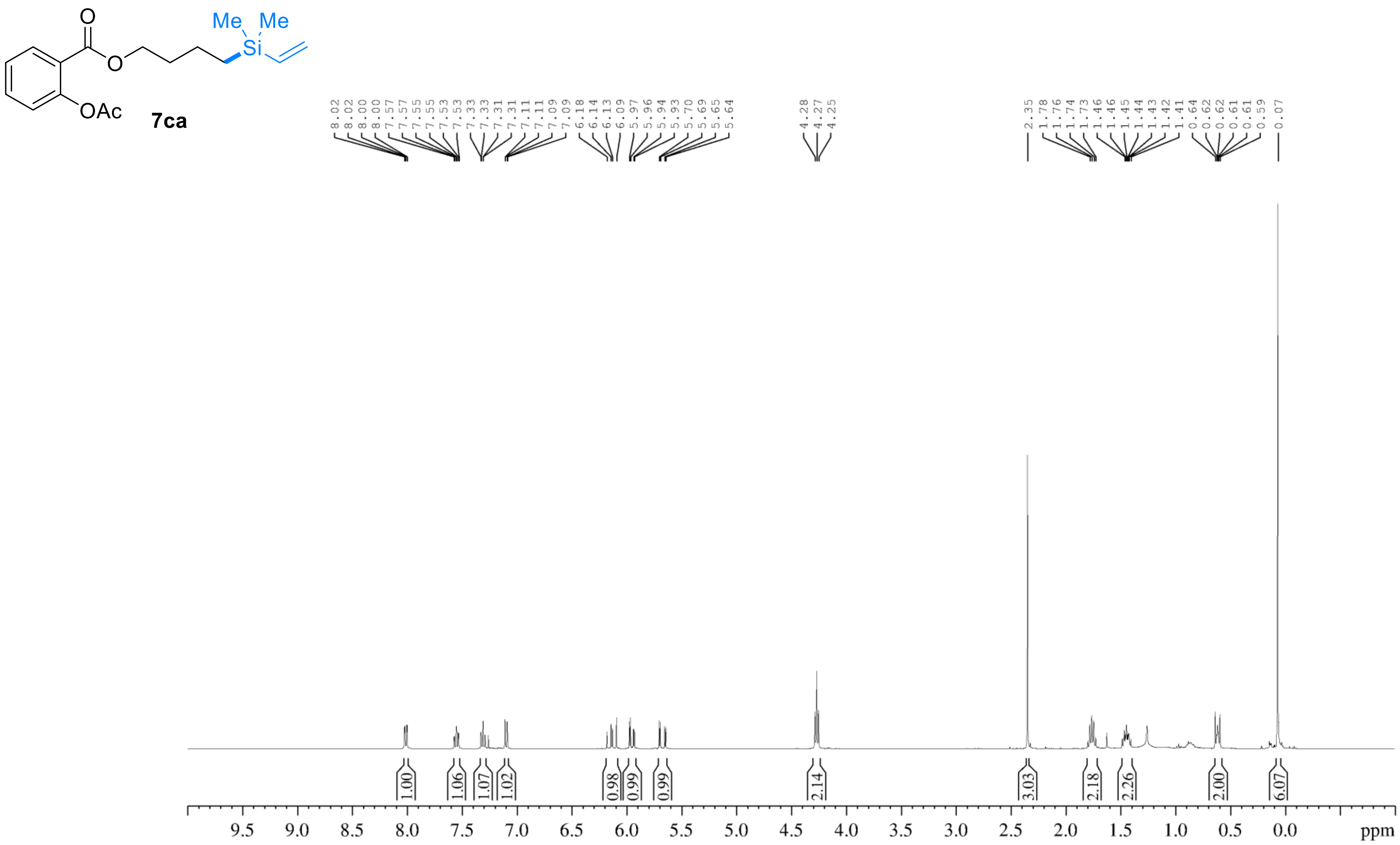

Figure S196. ^{13}C NMR (126 MHz, $CDCl_3$, 298 K) of **7ca**.

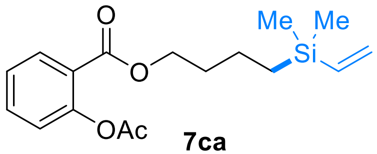

7ca

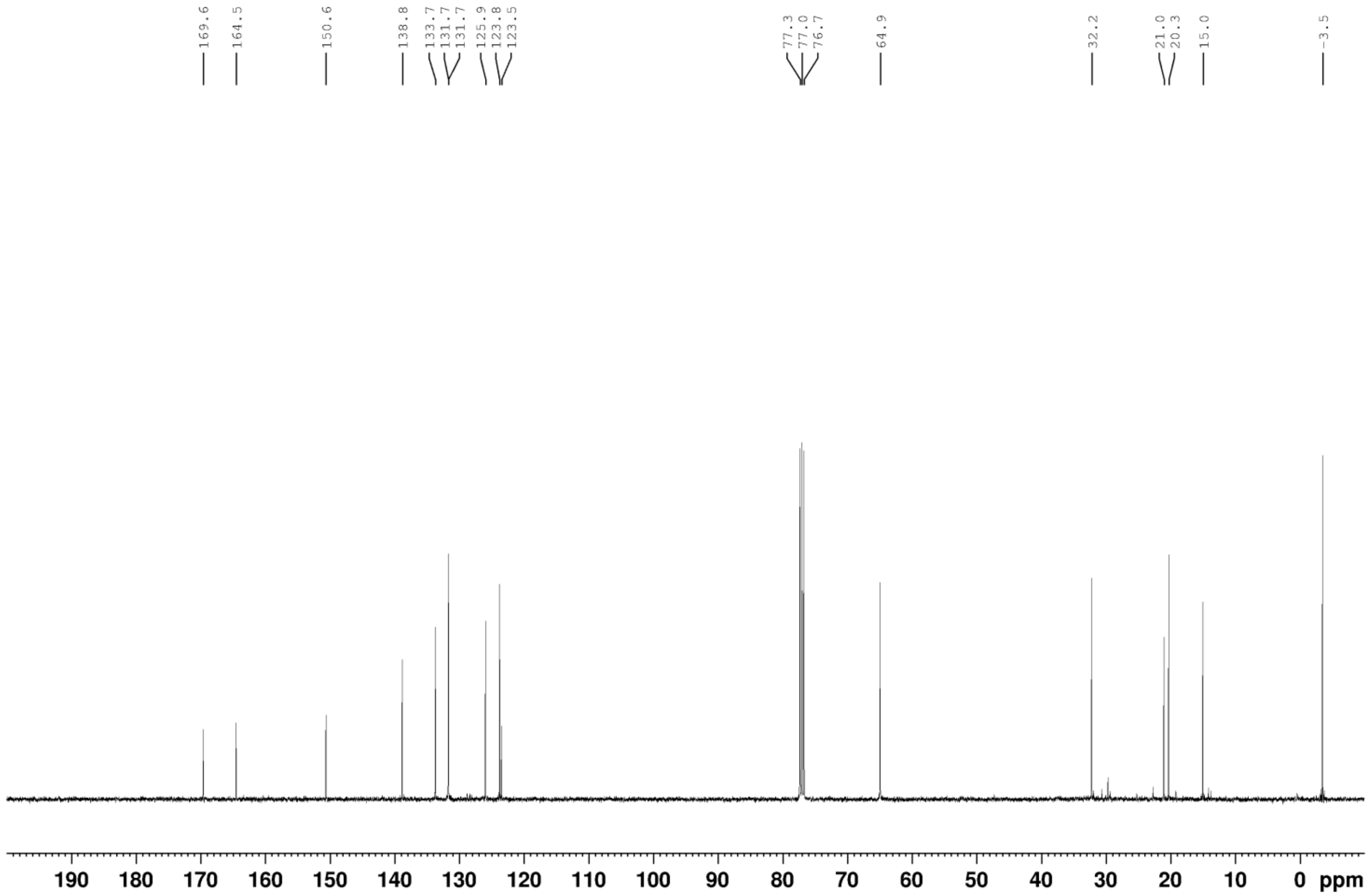

Figure S197. $^{1}H/^{29}Si$ HMQC NMR (500/99 MHz, $CDCl_3$, 298 K, optimized for J = 7 Hz) of **7ca**.

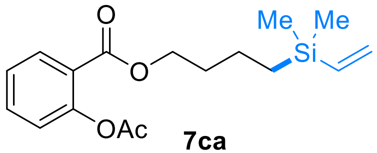

7ca

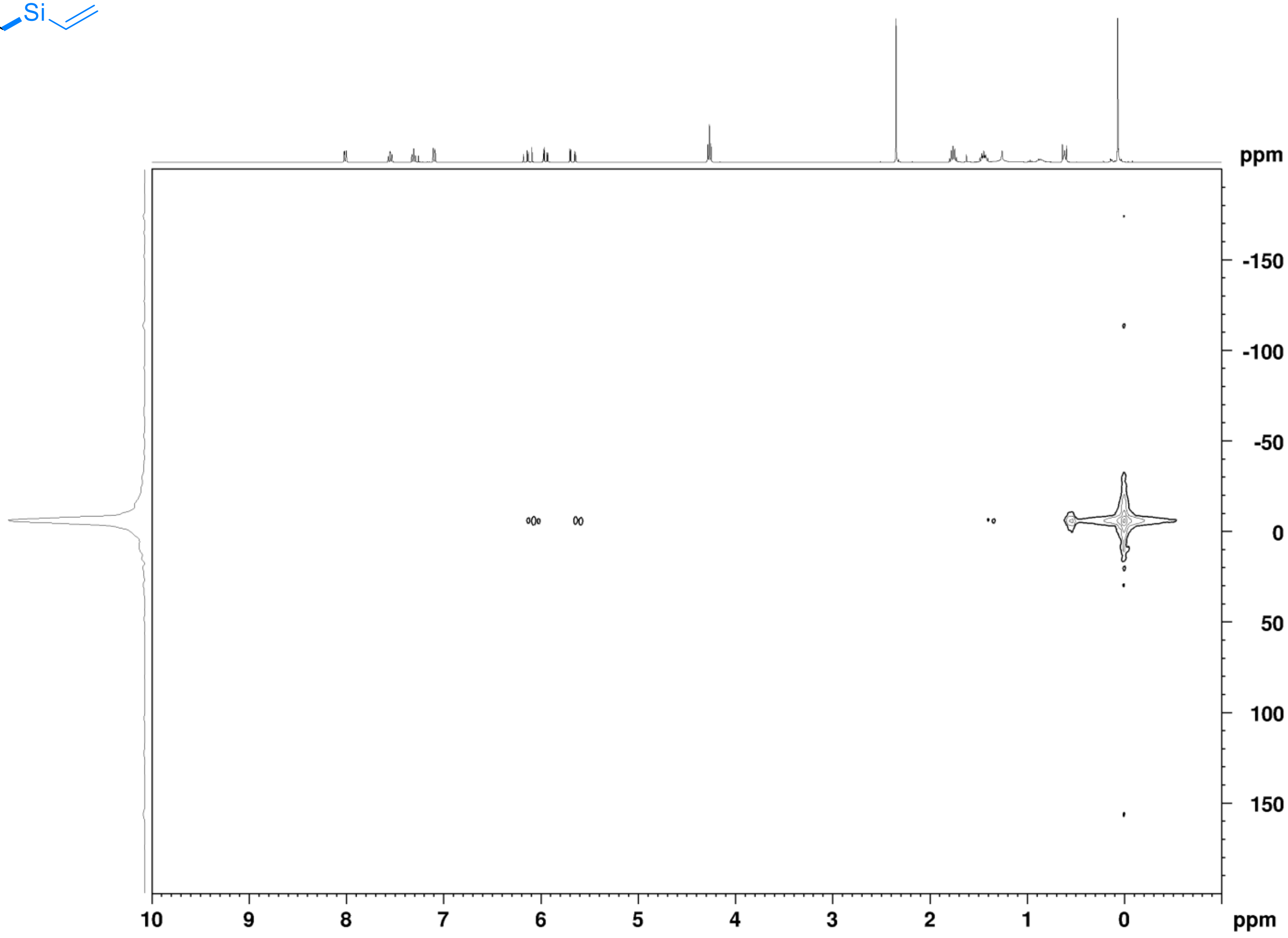

Figure S198. ^{1}H NMR (500 MHz, $CDCl_3$, 298 K) of **7da**.

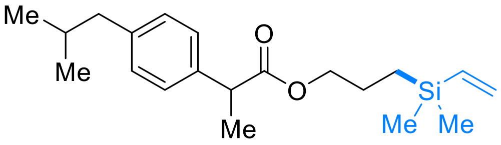

7da

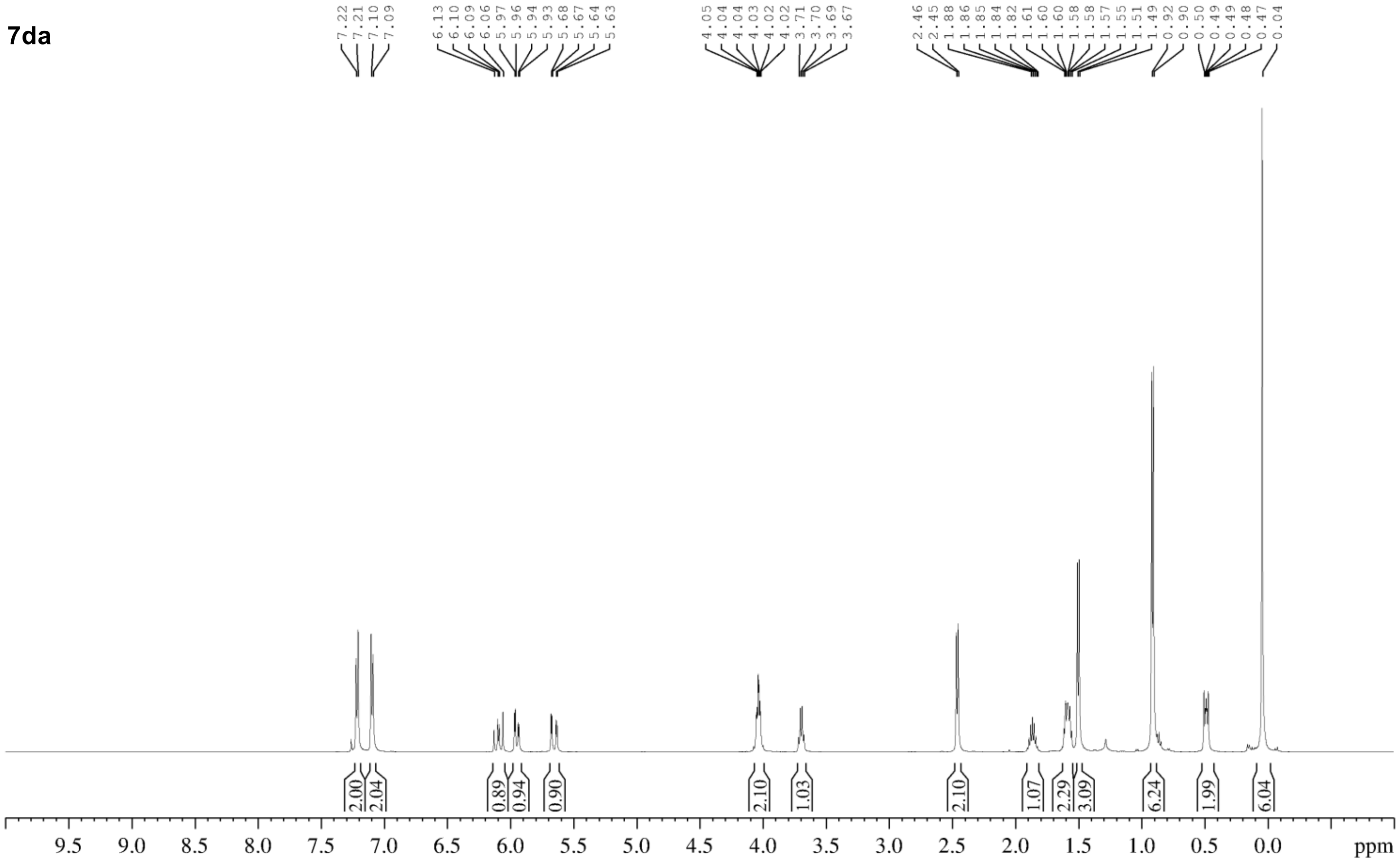

Figure S199. ^{13}C NMR (126 MHz, $CDCl_3$, 298 K) of **7da**.

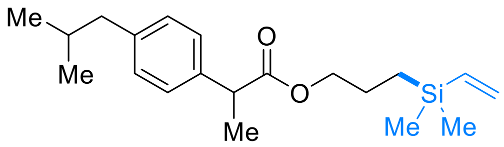

7da

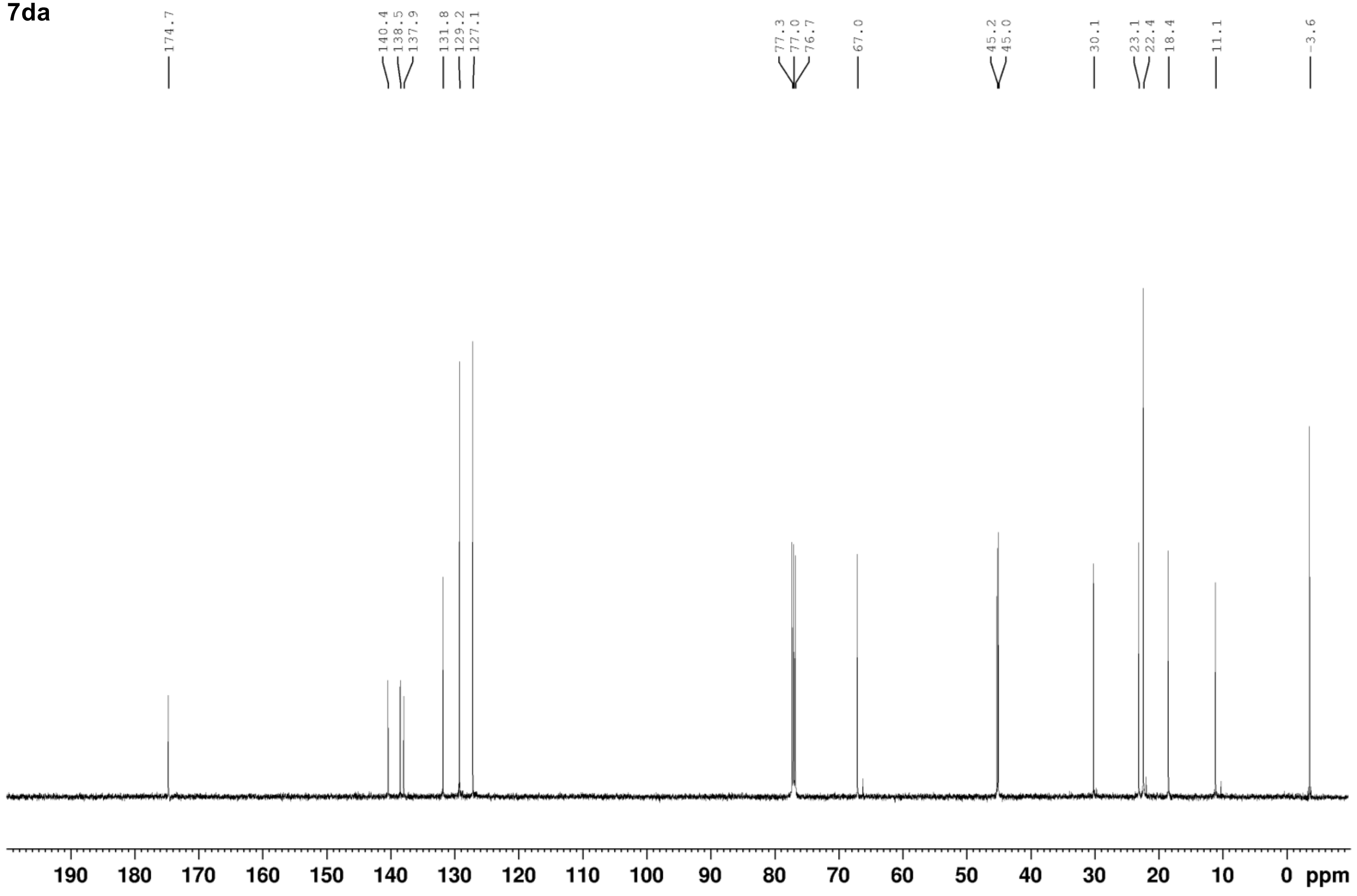

Figure S200. $^{1}H/^{29}Si$ HMQC NMR (500/99 MHz, $CDCl_3$, 298 K, optimized for J = 7 Hz) of **7da**.

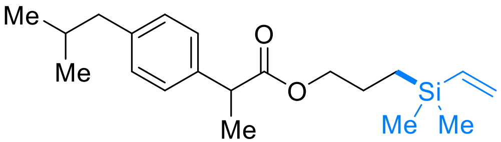

7da

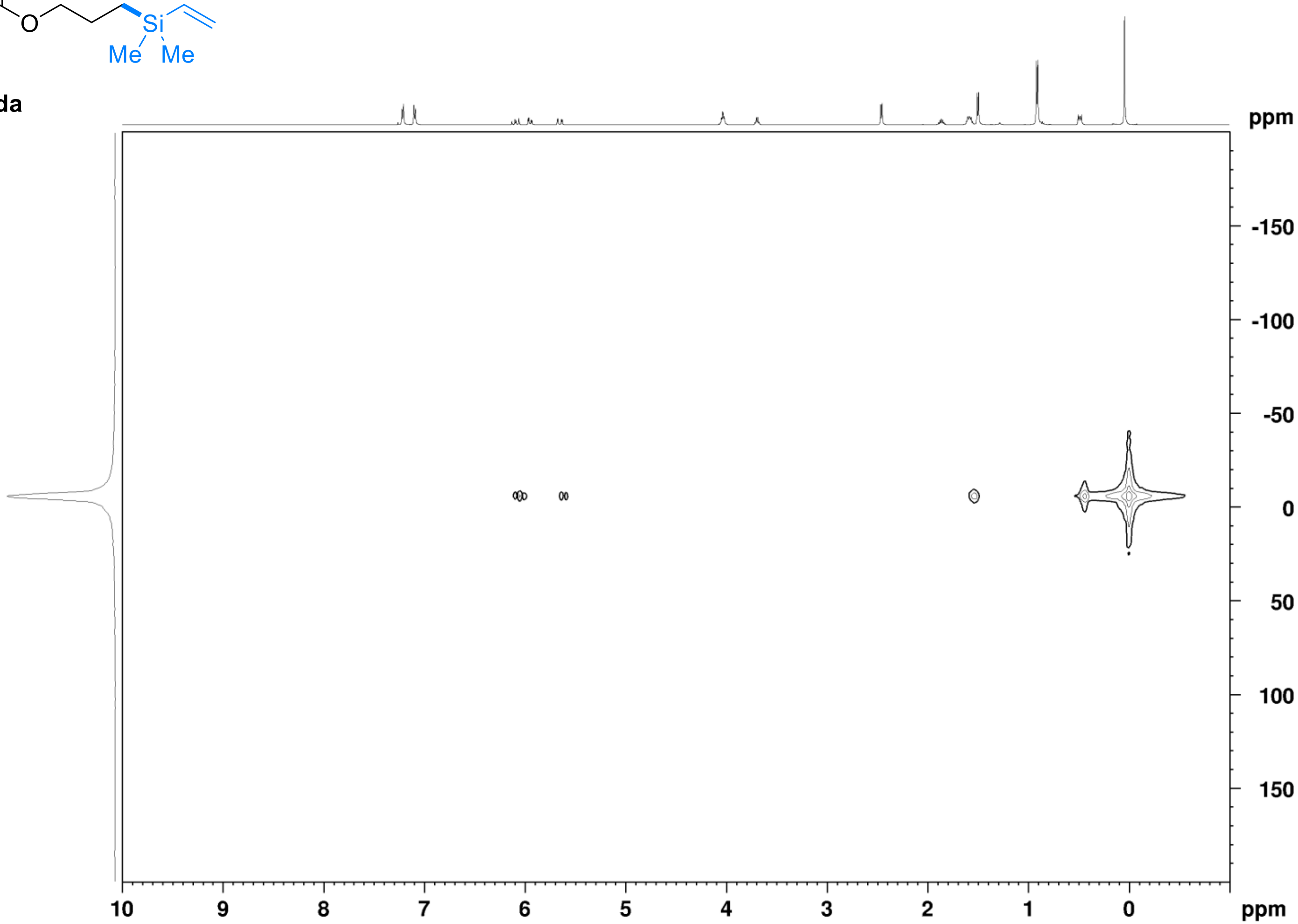

Figure S201. ^{1}H NMR (500 MHz, $CDCl_3$, 298 K) of **7ea**.

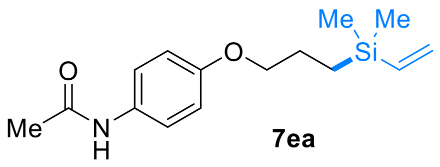

7ea

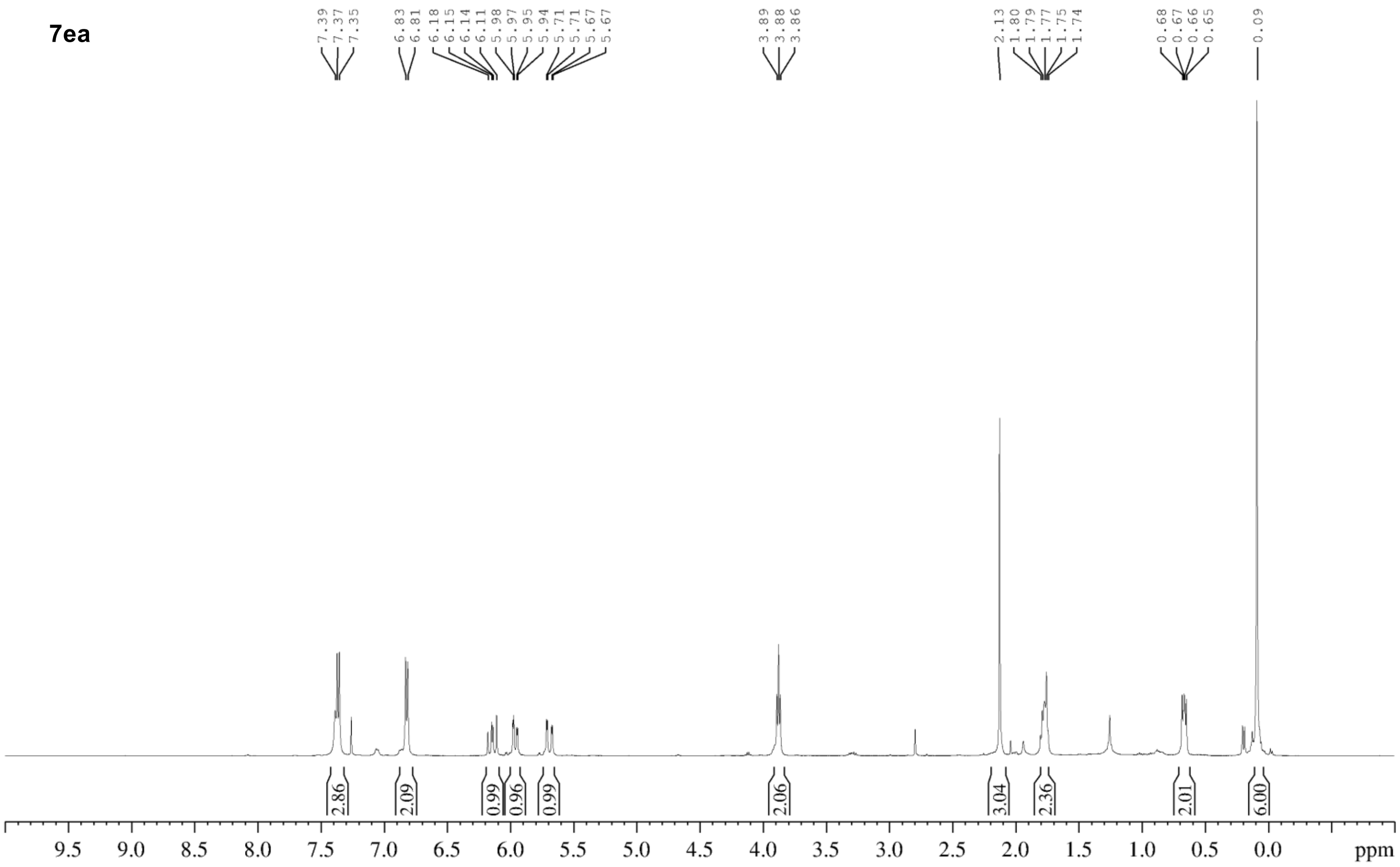

Figure S202. ^{13}C NMR (126 MHz, $CDCl_3$, 298 K) of **7ea**

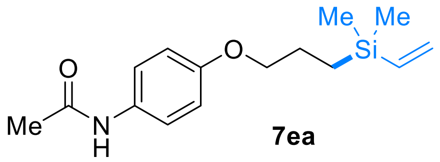

7ea

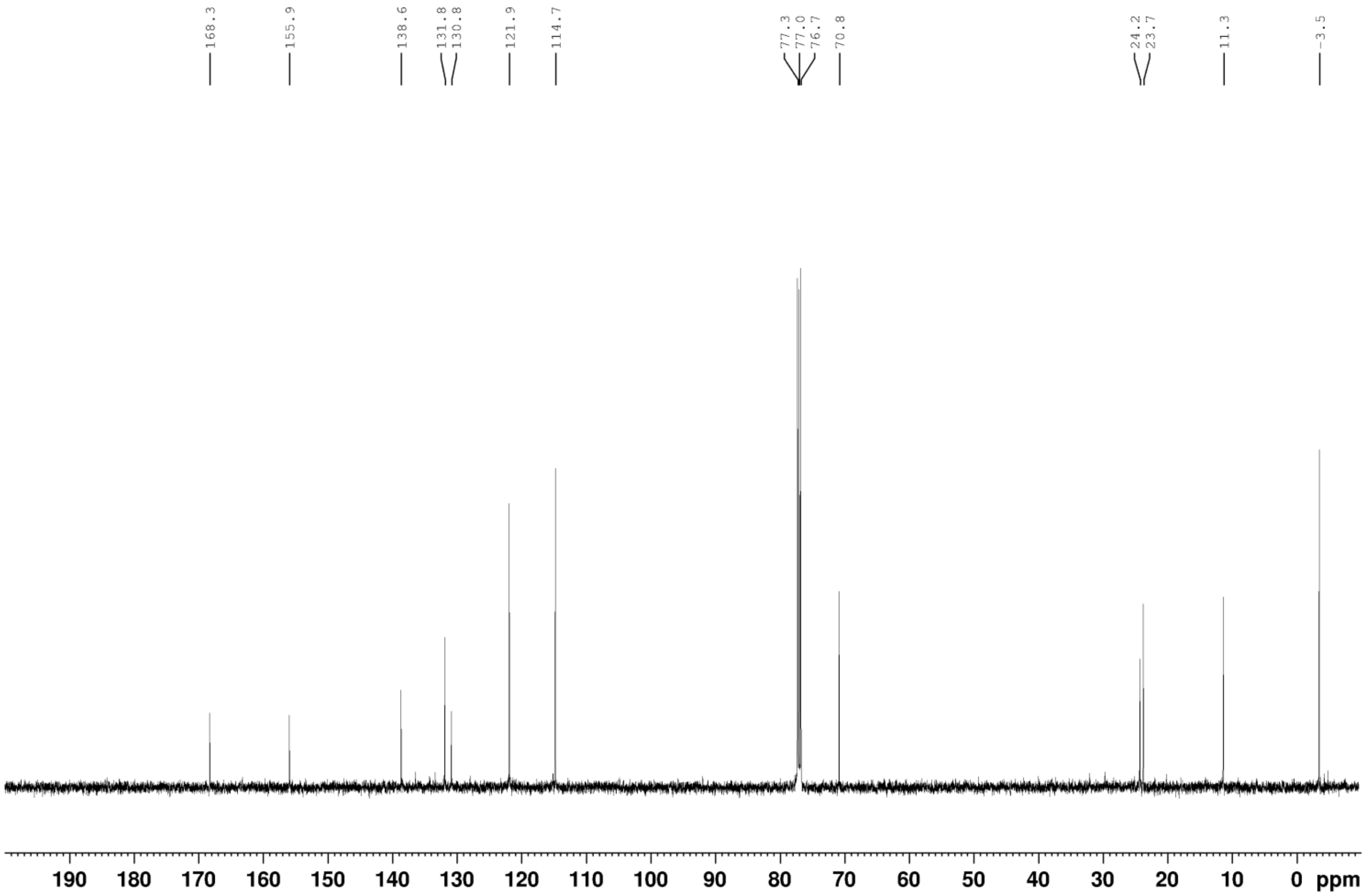

Figure S203. $^{1}H/^{29}Si$ HMQC NMR (500/99 MHz, $CDCl_3$, 298 K, optimized for J = 7 Hz) of **7ea**.

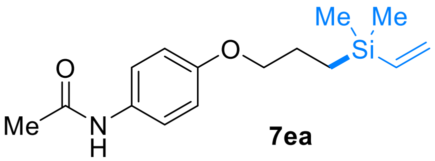

7ea

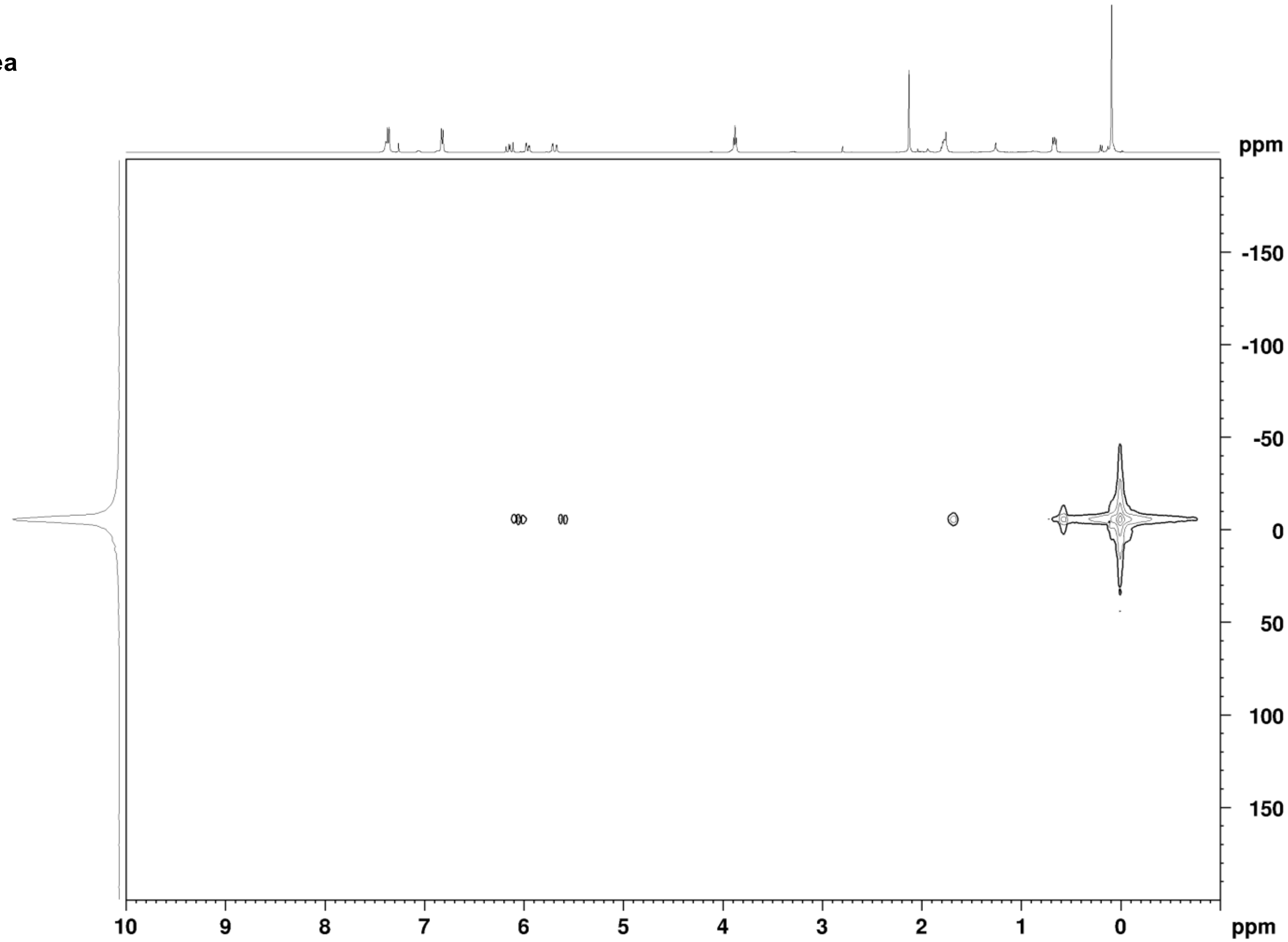

Figure S204. ^{1}H NMR (500 MHz, $CDCl_3$, 298 K) of **7fa**.

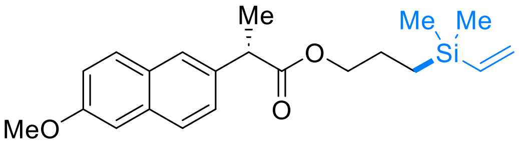

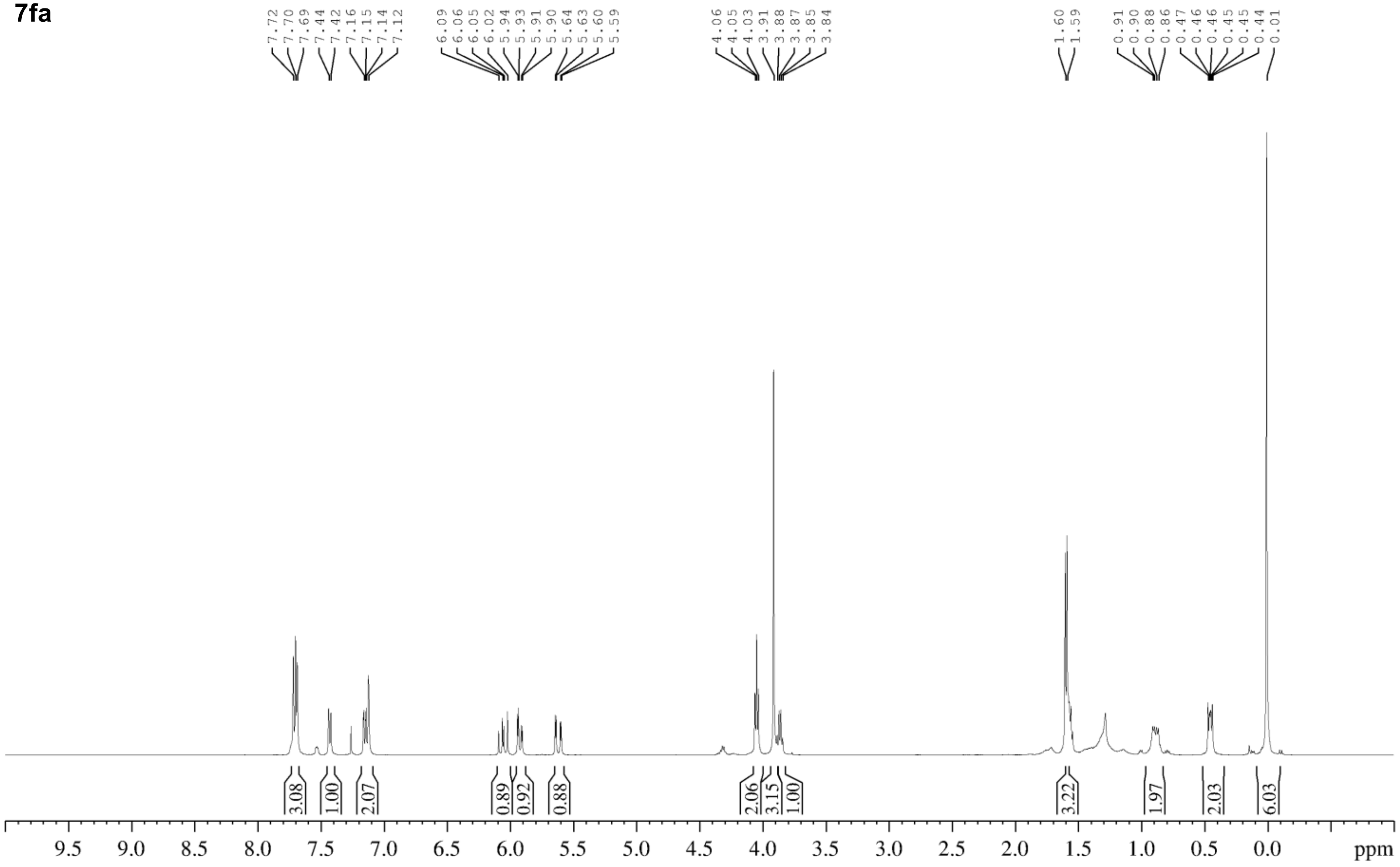

Figure S205. ^{13}C NMR (126 MHz, $CDCl_3$, 298 K) of **7fa**.

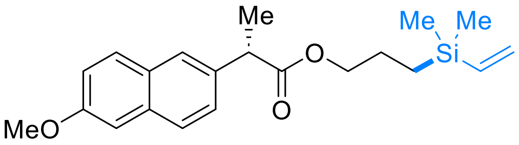
7fa

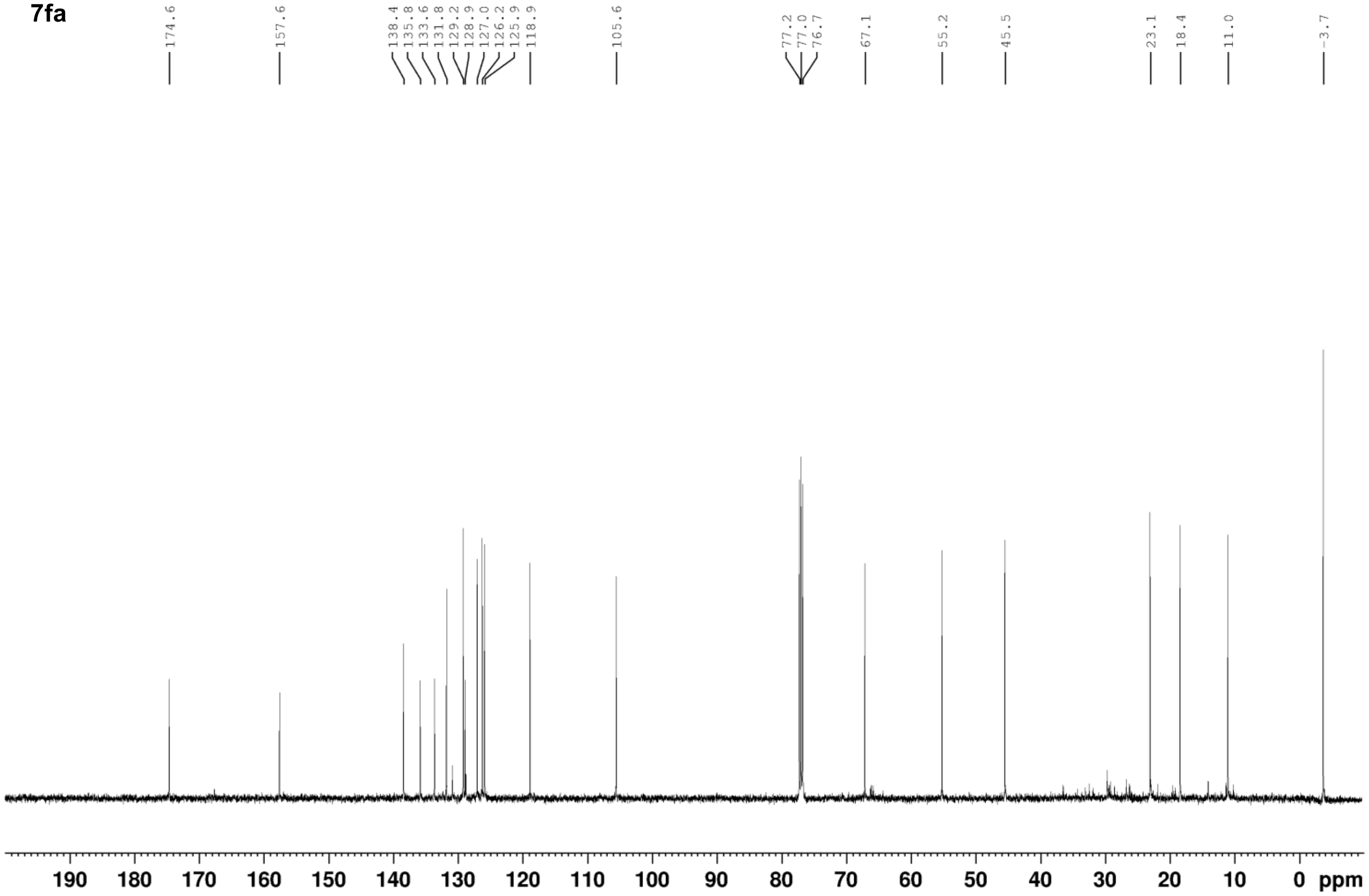

Figure S206. $^{1}H/^{29}Si$ HMQC NMR (500/99 MHz, $CDCl_3$, 298 K, optimized for J = 7 Hz) of **7fa**.

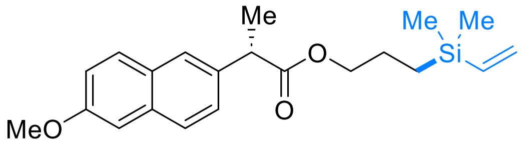

7fa

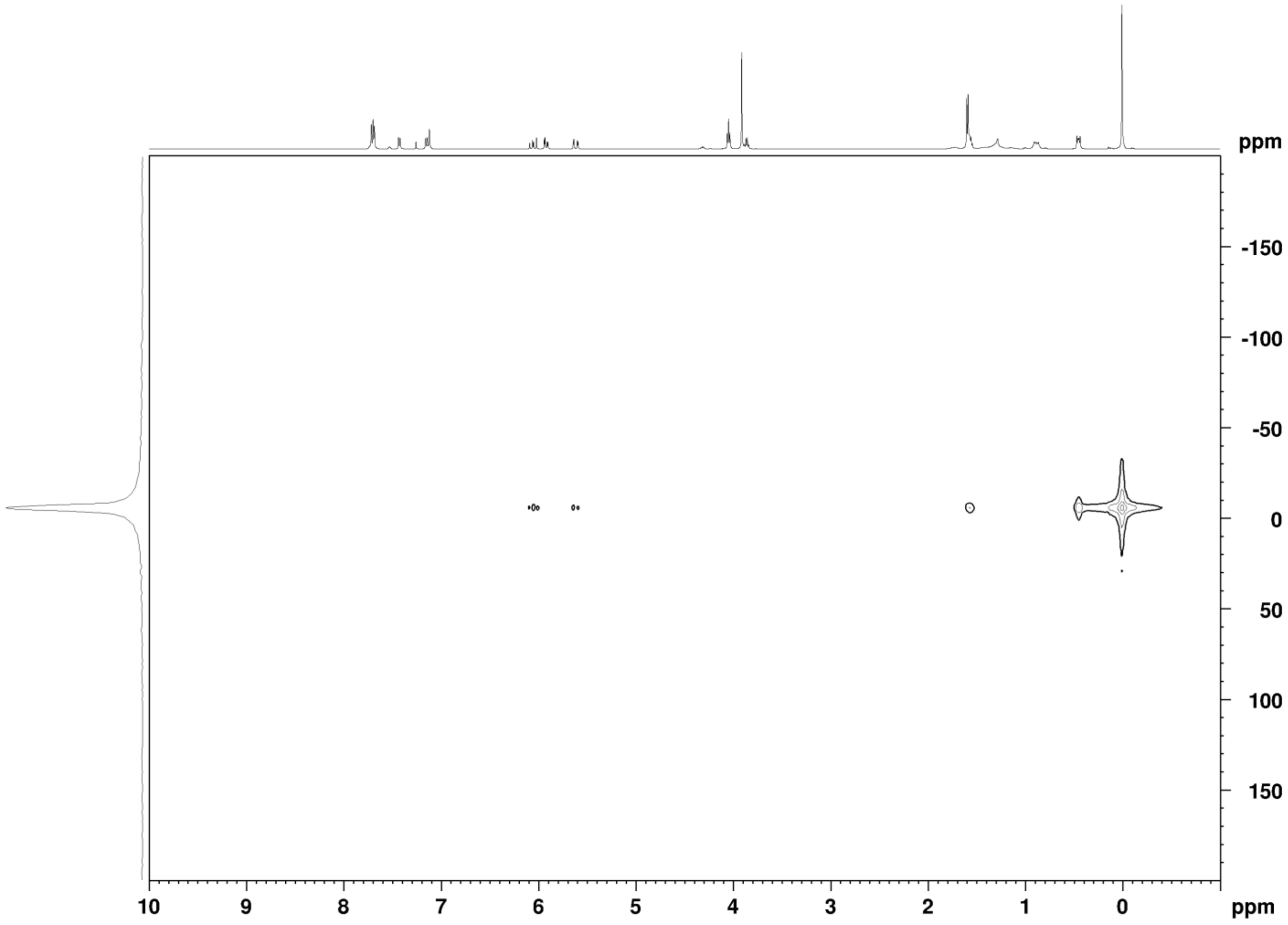

Figure S207. ^{1}H NMR (500 MHz, $CDCl_3$, 298 K) of **7ga**.

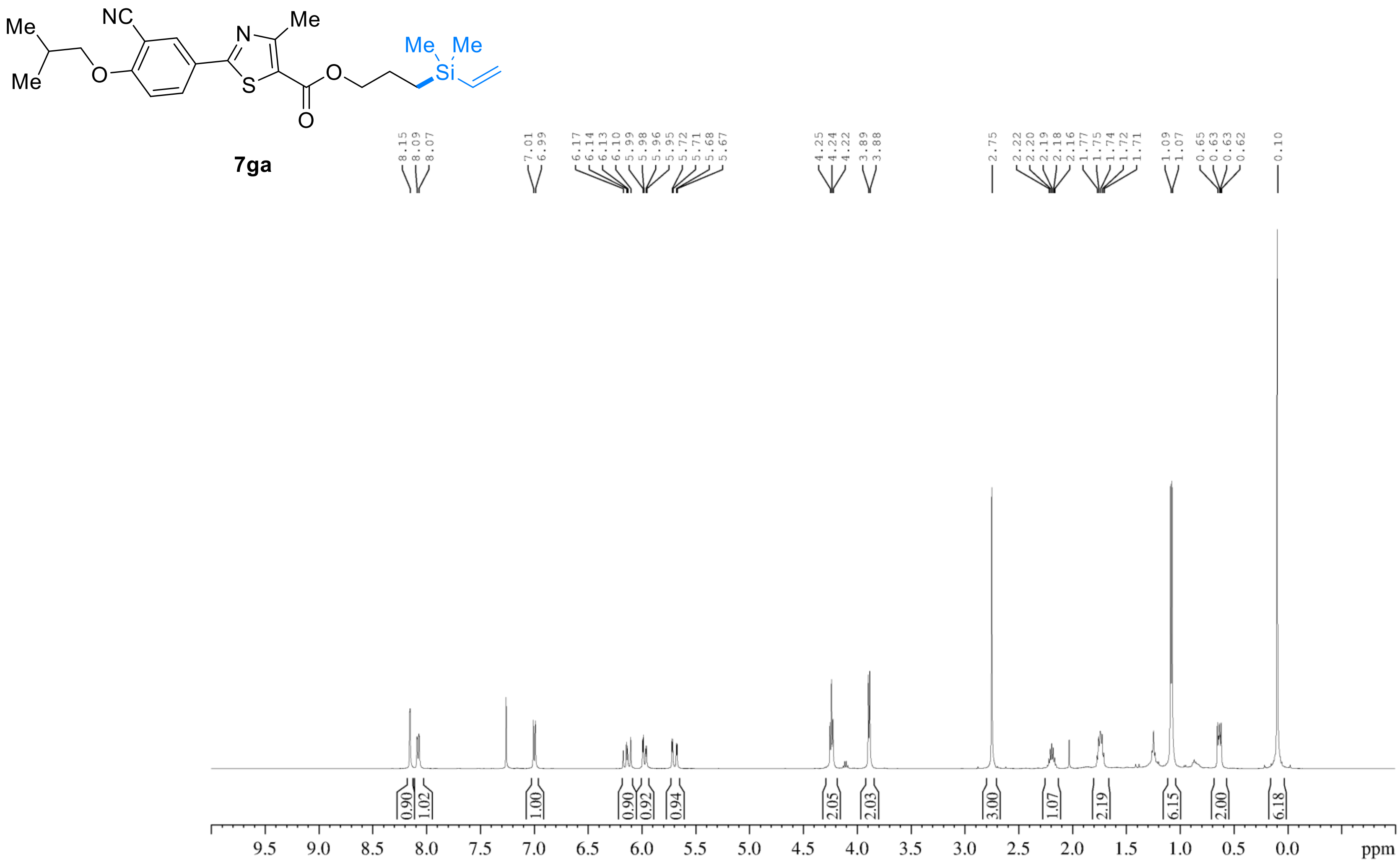

Figure S208. ^{13}C NMR (126 MHz, $CDCl_3$, 298 K) of **7ga**.

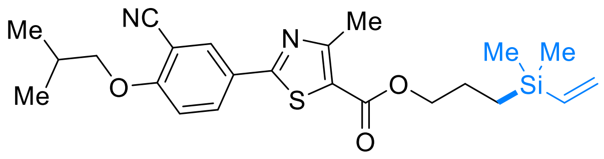

7ga

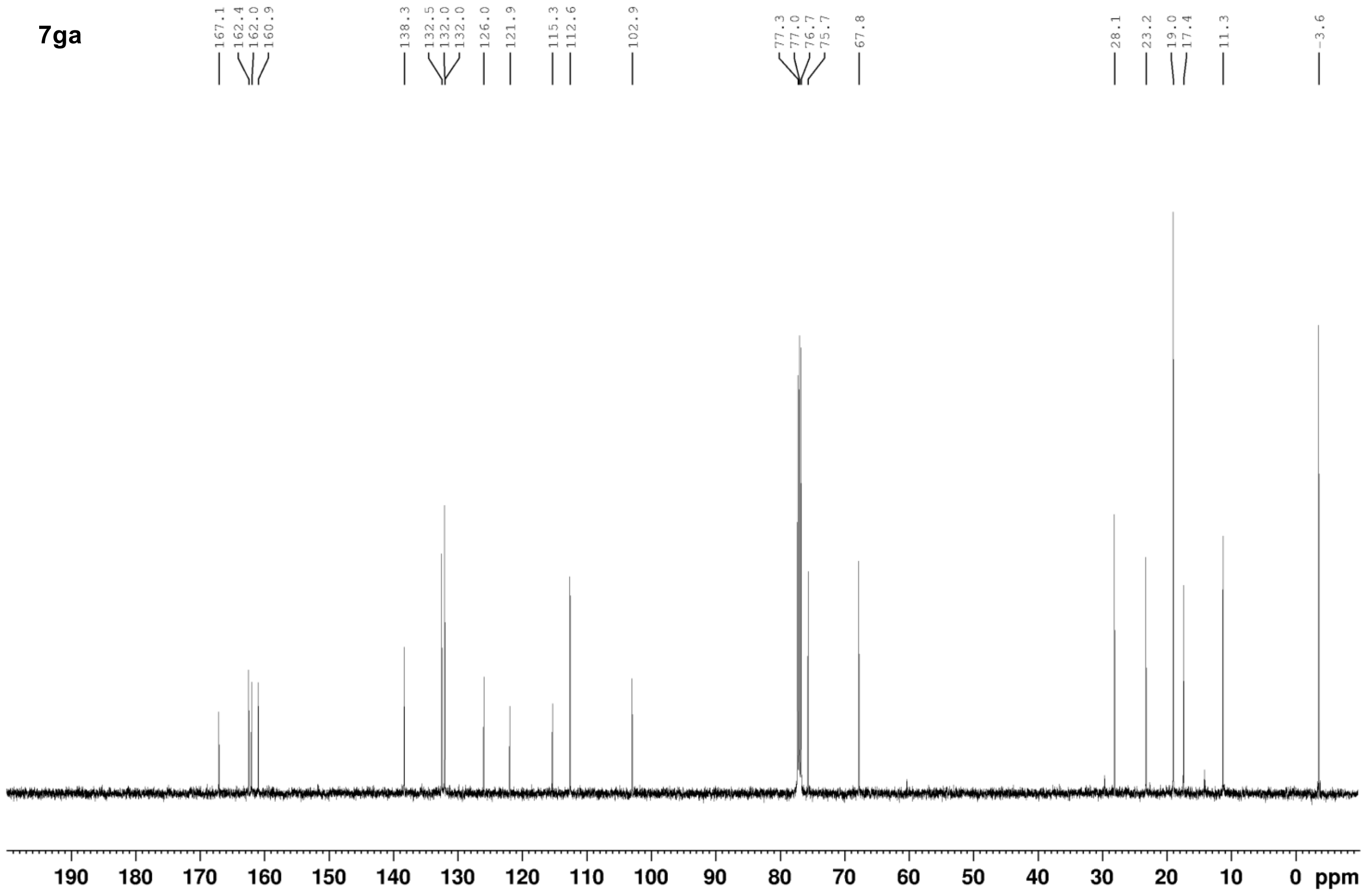

Figure S209. $^{1}H/^{29}Si$ HMQC NMR (500/99 MHz, $CDCl_3$, 298 K, optimized for J = 7 Hz) of **7ga**.

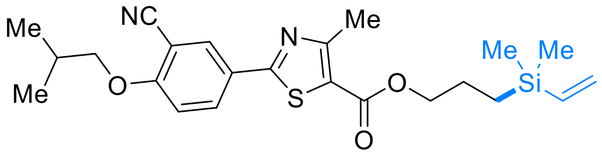

7ga

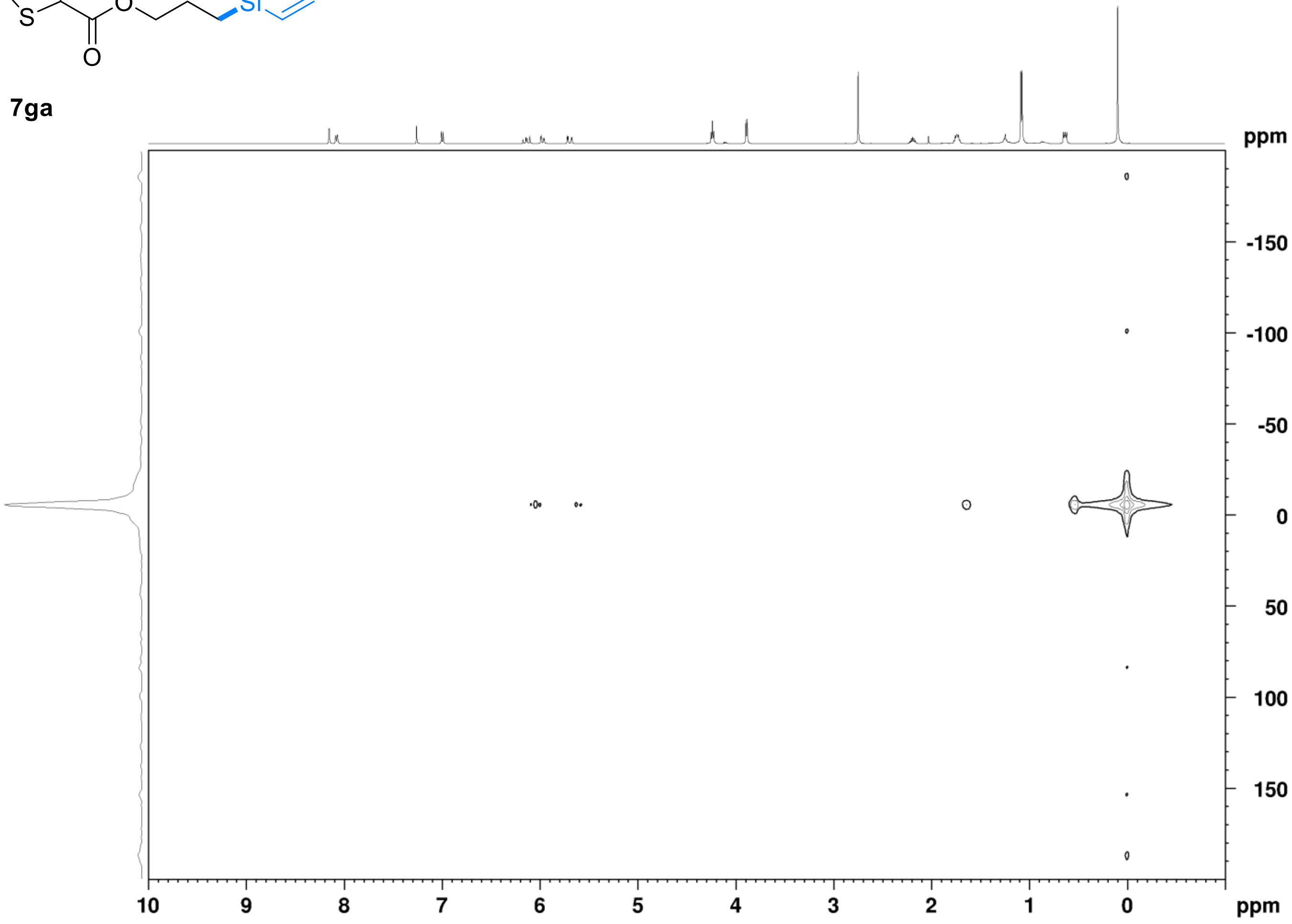

Figure S210. ^{1}H NMR (500 MHz, $CDCl_3$, 298 K) of **7ha**.

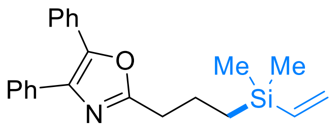

7ha

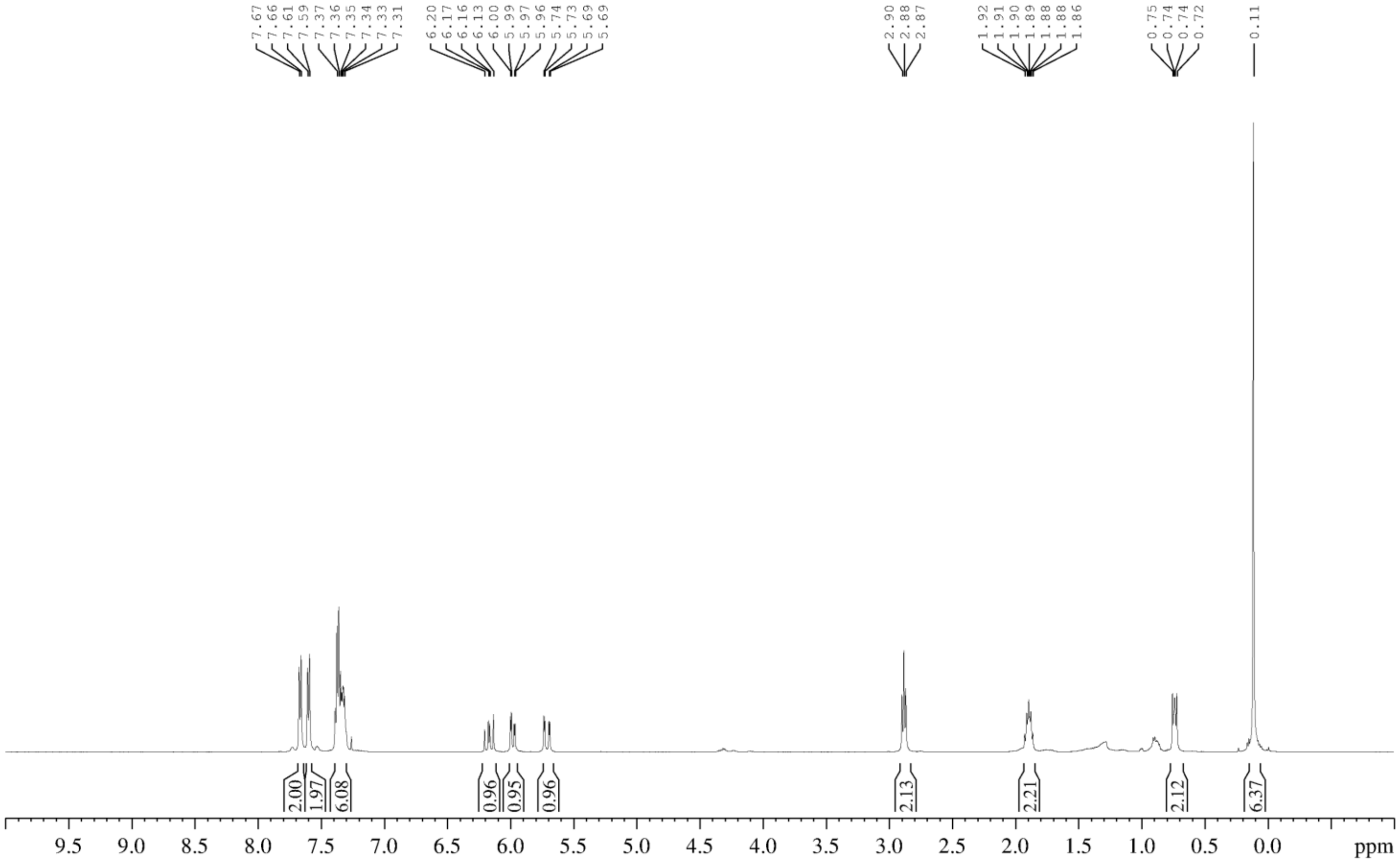

Figure S211. ^{13}C NMR (126 MHz, $CDCl_3$, 298 K) of **7ha**.

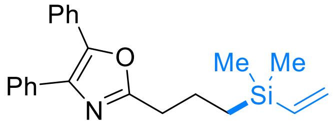

7ha

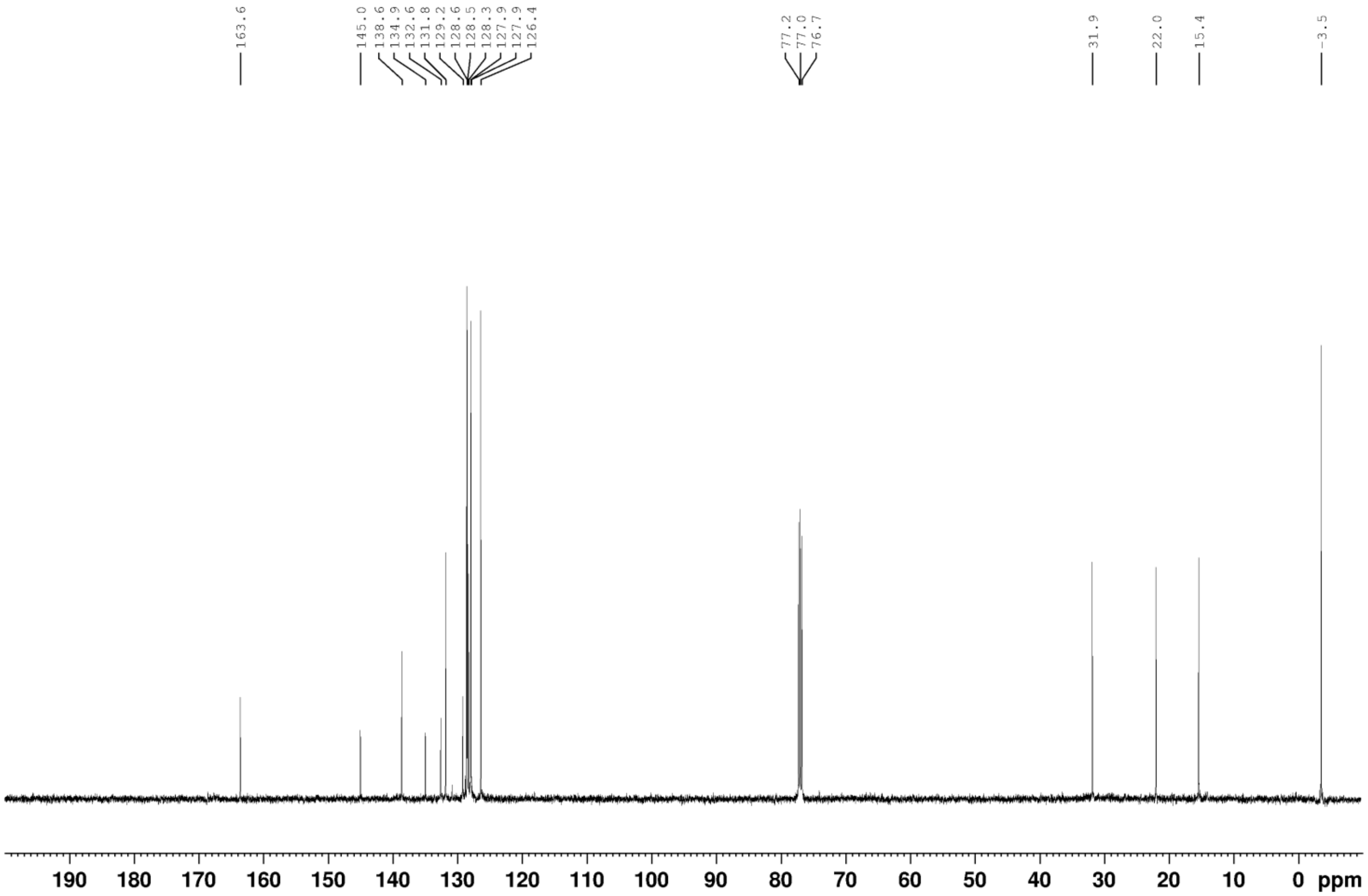

Figure S212. $^{1}H/^{29}Si$ HMQC NMR (500/99 MHz, $CDCl_3$, 298 K, optimized for J = 7 Hz) of **7ha**.

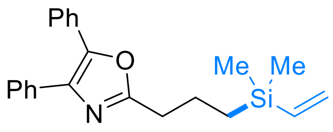

7ha

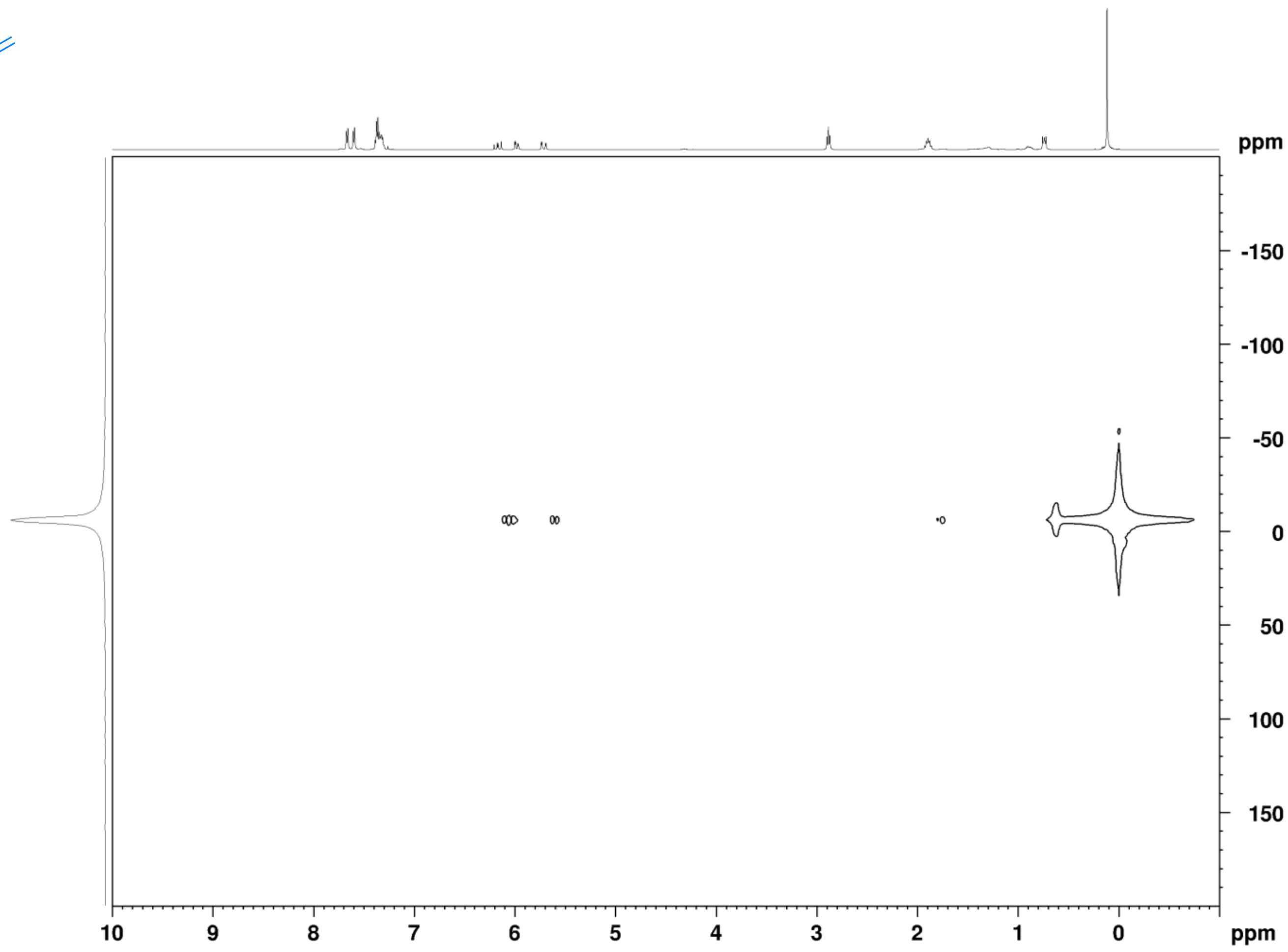

Figure S213. ^{1}H NMR (500 MHz, $CDCl_3$, 298 K) of **7ia**.

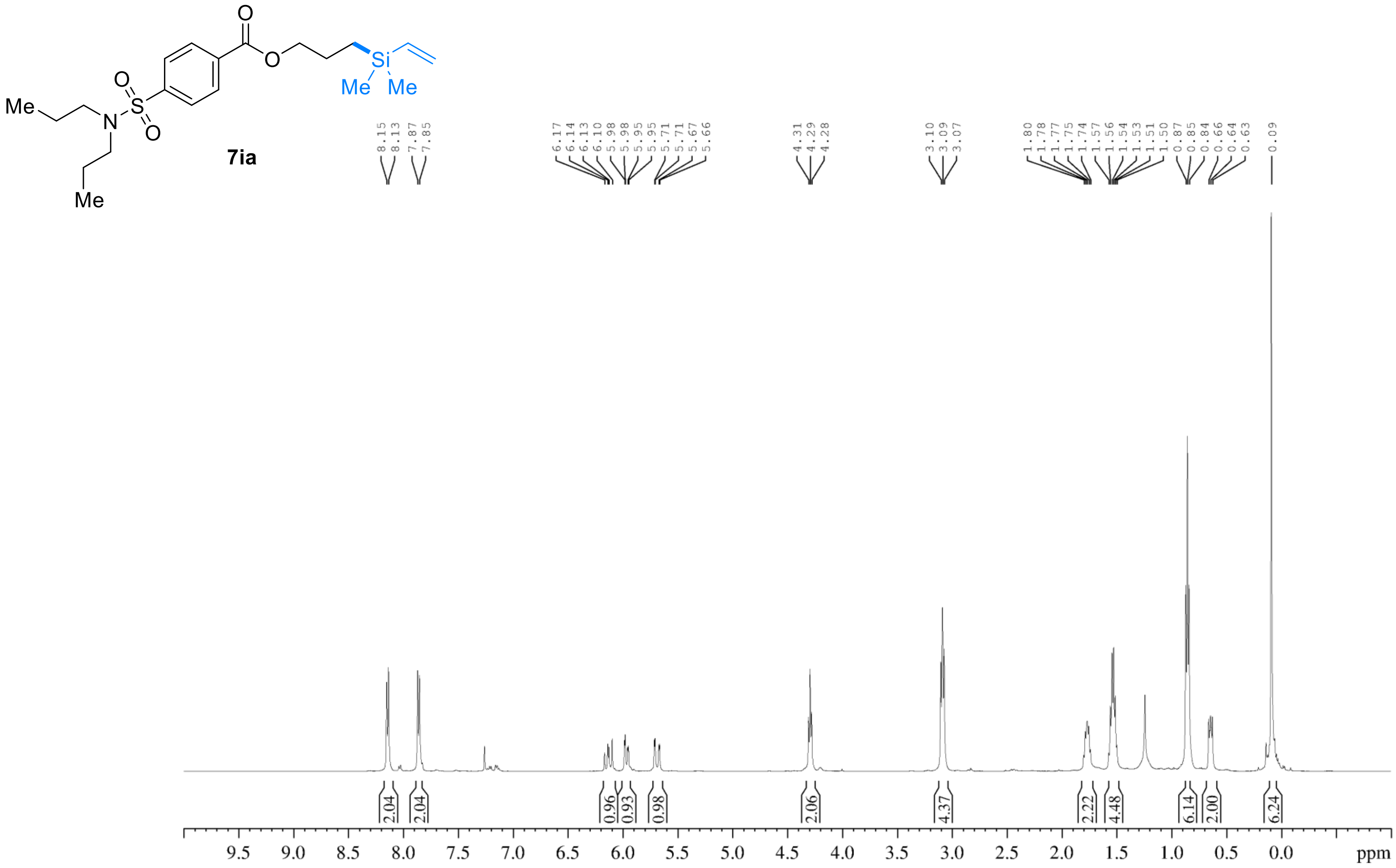

Figure S214. ^{13}C NMR (126 MHz, $CDCl_3$, 298 K) of **7ia**.

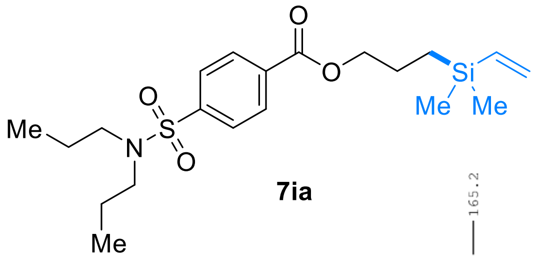

7ia

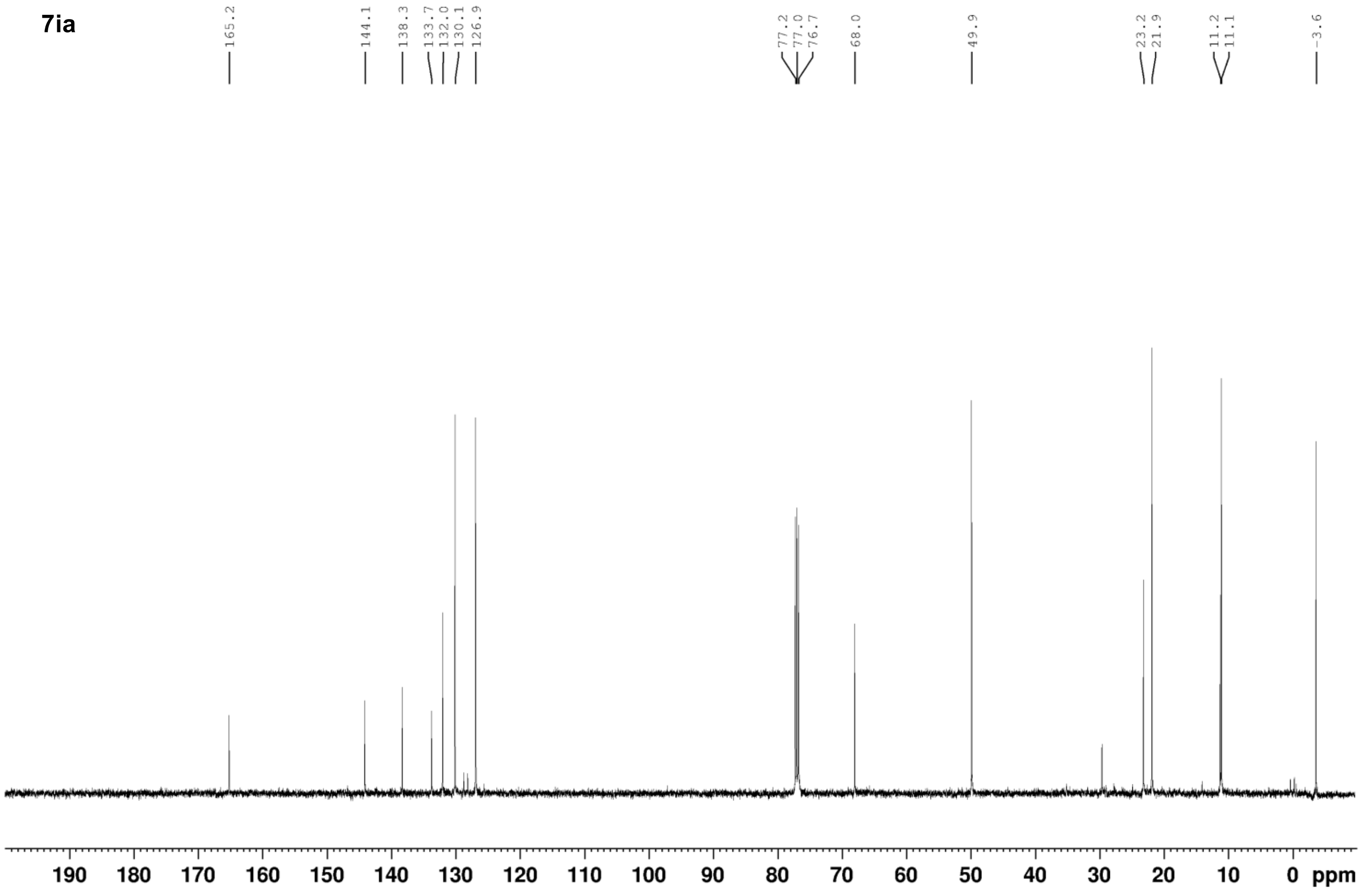

Figure S215. $^{1}H/^{29}Si$ HMQC NMR (500/99 MHz, $CDCl_3$, 298 K, optimized for J = 7 Hz) of **7ia**.

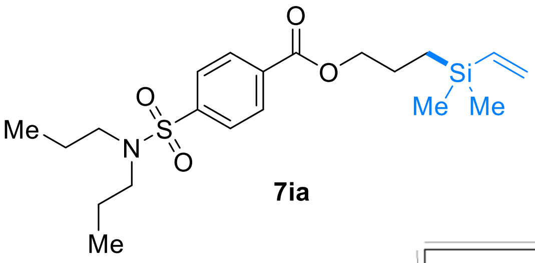

7ia

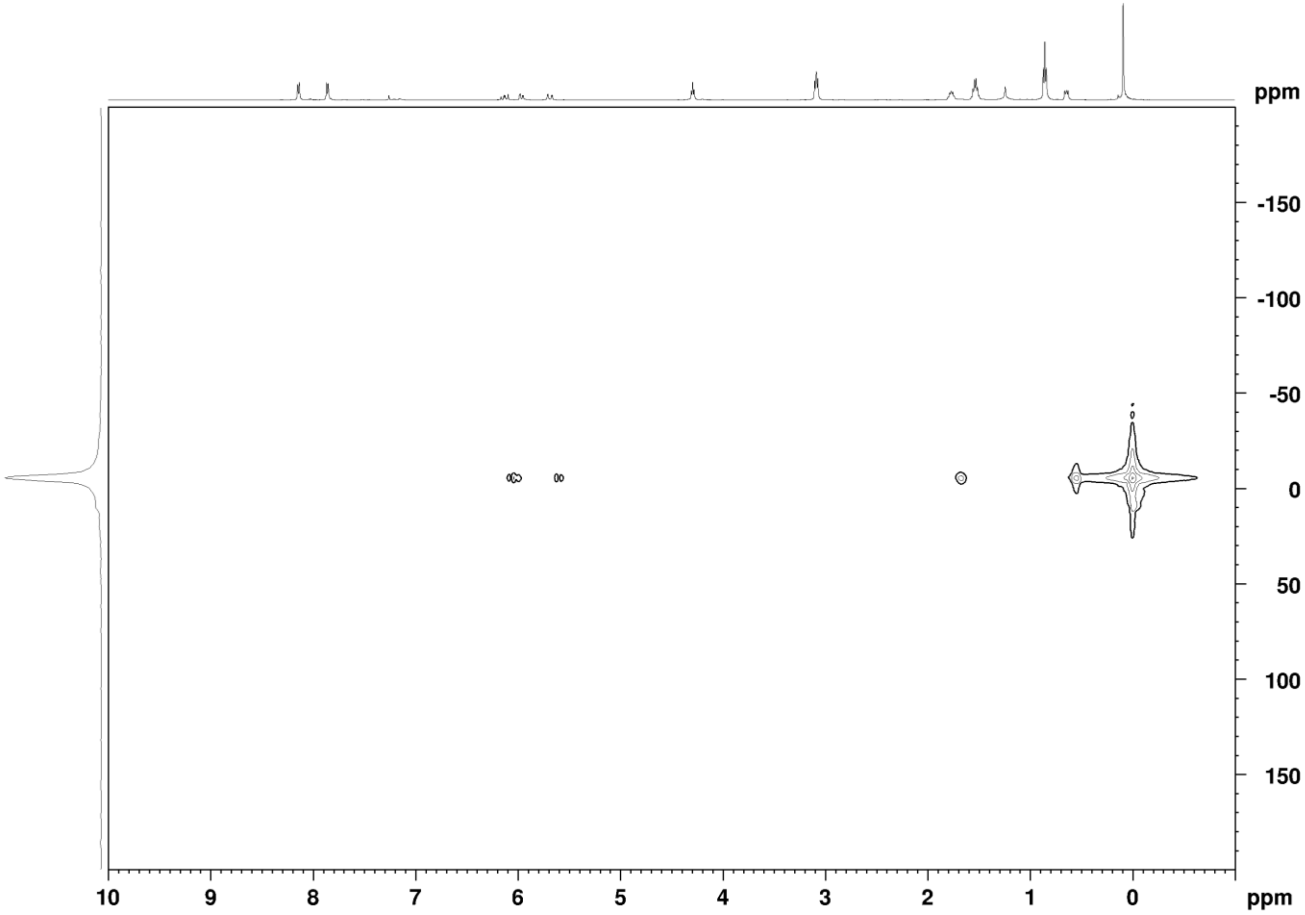

Figure S216. ^{1}H NMR (500 MHz, $CDCl_3$, 298 K) of **7ja**.

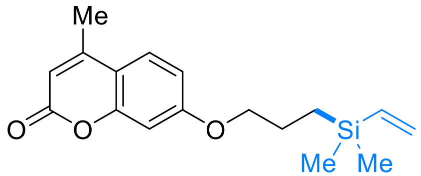

7ja

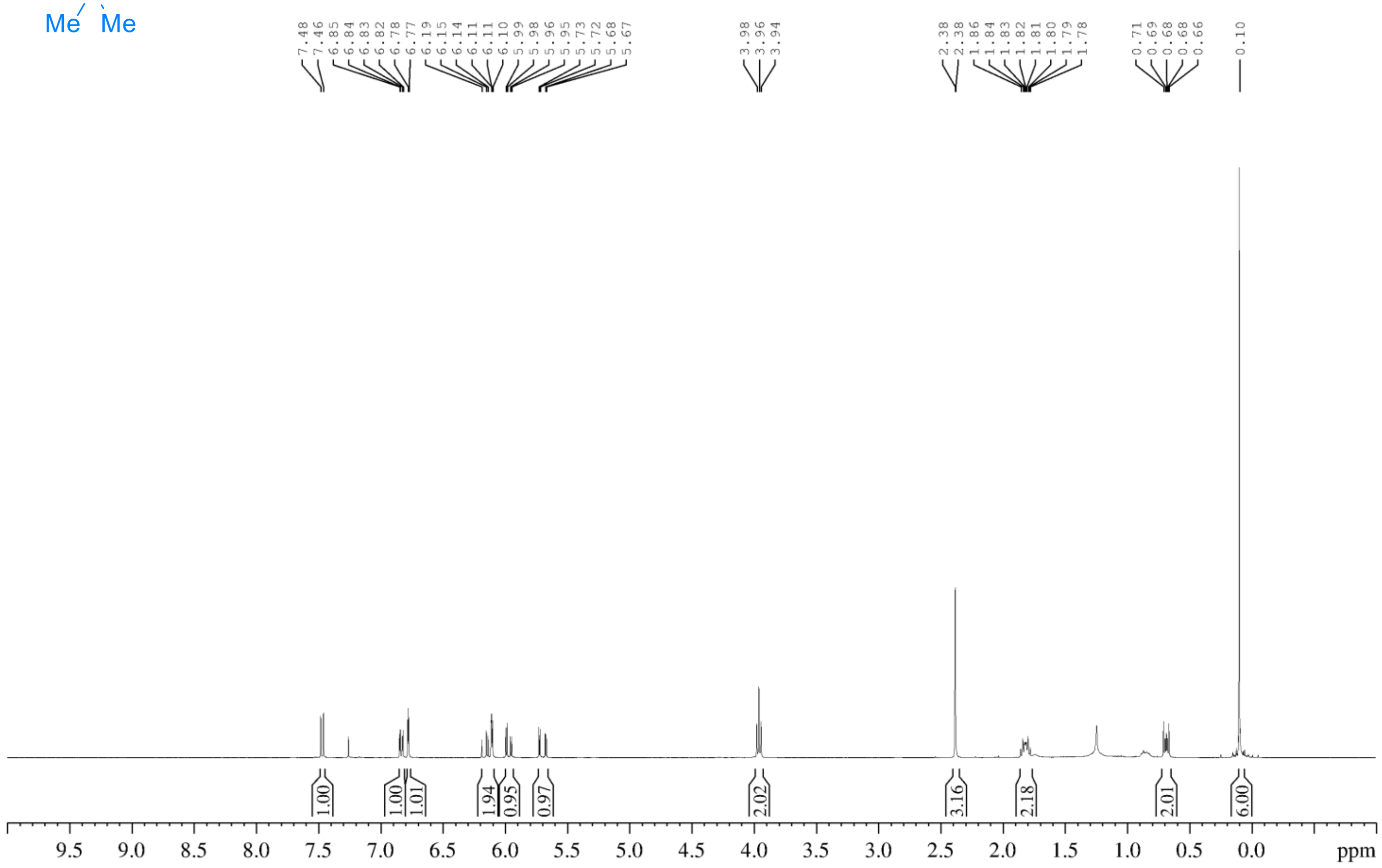

Figure S217. ^{13}C NMR (126 MHz, $CDCl_3$, 298 K) of **7ja**.

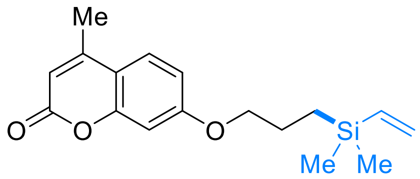

7ja

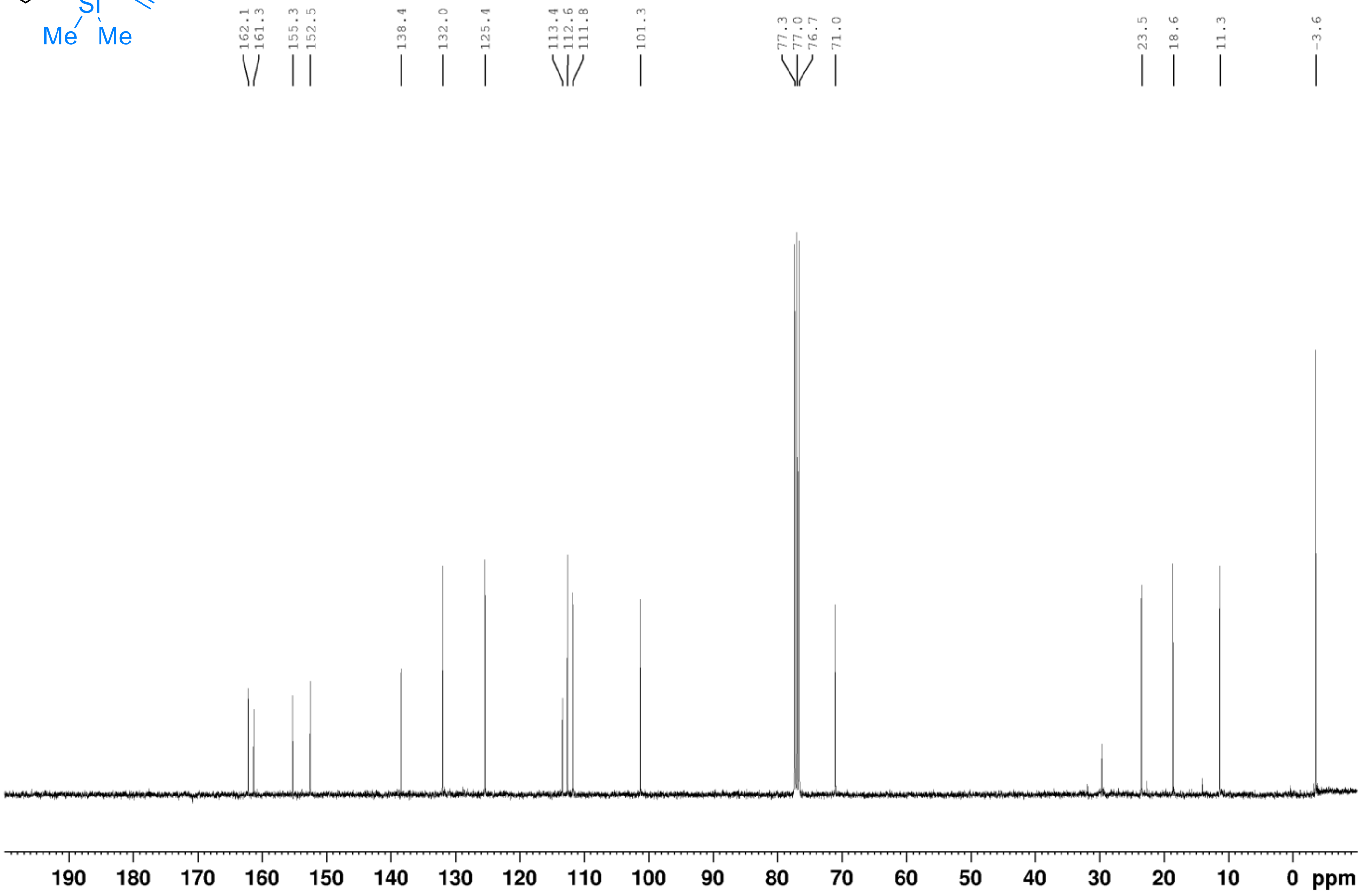

Figure S218. $^{1}H/^{29}Si$ HMQC NMR (500/99 MHz, $CDCl_3$, 298 K, optimized for J = 7 Hz) of **7ja**.

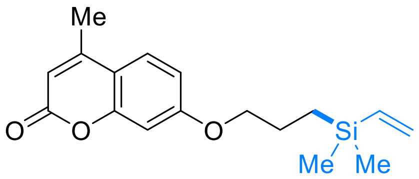

7ja

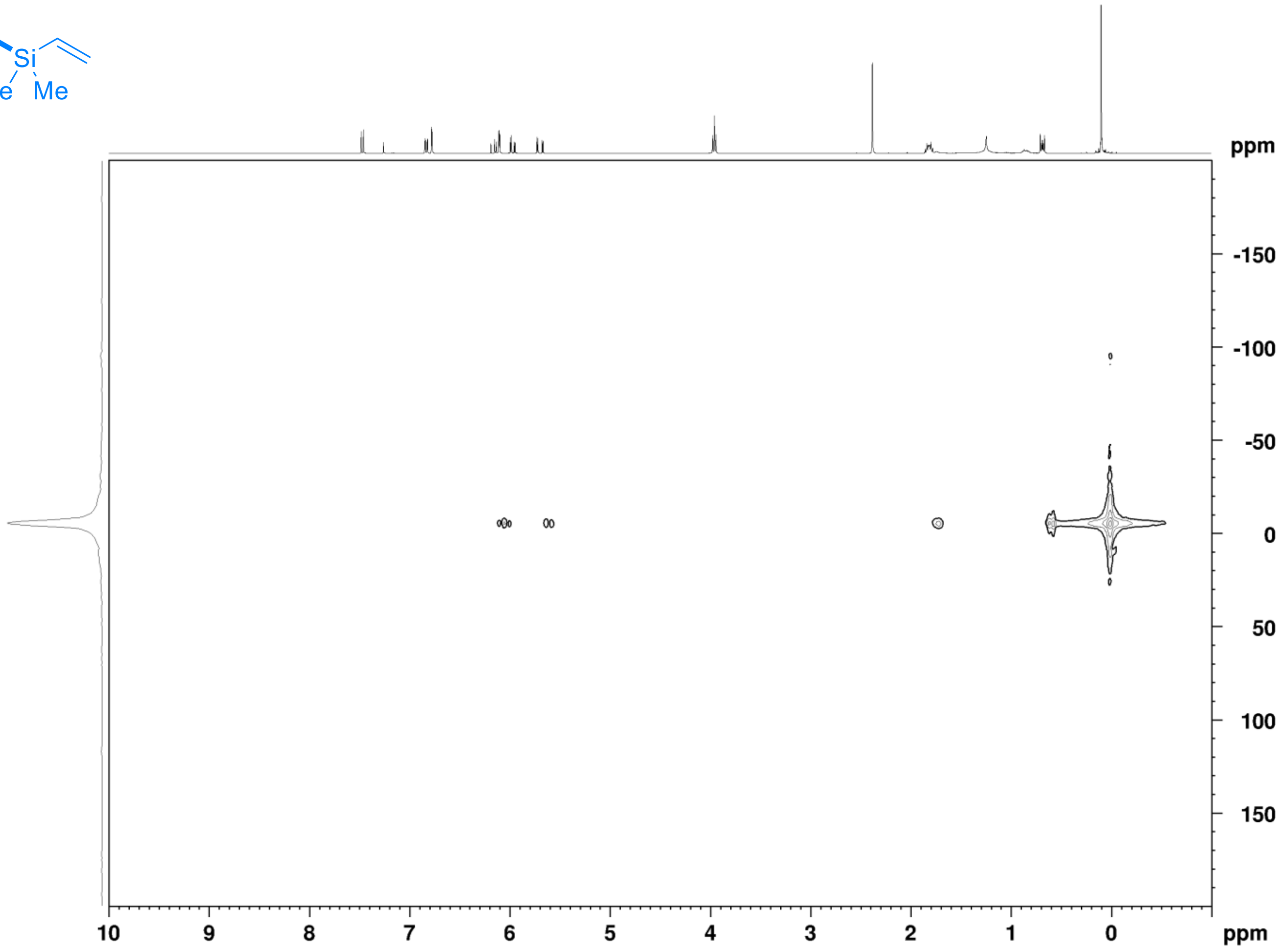

Figure S219. ^{1}H NMR (500 MHz, $CDCl_3$, 298 K) of **7ka**.

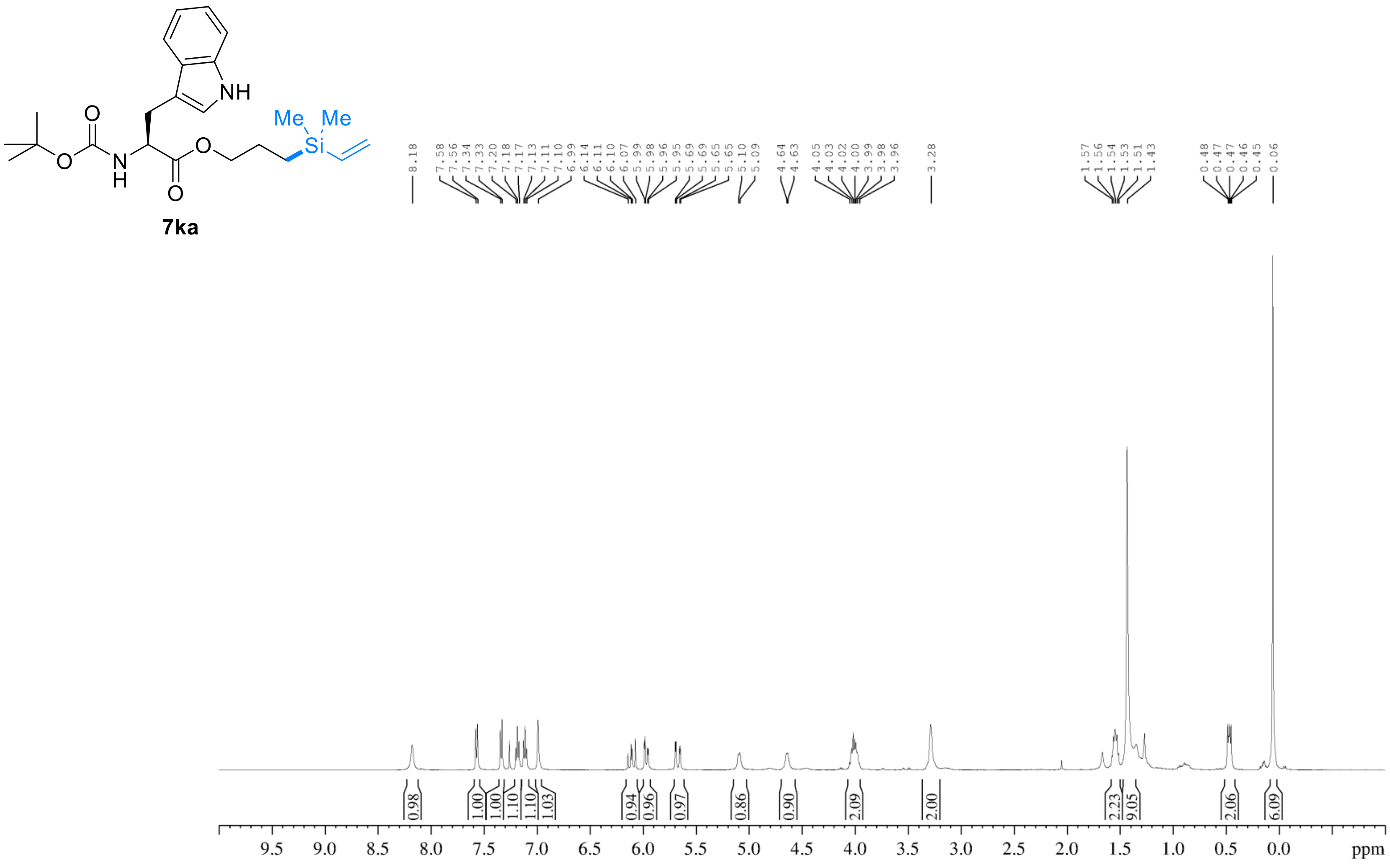

Figure S220. ^{13}C NMR (126 MHz, $CDCl_3$, 298 K) of **7ka**.

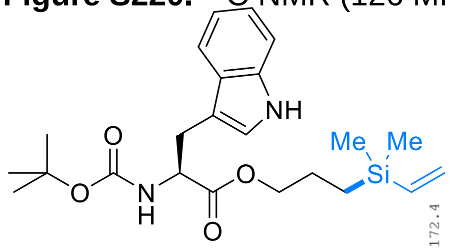

7ka

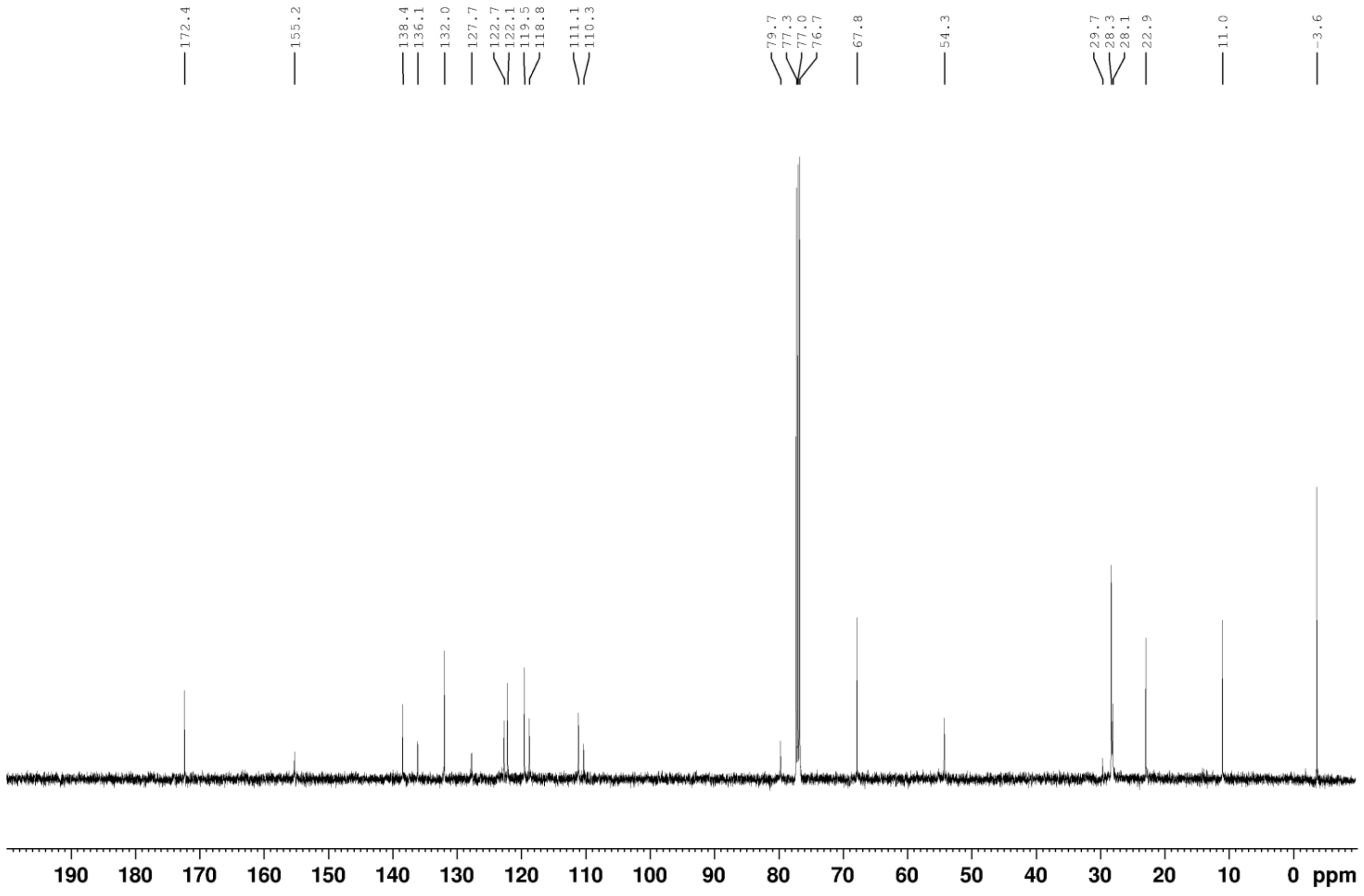

Figure S221. $^{1}H/^{29}Si$ HMQC NMR (500/99 MHz, $CDCl_3$, 298 K, optimized for J = 7 Hz) of **7ka**.

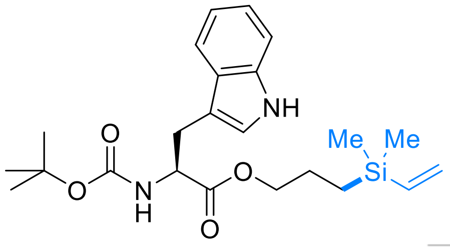

7ka

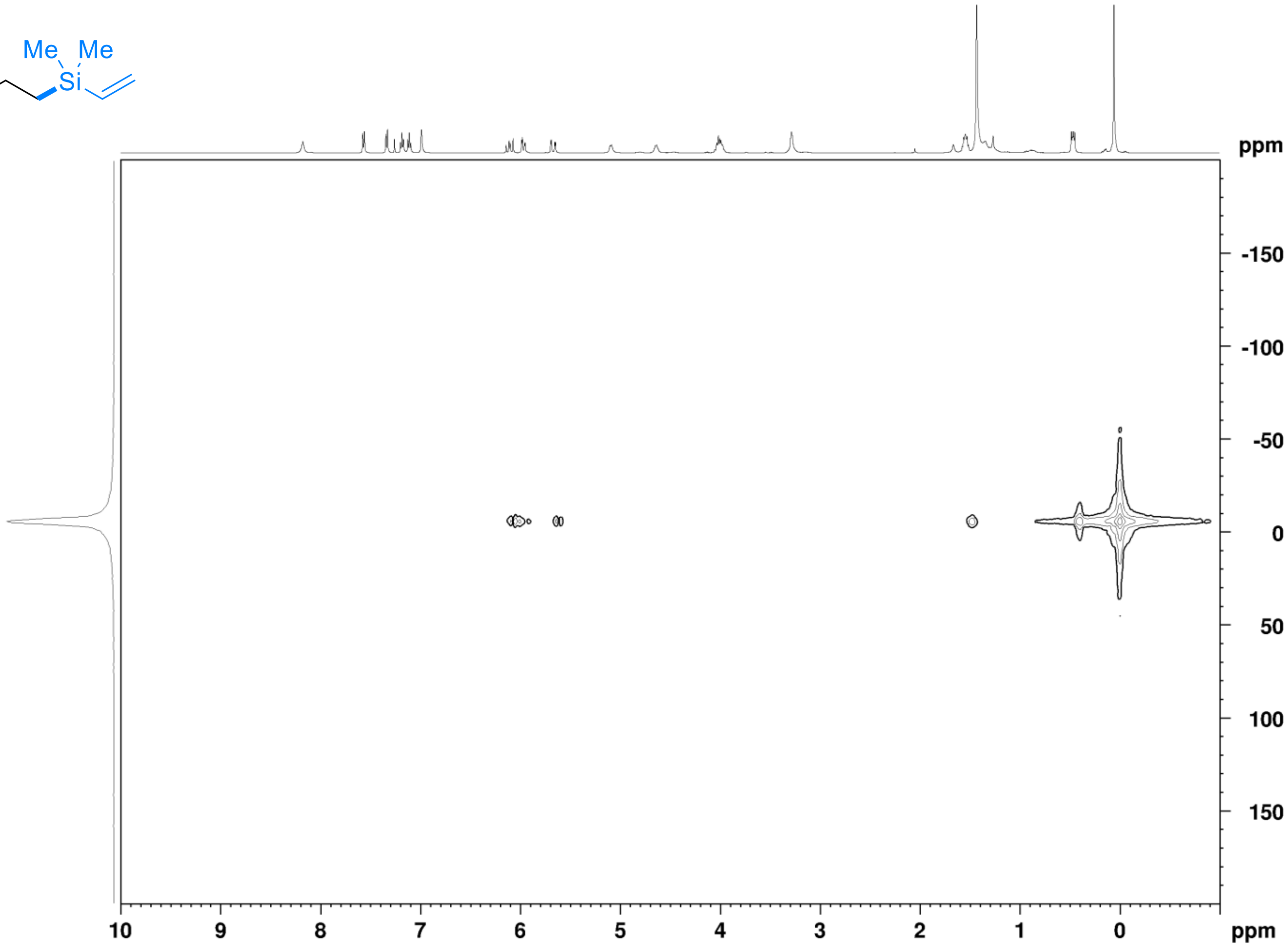

Figure S222. ^{1}H NMR (500 MHz, $CDCl_3$, 298 K) of **7la**.

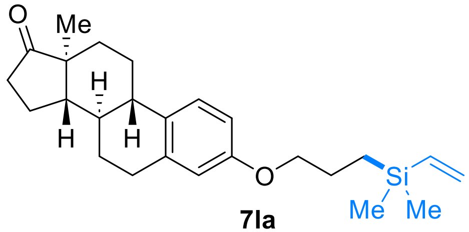

7la

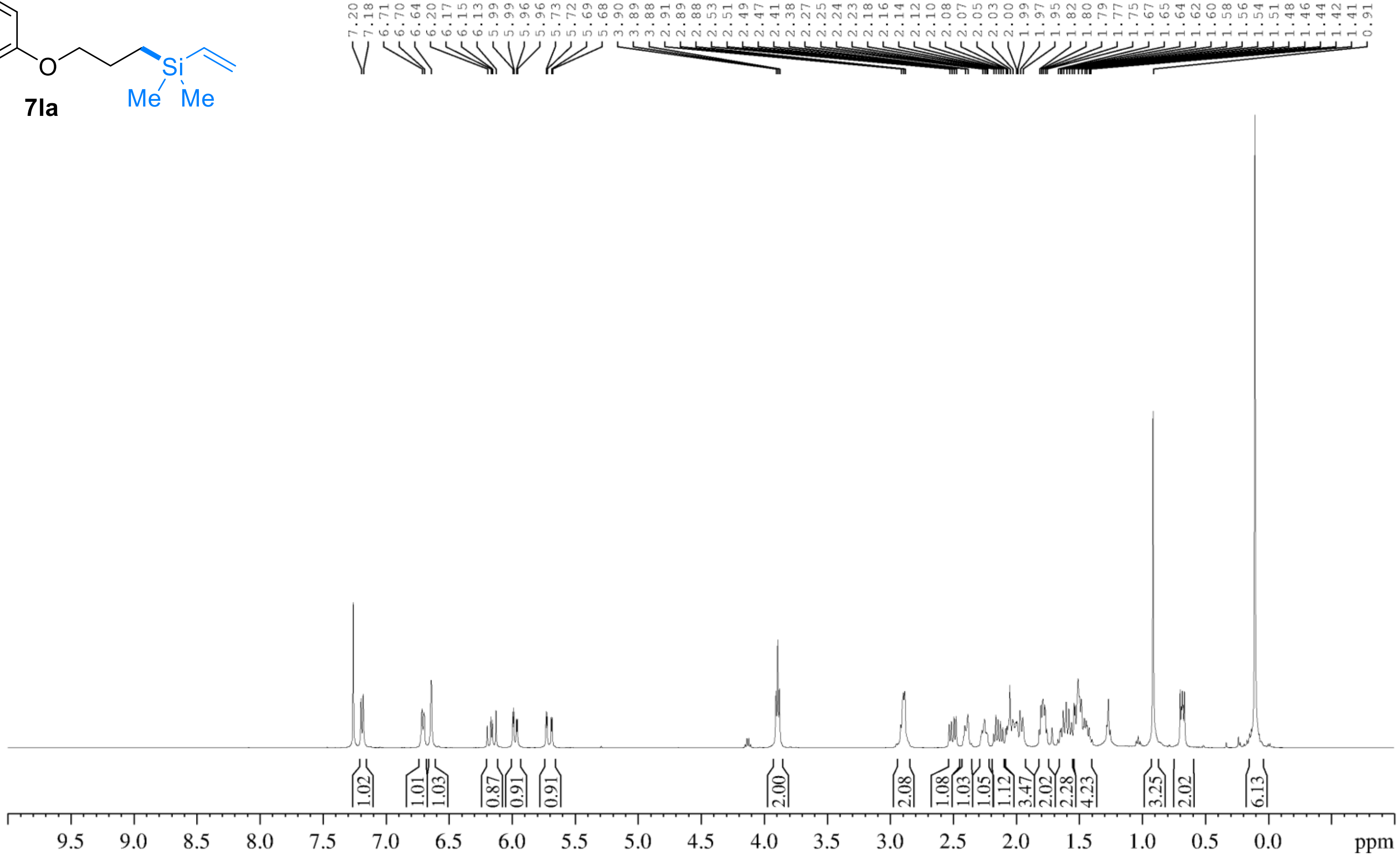

Figure S223. ^{13}C NMR (126 MHz, $CDCl_3$, 298 K) of **7la**.

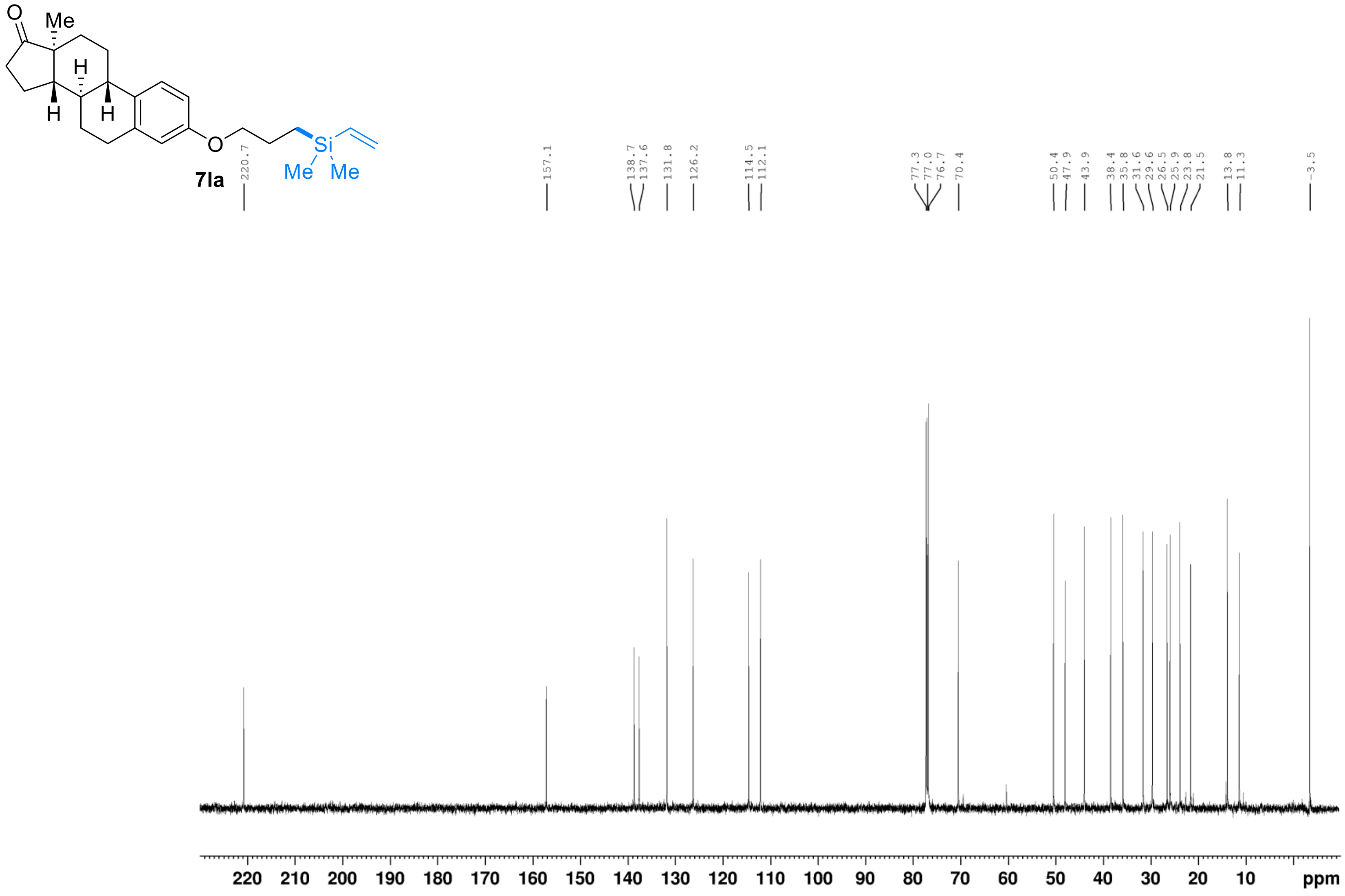

Figure S224. $^{1}H/^{29}Si$ HMQC NMR (500/99 MHz, $CDCl_3$, 298 K, optimized for J = 7 Hz) of **7la**.

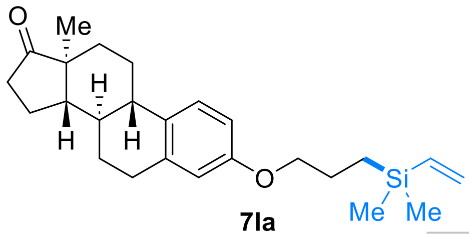

7la

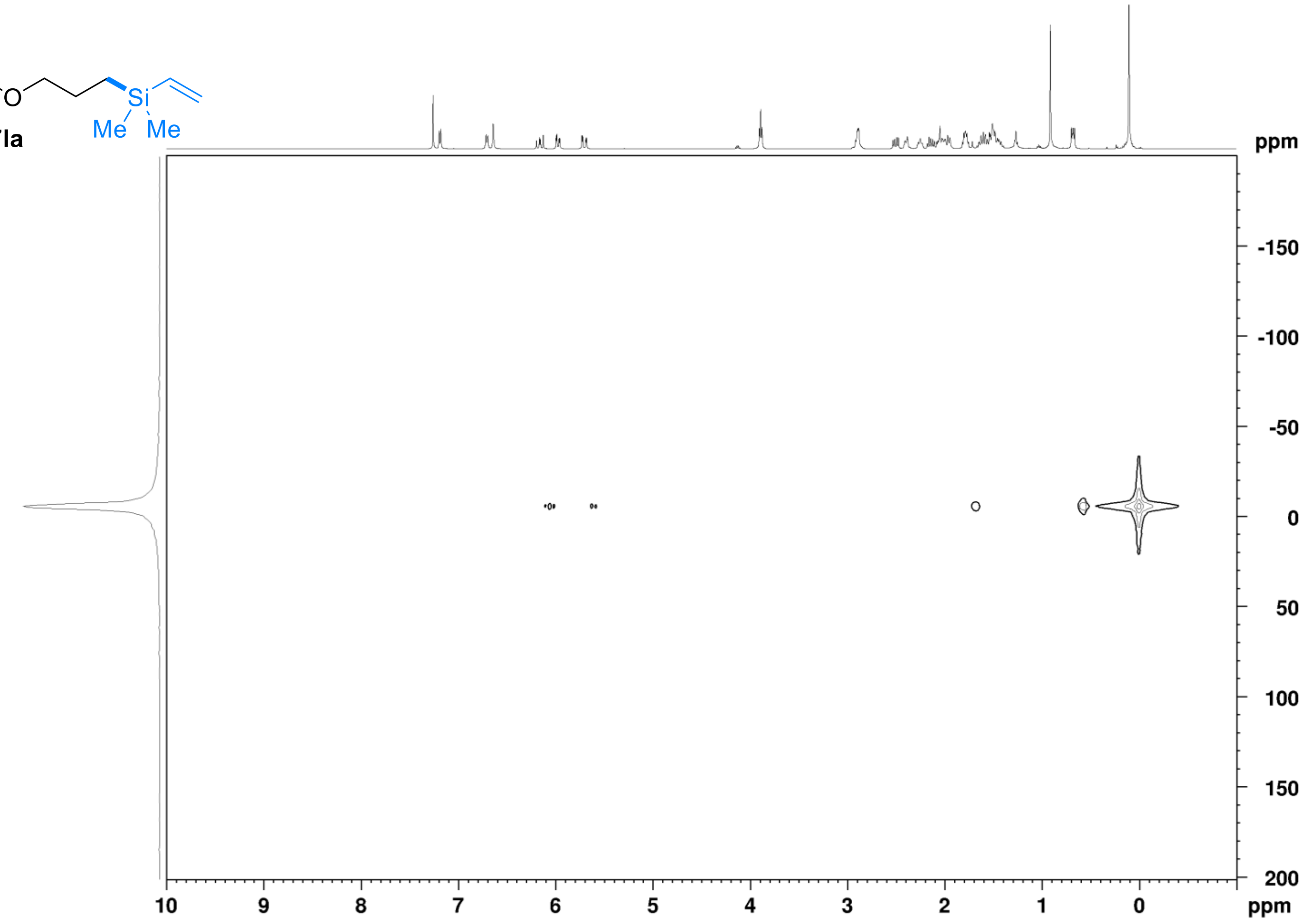

Figure S225. ^{1}H NMR (500 MHz, $CDCl_3$, 298 K) of **7ma**.

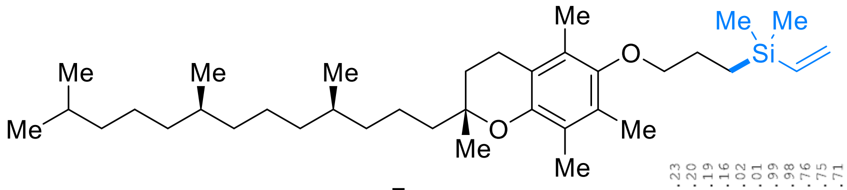

7ma

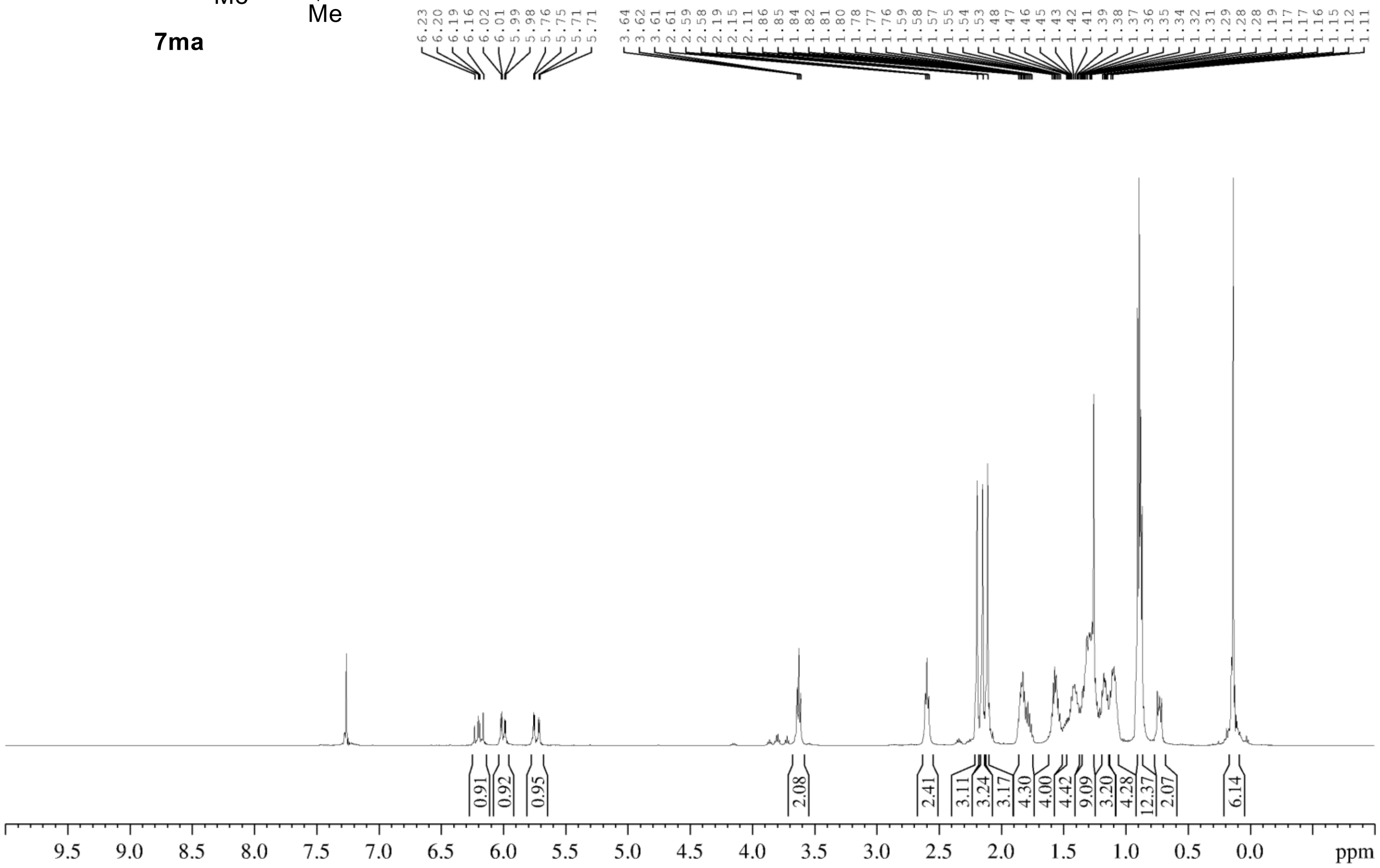

Figure S226. ^{13}C NMR (126 MHz, $CDCl_3$, 298 K) of **7ma**.

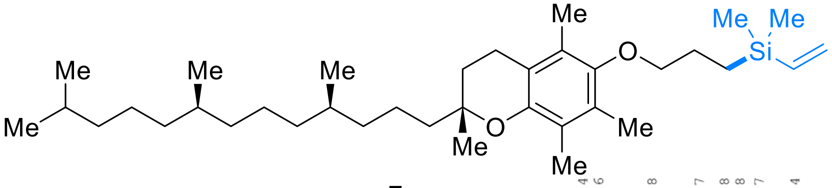

7ma

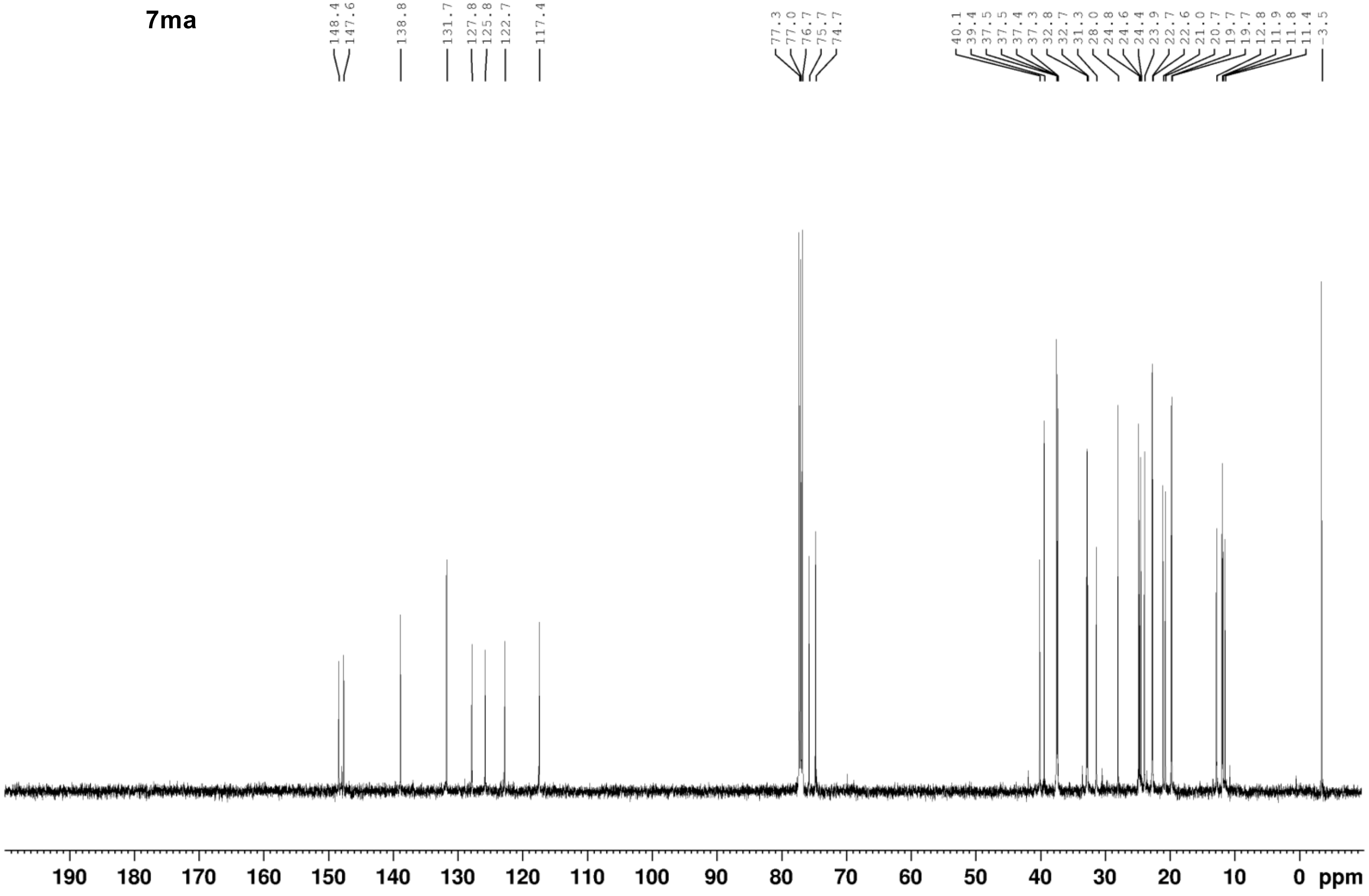

Figure S227. ^{1}H/^{29}Si HMQC NMR (500/99 MHz, $CDCl_3$, 298 K, optimized for J = 7 Hz) of **7ma**.

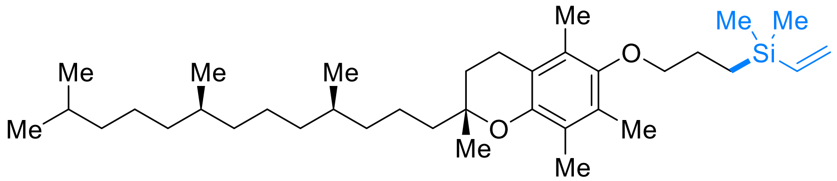

7ma

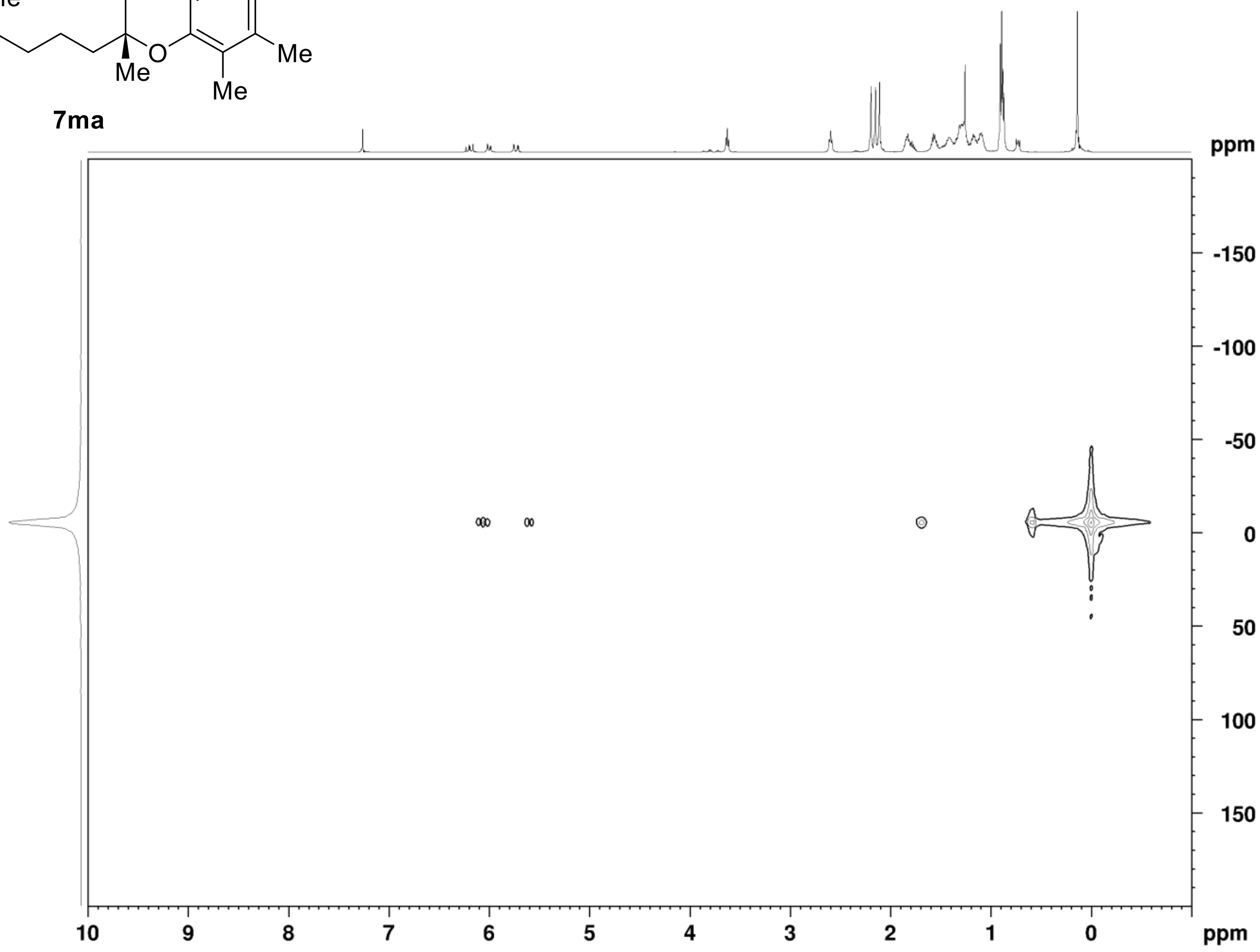

Figure S228. ^{1}H NMR (500 MHz, $CDCl_3$, 298 K) of **8a**.

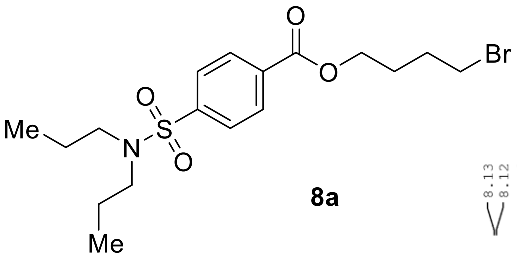

8a

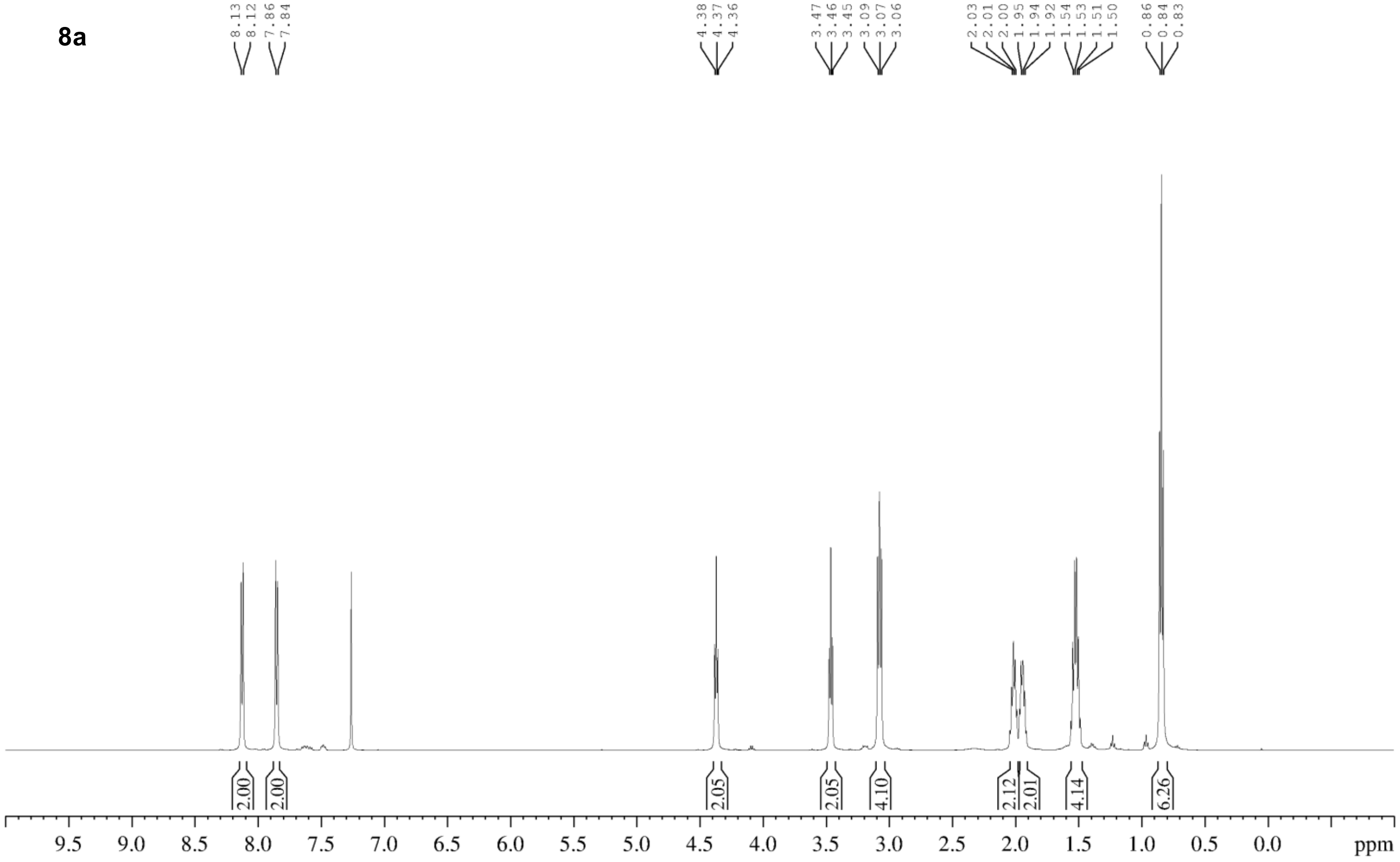

Figure S229. ^{13}C NMR (126 MHz, $CDCl_3$, 298 K) of **8a**.

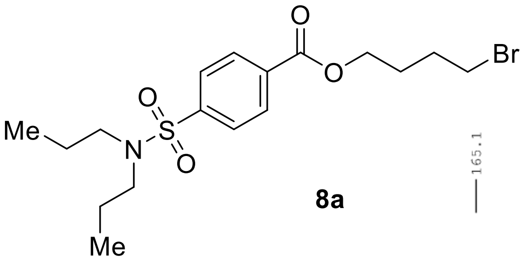
8a

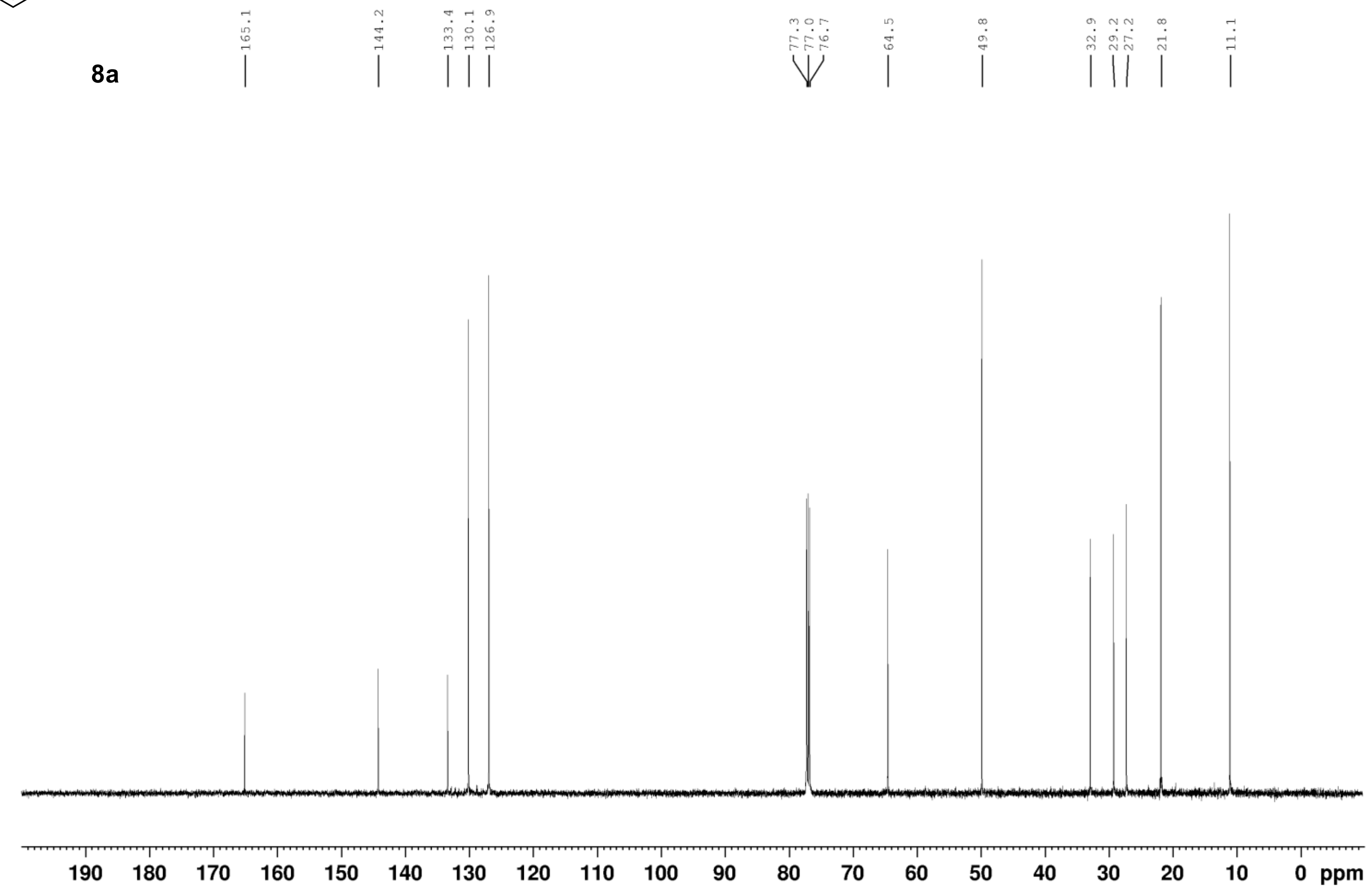

7 References

[S1] Harris, R. K.; Becker, E. D.; Cabral de Menezes, S. M.; Goodfellow, R.; Granger, P. NMR nomenclature. Nuclear spin properties and conventions for chemical shifts (IUPAC Recommendations 2001). *Pure Appl. Chem.* **2001**, *73*, 1795–1818.

[S2] (a) Wang, T.; Chen, F.; Qin, J.; He, Y. M.; Fan, Q. H. Asymmetric ruthenium-catalyzed hydrogenation of 2- and 2,9-substituted 1,10-phenanthrolines. *Angew Chem. Int. Ed.* **2013**, *52*, 7172–7176. (b) Pilli, R.; Chindan, B.; Rasappan, R. Unravelling the Mechanism of Nickel Mediated Cross-Electrophile-Electrophile Coupling Between Katritzky Salts and Acid Chlorides. *Eur. J. Org. Chem.* **2022**, e202200985.

[S3] (a) Hendrickson, J. B.; Schwartzman, S. M. Triphenyl phosphine ditriflate: A general oxygen activator. *Tetrahedron Lett.* **1975**, *16*, 277–280. (b) Chi, B. K.; Widness, J. K.; Gilbert, M. M.; Salgueiro, D. C.; Garcia, K. J.; Weix, D. J. In-Situ Bromination Enables Formal Cross-Electrophile Coupling of Alcohols with Aryl and Alkenyl Halides. *ACS Catal.* **2022**, *12*, 580–586.

[S4] Sanford, A. B.; Thane, T. A.; McGinnis, T. M.; Chen, P. P.; Hong, X.; Jarvo, E. R. Nickel-Catalyzed Alkyl-Alkyl Cross-Electrophile Coupling Reaction of 1,3-Dimesylates for the Synthesis of Alkylcyclopropanes. *J. Am. Chem. Soc.* **2020**, *142*, 5017–5023.

[S5] Itoh, T.; Mase, T. Practical thiol surrogates and protective groups for arylthiols for Suzuki-Miyaura conditions. *J. Org. Chem.* **2006**, *71*, 2203–2206.

[S6] Gericke, K. M.; Chai, D. I.; Lautens, M. The versatile role of norbornene in C–H functionalization processes: concise synthesis of tetracyclic fused pyrroles via a threefold domino reaction. *Tetrahedron* **2008**, *64*, 6002–6014.

[S7] Ohba Kiyomi, N. Y., Matsudaira Tetsuji, Hamada Maiko, Yamazaki Ryuta, Ibuki Tatsuya. Sulfonamide or sulfinamide compound having effect of inducing BRD4 protein degradation and pharmaceutical use thereof, EP 4105220A1. 2021.

[S8] Grimm, S. H.; Gagestein, B.; Keijzer, J. F.; Liu, N.; Wijdeven, R. H.; Lenselink, E. B.; Tuin, A. W.; van den Nieuwendijk, A.; van Westen, G. J. P.; van Boeckel, C. A. A.; et al. Comprehensive structure-activity-relationship of azaindoles as highly potent FLT3 inhibitors. *Bioorg. Med. Chem.* **2019**, *27*, 692–699.

[S9] Liu, L.; Zhu, T.; Cai, C.; Wang, L.; Li, S.; Lian, X.; Feng, Y.; Tian, Y.; Zhang, Q. Ligand-regulated three-photon AIE properties of manganese(II) complexes for photodynamic therapy. *Dalton Trans.* **2022**, *51*, 16915–16920.

[S10] He, B. Q.; Wu, X. Deuterium- and Electron-Shuttling Catalysis for Deoxygenative Deuteration of Alcohols. *Org. Lett.* **2023**, *25*, 6571–6576.

[S11] Duan, J.; Wang, K.; Xu, G. L.; Kang, S.; Qi, L.; Liu, X. Y.; Shu, X. Z. Cross-Electrophile C(sp^2)-Si Coupling of Vinyl Chlorosilanes. *Angew Chem. Int. Ed.* **2020**, *59*, 23083–23088.
